# Whole genome survey of big cats (Genus: *Panthera*) identifies novel microsatellites of utility in conservation genetic study

**DOI:** 10.1038/s41598-021-92781-0

**Published:** 2021-07-08

**Authors:** Jee Yun Hyun, Puneet Pandey, Kyung Seok Kim, Alvin Chon, Daecheol Jeong, Jong Bhak, Mihyeon Yu, Hye Kyung Song, Randeep Singh, Mi-Sook Min, Surendra Prakash Goyal, Damdingiin Bayarkhagva, Taisia Marchenkova, Anna Vitkalova, Hang Lee

**Affiliations:** 1 Conservation Genome Resource Bank for Korean Wildlife (CGRB), Research Institute for Veterinary Science and College of Veterinary Medicine, Seoul National University, Seoul, 08826 Republic of Korea; 2 Tiger and Leopard Conservation Fund in Korea, Seoul, 08826 Republic of Korea; 3 Amity Institute of Forestry and Wildlife, Amity University, Noida, 201313 India; 4 Department of Natural Resources Ecology and Management, Iowa State University, Ames IA, 50011 USA; 5 Department of Biomedical Engineering, UNIST, Ulsan, 44919 Republic of Korea; 6 Seoul Grand Park Zoo, Gwacheon, 13829 Republic of Korea; 7 Everland Zoological Garden, Yongin, 17023 Republic of Korea; 8 Wildlife Institute of India, Dehradun, 248001 India; 9 Department of the Biology, National University of Mongolia, Ulaanbaatar, 210646 Mongolia; 10 Land of the Leopard National Park, Barabash, Primorskiy-Kray, 692723 Russia

**Keywords:** Biological techniques, Computational biology and bioinformatics, Genetics, Molecular biology

## Abstract

Big cats (Genus: *Panthera*) are among the most threatened mammal groups of the world, owing to hunting, habitat loss, and illegal transnational trade. Conservation genetic studies and effective curbs on poaching are important for the conservation of these charismatic apex predators. A limited number of microsatellite markers exists for *Panthera* species and researchers often cross-amplify domestic cat microsatellites to study these species. We conducted data mining of seven *Panthera* genome sequences to discover microsatellites for conservation genetic studies of four threatened big cat species. A total of 32 polymorphic microsatellite loci were identified in silico and tested with 152 big cats, and were found polymorphic in most of the tested species. We propose a set of 12 novel microsatellite markers for use in conservation genetics and wildlife forensic investigations of big cat species. Cumulatively, these markers have a high discriminatory power of one in a million for unrelated individuals and one in a thousand for siblings. Similar PCR conditions of these markers increase the prospects of achieving efficient multiplex PCR assays. This study is a pioneering attempt to synthesise genome wide microsatellite markers for big cats.

## Introduction

The genus *Panthera* includes five hyper carnivorous apex predator species that are typically referred to as big cats^1–3^. These are the tiger (*Panthera* *tigris*), leopard (*Panthera pardus*), lion (*Panthera* *leo*), snow leopard (*Panthera* *uncia*), and jaguar (*Panthera* *onca*). Big cats are endangered and have great ecological, cultural, and historical significance, and thus needs to be conserved^4–7^. Major conservation challenges for these species include habitat loss, prey base decline, hunting, and illicit trade. From 1970 onward, several measures have been undertaken globally to fight the cause of falloffs. However, the success of such measures has been limited as these species continue to be listed among the IUCN (International Union for Conservation of Nature) endangered species^8–12^.

Incremental adoption of genetic tools and techniques for wildlife conservation and management have been observed globally in the past 25 years mainly due to the development of the robust protocols for DNA extraction and PCR (polymerase chain reaction)^13–16^. DNA tools are now increasingly employed for establishing species-level identity^17,18^, resolving taxonomic ambiguities^6,19,20^, wildlife conflict mitigation^21,22^, and more recently, establishing the source of origin^23–25^. Microsatellites or short tandem repeats (STR) are neutral, co-dominantly inherited, widely distributed, hypervariable, short repetitive nuclear DNA units that have been regarded as the best candidate to develop a genetic signature of the individual (DNA fingerprint), population, and subspecies^16,26–29^. Multiplex STR systems to undertake geographic assignments of confiscations have been proposed for tigers, leopards, elephants, rhinos and many other endangered species^23,25,30–33^. However, except for rhinos and elephants, microsatellite-based applications have failed to achieve global consensus in wildlife offense investigation. Efficient and simple protocols with established utilities in wildlife forensics across the range and species of rhinos and elephants have convinced wildlife managers and law enforcement agencies to adopt DNA methods for seizure investigations.

Tiger, leopard, lion, and snow leopard are the four most commercially exploited (by poaching and illegal trade) *Panthera* species. Their conservation demands stringent law enforcement. Here, we report the development of novel microsatellite markers for genus *Panthera* by mining the genome sequences of four (tiger, leopard, lion, and snow leopard) most exploited big cat species. This study is a part of an ongoing India–Korea–Russia collaborative initiative to develop and test microsatellite based multiplex PCR panels of the pantherine species for genetic identification of the whole genus *Panthera*.

## Results

### Abundance and distribution of STR in genomes of big cat species

We analysed the whole genome sequences of seven big cat individuals^34,35^ and found a total of 80,474,871 variant sites. These include SNVs (single nucleotide variants), indels, and microsatellites. Potential target variants were mined within these variant sites following the protocols described in the materials and methods section. Some of these variants were consistently polymorphic across all genomes, whereas some had limited polymorphism. Due to a large number of potential target variant candidates, we selected only those that were at least polymorphic in 5 of the 7 big cat genomes. Altogether, there were 8947 such potential target variants. Of these, 6283 were found to be located on unique sites in the genome (unique target variant, UTV). We found 2614 UTVs (Supplementary Table S3) in all seven genomes, and these were finally processed for microsatellite screening using the program MSDB^36^.

**Figure 1:**
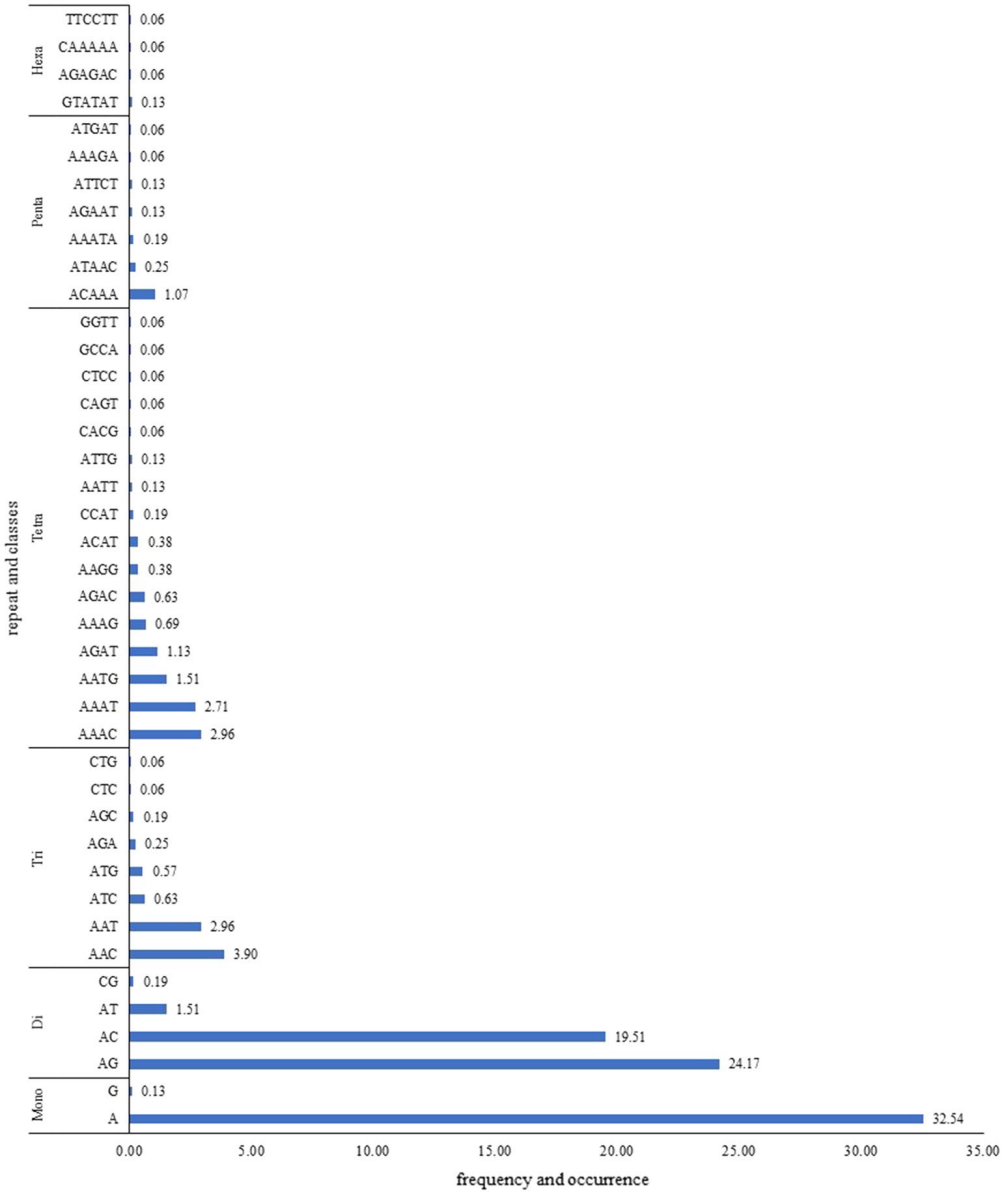
Frequency of occurrence of different STR repeat type classes across the *Panthera* genomes.

In big cat genomes, the dinucleotide microsatellite repeats were most abundant (45.4%), followed by mononucleotides (32.7%) and tetranucleotides (11.1%) (Fig. 1). The trinucleotides (8.6%), pentanucleotides (1.9%), and hexanucleotides (0.3%) were found in less abundance (Fig. 1). Relative abundance (mean number of STRs per Mb of genome analysed) was found to be the highest for Bengal tiger (357.3 STR/Mb) followed by white tiger (355.2 STR/Mb), Amur leopard (336.2 STR/Mb), Amur tiger (316.9 STR/Mb), white lion (312.3 STR/Mb), lion (310.7 STR/Mb), and snow leopard (304.4 STR/Mb).

Among all the mononucleotide repeats, (A)n was the most abundant (99.6%), while (C)n was comparatively scarce. In the dinucleotide repeat category, (AG)n and (AC)n were the two most frequent (96.3%) microsatellite motifs. Almost 80% of the trinucleotide types were (AAC)n, and (AAT)n in the *Panthera* genomes. Nearly half of the tetranucleotides were (AAAT)n and (AAAC)n. Among pentanucleotides, (ACAAA)n was the most abundant (56.7%). Hexanucleotides were the least among all types of microsatellites screened. The three most abundant microsatellite classes were (A)n, (AG)n, and (AC)n. Together they comprise 76.2% of the all forty-one microsatellite classes identified.

### Development of microsatellite markers for genus Panthera

Program batch primer 3 was used to design PCR primers^37^. About 4% of the UTVs were found suitable for primer design (i.e. sufficient flanking sequences and not single-copy sequences). These include 176 dinucleotides, 39 trinucleotides, 45 tetranucleotides, 11 pentanucleotides, and 3 hexanucleotides. The designed primer pairs for these loci were further screened based on GC content and the presence of secondary structures. Finally, primer pairs for 41 loci were shortlisted for oligonucleotide synthesis. PCR was subsequently attempted with the synthesised primer pairs with four DNA samples, one each of the tiger, leopard, lion, and snow leopard. Thirty-two microsatellite loci (Table 1) showed clear amplification in the expected size range and were considered further. The forward primers of these loci were fluorescently labelled with one of the four dyes—6FAM, VIC, NED, and PET. These labelled microsatellites were then used to genotype samples of tiger, leopard, lion, and snow leopard.

**Table 1:** Description of 32 novel microsatellite loci developed for genus *Panthera.*

Locus	Motif	Primer sequences	Annealing temperature	Allele range			
				Tiger	Leopard	Lion	Snow leopard
Pan1C2	(CA)n	F: CCTCAAGGTAACAGCAACA	61 °C	148–178	150–178	166–192	154–170
		R: TAGGCAAATCCAACTCACA					
Pan1D1	(TG)n	F: CCTACATCAACATAAACACACC	61 °C	184–194	184–186	184–188	182–188
		R: TCGGGCATACATCACTACA					
Pan1D2	(AG)n	F: AAAGGCATGGATACAGTCAG	61 °C	205–209	207–217	209–215	207–229
		R: GGTGGTTCAGTTGGTTAGG					
Pan10C2	(TG)n	F:ACTCCACTTGTCATCATTTGC	61 °C	147–163	147–155	151–153	147–165
		R: TAAGCCTCAGTTCCCTCCTAC					
Pan14C2	(CA)n	F: GCAAGAACTAAGACTCCAACC	61 °C	194–208	196–206	198–200	190–198
		R: TAAATGCCAGAGAGAATCCA					
Pan15C2	(CAA)n	F: TTCTGTAGGGTGTGGGTTC	61 °C	186–207	183–201	177–198	177–192
		R: AGTTCTTCTGGTGATGAGTGTC					
Pan16C2	(TTG)n	F: AAGTCAGGAGAAGATGGATG	61 °C	149–182	161–200	173–185	161–182
		R: GGCAAACTGAATAAAGGAGA					
Pan1A1	(TC)n	F: CTCCTTATTGTGACCCTGATT	61 °C	230–236	226–236	230–232	224–248
		R: AAACCAAACACCTGCTCTC					
Pan1A2	(AC)n	F: GCAGAGGAGGAGAGTATAGATTAG	61 °C	171–187	171–193	177 (M)	171–187
		R: TGAGTTTACATTGCCCAGA					
Pan1C1	(ATC)n	F: CTTTCTCTCCCTCTTTCTCTCTCT	61 °C	155–173	152–173	158–167	152–167
		R: ATGGTGCTTCCTGTGGTG					
Pan2D1	(GAAT)n	F: TCTTGGTTCCTTCCTCTGT	61 °C	123 (M)	123–135	131–143	127–135
		R: CTGCCCTATTCATTCATTC					
Pan2D2	(TG)n	F: ACCCACAGACAACCACAC	61 °C	122–156	118–154	124–152	120–140
		R: AGCAGTATCAATCCCATCAC					
Pan2A1	(TAT)n	F: AACCCAGAGCCCAACACA	61 °C	223–238	223–238	226–235	223–238
		R: GGTAGGAGGCACATAAAGAAACA					
Pan2C1	(CT)n	F: CTCCCATACCCTCACACA	61 °C	82–88	82–88	82–146	86 (M)
		R: GTTAGCCAGACGAGAGATG					
Pan3C2	(CT)n	F: ATCTGACCCTTATGAGTATGTGAG	61 °C	92–108	102–108	104–106	102–120
		R: ATGCCTTCCTACTAAATGACC					
Pan3D1	(CT)n	F: TCTTGTGGTTCGTGATTTG	61 °C	220–248	220–238	220–238	220–230
		R: GACTGCTTTGGCTATTTGAG					
Pan3D2	(TG)n	F: GTGCGTGTGTGTATCTGTG	61^O^C	158–182	154–174	154–158	160 (M)
		R: CAACTACGTGTGTGGTGAA					
Pan3A1	(AC)n	F: CTTGCTAATCCTGTGTTTGTC	61 °C	187–193	187–203	183–185	183–197
		R: CCCAGCATCCAAATATCA					
Pan3A2	(AGAC)n	F: TTTCTGATTCGGCCCTTT	61 °C	206–218	202–214	178–214	206 (M)
		R: CCTGAGATGGTTCCTGAGTTT					
Pan4D1	(TC)n	F: CTGTGTCTCCCTGTCTTTGT	61 °C	161–177	157–173	161–167	161–167
		R: TGTGCCTTTCTTCCATAGTT					
Pan4A1	(TG)n	F: TTTGGATTTCGTGTAGTGTG	61 °C	160–198	160–190	170–184	160–190
		R: AGAAGTGATTGGGATTGCT					
Pan4A2	(AACA)n	F: GAGAAGCATTACAAGAAGCA	61 °C	142–154	138–162	146–166	142–154
		R: CAGTCGTCACAGAAGGAAC					
Pan5D1	(AG)n	F: CTTTGTCTCTCAGCTCTTTGT	61 °C	143–153	139–151	141–159	145–163
		R: CCTTTGTCTTTCCAGTTCTC					
Pan5A1	(ATG)n	F: CTTCCTCATTCTCTTTGCTCTT	61 °C	183–195	183–213	171–192	189–204
		R: GCCACTGTTTATCCTCATTTCT					
Pan6C2	(GA)n	F: AGAGAAGCCAACCACAAA	61 °C	193–207	199–209	197–211	205–221
		R: GAGTTAGAGCCCACATCG					
Pan6A1	(CA)n	F: CCAAGTGTCCATCCAAAG	61 °C	145–165	143–171	147–163	145–159
		R: GCGTAATATCCTCTAGGTCAAA					
Pan6A2	(TC)n	F: ATTCTGTCTCTCTGCTCCTC	61 °C	123–127	123–133	143–153	123–129
		R: CCTTCCTCTTAGCTCTATTACCT					
Pan7C2	(TGA)n	F: GGCTCTATTCTATCCCTACACA	61 °C	200–209	188–206	173–176	197–200
		R: GTCTCCTTTCTTTCCTGGTC					
Pan7A1	(ATCT)n	F: TACATCCCTCCTTCCATCT	61 °C	165–193	165–185	157–161	161–185
		R: ATATTCCCAGTGCCTCCT					
Pan8C2	(AAT)n	F: GATTGTCTCTTTCTCTCCCTCT	61 °C	116–140	113–134	131–143	116–140
		R: TCAAACATTTCCTCCCACT					
Pan8A1	(AG)n	F: GGGTGAAGATGGTGTTGATAG	61 °C	149–171	145–161	157 (M)	153–161
		R: TTTCCCTGCCTCCTTATTT					
Pan9C2	(AAC)n	F: GGTAGGAGGTGGGAACAT	61 °C	214–226	214–226	223–229	214–226
		R: TCTGCTGATGACTTATTCTGAG					

*(M)* monomorphic loci.

### Microsatellite polymorphism evaluation

The fluorescently labelled microsatellites were used to genotype 152 big cat individuals. Overall, all loci were found to be polymorphic (4–18 alleles/locus), but some showed no variations within species—Pan2D1 in tiger; Pan1A2 and Pan8A1 in lion; and Pan3A2, Pan3D2, and Pan2C1 in snow leopard (Table 2). The species wise microsatellite characteristics and polymorphism are as follows:

**Table 2:** Characterizationof 32 polymorphic microsatellite loci in four big cat species.

Locus	Number of alleles									
	Tiger				Leopard				Lion	Snow leopard
	Overall	India	Russia	Korea	Overall	India	Russia	Korea		
**Pan10C2**										
*NA*	4	1	4	2	4	2	4	2	2	6
*HO*	0.02	0	0.1	0	0	0	0	0	0.29	0.25
*HE*	0.12	0	0.36	0.13	0.31	0.34	0.38	0.23	0.45	0.78
*PIC*	0.12	0	0.33	0.12	0.28	0.27	0.35	0.2	0.34	**0.7**
**Pan14C2**										
*NA*	7	4	3	6	6	5	3	6	2	4
*HO*	0.57	0.68	0.44	0.54	0.24	0.07	0.13	0.53	0	0.38
*HE*	0.69	0.74	0.58	0.66	0.66	0.67	0.25	0.73	0.11	0.69
*PIC*	**0.63**	**0.67**	0.49	0.59	**0.6**	**0.59**	0.23	**0.66**	0.1	**0.59**
**Pan15C2**										
*NA*	7	4	3	5	8	6	2	4	5	3
*HO*	0.47	0.53	0.63	0.33	0.41	0.46	0.07	0.8	0.53	0.14
*HE*	0.6	0.61	0.64	0.39	0.62	0.73	0.07	0.7	0.73	0.28
*PIC*	**0.55**	**0.51**	**0.52**	0.36	**0.58**	**0.66**	0.07	**0.6**	**0.66**	0.24
**Pan16C2**										
*NA*	8	6	1	6	7	6	4	3	4	5
*HO*	0.44	0.48	0	0.57	0.41	0.39	0.25	0.62	0.69	0.38
*HE*	0.67	0.73	0	0.65	0.74	0.79	0.51	0.56	0.68	0.73
*PIC*	**0.61**	**0.67**	0	**0.58**	**0.69**	**0.72**	0.46	0.43	**0.59**	**0.64**
**Pan1A1**										
*NA*	4	3	2	3	5	4	3	2	2	7
*HO*	0.42	0.19	0.86	0.5	0.22	0.29	0.07	0.27	0.47	0.5
*HE*	0.49	0.46	0.53	0.47	0.49	0.69	0.2	0.33	0.48	0.69
*PIC*	0.42	0.4	0.37	0.37	0.45	**0.61**	0.19	0.27	0.36	**0.63**
**Pan1A2**										
*NA*	5	2	1	4	8	6	5	6	1	4
*HO*	0.04	0.05	0	0.04	0.57	0.46	0.47	0.75	0	0.13
*HE*	0.21	0.33	0	0.14	0.85	0.81	0.57	0.84	0	0.44
*PIC*	0.2	0.27	0	0.14	**0.82**	**0.75**	**0.51**	**0.78**	0	0.39
**Pan1C1**										
*NA*	6	4	5	2	7	5	5	4	3	3
*HO*	0.09	0.14	0.2	0	0.35	0.36	0.47	0.2	0.53	0
*HE*	0.71	0.66	0.44	0.43	0.72	0.73	0.64	0.49	0.5	0.43
*PIC*	**0.66**	**0.59**	0.4	0.33	**0.67**	**0.67**	**0.57**	0.45	0.41	0.37
**Pan1C2**										
*NA*	10	6	4	6	12	9	4	6	4	4
*HO*	0.32	0.4	0.2	0.29	0.52	0.58	0.53	0.44	0.29	0.13
*HE*	0.65	0.8	0.28	0.35	0.88	0.83	0.72	0.83	0.51	0.44
*PIC*	**0.62**	**0.76**	0.26	0.33	**0.86**	**0.78**	**0.64**	**0.78**	0.45	0.39
**Pan1D1**										
*NA*	3	2	2	1	2	1	2	2	2	3
*HO*	0.04	0.13	0	0	0.05	0	0	0.15	0.69	0
*HE*	0.22	0.44	0.19	0	0.18	0	0.14	0.37	0.51	0.43
*PIC*	0.2	0.34	0.16	0	0.16	0	0.12	0.29	0.37	0.37
**Pan1D2**										
*NA*	3	2	1	3	3	2	2	3	3	3
*HO*	0.04	0	0	0.07	0.07	0	0	0.19	0.71	0
*HE*	0.11	0.1	0	0.14	0.41	0.49	0.24	0.45	0.68	0.55
*PIC*	0.1	0.09	0	0.13	0.34	0.36	0.2	0.39	**0.58**	0.45
**Pan2A1**										
*NA*	5	2	3	4	6	6	3	5	4	4
*HO*	0.26	0.14	0.22	0.37	0.45	0.25	0.47	0.63	0.63	0.25
*HE*	0.49	0.13	0.52	0.58	0.81	0.7	0.54	0.74	0.7	0.52
*PIC*	0.41	0.12	0.44	0.47	**0.77**	**0.63**	0.45	**0.67**	**0.62**	0.44
**Pan2C1**										
*NA*	4	2	2	3	4	3	3	3	4	1
*HO*	0.02	0	0	0.04	0.18	0.09	0.08	0.38	0.11	0
*HE*	0.12	0.08	0.19	0.14	0.43	0.32	0.22	0.61	0.3	0
*PIC*	0.12	0.07	0.16	0.13	0.39	0.28	0.2	**0.51**	0.28	0
**Pan2D1**										
*NA*	1	1	1	1	4	1	4	2	2	2
*HO*	0	0	0	0	0	0	0	0	0.46	0
*HE*	0	0	0	0	0.25	0	0.38	0.24	0.37	0.23
*PIC*	0	0	0	0	0.23	0	0.35	0.2	0.29	0.2
**Pan2D2**										
*NA*	10	7	4	5	14	11	5	5	8	4
*HO*	0.45	0.46	0.78	0.29	0.48	0.61	0.13	0.73	0.73	0.13
*HE*	0.81	0.78	0.61	0.5	0.73	0.86	0.24	0.65	0.84	0.44
*PIC*	**0.79**	**0.73**	0.5	0.46	**0.71**	**0.83**	0.22	**0.56**	**0.79**	0.39
**Pan3A1**										
*NA*	4	3	2	3	6	3	5	3	2	5
*HO*	0.27	0.33	0.5	0.13	0.07	0	0.12	0.07	0	0.86
*HE*	0.55	0.4	0.53	0.51	0.39	0.57	0.23	0.26	0.33	0.79
*PIC*	0.45	0.34	0.38	0.44	0.35	0.46	0.21	0.23	0.27	**0.69**
**Pan3A2**										
*NA*	4	3	2	3	4	4	2	3	3	1
*HO*	0.3	0.35	0.38	0.24	0.18	0.13	0	0.4	0.07	0
*HE*	0.5	0.43	0.53	0.49	0.28	0.19	0.14	0.48	0.2	0
*PIC*	0.46	0.38	0.37	0.42	0.26	0.18	0.12	0.41	0.19	0
**Pan3C2**										
*NA*	7	6	2	4	6	3	4	3	2	4
*HO*	0.31	0.41	0	0.3	0.31	0.43	0.27	0.25	0	0.25
*HE*	0.6	0.73	0.23	0.48	0.65	0.54	0.4	0.41	0.12	0.35
*PIC*	**0.57**	**0.68**	0.2	0.44	**0.58**	0.46	0.35	0.35	0.11	0.31
**Pan3D1**										
*NA*	11	8	3	6	6	3	3	3	2	4
*HO*	0.64	0.61	0.43	0.73	0.05	0	0	0.13	0.06	0.13
*HE*	0.78	0.78	0.56	0.75	0.3	0.37	0.28	0.23	0.06	0.53
*PIC*	**0.74**	**0.73**	**0.46**	**0.69**	0.29	0.33	0.26	0.22	0.06	0.46
**Pan3D2**										
*NA*	11	8	3	7	6	3	3	5	2	1
*HO*	0.64	0.68	0.38	0.7	0.26	0.42	0	0.38	0.13	0
*HE*	0.81	0.76	0.63	0.79	0.41	0.48	0.23	0.53	0.13	0
*PIC*	**0.78**	**0.71**	**0.52**	**0.74**	0.39	0.41	0.22	0.47	0.11	0
**Pan4A1**										
*NA*	12	5	8	7	8	4	5	5	6	7
*HO*	0.59	0.53	0.8	0.54	0.34	0.25	0.13	0.6	0.79	0.71
*HE*	0.86	0.74	0.86	0.67	0.48	0.44	0.25	0.67	0.7	0.89
*PIC*	**0.84**	**0.67**	**0.8**	**0.59**	0.45	0.39	0.24	**0.61**	**0.64**	**0.8**
**Pan4A2**										
*NA*	4	2	1	3	5	3	2	3	2	3
*HO*	0.07	0.04	0	0.1	0.32	0.19	0.2	0.56	0.06	0.13
*HE*	0.06	0.04	0	0.1	0.53	0.5	0.19	0.49	0.06	0.34
*PIC*	0.06	0.04	0	0.1	0.44	0.41	0.16	0.39	0.06	0.29
**Pan4D1**										
*NA*	7	6	2	4	9	6	4	4	4	2
*HO*	0.49	0.5	0.14	0.58	0.4	0.19	0.13	0.93	0.5	0.2
*HE*	0.73	0.72	0.36	0.53	0.79	0.72	0.45	0.7	0.46	0.2
*PIC*	**0.68**	**0.65**	0.28	0.45	**0.74**	**0.66**	0.41	**0.62**	0.41	0.16
**Pan5A1**										
*NA*	4	2	3	3	7	6	3	4	3	5
*HO*	0.05	0	0.2	0.04	0.43	0.44	0.53	0.31	0.29	0.25
*HE*	0.13	0.07	0.28	0.14	0.58	0.67	0.5	0.52	0.53	0.45
*PIC*	0.13	0.07	0.25	0.13	**0.52**	**0.6**	0.41	0.44	0.43	0.4
**Pan5D1**										
*NA*	6	5	2	5	6	5	5	4	6	6
*HO*	0.4	0.56	0	0.38	0.35	0.19	0.29	0.63	0.35	0.29
*HE*	0.63	0.73	0.21	0.5	0.52	0.5	0.41	0.61	0.66	0.75
*PIC*	**0.59**	**0.67**	0.18	0.46	0.48	0.46	0.37	**0.53**	**0.6**	**0.66**
**Pan6A1**										
*NA*	10	10	3	9	11	9	5	5	6	5
*HO*	0.62	0.7	0.63	0.55	0.48	0.5	0.47	0.47	0.41	0.29
*HE*	0.81	0.85	0.66	0.79	0.79	0.83	0.46	0.79	0.37	0.73
*PIC*	**0.78**	**0.81**	**0.54**	**0.75**	**0.76**	**0.78**	0.42	**0.72**	0.35	**0.63**
**Pan6A2**										
*NA*	3	3	2	3	6	5	3	2	5	3
*HO*	0.42	0.5	0.43	0.35	0.23	0.25	0.18	0.25	0.38	0.13
*HE*	0.57	0.65	0.54	0.47	0.52	0.71	0.33	0.23	0.63	0.24
*PIC*	0.48	**0.56**	0.38	0.37	0.47	**0.63**	0.28	0.2	**0.55**	0.22
**Pan6C2**										
*NA*	5	3	1	5	5	4	2	3	3	3
*HO*	0.32	0.47	0	0.31	0	0	0	0	0.11	0.13
*HE*	0.63	0.62	0	0.56	0.35	0.49	0.14	0.33	0.11	0.24
*PIC*	**0.57**	**0.52**	0	**0.5**	0.33	0.45	0.12	0.29	0.1	0.22
**Pan7A1**										
*NA*	8	6	3	7	6	5	4	5	2	3
*HO*	0.71	0.76	0.38	0.78	0.62	0.5	0.69	0.67	0.2	0.13
*HE*	0.82	0.8	0.58	0.83	0.74	0.65	0.68	0.62	0.19	0.43
*PIC*	**0.78**	**0.75**	0.45	**0.78**	**0.69**	**0.6**	**0.6**	**0.53**	0.16	0.35
**Pan7C2**										
*NA*	4	4	3	4	5	3	4	3	2	3
*HO*	0.44	0.35	0.88	0.39	0.25	0	0.4	0.4	0.06	0.33
*HE*	0.61	0.39	0.68	0.61	0.34	0.21	0.4	0.43	0.34	0.32
*PIC*	**0.53**	0.35	**0.56**	**0.53**	0.32	0.19	0.35	0.37	0.27	0.27
**Pan8A1**										
*NA*	3	1	1	3	3	2	2	2	1	2
*HO*	0.02	0	0	0.05	0	0	0	0	0	0
*HE*	0.1	0	0	0.21	0.25	0.44	0.13	0.25	0	0.67
*PIC*	0.09	0	0	0.19	0.23	0.33	0.12	0.22	0	0.38
**Pan8C2**										
*NA*	7	5	2	6	4	3	2	3	3	4
*HO*	0.42	0.61	0.13	0.32	0.03	0	0	0.09	0.62	0.14
*HE*	0.71	0.64	0.33	0.55	0.55	0.58	0.37	0.18	0.59	0.5
*PIC*	**0.66**	**0.6**	0.26	**0.51**	0.44	0.46	0.29	0.16	0.47	0.43
**Pan9C2**										
*NA*	4	4	1	3	5	4	3	2	3	3
*HO*	0.12	0.15	0	0.14	0.04	0.08	0	0	0.33	0.38
*HE*	0.27	0.27	0	0.35	0.26	0.3	0.23	0.23	0.59	0.51
*PIC*	0.26	0.25	0	0.32	0.25	0.27	0.22	0.2	0.48	0.43

*NA* Number of alleles, *HO* observed heterozygosity, *HE* expected heterozygosity, *PIC* Polymorphic Information Content (>0.5 -in bold).

#### Tiger (*Panthera tigris*)

We genotyped 67 tiger individuals of wild and captive origin. They were collected from India (n = 27), Russia (n = 11), and South Korea (n = 29, zoo individuals).Twenty-one (India—3, Korea zoo—20, and Russia—0) of 32 loci deviated significantly from HWE (Hardy–Weinberg Equilibrium) after bonferroni correction (adjusted p-value < 0.002, Supplementary Table S2), and null alleles were detected in 27 loci (India—15, Korea zoo—21, and Russia—11; threshold limit of 10%, Supplementary Table S2). Mean allelic diversity and gene diversity was found 6 (1–12 allele/locus) and 0.50 (0.00–0.86). Allelic diversity was found highest for tigers sampled from South Korean zoos (Amur tiger, 4.3 allele/locus), followed by Indian tigers (Bengal tiger, 4.1 allele/locus), and Russian tigers (Amur tiger, 2.5 allele/locus). Overall, the markers were found to be polymorphic (except Pan2D1) with a mean polymorphic information content (PIC) of 0.46. Fifteen, sixteen, and seventeen markers were found to have PIC ≥ 0.5 in tigers sampled from Russia, Korea (zoo), and India, indicating their informative nature and utility in conservation genetic studies (Table 2).

#### Leopard (*Panthera pardus*)

A total of 59 individuals belonging to the wild (India and Russia) and captivity (South Korea) were genotyped. Overall, markers were polymorphic in leopards with mean allelic diversity of 6.2 (2–14 alleles/locus) and average expected heterozygosity of 0.52 (0.18–0.88). Nine (Pan2A1, Pan2D2, Pan4D1, Pan5D1, Pan6A2, Pan6C2, Pan8C2, Pan9C2, and Pan14C2), seven (Pan1A2, Pan1C1, Pan1C2, Pan1D2, Pan5D1, Pan6A1, and Pan6C2) and three (Pan2D1, Pan9C2, and Pan10C2) loci deviated significantly from HWE after bonferroni correction (adjusted p-value < 0.002, Supplementary Table S2) in leopard sampled from India, Korea (zoo), and Russia respectively. Null alleles (≥ 10%) were detected in 23, 21, and 18 loci in leopards sampled from India, Russia, and Korea (zoo) (Supplementary Table S2). Thus, there is high probability of discovery of additional alleles in these developed markers, if tested with a greater number of samples. Thirteen of the 32 markers were found suitable for conservation genetic studies with PIC ≥ 0.5 (Table 2).

#### Lion (*Panthera leo*)

A total of 18 captive African lions from Korean zoos were genotyped. Out of 32 loci, 2 were monomorphic and 30 were polymorphic loci, with the number of alleles ranging from 1 to 8 (mean = 3.2). The mean expected heterozygosity was 0.4 (0.00–0.84) for lions. We did not observe any significant deviation from HWE after bonferroni correction (adjusted p-value < 0.002) in any loci (Supplementary Table S2). Null alleles were detected in 9 loci (≥ 10%, Supplementary Table S2). The mean polymorphic information content was estimated to 0.35, with 8 loci having PIC > 0.5 (Table 2).

#### Snow leopard (*Panthera uncia*)

Snow leopards (n = 8) were sampled from the wild (Mongolia) and zoo (Korea). All these samples were considered as a single population during genetic analysis as there were not enough samples from the wild or captivity to be considered as distinct populations. Moreover, Korean zoos sourced snow leopards from Mongolia.

In twenty-nine polymorphic microsatellites, the number of the alleles ranged from 2 to 7 (mean = 3.9), with mean expected heterozygosity of 0.5 (0.2–0.89). Locus Pan10C2 showed a significant deviation from HWE after bonferroni correction (adjusted p-value < 0.002, Supplementary Table S2). Null alleles were detected in 23 loci (≥ 10%, Supplementary Table S2). The mean polymorphic information content was 0.4 with eight loci having PIC > 0.5 (Table 2).

### Establishment of a universal microsatellite marker system for big cat species

This study aims to propose a universal microsatellite marker system capable of undertaking individual identification and geographic assignments of big cat seizures. We understand that the loci with higher expected heterozygosity (He) are more useful for individual identification. Similarly, loci with PIC values higher than 0.5 are considered informative enough for estimating genetic diversity. In our study, the locus wise heterozygosity and PIC varied across the species. We selected twelve microsatellite loci based on the comparative marker’s PIC, heterozygosity, and allele diversity (Table 3). These loci showed no signs of linkage disequilibrium (LD) with big cats’ wild populations. The average PIC of 12 markers was 0.45, 0.50, 0.63, and 0.66 for the lion, snow leopard, leopard, and tiger, respectively. The cumulative power of discrimination among unrelated individuals (P_ID_) was found to be 1.03 × 10^–8^, 8.6 × 10^–12^, 1.6 × 10^–12^, and 7.2 × 10^–15^ for lion, leopard, tiger, and snow leopard, respectively, using the recommended panel of 12 microsatellites. Similarly, the cumulative power of discrimination among siblings (P_ID_ sib) was found to be 1.1 × 10^–3^, 6.3 × 10^–4^, 7.6 × 10^–5^, and 3.7 × 10^–5^ for the lion, snow leopard, leopard, and tiger respectively.

**Table 3:** Probability of identity for unrelated samples (P_ID_) and for full siblings (P_ID_ sib) in 12 microsatellite loci.

Locus	Tiger		Leopard		Lion		Snow leopard	
	P_ID_	P_ID_ sib	P_ID_	P_ID_ sib	P_ID_	P_ID_ sib	P_ID_	P_ID_ sib
Pan6A1	5.19E−02	3.63E−01	6.25E−02	3.78E−01	3.53E−01	6.76E−01	3.84E−02	4.50E−01
Pan2D2	5.11E−02	3.62E−01	8.17E−02	4.12E−01	3.42E−02	3.60E−01	1.78E−01	6.36E−01
Pan1C2	1.40E−01	4.65E−01	2.39E−02	3.23E−01	2.49E−01	5.79E−01	1.78E−01	6.36E−01
Pan5D1	1.63E−01	4.84E−01	2.50E−01	5.61E−01	1.14E−01	4.73E−01	1.34E−03	4.34E−01
Pan14C2	1.48E−01	4.48E−01	1.78E−01	4.80E−01	7.78E−01	8.99E−01	8.74E−02	4.71E−01
Pan4A1	3.28E−02	3.34E−01	2.79E−01	5.87E−01	1.02E−01	4.48E−01	5.30E−03	3.50E−01
Pan3D1	7.42E−02	3.83E−01	4.91E−01	7.30E−01	8.66E−01	9.41E−01	1.14E−01	5.77E−01
Pan1C1	1.25E−01	4.33E−01	1.09E−01	4.26E−01	2.96E−01	5.95E−01	2.14E−01	6.44E−01
Pan2A1	3.32E−01	5.93E−01	6.43E−02	3.69E−01	1.41E−01	4.51E−01	1.42E−01	5.85E−01
Pan7A1	5.73E−02	3.61E−01	9.86E−02	4.12E−01	6.48E−01	8.32E−01	2.49E−01	6.52E−01
Pan15C2	2.01E−01	5.08E−01	1.67E−01	4.92E−01	1.05E−01	4.30E−01	4.00E−01	7.65E−01
Pan16C2	1.53E−01	4.59E−01	1.01E−01	4.12E−01	1.53E−01	4.69E−01	2.79E−02	4.45E−01
Cumulative	1.59E−12	3.66E−05	8.64E−12	7.62E−05	1.03E−08	1.13E−03	7.21E−15	6.28E−04

### Microsatellite multiplexing

The novel microsatellites were optimized in 8 multiplex PCRs (Table 4) to achieve cost effectiveness. The data quality remained similar in both singleplex and multiplex PCRs. Using multiplex PCRs, the DNA requirement was reduced to 25% and hence was found more efficient, especially with the fecal samples.

**Table 4:** Multiplex PCRs (4 microsatellite in each PCR).

	Primer name	Dye Label	Motif		Primer name	Dye Label	Motif
MPP1	Pan6A1	6FAM	CA	MPP5	Pan3C2	6FAM	CT
	Pan7C2	VIC	TGA		Pan3D1	VIC	CT
	Pan2A1	NED	TAT		Pan1D2	NED	AG
	Pan1A1	PET	TC		Pan3A2	PET	AGAC
MPP2	Pan1C2	6FAM	CA	MPP6	Pan9C2	6FAM	AAC
	Pan4A1	VIC	TG		Pan14C2	VIC	CA
	Pan7A1	NED	ATCT		Pan1D1	NED	TG
	Pan8C2	PET	AAT		Pan15C2	PET	CAA
MPP3	Pan5D1	6FAM	AG	MPP7	Pan5A1	6FAM	ATG
	Pan3D2	VIC	TG		Pan4A2	VIC	AACA
	Pan6A2	NED	TC		Pan3A1	NED	AC
	Pan16C2	PET	TTG		Pan4D1	PET	TC
MPP4	Pan6C2	6FAM	GA	MPP8	Pan2D2	6FAM	TG
	Pan2D1	VIC	GAAT		Pan2C1	VIC	CT
	Pan1C1	NED	ATC		Pan10C2	NED	TG
	Pan1A2	PET	AC		Pan8A1	PET	AG

## Discussion

Even with the development of more sophisticated and elaborate markers such as SNPs, microsatellites are still considered the best tool to study conservation genetics due to their codominant inheritance pattern and hypervariability. There are two kinds of microsatellites—species-specific and heterologous. The former is developed for a species of interest, while the latter is screened from a pool of STR loci that were previously described for other species. Geneticists have used both species-specific and heterologous microsatellites to study the genetic diversity and population structures of big cats^15,16,29,38–40^. However, the use of heterologous markers is more prevalent due to the availability of a limited number of species-specific STRs. Mishra et al. compared the polymorphism of species-specific vs. cross-specific markers in Bengal tiger and concluded the former’s superiority over the latter^41^. Moreover, the chances of genotyping errors due to mispriming, false alleles, and null alleles are lesser with species-specific STRs. In this study, the genome sequences of seven big cat individuals belonging to four species were analysed rapidly to identify and develop thirty-two polymorphic loci. The procedure of microsatellite development involved four steps: (1) mapping of big cat genomes on the assembled reference genome of the domestic cat to develop a multiple sample construct, (2) screening of the unique variant sites from the multiple sample construct, (3) scanning of unique variants to identify the polymorphic STR loci with conserved flanking regions, and (4) designing of PCR primers for these loci and evaluation of polymorphism with the collected samples. Since the whole process involved comparative genome analysis and selection of universally located STRs with conserved flanking regions, the developed microsatellite markers were regarded as species-specific for all the four target big cat species. This makes our study a pioneering attempt to develop microsatellite markers for a genus. In this study, we used Felcat6.2 genome assembly that is less recent compare to Felcat8.0 and Felcat9.0. Though the newer versions are more accurate and comprehensive and may provide additional in silico candidate sites as they were created using latest sequencing platform, but there is no guarantee they are better in any way as different sequencing platform output draw similar conclusions regardless of the sequencing platform and bioinformatics pipeline. In fact, less recent genome assemblies are typically more diverse and 'stable' since they were created using older technologies^42^. The autosomal location of each marker was assigned based on the karyotype of the domestic cat as its karyotype is reported to be similar to that of *Panthera* species. The microsatellite markers were named according to the genus *Panthera* (Pan) and autosome location (A1, A2, D1, etc., Table 1). For example, Pan10C2, Pan14C2, Pan15C2, and Pan16C2 are markers located on chromosome C2 in all Panthera species. Microsatellites were found to be located on six of the eighteen autosomal chromosomes, thereby ensuring at least 33% genome coverage.

We developed fluorescently labelled primer pairs for 32 novel microsatellite loci. Their polymorphism potential was evaluated with the DNA samples of four big cat species. All markers amplified successfully and produced scorable profiles with tiger, lion, leopard, and snow leopard. All markers were found polymorphic in leopards. Pan2D1 in tiger, Pan1A2, and Pan8A1 in lion and Pan3A2, Pan3D2, and Pan2C1 in snow leopard were monomorphic. Mean allelic diversity was found highest for leopards followed by tiger, snow leopard, and lion (Table 2). The evidence of null alleles in several locus suggests that more alleles may be discovered. No sign of HWE deviation was observed in tested lion population and only one locus (Pan10C2) deviated in snow leopard (SupplementaryTable S2). However, we reported significant deviation from HWE in several loci in tiger (India—3, Korean zoo—20), and leopard (Russia—3, India—9, and Korean zoo—7) (Supplementary Table S2). This could have resulted due to pooling of samples of different subspecies or populations into one group (Wahlund effect) or the analysis of first-degree relatives. Both are possible in our case as we sampled captive individuals and pooled samples based on broad geographical limits for Indian tigers and leopards. Moreover, we did not report any loci deviating from HWE in Russian tigers (sampled from LLNP, Russia), and few loci deviation in Russian leopard (sampled from LLNP, Russia) and Snow leopard (sampled from Mongolia). Therefore, we recommend further evaluation of these novel markers with more samples before drawing a conclusion about their polymorphism potential.

Microsatellite polymorphism levels vary greatly across populations and species. Markers with PIC greater or around 0.5 were considered suitable for genetic studies. Seventeen markers in tiger, thirteen in leopard and 8 each in lion and snow leopard had PIC values greater than the threshold (Table 2).

Identification of affected species, the responsible perpetrators, and their methods of killing are important aspects of wildlife forensic investigations. However, wildlife managers are only interested in the information about the affected species and population (source). Knowledge of the origin of the confiscated wildlife helps in the initiation of remedial actions in a timely manner. Microsatellite markers are great tools for the scientists and technicians involved in the investigation of wildlife poaching and trade cases. Microsatellite-based genetic IDs are useful to ascertain the number of affected (killed) individuals. The same information can then be used to reveal the source population (geographic assignment).

Tigers are the most illegally traded big cat species. In the past few decades, the increasing substitution of tiger parts with that of other big cat species has been observed. Except for pelt, commercially traded parts of big cats such as claw, bone, whisker, meat, canine, etc. are morphologically indistinguishable at the species level. In 2015, Mondol et al. successfully demonstrated the use of microsatellite markers to infer the source of origin of the leopard seizures from India^30^. Similarly, Zou et al. proposed a panel of microsatellites for tigers to identify individuals and subspecies^31^. In both studies, researchers generated a microsatellite-based genetic signature of all candidate populations (or subspecies) on their own, as the available information in the published domain was incompatible due to the use of different STR loci. Thus, to ensure the adoption of the microsatellite-based approach in forensic investigations, there is a need for the use of a unified DNA typing methodology for individual identification and establishment of genetic signatures. Moreover, the use of an established and universal methodology is more convincing during court proceedings. Here, we proposed a universal microsatellite panel for four big cat species that are most affected by illegal trade and are often traded with the same covert identity. The panel includes 12 microsatellite loci, distributed over five chromosomes. Cumulatively, these markers have a high discriminatory power of one in a million for unrelated individuals and one in a thousand for siblings (Table 3). Such a high degree of discriminatory power also makes this panel suitable for population genetic studies. In the wild, more than two big cat species often inhabit the same region or country simultaneously (e.g., tiger, leopard, lion, and snow leopard in India; lion and leopard in Africa; tiger, leopard, and snow leopard in Russia). The universal marker system for all the big cat species will reduce the necessary reagent cost and technical burden of researchers working on different big cat species in a laboratory or a network of laboratories. This will also promote data exchange and cooperative research. The similar range of annealing temperatures of primers (Table 1) for the markers in this study was useful in developing a multiplex PCR system. Our 8 multiplex PCRs showed good amplification success and genotype profile quality was found comparable to singleplex PCRs. Besides, since the markers are developed by mining the polymorphic STR loci with conserved flanking regions using the assembled genomic sequence of the domestic cat as the reference sequence, most of the markers have the potential to be applied to a variety of other endangered cat species. Hence, the proposed microsatellite panel is of great utility in establishing DNA fingerprints, population signatures, and wildlife forensics.

## Materials and methods

### Sample collection and DNA preparation

We analyzed the biological samples of tiger, leopard, lion, and snow leopard belonging to nature reserves, zoos, and sample repositories of India, Mongolia, Russia, and South Korea (Supplementary Table S1). These include blood, muscle, faeces, shed hair, and DNA extracts. The study does not involve any experiments with live animal. Blood and tissue samples used in this study were indirectly (previously collected for other studies/purposes) obtained for the purpose of this study. Therefore, ethical clearance regarding sample collection is not applicable to our study.

All samples were legally and ethically collected by partner institutions [South Korea—Conservation Genome Resource Bank for Korean Wildlife (Seoul National University, Seoul), India—Wildlife Institute of India (Dehradun) and Amity University (Uttar Pradesh), Mongolia (National University of Mongolia, Ulaanbaatar), and Russia (Land of the Leopard National Park, Primorsky Krai)], and wherever applicable, the necessary permissions and permits were obtained from competent authorities (Supplementary Table S1). DNA extraction, PCR and DNA fingerprinting were undertaken in the source country except for the Russian tiger and leopard samples for which CITES permit was obtained for DNA import to South Korea (ES2019-03989).

Commercial column-based DNA extraction kits (Qiagen’s QIAamp DNA mini kit and QIAamp DNA stool mini kit) were employed to extract DNA following the recommended protocols. The whole process was carried out in a sterile environment of a dedicated laboratory to avoid any chance of contamination. Further, a positive and a negative control per experimental setup were included. Post extraction, DNA was resolved on 0.8% agarose gel to assess quality and quantity. Finally, the DNA was preserved at − 20 °C for long term storage. The species identity of each of the sourced samples were re-verified using conservation genetic tools i.e., amplifying either species-specific primers^43,44^ or by sequence analysis of Cyt b gene using universal primers^45^.

### Microsatellite development for genus Panthera

In our study, we analysed previously published genome sequences of seven big cat individuals^46,47^. These include three tigers, two lions, a leopard, and a snow leopard. Additionally, we downloaded the assembled genome of domestic cat, Felcat6.2^48^, that served as a reference.

Each genome was processed independently for the variant calling. The FASTQ reads of the individual genome were mapped on the assembled reference genome (Felcat6.2) with the BWA-MEM^49^ using the default options. Duplicates were marked using Picard Tools. Thereafter, the variant sites were assessed using the Samtoolsmpileup^50^ and consensus sequences were generated for each species. A multiple sample construct was developed to make the genomes of different species comparable and to identify the variable sites. Samples without variants at the position were assigned the reference allele with the related coverage from the sample. The variants were then filtered based upon the following criteria: no heterozygous status for any sample, depth greater than or equal to 4 for all samples at that position (DP ≥ 4), and the number of different alleles among all the samples present should be greater than a specified value (like 3, 4, 5, or 6 unique alleles) out of the possible total. The resulting variants were considered as the potential target variants. These were then parsed for unique sites since it is possible to have variants called from different samples at the same site. The unique target variant sites were then expanded to ± 150 bp around the sites to create 301 bp regions for downstream primer design. The nucleotide sequence of the *Felis catus* reference at those covered regions was extracted by BEDTools^51^, and variant sites were replaced with the longest allele from all possible alleles at the site.

The program MSDB^36^ was used to screen the perfect STR repeats of 1–6 bp having a minimum repeat number of 12, 7, 5, 4, 4, and 4 for mono-, di-, tri-, tetra-, penta-, and hexa-nucleotide microsatellites respectively, from the unique target variant sequences. The repeats were classified into classes based on their start position and reverse complements. For example, TGG contains TGG, GGT, GTG, ACC, CCA, and CAC in different reading frames or on complementary strands. Microsatellite average length, total counts, frequency (loci/Mb), and density (loci/bp) of the motif were analysed^36^. The sequences of microsatellite repeat regions that passed the selection criteria were used to design the primer sets using software Batch Primer 3^37^. The loci with long enough flanking regions (i.e., more than 20 bp) and with no single copy sequences were shortlisted for primer design. Further scanning was done using Clustal X1.83^52^ to ensure that the microsatellite should not be published earlier. The criteria for searching of the primers were as follows: (1) PCR product should range from 80 to 250 base pair considering the utility of developed markers with samples yielding low quality DNA, (2) primers melting temperature (Tm) should range from 52 to 62 °C (optimal 55 °C), (3) primer GC content should range from 40 to 60%, and (4) number of returns i.e. number of primer pairs generated for each unique target variant sequence should be four. The rest of the parameters were set to default.

Non-labelled primer pairs were synthesised for loci qualifying the primer designing and selection criteria. These primers were subsequently tested for PCR amplification with one sample each of tiger, leopard, lion, and snow leopard. Gradient PCR (annealing temperature, T_a_—52–62 °C, reaction volume—10 µL and primer concentration—5 pm each) was performed independently for each primer pair. Primer pairs producing a single product band of expected size during PCR amplification were shortlisted for fluorescent dye labelling (forward primers) with one of four fluorescent dyes (6-FAM, VIC, NED, or PET, Invitrogen, South Korea) to perform fragment analysis using Applied Biosystems 3130 Genetic Analyser. During primer dye-labelling, due consideration was given to avoid dye range and product size overlap.

### Microsatellite polymorphism evaluation

Fluorescently labelled microsatellites were tested for their polymorphism potential in an independent PCR assay with 152 samples of big cats. In a reaction, the total volume was 10 µl, with 30–35 ng of extracted DNA, 1× PCR buffer, 0.25 mM dNTP mix, 0.5 U of i-StarTaq™ DNA polymerase (iNtRON Biotechnology, Inc), and 0.4 µM of each forward and reverse primer. The thermal profile of the amplification was as follows: initial denaturation at 94 °C for 2 min, followed by 40 cycles of denaturation at 94 °C for 40 s, annealing at 61 °C for 40 s, extension at 72 °C for 45 s, with one cycle of final extension for 30 min at 72 °C. The amplified PCR products were checked on 2% agarose, diluted (1:20, except scat DNA PCR products) with distilled water, pooled based on dye label and product size, and subjected to fragment analysis with an Applied Biosystems 3130 Genetic Analyzer. The alleles were scored with Gene Mapper 3.7 (Applied Biosystems).

During analysis, the samples were classified into sets: (1) based on species—4 populations, and (2) based on species and geographic origin—8 populations (Supplementary Table S1). The microsatellite data was analysed for possible genotyping errors of scoring and stuttering with MicroChecker 2.2.3^53^. Conformance with HWE and level of LD were assessed using Genepop 1.2^54^. The p-values for HWE and LD were corrected for multiple comparisons by applying a sequential Bonferroni correction^55^. Null allele frequencies were determined with the Dempsters EM method implemented in Genepop 1.2^54^. The software CERVUS was used to calculate the locus wise observed and expected frequency of alleles and heterozygosity, and the PIC for each population^56,57^. Allele range was calculated for each of the markers by compiling the observed allele range of all species. Program Gimlet 1.3.3 was used to estimate P_ID_ for unrelated samples and more conservative P_ID_ sib to test the discriminatory power of sets with a different number of markers.

### Microsatellite multiplexing

The microsatellites developed were optimized into multiplex PCRs to achieve efficiency and cost effectiveness. Program Multiplex manager was used to design multiplex PCRs and these were subsequently tested with 20 tissue and 40 scat samples. Multiplex PCR reaction (10 µl total volume) includes 5 µl of PCR master mix (Qiagen Multiplex PCR kit), 1 µl Q-solution, 30–35 ng of extracted DNA, and 0.4 µM of each forward and reverse primer. The thermal profile of the amplification was as follows: initial denaturation at 94 °C for 15 min, followed by 40 cycles of denaturation at 94 °C for 40 s, annealing at 57 °C for 1 min, extension at 72 °C for 1 min, with one cycle of final extension for 30 min at 72 °C. The amplified PCR products were diluted (1:20, except scat DNA PCR products) with distilled water, and subjected to fragment analysis with an Applied Biosystems 3130 Genetic Analyzer. The alleles were scored with Gene Mapper 3.7 (Applied Biosystems). The genotyping output was compared for multiplex and singleplex approaches for efficiency, data quality and efficiency, and cost effectiveness.

## Supplementary Information

Supplementary Information.
