# Supplementary material for: Whole genome survey of big cats (Genus: Panthera) identifies novel microsatellites of utility in conservation genetic study
Source: Sci Rep. 2021 Jul 8;11:14164. doi: 10.1038/s41598-021-92781-0 (PMC8266911; doi:10.1038/s41598-021-92781-0)
Supplement: Supplementary file 1 — Supplementary Information. [file 41598_2021_92781_MOESM1_ESM.docx]

Supplementary information for

**Whole genome survey of big cats (Genus: *Panthera*) identifies novel microsatellites of utility in conservation genetic study**

Jee Yun Hyun^1,2†^, Puneet Pandey ^1,2,3†*^, Kyung Seok Kim ^4^, Alvin Chon^5^, Daecheol Jeong^1,2^, Jong Bhak^5^, Mihyeon Yu^6^, Hye Kyung Song^7^, Randeep Singh^3^, Mi-Sook Min^1,2^, Surendra Prakash Goyal^8^, Damdingiin Bayarkhagva^9^, Taisia Marchenkova^10^, Anna Vitkalova^10^, Hang Lee^1,2*^

^1^ Conservation Genome Resource Bank for Korean Wildlife (CGRB), Research Institute for Veterinary Science and College of Veterinary Medicine, Seoul National University, Seoul – 08826, Republic of Korea

^2^ Tiger and Leopard Conservation Fund in Korea, Seoul – 08826, Republic of Korea

^3^ Amity Institute of Forestry and Wildlife, Amity University, Noida – 201313, India

^4^ Department of Natural Resources Ecology and Management, Iowa State University, Ames IA– 50011, USA

^5^ Department of Biomedical Engineering, UNIST, Ulsan – 44919, Republic of Korea

^6^ Seoul Grand Park Zoo, Gwacheon – 13829, Republic of Korea

^7^ Everland Zoological Garden, Yongin – 17023, Republic of Korea

^8^ Wildlife Institute of India, Dehradun – 248001, India

^9^ Department of the Biology, National University of Mongolia, Ulaanbaatar – 210646, Mongolia

^10^ Land of the Leopard National Park, Barabash, Primorskiy-Kray – 692723, Russia

**Table S1:** Details of the samples collected for genetic analysis. All the samples were legally and ethically collected from zoos, national parks, wildlife institutions with permit from relevant authorities.

| **Species** | Total | **Geographical location** | | | | | | | |
| --- | --- | --- | --- | --- | --- | --- | --- | --- | --- |
|  |  | India | | Republic of Korea* | | Russia | | Mongolia | |
|  |  | Blood/Tissue | Scat/Hair | Blood/Tissue | Scat/Hair | Blood/Tissue | Scat/Hair | Blood/Tissue | Scat/Hair |
| **Tiger** | 67 | 21 | 6 | 29 | - | - | 11 | - | - |
| **Leopard** | 59 | 11 | 15 | 16 | - | 3 | 14 | - | - |
| **Lion** | 18 | - | - | 18 | - | - |  | - | - |
| **Snow leopard** | 8 |  |  | 2 |  |  |  | 6 |  |

*from zoos

**Table S2:**Frequency (%) of null alleles across 32 novel microsatellite markers

| **Locus** | **Tiger** | | | **Leopard** | | | **Snow leopard** | **Lion** |
| --- | --- | --- | --- | --- | --- | --- | --- | --- |
|  | **India** | **Russia** | **Zoo** | **India** | **Russia** | **Zoo** |  |  |
| Pan10C2 | - | 28.5 | - ***** | - | 35.9* | - | 42.2***** | - |
| Pan14C2 | 31.5 | 20 | 8.7 | 40.3* | 17.0 | 15.1 | 35.1 | - |
| Pan15C2 | 7.0 | 0 | 0 | 29.9 | - | 7.3 | 23.6 | 7.4 |
| Pan16C2 | 16.1 | - | 12.4 | 25.2 | 22.8 | 42.8 | 17.8 | 3.1 |
| Pan1A1 | 17.9 | - | 65.4 | 23.9 | 92.7 | - | 14.6 | - |
| Pan1A2 | - | - | 0.0***** | 31.8 | 30.9 | 14.4***** | 31.2 | - |
| Pan1C1 | 38.1* | 25.1 | - ***** | 34.1 | 18.0 | 79.0***** | 41.3 | 0 |
| Pan1C2 | 30* | 17.2 | 10.5 | 19.8 | 38.2 | 36.3***** | 31.2 | 19.2 |
| Pan1D1 | - | - | - | - | - | - | 87.1 | - |
| Pan1D2 | - | - | 94.4 | - | - | 26.3***** | 47 | 22.9 |
| Pan2A1 | - | 39.1 | 16.9***** | 28.7* | 65.6 | 7.3 | 36.8 | 6 |
| Pan2C1 | - | - | 17.6***** | 23.2 | 92.2 | 47.9 | - | 24.5 |
| Pan2D1 | - | - | - | - | 89.1* | - | - | - |
| Pan2D2 | 20.5 | 43.3 | 25.8***** | 15.9* | 16.0 | 0 | 31.2 | 20 |
| Pan3A1 | 7.4 | - | 48.7***** | - | 15.6 | 23.6 | 28.6 | - |
| Pan3A2 | 19.3 | - | 25.2***** | 15.0 | - | 2.4 | - | 17.6 |
| Pan3C2 | 22.2* | - | 22.1***** | 7.1 | 16.5 | 33.3 | 18.3 | - |
| Pan3D1 | 10.5 | 68.5 | 7.4***** | 41.3 | 33.0 | 22.4 | 31.2 | - |
| Pan3D2 | 7.8 | 64.9 | 16.0***** | 0 | 29.9 | 26.8 | - | - |
| Pan4A1 | 14.6 | 20.3 | 12.2***** | 18.3 | 16.4 | 10.9 | 15.9 | 0 |
| Pan4A2 | - | - | 0 | 25.7 | - | 0 | 0 | - |
| Pan4D1 | 20.8 | - | 21.8 | 35.2* | 29.8 | 0 | - | 0 |
| Pan5A1 | - | 20.4 | 17.6***** | 17.0 | 17.0 | 12.8 | 20.7 | 44.6 |
| Pan5D1 | 23.4 | - | 16.1***** | 24.9* | 15.8 | 0.0***** | 33 | 20.6 |
| Pan6A1 | 8.6 | 0 | 27.7***** | 19.3 | 0 | 25.9***** | 44.3 | 0 |
| Pan6A2 | 24.6 | - | 9.6 | 29.3* | 15.4 | - | 0 | 20.2 |
| Pan6C2 | 6.9 | - | 26.0***** | 41.7* | - | 38.7***** | 22.4 | 0 |
| Pan7A1 | 9.2 | 63.7 | 3.9 | 1.9 | 0 | 27.5 | 29 | - |
| Pan7C2 | 5.8 | 0 | 19.2***** | 28.2 | 3.4 | 10 | 0 | - |
| Pan8A1 | - | - | 19.5***** | - | - | - | - | - |
| Pan8C2 | 13.9 | - | 16.7***** | 41.0* | - | 19.5 | 32.8 | 0 |
| Pan9C2 | 14.4 | - | 26.0***** | 24.7* | 93.7* | - | 72.3 | 24.1 |
|  |  |  |  |  |  |  |  |  |

**‘ - ’ – Insufficient alleles to calculate null allele frequency,* HWE deviation**

**Table S3:** Unique Target Variants (expanded to +150bp around the variant site) sequences used in the present study for primer designing.

>A1:371081-371381|varpos=371231

GTATTGTGTGTCACACATAGTAAGCACTCAATAATTGGTAGCTCCTATTATCAATGTCATTCTCATGATGATCATTATCACNGCTTTCCATTAGCTTAGAGATGACTTCTCTGAAGTAAAATGCTTCCAAGCTGGCATGATCNCTTATG[TACACACACACACACACACACA]CACACACACACATTTCCAGAGGCAGGATTTTTCCAATTCTGTTCTCTCAAGAAGCTGTTAAAAAGGCAGTGTTGGGGGCGCCTGGGTGGCTCAGTCGGTTAAGCGGCCGACTTCGGCTCAGGTCATGATCTCGCGGTCTGTGAGTTCCA

>A1:686568-686868|varpos=686718

CTCGTGGTCCGTGAGTTTGAGCCCCACATCGGGCTNTGTGCTGACANCTCAGAGCCTGGAACCTGCTTCAGATTCTGTGTCTCCCTCTCTCTCTNNNNGGCCCTCCCCTGCTCATGCTGTGTCTCACTCTCTCTCTCAAAAATAAATAAACATTAAAAA[AAATTTTTTTT]TTTTTTAATATATTTTNAGGTATATTTGATATTTTTGATGCAATAATAGTCTTTTTAAAAATTCCATTGTGTTTGTTGCTCATATACAGAAANACATTTAATTTATGCATATGTGCCCTCAGTTGGTTAAAGAAATTCCCTTCTATCTTT

>A1:694971-695271|varpos=695121

TNGAAGTTATACCGTGAATCTTTTTGACCACAACACTATGAAACTGGAANTCAGCCAGAAGAAAAAATCTAGAAAGACTACAAATACATGGAGGTTAAGTAACATACTACTAAACAATGAATAGGTGAACCAGGAAATTAAAAAAAAAA[AAATT]TAAAAAGTACATGGAAACAAATGAAAATTGAAANATAACAGTCCAAAACCTCTGGGATGATGCAAAAGTAGTCCTAAGAGGGAAGTTTATNGCAATATGGGCCTACCTCAAGAAGCAANNNNNNTCTCAAATAAACAATTTAACCTTACG

>A1:2136651-2136951|varpos=2136801

AGTTTACATTTAAGTGACAAAAATAGTATTTTTATTTCTCTGACTNATTTCACNNNAAGTCCTCCAGACCCATCCATGTGGTCACAAATGGCAGGATTTCCTTCTTTTTATGGCNGAGTAATATTCCATTNTGTACACACACACACACA[CACACACACACACACAGA]GTTTCTTTNTCCATTTGNCCATTGATGGATACTTAGGTTGTTTTCATTTCTTGGCTATTCTAAATAAGCTGCAACGCACACAGGGTTACACATATGTTTTCAAGTTAGTATTTTCATTTCCTTTGGATGAATATCCAGAAGTAGAATTG

>A1:2544745-2545045|varpos=2544895

TTGAATAGGAGAACAGATACAAAAGAGTGGATGTTTAATTATCGGACACTATTATGATTATTTATCAAAGGCCANTTGTTTGTTTTCCAGGAAAGAAAAACAAACCCATGTTCTATCTGTTTGAAANAGTTTTTACCTAGTCNNNNNNN[CTTTTTTCTTTTTTTTTTT]TAACTTTTCTTCTCCCTTTGATTTCCTAAGAATACAAGATGATAGACAANTAAAGTTGTTTTAGTCGTGTTACATTATCTAGTCTGAGGAGAAAAAATCANTTGGAACTTTACATAAAATTAAATTTAAAGGTCTTCACCATT

>A1:10924305-10924605|varpos=10924455

ACTGATGTGTTCAGTGTTGAAAAATTTAAGGATTTAGTTAAGCAAATAGAAGAAAATAAAANNNGCCATCCATAATCCCACCTCTTCAGTGTGATCACTGTTTGCATTTTTCATATTCCCTTCTGTATTTCTTTTTTTCACACATATTGTTTA[CAAAAAAAAAAAAAA]TGGAATCATTTAATATACTGTTTNGTAAGTTACTTTTGTNAGCTAACACAATTTACCCCAAATATCTTTCCGTGGTAGTAAATCATTACATAGATTTTCAGACAGACTGAAAGNTGTCAGACCTTGGTCCTGTGTTTTGAGAC

>A1:12159930-12160230|varpos=12160080

GCTGTCTACCAGCTAAGTACTCTGTGGCTCCATGGTGATTATCAAGCCAGTCAGAATCTCAACTGGGGCCTGATTACTAAATTTAATAGGATTATGCTTTTTTTCCTCTCAGAACTGAATCAAAAAAGTTCTTTTCAGCTGAGGTTTTT[GTGTTGTTGTTGTTGTTGTTGTTGTTGTT]NNNNNNNNCTATTAGTAAGTCAGTCCAGAATTATCCCAGAGACCTCAAAGATTGTTAGAGCTNCTTTTCACTTATTTGGGGGAAATGAAAGACAGAATTCTTGCAGGGNTGAAGCAAGANCATAGAAACAGACTCCTCCT

>A1:12644926-12645226|varpos=12645076

AGAGCCCAGAGCCTACTTAGGATTCTGTGTCTCCCTCTCTCTCTCTGTGCCTCCTCTGCTTGTGCTCCTCTTTCTCCCTCTNTCTCNNNNNNNNNNNNNNNNNNNNNNNNNNNNNNAAAAATAAATAAACATTTTTAAAAAATTAAAAA[AAAAACCAA]NNNTCCTTCTGATGTTACTTGGCTTGGGTGGACCATGGAACATTCTGAATTCTCTCTCCTCTGTGTCTATCTAAGCTTTCAAGTCATAACCCTGACTGTAAACATATGTCCAGTGCTTAACAATAGGAANAAGTTCTTCATATAGNGAGC

>A1:12688855-12689155|varpos=12689005

TACAGATGTANAGAAAATTGTTTTGGAAGNTGATGATGNGTTTTGCAATTCAGTGTTATACTTGGGGTAACCATTGAATTGTCCNTATTTAGCTACCCTCCAGGAAATCTAGAAAATTGCAGGAANTCAATAAAATGTTGAATAATATT[TTAATAATAATAATAATAATAATAATAATA]NTGAAAATATTGAACTTCTATCAACTATTCCACATAAAAAGTTCATTGAGTAATGACTATTGTGTGGGTCGCTTTGCTANGCAAGAATATTTCAGAGGTTTCACTATGTGCCATGCNCTGTGGTGGATGTTCTCTA

>A1:13036003-13036303|varpos=13036153

AAATATTAACATTTAAAAAATTAATAANTTTGTAATCCTTAAANNNNNNNNCTAAGACCACTAGTAACTTATTTTTCTTATAACTATTTAGATAATTGTTTTAATAAAACACATTGAATTTTCAAAAATTTAAAAATAAAGTTTTATTT[TAGAGAGAGAGAGAGAGAGAGAGAGAG]NNNNNNTTGGGGAGAGGGGCAGAGGGAGACAGAGACAGAGACAGAGATAAGAGAATCTTAACCAAGCTCTACACTCAGCATGGAGCCCTACACNGGGCTTGATCCCATGACNCTAGAATCATGANCTGAGCTGAATTCAAGA

>A1:13061768-13062068|varpos=13061918

AGAATCTCANGCAGGCTCTCAACCCAGAGCCCAACACAGGGCCCAGGTTATGAGATCATAACCTGAGCCGATATCAAGAGCCAGANGCTCAAGCACTCAGCCACCCAGGTACACCTATGGTGCTTTTATTANTATTATTATTATTATTN[TTATTATTATTATTATTATCACT]TTTATTATTTTTGTGCAAACATATCAGAATTCTCTTAAAATGGGACCGTTTATACACCTGTGCATGTTTCTTTATGTGCCTCCTACCGGGCATTTGAGAATGATCCTAGTAAGAACTTTTCCCTGGACTATACTCTGTTCCAGGCACTT

>A1:13333085-13333385|varpos=13333235

TTGACTTTGCCAAACCTCGTTTAAAATGAGTCANGTAAGGGTTGACGGGGTTCAGTCTTTGCTGGTGCCATAACCAGGATGGGAAGGAGATTCCAGGATTTCTGGATAGGCTCTGGAGGGTTACTCAGGGCAACAGAAGATNGTAGAGC[AAAAACAAAACAAAACAAAACAAAACAAAACAAAACAAAACAA]CAAAACTAAAAAACACACTTCTCAAAAGACAGTGTCAGATAACAATGTTGACAACCCCAAGTTTCNTGCAAAGGAAGNTAAGAGATCAGAAGCTATAAGGTTGGAGACTGCCAAGGCAGCTTCGGATG

>A1:13637550-13637850|varpos=13637700

ACCGTGATTAGCATAGTNTTGACTTATCACTTCTTTTTCTCTGCTGTGTGCATTCTTGGTCAAGGTTAATATATCTGCAACCACAATGCTGCAAATGGGCAGATAGCTATTTCCTATGAAATCTANTTATAGGGAGTCANAGTGTATNN[CCACACACACACACACACACACACACACACA]TGCACACACATACACACACCCATGAACTGGCAGAAGATTATAATAGCACCCTTTTTGAGATNGCTAAGAGGATCACTTATAGTGGTGGCAGGTAACTCTTAATTATTACTGCTAAAAAAT

>A1:14810646-14810946|varpos=14810796

AAAATAAAAAAAGAAAGAATCTACTACCCTATTCAGGAATTCAGTAAGACAAGACGAGGTTTCCTTATGCTAAAANGCTTCTATAAAATTGTGCAAATATACTATTCGACCCCTCATTAAAAAAANTTTTTTTAACNTTTTATTTTTGA[CAGAGAGAGA]NNNNNNNNNNNNNCACNNNNNAGGGGCAGACAGAGAAACAGAAATAGACTCTGTGCTGATGGCAGAGAGCCNGATGTGGGGCTGAGACTCACAAATGCTAAGACTGTGACCTGAGCAGACGTCGAATGCTTAACCNACTGAGAAGTCCA

>A1:15186178-15186478|varpos=15186328

GTAAATTTGTGGCAGAGAGAAGAACATGTTTTGCTTTCCAATCCTTTCTGGTNNGTGCCAAAGACTGCTTCCCATGAGGAGAGACAATATCTACTCTCACATTTGGAAATAAGAAGATTGCATTTNNNNNNNNNNNTAATAGTACTTTC[AGCCCAAGCACACGACGCGCGCGC]NGCGCGCGCNCNCNCNCACACACACACACACACACACACACACACACACACACTCCTACTTGCCNACTCTGGAGACTCGTAACTCATCTGGATTATTTGTTCTTGGAAGTAACTAAGCACAGAAAGGNAGAAGACATATCACAGAGACATG

>A1:16681925-16682225|varpos=16682075

AATTTTAGAATCTTCCTNTCTAGTAAGAGAAAGAATCTCTTCTCTAGGTTCTGAAATTTCTTTCCACTTTGTACAATTTTTTCAAAAATGTAAATGAATAAAATTCGTATTCTCATAAACTTCTTGCACATGTTTTAAGATTGTATTTT[CTTTTTTTTTTTTTTT]AACTTGGAATTGGTTCTTATTTTGTACTGAATGTTTAATTCAGTAATTTGATCATCACTGGTGTCATTTCTACTTTTGCTTCCTGAAATATTAATTTCAATTCTGCTACTGAATTCATAGTATCCTTACTATCCTAACTTTC

>A1:16785893-16786193|varpos=16786043

CAAAGCCTATCTTTANATAATGATCAATGAAAACTCACTATTNNAATGTTGGGAAATATTAATTAAATGCAAAGACTAATGTCTTTTGTGTTAAACCATTCTAACAATTATTGACCAAAAAGNCTCTTGCATTTGCTTTTACTTAGGAA[AAACAACAACAACAACAACAACAACAACAACAACAACAACAACA]NTANAAAAAATGCATCAGTGCTTTTGATTTCTACTGTAATCAAATTGCTACCATGAAATATTATGCACTTTATAATAAGAAGTTATATTTGTATATTTATTTGAAATG

>A1:17122551-17122851|varpos=17122701

CTGAGTGAGAATATCNGCTAAGATAGGAATCTAGGAAAATGTTGGCAATAAAAAATAAAAGCTTGCTAATCCTGTGTTTGTCTGCAAAACAATTACTTGAAATTTTACTGGCCCATGAAATTCAAAAAGCATTAAAGAGTAGATCCCTA[TACACACACACACACACACACACACACACACAC]GTACACACACATACCANATACAATACCTTTGCCACAGCCACAATCTGCCCCCACCANGCTCCCCCCTGATATTTGGATGCTGGGTATTAATTACTCCTTATTTTTAAAAAAATTTTTT

>A1:17849177-17849477|varpos=17849327

ACTAATAGGAGAGGTGCAGGGATCTCTAGTTAAGGAAGCACCCAAAGACTATGGTGGTGACGTTCCCTACCTTCTAGTNGCTGAGGCTAGCCACCCAGAACTTCACAGTTAAGAGATGAAATTCACATGCTATTAGTAAATTAAAAAAA[ATTTTTTTTTTTT]TTTTTTTAACACTTANTCATTTTTTGAGAGACAGAGAGAGAGNNNNTGCAAGTGGGGGAGGGGCAGAGAGAGGGAGNCACAGAATCCGAAGNAGGCTCCAGGCTCTGAGTTGTCAGCACAGAGCCTGATGTGNGGCTCGAACTCACGGACCACAAGATCA

>A1:18592916-18593216|varpos=18593066

ACNGAGGGTTACTGGAGGGNTTGTGGGANGGNGGNTGNGCNAAATGGGTAAGGGGCACTAAGGAATCTACTCCNNAAATCATTGTTGCACTATATGNTAACTAATTTGGATGTAAATTTTAAAAAATAATAAAATTAAGAAGATAAATA[AATAAATAAATAAATACATACATAAATA]GCATTAAATAAATGAATAGGGACACCTGGGTGGCTCAGTCGGTTAAGCATCCAACTTCTCTCAGGTCATGATCTCAATGGTTCCTGAGTTCGAGCCCCATGTCGGGCTCTGTGACAGCTCAGAGCCTGGAGCCTGCTTTGGAT

>A1:19007884-19008184|varpos=19008034

CATGTCNAAAGCAAGTTAATCCANAGTTGGAAGANGAGGTTGAGAAATCCCCAGAAAACAGAAGAGACCACAAAAGAAAAACCAGTTGATATAAAGGATAAAAACAGATCTCTAACACCTGGACTCTAGATAACAAAATATATATATNN[ATATATTTTTTTTTT]AAATAAATTTCCNAAAATTAAAGAAAATATCACAAAGTGATGTTTCATCTAACAAACATTTAAATGGTATTTACTAAGTGCCAGGTATTGTCCTCATGCTTCACAAATATTTCANTTGGTCCTCAACCTTCAAGATAGTTACAATT

>A1:19045251-19045551|varpos=19045401

GACTAACGAGAGTCAGTTGGCTCTAATTATCATAGTCATTGACCCAGGAAATGAANGAGGGAGAAGCCCCCACCTANGGTTAGAGTTATGGGAATGTAAGAGGAGAAATAGTGAGAGAGAGTGTCNGTCTCCTTTGTTAGGGATGGCCT[GTGTTTTGTTTTGTTTTGTTTTGTTTTGTTTTGTTTTGTTTT]NNNNNTTTAGCAACCTGGGTTAGGTGTTGGAACACTTCTATATATTGATAAGGGTTGTCTGAAAATTTGCCCAGGTGCTTTTTATTTGCTTAGAATCTTGCAAGGGAANTGGAACCTTTNNGGGGCCAAATCCA

>A1:19063943-19064243|varpos=19064093

TCATCTTAGAGGAAAAACTTTCAGCTTTTCACTGNNNNTATGACGTTTGCTGTGGGTTTGTCATATACGACCTCTATTATGCTGAGGTATGTTCCCTCTATATTCACCTTGTTGAGAGTTTTTATTANAATGTTAAATTTTGTNNNNNN[AAATTTT]TTGCATANATTGAGATGATCATATGCTTTTTATCCTTCATTTTGTTAATGTAGTNTATCACATTNNTTGATTTGCAAATNGTGAACCATCTCTGCATTNCTAGAGTAAATCTCACTTGACCATGATGTATGATTCTTTTAATGTATNGGG

>A1:21582272-21582572|varpos=21582422

CAGAGTTTCAGTTCNGGAAGACTCAGTTATGGAGGGAGATAAATGGTGATGGTTGCACAAGAATGTGAATGTACTCAACACCACTGAATTGTATACTTAAAATGGTTAAAATGGTAAATTTTATGTCTATTTAACTACAGTAAAAAAAN[AATTTTTTTTTTTTT]TTTTTTTTGTTTTATTTACATTCTTTTTAGGGGAAANAAAGCCAGGCCTGGGTACTCAAGACAGAGGAGCTGTGCATGCTCAGGCCTGGAGGAGTAAAAACCAGCCTGAGGCCTTCTGAGAACACAAGTATAAGTGCCCTCAGCCCTGGC

>A1:21874424-21874724|varpos=21874574

ATCTCGCNGTCTGTGGGTTTGAGCCCCGCCTCAGGCTCTGTGCTGACAGCTCAGAGCCTGGAGCCTGCTTCCGATTNTGTGTCTCCCTCTCTCTCTGNCCCTCCCCCATTCATGCTCTGTCTCTCTCTGTCTCAAAAATAAATAAACAT[TAAAAAAAAAAAAAA]AAAAAAAAAAAGATTAGCTTGTAAATAAATGATCNCNTGAGAATGCTGACANGACCTGGAGATCATNTTGGCTCATGGAGAAGAGAAGTGGTGTGGGTGATCTAANACAGTATCTTCNATGGTGGGGTCACTGTGTGAAGGCATGCATGC

>A1:24750152-24750452|varpos=24750302

GACTTAGCTAGCAATTGTATATAGCCCGTTCTGCTGGANATTTGGTTAAATATGGTTTTATTGATGGATAGTAATATGTCTTAATCAAGTTGTAGGATGCAGAATTTTGTGTTAAATGAGAAGAATGTAGCTAATCACTTAAAAAAANN[ACAAACAAACACACA]ACACTTCATTATANTTACCTGAATATAGAAATCTCCTTCTTGATAGTACAAATGCTTGCATGATGAGTCTTACCCAGATTTCAAAGACAGATCTCATCATTCAAAACTTGTTGGCTTTGACTTCAATTTAGTAGAATAAGCAAATTATCA

>A1:25775528-25775828|varpos=25775678

CCTGTTGCTTTTAGTGCCTCCAGCTGATGGCCTCTCCTAAATCTCAGTAGTTCATTCCATTTCTTCTGTTCCATCAGTCACTAAAACATTAACATTTGCTTGATCTCCAAAATGGTCTTTGGGACGTAGTCCTTCCTTTCATACTAATC[ACCCTCTCCCCCCC]NNNCCCTTGCTTTGTGATGCTTTTGCTANATTTTTAAGGTGCTCTTGGCCAAGATATCAAAGTTGATGTTACAATAAACAATGTCACATGCTTTTGAAGAACATGCTTTGCTGNNCCTACATAAATAATTTCTGTTGGGTATTTCAGCAT

>A1:28483914-28484214|varpos=28484064

GNAAATGAGTGAATAAATTGAGGATTATAAGAGCTATGTTTCTTACCATTGGGAAAGGAATTTATAAGCATAGAAAGGGAGAAGAAAAGAAAGACCCCTGAGGTGTTAGATTGGAATTGGAGGNATCAGTATGAACTCATGTTTTTTNT[TTAAA]AATATTTTAATATATATAAGCAGAGTAATAGATATAGATACTGTGNATGTATGAATATACAGACACATATTTTCTAGCTCTGTCCCCTTAAGAGGGCCTGAAAACTATGACATTCCAGTGGCAATGATCATACCAAGTGCCAAGACATTG

>A1:29158368-29158668|varpos=29158518

GTTTTTCAGACAAACAAAAACTAACAAAGTTCATGGCCNCCAANCCAACCCCTTAAGAAGTATTAAAGGAGCCNNTCTGGGAATTAAAGACCATAAATCATAGTATGAAAAGTAAAACGCACNAAAGCAGTAAAATATTTCTGTAAAAA[AAAAAAACCAAAAA]NNNATCAAAGGAATCATAAAATAAAAAGATGTAAAATATAACATCATATACTTAAATGGGGTAGAGGAGTAAAGAAGGGGTTCAAATTTAAGTGACCNTCAAGTTAATACAGAATATATGCAGAAGATATAAACCTAATGATAACCNCA

>A1:29352647-29352947|varpos=29352797

AGGATTGTTCAGATTATCTATTTCATATTGAGTGTCTTGTGGCAGTTTGTGTTTTTGAGCNTTTATTNTGTTTTATCTAAGCTGTAGTGATATGTGCGGAGNCTTTAGTGATACTCCATTTCATTCCTGTGATGGGAATTTGTNNNNNN[TTCCCCCCCC]CCCCCCAGTCTTGATGTAGAGGTTTGTCAATGTTAGTGAATTTTTTTCAAAGAATCAACTCTTTGTTTACTTCATTTTTTTTCAACTTTTTATTATAAATTTCGTTCATTTCTGCTATTTTCTCCTATCTGCTTGCTTTGGGTTTATTTT

>A1:29928412-29928712|varpos=29928562

TTTATTGGTCCTGGGCATATANGTTCTATCTTCCTGGAGNACTCTTACCCTCCAGCTTCTCTTGCTATCTCCCTCCTATCATTTAGATCCCNGATGATACATCACTCTTACAGAGACATCTTCCTTGACTTTTTTTATTATTNNNNNNN[AATTTTTTTT]TTTTTTATGTCTATTCATTTTTGAAAGANNNNNNNNNNNCACAAGCAGGGGTGGGNTGGAGAGAGAGGGGGACACAGAATCTGAAGCAGGCTCCAGGCTCTGAGCTGTCAGCACAGAGCCCAACATGGGGCTCAAGCTCATGAACCGTGA

>A1:32797105-32797405|varpos=32797255

ATAATAGATAAGAAATGGGAGTGTTTCTTCTGTTGTCTAGTCTGCATTTCAATGTTTTCAAGGTTTCACATCCTCCAGAATTTCCAAAATGCATGTTTGATTTCAAAATACTCTCTAACATTAGACAGTAGTGTTTGAAACATAGCCTT[ATTTTTTTTTTTT]NNNCTTTTTTACNATACTGGAGCATGTTGCATATTGTTTAAATTNAAGTGACATGGCAAAGATTAATTATTGGGGAATTATCTCCTAACAGGGATCTTGTACAGATTGCTTTTGTTCTTTCATAGCCTATTTTTATTCTGAGAGTA

>A1:32984456-32984756|varpos=32984606

TATTGAGCAACTTTATCAAAATACCTAATCGCTTGTNGNTATTGGNTACATTTCTATAAATTAGAGACTTCTACCATACATATTGAGCATTGNACTGCGATAAATGTTCTAAATGCNGGGNTTGTAACAATAAGCAAAACAGNNNNNNN[AACCCC]CCCACAAAATATAAATAAAGATACNGTAATATAACACCAAGTAGTGCTAGATGTTACAAAGAAAAAAANNNNNNGTAGGGTTGTGTGTATAGGATAGATATTAAAATCAGATGATTAAAGAAGTCCATGAAAACATGAAATGTGTATATGAATTCAA

>A1:33995794-33996094|varpos=33995944

AAATGCCTTATCTAAAACATTAATCCATCTGTTCAAATTAAGCCAATATATGTCTGAAGAAAATAAACTACAAGTAAATAAAACTGAATGATGAAATAATGCCTATATTCTCTCTGGGACAAATGTATCCTGGATATAAAACCATCACT[GTTTTTTTTTTTTT]CTTTCTTTNTTTNTTTGCTTATTCTGAAAGTCTGTTTTTATGGTTTTCCACATTTAAAATAGTTATCTTNAGATAAACAAGATAGTCTTGTGAAAAGATTGGCCTGAATTATTTTAATTTTATATTATCGTATAAAGTGGTCCA

>A1:34520838-34521138|varpos=34520988

GGGAAAAGAGTTAATAGTTATCAAATTATAAATGTAATACTGGCACAAAATGACTTTAGTGACTTTCTATGANTATTCTAAAAGTCTCTCCGGATTCAGGTAGCTACATTTTCTTCATTTTCTATAGAAAGATTTCCTTTTNAAAAAAA[AAAAAAATTTT]TTAACGTTTATTTATTTTTGAGAGGGAGAGAGACAGAGCTTGAATGGGGGAGGGTCAGAGAGAGAGGGAGACACAGAATCTGAAACAGGCTCTGGGCTCTGAGCTGTCAGCACAGAGCCTGACGTGGGGCTCNAACTCANGGACCANGAG

>A1:34753079-34753379|varpos=34753229

AATAGATACAGGAAAATAGCATGTAANTAGAAAGGAATTAAATTATTNNNGTCTTTGCATTCTTTCAGAGAAATGTAAAGATACTGATTATGTTTAGTGCCTGATAGGTCTGCATGCTTTTTAAAGCTTTAAAGATTCACAATAAAAAA[TAAAAAAAAAAAAA]NNNTACGAAAGAAGTTTATAGATAAGGGTCTAATCACAATAAACATGAATGTATTAATAAAATATAAAAACGGTCCCATTACATAAAATCCAGCTATGTACCATTTATAAGAGACATACGGGAACAGTAAAGATATAGCAAGANTA

>A1:35545304-35545604|varpos=35545454

GTAATATAAAANGAGGAANGGTACTTCAGTACTCAAGCTGTTAACCCATGTTTCTTTNAAAATTATTTGTGAACATCTCAACANCTGTGTAAAAACTAGGTAAACTGCTGATGTGGGTTTCTCTCTGCCCTAGCAAATGAATCCCANNN[GGGAAAAAA]AAAAANAAATAGGTGTAATAACCCTATAAAGAANTCTGGACTACCACTAATTCTACCTTGTCTCTTACCATTGTCTTAGGAANCTTTGTACCCATCTGACAAAATAACGGTAATGACAGTGGGAAAGATGGCTGTACCATAAACAAAACC

>A1:36007241-36007541|varpos=36007391

GCTCAGTCAGTTAAGCNTCCGACTTCGAATCAGGTCATGATCTCGTGGTTTGTGGGTTCAAGTCCCGCATCCGGCTCTGTACTGACAGCTTGGAGCCTGGAAGCTGCTTCAAATTCTGTGTCTCCCTCTCTCTGCCTTACCTGCCTTGT[GCTCTCTCTCTCTCTCTCT]GTCTCTCTAATATAAATACATAAACATTAAAAATGGATACGANATCTATGAAGGATATAATNNGGAGAAAGGGTATAAAAACAGTCTTTAAAACACCTCCTNTCCAAATCTTCCAGCGATTCCCTCAAGCTACATCCAACTA

>A1:36416994-36417294|varpos=36417144

ACTGCTGAAAATCAACTGTAAATTGGCAGTTTTATTTCATGCAACATTCAGTCTTTTGGCAGTAACTANCTCTGTGTAATCTCTAGACTNTCCTCATCATAAGGAACTTTCCTGGCAAAAATATTATGAATAATACACATGAAATCTTT[GAAAAAAAAAAAA]AAAAAAANNNTTGGTAATGTCTGTAATTCTTTCTATTCTAGACTTCAAACTTCTTAGAGGAATATCAAATATGGTTGCNNCAACAAGTGTATTCTTAGTGGCATCCTTATTTCATTTTCAAGCAAAATGGAAGGAAACCAAAACACTGGC

>A1:37562935-37563235|varpos=37563085

ATAAGAAAAANNNNCCTACACCATGTGGAATTGACTTTGGGGCTGAGCTANTGACAGGTGCTAGAAAAGCAGTAGAAAAGCTGTTAGAAAAACAGCGAGTGATCAATTAGTGGAGTCTATAAAAGTGGTGAGAAAATTGCTATAGCAGATGAAN[AAAAAACAAACAAA]NCAGTTGACCCTGATATTTTTAATTGATTTTGGCAAATAATCAACAATTTGTGTTCAGTACATTTGAACACAAACTGTCTAAGTCTACAAAATGGTGTATTAGGAAGCTGCAAACTCTTATGTAGTAACACTGAAAAGTAACATTGAA

>A1:39127838-39128138|varpos=39127988

AGCATATACATGCCTATGCCAAGAATATCTCTGAAAGGCTAACCATGTAACTAAGGAGATATTGAAGAAATAGTTGCCTCTGTATAGGACAACTGGGAAACAACAGACAATGACAGGGAGAAAATAAAATTTCACAATGTAATNNNNNN[AAATTTTTTTTTT]TTTTTTACATTTTATTTATTTTCNATAAAGACAGAGCACAAGTNGGGTAGGGGCAGAGAGAGAGGGAGACACNGAATCTGAAGCAGGCTCCAGGANCTNAGCTNTCANCACAGAGCCTGATACAGGGCTCAANNTCACAAACTGCGAGAT

>A1:39528928-39529228|varpos=39529078

TAAATAGTTCTTGACTTCATTGTACAATATATAGTGTGTTAAGTAAAGCAGTTTATTCAGTGTCTGTGGTGAGTTTAGACATCTTTTTGAATGCTGGAACATACATTAGATTTCCTTATATCCTCCAAAAATCTGGGAATCTGTAACNT[CTGTGTGTGTGTGTGTGTGTGTGT]NNNNNNNNNNTTGCAGAAAGCTATNTTTTTTAAATAAAAAGTCAAATTTAACCAAATTTTAGCATGTCCTAGAAAAATGNATTTTGCTGANAAGTTCTTAGTAAACATGTAATATTTTTANTGTCCATAAAATTCTATGATGG

>A1:41400656-41400956|varpos=41400806

AAACCTAGTTTGGAAAAGAGGTTGAAATTATAGCAAATCTTGATCACCAGACTAAGCGGTTTTGATNTTATATGGTAGNCAATGAATGTGGAGGCATTTAAATATCAAGCAAAGGAAGGACAATATTAGAGCAATAATGTAAAAAAAAA[AAAATT]TATTTGGCAGGAGTTTAAAAACATGTTGGAGGGAGAAAATGACCATTGGCAAAACAAGTCCCTAAGCACCTAATTTTAATTATCTGGGTATGAGGTAATTTGGTATGTAAACATGGAAGCATCAGGATATGCTGAAGAAATAGAATCTGT

>A1:41446662-41446962|varpos=41446812

CCTTCGAAATTTAGTCTACGGCAAAAATGTTAACAGATGTGAAGCCAGAGGAATGNCTCATTGCTAAATTCTAAGTGTTGTTGCTTCAGTGAATTCCAAGCTGTTTTAATAGATATTGCTACTTTGTTGGGGTGGAAGGGATGAAAAAA[ATTTTTTTT]TTTTGNTAATAAATGACAATAGCATGTGGAATACAACCTTTTGTATTTTTTGTCTGTTGTTTTTCTGGNCACTCTGAAAGCAGTGTGACCTGAGAATTCTGAAACCATCTACTNTTTCAATGGTGTGAGTCAAAGTTGAAAGGAGATAAC

>A1:41452425-41452725|varpos=41452575

GGGAGAAATTATGTAATACGCNATGATGACTATGTGAACCACAAAAATCTCCTGGTGTGACTTGATTTTTGTTTTTNCATTTCTTTTTTCGNNNNNNNNNNNTTNTTTTTTTGTTTTGCACCCACAGTAGGTAAGAAAAATAGACCCCC[CCCCAAAAAA]AAATTTTTTTTAATACAGAACATTTTTGCCAGAATAAATATGTTACAAAGGAGGTTTGGAGGATACAAAACATGGAGCCTGAAGAGTGCAAACTGAAGAGCTCCCTCAGAGGGATCTGAACTCGGTCTATAAATATCTTCAAGGTAACCG

>A1:41577464-41577764|varpos=41577614

ACATCTACTGAAAAATATAATGTACAACTTAAAGTAGTTCTGTGCGTCCATTTTAAAAAGTGCTCCTTTGATTCCAACATGCTCACGTTGGAGATACAAGAGTTATTTCACAGAAATAAAAAGGCACTTTCTCTCTAATGTTTTTANNN[GTTTTTTTTTTTTTT]ATAGATTCTCTAATATAATTAGTGGAATTACTGGGNCATTTATACTAGAAACTAGATAGCAATTTTAAACTTCTTTGTTCCTTTTATCATCCAAACGCTGGTNAAATTCAGAAAAAAATTATCTGCAAACATACTTNTTCATTTTTTC

>A1:41914904-41915204|varpos=41915054

CTGCCCCACGACCATCTGACAGCATGTCTTTGCCTCAGTGTTTTTATTTGTAAAATGGGAAATTTAGAGGTTAGATCTTCTCCACCTCTCCAAGTAAATATGCAGTATACATGTGAGATGTTATTAGATACGTTACCCAAAACTTGGTA[GTTTTTTTTTTTTTT]CCCCAAATACTGATCTCTAAACTTAGATCTTCATAAAATGGTGTAAATAATTTACTTGATAAAATNAACTGCAGGAAATTAGAAACTTTGCCACTGATTCTGTTATAATTCACCACTGATTCATTTGCTCTCATCTTGTTGAT

>A1:42025017-42025317|varpos=42025167

ATGAAAAGGAAGAGAATGGTAGTAACTCTGGCTGCCATACCTATTTTCTATGCCCAGNCACCTCAGAAAAGCAAAACCTGCATGCAACTGTTAGCCCATGTATTAGGCTAAATTCTTTCATTAGAAAACCAACAGAAACTTATTTTTCT[TTTTAA]ATTTTACCTTTAAATTGGTGTATTTTAATGTAAAGGGATAAGAAATTACTGACCATATTAGACCAAGAGATATAGAACTTTACAATCTCATACAAACTTANGTTGACTTTAGCAAAGATTGACCAGTGATTGACTAGTGATTAATTACTA

>A1:43791211-43791511|varpos=43791361

TGCCTAAGAATTTNAANTGGCACAAGACAGATTAACAGGANNNNNNNCATAAAAATTAATTTGATACAAATTTAACCCGACTAAAGNCTTCNTAGAGAATAAAGACCCAAATAAGTGGTAAAACCTAAATGTTTTTACATTACATTGAA[AAACAACAACAACAACAACAACAACAACAACAACAACAA]AAACAAAAAGGTGGAAAAGTAACTAAACTAAGTGGGAAGCTAAAGGAAGATAAGAATTATTTTAACAAGGTCTGTTTGTATAGAATTCTTTTGGCTTCAGCTTCTTGTCTGATGACAGGAA

>A1:45453017-45453317|varpos=45453167

TAAATAATCCTGGCTTTAGGTTTTGAGATCTAGTTTGAGAGATTNGATATTTCATTTTACTTTCTACAGTGAAAGAAATAACATTCTGAAAAAGTAGNNNNNNNNTAGTTAAATGTATATGAAATTCATTGCTAAGTTGACTCNNNNNN[CCCAAAAAAAAA]AAAAAAACAAAAACAAAAAAACAGGAAAGTCAATTCTCAGGNCCCAAGTGAAATATCCAGAACCCCAAAGTCATATAANAAACAAATCAATTTTCATCTTGAAGATTNGCCCTGAGGGTATCTGCTAAATGCTAAAGATCTTGAGATTCT

>A1:45671157-45671457|varpos=45671307

ATTAATTCCACTACTATTTACCAAATAAAATGAAAACACTAATTCAAAAAGATATATGCATGCCTATGTTTATTTCAACATTANTTAGAATAGCCAAGATATGGAAGCCACCCAAGTGTCCATCCAAAGATGAATAGATAAAGGAGATN[TCACACACACACACACACACACACACACACACACACACACACACACACAC]TGGAGCATTGTTCAGCCATATAAATGAATGAGACTTTGCCACTTGCCACAACATGGTTTGACCTAGAGGATATTACGCTAAGTGAAATAAGTCAGGCAGAGAAAGAC

>A1:46566288-46566588|varpos=46566438

TTTCTACATTGAAGTTTGGAGGGTTGGAGTGTTAACAAATGCAAACAGCTGAGAGGGGAATCGAATAGAATAATATCTACTACTGTAAAGGGTGTACCNTGTGCTTCAGTTTCATACCAGGCATAACCCTGTGACACACNCGNGNGCGC[GCACACACACACACACACACACAC]ANNNNNNCACACGGGGACTTNTACAGGAAAACTCTTTCTAACATTTTTTATAAAAGTGAAAAATTGAAAACAACCCAAATTTTCATCATCATGAAAATAGATATATATGTGGCAGATTTTNAAAAATGGAATACTATACAGCAGGGACT

>A1:49016912-49017212|varpos=49017062

CTTCCTAAAGAAGGAAGAAAGGTCTCAGATACACAACCTAACCTTACACCTTAAAGAGCTGGAAAAAGAACAGCANATAAAACCCAAAAATAGAAGANAGGAAATAATAAAGATTAGAGCAGAAATTAATGCTACTGAAACAACAACAA[CAACAAAAAAAAA]AAAACAGTAGAACAGATCAATGAAACCAGAAGCTGGTTCTTTGAAAGAATTAACAAAATTGATAAACCACTAGCCANTATGATCAGNAAGAAAAAGGAAAGGACCCAAATAANTAANATCAAGAATGAAAGAGGAGAGATCACAACCA

>A1:49083369-49083669|varpos=49083519

ATTTCAGCTCAGGTCATGATTTTGCCTCAGGTCATGATCCCAGAGTTGTGGGATNCAGCCCAGTGTCAGGCTCCATGCTGAGTCTGGAGCCCGCTTAAGACTCTCACTCTCCCTGCCCCTCTCTACGGTTCAAGCACTCTCCTTCTCTC[TGAAAGAAAGAAAGAAAGAAAGAAAGAAAGAAAGAAAGAAAGAAAGAAAGAAAGAAAGAAA]NNNNAGAAAGAAAAGAAATAGAATATGACACCATCCATTTTCATATCACTTTTAATATTTCCATTATAACCAGAGATANGCTTNTNTTGAGGCA

>A1:49730150-49730450|varpos=49730300

TTGCTAAATGTCCTATAATGCACAGAAAAGCCCTTTCCTTAAAAGAATTTCCTAGCCCCAAATCCCANTAGNGATGCCTATTGAGAAACACTCATTGAGGTGATGTGCAAATTTTTAACAATNGGACCAATTTTTCTCTTGTCANNNNC[ACATTTTTTTTTT]TTTTTTTTCTTCTCTCATACAGTCAAAAGTCATAGGTATTTTCTTGACTCAAACATTTATGCCTTTATTTAAAACTTTGATATTTTATTTATTTCAACATTTTTGCATTTATTTTTTAAAATATTTCATCAAAATATTATTTATTTTGAC

>A1:51305923-51306223|varpos=51306073

TGGGAGCAAGATTCTAGTGTATCTCATTTCCTCACTGATAAATCTGTGAGTTACGACAGATCTGTCTTGGGACTAGGTCTAAAAAGTTCTCTTCTCACGTGCAAAATGTTTTGCTTAATTCATGCTCTGCAGTTTGAAGACNTTTTTTT[TTATATATATATA]TATATATAATTTAGAATTGATAAGGGAACTAGTTTCCTGCCTTAGAGNTTTTATCAGATAAAATCAGGTTTTATCAGAGTTTATTTTATCTTTTTTAATCAGGTAAAACTCAGGTTTCCTTTCAAAGTGATTCATGATNACGATNTTCTC

>A1:51386921-51387221|varpos=51387071

CATTCTGGGACATACATGCCNGCTTCTTTGCTTGCCTATAATTTTTTATCAGATTTTTAAGTTACTTGGAAAGAGTTTTATCTTTCCAAGGATTGCTTTTAAGCTTTATCTGGCAGGTTCAGAGACATATTAATTTAGGACTGATTTTT[TTTCCCCC]CCCCNATTGCTAAGACAATATCTGAGTATTCTGTACTCCATAAATTATGAGGTTTTTCTACTNTGGCNTCTGGAATCAAACTATTCCTAGTNTGTGTGGGTTCNAAAGATGGTTTTGCCTATTTATTTTTANTGGTTCTTTCCCACAGCA

>A1:53419627-53419927|varpos=53419777

TATTTTAATTTGTTAGAGATTTCCTTAAGTTAAAATTCAACATTTTCTACTTANNNNNNNNACCATGTCTGCTAATATTTTTAATCTCNAAAAGTTTTCCTTTACTTGTCTGGATATTTATTTTTTTTATTATCATGGTCTTTTTTTTTT[TTTTC]CTGAGGAAGTAAAATTTCCTAGCTCTAAGAGGTAGAAGTTAAACGTGCATGCACACACACGCATACACACGTGCATGCACATACACACATACACTTAGAGCTTTCTAGTGCTTCCATCATTTTATTTTCTCTACATTTGTTATTTCTGTT

>A1:53648479-53648779|varpos=53648629

TGTAGGGGAAAGGCTCTCAGTTCTTCCCCANTGAGGATGATATTAGCTGTGTGTCTTCTGTATATGGCCTTAATCATGTTGAGGTATGTTCATTTATGCCTACTTTCTTGAGGGTTTTTNTCAAGAATGGATGCTGTATTTTGTCAAAT[GTTTTTTTTTTTTTTT]NNNCTGCATCTATTGACAGGATCATNANGTTTTTATCCTTTCTTTTATTANTAGGTGTATCACATGGATTGATTTGCAAATAGAGAAACTTCCCTGCAGCCCAGGAATAAATCCCACTGGATCATGGTGAATAAGTCTTTTAAAGT

>A1:54162271-54162571|varpos=54162421

GTGAGGTGGTATCTCATTGTGGTTTTGATTTGCATTTCCCTGACAATGAGTGATTTGAGCATCTTTTCATGTGTCNGTTAGCCATCTGGATATCTTTGGAAAAGTGTCTATCATGTCTTCTTCCCATTTCTTCACTGGATTATTTGGTT[TTTGGGGGGGG]GGGGNNNNNTTGAGTTTGATAAGTTCTTTAAAGATTTTGGATACTAACCCTTTATCTGNTNTGTCATTTGCAAATATCTTCCCCCATTCTGTCAGTTACCTTTTAGTTTTGTTGATTGTTTCCTTTGCTGTGCAGAAACTTTTTATCTTG

>A1:54287660-54287960|varpos=54287810

ACAGCAACAGCTTTGTNGGTTAGGAAAATGGCANTGTTCCAAAAAATCTTTTATATGAGCATTAAAGACAAAATGCAGCAAACTTCAATCTTAATTGGAAATATTGAAAAGAAACTTTGAAACCCAGAGGATGGGAATGAAAAAGAAAC[CAAAAAAAAAAAA]TGATAAACAAGAAACACAGACACTTCAAAATAAATTCCTTTAGGAAAAAGGTTAAAATTACATTGTTGCTTCATAGAGAAATCTCAATGGATAGTTTCTAATTTACATCTGAAAATAATGTTTTCTGATCACAAATGTCTAAA

>A1:55256511-55256811|varpos=55256661

ATTCTGACACAATCCAATTGTAAAGAAGACAACAAAGTTAAAAGAGGTTATTCCATTTCATGATGGCAACATAGGAAGATCATGAACTCACCTGCTCCCACAGACAAACTGTCTACAGCTATATATGGAACAATTTCCTGTTAAAAAAA[AAAAAAACACATAAA]NNGACTGGCTGAGTGATGACTACCTATTGGACAGATGAGAAGAANACCACGTTACAGTGGGTAAGAGAAACTGGGCACAATCTTGCAATAAAACAANTCCCAGTATTGACCTACAACTGGGAGAAAACTCAAAACCTGAAGCTTCTCCT

>A1:55438633-55438933|varpos=55438783

GATTTTATTTATGCTCTACTACAATGAAGAAAGTAAGTTTTGCAACTAATAAATATGACCATAATTTTTCTTTATAAGCATATATTTCTAATTCTGTTTCTTTCACCTAATAATGCATTAGGTTTTTCCTTATATGCTATTACTAATAA[CAAAAAAAAAAA]GTTGCTTTATCTTACTAAGANCACTTATNTATTTACCCCTAAANTGCATTAATTAGATCTGCTACTATTCAACTTNCTATTAATGAGATCATGCTGAATGNTACTTTGTAGTCTACTCTTCTGCTAATATTACATTTGAAAATC

>A1:55680493-55680793|varpos=55680643

CAATTACCTCCAATTCATATAGATTAATTAGAAGTATACATGAATTTAGTNAAAATGTTTTNNNATGAAAGAGTGGATTAATGCTAGAATTACTGATCTGCAATTCCCAATTACAATTATATATTCTGGTACTAAATTTGAATTACATTATTG[TACACACACACACACACACACACACACACA]ACCTAAAATCTAGCTGATAATTCTGATTCTTTTATTCCTATGACTCTTAATATCCAGGTCACATGGGACTTAGTCCTAACTGATTTAATAGGNCTCATTCTCAAATCTCGTTTGCATTTCCATGATAGGCT

>A1:56079905-56080205|varpos=56080055

TAGAAGTTCTAGCCAGTGTTATCAGGAAAATAAAAGAAAAAACTAAATAAATGCCAGCCAGATTGGAAAGAAAGAATTTTAAAGGTGTATTTGTCTGTCTATTTGTTCTTCTGATCATATGATCTTAGAANTAATCCATTAAAANNNNN[AAAAAACAAAAACAAAAACAAAAACAAAAA]CAAAAACAAAAANCTGCTNGAAATAATGAGGTCAACAAGTTCATAGGATATAAGATCCAGATACCAAAACCAATTGATTTCTAGACATTAGTAATGAAAAATCCAAAAATGATTCCAAAATTGTTTTACTTGTCTTTGTATTATT

>A1:58834851-58835151|varpos=58835001

GGTTCCTTTTTATTGTTGAGTATTACTCTATTTGATGGCTATACTCCTATTAAGTATGNTTCCATTTATGTATTTTATTTCAATTTATTTTTATTTTAAGATTGATGTTATTCCTTAGACCTTGGAAGTTTAAAANNNTAATACAGATTTACT[GTTTTTTTTTTTTT]TTTTTATAGTTCTGTCGGTCAGAAGTCCAGTTTAGCCTGATTAATTTTTCCTTTTGATAATTCACAGCACCAAAAAAANNNNNNTCAAGGCATTATCTGGCTGAATTGGTNGGCTGAGGCTCTGGGAAAAATTTACTTCAAAGGTCAGTNAGGTTGTTG

>A1:59280322-59280622|varpos=59280472

ACCTTTAGTATTACTAAGTACATTTCCTCTGATAATTTCATTTTCTAAAGACAATTAGATGGATTTTTGAATGTGTTGTGCTTATTACACAAACTCTAATCTATGTGCTCAAAATATTTTAAAGTCATATAATTTTGCTGTAAATGCTT[TCTTTACTTT]CNNNTATGTATTTGGAGTTATGAGTTATTTCTTGACAACTNTTTTTTTTAAGAGAACATACTATATAGGGTTTAGATCAATTAATTCTGTTGTCCTTAATGNCCTTAATATACCTTAATGAAAAGNATATTATGCATACATAATCATATA

>A1:60092118-60092418|varpos=60092268

AGGATAGAAATTCAATTAGAGAGANNNAAGCTGAAATCAAGATATGTAATCACTTGTAGAATTTTTGTTTGGACATTATCAGAAGTGGGATGACTGACTACCAAAGGATTTTAATTAGGTAAGTGACAATTTCAAATACACGTNTAGGAAAAAAAA[AAAT]TCACTCTTCCTACAGGAAGGGAGGATAATTATTAAATGCTGGGAAAGTGGTTATGAGGTTATTGTAGAAGTGCTGATCCAGAGAGATGATAGTTNTCTGTAATCAGGATGNGACGAACAGTAGAAATGGAGAGACCTAGGGTGATACAAA

>A1:60524287-60524587|varpos=60524437

TACCTGTATGATTTTGANGGTTTTCTGTCTGACATTTAGGTCTTTCATCCATTTTGGATTTATTTATGTGTATGGTATAAAAAAGTAGTCCAGTTTCATTAGCCAGTTGATTAAGNATGCATATAATGGGTGGCNGATCTATNNAAAAA[AAAAAACAAAAACA]ATAAAAATAAACCACAAAAGCATAATTATCACTAATGTCAGNATAGTGGTAAACTTGGAAAGACAAAAGTATGTCTTAATATAAAAGGTTACACAGGGAATTTCTGAAGAGGTACTATTTTTGAAAGGGCAGACATGCTTTACACCCTCT

>A1:60624830-60625130|varpos=60624980

GTTTCTTAAATTCCACATATGAGTGAAATCATATGATATTTGTCTTTCTCTGACTGGCTTATTTCACTTAGCNTTATACNCTCTNGCTCCATCCATGTCATTTCAANTGACAAGATTTCATTATTTTTTAATGGGTAATATTCCATTGT[TACACACACACACACACACACACACACACACACACACAC]NCACATCTTCTTTATCCATTCATCAATCANTGGACACTTAGGCTGCTTCCATAATNTNGATATTGTATATAATGCTGCTATAAACNTAGGGGTGCATGTATACCTTTGAATTCATGTTTTCTGTGTTCTTT

>A1:60781478-60781778|varpos=60781628

TATCTTTCCATTTCTTTATGTTATCTTCAATTTCTTTTATTAGTGTTTTATAGTTTTCAGAGTAGAAGTCTTTCACTTCTTTGGNTAGNTTTATTCCTAGGCCTTCTTATTTTGGATGTAATTGTAAATGGGATTGTTTTCTTTTTTTN[TTTAA]ATTNTTTTAATGTTTATTTATTTGACAGAGANNNNNCNGGGGCGGGGGGAAGAAGAGAGAGAGGGAGACAGAGAATACAAAGCAGGCTCCAGGCCCTGAGCTGTCAGCACAGAGCCCGATGTGAGGCTTGATCTCANGGACTGCAATATC

>A1:61110661-61110961|varpos=61110811

TGCAAAGTATCATGCAANATANTGGAATACTAATGACATTCTATAGTATCAAATCTATAAAAGGCTTTTTATCACNATTTCTATCACTNTAAACATATACTAAGATANAATAGTTTTTAATTTGTTATTTAATAGACAGATTTAAAAAN[AAAATTT]NNAACGTTTATTTATTTTTGAGACAGAGANNNNGTATGAACGGGGTGGGTCAGAGAGAGAGGGAGATGNAGAATCTGAAACAGGCTCCAGGCTCTGAGCTGTCAGCACNGAGCCCGAGGAGGGGCTCAAACCCACGAACTGCGAGANCGT

>A1:61343097-61343397|varpos=61343247

ATGGTTCATGAGTTTNGGCCNGCATTGGGCTCNGCACTGATAGTGAAGAGCCNGCTTGGGATTTTGTCTCTCTCCTTCTCTCTCTGTTCATCCCCTGCCTGTNNNCTCTCTATCTNTTTCTCCTCAAAATAAATGAATAAACTTAAAAA[AAAAAACAAAACAAA]NNNNNNNGAAACATAATTGATCAATATTACTAAATAAACCGAATCAGTAGTTTAAATAAACTTCCAAGGAATCAACAGAGAGACAAATTCTAACAAATGTGTAGGAGNNNNNNNNNTANTTNNNNNNGTTCATAGAAGATAAAAACANT

>A1:61869494-61869794|varpos=61869644

TATTTTTAATGTATNTACTATGCACACAACACACANNNGTGTGTATATGAATATTATATAGATAAAAATACAAATTATATATGCATNNATCAAAGAAATATAATAAAGCAGTAAGTTAATACATTTGCCAGACACCAGATAGAAAAAAA[AAAACC]CNTAAAATCTGATAATACCAATGGTTGGTGAAGATATGCAGAAACAGNAACACTCATATGCTTCTGTTGTAAGCATAAATGGGTACAAATACTTGATAGAAATATTTGCNGTTATCAGTTAATTGAACATGTACATGAATCAAGTCCTGT

>A1:62250017-62250317|varpos=62250167

ATGAAACTTTATTGAATACTCNAAGTTATTTCATCTATACAGAGTTATAAGTATTCCAGAAGTCAATATGAACCAGGAGAAAAAGGGTTTCAAGNGAAAGGAAGGNAACCATTTGAGAATAGAGCCATGANNNNNNNNACTAGGNAAAA[AAAAAAGGAAAAGGAAAAGGAAAA]NGGAAAAGGAAAANTTGTATTCAGAATGAAACATTGGATGACTGCAGGNTATAGNTTTCATTGTGAGACCCAAAGCAACATCNCTTTGAAAATTTAATCAGAGAGCNGGCAATCAATGACCCTGTTTTAACACATCCCAGTTAAACA

>A1:62565138-62565438|varpos=62565288

TCGTCTCACNTCATGATCTCAAGGTTTGTGGGACTGAGCCCTGGGTCAGCTCTGCGCTGACAACCTGGGATTCTCTCTCTCCTTCTCTCTCTGCCACTTCCTCTCTCTCCCTCCAAATAAGAAAATAAACATTAAAAATTAAAAANNNA[AAAAAGAAAGAAAGAAAGAAAG]AAGAAAGGGAACCCCTCCCATGCCTCTGAGACCCCACAGGGATTACTGACATTGCCATTCAACCAGGTGCCCGAGTCAGAAACTTAGGAATCATCCTACACTCTCCATCCTCCCTAGCTCCCACACCAAATTATGTAATTCTGCCTCTT

>A1:62888067-62888367|varpos=62888217

CACAGGGTANGTCTGAGTCACATTTTTAAAACTGAAACTTANCTCCNTTTCTCCTCAGTGTCCACTTCTTTTTTACATCCCTCCTTCCATCTATTANNCCCTCATTGATCCATTGATCCATCCATCTATTCATCTATCTATCTATCTAT[CTATCTATCTATCTATCTATAT]TTNCATCTTAACTTTGTCTAGACNTTCTTCCACTAATCAAATATTTATTGATTGGTCCAACAGGAGGCACTGGGAATATGAAAAACAGCAGAGTGATGGTTTCTGTTCCCATAAGATATTTTAACTAAGATACAAGTTCAGCTCCGCCA

>A1:64785718-64786018|varpos=64785868

GAAAACTGTGGATTGTTTGTAAACTAGCCAACAATGTACTTTAGTCGGTTTTTCTTGGCAAATTCACATTAAATGTCATCCATCGCCAGAAATCTGTTCCATAATGATGTCATCAACAGCTTGGGATGAGAAAAAGCTACNAAAAAAAA[AAAAAC]CTAAAAGAATGCAGATGGGAAAGTAATTCNTGCATATTTTATTGACCATATGTTCCACTCCCGCCACCCCCTAATTTTACTTATTGGGCTATCAAACTAAAATNCTCATTTGAATCTTGTAGTCTTTTAAGTGTATTTTNNNAATTTTTCCCCC

>A1:65269726-65270026|varpos=65269876

TCCCGGTGTTGAGGGTCTGGGGCACACCCAGACACCAGTTTTTAATCACAGTGACAAGAGCAGCAAGAACAGAACANCTGAGTACCTACCAGGTGCCCGGCAATGTGTGGCCTTAGGGACTACAGACNCATTTCTTACTCGTTTTNAAA[ATTTTTTTTTT]TTTTTTTAATGTTTTATTTATTTTTGAGAGAGAGANACACACAGAACACAAGTGGGGAANGGGCAGAGAGAGGGGGAGACACAGAAACNAAAGCGGGCTCCAGGCTCTGAGCTGTCAGCACAGAGCCCNANGNGGGGCTCGAACCCATGA

>A1:65804150-65804450|varpos=65804300

TAAAAATCAAGTATGTCAGAATTCAAGACAGNGAATAAATAGAAATGAGGCTGGAGGGGGAGGAAGGAGATCCCAGAGCTTCAAGTCGGCCTCTTTTGTAATTTTTTTAAAGTTTATTTATATATNNNNNNNNNNNNNNNNNNNNNNNN[AGCACGCAC]NNACAACCTGGGGAGGGGCAGANAGAAGGAGAGACAGAATCCCAAGCAGGNTCCANGCCATCAGCACAGAGCCCGAAGCGAGGCTCGAACTCACAAACTGTGAGATCATGACCTGAGCTGAGCTCAGGAGTCAGATGCTTACCCGACTGT

>A1:68626358-68626658|varpos=68626508

GAAAATTTGGCTTTAAGGTTTTGATTCCNNNNNNNNCAGGATCGTTTTGACAATGTAAGCATTAAATGTTGCTGTTTGCGAGCCTGAACTTTGACCCTTTGTGTTTTGTTTGAACTTTGTGCATACGTGCCCAAATGATTCCTTAAAAA[AAAAATTTTTTTTTTTT]TTTTTTTTGTAAGTTTACTTATTTATTTTGAGAGAGAGAACCCTAGTGAGGGAGGAGGGGAGAGAAGGAGACAGAATCCCAAGCAGGCTCCACACTATCAGCACAGTGCCTGATGCNGGGCTTTGTCTCACGAAGATATCAGAACCTGAG

>A1:69957728-69958028|varpos=69957878

AGTGATACAATGTCCTTTATTCCCTGACTCCAAATTACCTTTCCAGAATCATCTTCCCATACCATGTACCTGAGTCACATTAAAATTGTTGNNNNNNNNNNNNGTTTATTTATTTTGAAAGAGAGAGGAGGGGAGGGGCAGAGAGAGAG[AGAGAGAGAGCGCG]CGTGAGCATCCCAAGCAGGTTCCACACTGGAGTGTGGAGCCTGATGCCAGGCCCANACTCATTAACTGTGAGATCAGGACCTGAACTGAAATCAAGAGTTGGATGCTTAACCGACTGAGCCACCCAGGCACTCTTAAAATTCTTGCTTT

>A1:71435539-71435839|varpos=71435689

GATCATTTAATTTTGTAATAGGGATCCATAAAGTTGGGACAAAGAANGTGAAAAGTTTCCAAATAGTAGTTTCTAATGACTACTGAATCTTTGCATCATCCAGGGAGATATTTTTTNAAAAATTTTAAATGTTTTATTTATTTTTGAGA[TAGAGAGAGAGAGAGAGAGAGA]NNCAGAATGTGAGCAGAGGGGCAGAGAGAGGGGGAGACACAGCATCTGAAGCAGGCTCCAGGCTCTGAGCTGACCTCACAGAGCCAGACATCGGGCTAGAACCCACAAACTGTGAGATCATGACCTNTGCCAAAGTC

>A1:73278591-73278891|varpos=73278741

TTGTTTTTTTCCCACCAAAACTCTTGGAAGGGNAAACATGATTCNAAACTCTAGGAAAAATGNCTTTCNAATGCATCTNGTTTTCAGTGAAGGAAGTCATGGAATATTTTGGCCAGATACTTCCANTTAAAACTCTAGGATTTTTTTTT[TTTA]ATCATGCCAGTTTAAAATAAATACTTAGCTAAAATATGGCACATGTCAAATCTAGAACAGANTGTCTCCCAGCTTCTAAATTCTGTGCACNCCAGCCAGATAGATGGAATGTGAATTTTGGTGAAATACGAAGTTACTGCAATGAATAAG

>A1:74457287-74457587|varpos=74457437

CTTTTGTCATCATTCNTATAAAAGTTTTATCTCAGTCACCATATTTTTAAATTNTGTCGCCTTTATNGATTTTTTCTAGTTTTTCACTGNCACNTGTANTACATTGNCTTTTCTTGAGTGNAAGAAATCCATCCTATTTTATTAGAATT[TAAAAAA]AAAACATTGTTTATNTTTCTCAAAATGTTACCTTAAGGACATTTCCAAACAGAATAAAATGNAAGTTATTTATAATAGCTTTTGACTGTTTCCTCAAATAAGGTAAGGAATTTACCATGAATTCACATTCCTCATGCTCCCACTTCCTGA

>A1:74512041-74512341|varpos=74512191

TTCCATAAAAATGNNNNNNNCCACTGGGTTGTATTCATAGTAGGTAAAATTNATGCTAGTATTATAAAAGNNNNNNNNNNNNTAGTTCGGTCTTAAAATGCTGNATTTTAAAGTTAGAGGTTTCTTTCAGCNCNTGCTTTTTTTTTTTA[AATTTTTTTTTTT]ATGGTAAGGTCAGTTTTGTTCTNGATTAAATGTATTTTAAAGATGCATTAAGCAACTCTTCATCTTCTCAAATTTTCTTCCTTTTCTCATTTTCCCNAAACNAAATACTTATTCCTGCATTTNCTTACTCTGNTCCATATGG

>A1:75092184-75092484|varpos=75092334

AATTTTTATTTTTATAAATAAAATAACNTGGGTGGCACCTGGGTGGCTCAGTNGGTTAAGCATCCACCTTCGGCTCAGGTCACGATCTCGTGGNTNNTGAGTTTGAGCCCCATGTCAGCGCAGAGCCTGTTTGGGAGTTTCTCTTTCTC[CCTCTCTCTCTCTCTCTCTCTCT]NNNNNNGCCCCTCCCCCACTCANGCATGTGCACATGCACNNTCTCTCTCAAAATAAACTTAAAACTCGTTTTTAAATGAATCAATAAATCGCAAGTGAATTCCATCTCAGACTGCNNCCTTCAAAGGCAACATGAGTCAT

>A1:76452100-76452400|varpos=76452250

CAAACAAAGGAGGNCCTCACATTCAGANTCTAAATGAGATAATGCGTTCATTAATGGTGTGTGTGAAAATATTATTAATGAGTATAAAAGGAAGCTTCGAAATGTTACAACTTGTATGATCTTGCTTGAGCATTTCCTCATTNNNNNNN[AATTTTTTTTT]TTTTTTTAATGTTTTATTTAGCTTTGNNGNAGAGTGNGAGCAGGGAANGGAAGAGACAGAGGGGGACAGATGATCCAAAGGGGGCTCTGAGNTGTCAGCACAGAGTCTGATGCAGNGCTCAAACTCACAAGCTGTGAGATCATGACCTGA

>A1:76630825-76631125|varpos=76630975

AACTGTCTCTCAATTGACTTCTTTATTTTATTTTTCACTGTCAGGATCCTTTTGAAAAATAAAACACAGCCTGTTCCTTTGGGTTCAATTTCCCATTTAAGATTTACAAACANTTTAGAGATANACCCACAGTTTCTTATGGCCATATC[ATTTTTTTTTTTTT]TTTTTTTTACCATATCATTGTATCCTATGTGATTTAANAGNGATAGCAATAGTACTTGTAATTTTNAAAATCAAATTTGGTTANANTGATATCTTAATAATGCAGATATATACCCAGGGGAGCATCATCCTTCTCAAGTTAGACATATTATGTT

>A1:76707422-76707722|varpos=76707572

CCNCCCATTTGTGTATGGCCAGCAAAGTAGCAGAGATGGGACAAGGGGGGCATGCTAATGAAGAGAGCCAATGATGTCCTGCTTCATTANATTAAACAACANATTCTTGAGTTCCCATTTTGTGCTAGGTCCTTAGAGANATGCAAANN[AATATATATATATA]ATATAAGGCATACGTTCCCCACTCCCCACCCCAAAGAAATTAACAGGCAGTTGAGGAGCTACATGAGAAATGAGAGTGTTAACNTCTTCCAAAAAANNNNNGACCAGCTTTTATAAGACAAGAGAAACTGATGTAACTAGTCTGGNNGGTTGCCTT

>A1:76970439-76970739|varpos=76970589

AAATGTAGAACTTAATACTTCATGCCACNGTTTAACTACAGCCACACAGGTTAAATTCCCAGTATCACTAGCTCAGGAAACACAATAAAATGTATTTCAGGCATTTAGAAGCCAATACAATATTTTACNATAGAAAATCTAAAAGGTTT[TATCATCATCATCATCATCATCATCATCATCATCAT]TCATCATCATCATCANTTCACCTCACNTGANACATTCCATNAGCTTTTTGCCCTCATCCTTCTATAAAGACTCCTCTCGACAATGTNACCAACAGCCTCCTTGCCATCACTGTAACAGCAGGCACTCANCCTCCTGGTGCATTCAG

>A1:82322746-82323046|varpos=82322896

GAAATTTTCTGAGTATGTTTGTTNAGTCCATCTGGTCTAGTGTGTCATGAAAAGTCACTGTTTCCTTGTTNGCTTTCTGGTTAGATGATCTTCCCATTGCTGTAACTGGGGTGTTGACGTCACCTACTATTATGGTATGAGTAAAATGA[GTTTTTTTTTT]TTTTTTTATGTTTGTGATAAATTGATTTATATATTTGGGTTCCTTCACATTTGGAGCATAAATGTTTACANTTGTTGGNTCTTTGTGGATAGACCCCTTAATTACGATATAATGCCCTTCTTCATCTCTTGATACAGTCTTTATTTTAAA

>A1:82863550-82863850|varpos=82863700

AATAACACCGAGTGCAAGTAAAGAAGAGGGTGAAGATGGTGTTGATAGAAGAGCACATGCAAAGAGAGAAANGACAGGAGTGTTGGGGTGGGGGAAGAAAAAATANACGTTGATTAGGNAGAGATACAAAAGACTTAATCCAGAGAGAG[AGAGAGAGAGAGGGGG]GAAAATAAGGAGGCAGGGAAAACGAAATGAAGATAAAATTACCCAGTCAGAGAAAATATATGACTTGATTATTCCAGAGAGTGAGAAAGGAGTGTAAAGAAGNNNGAGGTGTATCAAGCCACCAAGACATGTATCAAGACAATGGATTAAANATA

>A1:83592854-83593154|varpos=83593004

TAGAAGTCCAGAAGGGAGTGGCAGGAGGAATTGGCAAGAAATATTCAATNTGCTGTATAGGAAATANATAACCAAGAATTCTTTATCCAGCAAAGNTGTCATTCAGAATAGGAGAGATAAAGATTTTCCCAGACAAAAGACAATAACAA[AAAAGAAAGAAAGAAAGAAAGAAAGAAAGAAAGAAAGAAAGA]NNNGAAAGAAAAAAGGAAGAGGAAAGGAAAGNAAANGAAAAGAAAANNNNNGAAAAAAGAANAGAANAGAANANNNNNNNNNAAAGAAAAGAAAAGAAAAGAAAAAGAAAAAAAGAAAAC

>A1:84060824-84061124|varpos=84060974

AATCTAGAGGAGAACACAAGCAGTAAGCTCTTTGACCTTGACCATANCAATTTCTTACTAGATAATCACTGGAGGGAAGGGAAACAACAACANAAAAAAATGAACTATTGTCACTTCACCAAATGTAAATTTTGATTTANTTATGATAAAAACTC[TAAAAAAAAAAAA]AAAAAANGAGAATTANAGGGAGCATACCTCAACACAGTAAAAGCATATATGAAAAAACTTTCAGCTAATGTTAAACTGAGTGGTGAAAAAANNNNNTTAGAGCTTTTTCTCTAAGATTAACATGTTCACTCTCAACACTTTCATTTAACAAAACAC

>A1:84499714-84500014|varpos=84499864

TATCAGTACGGAGGAAATGCTTGTTATGTTGANCTCCTANCTGTTGACCTCAGAATAACTTCATCCTCCTGCCAGTTCCTTATGGTCATAAGAAGACTGTNNCATCTTCAAATAATTATTGGTATGAAAGGAAAAAANCCATGAGAAAT[TAAAAAAAAAAAA]AAAAAAANTAATGATTGACAGCACTTAAGAGTGGCNTTTGCCACTTTCTGGCAAAACTCATCCAAGNTTTTTCATTTAAATCTAATTGATCATCTCTAGTTGAATGGATATCTATAANGGTTAATACTTTTAGCTAATCATTTGATTCCC

>A1:88177507-88177807|varpos=88177657

ATTTCTGCCGTGTTGGTGGTTACTCTCCTGTCTCACTTCTGANTATATTTATTTGGGTCCTTTCTCTTTCTTTTTTGATAAGTCTGTCTAGAGGTTCATAAATTTTATTAATTTTGTAATAATCATTTCCTGGTTTCATTGATGTGTTC[CATTATTATTATTATTATTATTATTATTATTATTA]NNNNNNNNNNNNNNNNCATTTAGTTTCTATATAATTTAGTTCTGCTCTAACCNTTATTATTTCCTTCATTCNGATGGCTTTTATCTTTGTATGTTTTTCTTTATGTAGCTCCTTTGGTCTTAAGGTTAGGTTGCTTA

>A1:88228283-88228583|varpos=88228433

CTCCNTCCTACACTCAACANCCCTCAGTTTATTCTCAGTTTTTAAGAGTCTCTTACCGTTTGCCTCCTTCCTTCTCTGTAACTTATAATCACCTAAAATACATTAAAGAATTAAATGTCGAAACTGTAAAACTCCTAGAAAGAAAAAAA[AAAAAAC]CACTGAGGTAAACTCCTCCATATTGTTATNACTTATGATTTTGTAGATTTGATACACACAATACAGACAATAAAAGCAAAAAANNNNNCAACTGAGTAGAGCTATATCCAAGTGAAGTTTCTGAACAGCAAAGGAAATAATCAACAAATGAAAAAG

>A1:88514544-88514844|varpos=88514694

AAAAATCAGATAAATTTATTTTGGATGTAAAATGACTCATATTGGAGTAAATCATGTCAATTGCATATTCCAATGACTATATTCCTGTGTTATATCAAAAGCATTATATACTNCTAAACAGAATAGACCCAGAATCATTCATTCATTGA[TTAATAATAATAATAATAATAATAATAAT]GTATATGTATTNGATGCTGTCATATCAANGCATATATTAGGATAAGGCACAATGTAATAGTAACTAGGGTAAATATAGCCCTTACCTTCACAGAATTTAAAAATTTAGAATTATATTCTGCAAATCTTTTAATATAC

>A1:88595410-88595710|varpos=88595560

GAATTACTTTCAAACAATTCATCCCAAAAGCAATACATGTTTGTTACAGAAAAATAAGAAAGCAAAGACCATTAAAGAAAAAAAATAATTCCTTAATCCCATATACCCATANACAATAATTATTTACAAACTTACATATGTGTGTGTGT[ATGTGTGTGTGTGTGTGTGTGTGTGTGTG]GATAAGAACACATAAGGCCTACTCTCTAAACATANAGGNATCTTTCAATACTTCCCCATGTGTTAACTAATTNTTTTGCTCCAGGCNTGGAGTCTGATATCTTAGAGATACTAAGCCAAATACAT

>A1:88600602-88600902|varpos=88600752

AACTTCTGCAATCAGACACTGTTAGGAAAATAAAATAATCCCCCAACTTAGAGNTTGGGATTGAATATTTGCAAGTCACACATCCATCATGTGAATTTCTTCCATAATTGTTAAAGAACATTCAAAACTCTTCAGAAAGGAAAAAAAAA[AAAAACAAACCAA]AATAAGCCAATTCTTAAAATGAGCAAAGCAAGAGATTGATTTAATGAAAAGGATATATTGGTCATTAGACATTAGGAAAATTAAACACATGATGAAATGTCATTGCACACNTATTAGTATAACAAAAGTTAAATAGTGACACTCCCNAGT

>A1:89113456-89113756|varpos=89113606

AAAATAAACATAAAAAATCAATTTGTCAATAAATTATATTNAAAANTTAAAAAAATTAAGTTTTAGGAGAAAAAAANNNNNNGAAAATCTATAATTCTATATTGCCAGAATTTCAATTAGACTACAATAAAAGCATGTTTGCTAAATANNNNNNNN[AATAAATAAATAGATAAATAAATAAATAAATAAATA]NATANATAAATANATAAATAAATGCTTTTAATACCACATACAAGTCCGTTTTTAACCCAAATGTCACCAGGTTATATCCTATTTAGTGTCCATATATATCAAATTTATACCTGATTTTGGTTAGTGACTTGAAACCATAGCACACTA

>A1:89139059-89139359|varpos=89139209

CACTAAAATTCTAACTGAGANNNNNNNNNGATCATGTATCTGTGATAACTAGCNATCAAAACTTATGTTTTCAATAAATGTGTACTGCCCAGATGACTTTTAAAACACTTTTTGTCACCTGCAAGTATTTTTAAATTTCAATTTACGTN[GGGGTGTGTGTGTGTGTGTGTGTGTGTGTGTGTG]TGTGTGTAACGAAAGGTNNNNNNNNAAAACACTCAAAAATATAGCTTACATTTGGATGAAATTCTCAGGAAGAAGTACAATGGGGGTGAGTTCAGGGAGGAAAAAAAGGATCATGTA

>A1:89535032-89535332|varpos=89535182

TAGATGGCTCAGTCAGTTGAGTTTCAAGCTTGTGATTTCTGCTCAGGTCATGATCTCATGGTTCATGAGTTCAAGCCCCACNTCAGGCTCTGCACTGTCAGTGCAGAGCCTGCTTGGGATTCTCTCTCCTTCTCTCTCTGCCCCTCCAT[GCTCATTCTCTCTCTCTCTCTC]CTNGCTCTCTCTCTCTCTCTCTCTCAAAATAAATCAATAATCATTAAAAAAAANNNTCCTGCAAGTAACATACTTAATGGTGAAAAATGAAAGCTTTCTCCCTAAGATGGAGTACATGACAAGGATGTCTACTCTTTTATTCAACATAGA

>A1:91038546-91038846|varpos=91038696

TGTCCAACTTCGACTCAGGTCATGATCTCAAGGTTCCTGAGTTCGAGCCCTGAGTCAGGCTCTGTGCTAACAGCTCAGAGCCTAGAGCCTGCTTCACGTTCTGTGTCTCCCTCTCTCTCTTCCCCTCCTCCACTCATGCTTTGTCTCTC[CCTCCCTCTCTCTCTCTCTCTCTCTCTCTCTCTCTCT]TCTCTCTCTCTCTNNNNNNCTCTGTCTCTCAAAAATAATCAAAAATTAACATNAAAAAANNNNNAAAGAAAGCTTCATCTCTGTCATTGCTGAGCAAGATCCCCGTTGCATGGATATACCACAATTTGTTTCTGTGGTAGGCAACAT

>A1:91289615-91289915|varpos=91289765

CTTTCAGTGGGTACCAACCCAGCTGAGGTCACTGGGATACATTAACGGTCACAGCCAGCACTCAGACAGCACATGCAGCAGGCCAGGCACTTATGTGAACTTCCCGAAAACTCNAGAAGGTGAAACTTTGTTTTTTGTGAGTTTTTTTT[TTCCCC]CCTNTATTTTGAAGATGAGGAAACTGTGGCACAGTGAGGGTTAGGAGGCCTGAGAAAGGATAAGAGCNTTCACAGCTGAGACCCCTTCTTCCAAAGTCATCTTCCTCCCTGGCACCTGAATAGAGGTGCCCTTTCCCCTGTGTAGGAATC

>A1:93647689-93647989|varpos=93647839

CTTGGAATTCTGTTTTCTCNNNNNNNNNNNNNNNNNNNNNNNNNNNNGCCTGCACTCTCTCTCAAAATAAATAAACTTAAAAAAGGAAAATACTGTAGAGATTGGCTACCTCTATANNNNNNCTCCTTAGCACATTTTCCTGCTATTAAAA[ATTTTTTTTTTT]TTTTTTAAATGTTTATTTTTGAGAGAGAGACAGAGTGCAAGCAGGGGAGGGGNNNNNNNNNNNNNNNGGAGACATAGAATCTGAAGCAGGTTCCAGGCTCTGAGCTGTCAGCACAGAGCTCGATGCAGAGCTTAAACCCATGAACTTCGA

>A1:94060140-94060440|varpos=94060290

GGCAAAAGTTAAACAGAGAATCTTAAAAGTAATGAGAGAAAAGCAACTAGTTATGTACAAGGGAAACTCCATAAAGCTATCAGCTGAGTTTTTANCAGACTCTGCAGGTGAGAAGGGAGTGGCATGTGATCTATTAAAATTGCTGAAGG[GAAAAAAAAAAAA]CCTACAATCAAGAAAATTGTACACAGCTAGTTTATTATTCAGAATTGAAGGAGACATAAAGAGTTTCCCAGACAAACAAAAGCTAAAGGAGTTCATTATCACTCAATCATCCTTAAAAGAAATGTTAAAGGGACTTCTTTAAGT

>A1:95917463-95917763|varpos=95917613

ATCACCAAGTCCAACCACACAGTGCTTGGCAAAACAGAACAGAAGTTTGAAATAAATCGTGAACATTAAAACAGAAAAACAATTCAGAGCAAAATGGAAATTAAAATCATAAAAATCATGGTAAAAGGAACATTAAGCTACATTAAAAN[AAATTTTTTTTT]TTTTTTTCTGCATAACAACCCGTTGTCATTGCCCCATCAAATACATAATCAACTGTCAAAAACCAAACTCGAATTTGACAGGGCAAGGCAAGGAGTTAATAGCACAAGAACAGTGATCATGTTAAACACCCATGCAATCACCTCTGCCAC

>A1:96279871-96280171|varpos=96280021

GATCATGACCTGAGCCAAAATCAAGAGTCGAACANGTAACTACTGAGCCGNTTAGGTGCCCTAATACTTTTCTTAACTTGCAACACAGATGGGTGAAGTATTTTACATTTTTCTTTCTTTAAATGTGACAGAACTGGGTTNCTTTTTTT[TTTTTTCCTTTT]TTAACTAACGTATATATTTATATGTTGTCTTCCTGCTCTCGTCCACTCATTCGTGTGGCTGTAGTTGAGGAATTACTGAAAAAAGAAACCAGGTGAGTAAGAGTTTGAGATGCCTGTGCAGCAGGATGGATTCTGGATGATAGNCTTT

>A1:96414335-96414635|varpos=96414485

TACAGTAGATTTTAATGCTCCCAGCAAAAGATTTTTGGCATTTTATGTCCTACTGTTTGTCACTCTTCTGTACTGACTCGGCTATAAATTTCAATTATAGCTCCATTTCCAGTTAATAGAGACTTGATACATGTAAAGTTTTTGTNNNN[CAAAACAAAAAAAAAAAA]NNNAAAAAGCAGAAGTAAGAATACATAACCCATGTGGTGAAGGACAAAATGATACCAAAAGAAATACCCTGATTTGTGGAAGGCTCGCTGAATGTAAAATATGTGGCTCATGCNCAATTGACCATGGCATTTCTGGTTANCTGGCCTCA

>A1:96549254-96549554|varpos=96549404

ACAATAAAGTAAGCTAGCTGAGAGAAAACATTAAGGAAGAGGACACCATAAGGAAGAAGAAAAACATTTANGGTACTTGANGGTTTTTATCAAAAACAAATCCACAGGTGAGCAGACCAGGCCAGTTCAAACCTGTGTTGTTCAAGGAT[CAAGTGTGTATGTGTGTGTGTGTGTGTGTGTGTGTGTGTGTGTGTGTGTGTGTGT]NNNNNNNNGTGTGTGTGTGTGTGTGTGTGTGTGTGTGTGTGTGTGTAATTGTGTATGTGCGACACACATAGGTATCTATAAATGTATATCTGTATATATACACATTCATACTTGTATATATTCAAACACATATCTCTGATAGCT

>A1:96688702-96689002|varpos=96688852

GCCCTCAAAATCCAGTGAAGTATATTTTGCTTACTCAGTTTCCCAAATCAGTGATAAATAGAAATTGAGCAAATCAAGAGCTTTGAAGGATTGAAACATCAGTTTCTTGAGAAGAGAGAGNNNNNNNNNNCCTATTCGGGCTCAAAAGA[AACACACACACACACACACACACACACACACACACACACACA]TCATGGTNTAAAACTGGTGGAGAATGAAGAGATTTAANAATGAGAGAACCAGAAATTATTTTAAACATATTTTTTATTGTGTTGATTTGTGACCTACTTTTTTTTNNNNNNNAGAACTGCTCATTGAGAAGATTGCT

>A1:96791680-96791980|varpos=96791830

CAGGNTCACAGGTACATACANCAATAATTAAGTATTAANGCATGAATCCTTGACTGTATCTTTGTTTCCAGGTGTCCCCTAAAGANACATGATTGTCTCAAAGGTGGTTGTGTTCNTACAGGGAAGGGTTCTGTTCCTGGCATANAANNNNNNN[TTTTATTAT]NATAGTTTTTCTTCCTCTTAAAAATATTTATTTATTTAAATTNAAATTAATTTACAGCATAATATTCGTTTCAGGTTTAGAATTTAGTNATTCATCACTTANATACAACACCCAGTGCTCATCCCAACAAGTGCCTTCCTTGATGTCCC

>A1:97522537-97522837|varpos=97522687

AATTCTTGATTTTGNNNNAGGTCATGATCTCCAGGTCATAAGATCNAGTCCTATGTTGGGCTCCATGCTGAGTGTGGAGCCTGCTTGGGATTCTCTCTCTCCCTCTNNNNNNNNAANAAAATTAGCCTCTCTAAATAAATAAATAAANN[TAAAAAAAAAAAA]GTGACATCCTATCACTTTGGCCATATCCTGTTTCTCAGAAGCCAGTCACCAAGTCCAGCCCACACTTTAGCTGAGAGGATTACAGANNGCNTGCATATCTGGAAGTAGGNACTGTTGGGAGGCATCTTAGAAGTCTGATTACC

>A1:97915414-97915714|varpos=97915564

GGGTTCAAGTCCCGTCCGTGTAGGGTTCTGGGCTGACAGCTCAGAGCCTGGAGCNTGCTTCAGATTCTGTGTCTCCTTTTCTCTCTGCCCNTTCCCAGCTCATGTTCTGTCTCTCTCTGTCTCTCAAAAATAAGTAAANGTTAAAAAAA[AAAAATTTTTTTT]TTTTTTTAAAGAAAAAAAGAAAGAAGAAGAAGCATTCAAGGTTGCCCACAGCAGAAAACCAGACTTCATGTGTCTGTAGACTACCTTCCCTTCAATCGCTGCTCACCTAGGAGGACACAAACCCAGGACCTCATCATCTCTTGCCTAGATAATTC

>A1:98132506-98132806|varpos=98132656

GGGGAGGTAGCTGAACCACCTATTGGCTTTGCTCTGAACCAANAGTGTGAGATTTAGTCATTATTCTGGGACAACTGGAAGCCTGGTGGTTGTATCTGTAATACCATTATTAACATAACATAACATAACATAACATAACATAACNTAAC[ATAACATAACATAACATAACGTAA]AAATAATATATGACTTGTATGGTGATGGCAGAGGTATACATAAAGAANATTTTCATAATACAGAGAAGGGACACTTCACCAAGACCAAGAAAGNAAGGTTCAGAACAATTTGCTACAGAAGAGATGGTCTCCCCTACATCATGATTG

>A1:98288410-98288710|varpos=98288560

GGTTAAGCGTCAGACTTCAGCTCAGGTCATGATCTCAANGTTGTGGNTTGGAGCCCCTCATCAGGCTCTGTGCTGATAGCTCAGAGCCTGGAGTCTGCTTTGGATTTTGTGTCTCCCTCACTCTCTGCCCCTCCCCCGCTAGTGCTCGC[ACTCTCTCTCTCTCTCTCTCTC]NNTCTCTCTCTCTCTCAAAAATAAACATTAAAAAAAANTTTTTNAATAGAACAAAGGAAAGGATAGAAAAGAGATGGTTAAATTGTTAAGAGTCTTCATCAGTTTACAAGTATCTTAGTATAGTTACNNNNNNNNGGGCAGTCTTATCC

>A1:99276791-99277091|varpos=99276941

TGCAAAAATTTTATGGAGGCAAAATTGACATAACATTATATTCNTTTCAGGTGTAACAGCATAATGATTTCATATTTGTACACAATGGGAAATGACTGCCACAATAAATTTAGTTAAGATCCATAACCATACATAGTAACANGNTTTTT[TTTGTTGTTGTTGTT]TTGTTGTNNNTATGGTNAGAACTTCCAAGATCCACTATCCTAGCTACTTTGAAATATGCAATACAGTATTCTTAACTATAGNTGCCATGAGGCACTTAATATTACATCACCATGACTTACTCATTTAGCAAGAGCTACTTTGAAGGCAAA

>A1:100053961-100054261|varpos=100054111

AAAAATAGCCAAGATGGAACTTGAGCCCAAATCAGTGCACATTCCAAATTCTCCATGAAAGCCTTCTGAAAAAGATAGTGTAGTTCCTCCTGAACTCCAGAATATACCTGCTGACCCCAAAATGAAAACATTAAGTGAAATTAAAAAAAA[AAAAC]CAGCCTCATAGCCCTCATGGTAGTAGGGAATGATGCAGAGTAATTATATGAAGAAGAAAAGTTTTACAGTCCTCCATGTCTGAGGATGGTGATATGTTAGTAGTAATGGCTTCTTCATTGGGAAAGTNATTTTTCGTTATAAAAGAAGAG

>A1:100178315-100178615|varpos=100178465

CTCAGTCAGTTAAGTGTCCAACTCTTGATTTCGGCTTAGGTCATGATNTCATGGTTCATGAGATCGAGCCCTGAGTCGGTTTCTGTGTCGGTTTCTAACAGCATGGAGCCTGCTTAGGATTCTCTCTCTGCCTCTCTCTGCCCCTACCC[TTCTCTCTCTCTCTCTCTCTCTCTCTC]AAAATAAATAAATAAACTGAAATAAAAACAAAAAGAAGCTAATCCATCAGTTAACTAAACCTGCGTGTAAATATATGTTCTTGTCCATTACATTACCCTGCTATCCCTTTGGTAATGTAATCTTACCNGTCTCAAG

>A1:101043987-101044287|varpos=101044137

NANANTACTCATTTAAACCATCTACTNNTCTTGATGATATAGCACCTTCAGCACANAATCAAAGCTTTNTATGTAATGGTTCTTAAAGCAGTGGGAGTCTCTTAAAGCACACCCAGTTAGTTACAGTTNNNACATTGTTGTAAACATGG[GGGTTT]TTTTTTTGCCTTCTACCTGTTTTATTTTTGTACCTTTCTGCTGTTTCATGGATCATAGTAATTTCTTCCAGAAGGTAGGTGATAAAAAGAGAAAAACTGAAATTCATAAAGTCTTTGGTAGTAGTGTCTCCTACAGGTCATCTAATGGCC

>A1:103408965-103409265|varpos=103409115

AGCAACAGAGGGAGACACCCTTTCACCTCTCTTACGGAGGAGGGATTATACTCCANGTGCTCATGCTAAATCTTCGCAAGATTATGAAANGCCAAGTATTTACATACGGGGAANAGGAAATTTGGGAAAAGAAACTTTAAAAAAAATNT[GTTTTTTTTTT]TTTTTTTAATCTTTATTTCTTTTTGAGAGAGAGACAGANTGCGAGCAGGGGAGGAACAGAGAGAGAGGGAGACACAGAATCCNAAGCCGGCTCCAGGCTCCTGGCTGTCAGCACAGNGCCCGACGCGGGGCTNGAANTCACAAANCGCGA

>A1:103655080-103655380|varpos=103655230

CTCAGGTAATGATCTCATGGTTTGGGGAATTTGNGCCCTGCATCAGGCTCCAGGCTGGCAGCACAGGGCCTACCNGGGATTCTCTCTCACTCCTCTCTTTGCCTCCCCCCTGCTTGCTCACTCTCTCAAAATAAATAAACTTAAAAAAA[AAAAAGAGAGAGAGAGAG]GAGAGAGNGAAAGAAAAACCTTTCAAAATCTCCTATCAGGAGCAGATCGATAGTTCAAGAAAATTTTGTCCTTTTAGCAGATNAAAGTAAATGTAGGGTTTTTTTNNNNNGTTTTTTTAAGTTCATTTATTTTGAGAGACATAGAGGGA

>A1:106562862-106563162|varpos=106563012

CAAAGAAGGTCCCATTGAAGAGTTTTTGGTTAACTTCAGGCCTGAATGATGAGTCAGAGTCAGCCTGCTGCACAGAAGTAGGGAAAAGTATTCCTTACAAAGGGAACAGCACTTATAAAGATGCTGANGAGGAAAANNNGAACTTAATGTGAT[TAAAAAAAAAAAAAA]TTAGAGAAAGGAAGCCAGAGTGACAAAGCACAAGGCAGGANCTAGGCTGATGGAAGATGAAGCTCGAGAGGTATGGGGAANATGCTGTANGGTCCTGTAAACCACGGGAAGGAAGCCAGAGTNTGTCCTAAATTCAATGAT

>A1:107526997-107527297|varpos=107527147

NTGGNNNNNTCAAAAGTATACAATNTAAGTGGAAGAGAAAACAATGGAAGACCACCAGAGTATTGTAAATGGTTTTTACTGTNNNNNNNNCATAGGTTTTTATAGTACTTTACAGTTTATAAACTGTATTTACAAANNNGTTTTTTTTT[TTTTAAA]ATTTATTTTGAGAGAAAGAGAGAGCATGCATNCGTGCAAGCAGGGGAGGTGCAAAGGGAGAGAGAATCCCAAGCACGCTCCAGTGAGGAGCCCAATGTGGGACTCANTCTCACTAACTGAACNCTGAGATCACAACCTGAACCAAAATCA

>A1:111972459-111972759|varpos=111972609

TGCAGTGATTAAAGTACAATGCAGGCCTCAAATGAGTGGTTTACTTCTTTGAGTCTCAGTTTTNNNCTCTGTCTGTAAAATGGGGGTAATATGAATTAGGTGAGGTTAAGCGTGTGTGTGTGTGTGTGTGTGTGTGTGTGTNNGTNTGTGTNN[GTATGTATGTGTATGTATATAT]CACGTACAAAAAGCATTTAGCATGCACCTTGTTTTAACAGGACAATGACTTTTCAACATAAGCTATTGTTTCTAAAGAGATGAAGTGCTCAGAAAAAGCAATTGTGATTTAAAATGAGGTATTTGGAGGGTGCCTGGGTGGCTCAGT

>A1:112203072-112203372|varpos=112203222

ATGATCTNGCAGTTCATAGGTTNGAGCCCCGTGTTGGNCTCTGTGCTGACAGGGTGGAACCTGCTTGGGATTCTGTCTCTCTCTGCCCCTCCCCTGCTCTCTCAAGCTCTCTCTGTCTCTGTCTCTCTCTTAAAATAAATAAATAAACT[TAAAAAAAAAAA]TCAGTAATAGCATTTTTTGAGTATTACTAAGTGCTGTGCACNGTGTCAAGTGCTTTACAGGGATTGTTTCATTTACTTGTGCACAGTAGCCAGACAAGGTAGATACTCAAGAATATTCCCATTTTATNATTGAGGAGACCCATTCC

>A1:113828871-113829171|varpos=113829021

CAGTTGGTTAANTGTCGGACTTCCGCTCAGGTCATGATCTTGAGGTTCATGGGTTCGAGCCCCNCATCTGGCCATGGGCCAACAGCTCACAGCCTGGAGCCTGCTTCAGATTCTGTGTCTCCCTGTCTCTATGCCCTTCCCCTGCTTGT[GCGCTCTCTCTCTCTCTCTC]NCTCTCTCTCTCTCTCTCAAAAATGAATAAACATTNNAAAAANTTAAAAAAAAAAGAGTAGTGTTNAGAGGAAGTTTGAAAATACCTTGGTCCTCAGAATCCTGCTTTCTTGCCAAGAAACTCAGCCATGTTCAGAGGAGACCCAGCAC

>A1:115246630-115246930|varpos=115246780

TGAGCGTCTGACTTTAGCTCAGGTCACGATCTCACGGTTCATGAGTTTGAGCCCCACATCAGCTTGCTGCCNTCAGGCTGTCAGCACAGAGCCCACTTGCTTTCCTCTGCTCCCCTCACTGGTTTGTGCTCTCCAAGAAAACAAAACAA[CAACAAAAAAAAAA]NAAAAAATGACAAGACTGGGGGCACCTGGGTGGCTCAGTCAGTTAAGCGTCCAACTTCTGCTCAGGTCATGATCTTGCAGTTTGTGGGTTNGAGCCCCACATCGGGCTCTGTGCTGACAGCTCAGAGCTTGGAGCCTGCTTCTGATAGT

>A1:117149197-117149497|varpos=117149347

TGGTTGAGCATCCGACTTTGGCTCTGTTCATGATTTCACCGTTTGTGAGTTCGAGCCCTGTGTCAGGCTCTGGGCGGACAGCTCAGACCCTGGAGANTGCTTCAGATTCTGTCTCCCTCTCTCTCTGCCCTTCCTCCACTTGCATTCTG[CCTCTCTCTCTCTCTCTCTCTCTCTCTCTCTC]NNNNNAAAAATAAATAATCATTAAAAATTAATTTTAAAAAGGCTCAAAATAAACTTTATTGTCATTGGCAAAAACAGTACCATTGTAANTGGAAGTTTCATGTTTTGTTGCTCAGTGTAGTTGCTGGATAGAGATGANCTAT

>A1:117547128-117547428|varpos=117547278

CAAACTTTTACTTGGATTTTTATTTACTGTGTAAAAAATAAGGGATTTATTAACACAATGATAATATGTACATAAACATCTGGTCTACTAACATGATATATCTTTCTATTTATTTTTTTCTCTCNNNNACTGTTTATACGTGTTTATGTGTGTAT[ATAGAGATATTTTTTTTAGAGATATTTTT]ATTTTNGTACAACTTCATTAAATATTTGATATTTTGATANAGTTTTAAANGGCATTTAAAAAATTTCATTTTATAATTGTTTTTGTTGTAGGTATATAGATATAAAACTAATTTTTGTATTATGTTTATATCCAGGGTCCTTG

>A1:119155332-119155632|varpos=119155482

TGAAATGTTAAGAGGGAGAAGGATGAGTCATGTCTTTTAATGTGACACCTCAAGCAGTCCTATTACTGAGCTCCCCAAATGTGCTANCATTGGGGTTAGGCAGTGGTGGGTGGAGACCTTGGAGGTCCTTTCTTAGGATTTTTTTTTTT[TTTAAA]AAGCAGCTTTGTTGAGATAAAATTCACACACTACACAATTCACTTNNNNNNGTGTANAGTNNATGGCTTTTAGTATATTCACAGAATTTTTCATCCATCTCCACGATTAATTTTAGAACATTNCACCATCCCCCAAAGACAGCCAGCCGT

>A1:119684321-119684621|varpos=119684471

TTTTTCTTACAATTTTTAAAATGAATTTTAATTTNGATAAAATTCCCTTCTTCATATAATTTACAAGNAAATAAAAATCTTCAAGTATCGTTTAGAAACCTTCTCCAACCAGTAATCTGACCCCACAAGACTGTTCTAATCTTAATAAA[GAAAAAAAAAAAAA]CCCTAAACACAGGCATTTGAGTTGTGAATACAAAAGAATACTTAAGAAGACCCTTAGGTCTCGACCAGTTTTTCAATAAAAAATTGTACNGAATTCGGCAGCCTCCAGTCCTCATGCAGGGGTGACAGTTGGCTCAGGTGGT

>A1:119778021-119778321|varpos=119778171

GTCTATATAAAAAGTTANTCATTACAGCAGTTTGTAACAGCAAAAGATTAGAAATAACCTATAGTGCATCAATCGCGNGACATTTTATGACTGATTTCTAAGTAGTTATTTGCAAAGAGGTTCAAGACGTGTTGTTAGCTGAAAAAAAA[AACCCC]CCCACAAAATGCACAAGTGTTATAGTANCTACCNTTTATGTAACACAGAGGGAGAAATAAGAATATATATGATATTTTCTTAGAGAAATATGAGAATTTTTTCCCCTCTCTTATTTTGGTAGTGGTGAGGGCNGTGGGAANCAGGTAGAA

>A1:120209781-120210081|varpos=120209931

TGAGCATCCNACTNTTGATTTTGGCTCACNTCATGATCCCATGGTCGTGANATCAAACCCCACATNGGGCTCCACCCTGAATGTGGAGACTGCTTGAGATTCTCTCTTCCTCTCTNCCTGCCTCTCCCCCACTGNCTTTCTNNNNNNNN[AAAATTTTTTTT]TTTTTTAAGTCAGANAATGAAATAGAACACAAATACATTAATGTATTAGAAGGCATTTGAAAGGGATNGAACACAGTAAATAAGAGGTGATGAGGATGTGACAGCTCAGGCTGTAGTCACAGGAAGAAATATCCTTTGGGGTTTTTGCAC

>A1:120474940-120475240|varpos=120475090

AGATTTTAACTCTCTTGTGAGAAATGTAATATATAAAAGCTTCACACATTAATTTTTTNNNNNAATTCAGTCTGTCTTGGTTGAATCACTCATATTTCCCTTGTATTTCTTCTCTATCCTGGAGATCACAAGTTAATTGGGTGACCTGCTGAGCC[ATTTTTTTTTTT]AAGTTTATTTATTTTGAGAGAAAGAGAGGAAGAGAGAGCTGGTGCACATGTGTGCAGAGGAAGGACAGAGACAGAGACAGAGAGAATCCCAAGGAGGCTCAACGCTTAGCGTAGATCCTGACATGGGGCTCAATCTCACTGCT

>A1:121161407-121161707|varpos=121161557

AGATGATGGACCCATATTTTTTCATTTTGATTGTATTAGTTTTAAAAGAGAAATTTGTAAAAGATTTATTCTAGGGAAATCAAGAGGAAAACTGAAGCAGTTAGTAGTATCAGAATTTTCTGTAAATGGTATTTGAAATCATTTTTNGG[GTTTTTTTTTTTTTT]GTTTATAAATTAATTGGATATCAGTGAGTTCAATTGCTGTGTTATATCAAATTTAAAAANTAGGACAAATAAAATCAGTCACTCAGATTTCTCAAACTAGTATTTTGGTCAGTTATTCTCTTGATAGCAACATGTGGTCATTT

>A1:121273808-121274108|varpos=121273958

CNCTGAAGGATTAGAATCAGAGACATCATTTACAGTGGGGCAAGATGGNAGACAGAAAGATAAAGATTATGATAAAGATAAAGACAGAAAGNTAAAGATTATGGCATAAACATAAGTGACGAACAGAGAGATTCTAAAATTAAAAAAAA[AAAACAGCA]CAATGGAGAAAGGGATAANAGAATACATTTTAGAGATGTTTTGATAAAAATTATGTTGACTTACTGGAGGTGAGGAACTNNNTAAAAGAGAGANCATCCAGNATAAGCCCTGAGACNGAATTTGAACATTACTGCATATACTGTTCTATA

>A1:125065000-125065300|varpos=125065150

ACTTTGTTCAGTTACCTTTTAATATTCTGTGACCTCCTTATTGTGACCCTGATTGATACATGCCAACCTATATTGCTTGATTCCATTTGATTCCTNAGTGTCTCAGATTCAATAATCTCCAAAGATGGAAACAAATTTATAGATAAGTA[TTCTCTCTCTCTCTCTCTCTCTCTCTCTCTCTCTC]NCCTTTGTGAGANCAAACNATCTTGCTTTCAGGGCCATCATACATTGCCTTGGTGGTAATTCAGACTGGACTAGAGAGCAGGTGTTTGGTTTCAAGCCTGGGGCTTCCATNCTGTTC

>A1:125545964-125546264|varpos=125546114

GTTTTCCAGAGTGGCTGCACCAGTTTGCATTCCCACCAACAGTGCAAGAGGGTTCCCNTTTCTCCACATCCNNNNCAGCATCTATAGTCTCCAGATTTGCTCATTTTAGCCACTCTGACTGGCNTCAGGTGGTATCTCNNTGTGTGTGT[GTGTTTTTTTTTTT]TTTTTTTTAACTCCTCACAGGCCCCACTAACACTTAGAGAATAAGAACTAGGTCTTCACTCNNNNNNNNNNNNNNNNNNNNNNNNATACTGGGGAGCATTAACTCCACTACACACCCAGTCAGCCTTGCCTTGGAAAAAAAANNNNNTAAAAACCCGA

>A1:126950682-126950982|varpos=126950832

CTGATGAAGAATCCTTCTGGTCACAGTTTATACTGTTTCAAGCATTATATNACTTTTATGAAAGACCTAAACTGGCATTATGCATACTGTCATGCAACTTTTTTCACTTATTATTATTTATATTAGATGGTCTTTTTCTTTTCTTTTTT[TTTAAA]AAGGTTTATATTTATTTTTGAGAGAGAGAGAGCACAAGTGGGGAAGGGGCAAAGGGAGAGGGTGACCAAAGATCCAAAGTGGGCTCTGTGCTGACAGCAGAGAGCCCAATGCAGGGCTCAAACTCACAAATGGTGAGATCATCACCTGAG

>A1:127478260-127478560|varpos=127478410

ATGAATACAAGATAAAACCTCAGAAAAAGAGTAAAATGAAATGGAAATAACCCACCAGATGAAAATTTCAAAAAAGGTCATAAAGATGTTNACTGAACTCAGGAGAATAGATGAACACAGTGAGAACCTTCAAAGATGTAGAAAAGATT[TAAAAAAAAAAAAAA]AAAAAAACAATCAGAGTTGAAGAATAAATGTAGTAAGTGAAACAAAAAANNNNNTATACCAGATGGAATTAACATCAGACTAGATNATGCAGNAGAATAGATCAGTGACCTAAAACACAGGATAGTGGAATTACCAAATCAGGACAGTAATAGCAA

>A1:127481125-127481425|varpos=127481275

ATGTTCCAAATACATAAAACAATCAACATGATACACCACATAAACTGAAGGGATAAAAATCAATGATCACCTCAATACATGCAGAAAAAGCATTTCTTGAAATTCAANGCATTTTATAAAATTCAACAACCATTCTTGATTNNNNNCAA[AAAAAAACAA]CTTTCAACCAAGTGGGTATAGTAGGAACATAATTCAAAATAATAAAGGCCATGTATGACAAACCCACAATTAACATACTCAATGGAGAAAAATTGAAAACACTTCCCCTAAGATGAGGAAAACAATGAGGCCCACTATTACTACTCCT

>A1:128640680-128640980|varpos=128640830

AGAGGAGAAAACTGAGGTACAGTGAGGTATAATNNGACTTTCCAGAAATCACAAACATCATTAGTGGCAGAATCTATGTCCTTGAGAAAGNGGCCTATTCCCAACTTGCTGCATCCACCTAGCACTTACCACCATNTCCTAAAAAAAAAAAAAA[AACAAAAACAAAAACAC]ACCAAACTGCCTCAGTCTCCTACTTCTATCAAGTAAAAACTTATATGATGCGCTNTAGAAGTTTAGAGGGAAAAAGTGAGGAGGGACATATTTTTAAACTAGAAACNTGTGTTAGGACTAAAGAAATTAGAATATATACTCAACAAAACA

>A1:130024365-130024665|varpos=130024515

AGGAAACCCANTGAACAAAGANACAGACAGACACATGCACAAACATGAGACTTTGTCAAGAAGGTACGCATGATAGGAATGGGCATTTAGAGAATGCTTTGCCATGTGGCAGATGTGAATGAGTGAATGAGTNNNTGTGTGTGTGTGTG[TGTGCACGCGCGCGC]CATATATACACCTTATTTTAAAGATGTATGTATCTTTACTTGTTATAAAGTTCAAACTATCAGTAAATTAGAGTGATTTGGACACAAGTCTGACTCCTAAGGCTGCACTAATTGTATCCATCAATGTATACCAGGCTTTCAAGTGTCTA

>A1:130880318-130880618|varpos=130880468

TGGTTTGATGTATGCAAGAACTCTGTTTAGAGGTTTGTTACAGTATCTAAGAAAAAATAGTTATAAAAATANCTTAATAGAATATATATTATGTGTNNNNNNNNNNNNNNNNNNNNNNNTATGTGTNGCATATATATGTGTANNNNNNN[ATATGTGTGTGTGTGTGTGTGTGTGTGTGTGT]NTGTGTGTGTGTGTGTGTGTGTGTATACAGTTGAGACTGATCAAATTAAATGTTCCTAAGAATAAAAAGCATGGCTTTCCCCTAGGAANTAAAAACTACCTTATTATCAAAATGTAATGTCTGGGTATTATGAATATTTATTCTATTAC

>A1:132978613-132978913|varpos=132978763

GGAGGTATAANTAATAAGCCAATAGTGAAGATAAAATGAATTAAGAGAGAAAAATGAAACAAAAACAAATAGAAAAACTAGGCAACAAGCAGCTAGATTAAAGACCAAAANCCNCTAATTACATTAAATGTAAATGAACAAAAAAAANN[AAAT]TAAAAAATGGAAATTGTCAAACTGGATAAAAAAAGCAAAACCCAACTANAAGTTGTCCAAAAGAAATCCACTTTAAATATAAAGACATGGGGTAGGGGCACCTGGGTGACTCAGTCATTTAAATGTCCAACTTCAGCTTAGGTCATGATC

>A1:134999321-134999621|varpos=134999471

TAGTCACAACATCAAATGCACAGTCCATGAAAGAAAAANNNNNNNTGGGACTCCATTAAAATTCACAAGTGTTGCTCTGTAAAGACATTGTTAATTAAAGAATGAAAAGATAAGCCACAAGAAATAAATAAAATAGAAATTAAAAAAAA[AAAAAACC]CAATAGAACAGATTAATGAAACCAGGAGCTGGTTCTTTGAAAAGAGCAACAAAACTGATAAACTTTTANCCAGACTCATNNNNNNNNNNNNNNNNNNNNNNTCNGATAAACAAAATCAGAAATGAAAGAGAAAAANCAGCCAATACCACA

>A1:135622688-135622988|varpos=135622838

TGGCCAAACCTAGACTCAAGGGTGGAGATACCTCTTGATGGAAAAAACTNNNNNNNNTATTACAATAGGTCATGCTGACANAGATAGGAAAAAATTGTGGNCATTTTTGNAATCTATTACATAGCCTACGCAGTGCTCAGAAAAAAAAA[AAATT]TGTATCTTTAAATACATTAGCAAAGAAGAATGATTTGAGTTTTTATCTCAAGAAGCTAGAAAANGAATAGCAAATTAAACCCAAAGAACATAGAAAGAAGGCAATAAAAAGTAAAAATCAATGGTACAGAAAACAAANNCATANAATAGGGAA

>A1:135729860-135730160|varpos=135730010

AACTCATACATGCAGATGTCAAGTAGGCAGATGGGTACATGGGTCTGAAGCTTAGTAGAAAGTTCTTGGCAANGGTTCAGTTTGGGGAGTTATCAGTGCACAGGATATGGATAAGATCATCTGAGAAGTGTGTCAAGTAAAAAAAAAAA[AAAATTT]TTAACAGATGAGTAGAAGNAGAGGGAGAATCTTCAGAAGAGAACACAAAANCTCTGGTCAGCCAGTTAAAAGGAAACCCGGTGGGAAAGGTGTTGTAAAAGTTTGGAATGTTTCCCCGAAGATGAGTATAGAATGTCTAACTCTGTGTTA

>A1:137838077-137838377|varpos=137838227

CACAGTTCATGGGTTCAAGCCCCACATTGGGCTCTGAGCTGATAGTTCAGATCCTGGAGCCTGCTTCAGATTCTGTGTCTCCCTCTCTCTCTCTGCCCCTCCCCCATCACACTCTGTCTCTCTCCAAAAATAAATAAACATTAAAAAAA[AAAATTTTATT]TTAAAGTGTGGAGTTAAATAGTCTGAAGCAGATATGGTGACTCNAAAATGTCCTGAAGGACCTAGGCTCCTTCCAGCTTCTAGGGTATGATCTCNGTCCTCATACTCATAAGGTGAATACTGGAGTTCCAGCCATCACTTCAGTGCTCC

>A1:139387846-139388146|varpos=139387996

GAGACTGGAAAATCCCAAACATCTATTCCAGACCATCTTTAAGACTCATGATTTTTACATTTGGCCATAATAAATATGAGAAGAGTTGAAAAAAACTTACAAACCATTTCTCAGTCCAAGTGTACTTANTGTGTTACCAGAGATTTTGC[GTTTTTTTTTTTTTTT]TTTTTTTTAAAGTTTATTCATTTTGAGAGAGTGTGAGGNNNNNAGGAGCAGGGAGGGAGAGAGAANCCCAAGCAGGCTCTGTGTTGTCAGTGCAGAGCTGNACACGGGGCTCGATTTCACAACTGTGAGGTCATGACCTGAGCCAATACC

>A1:140401680-140401980|varpos=140401830

TGAGCCAGCTGGGCACCCCTCACATATACTATTTTTTATCAGAAAAGCTGAATGACTTTTACAAGACAACATATTCAATAAATGAGTTTGATAAAAATTTCTGTTTNAGGCTGTGATTTACATAAAAGCAATCAAAGGAAAAGAAGTGG[TGGGGGGGGGGG]GGGGGCGCATCCAAAATTGAGATAGTACTGTCCTATTTAATGAATTTTTTCTTCCATTTTCTCTTTTGTTATTGAGAATATTACTTACTACCCAAGAATATTAATATTAGCTAATATTAACATTTGTTAGGAAAGGGCCAGCNGAATG

>A1:140699162-140699462|varpos=140699312

TGGGGCTCGCTGTCCACCNGAANNNNNNTTTGGCCTCAGAGACNCTAGTCTCACAGACTGAACTCATTTATTATAGTCCTTGGCCAGAACATTATGGAGGAAGAAAGTGGAGCAGATTGATTCTTCTCCTCTTGAAATAAATNNNNNNN[AAATTTTTTTTTTTT]TTTTTTTTACTGTGGTAAAATACACATAACATAAAATTTCTCTTTTAACCATTTTTAAGTTCAGTGGTATCATTNTTNGGCAACCATCACTACCACCCGTCTCTAGAGCTCTTTTCATGTTGNAAAAATTTAAATTTTATACCCATTAAG

>A1:143086513-143086813|varpos=143086663

TTAACTTCACCAATATTTACCGAATGCCTACTATACCCTACCCTAGACAATGTGCTCCTGAGTCAGGCTTCTTAGGGGGAACAAAACTCCATAAAACAAAAAATTTAACAACCTTATGTATGGCCCTAAACATTAAAGAAAAACAAAAA[CAAACAAAAAA]AANAAAGTGTGGTCCATTAAATCCAAAAGTCTGGCNACCACNGATTTAAAAAAATTTTATGGCACCTTTACTCTCCCNATGCCTAAGCAACCATCTGGTTTCAGATGAGCATCTCTGGAAGAGGCCACCAGGGACCAGGCTGTGAAT

>A1:143135957-143136257|varpos=143136107

AGCTCCTTGTTATGAAAGATCATGGACTTTGGATTCCTAGGGACTGTGTATAAATACCACTTCCTTCTCCATTGGTTAAAAAAGGATCATGAGCAAGTTACTTTACTTTCANGAACTTTACTTTTCTTGCCTTTTAAATATNNNNNNNN[AATTTTTTTTTTT]TTTTTTTGTTTTAAGTAGGCCCCACACCCAATATGGGGCTTGAACTCATGATCCTGAGATCAAGAGTCACATGCTCCACCCACTGAGCCAGCAAAGCACCCCTATTTTTCTTGTTTTTTNNNNNAAGTGAGGATAATGCTACCTATTGCATGGAAT

>A1:143546788-143547088|varpos=143546938

GGAAAGAAATTTCNGGAATTTTCTTGCTAAGATAATTAGTAATCCATCCTTATCTTCCAGTGCATAGAAATAAAGCCAAAGGAATATGGCTTTCAGAGGCTGCATGTGGTTGAGGTGAAATAAAAGGAGAATGAATGTCATTCAAAAAA[AAAATAATAAAA]AAAAGTTGCTATCTACCATTTCTCACAAATCAGGTTNGGTATCTCATACTTCATTCATANTTGATAGAAACTTCTATTCAACTTACTGTTCTATTATAGTATAAGATAGATGACCCTTTAGTACTCTTTTCAGAACTTTATTGCCACACA

>A1:144406571-144406871|varpos=144406721

TGATACAAACCTACCTCAACAAACAAGGAAAATCTCAATCTAACTTTATACCTAAAGANACTAGAAAAAGAAGAACAAAGCCCAAAATTAGTAGAATGAAGGAAATAATAAAGATCAGAACAGAAACAAATAAAATAGAGACAAAAAAA[AAAAAGAGAGAGA]NGAGAGAGAGAAAAGATTAATGAAATCAAGAGTTGGTTCTTTGAAAAGATAAATAAAATTAATATCCTTTAGCCAGGCGCATTAAGAAAAAAAGAGAAAGGACCCAAATAAACGAAATCAGAAATTCAAGAGGAGGGGTGCCTAGCTGGC

>A1:145097380-145097680|varpos=145097530

CTCACTGCCACACACTTNATACTACTCTTAAAACCACCTTTCTCTCAAAACCAAAATGGTAGCAGTTTTCATCCTGGGAGCACCCATACATCCTTGTTANCTGCTTCAGTTATCCTCAGTTCAGTTTCTTTAAAGAGGAGCNTTCTNNN[TTTTGTTGTTGTTGTT]NNGTTGTTGTTGTTGTTCTTTTGTTTTAATTTTTGTTTGGGCCTATTGATAAACTTCTTGAGCTCTGTGCCCTGGGATGGGAAAAGCAGAGAGTGGTTGTTTCATAACAACCTCCCTGCCCAAAACTTAANCCAGCATAAAACATCATTTAT

>A1:145587391-145587691|varpos=145587541

NGGNCCGTGAGTTCGAGCCCCGCGTCGGGCTCTGGGCTGATGGCTCAGAGCCTGGAGCCTGCTTCCGATTCTGTGTCTCCCTCTCTCTCCGTCCCTCCCCCATTCATGCTCTGTCTCTCTCTGTCTCAAAAATAAATAAACGTTAAAAA[AAAAATT]TAAAAAAAAATAAAGTATTGGCTTTATAGCTAAATAATGGAATTTAATTTTTTTGGATTAAAAAATAAATGNNNATTATAAAGCACTATCTACAAGTCACAAAAAGTTAAGAAAATCTCCATAAACTTCAAAAATCTGTATATTTGGGAA

>A1:145642214-145642514|varpos=145642364

ATCCTTGATTCCTATTCTCTTCCNTTCTCTAANTTTCATTATGTAGAATTAAATCTTCTAAAAAATATTTTATTAAATAGAATTTGTATTTCTTGTTATTTATTTTAAAAGGTGATCCTCCCTCAGAAGATTCTTTAACAACAAAAAAA[AAAAACCC]CAAAAAAACACAACTTCCTCTTTTTAAATAAAAAATTACAAAAATAAAAACCTTAAATTGAATTACCTTATGAATTAACTCTGACTCAACTCATTGATTCTCCAAAACAGAAAGACAGCTCTTATATTTTTATTCTTGACAGCACCACTA

>A1:146777615-146777915|varpos=146777765

TTAGAAGAACTGGAATAAAGACAAGGCAAGGAGATGCTCNTCTCTNGTGCTGCCTGCCTCCTGAGNTNGATGCNAGAGGATTCCTTGGAAATGTTTGGCTCCACTGCCTCCTAGACTAGGTGGGTAGAAAAGTCACACTAAGTTTTTAAAT[TAAAAAAAAA]AAAAAANAATTAGTGTTTATTTATTTTTGAGGGGNAGGGGGCACAGAATCTGAAACAGGCTCNAGGCTCTTAGCTGTCAGCACAGAGCCCCACACAGGNCTCGAACCCACANCACATGAGATCATGACCTGAGCTGAAGTCAGACNCTTA

>A1:148098836-148099136|varpos=148098986

ACCTAAGCCAAAGTTGGANGCTTAACCAACTGAGCCAATCAGGCACACCCATTCAAGTTCAATTTTAAGGATGAGTATTTATAGACAAATCTGTATTTTCTTATTGCATCATATCATGAAATATGTGTGTGTGTATGNGATTGTGTGTA[TACACACACACACACACACACACA]GACACACACACAGTTGTTCCACATTTAGGGATACAAATACTAATCAGTGGATTCAGANGGTGACAGCCTTTTATCCCTTATAAAATTTCCACCAATCCTTTACTTATTAGGCTTAGTTTTCACTANAATCACTGCATAGACTGATTT

>A1:149203598-149203898|varpos=149203748

ATAGATGANATGAACAAACAGTCTTGAGGTGAAAATCACTANGAGTTTATCTACNTAAAATGTAATAGAGGGTATACTTTGAGAATAGTGNTAGTGAACTGTGGAATCTCTCACTTTGGGGTCAAGGGCATAGCTGCTTTTTCTTTCTT[CTTTTTTTTTTTTTTTT]NCCTTTCTTAGATACTCTAGATATTTGACTTATATTCAGTGATNGATCCAAAATGAATTGTTTTGGGTTATTTCCTGATAGTAGATATTTATGTCTTAAGAGATATTTATCTGATAAAAAGTAATTAAATTCCTGTTGACCA

>A1:151095986-151096286|varpos=151096136

ATAAACTTGCAAANAATCTCCCTGCACCCACTACAAGTACTGTCTCGGAAGCATTAAGACATCTTCATCTTCCTTCATCTGGACAAGGCCTGGAATATGAGGAAGGTGTGTACAAGGGCATAAGGGAGCTTGAAATGAGGAGACAAGAC[TTTTATTTATTTATTTATTTATTTATTT]TTATTTATTTGTTTGTTNNNNNNNNNNNNNNNNNNNNNNNTTGTGGGCTTCATGCCCAGCACAGAGCCCANTGTGGGGCTTGGACTCACAACCTTGAGGTCAAGACCTAAGCTGAGATCAAAAGCTGGACACTTAACTGACTGAACTAC

>A1:151175171-151175471|varpos=151175321

AACAAACNATTCTAAAAATAAATAAATAAAAATCATTCCAATAACCTGCCTTCACTGCCCAACTTAGTATCTATGTATGCNTAAGTTTTTTCTTTTAATGTTTGTTTATTTTTGAAAGAGAGCCAGACAGAGCACAAGCGGGGAAGGGG[CAAAGAGAGAGAAAGAGA]NAGAGAGANNNAGAGAGAGAGAGANAGAGNGAGACAGAATCCAAAGTAGGCTCCAGGCTCCAATTCATGAACCATGAGANCCTGACCTGAGCCAAAGTTGGACGCTCAACCGACTAAGCCACTCAGGTGTGCCTATGTATGCCTAAGTT

>A1:153792176-153792476|varpos=153792326

AAAAGATAGTCTCTTCAATAAATGGTGTTGGGAAAACTGGATAATCACATGCAGAAGAATGAAATTCGANCTCATNTTACATCAATCACAAAAATTAAGTTANAGTGCATTAAAACCTTAGCTATAAGACCAGAAATTGAAAACTCATA[GAAAAAAAAAAAAAAA]AAAAAAAANAGGGAAAAAGNATCTTTACATTGGTNCTGGAAATGATTTTTTGGATAGGACACCAAAAGTACAAANGACAAAAGCAAAAATCCNAAGAGTGGGACTATATCAAACTAAAAAGCTTCTGCAGAGCAAAAGAAACCATGAAGTGA

>A1:156140981-156141281|varpos=156141131

AATATTGTAATTTATCAGCTGTNTTAGGTAANGCACTATTACTACAAAGTTGAGGAAGACATGATTTCTGACNTTTGGGAAGAACTCCCAATTTAGGAAGGAAGGCATGTTCAGGTAAGTTCTGTAGAGCCCTTTAACATTCTGAGTGT[TTGTGTGTGTGTGTGTGTGTGTGTGTGTGTGTGTGTGTGTGT]NNNATAGAACAATTTCTTTTTAAGTAGATTGGTAGCTGAAGGATTATGTNCCTNNNNNNNNNACATGAGGAAANTGATGTTTAGAGAACATGACTTGCCTGTATCACNTAGTTACTAAGTTGGCACTG

>A1:156802846-156803146|varpos=156802996

AATTGTATGATGTAATATTTTCTTGCTTCCTTAAAGCAGAAAGCATAGTCATGTGAAACAGTCTTTGAAACAGTGGTTAAATGTGGCTNCCTTTCATAATNNCAAAATTCATACCTCTCATGACTTCATAATAGTATCCAAAATTTTCATGGAA[ATGTTGTGTTTT]NTTTTTTTTAANCAATAGTATAGNTTTATTAACTAAAGAGTATTTTTCTTANTAATTCTAAGGTNATCTTTTCATTGCTTAAACACTTTTTAAAATNAACAATTGNGTATAGACTGTGAATAATAAANCCTAAAGAAAGTACAGAATCCT

>A1:157434984-157435284|varpos=157435134

AACTTAACTCCCTCACTTTCTTTGAGACATTTACAAATGTTTTNATCTGAGCCAGGGCAGTGCTGACCACCCTATAGNAGAGCACAACCAATACTCCTATCACCACCCCTGCTAACTACACTCCTGATTCTCTTTATCCTGTTTTTTTT[TTTCC]CTCTTCTTTTTCATAATGCTTCTCACATACAATCTACTTTTTATATTTTTTATGCTTAAGGTTTCTTGTCTGTCTTCTGCCACTAAAAANTAAGCACCATGAGGGAGGGTAACTTTGTTTTATTCACTGATATGTCCCAAGCATCTAGAA

>A1:158263941-158264241|varpos=158264091

AAAGTAAAACAAGANTTATTTTCTTATGTTTTATTTCTTATCCTAGTTTCTTGAAGGTTCTGAGTATGAATAATAAAAACCAAGAATAAGTGGCTTCTTGCTTTTTTAAACATCAGCATTTTCAGCTGTTTAAAAGATTTTGAAAAAAA[AAATTTT]TTAAAGGTTTTGCCTGGGTTTCAAATGGGAGACTCATTTTGGGGACACTATTTGACTTACACTAAGGTTTTTTTGTTTGGTTGTTGTTGTTGCTNTTACATTTGGCATTCCATATTCACTTTTTTTGTGTAGTAAACTGTACTTGATTACTTAA

>A1:158990171-158990471|varpos=158990321

TTGAAGAAGACACACATAAATGGAAAGGTATCTGATGTTCATGGATCAGAAATATTAATATTATTAAAATGTCCATACTACTCAAAGCCATTTGTAGATTCAATGCTATTCCTATAAAAATTTCAATGGTATTTTTCACAGAAATANNNNNN[AAAAATTACT]TACTAAAATNTGTGTGGAACTACAGAAGACATTGAATAACCAGAGCAATCTTGAGAAACAACAAAGCTAGAGATATGACATTTCCAAATTTCAAACTGTACTACAAAGCTATAGTAATCAAAACAGTANGTATACTAATTAAGAGAGTAT

>A1:159088946-159089246|varpos=159089096

GAATGCTTAATTCTCCTTTTCTAGTCATCCCTTCTGGTGAGTCTGTCTCAGGGACAAGGCCAGCTTCATGAACTTGTGATCTGTGCAGTCACACAGGACCCCAAGCTTGATTTAAGTCTCCACCGACTTGAAATTCTTAGCANNNNNNN[TTTTAAAA]AAAGGCCCTGCATTTTTATCTTGTATGGGCCCCAAGAATTATGTAGCCAGTCCTGATCNGTAAAATAAATTTGAAATCAAATGGAGAATCGAGCCCTGTGTTTTCTTCATCTTTANGAAAAAGTGCAAATTGCAGCTTTCATTCTTCAGT

>A1:159690072-159690372|varpos=159690222

AAAATTAAAAAACATAAAAACAATACAAAATATCAAATAAGGAAAACTAGATCCTAGTTGTGTTTTGGTCTGCTTGTTGAAAGAAGCTTGGTAGAATATAGGAAAAAGGAAAAGAAAAGAGGAAAAAAGNTAAGAAAAATATTAAAAAT[TAAAAAAAAAA]AAAAACTATGTAATGGAATAAAGTGAAATAAAGAAAATGATANANAACAAAAAAANTTTTAAANNAAAAATAAAGTAAAAAATAACTAAAAGTTTCCTNTTTCTGTATCCAAGAAAGAGGAAGAACACAAAGAAAGAAAAAAGGAACACA

>A1:160167836-160168136|varpos=160167986

TCATCAGACTTGAATTCACACAAANTGGTCTCTTACTAACTACCTATTAGAGAGGAAAAGAGGAGAAAGTTGACTGGCTACTCAGGTCCCTTTTTGAAGATTATCATTCCNTTCAAAGTTCTTCATCTTCCTTGAGGAGAAATTTAGCA[TACACACACACACACACACACACACACACACACAC]TAANAATTTATATATTTTTTAAAAGTTCCTTTAAAATATAAAAAGTGCTGGAGCTGGTGATGAGAACAGNGAGTGACTAGAAATGGGCACATGAAACCTGTTTGGAGTGATGGAAATATTTTAGAACTTGATTG

>A1:160782410-160782710|varpos=160782560

GATAGTTACTAAAGGNAAATTTACTAGATGGGAATGTAAGAAGTAATATGAAGTGGAACAATTAAGATGCAAAGTAAGGTGTATATATGTACATACANNNNCACACACATGTATGTGTGTGGTATCTACACACAAAAATATAATGGTTCTTTAAAAA[AAAAAATTAA]NCGTTTATTTGTTTTTGAGAGAGAGCACAAGTGGGAGAGGGCCAGAAAGAAGAAGACAGAAGGATCTGAAGCAGGCTTCATGNACTGGGCACGGAGCCCAATGTGGTGTTCTATCTNACCNTCTCATGATCTCTATTTCNGATCACNAC

>A1:161355483-161355783|varpos=161355633

TAAAATTGCAGTCTAAAAAATGGGAGGACACTTATACACANNNAACTATGTATCAAATATAGGGTTAATATCTAAAATATATAAAGAACTCCAACNACTCAATAACAAGGAACAAACAATTTGATTAAAAAATGAGCAGAGAAACTAAATGCAC[GTTTTTTTTTTTTT]NNNNCCAAAGAAAACATGCAAATGGCCAACAGGTACACNGAAATATGCTCAATATCACTAATCATCTGGGAAATTGAAATCAAAACCACAAGGANATACTACCTCACATTTGTTAGAATGGCTATCATGAAGGAGACAAGAGATATC

>A1:162686753-162687053|varpos=162686903

TTAAGAATAGNATTTCTATACTTATTTTGCCTTATTTTCTGAATTACATAAAAGGGGATATGTATGAAATCTGTTATCTAAAAACATAGTAATTAGTAAAATAATGTAAATAATAGCCATTGTTGAAACATGCTTACTTTCTNNNNNNN[TTCCCC]CCATGTTTTNAAGTTTTATTTTGGCATTGCAAACATTTTTGTAGTAAGANNCCCCCCTTGGGTTTTGGTGTTTTCCAATTTCATTAAGTTTGGGATGGAAAAAAGCTCATTCTTCCCCCAAATACATAATGCGGTTTTCACCATGTATTA

>A1:163356722-163357022|varpos=163356872

AAATTGTCTCTGTGTACAATTATTTTGAATGCTTCACCCAATTTAAATGTGCAAAATCATAGTGTTTCTTAATTTCATTCCAATATATGAAAATGAATCAATGTGATTCACTAGATTAATATAATAAAGGACAAAACCCACACAAACAT[CAAAAAAA]GAAAAAGCATATAGCAAAATNCAAAACACTTTCATAATAAAAATACTCAATGAACTAGGAATAGAAAGCAACTTCNTCAAACCAATAAAGCACACATGTGAAAAACCTATAGCATACTTAAAAAGTGAATCAAAGAGTTATCANAGGA

>A1:164432767-164433067|varpos=164432917

TAAATGGGCTGAATAGTAGAATAATCATAGATGAAGATTTAATTAGTGATTAAGAAGAGTGGACAAGGAATTCATCTAGAATTCAGCACAAAAGACAAAAAGATGAAAAACATGAAAGAAATAATTTAGGTTCAAAAATTGTTATATCN[ATTTATTTTTTTT]NNNNNNNTTTTTTTAACGTTTATTTATTTTTGAGNNNNNNNCAGAGCATGAACAGGAGAGGGGCAGAGAGAGACGGAGACACAGAATCTGAAACAGTCTCCAGGCTCTGAGCTGTCAGCACAGAGCCCGGTGCNGGNCTTGAACTCAC

>A1:165556033-165556333|varpos=165556183

TACTTGAGTAAGTTGTATTTAACATCTTTCTCTTTGTCAATTTATTGACTGCCAAATAGGAAGTCAATACACATGAAATAATGCTTAAAAGTGTACTGCCCATTCTGGGAAATATTATTTNNCAGAATATNGGTGTTTATTTTACTCAAATA[ATTATTTCTTTT]CTTTTTATTTTTGAAATACAGTATATACACTTTTTTAATAGTTGCAAATCATGCATATGGACCAAGTGGTCATCAACTCAGACATCTAGGATGCTGTAGGCTCAGCACAGCCTCCAAGCCACTTGCTCAGNTTCCATCAGGGTGATTAC

>A1:166153827-166154127|varpos=166153977

CAGTTTGCTTTATCTAAGAAATCTTATTGATCTAGAAATCATCAGTTTTCCCAACAACTTCAATAGCTTGGAGACTGCTGCATTTGTCATCACAAAAATCATGNTACATAATTTAAGTGTTATTTAATAACATATCTGACAAAGTAATT[GTTTTTTTTTTTT]NNNNAATACTGGTTCATTTTAATATGACTACTTCAATGACAGCATCCAGGGAGGCAACTTGAATATTTCTAAATCCATTTTAGGCAAGCTTCTTCTGGGAGGTTTTCATGCTGTTAGCTAATCTCATTCCTAGTGATTTAATTATTA

>A1:167042533-167042833|varpos=167042683

TGGACCTATCCAGGAGTCAAAGCCTTTNGTGAATAGGTNCATTTCTCGCTTACCCAATGTTTCCCATGTGGGCCATCANAGCNAAAGAGGGTGAGAAGGCCTGTATTTTATAATGNTTTATTTTTAATTTTTTNAATGTTTATTTATTT[TGAGAGAGAGAGA]ANAGANAGAGGGACAGAGAACAAGTGGGGAAGGAGCAGAGAGAGAGGGAGACACAGAATCTGAAGCANGCTCCAGGCTCNGAGCTGTCAGCCCAGANNNNNATGTGGGGCTCAAACNNATGAACCNTGAGATCATGACCTGANCNGA

>A1:167655363-167655663|varpos=167655513

ACCCCATTAAGATGGATGAGAAGAACTTCACACCTTTTGTTTCAGTCTGCTGGGCCCTGGACTTAACAAAACAGCAAGGCACTGATTTAGGGAGGGAAGATTATCTGCACAACCAGATAGGCTAAAACTTCCTTTAANNNNNNAAAAAA[AAAAATTTTT]TTTTAAAGCCCTTCTAAAACTTCTATGTTTGAAACACTCTTCAAATATCATTTTGAGAATGCTAACCCCAGAGTTACCCCATACCTGAACTTACATATATATTTGGCATGGTTCTTCAGTAATGTTTATAGAGCTCTGGGTTTCTTCTCT

>A1:167786612-167786912|varpos=167786762

AAAACAGCTGCAATAAATCTTCTAAAATGGTANNNNNNTCCTATCAAAAGGTCAGAAATTCTGTTATTTGATTAAATAACCCAGAAAATGTTATTTATGTAGCAATTCTACTTAATAAAAACATCATAAAAGCTGATGCAAAAAAAAAA[AAAAATTT]TTAAAACTCTGCACCTATGGCAGAGAAGCTGAATGGACACAGAAGACAATATAGAAACAATAGNAATTCAAGTAAAGGGTATTAATATTTAGAACAATTTCTCCTCAAGTACTGGTACTTTTTATGGAATGAGCTTCCGAACATCACTTG

>A1:168011753-168012053|varpos=168011903

AAATGCTACTCAACATTTTANATTAAATTCAGTTTATCAAGGATTTAAAAATGATTTGCTTCTATTTGATAANTAACTTAAAAATGTTGGTAAAATTTGTTACAAAAGTAAAAGGATACTTGAATTAAGAGTGTTTGCCATCCTNTCCCTNNNNN[TTTTTGGGGGGGG]GGGGGGGTTTGTTTTTATTGAAATATAGCTGATTTACAGTACAAACAGTTCCATCTTTCTTTTTATCGTTCCGTAGAGGTATATTTCAGTTAAGTCAGTTTACTCGCTGGTGCTTTCAAAGACTTTAAAGTTCACCTAGGAGATTAGTAA

>A1:168304418-168304718|varpos=168304568

GTTCCACCACACTCAGCCAATTAGTGTGATCAGGAAGTAACATGCTAATAAACAGTTCTNACACTGGAAAGGAGAGAAAGGAAACAAGATTTTTTCATTGGTTAGAAAAAATGGCTTCAGGCCAAATTACCAAAAAAAAAAAAANNNNN[AAAAGAAAAAAAGAAA]AAATCACCCAGGAAGAAGAGAATCTTTAATTCCTGTTTAGAATATACATTTAGAAATACATTATTTAAAAAGGACTATCCTTAAAACTGATATTTTTGTGAAGAAAACNTTNCACGTCATTTGTTGGAGCTTCACAAAGACTTTAATG

>A1:169031219-169031519|varpos=169031369

AAACTAGANTTGGCTGTACCATTTTGTCTTCTGACCAGCAACAAATGAGTGATCCCGTTTTTTCATATCCCCACAATCATTTGGTCTTGTCATTATATTTTATTTTAGTCGTTCTGNTAAGTGTGTAGTGATATCTCATTGTAATTTTT[TTAAAAAAA]AAAATAATTTATTTAATTTATTTTTTAATAGGCTTTATGTCCAGCATGGGGCTTGAACTCATGACCCTGACATCAAGAGNNNCTGAGCCACCCAGGCGCCCCTCTCATTGTAGTTTTCATTGTATTTTCCTGTTGGCTAATTATGTTGAA

>A1:169426806-169427106|varpos=169426956

CTTTTGGTTATGTACCTTCACCTCAGAGGAGGCATCCTGGAATTGCACTCCCTTCGCTTGTGTCAGTTTCCAGGGCTGAAAGAATTTCATGATTCTAGTTCATAAACNTATTTGTGTGTGTACACACATGAACATGTGCTANACACACA[TACACACACACACACACACACACACACACACA]ACCAATAAGCAGGAGTAATGGTTAATAAGTTGAGGGACAGCCCTCCTGTTCCCCTTCCCCCTTCATTTACCCTTATAATGCTACCTGGAAATCCTTATCTTAGCTGACAGGTATGNGAGCACAGTTCTGGAGA

>A1:169439782-169440082|varpos=169439932

TTGCCAGAGCTAACTCAAGAAATATTCAGAATAGGTGTAGAACAGCTAAAGAAATTAGGAGTTTAAAATCTTCCCACAGCATTTAACCCAATGAATGAAGAGTTATTTCATGCATAAACTTGTGCACAAATGTTCATAGTAGTTTTTTT[TTTTCC]CNCTACTTAAAGCTGGAGGAGTTTAATAATAATACCCTTTAACATGGTTCAAACCACTATAAACCTATTCANGGATGAAGTTCNTAGGCTTAGGAGAGAGAGCATACCATAGCAGTATGTTAGCAAGATAAACTATTATGTTTTAAACCA

>A1:169474511-169474811|varpos=169474661

GGAGGCTTCAGAGCCCTGTCAGGAACTGACACTGGGCCTGCCAGCCCNTAGTCTCAGTGTTTTCTCCTCTCTGTGGTTATGAGGGTCTGTGTTGCCACATAGTTTTTGTGATCNTGTGGTTTGGGCTTTTGTTTTTNNNNCTTTANTTAAAAAA[AATTTTTTTTT]TTTTTTAACATTTATTNATTTTTGAGAGAAAGAGACAGAGCANGAGAGGGGCAGAGAGAGGGGAAGACACAGTATATGAAGCAGGCTCCAGTCTGTCACACAGAGCCTGACGCAGGGATCAAACTCACNAGCGGTGAGATCATGACCTAA

>A1:169554131-169554431|varpos=169554281

GAAAACTTCATTTTAAGACTAGCTGTGGGGTGCCTGGGTGGCTCAGTTGGTTAAGCACCCNACTCNTGATTTAGGCTTGGGTCTCAGTTCATGGGATNGAGCCCCGCATCAGGCTTNTGCTAAGTGTGGAGCCTGCTTGGGATTCTCTT[TTTTTCTCTCT]TCTCTCTCTCTCTCTCAAAATAGATAAACTTTAAAGAAAAAAANNNNNNTAACAAAAACTAGCTATATCTTGTTAGGTGNAAATAAAGTTGCATTGCTGTTGAATAGAGTTCAGATGNGTGTAGAAAGGTGNATATGGGATCCAAGTAATCAAAGGG

>A1:170672313-170672613|varpos=170672463

CACCTTTTATTTAAACTTGTTTGGATGAATAACAAAGGAAAAATAAAAACNTTAAAGGAAGTTTGTCCTTGCTTTTTANGAATGAGGAAAAGAAGTAAANCTCAAAAAATAATAAGCAAAAACGTTGTTATCAGTTTGTTAAAAAAAAA[AAAAT]TCTTCCTGTTAAATTGAAGTTGGTGGGTGAGGTTCTTGTATAATAAAATAATAAGCATTTGACTGACCCAAACTTAAACATTTTAAAGTTTCATTTCCTCACATGAATGATCACAGTCAGTNNNNTTTACTACAAACTGTTTAGCACACAAACGCAGT

>A1:171016372-171016672|varpos=171016522

AGTGGACATCACTTTCATGTTCCTGGCCTTAGGGGGAAAGCTCTCAGTTTTTCCCCATTGAGGATGGTCTCAGNNGTGGGTCTTTCATATATGGTCTTTATGATGGTTTAGGTGTGTTCCTTCTGTCCTTACTTTCTTGATGNNNNNNN[TTTTTTAA]ATCAAGAAAGGATGCTGTATTTTGTCAAATGCTTTTTCTGCATCTGTTGAGAGAATCANATGGTTCTTAGCCTTTCTACTATTAATGTGATGTATCATGTTGATTGATTTACAAATATTGAATNAGCNCTGCAGCATAGGAATAAATCCC

>A1:171117094-171117394|varpos=171117244

TGTAAGGATTTCCACAAAATACCCACTTTACAGACATGACTATGTGCTATTTGGATATTTTACTGTAGTGAAGTCNGAGGGGAGGACAGTGAAATGGNNNNNNNNNNTAATGTATGCTCTTTGAAATTATGTGTCAAATTGTATGAATT[GATTGTCTT]TTCTTTTTTAAAGTAATTTCTTGCAGTATGGGCATATAAAATGATAGGAGCAGAATCTTGCATATAGAATCCTTAAGTGTGTGTCTTTTTCCCCCATTAGGACAATTTTTTCTTAAGAAGCCACTCCTCAAAAGNATAATAGAAATTAAT

>A1:171812902-171813202|varpos=171813052

AAAAAAATCTGTAAAGGCCTTTTAACCAATTCAAAAGGAGTCAAAACATGGAAGATAAGGGGANGGGATGAAGCCTTGGTCTCAAATGTAAAAATGTGATATTGTTAAATGNTGATTTTGTTATATAATAAAATTATATGCTTAAAAAA[ATTTTTTTT]AAAGNTTATTTTGAGAGACAGAGCATGAGAGACAGAGAGAGGGGAGGGGCAGGGAGAGAGGAANGAGATAATCCAAAGCAGGCTCTACACTGTCAGCACAGAGCCTGNTGCAGGGCTCGATCCCATGAACCATGAGATTGTGACC

>A1:172279778-172280078|varpos=172279928

TGATCTCAAGGTTTGTGGGTTCAGCCCTGCATTGGGCTCTGTGCTGACAGCNCAGAGCCTNGAGCCTGCTTTGGATTCTGTGTCTCATTCTCCCTCTGCCCCTCCCCCACTTGTGCTCNGTCTCTCAAAAATACATAAAAATGTAAAAA[AAAAAAAAATTTTTTTT]TTTTTTTCTAAATGAAATATAGAATTTCCACTCAGGTTAGTAGAAGCTAACAGCACTTAACTGTGTCAGGGACTGCTGTAAATACTGCATCATGTATATACTTCTCCCATTTTGTAGGGGAAACTGAAACACTAAGCCTTTAAGTGACTA

>A1:172430509-172430809|varpos=172430659

ACTCTNCTCTTCAGCACTCCAGCACAGAGCTTACAGTTGTNGAGAGAAAAGCTAGTGTAATAGGAACGTAAGCAGCTTAGGAGAAAGTNNNNNNGCCNCCAAGTTCTCAAGAGTAAAAAAGAAACAAACCATAATTACAAGTTTTTTNN[TGGGGGGGGG]GGGGGGAAGCACCCAAGAAAACAAACACCCCCCTTACTAATGAGTCTCAGAAGAGAACCACAAGTACACATCCTCCAGCACCATTAGGGAAGCCTCACTTTGCGTCAGTGGACTTTGTTTTCCGAAACAGAAAGTGGTGCTCAACTGCCA

>A1:174364212-174364512|varpos=174364362

CTATATCTTGACTCTCTCTTCTGTTCCACTGATCTATGTGGCTACTTGATGCCAATACCATGCTATCTTGATTAATGTAATAATTCTTGAATTCAGATAGTCTTAGACCTTCAACTTTGTTCTTCTTTTTCANAGGTGTGTGTGNNGGG[GGGTGGTGGTG]GTGGTGAATTCTGGCTCNTTTGCATTTCCATATGAATTTTGGAATTAGCTTNTCAATTTGTACAAAAACTTGCTGATATTTTAATTTGGATTGTGTTGACTATATGCATCCATTTGGGGAGAATTGACATGTTAATAATATTGAGTATT

>A1:176212470-176212770|varpos=176212620

AATGGATANAAAACATTGTACAATGCTAAAATTAAGTTTTTAAAAGCTGCGGTGGTTATATTAATATTGGACAAAGTAGATTTCAGAACAAAGAATAATACCAGGGAAAATANNCTCATTTCATAATGACAATGTGGTNGATTTATTTT[TTAAAAAAAA]AAAAANCCTAATATGAAATATTTATGCATCTAACAACAGAGCTGCAAAAATACGTAAAGTAAAAACTGTCAGAACTGAAAGGAAGAATAAAAAAACCCACANCTATAGTTGGAGATAGTTGGAGATTTCGATATTCCTTCTTAATAATTG

>A1:180473234-180473534|varpos=180473384

TCTGTGAGTTTAAGCCCCNCATTGGGCTCTGNGCTGACAGGTCAGAGCCTGGAGCCTGCTTCAGATTCTGTGTCTCCCTCTCTCTCTGCCCCTCCCCCNCTCATGCTCTGTCTCTCTCTCTGTCAAAAATGAATAAACATTAAAAAAAT[TAAAAAAAAAAA]AAAAAAANAAGAATACATGGTTTTTGTCCTTCATTTTGTTAATGTTGTGTATCACATTGATTGATTTGTGGATGTTGAACCACTTTTGCATCCCTGGAATAAATTGCATTTGNTTATGGTGCAAGATCCTTTTAATTGCTGAATTGTGTT

>A1:180511516-180511816|varpos=180511666

GTGTGTTTGGGTTATNATTCAATACTTCATCCTATTATNTTGTTGAAATTCTTTCAGCTTTGGNCATTACACCCACTTTCAGTTGTCTCCTGTGTCCCTTCGACATGTTTCCATGATTAAGAAGTGTTTTTCTGTTTGNTTTTTTATTT[GTTTTTTTTTTT]CAGCACTTCCTTTCTTTCTGTCACTACAGGATGCTCCAGGTTNNNNNNNNNNNNNNNNNNCCTCAGTCCTAGAATCAGCTATTTTTCAAGGGGCCCTCCTCATGTAGGTTTTGTGTAGTTTTTGTTATAGTTAATGAAGTATT

>A1:181158614-181158914|varpos=181158764

CTTCTTCTCCCTGCTCTGCAATGGCAATGCCACATGNGTCATTGATCAAGTGCCCACAAAGGGTCTAGTGGTGGCAGGCTCTTAAATATTTGTTGAGTCTACCCTGAAATGAATCGGCATTCATTAGGCATCTATTTATAAGTCCTGTT[ATGTTTTTT]TTTTTTTCATTTTTTTAATGTTTATTTATTTTTGTGAGACAGAGAAAGACAGAATGCGAGCAGGGGAGGGGCAGGGAGAGAGGGAGACACAGACTCCAAAGNAGACTACAGGCTCTGAGCTGTCAGCACAGAGCCTGATGCAGGGCTCG

>A1:182213775-182214075|varpos=182213925

GTCACCAGCTCTTTGTCCAAATCAGTCAATATAACCACTGGCCTTGGCTCAGCTTCTCTATTGTTAGGTTGCTCATGGCAAAGAAAGAAGCNAAGGAAGAGAAGGAGGAGTAAAGGAGAGGAGAGAAGAGAAGAGGAAAAGAAAAAAAA[AAGAAAAGAA]AAAGAAAAAGGAAACCCTCCAGCTACGTAAACATAAAAAGGGGGTTATTAACTCATGTAGATGAAAATTCATCATGTGGACTCATGTACTTAAAAAATACCTGGAATCTATCTCTNTGTAATCTTTAGCTCTGTTTTCTTCACCTCTTA

>A1:183988493-183988793|varpos=183988643

GGCAGTGCTGCCTTAACAGAATACAGTGTTAAACTGGCAAATTTTTTCTGCATCNGTTTTTCTTGCTGTGGATAACTGAGACCANAGAAAGTAACATGACATCCAATATTTTGTACCTCATATTTTTGGCAAAGAAGATCANNAAAAAG[AAAAAAACC]CAGTATGAATTCTGGTTTGAGAAACTTCAATATTAAGTTAGTCACAGAAGAGATGACCATAATGTGTNACCTCTCATGAACAACAACAAAATATCCTAGCACAGATCTAATCAGATACTAGTTGGGTAAATGAATTTATTGACTGAAACA

>A1:184206611-184206911|varpos=184206761

TGGAAGGATGTGCTACTGATGCTCTTCTNTACCCTTTGGATTTCGTGTAGTGTGTAATAATTAGCTATTAATGCAATGCAACTAATTGTTACAGGCAAGAAATGATTTCGGGGTAAATAACCTGACTTTCCCCCCAAAGAATGTAAATA[CTGTGTGTGTGTGTGTGTGTGTGTGTGTGTGTGTGTGTGTGTGTGTGTGT]TAGCAATCCCAATCACTTCTGCTTCTTCATGCTGAAGGGTGGAGTGTGTGTATTTGGCANGGAGTGCACTGGGCTGGATCAAAGAGGTGTCTGCAACNTGG

>A1:185578899-185579199|varpos=185579049

CAAGAAGGTAGTCACTAGCCACATATGTCTGCNGAGCACATAAAATACAGCTAATGTGACTAAGGAACTTANCTGCCAATTTCATTCAAGTTTAATTAGNTTATAACTAATGTTTTTAAAAATGAAACTGAATTCAGTTATTAAAAAAA[AAAAAACC]CTTTTAAGTATGTGATGAACAAAGTGGGNTTGGCAATCTACCTTTTTAACCATAANCTTTATAAACAGCAAATATAACTCAAGTATTTCTGATGAACATTTGCCATCAGAATCAAGATATACTCTAAGTGGAAAATAGAACTGGACATAG

>A1:185760409-185760709|varpos=185760559

ACAGGCAACCTACTGAATGAGAGAAGATATTTGCAACCAATATATTCAATAAGGGGATGATATCNNNNTATATAAAGAAATTNGTTCAATAACAANNTAACAACAACCAAACTCACAAACAGTCTGATTTTAAAATAAGCAGAGGATCTGAAAA[GAAAAAAAAAAAAA]CAGTTTTTTCCAAAGAGGACATGAAGATGGCCAAGAGGCACATAAAAAAATGCTCAACCTAACTAATCATCAGGGAAATGCAAATCAAAACCACAATGAGANATCTCCTCACCTCACACCTGTTCAAGTGACTATCATCAAAA

>A1:186029656-186029956|varpos=186029806

AATGGGCCTAGTTTATTCTGGTAGTCCCATCACTAAAATTTGCTAAATGTTCATTANCTCAAAACTCCCAAGGACCAGAGCCACTAAAANNNGGAAAAGGGCTGTCCTTTCATTAAAGTAAACTACTGAATTTTAGATGGTGATAGACATATT[TAAAAAAAAAAAA]AAANNNATTCTATGAAATTATAATAATCATGTTAATTTGGAGGTTTCAGTAAAAAATAAGTTAAACATAAAAAAGGAAGTACAAAAATGTCATTGGAAAACCATGCTTGTGAGTANNNNNNNNNNNNGTTTTGTTTTGTAATGGAGATGA

>A1:186274427-186274727|varpos=186274577

AGATCTCAAAGCTGCATATAAAATGTACTGGCAATGGTAATATAAGATTAGTGAAATTTTCNTAATTGTGCTATTTTGTTAGGAAATTAAGGAATTTTCAAAGTGAAACTCTCTAAATGTTATATTTAATACATGCATANTNNNNNNNN[AAAACCC]CCTCAGCAAATGAATAAATGCATCAGGGAAAGCAATACATCTTCCAGAGTTAAATTTGTGCTTCTATGATGTACAGTGATTTCACTTGCATGTACATATTTAATAGGTGTGTAATCTTCTAGTTTGAATGAGAGTCATCATATATGCAGG

>A1:186341229-186341529|varpos=186341379

TGGCATCAGNAAAAGGCCTAAACCTTCAGAAGGCTATATCAATCCCCCANAAGAAGTTTCTGTGGTGGGGCCANNNNNNNTAGTGTCAGTTTGTGATTTATTAGTGTACAAAGGCTGAAATCCAGGCANCACAAAGAAAAANNNNNNNN[AAAACAAAC]TTGTGAAAATATTTGCCAAGATGGTTACCTCAGTTATGACTNCCTCTAAGACAGTAGGATCACTGACACTAGGCAGGATCTGGGCCTATATGCCCTAGNAGGCACCCAACCCTGCTGTGAGTTTTTAGAACCAAGATAGTTTGGGAGGATAGANC

>A1:186368920-186369220|varpos=186369070

AGAAATAGNTAACTAATATAGTTGCTAACAGAAATACATAACTAATGTTGTTGATTCTTTTTGGAAAAGATACATTTTACCCTAAGATCATGCCTTTCCTACATTTTTAGTGTTAAAAATTGCCTATATTTTATGATTNTATTGTGATA[GTTTTTTTTTTTTT]TTTTTTTTCCCCTAAGCATATAGCTAGCAAAATAAGTCTATCTGAGNGCATAGAATTTCTACTTAAGTTTTCTGATTAATGAAATANAAAAGTTTGGGAACTTGGGCTCCAAACAATTTTCTTATCATTTACATTCTTCTATTATNACNA

>A1:187938658-187938958|varpos=187938808

GATCTCATGGTTTGTGAGTTTGAGCNCCATAACAGGCTCTCTGCTGTCACCACAGAGCTCACTTTGGATCCTTTCTCTNACTCTCTCTCTCTCATTNAAAAATAAATAAACATTAATAAATTAGCATTTTATTTTGAGAGATTCCAGAT[TTGTGTGTGTGTGTGTGTGTGTGTGTGTGTGTGT]GTGTGTGTGTGTGTGTGTGTGTGTGTATTTTTTAAGGGAANATCACCATTTGACAATGTAAATCTGGGAAATGGGGGNNCAAAGCATTTAACCTCCTGTGTTTCCCTTCTCNTAACCATTAAAGTGAGAGTAAAAATATATTTTGTGCT

>A1:188088677-188088977|varpos=188088827

AATAATCTCAGGNCCAACNCAGATTAATTTGTTTGAATACAAATGACCCTTNAAATTATCTGCTTAATAAAGGAAAGAGCCAATCCTCTCTGGATAAAAGTAACATTTCTTTAGTCACTATTATTCTTTAAAAACAATTACTGGAATAT[GTTTTTTTTTTTTTT]AAGTTGGGACTTGAAAANAGTAAGGAAAATATGATAATAAATTAAGAGAAAAATCAGACAATAGAAGCAGACCTAGAAATTGTTGGAGACAGTAGTCAAGTTGTTGGAGATAGTAGGCAAGGACTTTTAAAATAATTGTTAT

>A1:190369851-190370151|varpos=190370001

NTTAATGAAATAGTCTATCCTTTCCCTATTATTTGAAATCTTGTCTTTATCATATAGGGAATCTCATCTGNCCTTAGGTCTATGTTTTTACTCTGTAATTTGNCTCATTAATATTTCTCTCTCTCCATCTTTCTCTCTCTCCCTCTCTA[CATGTGTGTGTGTGTGTGTGTGTGTGTGTGTGTGT]NTNTGTGTGTGTGTGTGTGTGTGTGTGTGTGTATAGTGTAAGCATAATNTTGTTTTAATTATTTTAACCTTAAATATCAAGGCAAATCTCCCCTCATTATGTTTCTATTTATTTATACTTAACCATTNTGAGATAGTNACACTTTCATAC

>A1:191080331-191080631|varpos=191080481

GCCTCCCAACTCCACCTTGCCTTTTCCACAAGTAGCTACTGCCNTCATCTGAAGNTGCTACACTTGACTTCAAAATGACAGGGGAGAAAAGATCTTGTTTCCTTGGTNATGTTTATTTGCTCANCAGGTAAAAAACAAACAAACAACAA[CAACAAAAAAAAA]NNNNNCAAGGTCAAAGAGGTGAATCTCAGGTGCTGCCACAGTAGCTCTTGCCCCATTCTCACCATCCAGCATTAACTCCTTGCTGCCTGTGTGTCCAGCATAAGGGAAATAGCCCTTTAGGATTTAGGGGCTCAAAAGTCTTGCAGAC

>A1:191233268-191233568|varpos=191233418

NAGGGTGGTGGGAATGTGCAAGCATTGTGACAGGCTCATGCTAGATAAGAACCNTGNCTTCCTTCCTCTCTGAGCCCCGTTACAGAGTCAAATCAACANTTCAGGTATGTATATGTGAGNACAGTTCAGTAGATGTGCACATAGCATCG[CTGTTTGTTTGTTTGTTTGTTTGTTTGTTTGTTTGTTTGTTT]NNNNTTACAAGGCTAGCANTGGGACCTTCTGGTTTCAAGTAGTTTATATTTCAAANTATTATCTGTATTATGTTNTCACTTCCAGAAATGTTTAGGGAATTACTTAAAATACAAATGGGTC

>A1:191293498-191293798|varpos=191293648

NTATTACTCATACTCAAGAAATTCTGCACTACAGGATGGCCCAGGTCTCTTCNGGATGGTCAAATCCAAGATTCTATGAACCTCCTAACCTTATGAAAGTAGAATTAAACACTTTCACCAATAAATGGTACAGGTATGACCTTAGAGAT[GTTTTTTTTTTTTTT]AATTANAAATAATTTAAATAATTTCATTCANCANAATAAAGTCTATGTATCCTTTTATCTTCNTCTGAAAAACTTTGTNTTTANGACAAAGTGTCCATGGGCANGGCCTAGAACCAACTGGTCACCACAATNACTCACCCA

>A1:191415678-191415978|varpos=191415828

AGAGGGCCTCTTTAAAATNTAAAAAAATGAAACACNNNGTTTCTGGNGAGTTTTCCTTGGGGAAAGTTAGATGTTGGAATATCCTTAACTTCAAGGTAATTTATACATTTCAGACAACTGAAGGCNTTCATTTTTTGATCAAGGGAAAA[AAATATATATAT]TATATATCAATGTATAGATTTTTTTAACATTTATNTANTTTTGAGAGACACAGAGAGACAGAGNNTATGAGCANGGGAGAGANNNNNNNNNNNNNNNNCAGACAGACACACACACACGCGTACACACACACACACACACACACACACACACAC

>A1:192533656-192533956|varpos=192533806

CCNGGGTGGCTCAGTCAGTTGGGCATCCAACTTCATCTCAGGTCACGACCTCATGGCTTGCAGGTTCGAGCCCTGCATCAGGCTCTGCNCCGATTCAGATTCTGTGTCTTCCTCTCTCTCTGCCCCTTGCCCAGTCACACNNNNNNNNN[CTCTCTCTCTCTAAATAAATAAATAA]AAATAAATAANTAAATAAAAATAATAAAAAANNNNTTTTAAATGAGGAAAACTGAAGAGTATAGGCCCTATCTAAAGAGTGGTACACTTTTTAGCTCTAGAAAATTGTTGCCATATTTTGTGATGCAAAAGATAATCTGGAAACCTGGA

>A1:192947853-192948153|varpos=192948003

TCAGAAGCTGAGCACCTTCCTCATTCTCTTTGCTCTTTAGTTTTCTCATTTNTTCTACNTNGATANTAAGGCCTGGCCTGTCTACATTCCCATGTAACTGTCAAATTCAAAAGTGGCTGTGGCCTAAAAAGCCAAGGGACTAGCNGAGC[CATGATGATGATGATGATGATGATGATGATGATGATGATGATGATG]GCAGAAATGAGGATAAACAGTGGCTTTCCTTTGGGGGNCTGAATCCTTCTCTCTGGCTCAGGAATGCNTCATCTGCACAGAAGGCAGAGAGANCTGTGCATGGAG

>A1:193860723-193861023|varpos=193860873

GATTTTTTGCATCAATATTCATAAGGGATATTGGTCTGTAGCTTTTATTTTTCTTGAAATGTCCTTGTCTGGCCTTGGTAACAAGGTAANGCTGACCTCAAAGAATGAGAATNTATTTCTTCCTCTTCAAATTTTTACAAAAAATTTTT[TTTAAAA]AATATGAAATTTATTGTCAAATTGGTTTCCATACAACACCCAGTGCTCATCCCAACAGGTGCCCTCCTCAATACCCATCACCCACCCTCTCCTCCCTCCCACCCCCNATCAACCCTCAGTTTGTTCTCAGTTTTTAAGAGTCTCTTATGT

>A1:193893134-193893434|varpos=193893284

TTAAATGTCCAACTTCNGCTCAGATCATGATTTCATGGTTCNTGAGTTTGAGCCCCAGGTCGGGCTCTGTGCTGACAACTCAGAGCCTGGAGCCTGCTTCGAATTCTGTGACTCCCTCTCTCTCTGCTCCTCCCCTGCTCACACTCTNT[TTCTGTCTCTGTCTCT]NNCTCTCTCTCTCTCTCTCTCAAAAAATAATCATTAAAAAANNAAAAAAGAAATAAAAAAGAAATTATCAGATGTAGGGCCANGTTAGTTCAGAAGGGGTTGACCTTTTCTCCCACTGTGGGGAAAGGGTGACGCTCTGTGGAAAGCAG

>A1:194386795-194387095|varpos=194386945

GACAAANNTGTTAACTCTTCTGATCCATGAATATATGGGATTTTTTNCCTCATTTATTGTGTCATCTTCAATGTCTTTCACTAATGTCTTAAAGTTTTCANTGTACAGATCTTTTACTTNCTTGGTTAAATTTGCTTCTNTTTTANTTTTTT[TTAAAAA]AATGTTTATTTAACTTTTNNNNNNNNNNNNCNAGAGACAGAGCAGGAGTAGGGGAGGAGCAGAGAGAGAGNGGGAGACACAGATTCNGAAGCAGGCTCCAGGCTCTGAGCTGTCAGCACAGAACCCGATGAGGGGCTCGAACCCATGAAC

>A1:195879352-195879652|varpos=195879502

CGCTCAACCTACTGAGCCACCCAGGTGCNCCCCGACTGTATTTTTAAAATAGAACAGGACTAGATTTCCCTTGAAAAGAGTTTCAGTTTTATAGGTAAAATAAAAGTTTCTTTTGTTTCTAGTCCCTATCCCAAGTCCCTCTCTAGAGC[TGCTACAGCC]CGCTTGGTGTGCATCTTTCCAAACCTTTTTCTATGTGATTTAAGATTTTACACACATGTATGTNCATAGACGTGTACATGTCTGTGTATNTACACGTACATGTACGTACATATANACACACATGCAGTTGTATGTGGGGTTTTTTTAAAG

>A1:196825531-196825831|varpos=196825681

TTAGTTAAACATTGGGGTTATATGATCTCTATTTTTTGTCCCCTCACATATTTCACTCATCAGTGCAGCAAGGACACTTTTTTAATCAACATANNNNNNNNNNNNNNNNNNNNNNNATATNNNNNNNNATATATATGTATATGTATGTN[TACACACACACACACA]NNCACACACACACACACACACACACACATACATTTGTAGCATTATTGTTAATAGATGTTCCATGATNCATTTAATAAGTCCCCTCTTATNAGACACTTAGGTTGTTTCCTAACTTTTCATTACTATAAATGAGGCTGCAGAGGACATTG

>A1:200455956-200456256|varpos=200456106

GTGAGAGTAAACATCCCTATTGTGTTCCTGACCTTAGGGGGAAAGCTCTCAGTTTCTCTCCAATTANGATTAGCTGTGAGTGTTTCATATATGGCCTTTATTATCTTGAGGTATGTTCCTTCTATTCCTACTTTCTAAAGGGTTTTTTT[TTTTAA]ATCAAGAAAGGATGCTGTATTTTGTCATATGCTTTTTCTGCATCTATTGAGAGGATCATATGGTTCTTATCCTTTCTTTTATTAAATTGATGTATCACATTGATTGATTTGTGAGTATTCAACCANCCCTGCATCCCAGGAATAAATCCC

>A1:200628384-200628684|varpos=200628534

ATTCACTTTGCCAATGAAGGAGTTGAGTGTCCAAATTTATAATTCGATCTATTATTTAAATATCCCTAGGGGAAAACAGATGTATTCCTCTCATCTCTATAAACTGAAGTCCATTTGTCCTAATAAAATCTATCTCTCCATCNNNNNNN[CATCTATCTATCTATCTATCTATCTATCTATCTATCTATCTA]CCTACCTACCTACCTANCTACCTATTGACATTGTCTATGCAGCTGCAGTTTTTAAGAATTCTGGTAATATAATTTTGCAAAATAATGTTTTTNNNNNNNNNTTGTTTTG

>A1:202295616-202295916|varpos=202295766

AAAGGCTACATATTGTATGATTCCATTTNTATAGCATTCTTGCAATTNCAACATTATAGAGATGGGGAACAGATCTATTTGTTACCAGGAGTTAGGGATAGGGAAGACAGCTAGTTTGTTGTGCTATAAAAGGACAGCATGAGGGATCT[TTTTATTTATTTATTTATTTATTTATTTATTTATTT]TTAATGTTTATTTCTTTTTGAGAGAGAAAGACAGAAAATGGGTAGAGGAAGGACAGAGAGAGAGNNNNNNGGAGACACAGAATCCAAAGCAAGCTCCAGGCTCTGAGCCGTCAGCCCAGGCCCNGATGTGGGTCTCAAACT

>A1:203578318-203578618|varpos=203578468

TTTTTATCAAGGGCCAAGACTGTAGGGAGAGGAATGAGACAAATGAGGCTCTTCCTTCAGGCATAAAATTTAAGAGGCACCAAAAACTCAGTAAGATAAATAGTATTTTGATACGATGTTTTTAAAAATAAAAATTAATGCAAAAAAAA[AAACCC]CAATGGTGAACAATNTATCAAAAGTTGAAATANAAACACCATAGATATTTTGTTATCCTAGTAGCATAGCAGGGAATATCTTATGGAAAATACAGGGTCAAAGAGCAGAGGGAGGATTTCTTGTTCCCTATNGATCACTTACATGTGGTA

>A1:203663776-203664076|varpos=203663926

TTCCCATGTCTCAAAAATATATAAAGGCATAATGTTTTTAGTCCTATANCATGTTTTTGTCACTGCACATAGCGATTACATAATTTATTTCTTAATATTCTCACCNCCCNCAGAAAACTTACAAGGTGGTGATAGGATATTAAAGCATT[TTTTAAAAAAAAAAA]AAAAAAAAAATCTAAAGGCTTATTATTGAAGGCTTTTAAAATTAACTACACCAAAGTCCTTTGCCCACAGCAGATTTCANTTCCCTTACAATTTTCTACTGGAAGTGGCTTCTACTTTTCNTCATGTGTTATTTCTANGAGTTATTTTCT

>A1:204855247-204855547|varpos=204855397

TGTTTAAATGCTGAGAGGAATGGACAGTAGAAATAAATTAAAGACAGAAAAGAAGGGACAATTGAAGATGCAATACCCCANAANAGAGACAAGAGTTAAACCCAAGTGGAGGAATTAACTTTGGATAGGAAGAAGGNNTTTCTCTCTCT[CTCTCTTTTTTTTTT]TTTTTTAAGCTAAAGAGGAAGGTAGTAAGGATATGTCCAGGTATGTATATATTTGGAAGAGGTATAGTNGGAAGAAGAGGTACTAGGTGGTAAGAAATGAGATAATTTATGTCTGACAGCTTTCACTTTTTCTGTGAAATAGAAAGTAA

>A1:206821700-206822000|varpos=206821850

TGGACTTAGTTTTTNNNNCTGACAGCTAATTTTGTATTCATCTTTCTTGGGTCTTCTAAGTCATTTATACTTGTTCTGCTTTCTGGCATTCAAAATTCGATCACTGTTGACTCCCCTGCTGCTGTCTCCATCTTTGTGGATNNNNNGTTNNNTT[TTCCCC]CCCTGTGTGTGGCAAGAGTGAATAAAACTATGTGAATGTGTTCAGTCTGCCACCATAGCTTTTACAGATGTTCTATTACATTTTTTCAAATCTGTCTACTTNATTTNTGTAGTGATTTCATTATGGTTCCTATTCCTTTTTATTTTCTTCAT

>A1:207271082-207271382|varpos=207271232

CAGCAAAATTTAAATCCCATACAGAACCTAAGTGTGAAAACAGACTGATAATAAGCCGAGCTGATGNNTGCTGCATATCCTTTTACTCTGCCTGGGAGGCAGTTTTACATTTCTCTAANAAAATGTTTTAAGTNNNNNNNNNNNNNNNN[TGAGAGAGAGAGAGAGAGAGAGAGAGAGAGAGA]AAGAGCGTTCAACAAGCAGGGGAGGGGCAGAGAGNGGGAGACACAGAATCGTAAATAGGCTCCACACTATAAGCAAAGCCTGACTCAAAGCTCCNTCTCATGAACCGTGAGCTCATGACCTGAGCCAAAATCGAGA

>A1:208481499-208481799|varpos=208481649

CAAATTTTCAGGACAATCCCTTAAAATNTGAGGGGGAACCCCAGAAATATACCACAGAGNTCAGCTGNTTATGTCCCACTGGCCAAATCTAGTCCAATTGCTTGCCATTATTTAAAAGAANTTTTTTTANTGTTTANTTATTTTTGAGA[AAGAGAGAGAGAGAGAGAGAGAGAGAGAGAGAGAGAGA]AAGCCGGGGAGGGGCAGAGAGAGAGAGGGAGACAGAATCTGAAGCAGGCTCCAGGCTCTGAGCTGTCAGCACAGAGCCCAACNCGGGGCTTGAACTCAGGAACCTTGAGATCA

>A1:208517350-208517650|varpos=208517500

TCCTGGGATGACAGAAGAAAAGCACTTTCTANNNNNNNNNNATTTCCTCCTTTAAATCCTGTGGAACTTTACAATGTACATAATTTNTTAGTAGAGTAGCATGTGTATAATTTATAANNNNNNNNNNNNNNNNNNNNNNNNNNNCACAC[ACACACACACACTCAC]TCAAATATTTTTCAACCNATCGTATGCATGATCCGAAAGTGTGGAGCCCACCAGTTTAAGCATTCATTTCCACTTCTAACCCTAAAAAGGACCAGATTGCCCTGGTTTATTTTACTACTTATTTTTCCAGGTTGGAACCTGCAAGTT

>A1:209138281-209138581|varpos=209138431

TCTAATATACACGCAAGAACTCTTTTTAAAATACCTTTTTAAAAGTTTGATTTAATTTGTATAAAGTTGGGTACAAGAATGTGCTTTTAGGGAGCTTATCACTGAAAATTCAGAGTCTAGTTTTATATCNTATCTAAATGACTTTTTTT[TTTTTAA]ATTTTTAAGCTTTTTATTTATTTAGTTTTGAGAGAGACAGCATGCACAAGTTGGGGAGGGGCANAGAGAAAGAGGGAGACACAGAATCCGAANGCAGGCTCCAAGCTCTGAGATGTCAGCACAGAGCCCAACATGGGGTTGGAACCCACAAA

>A1:210136657-210136957|varpos=210136807

ATTATGACCTGATCCAAAATCCAGAGTCTGATGNTTAACCAACTGACTTAATCATNTAATCATTTCTTNTTGTTTCTTATACTTGGATTTTGTTGAATTTCTTAGATCTGTGAGTTTANAGTTTTCATTAAATTTCTGACCATTATTTC[CATTATTATTATTATTATTATTATTATTATTAT]NNNNNNTATTTCTTCTTCCTACTTCAGGAGCTCCAATTCCCTATATATCAGGCCGCTTGAAGTTGTCCCAGATCTCACTGATGCTCTGTTCNTCTTTTTCTTCAGTCTTTTCTCTATTTTTCATTTGAATACTTTC

>A1:211563139-211563439|varpos=211563289

ATGATCTCACAGTTTTGTGAGTTCAAGCCCTGCATGGGGCTCNGCACTGGCAGGGCGGAGCCTACTTGGGATACTCTCTCTCCCTCTCTCTGCCCCTCTCCCACACACNCTATCTCTGTCTCTCTCAAAATAAATAAACTTAAAAAAAA[AAAAAAAGG]GTAATTTATCTTCAGTAATTCTTAGATGGGGGAAAGAAGTATACAATTATTCCACTGCAATTTCATTTTAACAATTAGAAATTTTNTTTTCTTTCCAAGCATAATTTCCTAGTGATGGTTAGGTAAACAGACAGTAAATGAAAATATCCT

>A1:212727381-212727681|varpos=212727531

CAATGTCAGACATACCTGAAAGTTGACATGAGGAAACCCTTTTTCATAGNGTGGAACATAACTCACATTCTAATTCTGCAAATTAAAAAAGAAACTTCCCTCCACAGAACTTTGCCTAAAAACACACCCTTGCTTGGTTCCTTTTTTTN[TTCCC]CCTTATATTCTAGAACTTCCCTAATAAATTTTTGTACACAAATTTTTATCTCATGGTCCTTTCCCTGGGGACTCAATCTATGACAGGGGTAACTAGCATGNCCGCAAAATNATTCTGACTCAGAAAGGCAAATTCTGATGGTACCATCTT

>A1:215165979-215166279|varpos=215166129

AGTTGTGATCATGTGACATTGTAAGACTCGTATTGCAAGACATCGCTNGTTTATCTTGCAGAACAAGTTACTTGCAATCCAAGGTTTTACTGTGTAATTTTTTTGTTTGTTTTTACAGGAAAGATAATCCACTAGGGAAAATGAGNNNN[GTTTTTTTTTTTTT]NNTTTTTTTAATTTGGTGCTGGATGGTGCTGGGTAATTTGATATGAATATAGAAAAAATATAAACCTCTACCTTTTATCTTAAATTTACAAAATTTTTCTTTGGAACATAACATAAACCTAAACTTAAAACCTCANTCCTTAAAGTTTCT

>A1:215362152-215362452|varpos=215362302

GTTTATTGATGTGTCCCACACACTTAGAAGAGATCCCAACATATAGTAGGGAATGAATACATACATGTTAAATGGGTACACCAATTTGNGAATGAATGAAGGAATCAGTGAAATCCTAGCTTCCCTAAATGANATATTGGAAGTTTTTG[GTTTTTTTTT]TTTNATTATTAAAAATGAAAANGNAGATTTATATTTTGATACANTTTNGAAGTAATCAAAATTANTGAAAAAGATCATATGTTTACCATAATGATCACAGAACAATTTTGGAGGTCNGAAAGAAAAATAAATATCTACAAGAGTAATTTC

>A1:216014134-216014434|varpos=216014284

CTTTCTCTTGGGAGAAAGCTTTCTTTTATTTGCATTATATCATAAGGGTTAGTGAGACNATCTCTGCCATTATTTGACAAACAATACATTCAGCTTATCTGGGCAAGCTCTCATGCACAGACCTTTCTCACAATTTATGACATTTTGTA[GTTTTTTCTTTCTTTTT]NCTTTTTTTTTCCTGGAAACTTACCAAATGCCTTTTAAGAGGCTAACTAACTTTGCTTTTAGNAAATGTTTATTTTCGTGGATCCATTTAGAATGGTAAGATAGAGCCAGTCGATTCCACACTAACCCACTGAATTGCACTCTTGGAANA

>A1:216214426-216214726|varpos=216214576

AAGNCATTCAGTCTANGGTATTTTTGCTATAGCAGCNTGAAGGGCCNAGACAGGCAAAAANGTAAGATAAACAAACAGAAAACATGACTCAAGTTGAAANAACTAATGCANAGAAGATATCAAAACTACAAAAANAAACATTCTCTTGG[CTTTTTTTTTTTTTT]NNNGATACTTTAGGAGATCTTTTTANACACAGGACAANGAGAAACTTTGGAACAAATGAACAAACAGAAAATAGTGAATAAGAATGATAGCTAGGAAATCANGGGTATTACTCAAAATAGCAATACAATATTTCCAAGATAA

>A1:216770786-216771086|varpos=216770936

TTTNTTTAAAATGGTAAAAAGTGTGGAGTAGAATAATAACACTTTAAAATGTTACAAATTCAAGTAAGTTCTAAAATTCAAGAGACCAAAAGTATTCACTTGTAACTCATCTTTAAAATGATTTTAAAGTTTTATATTCCTCACACAAA[AAAAAAAGAAAA]GTGTAGAAGATATTGCATTTCATTTGTATGTAGAACAGTTAGTTTGACTATAATTCTGGGATTTAGTCTAGCATTGTCCATGGTAGTCATTAATGGATATTCTTATCTCTTAAAATGTAANGGTTTTCCTNANAGAAAANTANACA

>A1:217059383-217059683|varpos=217059533

ACTTTTTCCTACTGAAGGTGTNNNACTTTCACATCATTGTAAAGCAGAANAATCATAAGTCAAAACATCCTATTCTGGGGCAGTCTGAAATTAAGTCNTCATTATGTAATATATTANCATTATTTTCTATTCTNTGTGAACTATTTTTT[TTTTCCC]CTAAGGGATNATTTATCTATCTTATTACNGAATACCTTTGTTAAGCAGTGTTAATTATTCCCCTGGCTGTGTTCTTATAGCACTTTATAACTTTACATTGCTTCNGTTAGCAACCATTATTTCCTAGATAACAAATATGTTTATATNCCT

>A1:217887810-217888110|varpos=217887960

TCCAAATAGGCTCAGCACTGACCGTGCAGAGCCTGACAATGGAGTCAAACTCACAAACGTTCGATCATGACCTAAGCAAAATCAAGAGCCGGATGCTTAACCAGCTGAGCCACCCAGGTGCCCCTAGAGTAGTTTATAATCTTTANNNN[GAAAAAAAAAAAAA]AAAAAAAAGAAAACATGGGTGAATCTGCTAAAAGTACATATATTATGATTGAGTAGGATAGAGAAATGAACAAGGCTCTGCTTTTGACAAGCATNACAAAGGAACACATTTCTGAATCCTCACAAAATAGTNTCTATTGCAATATGTTTC

>A1:218420024-218420324|varpos=218420174

TAATAAGTACTGGGTGTTATATGTAAGTGATGAATCACTAAATTCTACTCCTGAAACCAACATTACACTATATGCTACCTAACTAGAATTTAAATAAAAACTTGAAACCAAAAAAATAAGAAAAAATNNNNNNNGCATGAATTGCATTGCAATCCCATGTTTGTTN[GTTTTTTTTTTTTT]TTTTTTTAATGACCTTATATAACATTAAGCAAGATAAATCTCTGTGCATCAATTTCTCCTCCGAGATATGAGAATCAAACCTAAATTAAGGGACTGTTATAGAGATATAACNAAACCATTTTTGACTCAAGNAAAGCATCTGCTGAATGC

>A1:218813670-218813970|varpos=218813820

ACGAGGATACAGTGATGGGGAGAGACATGGTTTGCCATTGTACTTTTCATAATTTAGAAAATGCCTTGGACTTCTGGATTCCAATCAGGCATGTGAGAAGCTTAGAAGCTNCCATTCTGTCATACCAACAAGTAAAAAGCCGAAAANCT[GAAAAAAAAAAAAA]NNAAACTCTTCTTAGATACATCAGAGAAATGATGTCACAGGGAAAGCTACTGCCTCCAAAACTGGAGAGACAGACNGACAGATACAGAGAATTACAACTTTCTGTAGGAGAAACCCATGAGCAGAAATCATTGCAGGAACCCGT

>A1:219612220-219612520|varpos=219612370

GAACAGCAATTCTANNNNNNNGAAGATGATGGAGTAGTGTAGTTCAGATTCTCAGGAAAAATAATATCCAGNTTTGACTTCTTTGCCCAATGAGAAAATACATGTACATCCAGTATTTTGGATGTCACCTCAAAATTGAAGAGNNNNNN[CAACAAGAAGAAAGAAA]GAAAGAAAGAAACATACCGGATTTGGAGAGGCAAAGGATTCTGGTTAAGAAAGATCAGAGGATANCAGCTATAAAGTCTGAATCTCAACNAGTTCCTTTTGGAACGTGGTAGGGGCTCTGGGAGTTTGTCTTCTGAAAGATTAAAATT

>A1:220285137-220285437|varpos=220285287

TGGGTGTTTGTCATGGATGAATGTCTTGCTNAACAGACCATAAGAACAAATTAAANACTACATTTTACAGTTTTAGTTATCATATCGATTTTCCAGGCTTGCAGCTAACAACACCCAGAGGAATCANATACTCTTGTGTGTATTGTGCT[TAAAAAAAAAAAAAAA]TACTCAAAAAACAAAAACCAAAAGTCCCNCTTTCAGTAATGCTGGCCCAAAATTGCCAAATGCCAATTAATCTTCTATGCAGATGAGATGGAGNAATTATCTGTTAGTTTTAGGATTTGGCATATAGTTTTTTGAAATCTTC

>A1:220767644-220767944|varpos=220767794

GCTCAAAATTCTATTNTGATGGTAACTGAATATTATGGAAAACTTCCTGCATTCAGCAACAGCTCAGCAGGAGGGACAGCTAAATTATTTGANGGTTTNTGAAANCAGGAAAAAAATTACTACTAATAATAATCANGAGGTGTTANCAT[GCACACACACACACACACA]NACANACACACATCNTCAGAAGCACAATGCAGCCAAATAAAAATTAGCAAAGAATAAGTAATAAGCACTATCCTGCTTTCAGATATTTGTTCTGTAATTCCACACATCCACTGTTGGCTTGGTTTTGAGCTAAATTCTCTCCTTTCTT

>A1:220967352-220967652|varpos=220967502

ATTCCCTCTTATTTGGGTAANGTCAGCCTTTTGTTCTGTTTTGGCCTCCAATTGATTGGATAGGGAAACTCCACATTNTGGAGGGCTATTTCTTACTCAAAATCTAATTTAAATGTATACCTCATTTAAANACACTATAATAAAAACAT[CAAAAAAAAAAAA]TCTAACAATATATCTACACCATGNCCCAGACAAATAAGTACATAAAATAAATTATCACAGTTACTTAATTTATTTGTACCTCATTTTCTTGTCTTTTATTACTAAGATAAGATTATTTATTAATGTTATGAAATCACATAAT

>A1:221258752-221259052|varpos=221258902

GGGAGAGAGCAAGAAGAGAAGAAAGGGACAGGGAGGAACTATAAAAACNGAAAGCAATTAACAAAATGGCAATAAATACATACATGTCAATAATTGCTTTAAATGTAAATAAANTAAATTTCCCCAATCAANNNATGACAGACTGTTAGAATGGAT[TAAAAAAAAAAAA]NNCACACACACACACAAGACTCATCTATATGCTGTACACAAAAGAGACTCATTTCNGATGTAGGGATACACANGGAATGAAAGTGAAGAGATGATAAAAGATGTTACATGCAAATAAAAATGAAAAGAACCCTNGCATAGAGGTAC

>A1:221350706-221351006|varpos=221350856

AATATATATTTTTTAATGCTTTCCTAGTACCACACTGAGTTTCTTACTACAATTCTATAACCCTCCATGACTTACTTTTCCCAGCTTCTGGACAACTATCTGTGTTTCTCAGNCTGTCTATGGTATTTACTTTAAAATGCTAAGTGTTT[CTTTTTTTTTTTTT]NCTTTTTTTAGTTTCAATATGATACCAAANGCCAGGGAAATCTATAAAATAATAGATGAATTTATAAGATAAAAGCAATCAGCATTCATTCTATTGTATTAGACTAGTTTTACAAGTATTTCAAGTTAAAGTTACTCATCAAG

>A1:221614787-221615087|varpos=221614937

TGATCTCATGGTCATGGGTTTGAGCCCCACATTGGACTCTCTACTATTAGCACNNANCCCACTTAGGATCCTCTGTCCCCCCTCTGTCTGCACCTCCCCTGCTCCCTCGCTCTCTTTCTCTCAAAAAATAAATAAAACATTTAAAAAAT[TAAAAAAAAAAA]AAAAACCCATAAATTTCACATGGNACAGCTGATACCTTGATAAATAACATGCATNCCTTANCTGTTTGAATAATTGAGTAACAAATAAAATATATTTCAATGAGTAAAATGATAGANTCNTGACCAAAAGAAGAAAAAACATTCATGTTC

>A1:221845818-221846118|varpos=221845968

TTATCATCTGCTGCACACATGAATANGNANGCACATATATACAGGCTATTTTCCAACCATACGAAATTCACTGCGATTTCTGCCAAATGCACTAGTCTTCCTTTTGCACAGAAGACACTGCCCCACATAAGTTTCTTAGTTGATTCTTT[CTGTTGTTGTTGTTGTTGTTGTTGTTGTTGTTGTT]TTTAACTATCTCCCATGTCTCATACCAAAGACTTTCATGAGTCCCTCAGGCAAAGCTGATCACTTCTTACTTTCTTCCAAAACGTCAGCATTTCTTTCAAACGCCTACGATACTTATAGCATTAATACTTTAAA

>A1:222188981-222189281|varpos=222189131

GGANATTTATTTTGTAGAGAGGCTTNTAAGATATTACTCATTTGTTTGCTTGTATGCTTTTCTTCAGCAGGGTTCANGGATTAGAAATAGGTAGAAATCACACAGCAGACTTCACTCAGTGAAAAGGTTGGCAACTTGCTGGTGNNNNN[AAAAATAAATAAA]AAATAAAGGTGTGAAAGAGGATAGGTCAGAGATCAGCAAACCTTTTAAGTGAAGGGNCAGGTTGTAAATAGTTTCAGTTTGTGAGCCATACAGTCTCTAACAAGTACTCAATTNCAACCTTGAGTGCAAAAGCAGCCATAGGTGATACGT

>A1:222365059-222365359|varpos=222365209

GGTGTGAGCTGTCCATAAGGACTCTATTCCAAAGTGTATAGAATGGAAANGGGGTGGGGGAAAAAAGATTAGNATAACAGTGGAGAAACATGACAAAAATACTAGGTTATCAGGGTTAATATTAGCAATGTTGAACCATGTTTCTTTCT[GTTTTTTTTTTTTT]TTTTTTTCTTTTTCTTTTATGTTGCTGTTGTTGTTTTGGTTTGGTTTTTTGAGAGACAGAGAGAGAGAGTGCANGCACACGTGCAAANNGGGGGACAGTGAGAGAAGGAGAGTGAGAATCTCAAGTAGGCTGGAAGCCCAGCACAGATNC

>A1:223032644-223032944|varpos=223032794

ATACTATTGGAACTGTTNACATCTTTGCAAAATGCCAAAAAGACGTGATGGTANTGAAATAAAATTCATGCAATTCATAAAACATAGAAATGAATATATTTGGGCAAAATGTTATTTTTGTAGTCTTCATCGTGAAAAACACCTGAAGA[CAAAAAAAGAAAAAA]GAAAGATGCCTCGTTTTATACCAGTATAGCATAGAATNAAGTGGACTTACAATTAAGAATAATCTATATAGAGAAGATAAGGAATATATCGAAGAGTATATAAAATTATACTTAGATACAGATCAAGANGTAAAACTACATGTT

>A1:224111525-224111825|varpos=224111675

ACTACCAGACNTTCCAACTCTCAAAATTCTTCTAATCATTCATTAAATTTATAAGAGCAGCNTATCTAATTGTGTATATGTTATTTCCAAAATCTCAAGTCTTTGTNAGCACTGCATTTCNAATTTAATGGCAGGGGGCCCAAAGTTTA[GAAAAAAAA]GCATATTACTTAAAGCATTGCAATTTACTGAATATAANATATTTAAAAACACTGNTTCTTTTAGAGCTGACTTAAGTTTACAGCAAACCAGAGAGGACGGTGCAGAGATTTCCAATATATTCTATANCTTCATAGACTCTACCATT

>A1:224532745-224533045|varpos=224532895

ACTTACTTTTNGCATTTTTCTTCTACNGNTGATTGGCTTGGCCTTTCTTCNGGTCTGTATNTATCTCTTATTATCTNANGCCTGCTTACTTATAACTATTTCCTTATAAANGTATCTTACCATGACNCTTCNTTGGATTGACTCNAGCA[CTTTTTTTTTTT]GTCCTATTCACTCTTTTATGTCCAGCNCCTATTGTCAAATGTGTCATTTCATTCATATTTTCCAGTTTTANTTCNTGAGCNCAAAAATATAANTTACCCAGNTTAATTTTGGNGCTCATTTCATCACCTATTTTGTTAATNTG

>A1:224659011-224659311|varpos=224659161

TATATATCAGGCTGTACCATTGCANCTCAACACTANGGGGCCTTAAGGGNAGCAAAAAATAATAATATTNCATCACAACATCTTCTATCATCCATATGATGAGCCTTTCCACTTTTCTGACTTATAAATCAATTCCCATGAATGCAAAA[AAAAAAAAAAC]NNNNNNNNNNNNNTCCTCTCATTTTATAAATAATTGTGNATTATNGAATTTCAGCACAGAGGGATGAGTTAATGTCCTACTTTTATATTTTAAAAATTTATTTATTTANAAGACTTTTTCTTTATGAATCATGGTAACCCCAATGTTGCC

>A1:224953091-224953391|varpos=224953241

ACCTGAANNACATTTCTCCAAAGAAGGCATACAANTGNCAATAAAGACATNAAAAAGATGTTGAACATNGATAATCATTNGGGAAATGCAANTCAAGCCATAAGATACCGCTTCACGTCTACTNTNAATAANAANANAAAAAAGAAAAA[AAAAAAAACAAA]ANNCGTTACNAGGATATGGAGAAATTGGAACCATGCATTGAAGNGGGNTNTGTNAACTATTGCAGAACGGTGGCTCCTNANAACATGAAACAGAANTACCATAAGGTCTAGCAATTACCCTTCTGGNTACATACTCAAAAGAANTGAAAT

>A1:225494125-225494425|varpos=225494275

NGAGAGAGAGGGAGACACAGAATCCGAAGCAGGCTTCAGACTCCGAGCTGTNAGCANAGAGCCCGACGCGGGGCTTGAACTCATTCNTTTCAACAANGTATTAGAGTTCCAGTTACTCCATATTCTTACCCCAACACTTGNNATTGTAG[GTTTTTTTTTTT]TTTTTTAATTTTAGTCATTGTGTTGAAGGTGTAATGGTGCAACTTTTAAAAGTACCCNTTTTATACCTTATCTGTGGACCTTCTTAACTACCCCAATCTGCCTTAGTTCCAGGCTTTTGTCAATTTATCACCCAAAGCACTGAGTATGGG

>A1:226254838-226255138|varpos=226254988

AGCAGTGTTTCCTTCTTTAGAAATAAACTTCCAANGTCTCAACNTTATTTCTGACNTTATTTCTTATCTCCACAACACAAAATAATTTCTATGATAATAATAAATAAGCATCTCAGGGTATGAGAACTGAGAGTTATGCTNNNNNNNNN[TTTTTC]CCCTGGCTTTCTGGANNTAATTTTAAACACNTTCATNTCAAACTGCCAGGTNTTTGAATTTTAANCCATTTTCAATTTGGTGTCACCAAACAAGTANGCAACATCAATGGTTCTTATTCCTGGTAAGTCTGTCCATTCACTCATTCTCCT

>A1:226264536-226264836|varpos=226264686

CTNTATACTTNAACGAGGATCAGATCAAAATATCTTCTCNTTAATCTACCAATTATATAGTCAGTGCTGAAAAATGCTCCTTCTGCAGAAATTTNATATCCTNCAANNTGAGGTTTTATTTCACTCTGTTTGAAACCTANNNNTTTTTT[TTTAAAAAAA]AAAAATACATATGAATTCTTGTAGTTATTATTATAGCCCTTTTAAAAAATCTGTTTTTCACAGTTGATGATCTGTCTAGTTAAAGGTTCTTGGGCACACANAAGAAAACGATATAGAGTTACCAGTTGGTAATGTATTCACATTTCATTA

>A1:226379066-226379366|varpos=226379216

ATCGCTTTCCTCTGTGCACTATTTTTAAAAGAAACATTACACACAGGAGCCCTAAGGCGATGGCACCGGTGAAACAATGCCAGCTTGGAAATCCGGAGGAGACTCTGGTTTTCATTATGTGAGCAAGAGGTCATTCATTATTTAAGTGC[TTCTCTCTCTCTCTCTCTCTCTCTCTCT]NNNTTTTTCTTTAAATCTACAGCCTTTTGGTCTCCAGCCTTCCAGTGCATCCATGGTCATTATTCTTTGGATATTTTNAAATTATGAACATAGAGCCAACTGGAGTCTCACAGTCNAACTTGCTAATGGAAAGCCT

>A1:226982498-226982798|varpos=226982648

CCCTGCATCAGGCTCTGTGCTGACAGCTCAGAGCCTGGAGCCTGCTTCAGATTCTGTGTCTCCTTCTCTCTGCCCTTACCCTGCTTGNGNTATGTCTCTCCCTCTCTCAAAAATAAATANACATTAAAAAAANTTTNAAGAACAAAAAA[AAAAAAT]NNNNTAAAATGTCTGTACACAATGGTAAACTANTACAGAGGTAACAAATTCACGTGGGCCATGAAGAATCTTGTCTCTTACTCACTTCTCAAGAATGCCGAGTGGAATAATATAATGAGAATAATTAACAATCCAANGCATATATTTTCC

>A1:227837257-227837557|varpos=227837407

CTGACTTCCCTACGTCTTGATTATGGTCTACTTCATCAGAATGCACTTCCCCACTCATTCTATGATTTCATCGGTTTAGAAAAATATTACAAATTATACTCTATGTGTTACTGATGAAAATAAGTGGACAGTTGGCTATGCCCATTTCT[TTTTCTTTTTTCTTT]TTTTTTTTAATTTAAATCCAAGTTAGTTAACATAGTATAATAATGATTTCAGGAATAGAATTTAGTGGTTCATCACTTACGTATAACACCAGTGCTCATCCCAACAAGTACCCTCCGTAATGCCCCTCACCCGTTTAGCCCATCAGCCC

>A1:228814742-228815042|varpos=228814892

TCAGTTCAGGTATGATCTCACAGTTCGTGAGTTTGAGCCCNNCGATGGGCTAGGTGCTGACAGTTTAGAGCCTGGAGCCTGCTTTGGATTCTGTGTCTCCCTCTCTCTGCCTGCTCATNCTCTGNCTTTCTCTGTCTGTCAAAAANNNN[GAAAAAAAAAAAAAAA]GAAAGTAAGTGTGTTTCNTATTATAAAATATAATTGAGATAAAATATTTTAAATAAAATAATGGTTAATGCGACCATTTGGAATTTTTCTGCACTGTCAAAACAGTGAAACANTACTTGCATATGTATATACATATTTAAAT

>A1:229090464-229090764|varpos=229090614

TGAAATCATTTTCNGAGAACAAGCCCAAATGTAGCTAATTTCAGAATATAAGACACTGCAAAAATGAGAATTTCTTATATATTTTGAAATTCTAGCATAATACACAGGGCAAGTTTCTTTCTTCTAATACATTTCNGCTCTCNNNNNNN[TTTTTCCCCCCC]CCCCCTAATTTCTTTAGTTACCAATTTACTAGGCTATTATGATGAAAACACTGTTTGATATGTCTGGAGATTATGACATTTAGAAGCTGAAATAAACANGTAATTATCATTTCACTCAACAACACAGACAATTTTGACTAAGCAAGTATG

>A1:229369167-229369467|varpos=229369317

CCNAAGTATCTCTTGATGTCTGTAATATACTTTGAATTCTTTCAAAAATAAAATATTTAATGGATAAAGAGATCAATAAATTAATATCGTCTGACAAATAATTTCCTAACATATTCCTTGTAGAANAAGNGGTATGNAAATTCCTTTAA[GTTTTTTTTTTTTTT]NNNNAATTTGAAAATGTTTACAATATGGGAAAGCATGAATNTGATGACACGTTTAAAAAAAGCCAGATTGGACACNATACAAAAAAAATAAACTCAAGACTTACCGAAGGATGAAAAGTAGGAGGGTAAACTTTAAAACTACTGGA

>A1:229745162-229745462|varpos=229745312

GGTGTGATTAGCTTTACCTGTNTCCTGTTAGTTTCACCTTCTACATGTTTAGAAAACTTTCCAAAAGCACTTTGAACACACCATTGTGTTCGTTGCATTGTTTTAATACAAGATTTCTTTCAAGAAGAAGCAGAAAGTGAGGGAGACAG[ATTTTTTTTTTT]NCGTTTCCTTTGGAGTCTGCTGTTCAGTTTTAAACNTGATTCTTAAAGGACTAGTGGNTACTGTTCTGTGCTTATGTGTGTACGCAGACAGGTGCCGGTGAGCAGGAGTAAAACTACGTGTGTTGTANNTGTGAGTTTCATGTT

>A1:231714783-231715083|varpos=231714933

ATATTTAAATCTCAGTGCAGTTCATGAACAGCCAGCCTACAGCTCATGGGATCTATTTCACATTACCAAATTAGATTTGTAATATCTAAATTTTTTTTTNCNCAGTGGAGCTGTTGCTGCAGGAAGTAGATAAAGAGCAAGGGTCNTNN[TAAAAAAAAAAAAAAA]GGAGATAACTCTGGGGAGGGGTAAGTTTTTGAGGGAAGAAACAGCAATAGCAAAAATTGGTATAAGAATAACCTAAAAAGCTAAAGCACNCTTANGCCCAAGGGCTTCAACACTTATGATCTGAATTCAACTTTCAGCCC

>A1:233856313-233856613|varpos=233856463

GGANCTTTCATAGTTTTTAGAGGCAGTGGATCCTTTCTTGTGTTCCATCNTAGTTTGGGATTTTTAAAAAATGCAATCATTTCTTTAAAACCTGGAGTAGGAGAAACTCCATCTTGGTGTGGAAANNNNNTTNCTTTGTGTCTTTTTTN[TTTTCCC]CCTTTTTTCAGGTTTTTATTTAAATTCCAGTTAGTGAACATACACTGTAATATTGNTTTCAGGAGTAAAATTTGGTGATTCATCACTTACATACAACACTCAGTGTTCATCACACCAAGTGCTNTCNNTAATACCCATCATGCCTTTGGC

>A1:234524865-234525165|varpos=234525015

GCTCCTGNGGGCTCCAGTTCTTCTCCGATGGATCTGGGAGAATAACCAATCTCTCCTAACCACAGAAAGGCAGATGCCAGGATCNCCCTTTAAATAAAATAACTNNNNNNNNNNTCATCCTAATTCAATCCAGCAGANTTCAAACTCAC[GTTTTGTTTTTTTT]NNTTTTAGATTAGTTTGATTTATGAATTATAATAATTTCCTTTAAGCTTTAAATAATTTTACTGGTTGATCCTACTGTTGGAGAAACTGTTTTCGAAGAGCTCAGATTATGATGTTTCCTAATATACACTGCACACGTGATCAAAGAA

>A1:237323122-237323422|varpos=237323272

TGGAGTAGTGGGTTGACTTTGGCTTCAAGCGGTTGACTGTAAGATCTTCCCACACAGATTTTCAAGATNATGTGATCCCATGGATGGTCGATTGTTTTTACTGAACTATTCCATATGATNGTAATCAGGAAATTTTGAAAGTGATCTTA[CTTTTTTTTTTTTTTT]AAGAGTCCTTCCCCTAAATTAAACTTCAGAACAACAGAGTTTTGTCTTAAGCAAGNNNACTNTGTTGACAGCCAACTCTCCAGCTNTGCAAAATTAGGCATTTTGTTGTTTATTACATGGAAACAAAATGNGATTGGAGTAAATGCAC

>A2:4767896-4768196|varpos=4768046

TGTTAATCCATATGCATGGATGTTTTTCTGGACACTATNCTGTTCCATTGATTGATGTGTCTGTTTTCATGCCAATACCTNNCTGTTTTGTTGGCTATAGCTTTGTAATATAGTTTGAAATCAGGAAGTATGATGCCTGNAGCATTGTT[CTTTTTTTTTT]NTTTTCTTGAGATTTCTTTGGTTATGTGGGGTCATTTGTGGTTCCATACAAATTTTAGAATTATTTTTTATATTTCTTTGAAAAATACCATTGTAATTTTGATAGGGAGTGCACTGAATCTATAAATGGCTTTGGGTAATNTGGA

>A2:5250111-5250411|varpos=5250261

ACTTTGTTGAAAATTAGTTGACTATATGTATGGGTTGATNTGGGGACTCTCTGTTCTATTGGTGTNTCTGTTTTTATGCCAGTACCATAATGCTTTGAACACTATATTTTTTTAATACAATTTGAAATCAGAAAGTGTGATGNCTCCAG[CTTTTTTTTT]CTTTTTCTCTTTGCTCAGAATTGCTTTAGCTATTTGGGATATTTTGNNNNTAGGTCCATATGAATTTTAAAGTTGTTTTTTCTATCTGTGAAAAATGCCATTGGGATTTGGATAGAAATTGCATTNAATCTGTAGATGGNTTTGGGCAGAACGGGCTCTTT

>A2:6310136-6310436|varpos=6310286

CTTTTAGACAAGCTGTCCTCTNGCCGTGCCTCGTATGGCCTTTCCTTGGTGTGCACTCCTNGAGAGAGATCTCATGTTTTGAGATTCATGCACGTTATTGCGTTTACTGATAGCTTATCCCTTAAAAGAAAATGTTTATTTATTTATTT[TGAGAGAGAGAGAGAGAGAGAGAGAGAGAGAG]NACGTGTGTGCAAGTCGGGGAGGGGCAGAGAGAGAAGAANNCAGAGAGAGAGAGAATCCCAAGCAGCCTCCANACTNAGCACAGAGCCCGACGTGGGGCTTGATCCCACGAACTGTGAGATCGTGACCTGA

>A2:6654854-6655154|varpos=6655004

TGCACAGNGAAGGACACAATCAACAAAACTAAAAGGCAGCCTATGGAATGGNAGAAGACATTTGCAAATGGCATATCTGATAGAGAGCTAGTATCCAAAATATANAAAGAACTTATAAATCTCAACACCCCAAAAGCAAATAACCCAAT[TAAAAAAAAAAA]AAAAAANTGGTCAGGAAACATAAAGAGACACTTCTCCAAAAACGACATCCAGATGGCCAACTGATACATGAAAAAATGCTCAACTCACTCATCATCAGGGAAATGCAAATCAAAACTATAATGAGATGTCACCTCACACCTGTCAGAGTG

>A2:7077448-7077748|varpos=7077598

ACAAAATCCCAAGCAGACTCCAGGCTCNGAGCTGTCAGCACAGAGCCCGACGTGGGGNTCGAACTCACAAGCTGTGACATCATGACCTNAGCNAAGTCAGACACTCAACAGACTGAGCAACNCAGNNNCCCCCCCTTTTTTNAAAAAAA[ATTTTTTTTTTTT]TTTTTACTGTGCAAATNAAGTTTTACTAGACAAAACCACACACATTTGTTTAGTAAGGGGTATGGTTGCTTGTGCAACTACAAAGGCAGAGCTGAAGAATTCCAGGCNGGATGGCCAGCAAAGCAGAATGTACTCAATGCAAGAGGACTT

>A2:10894389-10894689|varpos=10894539

TTGAGTTTCCATTTTCTCACCTGTAAGACTGTAGATTATAGAAGCANTCTCTCACCAGGCTGCTATGAGGACTCATTGAGACAATATTGTATGCCATTCCTCCCCAGTGCTTGTTATATAGGAAGGTCTCCATAAATGGTGAGACTCAA[GTGTTTTT]TTTTTTNATGGAGGTGAAATTCACATAACATAANATTAGCCATTTTAAGTGAACAACTCAGTGGCAATTGGTGAACAGTGTTATGGAACCACCATCTGTATCTAATTCTAAAACATCNNCATCACCCCAAAAGGAGAGCCCATGTCNAT

>A2:13893862-13894162|varpos=13894012

GCTTCTCTTTATCCCTCATGTCTCAGCTCCTGGGCAAGGCCTTCCCTGATCACCACGCTTAACCGGATGTCACTTCCATCTCATTCTCAACCTCAGCACCTTGTTCATTTCTCTTATAAATTCTCATTCTTTATTACATTACANTTTTA[CAAAAAAAAAAAAA]CAAAAACAAAAAAAGTTAAAGCTTGGATTCTTGAGGGCAGGGGCTTCTGNGAGCCTCAGTTTCCTTAGCTGTAAAAATGGAATAATAGTACCCACCTCAAGGTTTGCTACAGAGATGGACCCCATGCTATGCCCAGTGGAGTG

>A2:17052968-17053268|varpos=17053118

CCTNAAAGTTCCAACTTAATCACATGGTTGATTTTCCTGNCAACTAGCCCCCATTCTTAGGTGCTCCCCAAAAGCACTTTGTTAACATAAACTCAGGTATGGTTGACTGTAGTTTGTTATCAATATCAAGACACCTTTCCATTCNNNNN[ATTTTATTTTTTTTT]NTTTAATTTACATCCAAGTTAGTTAGCATATANTGCAATAATTATTTCAGGAGTAGATTCCAGTGTTTCATCCCCTATGTATAACACCCAGTGCTCATCCCAACAAGTGTCTTCCTTAATGCCCCTTGCCCGTTTAGCCCATCTCCC

>A2:17677178-17677478|varpos=17677328

AGTTCTCTACACAGAAAAGAAACAATGAAAGAAGGAACTCTGAAACATCAGGGAAGGAAGGAAGAACACAGTAAGCAAAATTATGGGATAGAGATAAAAAGGCCAATCTACCAAGAAGACACAGCANTTCAAAATGTGTATGTACCAAA[CAAACAAACAAAAAAAAAAA]AAAANCCTACNAACTATGTATAGCAAAACTGATAGAATTGAAAGGAGAAACAAACAAACCCACAATTACAGTTGGAGATTTCAACTCCCTCTATCGACAAATTGGTAGAACAAAAAGACTGAAAATCACCAAGGATAAAGAAGAACT

>A2:18073163-18073463|varpos=18073313

TCTCTCTCTCTCTCAAAAANNNNNNNNNNTTAAAAAAAAAAAAGTCAGATATTTAACCAACTGAGCCACCCAGGCACTCCTGTATTTTTTTTAAAAGATTACAGCACTGACATATAGATCTTTCCAAGTCAGTACACGAGGTAAAAAAA[GAAAAAAAAAAAAAA]GAAAAAAAGAAAAACCAAAGCAGTAGTACAACATACACAAAGATCCATCTTGGAATCTGACNTCCTAAAGGCTTATAAAAGTATACTGTCCCCTCTCCCCCTTACAACTTCCCTAGGCTAGCCAAGACTGACTCACTACTCTCTCTT

>A2:18895333-18895633|varpos=18895483

GTTAAGCAGCCGGGTAGTGATCTCATGGTTCATGGGTTCAAGCCCCGCATGGGGCTCTGCACTGACTGTGCGGAGCCTGCTTGCGATTCTCTCTCCCTCTCTCTGCCCCTCCCCAACTTGNGTGCNCTCTCAAAATAAATATATNAACT[TAAAAAAAAAAAAAAA]GATATAAATGCTCAAGGCTCCACTCACAGCCTAGGGGGCACTGACACATTCCAGGGCATGGTCCCTCTGTTCTCTCCCTCCAGTGTGTGGCCTCTGCTGCATCTTGTGTGCTGCCTAATTCTTCTCCCTCTGCTC

>A2:20838758-20839058|varpos=20838908

CACAGCATTTTTTACAGAAACAGAAAAATCCATCCTGAAATTCACATGGTATCTCAAAGAGCCCCAAATAGCCAAAACTATCCTGAAAAGGAAGAACAAAGTTGGAAGACTGTATTAGGGTTTTTCAGAGAAACAAAACCAACAGGAGA[TACACACACACACACACACACACACACAC]NGTATGTTATAGGAATTGGCTCATGAGATTATAGAGGCCAAGAAGCCCAGGATCTGCCATCTGCCAGTCAGAGAACCAGAAAAGTCAGTGGTATGTATAATTCAGTATGAGTCTGAAGGCCCAAGAA

>A2:21393362-21393662|varpos=21393512

TCAGTTGCTANGTGTCCAACTTCGCAAAGCTCAGATGATGACCTTGAGGTTAGTGAGTTGGAGCCTCACGTCAGGCTCTCTGCTGTCAGTGCAGAGCCAGNGAGCCCGCTTCGGATCCTCTGTCCCCCTCTCTCTCTGCCCCTCCCCNN[GCTCTCTCTCTCTCTCTCTC]NNNCTCTCTCTCTCTCTCTTTCAAAAATAAGTAAAACATTAAAAGAAAGGGAGGAAGGGGGCNCCTGGGTGGCTCAGTNGGTTGAGCNTCTGACTTTGGCTCAGGTCATGATCTCNNGGTCTGTGAGTTCGAGCCCT

>A2:22256908-22257208|varpos=22257058

GATCTTGTGGTTCNTGAGTTTGAGCCCCATATCAGGCTCTGTGCTGACAGCTNGGAGCCTGTAGCCTTCTTNGNATTCTGTGTCTCNNNNNNNNNGCCCCTCCCCTGCTCNCACTGTGTCTCTCCCTCAAAATAAATAAGNATTAAAAA[AATTTTTTTTT]TTTTTTTAAATAAACATTAAAAAAAAGAAATTGGAATTCATCACAATTTAAAACTTTTTCTCTGTGAAAGATACCATTAAAAAGGTGAAAAGAAAACCATGCATTGGGNGAAAATATTTGCAATTCATGTGTATGACAAAAGACTTGTATC

>A2:22361922-22362222|varpos=22362072

TTCACCTGGAAAAAGAACTTTTCACCAGTGTAATTCTACTTAACTACCATTCTTTATGGTATTATCATGTATTGTACTTCTATGTGTTATAAATACCATAATAAATGTTATTTTTATTTTGTTTTATTTTTGGTTTAATCAGCTATTTC[CAAAAAAAAAAAAAAA]TTAAATAAGAAGAAAGATAGCAATATATTTACCCACACATTTTCCATTTCTGGCACTCTTCATTCCTTCCAATGGATTTGAGTTTCCATCTGTTATCATCTTNAGGCTGAAGAACCTCNTTTAGCNTTTTTAATNNNNNNNN

>A2:23410924-23411224|varpos=23411074

GCACCATGTTATGCAATGATAACCAGAGAACTAAGCCCTGTGTGCAGAAGTATTTTGAAAGGACATAAATCATTGTGATTAGACAAGATGAGACACTTCAGAGACCACNTCACCAAGCTTAATTGCAAATCAAATGTCATTCCAAAAAA[ATTTTTTTTTTTTTT]TTTTTTTTACAAAAGTATGTGATTTATTTGTGTAGGCAGTAATTATCTGCCTCAATCCAGAAAAGGATGTGAGGTAGTGAGGCATCTGAGGCAAAACTGAAAATGAAGCTGAACTNTTCCTTGGAATTCAGGCATAAAGCTCACCATGGC

>A2:24370826-24371126|varpos=24370976

TTCTTCGTTTTAAATGTAGAAAATCAAATCCTTCACGTTTGTTGTCTTCTAAATTATAAAATGTGTTCATTGCATAACTGTATTTTCCAAATAACCTAGATTTATTTNNNNTAAAAATGCTTATTTAAAACTTGTACATTATGTAGTACAACACTGT[GTATATATATATATATATATAT]ATATATATATATATTTTACAATAAANCGGACATTTTTTGTAAAGCTTAGTAAAGAAAAAAAAACATTTCTGCTCTNTCACAATCTAGGCATTTAAGTCCTGTAAAAACACTTTCCACTTACCTTACGACAGGTTTCTGACCTGTTCACA

>A2:24615708-24616008|varpos=24615858

TCCTGCTTTTGATAATTATATGGTATGTGAGGATGCTCTTGTTCTTACACTGAAGTATTCAAGGCCCAAGGGACATAATGTCTGTAACTCATTCTCAAATAGGAAGAGAAAAATAATTCATATTTATAGGTTATATAAATGTTTACTAT[ATGTGTGTGTGTGTGTGTGTGTGTGT]NTGTGTGTGTGTGTGTGTGTATTCTCCACTATTNATATATGGTGTATACATATATATAGTATATACTACACATACATACATATATATATACATANNNNNNNNCATANATATACATANATATATAATATACATATANATACANNTATATA

>A2:26085235-26085535|varpos=26085385

ATAAATAGGGTGCCTTTCCCANTGGGTTCTTGTGAGGAATAAGTGGGCACAGGTTGAGGCTCAAGACATATTNGGTNGTCTTCTTTTTTTGAAAAACTTTGTTTTAATTATTTACTGATTTTTGAGAGAGAGANNNNNNNNNNNNNNNN[CAGAGAGAGAGAGAGAGAGAGAGAGAGAGA]ATCCTAAGCAGGCTCTGTGCTGTGAGCATAGTCTGTGCAGAGCCTGATGTGGGGCTCGAAACCACGAACCATGAGATCATGACCTGAGCTGAGATCAAGGGTCAACCGCTTAACCNACTGAGCCACCCTGGCA

>A2:26868253-26868553|varpos=26868403

CAAAATTGGCATAAGACATTTTTAAGAAATGAAACACCACAGATCTGTTAAATTTCCATGTACACCACTCACCANTCCCATCCCCTCCCNCCTTCCAGAGGGAACANTGTTCCTGTGTTTGGTTGTGTTAATCACCTGTATTNNNNNNN[AAAATTTTTTTTT]TTTTTTTTACTAATTCTGTACCCTTAAAGAATATATAGAATAGACATTTTTATAACTGTATAAATGGTGTTATGCTATGGATAGGTACATATCATTCTGCNTTATTTTTCACTCAGCATAATGTGTTTATTTTTCCATATTGATACAGAA

>A2:28235081-28235381|varpos=28235231

TTCAAGTCTNTACACTGATAAGAACANGGAAGCAAATCACAAGGATGTGAATGCCCAGGTTTTTCCACAAAAGCTGACCTGTACACATGGAAGGGTAAAANAGNACAAAAACAATCCTACNGCAATACAAGNAAGGAGAACAACCGAAA[ATAATAATGTGTGTGTGTGTGTGTG]TGTGTGTGTGTGTGTGTGTGTGTGTGTGTNTGNGNGNGNGCTTTAATTATTCTGAATGTTNGTTGAAGGGAAACAAGTTTAATTAGAGGNTGAGCTGATAAAAACAGTTATGAAGATTCTTAAGGGAACTTCTAAACACATTCAAGAAT

>A2:28689067-28689367|varpos=28689217

GGTAACTGTAAACATGTTCCCAGTTGCTGAGCCAATAAATAGAAATACCCTTTCTAGAGAGAGAAACNTGCACTATCACATAGTAAATTCTAAGGAAGAAAAGAGACAGTGCAAGATAGGAGTTTCCCAACATGGCTTTTNAGAATTAA[AAGAGAGAGAGAGAGAGAGAGAGAGAGA]TTCAGGACACCAACCTTGACTTATTAGGTCAGAATGGGGAAGANATAATAACAGAAACTGAGTTTTTTTAAAAAGCACCACAGGGGTTGTGATCAATAAGGTTGGGGAGNCTCCATTTGCTAA

>A2:28787783-28788083|varpos=28787933

TTTAAGAACCAAGTACATGAAAGTTAACTCATGCCATTTCTTTTTTCTGATTCGGCCCTTTAACCATATTATACTGCTATCTTATAGGTTGTTCCTNGCTTTGCTGNTTTTTTTTTTTTTTAATGTTTATTTATTTATTTTGAGAGNGAGAGA[GAGAGACAGACAGACAGACAGACAGACAGA]CAGACAGACAGACAGAATTCTTAGCANGCTCCTTGNTGTCAGCGCAGAGCCCAGTGTGGGGCTCAAACTCAGGAACCATCTCAGGAACTGTGAGATCATGACCTGANNNGCTGAAATTAAGAGTCAGGCCCTTTACTGACTGAGCTACCCAGGTG

>A2:30930943-30931243|varpos=30931093

AATAAACATTTTAGGTCTTANAAATAAACATTTAGGTCTTATGGGCTGAAGGAACAACCTACATCTGTATAGCCTTTAGCGTAGAAGTTAAACTATGTTTCTAGCAGTAAAATGTGCAAATGCAGTGTGATTTTTTATTCAGAGCGTTT[TTACACACACACACA]CACACACACANANATATATATATACACTCATATGTATCAAATATACATGTATCAAATATATATACATATATATTCCTATTTTGTTTAATAACCATTTGAAGTCAGAAAAACGTATTTTCCCTATTTTTCATCTAAGCATATGGTTTTAGG

>A2:30964875-30965175|varpos=30965025

TTTCCACCATTTTTGGGGTTGTTTTGTTTCTACTACTTCTTAGATAAAGACAGAAAGCATGCATCATTTCACTTTTTGAAACTTATGTTAGCATGCAATCCAGGAAAATTTTACAGAGTTATTTCTAAATCTTTTTAAAATAAAAAAAT[CGTGTGTGTGTGTGTGTGTGTGTGTGT]GTGTGTGTGTATTTTTAACGCTTGTAAATTTTCAACTTAAACCCTTTCTTATAAGAGCCCTGCGTTAAGTTTAGATTGCAATAATATTTCCTTATTAATTTTTACTCTTATTTAAACATTTTTGTTGGACTTTGTTTCTTTAGCAGAGAA

>A2:31979095-31979395|varpos=31979245

TATAATTTTAAATCTTCTAGGAGTCACAGTTTAAAAACAAACAGGTGAAATTAATCATAATATACTATTCTATTTAGCCAAATATGTAAAAAATGTGTAATTCCAACATATAATCAATATAAAAACTACTAATGGGATATTTTAATATT[CTGTGTGTGTGTGTGTGTGTGTGTGTGT]NNNNNNNNNNNNNNNNNNNNNNNNNNNNNNNNNNNNNNCCTAAGTCTTAAAAATCNGGTATGTCTTTTACACTTAGAGTATATTTCTATTTGGACTAGCCCCNGTTCAAGTGCNCAGTAGTAACACGTGGCTTGTGGCTACTAGAGTGG

>A2:32761094-32761394|varpos=32761244

CTCANGGTTCATGGGTTCNAGCCCCGCATCGAGCTCTGNGCTGACNGTTCAGAGCCTGNAGCCTGCTTGGGATTCTGTGTCTCCCTCTCTCTCTGACCCTCGCCCACTCACACCCTGTCTCTCTCTCTCAAAAATAAACACNNAAAAAA[AAAATTTTTTTT]TTTTTTAATTGTAATACTATTTAAACACACACACACANNNNNNNNNNNTACAAGATCTCTTTGTCAAACCTACTAANTTATAAACCGGACGTCAGTTTTGTAAAGACTTAATTTTAAATTATAATAGNACACATCTGGGGTGATTTTTGACTCTCCACAAATA

>A2:33825928-33826228|varpos=33826078

NTCACTAGATTATAAAGCATGGATNGATCCAGGATGGTCTGGTCTTAAGTATGAATGAAGGCTTNAATGACTTTACTAATCCATTGTTTTTTCCCTTGGANTGAAGGTGTGACTTTTNNCATAATTGAATTTTTCNGCTTTGTTTAATTAGGG[TAAAAAAAAAA]NAAATGCCCTGTCTGAAGCATCAGCAAGCTGATGGGGAATTTATTTGGGTGCTTCTCCCTTGTAGCCCTTCACTCAAATGACANCTCATTGATACATGCCAATNAGGACTAGCAACTCTTGGACCAAGAACTCTCTNTGCAAGGA

>A2:34920854-34921154|varpos=34921004

CACGCTCCACGGAGAGAAAATCTGGTACTTAGAGCAATACNTTGGGAATCATTTAGACATCNGGAGCTAAGTCTTTATCTCTCACTAGGTTTCCAATTTTCTTCATATTGCTCAGGCTCAGTGAGTTAACTTTAAACTGAGTGCTCCCA[GAAAAAAAAAAAA]TAATAGTCTTTTACTCAAAGCTGATTTCTGGAAANAGATAGTCTCCCTAGATTAAGTGAAAATGTGTAGCTTTTGTGCTTTGTCACTTAAACCTTGACTTTGACATCAGAATAAATCATGCTAATCAAAACTCTGAAAAGTGT

>A2:35238099-35238399|varpos=35238249

TTTTTTGGCAGGATTGACATTTAGTTTTATTTTCCTTGAGCCATTTCTGCTGAATTATGACCATATGGTTATATATACNTATATATGGTTATATANNNNTATATATGGNNNNNNNNNNNCNTATATATGGTTATAGNTAGATAGATANN[TAGATAGATAGA]NGGTCAAACATATGGTTTNACACAAATTCATTGATCTATTTATTAAGTGAATGGATAGGCTTATGCTTTCCACATCATTTCATAAATAACAACTGTNCTTTTTCTGACCCTTCACAGATTTGAGTCAGCACACTCTTGATGAGGTAGGA

>A2:36785453-36785753|varpos=36785603

TAGGTGCATGTTGACACAACACTGTCACTTGCTCCTGGATTCCTCAAATTTCCAAACATTGTTTCATTTTGTTTGGGGTTGGTTGTGAAACAGAACGATACATAGGCTCCGCATTTGTCCCTTCGACCAGCGCATGATCATTTTTNNAA[GTTTTTTTTTTTTTT]AATTTAAACGTTTATTTATTTTTGAGAGAGAGCCAGTGGGAGAGGGGGNNNNCTGAGAGAGAGCGAAACAGAGAAACTCAAGCGGGTTCCACTCTGTCAGTACAGAGCCTGAAGTGGGGCTTGAACTCACAAAACACGAGATCATGAC

>A2:37677189-37677489|varpos=37677339

GTGATAATAAAAGGGGTTTGTGNTCAGGTCNACTGTTCTGTACGTACCCAATGGANTCAACGGGTGGGAAATCCACGGGAGAAGCATTACAAGAAGCAAAGATGAAAAACAACAACCCGATAATAATTATAATAAAGCTCCAATGACGA[TAAAAAAAAAAAA]CANNAACAAACAAACAAACAAAAAACCTGGCAGCAACCTGGGTTTCAAGTTCCTTCTGTGACGACTGNGATAGCAGAGCTGATGGTGTTCTGGCTCCCAANGCAAAACCTGTAACAGCGCTTCAAGAGGGAANAATGTGTTT

>A2:38521679-38521979|varpos=38521829

CTCNCAGTNNGTGANTTCNANCCCCNNGTCNNGCTCTGTGCTGACAGCTCAGANCCTGGAGCNTGCTTCGGATTCTNTGTCTCCCTCTCTCTCTGTNNCTCCCCTGCTCNCACNNNCTCTCTCTCAAAAATAAATAAAGGTNAAAAAAA[ATTTTTTTTTT]TTTTTTTAATTCCTTTCTCNTCTGCTAGGAGAGTCAGTCTCTGTAGCTTGCAACCACTAATGCTTACAGACANGCAGAATCCCAATATTTAAAAGGAATAAAAGTGGATATGGTCTGCTGAGACGTAGAAGAGTGGNCAGGGGCTTCTCC

>A2:39101634-39101934|varpos=39101784

ACTTAACAGANTACCTTCTAGGGGCACCTGGGTGGTTCAGTCAGTCAAGTGTCCCACTCTTGGTTTNGGCTCAGGTCATGATTTCAAGATTTGTGAGTTTGAGCCCCATGCAAGGCTCTGNGATGACATTTTTTAATGGGATTTTTTTT[TTTAAA]AATTGTTGAGTTTTAANGGTTCTTTGTATATTTTGGATAACAGTCCATGATCTATTTTGTCTATTGNGATTATTTTCTTCCAGTCTGTAGTTTGTCTTCCAGTTCTCTTGAGATTGTCTTTCNCAGAGCAGAAGTTTTTTATTATAATGA

>A2:40663495-40663795|varpos=40663645

TCGAGCCCCACATCGGGCTTTGTACTGACCGCTCAGAGCATGGAGCCTGCTTCAGATTCTNTGTCTCCCTCTCTCTCTGTTCCTCCCCCTCTCNTGCTNNNNCTCTCTCTGTCTCTCAAAAATCAATAAANGTTAAAAAANNNNNNNNN[TTAAA]AATTACAGAAGGTATTTTAAAAACCTATAAAANAACACTTGTTAATGAAAACCTTATATGGAAATGAAAAATCCAGGAGTTCTGCTTCAAAAACTTAAGGCTGATATGAAAACACATGCAGATTGCTATCTGCCTTGAAAACTCCCTNGG

>A2:40664689-40664989|varpos=40664839

TTTTTTTCCAACATTAAGTTTGTTGTTTTTNNNNCTTCTTTTTAATCAATAAATATCCACAACGTAGCATGACCAAGATGCANNCTGTTTTACACTCAGTAGGTATTTTTCAAAACAGACTGNTGTGTAAACATTTCACTATTGCTAGTTTTTT[TTTTAATA]ATATTTATTTATTTTTGAGAGAGGGAGACAGAGAGCGTGAGTGGGGGAATGGCAAAGAGAGAGGAAGAGAATAGATCAGAAGCAGGNTTATGCTGACAACAGGNGGCCTGATGTCGGGCTTGAGCTCAAAAACTGAGAGATCATGACCAG

>A2:40933937-40934237|varpos=40934087

ATACTAACAAGCTGTGCTATAATATGTAAAAGACAAAAANNNCACACACACACACACANATTGGCTAGCAAAGGTCATTTGCTTTAGGGATGCAAATCACTGTGAAGCTGGAAGCCAAGTGAAGGTGATAGAAGGAATGAAAGGGGGAAAAAAA[AAACCC]CCGAAAACATACTAGGATACCCAGCCAGAAGGAAGGTNTTCTTCCTCTCTTGCTGAAGGAGAGATGAGAAATAAAAGCCTTACTATTCTACTTTCTTGCCTACATTCCTCTGATTAGAAGAAGCTGNGTCTTGGAGGGACTAAAATTTAC

>A2:40970397-40970697|varpos=40970547

TGACTCTTATAATTATACACTCAATCATTTTGACTTTTTGTTTTNTATTGGTCTCATGTACTAGATTTTGTGTTCTGTGAAAACAGAGATCATGGCTCCTTTCTCTTGCCATTTTATCTCAAACACTGCACATNCTGGGCACATACTGG[ATTTATTTTATTTTATT]NTATTTTATTTTATTNNNNNTTATTTTATTTTATTTTATTTCNNNNNANAGAGAGAGTAGGAGAGGGGCAGAGGNAGAGAGAGAATCCCAAGCAGGCTCTGCACTGTCAGCGTGAAGCCTGACACAGGGCTTGAACTCACAAACTGTGA

>A2:41115878-41116178|varpos=41116028

CTTTATGATAAAATGGTCCCTTATCTCTCAGTATTACTTCAGTGGTATTCTTATTGATAATTACCCACCATATTAATAAGTAACATTTATATTTTAATACATGGAAATATATTCTCAGAACCAGGATATCACTAATTNTTTCATGCTCT[TAAAAAAAAAAAAAAA]CTGTTACTATCCACCTTGATTTGCCACATTTCAGTAAACTTTCAGAGAAGCAGTCTNCTATTTTGGACTAGTATTGAAAGCTATCCAGTTGTACCCCACTTACTCATGATTTTAAATTTAAGTTGTGTACCATCATTA

>A2:41582749-41583049|varpos=41582899

AAGTATTTTGCTTTGCTCTTTCAAGATCTATCTTNTCTATAACAAGGAGTTTCATAGGTACTGTTTCCNGATTTTACATTTTTGCATGTAATGTAATTATAAGATTTAGTTACAGTGTGGGCATTAGTGTTTTAATTTTTTTAATNTTT[ATTTATTTATTTATTTTTT]TTTTTGAGAGAGAGAGAGAGAGANCNAGCATGAGTGGGGGAGGAGCAGAGAGAGAAGGAGACACAGAATCTGAGGCAGGCTCCAGGCGCTGAGCTGTCAGCACAGAGCCTGACGTAGGGCTCGAACTCACAAACTGTGAGATCATGGCCT

>A2:42053422-42053722|varpos=42053572

TAAAAGGGCCACTTTGCCCATTAAATAGTGAATTTACTAAAATGGCAAGATNGGAGGCAAGAAGACTATNTAGGTGCCTCTTTGAAGCAAAATAAGGGATGTTGATCACTTAAAACAGGTATAATAATGGTGATGAAATGGTGAAAAAT[TAGAGAGAGAGAGAGAGAGAG]CACAATTGGAGAAGGGGCACAGAAAAANGGAAACACAGAATCTGAAGCAGGCTCCAGGCTCNAAGCTGTCAGCACAGAGCCCAATGTGGGACTTGAACNCACAAACTAGGAGATCATAACCTGAGCTGAAATCAGTCACT

>A2:42440795-42441095|varpos=42440945

GTTAGAACGAAATTGTCATGAGAGGTTATGTGTATNTGGACATTTTTATCTCCAAGTTAATTACACACATGTAATGAAACCTACACAGTTCATAAAGTAGAAAATAAATAAATAANTAGCATCTACAGCATTTAGTGATGTAAATCCAG[TGTGCGCGCGCGCG]CANGCACACACACACACACANNNNNNNNCACANAATATAATAAAATAGATTTGTACTCGCATAGGAAGCGCACATAGTGAGAAAGAGCACGGGCTTCTCATTTTANCAGCTGTGTGGACTTGGAGANGTCAGAGCCTTTGGAACATCAG

>A2:44565731-44566031|varpos=44565881

AACCATCACATAAGATTCCATCAGGTTCTCACCTGCACCTATGAATAATCTCCTCTTCCATTTCTGTCCTTGCCAGAAAGAGAACATCTTGACCCAACTTGGTGGGGATTGTGTTTTGAAAATCACCCTGTCTCTTTTTTCTTTTTTTT[TTTTA]AACCATCAATAAATAATCATAGAATTAAATATATAAAACTTAACCTAAGTGCTATGAAAAGATAGGGGGTTTTGACAGAAATAACAAGACAGTGGCTAAAGAGCTATAAGAAAGGCAGTGGCACTNAATTAAGTCGGAGAGCCCTGCATT

>A2:44843189-44843489|varpos=44843339

GGGTTGTGAGCATTAAGTAATTNATGTGAAGTGCTCCATGCCTTGCACATTGCATAGTAAGAGCTAAGTGCTGTTAGTAAGTACTAACTGCTATTATTCTCATGACTACTACCCAGCCAGAATGACCCCGCAATGAACATTAAATGTAA[AAGAGAGAGAGAGAGAGAGAGAGAGAGA]AGTGTTTATATGGCTAGAAAATACTTCATTATTTGGAAAGGATGTTTCCTGTCTTTCTTGGCCTAACAATATAATTCCCTTTGCTTACTATTTATATTTTGTACCCAGAGTAACTAAGATTGAAATGTGTTGC

>A2:44883430-44883730|varpos=44883580

GCCTACTTTTAGAGTTTGTCTANTGTGTTCTTCTCTGACATTCTCACATGGCTCCTTCCATTACCTCTTTCAGGTACCTGCCCAAATGTCACTTTATCAGTGAGGCCTCTTTGATCAAGATACTTAATATAGTCACTCCACATCCAGTT[ACCACCCCCCCC]CCCCTTTCCTGCTTTGTTTTTCTTAATTTCTCCATTTTCAAAAATACTATGTTTCATATACTCATTTATTTGNTGTCTATCTCCTCAAATAAAATTTAAGGTCCATCAAGTTAGAGACTTCAGTCTGTTTTGTTTTCACTGTTATATC

>A2:45009790-45010090|varpos=45009940

AGGTGGCTCAGTCTGCTATGCATCTGACTTGATTTTGGTCTCATGGTTCATAAAATCAAGTTCCATGTGCTGCTCTGCGCTGACTGCANGGAGCCTGTTTGGGATTCTGTCTCTCTGCTCCTCCCCCATGNTTTCCTCTCTCACACACN[TTCTCTCTCTCTCTCTCTCTCTCTCTCTCTCTCTCTCTCTC]AAAATAAATAANAAATAAANNNNNNNNNNAATGAGGTAATAGAGCTAAGAGGAAGGGGCCAAACTGAACAGGGGTCAAACTTAAGGCCTCTGATCTGGAATTTGGATTTTATGCTCATAT

>A2:45145302-45145602|varpos=45145452

AACTTCCTCTGGACTTCANATTGCCAAAATAATTTTTTTNNNNNNAATTTAACAGTGCTGTCTGAACATGCACCTGGACCTTAACATGTACCTCCTTGTGATATCCCAATGTAGCTATCTTAAATTGANATTGATCTTNACACTCATGATAAAAAN[AAATTTTTTTTTTT]TTTTTTTAATGTACATGTTTTCTCTCTCCAGAAAACATAGGGCAGAATTCTGGACAGTAGGTCTTCAAAAAAACATTCATTTAATGAAAATACAATAGGCATGGTCTGGGACTATGTGCATAATAGGTAAAAGAGCAGGCTGGAAGATAA

>A2:45268030-45268330|varpos=45268180

AAAAATGGGGNNGGGGGNAATGGGACACAGATCATGAACTCAGATTTCTTCACTGTCNTTTCNTTCCTGCACCATTTTAAAGACCCCAAACTGGAATTGAGAGTAAGCATGACANGGANTCTAATCAGTATACCCCACACCACCCCCAG[GACATACACACACACACACACA]NNCACACACACACAGACTTTGGCCCTGTATCAAAAGACATACAAATAAATGTACCAAAATTACTTGTTAAAATGTTTCNCNNNNNNNNNNNNTTTTCTATATCTCTTCCATNTATATGTAATTTTTATGACTTTAATAAGAGAGTTTTG

>A2:45813852-45814152|varpos=45814002

CTTTANAAAGATGTCAATTCTCCCTAAATTATCTCTAAATGTAAACATATATATCGATGTTAAAAAGTTTTTAGGAAATGATATACACTTACNATAACATTAATATGAAGAAATAAACATGCCAAAGTATGCAGGAAAATCCNNNNNNN[CCCCAAAAAAAAA]AAAAAACAAGGGTTAACCTAGCTGCATTAAGTACTAAAATGTAAAGCTGTAATAATTGAAAAGGCATGCTACATGCATAGACAGTAGCTCGATGGAACAGAAATGCACTTAAATACAAAAGAAATTTAGTATATGATAAAATGATATCAT

>A2:47059963-47060263|varpos=47060113

GGTACACTTGTAGTGAACAAAGCATAACATATATAGAAGTTGAATCACTATGCTGTACTCCTGAAACTAATGTAACATTGTGTGTCAACTATACTCAAATAAAAACAAACTTTAAATAGAAAATAAAATTGAATAAAGGGAAAGAAGAG[GAAAAAAAAAAAAAAAA]CTGATGAGATCTCNGTGGATGAGAGCAGAGTAAGGAAGGAGCAAAGAAATAATNACCAAAACTTGGGAAATCCTTATTTCTGTGGTTGGAGGTAGAAGTGAAAGCCAGCAAAGGAGCTGGGAGACATTACTAAAAGGAAGAG

>A2:47331436-47331736|varpos=47331586

AGGTATATATNCCAAAGAATTCAAAANNNCAGTATCTTGAAGAGATCTTGGCATATCAGTGTTTAGCAGCATTATTCATAATAGCNNNNNNGTAGAAACAACCCAGATGCCCATTAACCGNTAAATAACAAAAATANCGTGTATGTGTATATA[CACATACATACATACATACATACATACATACATACATACA]GTGGGNTATTAGGCAGCCCTGAAAAAGAAGGAATTCTTATTATATGCCACAATGTGGGTGAACCTAGAGCACATTATGATAAGCAAAATAATCTAGCCTCAAGAGGACAAATACTGCATAATTCCACTTGT

>A2:47561542-47561842|varpos=47561692

CCTGGGNNTGGCTTAGTCAGTTGAGTGTCTGACTTTGGCTTAGGTCATGATCTCANGGTTTGTGAGTTTGAGCCCTACATTGGGCTCGCTGCTGTCAGTGCATAGTCTGCTTTAGATTCTATGCCCCCCTCTATCTCTGCCCTTCCCCTGCN[TTCTCTCTCTCTCTCTCTCTCTCTCTCTCTCTCTC]NNNNNNNNNGAAAAAAAGAAAAAGAATCNAGACTTGGAAGTTAAAAAGAAAGCTTCCTGGNAGTGTAATTTGTAGGTCATAACTATATTAAGGCTTAGGAAGAGNATTCTGTTAAAAGGCATTAAATA

>A2:48533167-48533467|varpos=48533317

ATGTTTGAAGTCTCAACTCCAGGTAACTGTGGATGAGACCGTATTTGGAAATAGTGTTTGCCTATGTACTCAGGTCAACATGAAATCATTATAATGGNCTCTCATCCAATATGATTACAATACCTACAGGGAGGGGACATCTGGACACA[GACACACACACACACACACACACACACACACACACACA]GAAGAAAACCATATGAAGACATAGAAATAGAGAAAAGACACTAATGTAAAGAAAGGGGCAGAGATTGAGGTGTACTGCTACAAATCAGGGAATATCCAGGGCTACCAGAAGTC

>A2:50200549-50200849|varpos=50200699

GTCATGATCTCACAGTTCATGAGTTTAAGCCCCATGTCAGGCTCTGTGCTGACAGCTCAGAGCCTGGATCCTGCTTTGGATTCTGTGTCTCCCTTCTCTGACCCTCCCCTGCTCACANNNNNNNNGTCTCTCTCAAAAATGAATAAACT[TAAAAAAAAAAAAAA]NNNNNNGAATCTTCAGATCTTTAAGCATGGATATTTTTAAGTCTTTTGTCATTTGCACTCTAATGCTTTTGCAGAAGTGAGTTTCATGGAAANGACCCCACCAAGTATTCTTGACNATTGACACCACTGCCAAATTATTGTTAGAAATA

>A2:52677578-52677878|varpos=52677728

AGATCTCCGGGCCCTGGCAAAACATTTGTATGACTCATACATAAAGTCCTTCCCGCTGACCAAAGCAAAGGCGAGGGCGATCTTGACAGGAAAGACAACAGACAAATCAGTTAGTTCTCTTCTGCTGTCTACATTGGGGTGGCTGGGTT[CCTTGTTGTTGTTGTTGTTGTT]GTTGTTGTTGTTGTTGTTGTTTTGTCCTTGAGAAAATAATTCACCCTGTTACACACACATATACAGAGNNTCAATGTTCAGCTGTTGGCTGTTACATGTTGCTGGNTGTGTCTGAAACACAGGAAAGGAAATGTTTNCAGATAGGATAAGTAAA

>A2:52896671-52896971|varpos=52896821

AGTTGAACTTGAATGAAGCCCAGGAATCTGTATTTTAGCCGTCAGTTGATTGTAATGGTAATTAATGGTCCTCAGCTANGGTCCAAGCACCCATCTAATAACAAGGAGTGGCAAAGAGACACATATTTACAAAATAAATGACAAGAAAA[AAAATTTTTTT]TTTTTTAACTTTATTTATTGTTNNNNNNNNNNNNNNNNNTGNGAGCAGGGGAGGGGCAGAGAGNNNNNNNNNNNNNNGGAGACACAGAATCTGAAGCAGGCTCCAGGCTCTGACTTGTCAGCAGAGCCCGACGCAGGGCTTGAACCCATGAACCGT

>A2:54141614-54141914|varpos=54141764

AGCCATAAAAAAGAAGTATATTTTGTCATTTGAACCAGATGGATGGACCNTTGAGAACATTATACTAAATGAGATAAGTCAGACAGGAAAAGACAAATACTGCATGATCTCACTNANATGTGGAATCTAACCCCCTTCCCTAAGGAAAAAA[AAAACAACAACAA]ACAAAGACAAATATAAGCTTATAGATACAGAGAACAGATTGGTGGTTGACAGAAGTGAGGGTAGGGGGTGGACAAAATGGGTGACAGGAATCGAAAGGTATTTAAACTTCCACTTATAAAATAAGTAAGTCATGGGGATGTAATGTAAA

>A2:54773549-54773849|varpos=54773699

GGGATGGGCTAAATGGGTGATGGGCATTAAGGAGGGCACTTGTTGGGACGAGCACTGGGTGTTATATGTAAGTGATGAATCACTGGGTTCTACTCCTGAAACCAAGACCACACTGTATATAAACTAACTTGAATTTAAATTTAGAAAAA[AAAAAAAAAAAAAAGGAAGAAGAA]GAAGAAGAAAGAAGGGAGGGAAAGAAAAGATTTTTTAAAAAAATCCACAGCCTGAAAACAACCTAAATATCTAGTACCATGGGGAGTTGCGTAAATGTTGCCTTCTTTTCCTGCTGGGATATTCCAAAGCNGGTGTNNGGAAAATGCA

>A2:56084263-56084563|varpos=56084413

TGTGAAGTGGTATCTCACTGTTGGCTTAATTTATATTTCCCTAATGACTGNTGATGTGCATTTTGCGTATCTTTGAAGAAATGTTTATGCAGGTCCTGTGCCCCNNTTTTGAATTGTGTTGTTTGTTGCNNNNNNNNGTGTGTGTGTGTGTGT[GTGCGTGTTT]TAAGTAAGCTCCATGCCCAACATGGGGCTTGAATTCATGACTGTGAGATCAAGAGTCGCAGCTCTTACAGACTGAGCCAGCAAGATGTAGGAGTTCTTCAAATATTCTGAATATTAANGTCTCATCAGATGTATGATTTACAGGTATTTT

>A2:64124654-64124954|varpos=64124804

CTCTTAGTTTTGGCTCAGGTTATGATTTCATAGTTCGTGGGTTCAAGCCCCACATCAGGCTCTGTGCTGACAGTGTGGAGCCTGCTTGGGATTCTCTTNCCCTCTCTCTTTGCCCNNNNNNNGCCCCGTGCTTGTGTGTGTGAGCTCGC[GCGCTCTCTC]ACTCTCTCTCTCCCANGTGCAAAAATAAATAAATAAATAAATAAATAAATNNNNNNNNNNNNNNNNNNNNNNNNNNNNNNNNNNNNCAACATTTNAAAAAATAAAAAGAATATCAGATACTTGGNTGACAGTATGGAAAATGGCAATATTGGGTGCTTCACAAAATCAGTTCTCCCT

>A2:66066385-66066685|varpos=66066535

CAGTGGGGCAAACAGGAAGTGCACCGACAGAGGTTTACAGAGACCAATTTAAGATGGCGGAGNNCTCTGAGCACGGATGGTGTATGTTAAACCACAAAAATTNACCAGATAATTGTCAATCCAGGACATCTCAGAATAACAATCTTGAT[CTTTTCTTTTTTTTTT]NNNNTTTTTCCAATGTGTAAATTGGGTAAGAAATATCTTCTTACTCGTTATGGATCGTAATCCTCATCCTTCTCATTTCTCCTTATTCTAAATGCCCATACAATTAGAGTTGATCTCTGTTTGGCAGTTTCCCTCTTACAGATCTAGCGT

>A2:67835996-67836296|varpos=67836146

CTCAATTCTTTCCATAAAACAATACATTTAAAGAAATATTTAGGAATAAACCTAGCCAAGGAGAATAAAGCCTTATACATTGTAAANGATAAAATATTGCTGAATGGAGTTTTAAAAGACACAAATAAATGGAAAGNCATCTTTTTTTT[TTTTTTTA]AATGGATCTGAAGACTTAACCATTAACATGACATTGTTACTCAAAGTGAGCTACAGATTCCATGCAATTCCTATCAAAATCCCAATAGCCACTTTTAAAGGCTGGNNCCTGGGTGCCTCAGTCAGTTTTAAAGGCTGGCACCTGGGTCAC

>A2:68610428-68610728|varpos=68610578

CAGGCTCGGAGNTGGCAGCACAGAGCCCGNCGTNGGGCTCGAACTCACAAACCGGGAGATCATGACCTNANCCNAAGTCGGTCGCTCAACCGACTGAGCCNNNNAGGCGCCCNTAGGATAAAGAAATTTAATCCCTANCATACACAATT[TAAAAAAAAA]AAAAAACAAATGAAGTATAGACTAATTAATGACTTAAATATATGACCAAAATTGTAATTTAAGAAGAAAACATCAGTATGACCTATGATGAAAAAATCAGAGAATACCTACAGATGAATTTTACATAGATAAGAAACAAAATAACCCATA

>A2:68901078-68901378|varpos=68901228

ATAATTAACATGCTGTTATATTCGTCTCAGGTGTACAATATAATGATTCAATAATTCTACACATTGCTTGGTATTCATCGAGATAAGTATACTCTTAATCTCCTCTCTGTATTTCACTCATCCCCTTTGTTCTGTGTAATTAAGAGTCT[GTTTTTTT]NNNNTTTGTTTGTTTGTTTACTNGTCTGTCTCTTTTTTCTTTGTTTGTTTTGTTTCCTAATTTTCACAAATGAGTGAAATATGGNACTTGTCTTTCTCTGGCAGATCCATTCTGCTTAGCATAATACACTCTAGGTCCATCTAGATTGTT

>A2:69885889-69886189|varpos=69886039

CTTCTCCAGCCCAATTTCAGTGTAAATGGCCCCTGTCATGCCTTGTTGTTGNCATTGAGCTAGGTCCCATTTCTCCTTTTGGTTACNCATTGCTTTGTTCCCTTTGCAAAAGCCTCAATAACGGTACATNATTGGAATCCTATTGCCTT[CGTTGTTGTTGTTGTTGTTGTTGTTGTTGTTGTTGTTGTTGTT]GTTGTTGTTGTTGNTGTTGTTTTATTGCCTAGGCTAAAATTCTACACGAGTTCTAGCAGCTTTCCAGGTNACCAATTTTTCTTGGGCATTGACCTNGGAGTACTGGAAGTTTTTCTGTCCTGGGTAANTCTACCTTTGAGCTTTCCTGCT

>A2:70163960-70164260|varpos=70164110

CGTNGAAAATCCCTCCCATCTAGNNCTGTACCACAGTTTTATTTCTAAGATTTTAATTGATTTTTCTTTATATCTCNCTGTTTTTAGCTCTTCTGCTTTTTTAAACATATTATTGTTCACATTTTTATANGAGCTATTTCTTTCTGTAT[CTTTTTTTTTTTT]NGGCATTATCATGGTAATTATTGCAAATATTTGTCCCACTATTTCAAGTCAATGTATCTGCAGTACATTTACNNNNNNNNNNNNNNNNNNCTATTTTGTTTAACATTTAAATTTGCTCTTTTGTTTCTTAGTTTTGTCTTATGT

>A2:70362967-70363267|varpos=70363117

TGTTTCTATACTTTTTATTTTTCCAAAATGCCATATAAATGGAGTTGTAATTTGTAGCTTTTTAAGTCTGGCTTCTTTAACTTTGTACAAATCATTTGAGATTCACCCCTTTGTTNTGTGTCGCAGTGATTCATTCCTTTTTTCTGTTTT[GTTTTGTTTTTTTTTTTTT]NNTTTTTTCTTGTTGTTGGTAAATAGTATTCTATTGTAAAGATGCAACAATTTCTTTATTCAATNAANGATTTCATTTAATTCTATGNTTTATTTTAAGTTAATTTCTAAATAAGGTTGAAGTACTTTTTTTCCCTTGAATTGTTTCC

>A2:71537402-71537702|varpos=71537552

GTGCTTACTAAATTTTCTGCCATGAACATACATATGTATTTTAGTATATAAATTCCAGATCTCTGATGTATGATTAGGACTTTCAATGTTAAGTCTAAANNGTATTTTTTTAAGATCAGAAAATGTTTTTATCTTTTATTTCATTGGTA[ATTTTTTTTTTTTTT]GGAATATCCTATTGTTGCTAGATTTGATCTNTAGATNTTTTTTATTGTCTATTTTCATCTCTTATTTCNACTGGGTTTCAGGTGAATTTTCTTAGTTTGTTTTCTAACTCAGTGAGTGCCTTTTGGGTAGCCTCTATGTCAGG

>A2:72189471-72189771|varpos=72189621

GTACTATGTAGTTCCATTTATAGAAAGTGCAAAACAGGCAAAACTGATTGATAGTGTTGGAAGCCAGAATAGAGTTACCTTTGAAGGGCTGACCTGCCATTCTTTACTTTGTAAACTTCTGNTTACTTTNTCTCTCTCTCTCTCTCTCT[CTCTCTCTCTCTCTCTTTCT]TTTCTCCCCCATTAGGATAGCTAGTGATTCAGATGGAGTTCTGATGTGCTGGAAATTTTAGCTCCTTTCACTGAGCTGTATACTTCTGTTTCTGCACTTTTGTGCATATATTTTAGTTTGCTAAAATAATAATACAGAGAATACCCA

>A2:72414479-72414779|varpos=72414629

AAAGCATTGGAATACTANATTTGACCTNNNANNTTAGAANAATGCTAATAGACATAGAAAATAGATNTAGAAACTGAAAACGGTAAAATCTTTTTNGGGGTCAGCTTCCAAATATATGTGTNCGTGTTNTGTGTATGTAAGTGAAGATT[ATGTGTGTGTGTGTGTGTGTGTGTGTGTGTGTGT]NACATAAGGATANATTCTCTATTAAAAATTTTATTCACTTGTATCCAACAATTCCTCCTATGGGACTACATCATAAGTCCAGGTCGTATNGTTTCATTGTGAANATAAACACACACCAAAAAACAAATAATCCA

>A2:72992870-72993170|varpos=72993020

TCTAACATTGTGGTANCTGCACGTCACAGAATATTATTCTGCTACTTAAAATGATGCCTCTGGTGAATATTTATGGGCACGGTCAGTGTATATTACACATGGATAAATTTTATATAAAGTGGCTAACTAAAGAGTATGAGGTGTGGTCT[CTTTTTTTTTTT]ATGAAAAATAGCTCCAATGTGCTATATGCATGTGTAATAGCTATTTTCAGTAGGAAACAATCTTTTTTCTCTCCACATACTTCTTTCCACTTTAAACCNTTTGGGTTCCCATTTCCTTTCCCCTTAAAAATAGATGATTCCAT

>A2:73592485-73592785|varpos=73592635

GATTTTTGTCANGTTTTTATNNNNNNNNNNNNNNNNGTTTTTCTTTGCTATTCTGTGTGTGTAGTGATATCACACTGTGGTTTCAGTTAGAATTTGCCTAATGACTAACAATGCTTTATATGTGTGTATGTGCATCTATGTATAATGAT[GATATATATATATATATATA]NNNNNNNNAGTATATATGATGATATGTATATGTAAATATATGTATATATGTATATATATATTTAGGGTGAGTGTTGCAAAGCATGTACTGTCTTATACAATGAAGACATGTTTAGTAATCTCACTCTGGGGGATTGAGTTTGATATA

>A2:74693570-74693870|varpos=74693720

TTTAAGACAGTGTAAATACTCTGTCAGATTCTCTGTGTCTGGGGGACTTTCTGTGCCCCTGTCTCTCTTTCTCTGTGTTCNTATCAGTTTCTCTCAAACTCTACCAAACTGTTGGATTCTCTGTCTCTCTCTGCTTGACTNTCTCTCTG[ACTCTCTCTCTCTCTCTCTCTCTCTCTCTC]NNNNAACATATANATTTAAATATATATTTCTAGGGGCACTTGGGTGGCTCAGTCGATTAAGCATCAGACTCATGATTTTGAGTCAGCTCATGATCTCACANNGTTTGTGAGATTGANCCCCANGACAGGCTCTGTGCTGCC

>A2:75462859-75463159|varpos=75463009

AAATAAGACAGGCAGACTTCAAAAATCCAGGGAAACAATTCATTTGTTTGCAAAGTGCCAGCGTATGGCAAAGTAATACTCAACAGATCTATTTCTAGAATTCAACAGAAAAATTATATCCATTATAAAGGAAAATAATGTATCNNNNN[CCACACACACACACA]ACACACATTTCAATTTAGGAAAAAGGTCGATGCAAATTACCTACAGCCAATTTAAGATGCAAAGACAACATCATATTTCATATTTAATTTCTCCCNCTGAGAAGAGGTAAAATCAAANTCACAGATAATTAAGATGATAAAACCAGACT

>A2:75636104-75636404|varpos=75636254

AAACTTGCCATAATTGCTTATTAGTTCCAGAAGGTTTTGATCAAATCTTTCAGATTTTCTACAGCTAATGCCAATGGCTAACAAAGACAGTTTTATGTCTTCCTTCCCAATCCTTTCCAAACTNTAAACTTTCATNTATTTATTTATTT[ATTTATTTATTTTTTT]NNNGCCTTGCTGATTCCTNAATGGTGTTGAAACAATGTGTTGAGAGGGGNCATCCTTGCCTTNTACTTGGTCTTAGTGGGAATNCTTTGAGTATTATGTTAGCTGCAGGTCTTTTGTAGATAGTCTTTATCAANTTGAGAAAGTTCC

>A2:76573567-76573867|varpos=76573717

TATGGAAATCGTTCACAATACAATTTCAACNNGATATTCTCCTTAGAAATCCTCCACTNAAAGGAAAAAAGGAAAAAGCATCATGTGAGTGAAAGCAACAAGGAGAGAAACAGTTTTAAGAANATGGGAAATNCCNTCCATTTCCATGT[TGCAGCAGCAGCAGCAGCAGCAGCAGCAGCAGCAGCAG]GTTCAAGGCAGGGGGTTCTGACATTCATTGCTGAAGAGGTAGGCAAGGGTTAGATCCTAAAGAANTACCACNGTAAGTTGCCCAGGCATTATTCTNTAGACAGTCACTGAAGG

>A2:78341756-78342056|varpos=78341906

AGATGATGGCAACTACGTATGCTCCATATTCCAGGGGTGGGACACACTTCTGTGAATAACAGTACCTCATACCAGTGGGAACTTAGCTGGTCAGAGAAGAGAAGAGTAGCTATTGGCCTTTCTGGAGCAGGGAAGNTTCTAATATATCC[TACGAGAGAGAGAG]GAGAGAGAGAGAGAGAGAGAGAGAGAGAGAGAGAGAGNNNTCCAGGCAGTGTTTCCTTTAAAATATATACTTTTGGGGCACCTGGGTGGCTCATTTGGTTAAGTGTCCAACTATAGCTCAGGTCATGATCTCGTTGTTCCTGAGTTTGAG

>A2:80840131-80840431|varpos=80840281

TGACAGCTCAGAGCATGGAGCCNGCTTCGGATTCTGTGTCTCCCTCTCTCTGTTCCCCNNNTCCCCTGTTCACACTCTGTCTCTCTCTCCTTCAAAAATAAATAAACATTTAAAANNTATGGGACTGTTTTCTTTTTTTTTAATGTTTACTTATTTT[AAGAGAGAGAGAGAG]NNNNAGAGAGAGAGAGCACAAGCTGGGGGAGGAGGGGCAGCGACAAAGGGAGAGAGAGGATCCCAAGCAGGCTCTGTGTTGGCAGGCTCTGTCAGTGCAGCTTGAACCCATGTACCCCAAGATCATGACCTNAGCAGAAACCAAGAGT

>A2:81912327-81912627|varpos=81912477

TTGAATCTCAGTTGTTGACTGATTATTATGGGNNACATAGTGTTAGAGATCAGATTCTTTAAGGTGCTCTTTTGTAANNGGCTGAGAAACCATATGGCGTGCAGAAACATGGTGTGATTGCTTAATTCAGATGTCTTGTCAGTACTTTGNNN[TTTTTAAAAAAAAAA]AAAAAAAAACAGCTTAGGTGAGCTATAATTCACATACTCTACAGCTGACCTGTTTTAAATGTACAGTTCAGTGGTTTTCAGTACACTCNGAGTTGTGCAACCATTCCCACATTTAATACATTTTCATTACCCCAAAAAGAAACCTCATGT

>A2:81968899-81969199|varpos=81969049

CNNNNNGTTCNTGAGTTCCAGCCCCACNTCAGTCTCTGTGCTNACAGCTCAGAGCCTGGAGCCTGCTTTGGATTCTGTGNCTCCCTCCNTNTTTCTGCCCTCCCCAACTTGTGCTCTNNNNNNNNNNNNNNNAAAAATAAACATTAAAA[AAATTTTTT]TTTTAAAAATATGATGACATGTTATGATAANTTAATANATAATTCCTTGATTCTTGGATTAACTGAGCAAAAAATGGAATTGTTTACCAGAAGAAAAATTTTGTTATACTAAAAACCCAAAATTATAATATNAAGTTAATNATTTACCTTTA

>A2:82571579-82571879|varpos=82571729

GTCCAACTTATAGTTTCAACTCAGGTTATGATCTCCTGGTCCGTGAGTTTGAGCCCCATGTTGGGCTCTGTNCTNACAGCATGGAGCCTGCTTGAGATTCTCTCNCTCTCTCTGCTTATCCCTCACACGCACTCTCTCTTAAATAAACT[TAAAAAAA]GAAAAAAGTCATTATGACTTGAGATAACANTCTTTTGGCTCTGGTAGAGTGTTATTTTGCATATAACAATACATATTAAAAGGCAAATTTTTGTGTGTGTGAAAACTATAAAATTAAAGTAGTCTGTATGCTGCATGTTTATGGTATC

>A2:83218923-83219223|varpos=83219073

AGAAAGACCACATCAAGGCTGTGATTCAAACTCAGAAGGAGAAAGGTTTCTTAGGTCTCCTTCAGGTCAGAATGGAGGATATGATAAGATCTGATACATGTGTTTATCGTGTGCCACAGTCTAGACTGTGCTGACTAGAATATAAGTNN[GTTGTTTTTT]TTTTTTTTAATTTTTAATGTTTATTTTTGAGAGGAACAGAGTGTGAGCAGGGTAGGGGNNNNNNNNNNNNNGGAGACACAGAACGGAAGCAGGCTCCAGGCTCTGAGCTGTCAGCACAGAGCCCGATGTGGGGCTTGAACTCACAAGC

>A2:84727550-84727850|varpos=84727700

TTCTAGCTCCATTCATGTNGTTGCAAATGGCAAAACTTCATTATTTTGTAATGAGTTAATAATATTCCCTTATTCCCTTGTTTGTATATACCACATCTTTATCCATNNTCATCTATTTATGGAAACTTGATAGCNGCTATTGTAAATAAATGCTG[CAAAAAAAAAACAAAAAAAAAAA]NNNNNNNCATAGGGGTGCATGTATCCCTTTGAATTAGTGTTCTTGTATTTTGGGGGTAAATACCCAGTAGTGCAATTATTGAATCATAGGGAAGTTCTNTTTTTAACTTTTTGAGGAACCCCCATACTGTCTTCCACAGTGGCTGCACCA

>A2:84829408-84829708|varpos=84829558

TTTATCTCACTCCCATCCTNTTTCCCACAAACACTGTAACTACACTTTCAAACTGGCCCTCAATGTGTGTGGATTTTCTTTTCTTAAATTTGGCANCCCAGGTTAAACATGTTAAATCCTGGACACCCATATCCAAAGTATGATTAAAA[CTTTTTTTTTTTTTTT]NNNNCGTCAGTATGGCTGAAACTTTACTTTTTGCTTCAAGGTCATGTAAATGCAGCTCTTCCCTCCTTCGGGTAAAGAATGTTGCAGCATCTTTCAATGAGGAGATTCACCTGCAAATCCCTTTGATTAGTGAAAGCAGCTGTTC

>A2:85377445-85377745|varpos=85377595

CGTTCTGACTTGTGTGAGGTGATATGTCACCATGGTTTTGATTTGTATCTCCCTGATGATTAGTGATGTTGAGCATCTTTTTATGTGTCGNCCATCTGTACACTTCTTTGNAAAAANGTCTACTCAGGTTCTCTGTCCATTTTTTAATT[GGATGATGATGATGATGATGATGATGATGATGA]NNNNAGATTTGGTGTTGAGTTATATAAGGTCTTTATACATNTTGGATATTAACCTCTTATTAGAGATAACATCTGCAAATATTTANTCTCANTCAATACATTGCCTTTTGGTTTTGTTGATTATTTCNTTTGCTGTG

>A2:85580849-85581149|varpos=85580999

TNGAGCCCCACGTCAGGCTCTGTGCTGACAGCTCAGANCCTGGAGCCTGCTTCNGATTCTGTGTCTCATTCATTCTCTGCCCCTCCCCTGCTCGTGCTCTGTCTCTCTCTGTCTCTCAAAAANNNNTAAATAAATGTAAAGAAGACAATNTTTT[TTTAAA]ATTTAAAAATTTCAGAATCTTAATAGGACCTTAGGATAAACACCTTGAGANCAGAACTATTTNGACATTGCTATCTCCCCTGTATTGTCTGGTATGTTTTTCTGCANATGGTAGTTTCTAAAANAGAGTTTACTGATTTTAATTTGTTTC

>A2:85720640-85720940|varpos=85720790

AGTTAAGCATCCAACTTTAGCTCAGGTCATGATCTTGTGGCTCATGAGTTCGAGCCCCGCATCAGGTTCTGTGCTGACAGCTCAGAGCCTGGAGCCTGCTTTGGATTCTGTGTCTCCCTCTCTCTGTGCCCCTCCCCCACTTGAACTCC[ATCTCTTTCTCTCTCTCTCTCTCT]CTCTCNNNNNNNNAAAAGTAAATAAAAAATTTAAATATTTAGAAGAATCTTTAANCAAATCTTGTACTTTTAATCACTTTGTATTTTGTCTTTGGGTGAGGAATCCACANTTGGCAGAGTGGAGTTTNTACAAGTTATTGATATT

>A2:85863274-85863574|varpos=85863424

TTATTTATCCATTTGCACTTTTATTCTNGACATGTGGAACTACTGAGAAAATGAATCTGAGCCTCACTAATGGGGCTTGATAACAGGTACATTTGGTTGAATAATATACTCACTTATACTGATTTACAATAATGAATAGGCTCAGAGGCCTAN[ATTTTTTTTTTTTT]AATGGGGAAACACAGTACTGAACTGTACTTTCTTAATTCATGGTAAGATTTANAAGAAATTCAAGTACGTGCACAGAAATCGCTGGAAATGCTTTAAGAGCTGCACGCGATGAGTTAACCCTGAAGAACACATTGATGTGTT

>A2:86834326-86834626|varpos=86834476

TTCTAGCCTTTTTGAAGGATACTCATACTGCCTTCTACAAACTACAAAATTGTACTGAAATAACTTAAGATTATAANTTCAGGAAGAAAACACAAGGCATTCAACAAAGAATACTTGCAATAACACTAGACTTTGACCTTATNAAAAAA[AAAGGGGGGGGGG]GGGGGGATATTATNGAACAGCATTATCTTCCTTTATTTCTACTGGTAAATTTCTGATGAAAGCACTGAGAATAATTTTGTTAGTATACTAGTGGTTACCATATCTGTCCTTTGTACTTGTGAATTTCTAAAACAAACAAGATGTCTTACA

>A2:87040621-87040921|varpos=87040771

CCTCAAGCCTCACAAAACCATCATATGGATCAAATGAGACAGTGGCAATAAAAAAGCNTTGTACAAACGTCTCCCCAAATTATAACACTTTGCTCCATTTAACAGTTATCCTGTGTTGTACTTTTCATGGTTAATTAGGGACAATTTGA[GATTTT]TATTTTTTTTAATTATGTAGTACTGTCTAATTTGAACACAAGTGTACTAATTTTAAAGTCTCTGATTTCTTTCAGATANCCAAGCCAGAAGCAATTCTAATCCTTGACCCTGTGTTACAGCCTTAATGAGGCTCCAGTCACCTGCTGGTA

>A2:87055565-87055865|varpos=87055715

ACAGGTAAACTCTTGAGGGCAGGTATCACGTCTTATTTATTGCTTATCCCAAGTGTCTAATATAGTACCTGCCACATACTAGATAATNGTAATGATTATTTATACAATAAGCAAACACCCAATTAAAAGATAAAAGTTATTTCAGGANN[GTTTTTTTTTTTTTT]AACTTTTTGGTTTACTATTTGCTTCTAGTGCTTTATCTTANAATGCTAATTTACCATTCAGCTTGTTCTAAAATAAGTTTTATATTTTCATTTCATTGGATCTCAAAACATTAAAAACTCCAAAACCTCAGCAGAGGGACAAA

>A2:87934377-87934677|varpos=87934527

CAGCTTTTTAGAATCACTTGAAAATAATGTTNGCATGAGGATTTTTACCATCCTCTGTCATACTTGAAGCACTGTGCAAACAAATTAGTAAATATATCTATAAATACTTGGAAGGCACGCATCAAACACAGACTCATTGCATTTTTAAA[GAAAAAAAAAA]NNNNAAGAGCAGTCAATATATATGGTGATTTAAGNTCTCTTCATCTTTGTCAGAGTGANCTTTTTTATCTTTCTACTTAACTTCATAAAACTATGTAGCCAAGTGCTACCTTACGATGAAATTCTGTGCATAACTAAGGGAGGAAAGAAC

>A2:88209895-88210195|varpos=88210045

TGATGTCCCTACATGTTATTTTACACTTTCCCATTTTCNGCAAACAGGATGACCTAATAACAGCATACAACTCTAAGCAGTGCACGTGNAATAANANAAATTTGTACGTCTGTGCCATACCTATAANAGAACCTCACAACACTGNNNNN[GAAAAAAAAAA]AAAAAAACAGATTTCTAATGGCACCCAGTTAAANGTCAGGGATAGCTTTCTTCAATTTTANACTCGNAAACTCCTCCNNNATCTTCACAGTGCTGGAGTACTTTGACAAGTGGGCTATTNNNNNNTTTTTTTAATGTTTATTTTTGAGAC

>A2:88744790-88745090|varpos=88744940

CCCTCTCTTCTGGTAAGCTGATTTCCCTATTTTTTCCCAATTATTTCTTCTGGGCTAAGCATAATTTCTGAAACAGAACATGGTTCCTAAATGAGTGAATAGATTAATAAATGAATGAATTTCCTAAACTAAAATATGTAAGTTTGTGA[GTTTCTTTTTTT]NNNTTTTTTTTAGAAGAAAGAGTCACATTTTCTTGCTTTTTGGCAGTTCATTAGAGNNTTANTGGGATGAACAGAACTCATCATAGCCCTAACAGGGTTTACGTTCCCAGTTAAAGAAATGCTTAGACATGCTCCTTTAAATTGCAACTATTTC

>A2:90766983-90767283|varpos=90767133

TTAAAACTCTCAGAACATTTTGGGAAGGAGACAGGGGTGAAAGTAAAANNNNGTTTGAGTTAAGNATGGTGTGTTATATAAAACACTGCTGTCATGTGTTATTTAAAATATTTCTCATAAGTCTTTCAAAAACAGTTTAGAGTGGAAAA[AAAAAACAAAACA]CACATCTGTATGCATAATGAGGTCTCTTTAGAGGATTTNTTAAATCTTTTGCGATGCNTATGGCATAACTTAACACCAGAATTTGTACCTATCTGCATTAGGCAGTCATCCAGTAGACTGTCAGATAACGAGCACTNTTCCACTAT

>A2:91624567-91624867|varpos=91624717

AGTGATATTCATCTCTGAAGATCAGACATGGCTCTATCCATTCTCAAGCAGGCCTTTTCATATTTCAGAGAAGTCCTGTCAACATGGCGTGGAGCTTATCAGCGACCCTCAAAAATGTTAAGTATCCTTAGTCTGAAAGTTAACCAGCA[TACACACACACACACACACACACACA]ACACACACACACACNGAGACCTAAGAGACGATTGTGTGGCGGTTGCTTTTGGTTTACCCATTGCTGGCAGTGGGAGCTGATTTAGGCTGGGTACACAGAAAAGCATTAAAAGAATTAAAATTCACTACAGGTTTTTTACTACTTTTAAAG

>A2:93657921-93658221|varpos=93658071

TTTGGCTCTCNAGGTGGTACATCTTACCATCTCAAGAGGATCTGGGTACCTCAGTGTTATTTCCTCATTATATCTCTGCNTTAAAANATCTTAGTTTGAGCCATCCTTTTCCTGCAGAGACCCTGAATGACACAAATAATAGCCATANN[GTTTTTTTTTT]TTTTTTTGTAANGTTTAAATAAGTTAACAAACATGAAATGAGTGGAATTGTGCCTTGTTTATAGTAAACCTTCAATAAATGTTAGTAANTTATCCATGGAAAACATGTAACCAGTAAGCAATACTGCATCCCTCATGAGGATCTTCTGTA

>A2:93717513-93717813|varpos=93717663

TCTATGAAGAGTAAAATAGTATATATTTTAGGCTTTGCAGACAAAATGGTTTCGTTGGAACTATGCAACTTTGCTATTGTGGANAAAAAAGTAGCCATNGACAACACATTAACAAGTAGATGTGGTTATGTTCTAATAAAATTTGATTT[GAAAAAAAAAAAAAAA]GCATACTGGCATTGGCCTACAATTCATAGTTTTCAGAATCCTCTAGTAATGGGTAACTTGGAAAAGTTCTAGGCGAGAGCGGGGTTCAGAGGAAGAAGCTACTTTATAATTCCAAGTCCAGAATNTGGTGAAAGTTTCTCTG

>A2:96466006-96466306|varpos=96466156

TCAGTTGGTTTACCGTCCGAGCCTTGATTCCGGCTCAGGTCATTATCTCCTGGTTGTGAGACTGAGCCCTGCATCAGGCTTTGCACTAAGTGTGAAGCCTACTTAGGATTCTCTCTCTCCCTCTGCTCCTGCCCTGCTCAAGCACACAT[GCTCTCTCTCTCTCTCTCTCTCTCTCTCTCTC]AAAATAAACAAATAAAAGAAAAAAATGATAATTTACAGCAATAATTTATATAAAGTGCATAGCACAGTGCCTNATAAACAGTACTTGTCAATATCAATACATTACCCATTATTATTATC

>A2:97505660-97505960|varpos=97505810

TGGGTTCAAGCCCTGCATNGGGCTCTGTGCTGACAGCTTGCTCAGAGCCAGGAGCCTGCTTCGGTTTCTGTGTTTCCTTCTCTCTCTGCCATTCTCCTTCTCACCCTCTGTCTCTCTCTCTGTCTCTCAAAAATAAATAAATGTNNNNN[AAATTTTTTTTT]TTTTTTTTAAATATNNNNNNNNTGTTGAGCCTGGAAGTAGTATTTAACAGTTCTACTCACAAGTAATAGGCCAGAAATAATCATATGGNGCCACTAACCCNTAATTGGTTGAGAGAGTGCCATAATANAAGAATATGAAGAAACAGAAAT

>A2:100211240-100211540|varpos=100211390

AGTTCNTGGGTTCGAGCCCNGCATCGGGCTCTGNGCTGACAGCTCNGAGCCTGGAACCTNCTTNNGATTCTGTGTCTCCCTCTCTCTCTGCCCCTCCCCCACTCACACTCTGTCTCTCTCTCCTTCAAAAATAAATAAACATTAAAAAA[AAATTTT]TTTAAATAAAATATGATCAAATGAACACAAATCATTAGATTCTTAGACATATTTACTGTTAAAGGAAATGGATTTATGGACTAACAATGTATTTAACCCTATTAAAATGANACATAATGACNGAGGAATCAATTTTCTTAGTTAATCCAA

>A2:100558280-100558580|varpos=100558430

CTTTATATGTATTCTAGATACCAGTCTTTTGTCAGATATGTGGTTTGCAAAAATTTCCTCTAGTCTGTAATTTGTTGTTCATTCTTGTAACAGTCTTTTTCAGAGCAGAGATTGTTTTTTGTTTTTTAATTTTTTATAACATTTATTTT[TGAGAGAGAGAGAGAGAGAGAGAGA]NNNNCANTATGCGTGCATGNGAGTTAGGGAGGGGAGGAGAGAGGGAGACAAANGATCTGAAGCAGGCCCTGGAGCTGTAAGCACAGAGCCAGATGTGGGGCTCAAACTCACAAACCTGTGAGATCATGACCTCAGCCC

>A2:101322113-101322413|varpos=101322263

NGTCTGGCTCTTTATTTCCTTTGTGGTTATTTGTCCATTTAGTTTTTATACCTGGAGTCAGTTTTGATAATTTACATTCTTAGAGAAATGTGTTTCATTTAAGTTTTCATGTTTAATAGCTATTTATTATATAAAATGTTCTCGTGTGC[ATGTGTGTGTGTGTGTGTGTGTGTGTGTGT]TTTAATTTTATCTGTGAATGTGGATATAGTTTAAATCCTGATTGGAGCAAACCAAGTGTAAAAAAGACATTTGTGAACTAATTGANGCAATTTGATTATAGAAGACAGTTGTGGAAATTTG

>A2:101798708-101799008|varpos=101798858

ACAAATATGNNNNNTTANTTTATTTTTTACCTTAACCTTTATTTATTAAAGAACTGAGAACATTCAGATAATAGAACATAAAATTAAAGCAATAAAGGTGAATGATGAAATAGAAATTATTCTGATGGCAAAAANTATATTATNNNNNN[CCCCAAAAAAAA]AAAAAATCAGATNCTCCCCAAAGCAGTAATTCNAAAAGTGCTAACTTTTGAACAGAAGTAGCAAGTAGGCTCGATGAATTGTCTGAAAATGCAGTTAATGAAGAAGAATGAATCCTATGAATCCCTTGGGGAAAGAGCTGTGTTGCTTGG

>A2:102182678-102182978|varpos=102182828

TTTCATAATATGCTTATTCTATTCAAATCTTTTGCCTATTTTAAAAAANNNNNTTAAGTTTATATGAGTTATGAGTTCCTAATANGCTCTAAATATAAATCCATTATCAGACCTAAGCTTGAAAACATTTTCTTCAAATGTTTAACTTGCTTTTC[ATTTTCTTTTTT]ATTTTTGTCTTCTCAGGTTTTTAAAAAAATTTGTTTTTNNNNATTGAAATATAGTGGATATACAATATCCTATTCATGTATATTTCACACTGATCCAATATTTTTATACATTACAANATGATCCTCACAATAACCCTTTCATTTGCTTAACAGT

>A2:102489960-102490260|varpos=102490110

AGGTCAAGGCCTACNTTTTGGCTTAGGAAAAAACTTCTGGAAGAAGCCACTTCTAGTTAGATGACAGCTTTGTGTGTGTGTGTGTGTGTGTGTGTGTNNNNNNNNNNNNNNNNNCAAGTGAGCTCTTGTTTTCTCTCTCTTTTAAAAAA[AATTTTTTTTT]TTTTTTAACATTTATTTATTTTTNNNNGAGACAGAGAGAGACAGAGCATGAANGGGGGAGGGTCAGAGACAGAAGGACACACAGAATCTGAAACAGGCTCCAGGCTCTGAGCTGTCAGCACAGAGCCCGACANGGGGCTCTAACTCATGGACCAC

>A2:102778416-102778716|varpos=102778566

TAGTATATTACCTTATCAGATTGGCATCTTTCACTTAATAGTATGCATTTAAGCATTTAAGATTCCTTCATATGTTTTCATGGCTTGATACCTCATTNNTTTTACCTCTTTTTATTTTGATTCATCTATTATTTAGAAGTATNNNNNNN[TTTTTA]ATTTTTAAAAATAGCTCTGAAAGAAGTTGGTATTTATTTGAGAATAAATAAAAACTTTAACCCTATATATAAATGTTAACTCAATATGGATTATAGAACTAAATTTAAAAAATCTGTAACTATAAATCTTACAGAAGAATACACAGGAGA

>A2:102807826-102808126|varpos=102807976

ATGTCCATACAGAACAGTGNTGATTTTTGTTTTTCTTGGGCAGCCAGCCTTTGGTATTGGATATAAGATATTATACCCACAGAAGATTAGTGAATGGCAGTTTAAAAATTATAGTAGTTCTTTGCTGCATGAAAAGAGCTTTAAAAANN[GTTGTTGTTT]GTTTTTTTTGTTCAAATAACATTGCCTCCTGAAAATGACAAAAATGAAACAACTAGCTATCTTAATTTCAAGCAGTGTTATCTCAAGCAACAATAGCTCAAGGATTTTGTTTTTTTTAATGACCTAACCCTGGGGGTAAATGATTGATA

>A2:102944930-102945230|varpos=102945080

ATTNTGGAGGCAANNTCAGCTATGACAAGTGNTGCTTATCATGTATTAATTCACCCCCAAAGGGATCTCCAAAGTGTTTTTCANATAACTGCTATATTCANGGATCAGTTAAAATTTTTCTTAATGTTTGTTACAAGGAGATTATTNNN[ATTTTTTTTTTT]GCTCTTTTGATATCTAGAGCTCTTTGTTGGATCACCAATGAGATTCCAACTCAGCGTTGTCAATACTGAAGCCTAGAGTTTTCATTCTAGCNTGGTATAATAAATACAAATCTTCTGAAAAATAATACTGATCGAGACAGGAT

>A2:103543828-103544128|varpos=103543978

CAGCTAATTAATATTAATAACAATACATTATATTTTGAATGTTGCAGTGTTTGTGATATTGCCAGTTTATATTTTAATTTGTTGGGATTTATAATCTCAAACAAAGTAGTGTTTACATTCATATCTTGTTCTCTATTTGTAGACTTGTN[ATTTTTTTTTTTTT]CTGCAAAGAGTGTCCTAAACAATATAAGCTTCTGCTCACACAAAACCTGGATCTACCCTGAAAACAGGGAAGTCTTTCATCACTGTGTTTGTGTGTNTCTGTGTGTGCTGTGTATATATACATGTATGCATAAATTGAGCCAT

>A2:104693896-104694196|varpos=104694046

CCACNAAGTAGAAAAAAAGGTAGAAATTTACTTACAAGACGTAAGTATTTGAGCTAGGTTTACTAAGAACCTCCCCAGTTACTTTAATTTTATAATATAGCCCTAGACTTTCTAATTAAATATGCAACTTCTGTCATAAGGAAAAAAAA[AACCC]CATCTTANGCTNTTTACACTATATTTAATTAAGCATTTCTTGTAAGTTCNTTTCAGTATCTATCATGGCCCACTAAAGTTGGATTCAGTTATAAAATGGTAGATTAATTACCTTTTGTATTAAATGAAATAAGAATCTCAAATATGCAAA

>A2:105401860-105402160|varpos=105402010

TTCTGCTCCCATTTGGGTAATTCAGGCAACNTTATTTTTCATTAGTTTTTACTGTAAGNNTCCAGCAAATTCTACAAAGTTAAGAGCAAGTTAATGTTTATCACTTGAGTTGTGATAAATNCCTATCACAAGCTCTTAATAATTACACA[AATACACACACACACACA]CACACACACACACACACAACTAAGTCATTTGTACTAAAATACAAGTATTGAAGTACAGTGTCATTTTCCCATCAGTGATCTACTTCATTACTTTTGAGGTTANCAGAAAAGGAAATAGGGGAGAAAGAAGGATCAAAGGCATTGGGGCA

>A2:105778769-105779069|varpos=105778919

TCTTTAAGTGCTTGATATATTCTAACTATTAGGCATTTAATGTTTTTCTGAGGTGACCAAAAAGTATGTGGTTGTTAATATGNAGTTTTATATTANGAANATATAAGTCCTGAAAGTCATTATTATCACTATTCCTACANACAAACAAA[CAAACAAACAAACAAACAAA]NNNNTACCTCTCAGTAATACNGACAACAAGGGNATATCCCAACAGCTCTATTGCTTAAGTTGGTCTCAAACAAATCAAAACACAAGCAACACAAAACAATAATAACAAAATAACAACACAAAATAGCTTCAACAGTGTTAAAGCTAAAAA

>A2:106280932-106281232|varpos=106281082

CCTAAGGAAAAGCTTGAGAAAGATCCATGCCCTGTCCTTGGAGGTGGAGTGCTAGGACCCAAAGNCTCAACCCAGGTTGTGCTTTCTCCTAATTTAGCTTGTTGATCAGTTTTACTTTAGTGAATGGATTCTTTGGTGCNAGCTAGTGA[TACACACACACACACACACACACACACACACACACACACACACAC]CACACACACACACACACACACACTCAGGGAGTGGCTTGCATAGTATATACTTGGGTCTGTCCCTTAATACATATATTTTATGTAGCAAAATTCAGCTTGTTATAGAACTACGAATGTATGAAAGGTAACCATGAACTTGTCTGAG

>A2:107214096-107214396|varpos=107214246

TAGNNNNNNNAAAAAAAANACACTTCTCTCTTCCAGTGATCTGAAGCCATATGGGAAAACAGTTGCAGTCAACCATCTCTGTGTATATCTCTAAACAATTNCTCATGAAATCTTTACCATGTCCGTTAAAGAAAGAATGAGGAGAAACA[TAAAAAAAAAAAAAAA]AAAAAAAATGCCTAGTATTCATCTTTAAANCTGTGAAAGATTGTCTACTATGTTGTAATGACCATTTACTATTTATCTCCCGATTTCTAATATCTGGTAAATAAAATAGCAGAGAATAAATAAAGTTACCAACACGTTTTAGAATTCAAA

>A2:107934465-107934765|varpos=107934615

AGTGTCAAAACTGAGGANACCNTGTTGGAACTGCTGATCATTCTCATCATTCTAAAAGCTGTANGTCAAATTTGTACAGCAGATCTAGTTTATTCACTTTATTGCTCAGAGATACACTTGAGGACAAGGGATAAAATNTGAAAGCCAAA[TATAATAATAATAATAATAATAATAATAATAATA]CATAGTTCTCAGCACTATTTATCAAGATTAAAAGTGAACNNNNNNNNGCTGTGAACCTTAAACTACTCTAAAAATAAAAAGTCTATTAAAAAACAGCAAATGTCCAGAAAATAGGATATGAAAGAATTTAAA

>A2:108442583-108442883|varpos=108442733

TGTTGTGTCATTTTATTATTNACAATTGTTATCCATTCCCCTGAAACTGAGCATTCCATTTGCTCTTGATAATTTGCCTTAAGAAATAATAAGGTGATGGTAATCTCTCTACAATAATCCTTCAGCGAATCTTTGTATTTTNNNNNNNN[TTTCCCCC]CCCTCCCTGTCTTTGTCCCTCTCTTCCTTCTTCTTTCCTTTCTTCCTCCTGATTTTCATACCTTCCTCNCCTCTTAATCTTTGTATTTTCTTATGGCATGTTGTTGTGGTTGGAATTANTGTGTCAAAGACTACAAATACTCTAAATTTT

>A2:109576034-109576334|varpos=109576184

TCACACCATCTGACCCAAGCAAAACAGATGTAAGTATTATGGCTGATAGAGAATTTAAAANAAGTATCATAAAGATACTCACTGGACTTAAGAGTGGAGGACATCAGTGAGACACTTAACAGAGAGNAAAAAAAACAAAAACAAACAAA[CAAACGAAAAAGAAAAAA]AAAAAAANAACCAGAGATCAAGAACACAGTAAGTGAAATTAAAAATATACTTGNTGGAACAAATAGCAGGCTACATAAAGCAGACAAATTATTTAATGACCTGGAACACAGACTAATGGAACATAACCAATCTTAGCAAAGTAAAGATAA

>A2:109703365-109703665|varpos=109703515

TCTTATTCAGGATCCCAAACTCCAATGAACATATTAGCTATGATAGTACTTCAACAAAATCCATGTGTAAAATTTTGATTATTACTTTTCTTTATTTTTTATTTAATTTTATTTTTTTTAATGTTTACTTATTTCTGAGAGAGAGAGAG[AGAGAGCGAGAGAGA]GAGAGAGANAGAGCNTGAACGGGGGAGGGGCAGAGAGAGGGGGAGACACAGAATCTGAAGCAGGCTCCAGGTTCTGGGCTGTCAGNATAAAGCCTGCTGTGGGCTCGAACCCACTAACCATGAGATCATGACCTGAGCCGAAGTCAGACC

>A2:110315475-110315775|varpos=110315625

AGTTAACTAATAGAATGAAAGGACAAGATACCAAGTGAGAGAAAATGTCTGAAAATCACNTATTTGACNAGGACTTGCCCCACAGNNNNNNNNGGAACTCTCAAAATACCACAGTAAGAAAACAACCCAATTAAAACATGGNNNNAAAA[AGAAAAAAAAAAGAAAGAAA]AAAGAAAAGAAAGAAAAAACAACAACCTACCAGACACTTCAGCAGACAGAATATTTGGAAGGCAAAGAAACACAGGAAAAGATGTTCAACATCATTAACCACTGGGGAAATGCAAATTAAAACNATGCTGAGTTACCATCACACCCAAGT

>A2:110455824-110456124|varpos=110455974

TTCTAGCCCTTAATTATTCCTACCAGTTCTCTAAATATTTTACCCAAATTCATTTATCATACAAAGTCATGCAAAGCAAAATCATTTGTCAACAATGAATTAATAATTCTGGGTAAGCAAATGAAGAAAAACCTCATTCNTAATAAAAA[AAAAAAAAAACAAAAA]AATTAAANAAAAAAACACTGAAATATTTTAAGTCTCAAGTAGAAATGATACATCTACTTCTTAGAATGGANGANAAAATGGATTTCTTGTAACTCAGAATTNTATTTATTTTTTTAATGTTTATTTATTTTGACAGGGAGAAAGACA

>A2:110557745-110558045|varpos=110557895

TGTGCATGCTAGGCATCAACATAAACATGATTTCAAAAAATCTGAATAGAATTATTAATGGCATTTTCCCAAACTACACACATGATTGGGGTTGAGAGGATTATTCTATTATGGGCCATTTTCCTAGGAGTAACTATGAATTAACNAAA[CACAAAACAAAACAAAACAAAACAAAACAAAACAAAACA]TTTAATCAGTGAGACTTAGAATTATAACAATTTGCATGGTAACTAATAGAAACCCCATTTATGTTTTAAATCAAGAAAAATGGGTCACAGAAGGCTTAATAGTGAACTTGTCTTTGCCTACTGTCACCCTGG

>A2:113856367-113856667|varpos=113856517

GGTTGAGTGTCCGACTTNGGCTCAGGTCATGATCTCGTGGCTTGTGANTTTGNNTCCTGTGTCGGGCTCTGTGCTGACAGCTCGGAGCCTGGAGNCTGCTTCAAATTCTGTGCCCCTCTCTCGCTCTGCTCCTCTCCCNCTCATGCTCT[GTCTCTCTCTCTCTCTCTCTCTC]NNNNAAGAATAAATAAAAAATNNNNNNNNNNNNNNNNNNNAAGAATTATGTTAAATCTACTCTCTGCTAGNGCTCTATTAANGGAANAACAAAGCCTGGCTGACANNACAACTGTTTACAACANGGTTTACTGATTATTTTA

>A2:114116291-114116591|varpos=114116441

AGCCTAATGTGGAATAAAGTTTGTGATAATGGCTATAATTCAGTTCAGGACAATCTATGGTAACTCTGTCTACCTCTGAAGAACTGAAACAAACTTCAGATTTTTAAAAACTATTAGAATTCCATGTAGAAGTAGTCAGATTTTCCTTT[GTTTTTTTTTTTTT]NNNNNNTTGTTTGTTTTGTTTTTCTACCACCTTCTTAAATTAGTGTGGTTCAATAAAGAATGAATGGGATGTAGGGAAATTGTTTTTTGGTTTTTGGNTTTTTTTAATATTGAAATTTTAAAGGAAATAGTCACATCCTTTGGGGTCAGTT

>A2:114399262-114399562|varpos=114399412

ATATAAATCTGAGGGGATACCTTTCAGTAGATAACACTGGCTTTTCCAAAGTGAAAGATTCCAACNATCTTTCTGGCAAAGATTAAAATAATGAAATCATGGTTAAAAATAAGTCAAGAAGGAAGGAAGTGTGGGAAGAAGGAATTTGG[AACACACACACACACACACA]NACACATGAAAGGTGGAGAATCCAGTTTTTAGAAGTTGCTCTTGAAAATGTTTATAGCCCAAGTGCTCAAGAAAAACTTTGGAGACCTCCCCATCATGAGTTATTATAAGCATGTTAAGTATTGATAGTTTCATCGATCNC

>A2:115525163-115525463|varpos=115525313

CCGTGAGTTCGAGCCCCGCNTCAGGCTCNGAGCTGTCAGCACAGANNNTGTTTCAGATTCCGTGTCTCCNTCTCTCTGACCCTCNCCTNTTCATGCTCTGTCTCTCCCTGTCTCAAAAATAAATAAAACGTNNNNNNNNNNNAAAAAAA[AAAAAAAAAAAAGAAA]TGCCCTGAGTAGCTAGCTACTGGTCCCACAACAGAAACACATGGAACATGTCTAAACTCAACTCACAGTGTGAAGGAGAGCCACCCAGCCCACCTGCAGACCATGAGNATGAAATAAGTGATTTGGGGGTTGTACTTTATACATCAG

>A2:115854805-115855105|varpos=115854955

CTGACTTCAGCTCAGGTTATCAGGTTACAATCTCACAATTCATGAGTTTAAGCCCCACATCGGGTCCTCTGGTGTCAGCGCAGAGCCCATTTCCAATCCTCTTTCTTTCTCTCCCCCTCTCCCCCAAATAAGTAACAACATNNAAAAAA[AAAACCC]CCAAAAACCTAGCGTTATTTAAAAACAAAGAAACACTTTCNCTTGTCAAATATTTGTAACTCTGAAGCACAGTAGGTACAGTGAAGACAGGATAAATAATTGACTNATTTCCCTTCTGCAGCGGATGGTTTTGTGCCCCTTATCACAACCCCTCA

>A2:116482198-116482498|varpos=116482348

AATTCATCTGGTCTAGTGTGTCATTCAAAGCCATTGTTTCCTTTTGATATTCTGTTTAGTGATCTAGTCCATTGATGTAAGTGGGGTGTTAAAGTCCCCTACTATTATTTTATTACTATCAATTATTTCCTTTATGTTTGTGATTAGCN[GTTTTTTTTTTTTTTT]TTTTTTTTGTATTTAGTGCTTTCATGTTTGGTACATAAATATTAAGAATTGTTATATCTTCTTATTGGATTGTCCCTTTCATGATTATATGGTGTCCTTCTTTGTCTCTTGTTATAATCTGTGTTTTAAAGTCCACTTGTTTGGTATAAA

>A2:116545655-116545955|varpos=116545805

ACTCCTAACTCCTCTCTCTCAAGGGAGACAAGGATGCNACACATTCTCCTTCTTCACTCACCTTTCTCCTTCCAGCCCCAAGTCTTGNTTTANCTGTAACAGCTATATGACTCTTAAAAATACTAATATGAATATTCCTTAAAAAAAAA[AACCAACCC]CAGTTCTGTATGCTAGGCATTGTGCTAGCGACTTTACAAACCTTTAGCTCCTTTCCTCCTAACAGCTACCCTGTCAGGTCAATGTCATTATTCTCATTTAAGATGTGGAAGCTGAGGCTCAGGCATGTTAGGTGGGAGCTTGTTCCTAAA

>A2:119547952-119548252|varpos=119548102

CTGGAGGATGGGAGCNGGGCTGGGGGGGCAGGAGACAAANACTGAAAAACAACACAGATGTTTTTGCTGTTACTTTCATTCTCTTATTTTGTTGATAAGCTTGACTTTTTATCACTTTTATTATCTANTTTTCCAAGAAATGTATTTTT[CTTGTTTTGTTTTGTTTTGTTTTGTTTTGTTTT]NNNNNNNNNAAGAAGTCTAAAACTAAGCAGATACCCTACGCCAAAGCACACCTCGGAGTGGGTATCTTGGTTGCTGAACACTTTGCAGATTAAGGTCTCAGTCTCTTTAAAAGATTATTGACAACTAAGCATTCTCTCACNGTTATG

>A2:120324703-120325003|varpos=120324853

CTTAACCATCCCCTATNGTTTGACATTTAAGTTTGTTGTGGNNNNNNNATTATTATTANNNNNNNNNNNCNCANATGCCGAGTCTTTTGGCAAGANAAATATATACTCTGAAGACCTTAATCTCCTTNNTCNTATGCTGGCTGNNAAAA[AAAACCC]CCTTCCTTTAGGCTTGAGTTTGGACAAAAATGTGTCNGAGCTGAAGCTGGTCTTGGACACCCTCACCCTCACTTCTATCCCTCTTATTTCTGCCCCTCTTCCTCTTAGCACATTCTGTTCCACTGCAGTGCCTGTCACAGGAACTCAAGC

>A2:121771209-121771509|varpos=121771359

GTAAATGTCCTACCTCTGTCCTTTTCCTTTTCCCAGGAAAATCTGATAAGCATTAGAAGAAATTTGAGATAAATGGNNNNNNNNTAATNCAAAAGNTTAACATTAAGAATATTTACATAGCNTTTCTCAATTTACAAGTGTTTTCACAT[GATTCATTCATTCATTCATTC]NCATTCNTTCATTCATCAAACATTTATTGAGGGCTCACTTTGCTCACCAGGCCAGGGCCTCCTAGAATAGGATGTNTGCCCTCACTTTCCTGGGGCCATTGTACCCATCTGAGAAGCAGTGTAGCATAGTGGTCAGGGGTGCAAAGGT

>A2:122935033-122935333|varpos=122935183

AAAGGTACAACAGCAANTGTGTGCCTAGATTGGTTATGAAACGCCCATATTGTGGACTATCATGCAGGNATTACATGATGCTGTAGTTCTTTNTTTTCATGATGCNTTGTTAGATGAGAATAAAGTTACAGCATAGCATGTACGGCATA[AATATATATATATATATATA]CACACATTTTATTTATTAGAAATATGTACATATATTAGAAAGACACAAGTTATGAAATACATTTTTATTACACACAGAAAAATCTAGAAGCATATACACCAAAATATTAACATTGATCTCCTTGNATTGAGATCTGGGA

>A2:123767669-123767969|varpos=123767819

ATCCTACTTCGGCTCAGTTCATGATCTCTCCGTTCACNAGTTCAAGCCCCATGTCGGGCTCTGTACTGACAGCTCAGAGCCTGGAGCCTGCTTTGGATTCTGTGTCTCNNNNNNNGNCCCTCCCCAACTCNTACTCTNNCTCTGNNNNN[GTCTCTCTCTCTCTCTCTCTCTCTC]CTCTCTCTCTCTCAAAAATAAATGAAACATTAAAAAAATANATAAAAATAAATAAATAAGTAAAACCTGGTTACTGAGATCAGCAGCCCCACTGCACTTCTTCGGCCCCCTAAGAGGTTCTCTTAATTTCCTGCAATAACAGCTC

>A2:124157196-124157496|varpos=124157346

GTATTTCTGCAGTGTTGTTTNTGATCCCTCCTCTTTCCTTGTTGATTTTATTTGGGTCCTTTCCTTCTTCTTTTTGATAAATTTGGCTAGGGGTTTATCAATTTTGTTGATTCTTTCAAATAACCAGATCCTGNNNGATGTGTTCTATT[GTTTTTTTTTTTT]ATTTCAATATCATTGATTTCTGCTCTAACCTTNNNNNNNCCTGTCTTCTGCTGGTTTNGGGCTTTGCTGTTCTTTTTCCAGCTCTTTAAGGTGTAAGGTTAGGTTGTGTATCTGATACCTTTTTTCNTTCTTTAGGAAGGCCT

>A2:124891573-124891873|varpos=124891723

CAGCATGAAATCTGAGNTTAAATGTTTTGCTTGGGGCAGAATGACAGCACATGCTNAGGGTCTTTTAAATTTTCTGTGAANGTCTACTTGACTTTATGTTCCTNTTCTCACTATTTCTCCCTTACGCNTGCACACGCNCACACACACAC[AGACGCACAC]CACACGATCAGGATTAAAGTGAAGTGAGAGAAATACTTGCCCTGGGCACAGAACTTAAAGGAATACCAAAAAACACAGTCATCAACATAAATAATATTTTCATGCAATATTTTTAAAAATCAAAATTAATGCAAAAAAACCCCCATCATG

>A2:125143954-125144254|varpos=125144104

GGAAAAATGCTTTCCAGGCAGAGTCCAAGGGGCTTGGAGANTTTGAGGAATAACAAAAGGCCACTGTACTGGAGGCAGAAGGAGCGAGGGGCAGAGGGAGAAAAAAATGCAGAAACATANGGCTTTACAGACTGTGGAAAGGGCNTTGG[GTTTTTTTTTTTTT]TTTTTTTNAATATGTTTTTAACATTTATTCATTTTTTGAGAGACAGAGAGTATGAGCAAAGGAAGGGCAGAGAGAGGGGAGACAGAATCCGAAGCAGGCTCCAGGCTCTGAGCTGTCAGTGCAGAGCCTGATGCGGGGCTTGAACTCAGGA

>A2:125376739-125377039|varpos=125376889

GAATTACACCTACCTTTTAGTGACAAAGAAATTTGGATTTATAGCTATTTCAAACCCATATGGCCTGCATTCTTCTTNTTCTCTCTNNCTTAGCATCTGTTGAAGTTAGAGATCCATCCGTTACTAAGCCTATTTNNNNNNNNNNNNNN[TTGTGTGTGTGTGTGTGTGTGTGTGT]TTGTTTAGTGATTCTGCAAGTGGATTCCCCTTAAGGGCAAGAATCCAACAAGGATAACACTATCATCCCAGTCATTTAATCCTATTTTATCAGCAATGCCCAATGAAAGAAATTAGACAANNNNNNGAAATAGAAAAAA

>A2:125693406-125693706|varpos=125693556

CAACAGGAATTTTGTAAAACTTGACAAGATAACTCTAAAAATTTATATANAAATGCTTAGTCAAGACACTAAGCANNNNNNNNTGGAAAACCTTGATGAACCAGGTACCAAACCTTATCATAAAGCCATAATAATAATAATAATAATNN[TAATAATAAAAA]CGTTGTGGTATTGTCTTCAGAGTAGACAAATTAGCAAATGGAACAGAGAAAGAATNNNNNNNNTGCTCACATTTGGAAAACTTGATTTATTTCAGAGTAGCATTGCAGAGGGATGGAGAAAGGATACTCTTTCAGATACATCATAC

>A2:126592895-126593195|varpos=126593045

TTCTCTCTGATTTTATTCTTTCCCCCAATTTTAAATCTCTGCAGCTTGCCCCNTGCTTGANACAGTGGTATTCTATAACTTNGTCTTGTGTATTTTTATTTTTATTTATCTTATTATTTTTAAAAATTCTTTTGATATTTATTTATTTT[TGAGAGAGAGAGAGAGAGAGA]CAGAGTGAGAGCAGGGGAGGGGCAGAAAGAGAGGGAGACACAGAATCTGAAGCAGGCTCTGGGCTCCAAGCTGTCAGCACAGAGCCCAGTGCAGGGCTCGATCTCACGAACCGTAAGATCATGACCAGAGCCGAAG

>A2:128874517-128874817|varpos=128874667

CTGTTCATGAGTTCAAGCCCTGGTNGGGCTCTGTGCTGATAACTGGGAGCCTGGAGCTCCTTCAGATTCTGTGTCTCCCTCTNTCTCTGCCCCTCCCTCCTTGCACTCTGTCTCTGTCTCTCTCTCAAAAATAAATAAACATTAAAAAA[AAATTTTTT]TTTTTAAAGNATATAGGTCCAGAAATTATGTATTCAATGTAATTTAGATCGCTTTACTTCCTCTTTTTTTNNNNNNATGTTTGGAAATGTTGGGACTCATAAGACAAAGCACTTCTGCATAAACATTACATAGACTTAAACAGTTAAGAGACATGAT

>A2:128920489-128920789|varpos=128920639

AGTAGGAAGATGTGAGAATCTGATTCTATTTCTCTCNAGGTAATTTTAGTAATCATATCATGTAGCTTCTTTGTCAAAAGAAAACAGGCAGAATTATCATTATAACTAGAACAGGTGCATTTTGATGGATAGCTATCATAGTAAAAAAA[ACCCC]CCACACACACAGTGTTTAAATCACAAAGGTTATAAATAAAGTAAGGGCTATACTGGAGGTTGACATAAAAAGGAACTCTAGTTTATCCTTTTGTAAAGTAAAAAAGGAAATTGGCTCCTNNNNNNNCAAATATACATTTTAGTAGATTGT

>A2:129025216-129025516|varpos=129025366

CTTCACATAGCATAGGCAGAATTATTCGGGTTTTAATTTTTTTAAGTAAGCTCTAAGCCGAACATGGGGCTTGAACTCACGACCCCACGAGTCCCATGCTCTACCAACTCAGTCAGCCAGGCACTCCCCAGGGTTTTANNNNNNNNNNN[TTTAAA]AAGTGTTACTCTCTCTCAANATTAAAATAACCCTGCATACATTTTCCTAAAATGCCTTTTTCTTTGANTACTATGTAGATAGTTTCCTTGTCTANTTTTCTAAACTGGAAAAACTGAATATGTATCATCTAACTGTTTTTTGTTGTCTTA

>A2:129244822-129245122|varpos=129244972

AAACACTAGCTTATAAANATACATGCACCCCTATGTTTATTAAAGCATTATTTACACTAGCCAAGACATGGAAAAAACCTGTGTCCATCAGGTAGACAAATGGGTAAGGGAAATNTGGTATATATACACATAGGAATATTACACAACCA[TAAAAAAAAAAA]NGATGGGATTGTGCCATTTGAAACAACATGGATGGAACGAGAGGGTATTATGCTAAGCGAAGTAAGACAGACCAAGAAAGGTGAACTCCATAAGATNNNNCTCATAAATGGAATCTAAAAAAATTTAATAAACAAATAAAAAGA

>A2:130213960-130214260|varpos=130214110

NTAACAGTAGTCAATCCCCACCANGAGTTAACAGGATGCAGAGGAGGAGAGTATAGATTAGAATAACTAAAGCTTGTTTTCATTTAGCGATTTGGCTTCTAAATTTATTTTAAACTGTACTCCACATTAAAATAAATTTGATACATTNC[AACACACACACACACACACACACACA]ATTTAAGTTTCATGAAAACTCTGGGCAATGTAAACTCATATNTTCTNNNNNNNNNAATGCTGGATGATCTATTAATTGATTTCATTAACTACTAATAAGTCATGATCTGCAATTTGGAAAACATTGAGCACCTACTAGTAC

>A2:130596208-130596508|varpos=130596358

TCTTCCACTTCTTGTGTTCACAATTCCACTGCCNACCATCTCCTGCACCACAAAGTCAAGCCCAGAANTTTTCTACGGAGAGACTCTCAAGNAGCATGTGGGANNAGTGCCAATCTGAAGGTGTGCAGATTCTGCTCAAGCCNNGGGGG[GGAAAA]AACCAAGCTACCTTGTAAACAAGTTGTCCCTGTTTTCAGAGGCTCAAAAGAAAAGTCTCAGTACAGTCCTATCCTACCTCNATTGGAAGGGCACCTGTACCACATAAAAATCAAACACTTTTAAAAGCTTCTATCACAGGGGTGTCTGGG

>A2:130997260-130997560|varpos=130997410

ACCTCGCAGACAATAACAAGTTCNNNNNNNNNNNTGTCAAAACANTATTTACAATGATTCTACTTGCCACAATTCAATTCAAGTCTGGCAAAGGCTGCCCTTGCCCTTTATGGGCTGACAATCTAAGATGAATGGAATGTAACGTAAAA[GAATAATAATAATAATAATAATAATAATAATAAT]GAAAATGCTTTGTCCCTTCTGACATAAAGGAAAGAAACTCATCTCTCNAAGTCAGAGTTTTGGCTTCGTCTTTCTAGTAATAGTNTCTCTGACTCATGAACAGATTGTGGTCTGAAAGGTCACCTGTGATCTGACTAT

>A2:131364307-131364607|varpos=131364457

GTGGTTGNAAATGGCAAGATTTCATTCTTTTTGATCTTCACTGGGGATATCAATGCCCAGCTGGAGACTGCATTTCCCCATCTCCCTTGCAGCTGGATATGGATATTTNGCCAGGAGCTGGGGGCAACTTACTGCTTTTTAAAAAAAAA[ATTTTTTTTTT]TTTTTAATGTTTATTTATTTTTGACAGAGAGGGAGAGAGACAGAGCATGANCNGGGCAGGGGCAGAGAGAGAGGGAGACACAGAATCCGAAGAAGGCTCCNGGCTCTGAGCTGTCAGCACAAAACCCANCAAGGGGCTTGAACTCACAGA

>A2:131494728-131495028|varpos=131494878

CTAATCATGAGTAGGTCGATGTCAAAAACCATTATTTATTATGATAATTGAATCAANTAAAAATAAAATTCTGCAAAATGAGCTGATAGTGTCCTATAGATAAATCTGTCACTACAGTGATCTTTTTCCTTNTTTTCACCCANNNNNNN[TTTTGTGTGTGTGTG]GTGTGTGTGAGCTTTGATCACTGTCTTCATTTCCTTTCTGTCCCCTCATTATAATAGTTTGGTACATACCTGTCCNCCTNANAAGATACACCCTNGAATGTCACTCATTACTTCCTTATTGAAAAATTATGTTTCTTTTACTCTCATGC

>A2:132076783-132077083|varpos=132076933

GAATCTGAAGCAGTNTCCAGGNTCTGCACTATAAGCACNGAGCCTGATGCAGGGCTCAAACTCATGAACCATGAGATCATTACCTGGACTGAAGTCGGAAGCTTAACCCACTGAGCCACCCAGGTGCCCCGTCTGGAGTCCATTAAAAA[AATTTTTTTTT]TTTTTTNAAGTTTTTTATTTTGAGAGAGAAAGAGAGAGANNNNNNNTAGAGTAGGGGAGAGGCAGAGAAACGGAGGGAGAGAGAGAGAATCTCAAGNAGGCTCTGCACTGACAGCAGGAACCAGATGCAGGGATCAAACTCAGGACTGAGAGATCATGA

>A2:132101839-132102139|varpos=132101989

AGTGNAGAGCTGGANNTGGGGCTCAATCCCACNAACCATGAGATTGTGACCTGAGCTGAGATCGAGAGTCAGATACTCAACTGACTGAGCCACCCAAGCACTCGAGACTGCACAATTTTAATTTTTTAGCAGTTTTTTAAAGTNNNNNN[AATTTTTTTTT]TTTTTTCCATATAATTTTTAATTTAAACCCACGTTAGTTAACATATAGGGTAATAATGACTTCAGGAGTAGAATTTAGTGATTTATCACTTACATATAACACACAGTGCTNATCCCAACAAGTGCCCTCCTTAATGCTTATNACCCATTT

>A2:132383225-132383525|varpos=132383375

CAGTCCAGAAATAAGGTCTTCAAAAAGTATCTGGACTCAGAATGTGCCTGAATGTCTCCTTGGTGTCTCTCTACCTGCCTTGTTNGTTTGAAGTGGACCTNAAGTTGGATGCTTTTAGTAACCTTAGNNNNGGAGTGAGGAATCTAACAGACCAGAANTCAC[ACAAACAGTCAGTCAGTCAGTCA]GTCAGTCAGTTATCTCCATGTGTTTATTTTTATGATTTATTTTTTAATTTTTAGGNTGGAAGNAAAACCCCNTTNGTACTCAAGGACCAATTCTGTTAACTCTATTTTCATATAAGGAAAGTGTTGTATTATAGTTGCTTGCTACCAC

>A2:132786789-132787089|varpos=132786939

TATAAAATGTTTAGCTTTAAGGATGTGAGGATTTAGTAAACCTTTGACCATCTATGTGAATTTGTTCATGTTAAAAGAATGAATCTTGTTTATTCTNATCCTAACAAAATATTCTTGTCATATAANAGCATCATGGGAAAAAGAAAAAA[AAAAACCC]CCAAAAATTTATTGACAAAAATAATCAAATGATAAATGAATAATAATTAATTCTACCTTCAATGACTATATAAAGTTATCATCTAAACTGGGAGACTTTTAAGAAGGAAAGGGATAACTATTAATAATTATAGTAAGGATGAATGTCATT

>A2:133797023-133797323|varpos=133797173

TATTTCATTTTCNTTTAATTCAAATTATTTTCTAATTGCCTTTATGATTCCTTCTTTGATTCANTGACTCTTTAGAGGCATGTTGTTTACTAAACATTTTCAAGTTTTAAAGCATTCTTTAGATATCTTTTGTTATTGGTTTCTTTTTT[TTTAAA]AATGTTTTTAACTTNATTTATTTACTTTGAGAAAAANAATNAGCTAATACCAGCGGGGGAGGAATAGAGAGAAGACAGAATCCCAAGCAGGCTCAGCANCATCAGTGCGCAGCTCAGTGTGGGGCTCAAACTTACAAACCCTGAGATCAT

>A2:134893450-134893750|varpos=134893600

AGTCGGTTAAGCGTCTGACTCTTGATTTTAGCTCAGGTCATGATCCCAGGGTTGNTGAGATCAAACCTTGCATTAACTGCTTAAGATTCCCTCTCTCTTTCTCCCTCTGCCCCTNCCCCCANGCACTCACTCACTCGCACTCTCTCTCTCTCTC[TCTCTCTCTAAAAAAAAAA]AAANNAAAGTTCTTATTGCCCTTTCAACTTCCCAGCATCTTGGCTTTCNGATAAGAAATGTGTACATGTTGCGTGGAGGACAGCTTTACGACCTGTAGACCCCTNGTCTTAATCAGTTTGGGCTACCATAACAAGACACCAGAAATTGGA

>A2:138075716-138076016|varpos=138075866

CCTAATTTCTAAACTTCTGGTGGAAAGAAACTTAGCTGTTACTCATTTACAGTCCATCATTTTCTACATGTAAGACTTTTGGGGGCTTACTGATAGTTACACAATTAGCAGCAGCATTTTTGTTATTTCATTTTTCACTNNNNNNNNNN[TTTTTCC]CTCTCAAGACCATAAAACATTATCTAGGAATATGTAATAATAGCTATCTGGANATTACTGCTGATACTACAGAGTAAGAAAAGGAATAAACTTTNNNNNNATTTCTCTGCTAGTTCTGCTCTCTGTGACTCACAGTACATTTCTCAGCAT

>A2:138263766-138264066|varpos=138263916

AATTATTATTTGATAACTACCAACACCCACACTAGGATTCTTCCCCTGCNGAATTAATCTCTCTACTTTAATTNTTTGCTTTTTGTTCCANTGTTGGAGTGNAATTATTTCAATGTTAAAGGAAGNAAGATCTGTAATATTGCNTGTTGTATN[TCACACACACACACACACACACACACACACACACACACA]TCATATGAAGTTGGGAAATTGGGGCAGGATTCTAGCTTCTGACTCAGGGACACTGAATGANTANGTATTTTCTTTGTACTCATAGGCCAANNGGAGACTAAGTCAGTTGGTC

>A2:139068540-139068840|varpos=139068690

AAGTTAACTGGTAGGGAAACAGCTAANTGAAAGACAGTGTTAAGAAATGTTGTACGGGTTATCATCCTAATGCTGAGTATTCTCGGTGTCCTCTCTGTGTCACAAAAAGGAAGAAATTCAATAGATACACGCTAGCTAGCTGTAGAGTT[TAAAAAAAAAA]NNGAGAGAGAGAGAGAGAGAGAGAACAAAAGATCGCTCAGGGCAACGGCCACCTGTGCAGGTATTNGGCAGTAGGTCCTATANATCTTCCAAGAGATGGTACTTCCAGGTCACTTACAGACGTCTGTTCTGCTNAAGTCTGAGGCAAGGT

>A2:139197719-139198019|varpos=139197869

ATAGGCTAGATGAAACTTTAGTTATAAAACAACCTGAAAAATAGCAGAACCCCAAAGTAACACAGCTTTACTGCTTTCTCCCACATGTCCATCACAGGTTGGTAATGGCTTTGCTTATTGTGGTCACTCAAGAACCGAAAGGAAAAAAA[AATTTTTTTTT]TTTTTTTGAGGTCTGGCATTGTCACACAGCCCACAGCTATCACATCGTACATCTGTCAACAACTCGTTGGCCAGATGTGGTCATAGCCTCATCCAAACACAAAGAAGCAAAGAAGCATAAATGTACAAAGTGCCCAGAAGGGCAAGAACA

>A2:139360166-139360466|varpos=139360316

ACAAATGTACCACTTTGGTGGTGANNNNNNNNNAATAGGAGGGGTTATGTATGTGTGGGAGCAAGGGATATATAGGAAATCCCTATACCTTCCTCTTAATTTTGCAGTAAAACTTAAAACTGCTCTTAAAAAGGCATTTAATTTTTTTT[TTTTTTTAAAA]AAAGGAGAGAAAATAGTAACTATGTGAAGTGACAGGGATGTTAGTTAGCTTGATTTTGGCAATCATTTCCCTATATATGTATTAGCAGAANACTAAGTTGAATATCTCAAATATATACAATTTTCATCAATTATATGTCANTAAAGCTGA

>A2:140271692-140271992|varpos=140271842

ATCTTGCAGTTCATGGGTTCAAGCCTCGTGTCAGGCTCTGTGCTGACAGCTCAGAGCCGGAGNCTGCTTCAGATTCTGTGTNNNCTCCCTCTCTCTGCCCTNCCCCTGCTCACNTTCTCTCTTCTCTTGTGCTCAAAAATAAACATTAAAAAAA[AATTTTTTTT]TTTTAAGATGCAATAAGGTATTAAAAGTACTGTAGTTTCAGTGTAGCTCATGAATGTAAACAGTATTTCAAGATAGCTGCAATGAAATATGTGATAGGAAGAAACATGTATTTTNATTGGAGATAAAGCCACAGATAATAGCCAGTACTA

>A2:140438263-140438563|varpos=140438413

AGGGGGAATTAAGCTTCTTATTTTTAATTCGTTTTTACCCTTAGAGGTACAATATTCCAATGTTTAATTTACACCAGACTTGTGTTGCCCTGATCCCATTTNNTTTCTAGATACTTGGATTTTCACATTCTCTCTCTCTTTCTCATANN[CTGTGTGTGTGTGTGTGTGTGTGTGTGTGTG]NNNACTAATTTACTTCTCCATTCATTAACACATTCTTCCATTAAATATGTTGCTATTTCATTCAATCAAGTTACCATTCACCAGTGGAGCCTTTCACTCTCTTTGAGGCAAATCTGNTTACTGTTGTTTTATACATTGA

>A2:140904801-140905101|varpos=140904951

TTGTTATGTTGTATACCTGAAACNAATATAATGTTACGTGTCAATGATACCTCAATAAAAACTTTTTAATTTTGAAAAAATAAATAGCACTAAATGTATGGTGTATTGGAGTTGGTAAGACTCTAATTCAAAGAATTTTAGTATTTTTT[TGAGAGAGAGAGAGAGAGAGAGAGAGAGAGAGA]NNNNNNCANAATGTGCAAGAGTGAGCAGGGAAGGGGCAGAGNNNNNNNNNNAGAGGATGTTAAACAGGCTCCATGCCTAGCACAGTTTGATGTGGGGCTCAATCTCACAACTGTGAGATCATGACCTGAGCTGA

>A2:141353521-141353821|varpos=141353671

TAATGTTCATGTGATGAGAACACAAGGAGAAGGAGGGAAAGAAACAGANGAAATATTTGAAACAATGATGGATTTACNCATATTAGTATCAGACATCAAACCACAGATCCAGTAATCTCAGAGAACATCAAACAATAAATGTAATAATA[GTAATAATAATAATAATAATAATAATAATAATAA]AATAATAATAGCAATAATCAAGTTAGGAATATTATATTCAAACTACAGAAAATCAAAGGTAANGAAAAAAAATCTTTAAAGAAGTTACAGGGANNNNNNTACCTTACTTACAGAGGAACCAAGATGAGAATT

>A2:142226286-142226586|varpos=142226436

AAGATTTTAAATGGTAAATGTTTTTCCTGGNNCACCTGAGTGGCTNGGTNAAGCATCNGATTTTGGCTCAGGNTGATCTAACAGTTCGGGACGTGGAACCCCACATCTGGCTGTGCNCTGTGCAGAGCCTGCTTCAGATCCTCTGTTTC[CCTCTCTCTC]NCTCTNTCTCTCTCTCANNNNNNNNNNNNCATTAAAAAATATATATTTCTCCTGGAAGTAGTTAGATTTGGAATTACTATTAGTACACAATGTTTTATATCCTTTTGCTTTGNTCAAAGCAACAAAAATATATAAAACATTTGACAAGT

>A2:146836598-146836898|varpos=146836748

CCTACAGCCCAGCAGTGTCCACCAAGGACAGTCTGGTGCTTTACTTTTGANAACTCTATTCTCATTGCTGCACGAAACACAGACCAAGATCCGGTTTTTGATCTNAGGNNAGTCAATTAAAATATTTTTTAATGTTCATTTTTTTATTT[TGAGAGAGAGAGAGAGAGAGAGAGAGAGAG]CAGGGGAAGNGCAGAGAGAGATGGAGACAGAAAATNCCAAGCAGACTNCGNGCTGTCAGTGCAGAGCCCNATGCAGGGNTTAATCTCACAAACTGTGAGCTCATGCCCTAAGCCGANATCA

>A2:148773224-148773524|varpos=148773374

GATGGGGGAAAGGATGGACAGATAAGTTCTCTTTTCTNTAATACTGGATGCAAAGAAAGCCCAGATCAGAANTCAGAAATGGTCTTCCCCGTGCCGGAATGTGTAGCCCTTGTGAAGGATAAATGAGACACNTGGATCCCTACACGTNN[AAAAAAACAAAAAACAAAAAACAAAAAACA]CAAAAAACAAAAAACACAACAGACAACTNTGGATTGATTAATGCTCAGAAANTAATTGCACANGACTAACTTATGATGTTAAAAGCTTATAAAACTGATGTGCTATATATATCATTTCATATCANGGTAAGAATATAATGGCTA

>A2:150168897-150169197|varpos=150169047

CTCGGCTTTGCCGCTCGCGTCTCCTGAGCACTGCCTCGCGCATCCGGAGGGTTGATGCCTTGCTTCCTTTTTGCGNGTTTCCCTCTGCCCAGCTGCNCAGCAAGTAAGCTCACACCTCTTGGTCCTACTTACGAAGGGCAGGGGGATNA[TAAAAAAAAAAAAA]AAAAAAACAAAGATTCTGGAGCCCACTGACTTCCCTTAATTCTTCTCTTTGAGACATGACACNCCATCTCCTGATAGACCCAGATGTCATTTGGGGAGGTCAAATTAGATAGGGTGTCAGAACAAAAAAGCCAGGTGCCAGTGAAGAAGT

>A2:152640890-152641190|varpos=152641040

TTNCCAATTACAACTTGTCTTGGGGTCTATCTGCTTTTGTTGATTCTGATGCAAGTTCTCTGTGNCTCCTGGATCTGNNTATCTGTTTCCTTCCCCAGATTAATGAAAGTTTTCAGCTTTTATTTNTTCAANTATATTTTATNCNNNCC[CCCTTT]TTCTCTCTTGCTCTTCTGGGACTCCTATAATGTGAATGTCATTATGTTTGATGAGANTCACTGAGTTCCCTAANACTATTCTTGTTTTGCNTAATTCTTTGCTCTCTCTTTGTTCAACTTGATTGCTTTCCATTAATTTGTCTTCTAGGT

>A2:153113445-153113745|varpos=153113595

ATACTAAGAAAGATTTATTGATCAAAGGTGGATAACNCTGACTTGGTATTTATCATNCATCCAAAATGTAAAACAGTCCACATGAGGTTATTTCAATATNCTAAGGTAAGCAGGGANATAAGTAAGTAGATATTTAAAATGTTATTGNA[AAAATAAATAAATAAATAAATAAATAAATAAATAAATAAATAAATAAA]ATGCTATNGTAGAAAGCTGTTTGTGAATACATTAAAATGAGGAAAATACNTATCAAAAAGAAAACACATTACCAAAACTGAATTCCCTTTATTTTTCAATTATGGAG

>A2:153311725-153312025|varpos=153311875

CTGGTTGAAAAACAATACCTTGAAAGTGATGGCTTAAATTTGCCATGGCAGAAATAGAATATTCAGGTGAAATGATTATAATTGATTATCAAAGGACATTTCTTTGATCTTTGATTATTTGCTGTTTGTAATGCAGGCCTGAAAAAAAA[AATTTT]TTTAAGGNTAAAATTGTTTGAATTGATTGTTACAGTTTTAATATACTTTACCAACCAATATATTGATTGGGAAATATNATCAAATTCAAAACNCAAATCCATACTTGTGCTAGGGCATTGNCTAAAGTTGACNATTCATATTGTAGTTCA

>A2:153803437-153803737|varpos=153803587

TGAGGTTAAACACCACCCCNGTTTTTNTGTTAAGATGCTGAGAAAACTTCAGACACCATGACCTATGAGGCTTATGTGGAGGATATTTTGGTGCACTGTTTTGTTTTGTCTTAAAGATNTTATTATTTTTATTAATGTCTATTTATTTN[TGAGAGAGAGAGAGAGAGAGAGA]NNNNNNATGAACAACTGGGGGAGGGGCAGAGAGAGAGNGGGGAACAGAGGATCCAAAGCAGGTTCTGTGCTGACAGCAGAGAGCCTGNTGCGGNGCTCAGACTCNCAAACCGTGAGATCATGACCTGAGCTGAAGTCAGACG

>A2:154513221-154513521|varpos=154513371

AAAAAAAAGGTTAGATTATTCTAAAGAAGGAGATNATCTGGCCATTTTTTATGNCCTAAATTTTTATGCCCATTGTTTTTTTGAGGTTTTAGTAACGGTCTTAAGTGTTCCAAATTAAGTCTAGACGGAGTGTTCTGAATTGCCANNNN[CAAACAAAAAA]AAGAAATCCANACATCTTAATCACNGACTATCCTTTGCCTGAATGGCANGTCTTTCCATTTGNTTTTCCTGAGAGTAGAGCTTAATATTTGGTCCNGTTTTTAGTTTGCTGATCACGCCTATAGTGGAAATACCCCCATGTCAATTT

>A2:155412665-155412965|varpos=155412815

CTTCTATCAAACCTTTAAAGAAAATACAGTAAGTNATTAATATGTGAAAGCATATAAAAAAGGAAAATCTTCCAAGTTCATTTCATAAAATTAGCAAAATCCAGATATCCAAACTTGACAAAAAGAATACAAAATNNNNNNNNNNNCAA[AAAACACACACACACA]ACACACAGACACANNNNAATAATCCAAGCTGTGNGGATAGAAAACCTCTTAAATTCTATTTTCCCTCTTCCTTTCAGTTCCCTCTCCCTCTCTCACTCTTGTTCTTTCCCTCAAATTAATGTTAGTCATTCATCAGGAATCCAGTAAAAT

>A2:155969935-155970235|varpos=155970085

CAAAGGCATATGAAGGAAACACTACAGAGTGGNGAGCATNGGAGGGGCAGATGTGAGTAATTCTGGTTAGAAGGATCAGGAAGAGGATCATGAAAGCTCAGAGGAAGTGAACTTTGAGAACCATAACTTTTTGAAGCTGGAAAAAACCT[GTGTTGTTGTTGTTGTTGTTGTTGTTGTTGTTGTTGTT]NNTTGTTGTTTCTAATGCNTATAAAGCAAGGAGATGGCTTCTCCTCAAGGGTCACNTTGGCTCCAAGACTCTTTGATGTGACACAGCTCCCTCTTAGATNTGAGGATTGGGCCCT

>A2:156903719-156904019|varpos=156903869

TACTTTGAANGGAATGAATGAATATAATCCGGTTAAGAAATTTACTCTATCTTCCAATATGGAAATAAACAATTTCATATTTTAATTTCAAGTGATTAAAGTCTTTAAACTATATTTTGTTTGAATCTGAATGTTAATATACCATGATT[TAAAAAAAAAAA]AAAAAAATCATCTCCCATCTCACACTGTATCCTCTTGGGTTTATAATAAATAAAAANNNNGAGATCTCATCTNTAAGTTTTATCTCCTTACGTGATTGCTACTTTAGCTACACAAAGACTCAGATGAAGCCTGTAAGTTACTGCTTATAAGAGCC

>A2:158901319-158901619|varpos=158901469

GCTATTATTTGGTCNCATAAACNTTCCACCCCTTTTTCTTTCTCTTCATCTTCTGGGACCCCTATGATTCTGATTTTGTTCCTTTTAACGAGTCACTGAGTTCTTTAATTCTTANATNGTGCCATTTTGCCTTAGTCTCCCTNNNNNNN[TTTTTCTTCTTC]CTTCATTATTCTCCATAAGTTTTTCCTCTTTATTACTGATTTGCTNTCTGCTTCATCNATCCTTGCTGCTGTGTNCTATTCCAGATTGCAGCTCAGTTATAGCATTTTTNATTTCATCATGACTAGCTTTTACTTCTTTTATCTCTGC

>A2:159160889-159161189|varpos=159161039

GGTGAGTTCGGGCCNNNCNTTGGACTCTGTGCTNANAGCTCAGAGCCTGGAGCCTGCTTCGGATTCTGTGTCTCCCTCTCTCTCTGCCCCTCCCCCACANGCACTCTGTCTCTCTCTGCCTCTCAAAAATAAAGNAAANNNNNAAAAAA[AAATTTTTTTT]TTTTTAAATACCACCATTGGGGTGCCTGGGTGGCTTAGTTGGTTAAGTGTCTGACTTTGGCCAGGTCATGATCTCACAATTTGTGGGTTTGAGCCCCATGTCGGGCTCTGTGCTGACAGCTNAGTTGGAGTCTACTTCAGATTCTATGTC

>A2:159266914-159267214|varpos=159267064

AGTCAAAATATNATAGAAGTGANNNNNNTAGAGTTTAGAACACTTTCCAGATTATNATGTATACAAGAANAGAAATCATCCTCACTTCTCTTTGGNCTAATTAGACNGTATTTTATTTATTTNTNTTTTATTATTTTAGAGACAGGGGG[AAGAGAGAGAGAGAGAGAGAGAGAGA]ATCCCAAGCAGACTCTGCACNATCAGCACAGAGCCCAANGTGGGGCTTGATCTCATGAACCTTGAGGTCATGTTCTGAGCCAAAATCAAGAATCCCGTGCTTAACTGACTGAACCATCCAGGTGCCCCGAGAGTG

>A2:163872733-163873033|varpos=163872883

TTACATTATTCTATTCTATACATCCCTTTTTAANTTTCATTGTTATAGCCAGTGAAAGACAGCTTTAAAATTCAAGCAANAAATTGTAAAAAGTTCTCAAAAATTCCTTTAANTTCAATTTTCATTAAGATACGCCCCAGTTCTATTAAAA[AAAAAACAAAAAAAAAACAAA]NNCAAACAAACTTGAGAATTTGTGGTCTTAATCCTCTAATTACAACTCATAGCCTCAATATTAGCATCTTGACTGTCCAAAACCTCTNTAAGAAGCAAAAGTCGTCACAATAACTCTAAAANNNTGTCTAAATCATTANCAAATGGATCTCCC

>A3:6601944-6602244|varpos=6602094

CTGAGCCACCCAGGTGCCCCTGGNAATTCTATTTTTAATTTTTTGAGGACATGCCTTACTGTTTTCCATAGCAGCTTACCATTTTACCNCCAACAGAGCTTAAAGGTTCCAATTTCTCCACATCCTTGCTAACACTTGTTATTTTCTTT[CTTTCTTTTTTTTTTT]NNNNCCCTTATAGTAGCCCTCCTAATGAGTGTGAGGTGACCCATTTAATTTTTAATTTTAATTTTTGACAAGGTTATCCAGTCCTTTGTAGCCAAACAAGAAATGATGAAAACATCAGTAGTGGAAAAATATTTNTCCTGCCCTGA

>A3:7889704-7890004|varpos=7889854

NCGNGGTCTGTGAGTTTGAGCCCCGTGACGGGCTCTGTGCCCACAGCTCGGAGCCTGGAGCCTGCTTCCGATTCTGTGTCTCCCCCNCTCTCTGCCCCTCCCCCACTCGTACTCTGTCTCTCTCTCAAAGACAAATAAAACATTAAAAA[AATTTTTTTT]TTTTTTAAAAAGAAAACATTGTAAAAAACACATTATGGGACTGGAAGATGTAATTATAAAAGGAAACACAAGGGACCCTTTGGTGATGGCATCGTTCTGTATCTTAACTAAGGTGGGTGGGTACAGAAACCCACAGGTGATCAAATTGCA

>A3:8089053-8089353|varpos=8089203

AAGTTTGTTTCTTTCTATTTAATAATTGTATGTGTGCCTGTGCCCACNAAATATACATACATGTGTNGTATTTTTTAAGAAAGTATACAAATAGGATCATACTATACACATTTTATGCACCTTTNNNNNNNCACCCAAAGCTCTATTTTGGAG[ATTTTTTTTTTTTT]CATCTAAATACACACAGACTCACGTTACCCTTTTTGAAGCTTCCACCCTTGACATTACAACTATGGAAACGTCCTGCTCTGACACACATCCTTCTTTGTTCTCTNNCCATGGGAAAAAGGAGTTATACCACCAAGGTTTCT

>A3:8674097-8674397|varpos=8674247

TCTCACTGACATGTGAAGACTATCCCCCAAATATTAATTAAAACAATTGTGTTTCNATCTCAACTTCTTTGAAAAAAGACTCAGCTTCTTGGGTTCAGGGTCAGGTCTTACACTGTATTGCTTGTATAAACTTTANNNNNNNNNNNNNN[CTCTCTCTTTTTTTTTTTT]NTTTTTTTTAAGATTTTGTTTTTAAGTAATTTCTACACCCAATATGGGTGTATCTCAGACTCACAACCCTGAGNTCAAGAGTCATGTGTTTTTCCAACTGAGCCAGCCAAACGCCCCTATAAACTTTATTTTCAAGAAAAAATNTATATG

>A3:15140526-15140826|varpos=15140676

CACTCTTTCACCACTGAATATGATGATAGTGGTGTATTTTTCATAGATANCNTTAATCATGTTGAGGAAGTTCTNTCTGTTGCTAGTTTCATGAATGTGGTGCATTTGTAAAATGTCTCTTCTGTTTCAGTTGAGAGTATCATGTAATN[TCCCCCCCCCC]TTCTTTATATTNATGCNTTTGATTACATATCACTGAAATAGATAAAAGTTTCATAGTTTTTAAAAATAAGCTATATCTGTGTACTGAAGGAGACTGTATTTGGAGTTTAGAATATCAAAACAAGGTNCAAATCTTCACTGCTAT

>A3:17124019-17124319|varpos=17124169

CTGTTAAATTCCATAAATGAGTGAAATCCTAGATTTGTCCTTCTCTNNNGACTTATTTTGCTTGGCATAATANACTCTAGTTCCATCCNTGTTGTGACAAATGGTAAGATTTCATTCATTTTGATGGCTGAGTAATATTCCGTGTGTGTGTACG[TGTGTGTACGT]NNATATACACACGTGTACACACACAAACACCACATCATCTTTATCCATTCGTTAGTCGATGGACATTTGGGTGAGAGAGAAGCAATTTTCAAGAGGAGCTGGAAAAAGCAGATTTGTCAGGTGTGAACTTCGCNTGTAGGGGTTGAGTCC

>A3:18850575-18850875|varpos=18850725

AGGCCTTTTCAAGTTTTGATGGCTATTGCTAAATTGACCTCCATACAAATTTCACCAATTTATACTCTCCTATTGCATGAAAATGACACAGTCTGTTTTTCTGATTTTTGTCAATCTGTGAAGAAGGAGGANGAGCCTCATTTAAAAAA[AAATTTTTTTT]TTTTTTTAATGTTATTTTNNNNNNNNNNNNNNTGAGCGAGCACNAGAGGCTGAGGGGTAGAGGGAGAGGGAGACGCAGAATCCAAAGCAGCCTCCAGGCTCTGAGCTGTCAGCACAGAGCCCAGTGCAGGCCCAAACTTAGGAACCATGA

>A3:19454376-19454676|varpos=19454526

GAAGTCCTTCCAACACTCAGACAATGTCAGAGGCCCGTGAGGGAGAAGGAACAGAGCCNNGGTCAATTCTCTTCCCAGACTCAGGTGATAGCATGAAGCCTGACCTGTAGAAGGGTGCTTGGGGTTGGAAGTTATTTTCTTTCCAGACT[CTTTTTTTTTTTTT]NNTTTTTNAATTAATTAATTTAAATCCAAGTTAGTTAACATTGANCATAATAATGGTTTCAGGAATAGAATTTGGTGATTCACCACTTACATATAACACTCNGTACTCATCCCAACAAGTGCCCTCCTTAAAGTCCATCACCCATCTAGC

>A3:21113899-21114199|varpos=21114049

ANGAAGTACCGGAACCACACAGGTTCAGAAAAGGTATGATCACTGTGGGCCTGGATGGTAAAGTAAGGTAGGTNNNNTCCTCTTTACAAGGCAGAATTTAAAATGAACCAACAAGAGTAAGATGTGGACAGCTGAGAGAGAGAGAGAGAGAGAGAGA[GGAAGGAAG]GAAGAGGAAGAAAACAAAAGGGTAGGTGAAGATGGGGAAGATCATGATGGAGTCTTAAGGAAATGTTTAATTTAGGAGTCTGACCAATGAGCAGGGCTCAGGCAGAATTAGCAGCAGCTAATCTGGCTAGGGGCCAGATGACAGCCAG

>A3:23211954-23212254|varpos=23212104

TGACTTCTTAACCTGGAAAATTCAGGAGAATGGAACACATATAAATATATTTTAAACTTAACCTGGGCCCTGTGGGTGGGTTCAAGGATATCTGGAAACCACCTGAAATTATATGCAGATGTACNAAAATTCCAGAAGAGATACTGTAA[CTTTTTTTTTT]ATTTTTTTTCAGATTCTGGGGGCTTACTCACCCATACAGTTCAGAATAACTGAATCTAATTTTTGGATTTGATGTCTAAGGTCCTTTCCATTCTAACATTCCATAATTCTAGCAGATCTCCAAGATCTCTCCATTTCTAATATTTCATT

>A3:27281071-27281371|varpos=27281221

GGTCATGATCTCANGGTTGGTGGGTTCAAGCCCCACAGGGGACTCTGTGCTAACAGCGAGGAGCCTGCTTGGGATTCTCTCTCCCTCCCTCTCTGTCCCTCCCCTGCTCATGCTCTCTCTCTGAAATACATAAACTTTAAAAAATAAAT[TATAAATAAATAAATAAATAAATAAATAAATAAATAAA]NNNNNATAGTATGCNAAAGCAAAAAGTTGTGTTTCCCTTTTTTCCCTTCCTCTATGTGCCCTAGATTTTTGGATTTCAGGGGTGTACAGAGATTTTCTTAGCCTGCCAGGGACTTTCATCAACATCCCTAACACTGCA

>A3:28862203-28862503|varpos=28862353

GTGCTTCTGTCCAGGTGTCTTCCCAACATATTCTTAGACATATTCTTATTCTTACAGTTCATTGATTCTCTCTNCATCTCTATCTAAAGTTCCTAAAATCAGTTACTGTATCTCCAGTTTCTGTTAATATTTTCTTCTNNNNNNNNNAA[GAGAGACAGAGACAGAGACAGAGACAGAGACAGAGA]GNCAGAGACAGAGAAAGAGAGAGAATGCACTCGTGAACACGGCAAGGGCACCGANTGGGGAAGAGAGAGAATCCTAAGCTGGCTCCACACCCGAAGNGTGGCTCACAACCATGAGATCATGACCTGAGCAGAAATCAAGGGTCAGAT

>A3:31878154-31878454|varpos=31878304

TTAAGTGGCAAAGCNGTAAAAAGTGTGNCTGTACCCCTATCTCATTCCATGTTTATTGTAAGCCCTGGTTCCTAAGGAAAATATGTTTTCCTCTGTCATCCTGCTTTGAAAGAATTCCTTAGAAACGCTCCTGCCAANTGGTTTTTTTT[TTTTC]CTTAGTACATCGAGATCTAAATGTGAAAGGCAAAATAATAAAAAGTTTGTCAAATAATGAGAACAAAGAAAGGTAAGTCTGAATATAGGAACTGGCTGTGGGCCAATTCACAATAATGAAAAGACTTTTCTTGATGAGTAATAAACTCAG

>A3:33105923-33106223|varpos=33106073

TTCAATGAAAATGTATTAGATCTACTGTTTTCAGTGTTTTGAAATTTAGACACGTAGGGCATATACATTACTNNNCTCTCTCCCTCTCTCTCTGTCTCTCCCTCTCTCTCTGTCTTTCTCTNTNTCNNTCTATATACATAGTACATAGT[GCACACACACACACACACA]CACNNNNACACAGTTGTAGTATATACGTATGCTAGTATACTATACTATANNNNNNNNNNNNNNNNNNNTATTAGCTATATAGCATAATAACTATATACTATACAATACTATAGTTTGTACAAAAATGAATATAACCTTTTATATATACACACACACTATGTATG

>A3:33205208-33205508|varpos=33205358

GGAAAGGGAGTCTAGAGCACAATCACATATAAGCATGTGTAGTTAATATATTTTTAGCATCTATAAGGAAATAAGTTTTTTTTTCCCTTGGACAAAAGAGTCTGTATGATATAATCTGTTTCCACTTTTTCAATAAAAATATGTTGGNT[CTTTTTTTTTTTTTT]AACACTTTTACCTTCTTTCTATTTCTAAAATTGTCCAAAGGCAATGTGATTTTTGTAAAATGTCCTAANGNTTCAAAATCATTCTACTGCCAATGTCTAATTCTAAACCTGATGTTCTGGAGAAATGTGTGGCCTAAACCA

>A3:34240094-34240394|varpos=34240244

CCTGCCTGGTAGCCCCCCTGTACTTANAAACAATGGGATGCCTACCAGAGTACAATGGATTGGACTTCCCTGAGATTATACTNNTGTTCCTGCCTCTACTTTTTTCTAGATTCAAAAAGTTTATGAGGAAAGAAATATAAGACCAAGCA[TCACACACACACACACACACACACACACACACACACACA]NNNNNNNTTTCATAGTGGACATTTGCAGTTTGGCCAGATATGCNTGGTGGATTACATTCCAGAGCCAGATGCTAGAATCAGTACCCACATTGCTACCCCCANGCATCAGACATGTAAC

>A3:34307239-34307539|varpos=34307389

TGTTCAAATTCTTGACCCTTCTCTTCTGTAGGCACCTTCTACCTACTTTCCTTGAACCCTCTATTTGCTCTCCAACTATATACATGCCCCCNTGCATAAGAATGATCTTTTTAAAAAACTCTAAACTTGATCATGCCTGAAATCTTTTT[CTTTTTTTTTTTTTTT]AAGTTTATTTATTTGTTTTGAGAGATGGAGGGAGAGAGAGTGTGTTGGGGAGGGGNNNNNNNNNNNGGAGAGAGAGAATCCCAAGCAGGCTCTACACCATCAACACAGAGCACGATGCAGGNCTCAGTTTCACAAANCGTGAGAT

>A3:34627341-34627641|varpos=34627491

GCCTTCAAATTCTAGCTCNGCTGTCCCCTTCCTTCTGAAGACTTTCTCATCCTACNCTCCACAGGCATGGTTAATTATTTCCTTGTATGTTATTCCTATGTACCTTGTATATACTTCCACTGTANNNNNNNNNNNNNGGTATGGTNNNN[ATTATTTTTTTTTTT]TTTTTTTGANAGAGAGAAAGAGAGAGTGTGTGTGTGNNNNNNNNNAGTGGGAGAGGGACAGAGANAGAGTGGGGGACAGAGAATCCAAAGTGGGCTCCNTACTGACAGCAGCGANCCCAACATGGGCTCAAACCATGAGATCATGACCTGAGCTGAAGT

>A3:35126806-35127106|varpos=35126956

AACTCTCCCTTAGCTGAGTTGTTGGAGAACTTTGCAGCTGCAGAGGAAAGCACCCATGTTTCACTTACATGGACTTTTGTGGGTACCTGACGTTTCCTCTTTGTTTTGAACAGGNCAAGANNTGCTGTCCAACTTCCTCAATTTCATTG[ATTTTTTTTTT]NGCCATTATTAATAATGATATTAAACTTGAAATACANTCAGGTATAAAGTGTAATACCATCCAGTGCATTTTCATACTGAAGTTTNGTTGAATTAAGTACAATTGAGAATGGGTCATCAATAGGACATGGAATTTCACAGTAGCA

>A3:37825135-37825435|varpos=37825285

AAAATGTTAAANGTATTTCCATTTTTAGAAGANTCTCTTGATGTTCTAAGACTTAAAAGGTCAAAGACACAAAGGTCTAATGATGGAATGGNGGGCAGAATTGCTCATGCCTTTCTTTTCGTTTTCTTTTTTTAACATTTATTTATTTC[TGAGAGAGAGAGAGAGAGAGAGAGAGAGAGAGAGAGA]NAGAGAGAGAGCAAGTGAGCAGGGGAGAGATAGAGAAGGAGACANAGAATCCAAAGGAGGCTCCAGGCTCTGAGATGTCAGCACAGAGCCCGATGCAGAACCCACGTACTCTGAGATCATGACCCAAGCCGA

>A3:38524816-38525116|varpos=38524966

CTGGCTCTTGATTTCAGCCCAGGTCATGATTTCACAGTTTGTGAGATCGAGCCCCACATAGGGCTCTGCACTGACATCACAGATTCTGCTTGGGTTTCTCTCTCTCCCTCTCTTTCTTCCCCTCCCCCACTTGCATTTTCTCTCTNTCT[TTCTCTCTCTCTCTCTCTCTCTCTCTC]NCTCTGAAAATAAATAGNNNNNAAAAAAANAACTGGGAATCACAAGAACTAGTTTTGAGACCAGCTCTGCACGTTGCTGTTCCCCTCAGGAACTTGAATTCTCCAATCCAGTTTCTGTGCCTGAAATTCAGATCTA

>A3:40008042-40008342|varpos=40008192

GCATTAATAGATTAGTTTTGCCTGCTTNTGAGATTTATAAAAATAGAGCCATATAATTTATACATGCTTATATNTCAGTTCTTTCACCCAACATTAAATCTATGAGTTTTACCCATATTTTTCATGTAGCAATGCTTTGCTNNNNNNNN[TTTTTAAA]AAGAAATTGCTATTTAATATATCCTACTGTGTGAATATACCATANTTTTTCCATTTTNNNCTTTTTGATGAATATTTGGTTAGTTTCCAGTTTGGAAATATTCTGAATAGAGATTCTGTGTTCTTGTGCATGCTTTTCAGTGNCCTGAAAAGCA

>A3:40063939-40064239|varpos=40064089

TCAGTTGGTTAAGCCTCCAACTCTTGAATTCATCTCAGGTCATGATCTTACGGTTTTTGAGATTGAGACCTTCATGGGACTCCCTGCTGAAGCATGGAGCCTGCTTGGGATTCTCTCCCTCTGNCTCTGCCTCTCCCCTGTGTGCACAN[GCTCTCTCTCTCTCTCTCTC]TCTCTCTCTCTCTCTCTCTCTCAAAATAAATAGACTTTAAAAAGTGTTTAAAATGGTAGTGACTCTGAAGGGATATAATTNAAGTGTATAANAGTATAAATGCTATCCATAAGTTGTCCATACTATTTTNNNATCAAATTCCA

>A3:40340179-40340479|varpos=40340329

GAGCCTATTATTTAACCTTCCAATATTTCTTTAAGAANTATAAATTTTTAAAGAGGCAGGTGTTTTACCTCTAAGAATATCTGTATATTCTTAGAATAGAGAGAGTGGGAATAATGGAGGAATAGAGTTTTATATATATAACTGTGTATAT[ATATATATTTTT]TTTAACATAGTTTTTACAGTATTTGGTTATAAATCTCACATGATGACTGTTATTTTGTGGAATTNTTTTTTCTTATCGATAGAAGGGTCTCCCTTTTAAATTTCCACAGTGTCTATGTTACCACAGAGAGTAAGGACTAAAATTTGAAA

>A3:40936509-40936809|varpos=40936659

CTGTGGCCTCATTATCCTTAGTACACATTTTAGCAGGTACTCACACATAAGAAATAATTTTTATTCACAAANGAAATAGTNGAAAGCTCAGCTCTTCCTGAGCAAGGAAATTCTTTAATATTACTACAAAAATCTGTGTGAGACNTTGG[GAAAAAAAAAAAAAA]CAACTTTAGAGATTGAACTGTATTGTATTACATAATGTATTATGAGTAATATTGATGTGGTACATTTGGTATNATGNAGACATCAGTATCTACCAGAGCAGTACTACTCAAGCTTAAATATCCATATGCATCATGTAGGCATC

>A3:41315469-41315769|varpos=41315619

GCAAGTCTGTATCATACCATAATGTATAAACTTGTCAAATCACTAAGTTGTACACCTGAAGCAAAGGTAACATTGTGTATCAACTATGCTCAAAGAAAATGAATTTGCACATAAAAAAATAAATATAGCTTATTTTATAAATTGAAAAA[AAAAAGAAAGAA]GAACNAGTGGTCTTTTCTGGAATAAACAAAGCAAGATCCTATTTCAATCTTTCAGATTGTCAAAAAGTTATCTGAGATATTCTAAACAANACTACACAGCATATGTGNCTNGTTAGACCTTGTCTATATGAAGTATAGACAGGAAAA

>A3:42210493-42210793|varpos=42210643

GGAACACACTGACCCTTGCTTCCCCTAGATCCAAANNTGGAGTGGGGGTGGGGGAGGGTGGAATATAAAGAATTTCTCTGGNNTAATCCCTTTCCTGTGATTATTTAGAAATCTTGCCATGTTTCTATGCTGTGTGCGATTCCTTTACA[GTTTTTTTTTTTTTTT]CTTGCTTGTTTGTTCATTTGTTTAAGCCTCTTTGAATGCACAGGCATGCATAATGTGAGTTGATATATGCANGTCTTCTTGTCTAAAAAGGAAAAGAAAAATAATAATAGTAATAAGTCAGCTATNAGGATGCCAGTTGCAG

>A3:44624886-44625186|varpos=44625036

GGCTCCAGGTTCTGAGCTATCAGCACAGAGCCCAATGCGGGGCTTGAACTCACAGACTGTGAGACNGTGACCTGAGCTCAAGTTGGATGTTTAACCAACTGAGCTACTGAGGTGCCCCGAAATATGTGNTTTTTATAGTACNTTTTTTN[TTTTAAAAAAAA]AAAGCATGAAACATGTTTACTAATGAATCCAAATTTATCTTTTAGTTTCTCTCTATTAAGGAAGCCAAAAGTAGATAAACTTCTGTTTGGTATTTAGTGTTTCATTATTTTATCTTACTTGGAAATGATGTAGATATTGAATGAATTTAA

>A3:44830706-44831006|varpos=44830856

GTTTCTATTCTTTGATTTCAGCTGTAGCAAGTCTGAAGAAATAACCCTAAATCTGTAAGGAGATANNTATTCAATACTATTCACTAAGCTTAATCATAATACTAAAAATGAACACATAAAATGATATATAAATCAATAANNNNNNAAAA[AAAACCAAA]AATCTCCCACAAGAGAGGGATGGCTAAACTTGTGGCTATCCATGGGATGCCTGTGCAGNAACATTTTAAAAGGTTACAAAGAGTGAAGAGAAGTGGTTATGATATAATTTATTGTGAAAAAAATCAATGCAAAANGAATAGAACTGTGAG

>A3:46980166-46980466|varpos=46980316

TGATCTCACAGTTTGTGGGTTCAAGCCCCATATCGGGCTCAGGGCTGACAGCACAGAGCCTTCTTGGGATTCTCTCTCTCTCCTTCTGTCTCTGCCTCTCCCCCACTTGCATTGTATCTATCTCTCTAAAAATAAATAAATAAANNTTN[TTAAAAAAAA]AAATAAAACTATTTTACTGCACATGAGCTTCCCAGCCCCCATTTCCTGTTTAAGAATGAGATACTCTCAGTGGTTCAGTCATTAAGCATCCAACTTAGGCTCAGGTCATGATCTCACCATTCACGGGATTGAGCCCTGCATCAGGCTCTG

>A3:48447780-48448080|varpos=48447930

TCCCACTTCTGGGTACTTACCCAAAAGTACAAAAACACTAATTCAAAAGGATAGATGCACCTCTATGTTTATAGCAGCATTGTTTGTAATAGCCAAATTGTGGAAGCAGGTAGATTGTCTATCAAGAGATGAATGAATAAATAAGATGT[GACACACACACA]NACACACACACACACACACACACACAATGAAATGTTATTCAGCCATAAAAAAAGAATCTTTCCAATTGCAATGACATGGATAGAGCTACAGAGTATAATGCTAAGGGAAATAAGTCAGANNNNNNAAGANNNCATATGATTTCATTCAT

>A3:53354486-53354786|varpos=53354636

GATGATGATGATGATGACAATGACGATGATTGGTGTAGAGTTATACCAGTTCTTTATATATTTTGGGTACTAACNCTTTATCAGATATGTCATTTTTAATTATCTTCTCCCATTCAGTAGCNTGTCTTTTAGTTTGTTGGTAGTTTTTG[TTTGTTGTTGTTGTT]TGTTGTTCTGTTTCTTGGCCTTTTATGTGTAAGTNTAGGTTTTTTATTTGACATTTTTCTTGCTTATTGAGGTAGGCCTATATTACTATAAACTTCCCTCTTAGAACTGCTTTTGCTGCATCCCAAAGACTTGGACCTTTGNNTGTTCA

>A3:55270472-55270772|varpos=55270622

GGTTCTAGACCACCACAATAAAGTGAATATTGCAATAAAGCAAGTCAAGTGANNTTTTTGATTTCCAGTGCATGTAAAATTACTACTATACTGTGGTCTATTAAGTGTGNNNCAGCAGCATTATGTTAAAAAAAAACAATGTATGTACTTTAAT[TAAAAAAAAAAAA]CATTATTGCTAAAGAATGCTCACCATTATCTGAACTTTCANCAAGTTGTAATCACTGATCACAGATCATCATAACAAATATAATAATGATGAAAATGTTTGAAACATTGTGAGAATTGCCAAAATGAGACACATAGAAATGAA

>A3:55428965-55429265|varpos=55429115

AATTCACTTAAATTCTTGGTTTCCTTCTTTAAAAAATACAGTATAATAATGGAAGCTATTTGTAGGGCTGTTAAAAGATTAATCCAGTTAATATATATAATGCTCAGAATAGTACCTGGGAAATAGTAAGCACCATATAAATGGTAAAA[AAAAAAAAAAATGT]TTATTTAAGGACTCATCTTTGCTGAGTAATGATTTCTTCTTTTAATTTTTTAAGTTAAAAAAANNNNNTATTTTATTTTTAANTAATCTCTACACCCAACATGGGGCTCAAACTCACAACCTGAGATCAAGGGTCATATACTCCACCAACTGAGTCA

>A3:58641072-58641372|varpos=58641222

TAAAACATAAAAAGAAACCTAATTCTCACATCCACTACATGCAAAAATTAACTTAAACCTCANAGTCATAAGCNAAAAGTTAATTCTTCTAGCAGAAAACCTAAGAGAAAAAGTGAGCGAGGCAAACATTTCCTAAATATGAGAAAAAA[AAAACAAAC]CCACAATTCATTAAAGGGAAAAAAAANNNNNNNTGAATTCGTGAAAATTAACATCTCCTAGTAATTGAAAGACACTGTTAATAACATGGAAAAGCAAGCCATAAACTTGAAGAAAACACTTCAAAGTACATATCTAAAAGGGGCATGTATCCCNAATG

>A3:58900791-58901091|varpos=58900941

ATGTTACTGTATTTTGATCATAAGAGCCTTGAAGTTCCTATTGTCCTCCATGTTATGCTTTAGTAATATAGTNCATAGTTAATATCATCATTNTTTGCTTATATGGATTTGATAATACTTTGGGAGATTCAAACTCACTGTNNNNNNNN[TTTCCCC]CCCTTCATGAAGATTCTTGTGACTGAATTCAAGTTTGTAACGCTAAAACCTGTTACAAATGCTTTAGCTTCATCAGTCTTTGTTAGCATAAAAGGTTTGAAAATATGTGTAGAGACAAGTATTTGTGAATAGATGACTGAGNAAAAGAAA

>A3:60719753-60720053|varpos=60719903

CTGACAGTATTANCACAAACTTCAATNTGGCACTAAGATTATTAAATAAGAAAGTTTGGGATAAAGTTATTGGGGATATTAGACATAAATATTTGTAATAGGCATAAAAGTAGGTATTTATTATAGTCTGAGTATTAATTTCTACCCCC[CCCCCCAAC]ACACACACACAAGCATTCATTCTGAAGGTAGACACTTAAAAAAANNNTGATTTCCTAAGAAATATAAAGAAATGTCTTATATGTGGAACCCTTGCACAGAGAGGATTTCTTGGTACCTGGTCTCTCTCAGCTGAGTGTTACATGACCATTACTCTC

>A3:60721057-60721357|varpos=60721207

AGTTCGTGAGTTCGAGCCCTNCATTGGGCTCTGTGCTAACAGCTCAGAGCCTGGAGCCTACTTCAGATTCTGTGTCTCCCTCTCTCTCTACCCCTCCTCCTCACACTCTGTATCTCTCTGTCTCTCAATAATAAATGAACATTAAAAAA[AAAATTTTTTTT]TTTTTTAAACTAGCTACTGATAAATAAAACCCACAAAACCACCTCACCCTGCCCCAGGACTTCACCAGCAATATAACACAGACACTTGAGGAAAGAAATCTGAGAATCTCATCCAAGCAGTGTTACTGGAGCCTTTCTACTTCAAAGCAA

>A3:64169150-64169450|varpos=64169300

ATATACAACAAATCCACAGCTAACATACAGAACAATGAAAAGTTGAAAGCTTTCCCACCAAGGTCAGNAAGAAGACAAGAATTCCCACCCTCACCACTCATATTCAACAAAGTATTGGAAATCTTAGTGACAGCAATCAGGCAAAAAAA[GAAAAAAAAAA]NNNAAAAAAAAAGAAAAGGAAAGAAAAAAAGAAATAAAATACATCCAAATAAGAAAGGAAGAAGTAAAACTGCCTCNNTGTGCAGATGATATAATTTTATATANAGAAAACCCTAAAGATCCCACCAAAAAAACTGTTAGAATAA

>A3:66920994-66921294|varpos=66921144

AGTGGAGGGTTGGGTGCTCTGTGCTCTGCCTAAAACTCTGATTCTAAGGGAGGCGGTGAAAATGGCCTAGCACCTTTCCTTTGTCCTGAAANNTCAGACCAGATCAATGGAACAAAGCAAGTGTTGATTCCACAAGTGCATTATATAGAGGG[CTGTGTGTGTGTGTGTGTGTGTGTGTGTGTGT]ATTTTAATAGCCAAACTAGTGCAACTGTCCATGTATTTTGATCAGTAACAGAAGGGAGAGTTGAAATGGTATCTCCATTCCAATCAGTGACCCNCTCAGCCTTTTTTAAATAGATCCAC

>A3:70027561-70027861|varpos=70027711

TCTGAAGCAGGCTCCAGTCTCCGAGCTGTGAGCACAGAGCCTGATGTGGGGCTAGAACCCACNAACTGTGAGATCACAACCTGAGCTGAAGTCTTTNGCTTAACCAACTGAGCCACCCAGGCACCTGTAAAATAAATAATTCTTTTTTT[TTTTTAA]ATTTAATTTATTTTTTAAATTTACATTCAAGTTAATTATCATATAGTGCAACAATGATCTCAGGAGTAGATTCCTTAATGCCCCTTACCCATTTAGCCCATCCCCTCTCCCACAACCCCTCCAGTAANNCTCTGTTTGTTCTCCATATTT

>A3:70509123-70509423|varpos=70509273

TCTCTCTCTCTCTCTCTCTCTCTCTGTCTCGTGAGTATGCATCCAGGGCTCAGGGTTTAGGCACTTTTTACTTTGTTTCTGCAGNATAATACCTAGCTTGGTGATTGGAGCTAAGTAATTTTTATTTGAATGCTATGNTGATTTTTTTT[TTCCC]CCTCCCCTCTCACCCTTTTCTGGTNCCTTGGGTCTCTTATATCTAGTACAGTATTTGCCTGTATTTAGTTTTGGCTTCTTTCTTTGATATTCCAAGTCCTGTTTTCTCTCCTTGCCCTTTGCATTTTGATCAAGCTCATCCTACTCTTTT

>A3:70809444-70809744|varpos=70809594

CACAGGTTGTTGTGGGTATAAAAGGAGTTTATACTGCAAAGCACTTAGAGCAGCATCCGACATGTGAAAAATGCTCAATAAATTTTAGGTATTATTTCTATAATTATATATGTTTTCCCCTATGATGATAAGAATAGCTCTTATTTATA[TTATATATATATATATATATA]AATCTTCCNCCACCCAGCCATTTCTACCATCCAGCCATTTGCCCTGCATGATGTGAAGGAGCTCAAAAAAGATACATCNTAAGGGATAAATGAATAAATTTCTCTCNCCTCTCCTCAACTGGTTTATAGCAAGGCTCT

>A3:70888556-70888856|varpos=70888706

GAACATGGTAGTTTCAAGATTTGAACTAACAATCTACATGAACATCTGTGAGTCCAGTGAAGTGAACTGCATTATCCATTTCCACATGGGTTTGAAATCAGAATGAGAAGGCAGGCTCAGAAGCATGGTGAAGCACAGAGACACAAGGG[GAAAAAAAAA]AAAAAANCACAGGGCACATGAAGGAATGCTGGGTTGCTTCCCAACNTTCCTGCCTAACAGGGCTGCTAAAGCAAGCTCTCACCTAAGCCNTCAGCAAGAAGGGCTTCCACTACCACTACTTCCTGGAAAACTCGGACAGCTCAGCAGGGT

>A3:72082598-72082898|varpos=72082748

NAATNGTATTATGAATTGTGTTATAAATTGTATGATTNCAATTATATTGCATATATGGATATATATGACATACACACATACATAAACACATTCCTGGAAGGAAATACACCAGAATGTTACCATTTCTCTGTTGTACAAAAGTCTGTGGG[GGTTTTTTTTT]TTTTTTTGTTTGTTTTCTTTTTGCTTTAAATCTTTTTATATCTTTCAAATTTTCTATAATGAACACTTTTTTATAATCAGGAAAAAGTTAATAATATTAACTTCTTAATTTAGACTCATTTTGCCTTGTGCATGAATTATTCTACATAAA

>A3:72860002-72860302|varpos=72860152

AATATACAAAAATGTAAGTAATAAAGCAAACACTAATATTTGAGATACATTAAAAATATAAAAAAANNNNNTAATATAAAATAGTTGGCCAGTTTCCATTTTACCTTATAAAATGTTTGGAAGTTGTCACTNCCACTTAAACANTAAGGAAAGAAA[AAAAACAAAC]AAACTCTTCTTAGATAGACTACAGAATTGAGGTCACAGGGCAAACGGTCAACCCAACAACACTGGAGACACAGGATACAGACAATCACAACTTACTCCTACCCAAAGCCACTGGTAGGAACATTAAAAGAGTAACTGATGAGTTGCTGGA

>A3:73390964-73391264|varpos=73391114

ATTGATTAGGTTTTAGCTTATGAATTATTATGGAACAGAAACATTCAGCCTATAGCAAGGGCATTATCACTAGCTCTCTCTNAATTTAAGTATTAATAATGTTGCTATGTGACTGNAAAGTACTACATGGTGTTAAAGAATTTTTTTTT[TTTTA]ATGTGGGCTCTGGGTAGAGGGGGTTGCATTTTGAGTTAAATGCAGAAACATTGAAAATACCAAACAAATAACTACTGTTATTTTTCACAAATGTGCTAGGCTGACAGGTTGTTTAATATAAACAGAATAATGTGTGTCCTGACATTTTCA

>A3:73480933-73481233|varpos=73481083

GCTATTCNTGNGTCCTNTTGATTTAACTGAAGAGTAATTAAGCTGGGAATTTAGATATTAGGGTATTTTTTAGTGCACACTCATTTATTCTCACTCTCTCCCTCCATATCTTTAANTAACTCTAAATACTGCACTTTATTTTTTNAAAA[AAATT]TAATGTTTATTTTTGAGAGAGAGCATGAGTGAGGGAAGGGCAGAGAGGGAGATAAGAGAATCTGAAGCAGGCTCCTGGCTCTGAGCTGTCAGCACAGAGCCCANTGCAGGGCTCGAACTCACGAATGGTGGGATCATGTCCTGAGCCGAA

>A3:74762825-74763125|varpos=74762975

GCTGTCAGGAGCCTGACACAACCACAAAATTCAGTAAGCTAATAGTTAAGAACACTTAAATAACTTTGTTAATAGCTAGATACAGCATTCCTTAGCAAGTGTTTCTATNTTAGTGGTTTGTGTGTCTGCNCATGTGCACANNNNNNNNN[ACACACGCACGCACGCACGCAC]NNGCACATATAAAGGAGATTTAACACAAATATGCATATATGAATAACTTTTAAAAATCAGTGATTTCTCAGGGCACCTGGGTGCCTCGGTCGATTGACTATCCTACTCTTGATTTCCACTCAGGTCATGATCTCAGGGTCNGGGGATCA

>A3:74764757-74765057|varpos=74764907

TCCATGGTGCCAATGGACAACCTTTCTTCAAAAAGTAGCGAAATACTAAAAATTTCTCATACTTCAGGCAGTATGCCAGATGAAGTTCATTAAGCTGGGCTCCATATTTCAAAAGTATATTCACAATTCCAAAAAACTCGCAGCTCCAG[CAGAAAGAAAGAAAGAAAGAAAGAAAGAAAGAAAGAAAGAAAGAAAGAAAGAAA]NAGAAAAAAGAAGACAAGTTAGACTTTCATANGAAAAACATTCTTGCAAATAGTTAATAGTCTAATAGCTCTCTTCCTGTTGTTGTAGGCTCTTGTTT

>A3:75295295-75295595|varpos=75295445

GAATGATGTTAAGGATTTGGTTTTGCACATNGCTTTGGTCTTACCTTTGGATTCTCTGCCANAGAAGGATATAGACAAGAAATGATAGCCAAATAGCAAACANAGTAATACTTTCATTAACTGCACAAGGACCNGGTTCAACAATGGGA[TAAAAAAAAAAAAAAA]NNNNGACTACAAGCTAGATTACAGAAACTGACTTAGAGTAGGAGGNCAAAGTGAGCNCTCTTACTTCCCCNGGGATTTCTCTTGAATTCTATACCCTCTCCAAATAGCTGCATGATTATGGATGACTGGGCCATCAGCTCCATCCA

>A3:75428149-75428449|varpos=75428299

NGNTGGCTTTCAAGTTGCATGAACTTTGAAACAAAAACATATAAGAATCCAGGTGCTTAAAACTTCCTCAAACTTAGATACTTCCTTACAACCTTCNTCNNNNNNNNGTCTTTTTAAAGTTTTATTAAAGCAATTTCAGTTNNNAAAAG[AAAAAAGAAG]GGTGCTTTGGCCCCATATTTTGACTNATTATGTTTGAGNGTAAAGCTAGTAAATAAAANNNTGTCATAAGATCCTATTTCCCTTTTCAGACTTAGAAACAAANTTAGAAACTTGGTGTCAGATCTGAGTTTGAATTCTGTTTTCATCACTTACT

>A3:76081251-76081551|varpos=76081401

NNNNNNNNNNNNNNNNNNNNNNNNNNNNNNNNNNNNNNNNNNNNNNNNNNNNNNNNNNNNNNNNNNNNNNNNNNNNCCTCCCTCTCTCTCTGCCCCTCCCCCACTTGCACTCTGTCTNTGTCTCTCAAAAATAAATAAACGTTAAAAAA[AAAATTTTTTTTT]TTTTTTTTAAAGAAATAGCTTCTNNNNNCTCTCCTTACTTCCTTCAACCACTGTGTAGCCCCTCGGGGTAGGGTCCTTAACATGGGTCCATTTTAGTAGCATGATCCANGTATGCTCCTGTATATATACTTTTCTTGGGTGAGGGTCCAT

>A3:76630735-76631035|varpos=76630885

TACTCCTTTAGTNCAAGTTCACATTATTTCTTGCTCTGACAGCATTCTCCCTGTCTGGTTTCCCAAATTTTAGTGTCTCCCCCCAGCTCTCATTCTCTCCNACTTCATCCTCCAGGGCTAACNTCTAAGCTGGGAAAAACTACCAAAAA[GAAACAAACAAA]NAAAATCAGATTAAAAAAAAATCAAATTCAAATCAAATCTACATAAAATTCTCTGAGTTCCCCATTATCTCCTTTCAAGAAATNTTTATACATTTCTATGGTCAAAAAAGTTAGGGAAATAGTATATATCCTATTTCTAGAGATTCAG

>A3:77176165-77176465|varpos=77176315

GGTGAAAAAGGGGAAGGAGTCAAGGAGAAGGAGGTGGTCTTCAGCACNNAGGTGANTGTAGAATTAAAGTATAATTACAAAGTNAAGGAAATGATNGACAATTTTAGGGGGAGAGAAGATAAGTCTGGTTGAGGTCCTGTTTAGTTTGG[GTTTTTTTTTTTTT]TTTNNTTTTAGAAGAAAATGTCTAAGAAGCAGCTGGAAATTCAGGACCAGAGCTTGTAAAAATGAGGGTTGGAGAGGGCATCTTGAGAGCAGTACAAAAAGGAGTGACAGGTGAAACAAAAACAAAGATGGTAAGAACTGTACCTTGGGG

>A3:77637279-77637579|varpos=77637429

CAAATCTCTCCTCAGATTTTGGAATTTCTCAGTCATTATTTCATTAAATNAGCTTTCTGCCTCTCTTATGATACAATGACCCTAGTAANGTGCAGATNGTTTCTTTTAATGGTATTCTATAATTCAAGTAAACTTTCTTTCCTANNTTT[ATTTATTTTTTTTTT]TTTTACNTTTTGCTCCTCTGGCTGCATGATTTCAGATGACCTGTCTTCTACTTCATTAATTCTTCTANATGATTGAGTGTTCTTTTGAAGCTTTCTATTCCNTTCTTCAGTTCAGTCAATGTATTTCTCAACNCTAGGATTTCTGGT

>A3:78661864-78662164|varpos=78662014

TTTAAAATTTGAAACCTATTGCTATAGGGAAATTTCTTCTTCATGAAATGAGCTCANCTCTATTGCCACACAATAAAAGAATTCTCAGCTTTTGAGAGCTTCTGGCTTCCTTATGAAATATGGATAAACTTTGCTTCTGAAGTGTTTTG[GTTTTTTTTTTTTT]NNTTTTTCCTTTCGATATGGAAGAAGAGGAATAAAGAGTAGGTATGNNNNNNTCAGACACAGGAATATCCATGTTTATGAAATAAAGGAGAGNNNNNTTAAACTCTGAAGCTCTAAATATGAGAATTCACTTGAAAATAGTTT

>A3:78752442-78752742|varpos=78752592

GATTATTGTATGCCTTAAGCCTCTGCTACTAAAAGTCTGCTGTGCCAACCAGCTGCACTGGCATCACCTGGAGCTTCTTAGAAACACAGAANCTAAGTCTTNTCTTAGACTTTCTGAATGAAACGTGTATTTTTCATGTTTGTTATTTT[TGAGAGAGAGAGAGAGAGAG]TAAGACAGAGGATCCAAAGCAGACTCTGCACTGACAGAAGAGGCCAGTGTGGGGTTCAAAANNNCTCACAAATCGCAAGATCATGACCTGAACCAAAGTTGAGGCTTAACCACCTGAGCCACCCAGGCTCCCCTGAGATCTGTAT

>A3:79613549-79613849|varpos=79613699

TAATNTGCATGTCANCTNTGACCTAGACAGAGGAATAANATGTAGAGGAGGTACACCAGGTAATTGCTTTCATGGGACAAGCAGAGAAATTCCAGAAATACCTCCAGTGTCTACGACTAGGTATAGAGACTTCAGAATCATTTTTTTTT[TTTGG]GTAGAAGTCANTGAAACCGTAAGGAGAAAAAGACCACATTCNCTAAGAGATAGAATATAGAGCTGGAACAGTTAAAGATGGACCTCGAGTCTATCAGCATTCAGCAAACTCTTTCTGTAAAAGGCCAGACAGTAAATATTTTAGGTTCTG

>A3:80805063-80805363|varpos=80805213

TCCAACACTGGCAGTGTCACCATCCAAAGTGAGCCTTCAGAACTAGCTTGTGTGTGCAGGTNTTGAATGGCCAATGCTGCCATCACCCAGCTAGATCAGACCTTTAGGGAATTCTGCTGGGAGCCAAGCTTGAGAGCCAGTCTTTTNNN[TTTTCCCCC]CCTTAAGTTTTTATTTAAATTCTAGTTAAACATATAGNGGAATATTGGTTTCAGGAGTACAATTTAGTGNTTCATCACTTACATATAATACCCAGTGCTCATCACAAGTGCCCTCCTTACTACCCATCACCCATTTAGCCCACCCTCCCA

>A3:80826477-80826777|varpos=80826627

ACAACATTCTGATCATTCTAATTTTACATTAAGTCTTGAAATAAGGTANTATAAATCCTCCAACTTTGTTTTTCTCCTTCAAAATTGTTTTGGCTATTTTAGGTATTTACATTTCTATATAGATTTTAGAATCAGTTTGTCAATTTCAA[CAACAACAAAAAAAAAA]NNAAAAAATCCTGCCAAGATTTTGNTTGAAAATGAATTAAATTTATTGATCAGTTTGTCTCCCAATTATGTTTTAAGGTCCTGNAGGACAGGAAACTATCTTACATTTCCACGTGGTTCCTGAGTGATGGTCAGATATCCACCTTTCTGT

>A3:83588249-83588549|varpos=83588399

TAACTCACTTANGTCTAATTAGTNTCTTGCTCACTGTCAAGTCTCAAAATTTAAGAAAATTAATNATATTATATTGCTAGGTAANCTAAAAAGCAAGCAGGAAATCAGTATATAAAACAAAGAATCCCACATTAGTTTNNACACATCTGATTTTTTT[TTAAA]AAGATTTTAGTCNNNNNNNNAAAGTCTTTATTTACTTATTCATTTAAGTAATCCCAAAGANGGGGCTCAAACTCACGACTCCAAGATCAAGAGTCACCCACTCATCTAAGTCAGCCATGTGTCCCAAGCGTCTATTTTTAAAAAGCATTA

>A3:83705298-83705598|varpos=83705448

TGCNTCAGGCTCTGTGCTGACAGTTCCGAGCCTGGACCCTGCTTCATGCTTCNGATTCTGTGTCTCCTTCTCTCTCTGTCCCTCCCCAACTTGTGCTCTGTCTCTCTATGTCTCTCAAAAAATAAATAAATNNAAAAAAAAATTTTTTT[TTAAAAAA]AAAAGAAAGAAATGTTAGCAACACAGTGGAGCAGAAAATGGGTGAATNAGAACATAAAACAGAAAGNNGAAAATAGCTTCATTTTGCCTGTTTCATCATCCCATCCCCCAGGGCTGTCATTACTCAGCACCAGGAGGGANCTCCCCAGNTAGAAAC

>A3:83773115-83773415|varpos=83773265

GTTGGTTAAAGCACCTGACTCTTGATTTTGGCTCAGGTCATGATCTTGAGGTTCATGGGATCNAGGCCTACTTCAGGCTCTGTGATGACACCNTGGAGCCTATTTGGGATTCTCTCTCCCTTTCTCTGCCCCTCCCCAGAGGGTGCACC[TTCTCTCTCTCTCTCTCTCTCTCTCTCTCTCTCTCTC]CTCTCTCTCTCTCTCAAAATAAGTAAATTAAAAAAGAACAATAAACAAACAACCACTGAGAGAAAACACTTAATAGGGACACCTTTTGGGATGCCATCAAGGACTAGAGAAAAGGTCTCAGTCCTGTGGGACCCTCTGTTTCTCC

>A3:84710448-84710748|varpos=84710598

AATCTCACAGTTCNTGGGTTNGAGTCCCAAGTCAGGCTCTGTGCTGACAGCTCNGAGCCTGGAGCCTCAGATTCTGTGTCTCCCTCTCTCTCTGCCCCTCTCCTGNTCATGCTCTGTCTCTCTCTTTCTCTCAAAANTAAATAAAACAT[TTTAAAAAAAAACATTAAAAAA]TTAAAAAATGTTTTTTAAATAAAAGAAATGGCTTTAAGGCCCTTCATGATCTGGCCCTGGCTCCCACTCCAGCCCCGAGNTCTAGAAGGTTGTGGTAATCAAAAGCCTGACTTCAGCCTAAGTGGCACAAAACTATACACACGGGGCATG

>A3:86325512-86325812|varpos=86325662

CAGGTAAATTCACCTCATCCTTCGCTGTTATCTCAAGCTGCCTGCCTTGCCTGCCATCTGTGCATCTAAGATTTTGCAGAGGGGAAGCCAGGATCTTTATGCACTGAAGATCTCACTGTTTTTCAACCCTCTGGAACCTGGTTNNNNGT[TTTTTGTTTGTTTGTTTGTTTTGTTT]TTTGTTTTTTAAGGGTCTTTTGGCACTATCTGTTGACTTTGCTCATTTTCTGGCCTCTTCTTGGATTTTTATCTCCCTTCTAGGACCTATGAGTGGAATGGGCATGAATATGGGCATGGAGGGGCAGTGGCACTACATGTAACCTTCAT

>A3:88137609-88137909|varpos=88137759

CCTCCTGCCATGGANTCCAGTCTCCTGTGCACATTTAGGTCAAGTGGTGGCAAGGGGGTGTTGGATACTTGGGGGTCTAGAGAAAATGAAGTTCAGCAATTTATTTCACAGAAGAGGAACTGAAACCAGAGAGGTTAAGCAACCTTATT[CTTTTTTTTTTTTTTT]ATTTTTAAAGCAATCCTTCTTCACCCTCCTTCTTAGTAAGAACTAATTCTGTTGAAGCTGATTTTGANNATTGCATTTAATCATGNTCTCTGGACTATGTGCCTTATTGTCATGAGGCTCTCATTTTACTGATTGATAAA

>A3:89614267-89614567|varpos=89614417

TTAAGCCTTCTTGGTGACTTTAACCAGTCCTTCTGACCTCATCTCCTTCCCCTCTCAGCCCTCTAACCAACTGAACTAATTGTTCTCTTCAAAACCAACCTAGTTCCTTTTAAGGGTCTTTGCATGGGCTGTTTTTCTTTTNNTTTTTT[CTTTTTTTTTTTTTTTT]AACATTTATTCATTTTTGAGAGACAGAGTGTGAGCGGGGGGAGAGGCAGAGAGAGAGGGAGACACAGAATCNGAAGTAGTCTCCAGGCTCTGAGCTGTTAGCACAGAGCCNGGTGCAGGGTTCAAACTCATGAGCTGTGGGATCATGA

>A3:90782455-90782755|varpos=90782605

GCGCGGTTTCCCCTGGTGGTCTTTTCACCATGGTCTTAACCACAGTTGTGTCAAATGTGTGTTAAAAATGCTGCTATTNGAAAAGGTGTGTATGACTCTNNNCCCTTTCTCTAGCATGCTGGGGTGTGTGTGTGTNTGNGAGAGANNGA[GACAGACAGACAGACAGACAGACA]GACAGACAGACAGACACAGAGAGAGACTCNGAGAGAGACAGAAAGGAGAGAGGGAGAGAACAAGAGAGGTTGCATCTGTGATCTGAACTGACAGNTATCCTTGCTGTCTACCAGGGAAGCTGGGGCAGCCATGTGGCTGTGACCTGATG

>A3:92430529-92430829|varpos=92430679

CTCTCCTTGCCTTCAAAACAGCACTGGTGCCTTTGCCACCTTGAACAGCTCTTAACGGCTCTGCTTGCCATGCTTAAACATCTGTGTCTAGAANTTGTCTCANNNAGCCTAATGTTCTATANTTTTCCCATGGCTTTGGANTTAAAAAA[AATTTTTTTTT]TTTTTTTAAGTAGGCTTCATGCACAGCACAGTGCCCGACACAGGGCTTGAACTCACAACCCTGAGATCCCAACCTGAGCTGAGGTTTAGAGTCAGCCACTTGACCAACTGANCCACCCAGGCACCCCTTATATTATTCTTAATTGAACCA

>A3:93926559-93926859|varpos=93926709

NGGCAGCCAGTGTTATCCTGGATACTGGNATGTATCATCCCACCCTGGGAGGCCTCTGCTCCCTNGGGTGCCTGGCTCCTANTGAGTAAAGGTAAGGGGTNGTTTAGTCACTGGTGTGATATCCAGCNCTCTGTGACNTCCATCTCTGA[AAACAACAACAACAACAACAACAACAACAACA]NAAACCACTGGGTTCNCCACTCTGCATCTGAATGGAGTCTACCCTTTGCTCTTGCAAGCTTAAAAAACTAATTGCCATGCATGAGCAAGCCCTTGGAATCTAAAGTCACTTCCTTAAGTCGGAGAGGTTGTACCTCT

>A3:94594948-94595248|varpos=94595098

AATGCTTCATTGCTGTTTCTGTCCTGCTCCAGCCAGACGTGCTGATTTAGTTTAGTTCCTTAAATGTATTGGTCTTTGTCAGATTCAGTTGCAGATGCCTGCAACTCCTCCCTTCTTATATATATTCTTATATATATTCTTATATATAT[ATATTTTTTT]TTTTCCAATTTCAGCTCAAATGTTAGCTCCTCTGGAAGTTATTCCATCACCNCNTTACCATATGCTTCCATATTGCATGACCTTCCATTACCCTTATTCCATTTTTTAGTAATTTCCTGAATAATAAAACTCCATAGAAAATAACTCCA

>A3:95963842-95964142|varpos=95963992

NCAGAATGCACATTCCAANGACATGGGAGGGAGGGAAGAGTCTCAGATTGCCTGGGTCCAGCAAGAGTAATTCACTGGNCACCTCCTCTGGATGACTTCTNCCAANTTGCCCCACCTTAGTTACTAGTAGTAAGAAGCAAGAAGAANNN[GGAAAAAAAA]AAAAAANAGTTAACTATGCCTTAGATATTTAAAACATTTTNAAAAATTGTGTGCAGTATGATTGATATTTTGAGTGTATTTTTAAAGTATNNNNNNNNTCTTTTTTTAATGTTTACTTATTTTTGAGAAAGCGAGAGAGAAAGAGAGTGT

>A3:97073475-97073775|varpos=97073625

AGAATCATACAGCACAGAACATANGATGAACCAGTAATTAGTTTGTGAATTCAGTTACTCTGAGCAGTCTAAGTGCATATTTCAGGCTTTGATTACCCTTCCATCAGGGTTTTTGTTTTTCCTTCCCTTCAATGAAGCAAGCAACTATA[GAAAAAAAAAAAAAAA]NNAAAAACAAAAACAGTATTTTAAAGGTTTATCACACCTGGATAATTGCAATTAGACATGGCTTCNGAGATACGGAGTTAGTTCTCCACAGTAAGANTTACCTAGTATATAAAACAGGTAAATTAGAAGGTNGTTTTGAATGTTA

>A3:97219890-97220190|varpos=97220040

AAACTATACCTTTACATTTTTGTGTTCGATCTTATATCCAGTCACCTTGTTGAAATAACTTTACTAGTTGAGGAACATTGTTCTTCTTGTGTTTTTCTTACCAATTTGTATAACCTCTTTGTACGTAAAGACAACAGTATATGGCAACT[TACACACACACACACACACACACACACACA]NNNNCAGACGTAAATAGGATATAATACTTCTATTTTTCTTTTAGTTTTCTTCTAATTTTAGATATCTTTTCCTATTCACTTAGATTCAGTATGAATGAGTCATTCTTCATATATGAAATACACTATAGTGTGAAC

>A3:98301279-98301579|varpos=98301429

GAAGACAGTTAATCAGTGGACTGGCTGACTATGAAGCACTACTACCTACCTAAATGAATTAAAAACAAANACACAAATTTGTCCAGTTGAAAAAGTTATCTGAAAGCCAATTGGTANATTTCTCAGCAGTGCTNTGGAGATTTTTTTTT[TTTTC]CTTAGATGGCACTGCTTTTCTCCTAAGGCCTTTGCCAACTTAATCTTGAGNCATTACATACAGATTTCATAACAACTGGCCTTCAGCAGTCGGAGAGCACCAGCTGCCTCCCTGGCATCATTCAAGTAGCCCTGCAGAAAAAAANNNNNNTAACAGA

>A3:98460515-98460815|varpos=98460665

TCAACTCTCCAACAGTCTATCATATAGAATGACAGTGTTTTAACTTGGGTGATCTGGAAAACCATTTTAAGATAATAGTCACAGGATCTTTTCNATCCCTTTCCATCCCTTTGCAAAACCCCTCAGGAACTNTGGAAGTTAAAAAAAAA[AAAAT]TGATCCCTAAAAGGTTTTTGCCTACTTTAACTTACAATTCTGATATGCAGGACTGAGGAGTGAGTGAAAGTTCATATAAACCTGGCACTGGCTCTTATTGTATTAGGAAACCTTAAACTTAAAATGGACACTACAAATATTTATGTACAG

>A3:98507444-98507744|varpos=98507594

TGCAAGTTTGAGCCCTGTGTCAGGCTCTGTGCTGTCATCTTGGAGCCTGGAGCTCACTTCAGATTCTGTGTGTGTCTCTCTCTGTGCCCCTTCCCTGCTCGTTCTCTGTCTGTCTGTCTCTCTCAAAAATAAATGCACATTAAAAAAAA[AATTTTTTTTTT]TTTTTTTAAGTAACCGGAGTTCCTTTTACCTCTTTCCTTCCTGGGTCTGAACATGTTGAGGAGTAAAACAGTTAAGAGATTAAAATCGGTTTTGGGGCGCCTCGGTGGCTCAGTCNNTTAAACCTCTNACTTCAGCTCAGGTCATGATCT

>A3:99263123-99263423|varpos=99263273

TTCAAAATGAATTATGGTCAAGAGTGTNTAACNCTTAATTTTTCAGGATACACTAATTGATANAACACATTTGGAGAGGAAAATTTTAAGATCGGAGTCACAAAATCTGGGAATATAAAAGATTAAAAGTGGATAGTAAAAAAGGAAAA[AAAAAAAACCAGAAA]TNCTGGATTAAATTTGGGGAAAAAGTCTTTTATTAACTTTCTTAGTGGCCATTGCTTTGGTTCCAAAACAGTCACTTAGAAGCATANTTTTGTTGGCCTATTGTGAGTCTCTGTTCCCAAATAGTGTGAGNCCAGTGCTCCCTATGG

>A3:100664058-100664358|varpos=100664208

TATTACCCTTAGAATGTAATACCCACACTTTGCAGTGCTAATGCTATTTAATGCTCCTTTCTTCCCTAAACACCTANATAGAGAACTATTTTGGTTTTAAGNCTGGTATTTATCTTAGGTACTTTAATATATCTCTAAAATTGTTTTTT[TTTTAAA]AATATTATTTTGATGGAACAGAAACTGTATTAGTCCATGCNNNNNTAACACCCTATATTTAAGTATNTTATAAGGAAAGGTCTATTATTCCTCCCTACTNTGGGCCTATTCTAGTGCTGCCTAGAAATTTTGCTCACACATTATCATCCT

>A3:100921860-100922160|varpos=100922010

TTTCTAACTTAGATGAGAAGTCTTGATCAGATAATTAGCATAGAAATAAATGAATGTTAGGAAAAGTCAAAGGCCACCACTACACTGTCTTTCTTTCTCTTATCGTTCTTAATAGAGTAAGAAATATTTTAAANTCCAATAAGAACTGT[GTTTTTTTTTTTTTT]TTTTTTTCACTATTCTGCCACCACCCNCCCTGGTTCACCTTACATGTGAGAAAAGAAAAGGGAAAGAAAGGAGAAGGGAGGGAGAAGGAGAATAGGGAGAGGGGTGGAGGGAAGCAAAGAGAAGANNNNNNGGAAAAGGAAAAANTCAGC

>A3:101298340-101298640|varpos=101298490

CGGTCNNTGGGTTCGAGCCCTGCATCGGGCTNTGTGCTGACAGCTCAGAGCCTGGAGCCTGTTTCNGATTCTGTGTCTCCCTCTTTCTCTGACCCTCCCCTGTTCATGCTCTCTCTCTTTCTGTCTCAAAAATAAATAAATGTTAAAAA[AAATTTTTTTT]TTTTTNAAAAGACTNAGAGAGATCTATATTGAAGCCATAACACATCAAGGTGAGTTTGAACAAGTGACTTAAACTCTCTAAGCTTCAGATTTGTCATTTATAAAATGGGGCAATAATAACAGTGCACAGAGGTACTGTGAAGATAAATTA

>A3:101699525-101699825|varpos=101699675

AGATTATGAATTATTGATTAATTNTCAGAATTAGCCAAGAGGCTTAGGAGACAAAAAACATTTAATATTTATTGAAATAATAATATTGATATTACTTAACAATGAATTTTANATGTGTATATNAGGATATATGTGTNTATATGTATGTG[AGTGTGTGTGTGTGTGTGTGTGTGTGT]NTGTNTACATACATTACAGGAAAATATAATTTGACCCATGTTCAAAAAAAAANNNNNNNNTCAAGTCAAATTCATCAAGTCAAGTCAAAATGGAGTCAAGCTGGCTGGCTCAGTCGGTTAAGCATCCGACTTTAGCTCAGGTCATGA

>A3:101760429-101760729|varpos=101760579

CTCATACAGGCCCACAGNNNNNNNNGTGCAAAAGTGACCTCTTATTTCAGATGTCCCAGAGCTGAGGACAAAATATCTGCTATTGCACTNGGTCATTTCAGGAGCACTGGTGCCTAACTGCAGGGTAGAACAGAGGATGGATGGATGGA[TGGATGGATGGATGGATGGACGGATGG]GCGGATGGACAGACAGATGGACAAATGAATGGATGCCCAACCTACCAACTGCATCCTGAGTCCCACTAAAAGCTCAGGGCTGAATCTATTCCAATGTTCCATCAGTCAGACTGCGGAGAATTTGAAACATACTTTACAAGGTTA

>A3:101920584-101920884|varpos=101920734

AGCTGAACCTCTGTCCTGGAGGGGAGAATAGGAACTTCAACANCACACAGCCACGACCAACCAAGTCCAGACAAACAGCTGAGCCTGAGCCACACTCCCTGGGCATCATTCTTTCCTCAGATTAAAAAGAAAATGCACAAAACTATATG[AGTGTGTGTGTGTGTGTGTGTGTGTGTG]GTTGATTATTATTACTTGTGGTAGTCCTGTAAAGTTTCCATGAACACTGAATTAGCAAATACTGAGCAATTGCTTCTAGGGGAAATTCAGGGTTAGGTTCCTGTAAGCCTCTGGTCACATTTTCATAAGTAGACC

>A3:102285687-102285987|varpos=102285837

AGCATGGAGTTAAGTCCGATTCCTGGTCCAGAGCCCTGTGACAGAGTTTCTGCCTGCACCCTACTCTGAGCGACAGGTTTCTGCTTCCTTTACAGCTNCAGTGACCATTTATAGGGATACTGAGGTCNTTTTAAAAGTGTTTTTTTTTT[TTTTTA]AATCTAGATTGACCCCAGATGTCTCCTTTGTTTAGTTTGCAAGTCTTGTTGAAGAGACAGAGGGATGTTGGGAACAGAAGGACTTCAGGACAGTGGTTTCCAAATCTGGTCCTCAGAACACCCATGGANCATGATGATGACATCACCAGT

>A3:102741213-102741513|varpos=102741363

TACTTACATAATACAGACTCGAAGATAAATTGGTTAAAAACCTCATGCACNGCCACGTTCATTACAGCATTATTTACAATAGCCAAAATATGGAAAGAAGCTAAGTATTCATCAATGGATGAAAGGATCAAGAACNTGTGGTNTATATA[TATATATATATATATATACATA]CACAANAGAATATCATCCAGCTATAAAAAAGAAAGAATTCTTGCCATTTGTGACAACATGGATGGGTCTGGAGGCCANTATGCTAAGATAAATAANTCAGAGAAAGACAAATACCGTATGATCTCATTTATCTGTGGAATCNAAAAA

>A3:103028198-103028498|varpos=103028348

AGCACCTGACTCTTGATTTNAGCTCGGGTCACGATCTCACAGTTCATNGGCTGACAGTGCGGAGCCTGCTNGGGATTCTCTGTCTCCCTCTCTCTCTGCCTCTCCCCTGTTCTCTCACTCTCTCTCTGTCCAAAATAAACATTTTTTNN[TAAAAAAA]AAAGTAACAGGTATTCACTGCGTTGTTCTTTCAACTTTTCTGTGGTTTAGAGTTTTTGAAATAAAAAAGTAATAAATTTAAAGAATCTTCCTAAGTTTATTTATTTTGAAAGAGACAGCATGGTGTGCAGGGGCAAGGGNAGGGGGGNGG

>A3:106938197-106938497|varpos=106938347

TGTGTGTGTNTGTGTGTGTGTGCATGTGATTCNTGTCTCTGGGTTTAAAAAGAAATAAAAAGGAAGGAAGGAAATACTAATTGCCTGGCAAAGGCTTAGAGTTAGCTGAGTAGATCGCATTAATTTAACTTCAGGGGAACACTTGGTGA[AGGTGG]NNGGGGGCNGGCAGGAGATCTCTGCAGTGTTGCTGGACTCCAGTACGCTGCTGTCTAGGAGCTCTGCCTTCCGGTTTTGTGCTTTTAAGGATTTATTGGCCTTTTAATAACTGCCCTGNAGTTNCCCTAGGNTTCACCAGGTGGCAGTGC

>A3:107014075-107014375|varpos=107014225

TATCTTTCATCTGTTTTGGAAATTTTTCTGNCATTGCTTCTTCAAATTCTGATTCTGTCCCTTGCTCTCTCTTTGCTTCTTCTGGGGCTCCAATTACACCTTTGTTGGGCTCATTTACACATCTTCCTTATCTTCTTTTCTGTTTTTTT[TTTCCCC]CCCTTTGATATTCACTTATAATCCATTCACATTTACTGTAATTATTGATGTGGTTGGATTTCCATGTGCCATTTTGCTACTTGTTTTCTATTTGTCTCTTTTTTGTTCTCTGCTCCTCCTTTACTGATATCTGTTGTGTTAAATATATAT

>A3:110818837-110819137|varpos=110818987

AGGAAAATGATTGACTGGTGGCTGTGTCCGTGTGAAAACCAGTGAAGAGCACCTTGCCTGACTGGAGCACCGCCTGGCGATCCAGTCTCCTGCTCTTTACCTGTCGTTCTGAATATAGATCCTCTGGGGTTACTTGATAACCGCACCCC[CCCACACAC]CACACACACATTATTTTATTTACTAAATTCCTTAAAGCATATCTTTGTTGGGGAAATTATAGATTATTTAATTCATTTTTTTNNNNNNGTCTTTGATTCCCAGATGTATTGTAGAAACTGAAAACTTAGAAGAAAGAGTAGCTGTGGTGAGTCGAAT

>A3:113420829-113421129|varpos=113420979

ATGATCTCATGGTTCTTGAGATCGAGCCCTATATCAGGCTCCTCATTGACAGTGTGGAGCCTGCTTGGNATTCTCTTTCTCCCTCTCTGCCCCTCCCCTGCTCACCCACTCTCTCTCTTTCTCTCTTTCTCAAAATAAATAAATAAACT[TAAAAAAAAAAAA]TGACTCTCCCTTATCAAATATTTGGTTACCCTGAAATAAAGTTTCAGAGGAAAAGAAGGATATTTAGCAGTTTTTCTTTATTTGTTAATCTGTAGAATACTGAATTTGTTCATTGAATTGTCCATTGTGGTAAACAGTGACT

>A3:113691994-113692294|varpos=113692144

AAAANNNNNNNNNNNNNNNNTTATTGTGCCAAGAGTTGCAATAGAAAATNCAGAAATTCTGAAAGGAGAGAAATGGGAAAAANNNNCGAGAGAGGAAATTACCTAATAAATATATATATTTTAAATTATNGATGCTGACATGCATGCCTCTCCA[GAAAAAAAAAAAAA]NNNNNNTGTCCATCTAAAAAGTCCAGCAAAATGAATGAAAACAATATATCCACATTAAGGCACATCACAGGAGATTTTAAAACACCAGGNGTNTAGAGAAACTCCTAAAAGCTTCCAGAGCAAAAAAGTACANTGNTGTGTTTACTTTGA

>A3:116612312-116612612|varpos=116612462

TGTAGTTATTTTTATGCATATATAATGCAATTTCCATTTTGTTAATATGAAAACTTTAAATATATTCAAGTTTATCTTATTATATGGCTTCCAGAAGTAATGATTTTCCTTTCCAGAAGTGTCTTTTTTTAAGTTTATTTGTTCNNNNN[TGAGAGAGAGAGAGAGAGAGAGAGAGA]GAGAGAGAGAGAGAGAGATAAAGAGTGCAAGTGGGAGAGGGGCAAAGAGAAGGAGAGAGAGAATCCCAAGCAGACTCCNCATGGCCAGTGCAGAGCCCAGTGTGAGACTNGATCTCATGAACCGTGAGATCATGACCTGAGCTGAAATCA

>A3:116632653-116632953|varpos=116632803

TCCTTTTGTAGGTCACATTTCTCTAATTTTTCAACTGTTTCCTGTATATGGATTTAATATTCNGTACATAGTTTCACTAGTTCTAAATATGGTGAGATTATTCCTTCTGGATCTTGTCCCTATATTCTCTTAGTAAAATACAGGGTATA[TATATAC]CATATAGGTTTTCAGTAAATGTTTTTTTNNNNNNGATTAAATGATCAANGTAACCCGAGATCACATTAACATTATTCATGCTGATATNGCTTACATACTATTGGCTAATACACTTGTGGTCAGCTAAAACCCAAGTCTTTTTCATACAGTTAAATTG

>A3:117606593-117606893|varpos=117606743

CAATATAAAGCTTTTTGNTCCATGAACAGGAACATGATCCATGNACATTTTTTAAGATCTTCTTNNNNTAATTTNTTTCAGCAATGTTTTATTGTTTTCACTGTAAAAGTCCTGTACTTCTTTTGTTAAATTTATTCCTAAGTATTGTATTTTTTT[TTTTAA]ATGTTTATTCANTTTTGAGAGAGACAGAGACAGAGTGCAAGTGGAGGAGGCNCAGAGANAGAGAAGGAGACANAGAATCCAAAGCNGGCCCCAGGTTCTGAGCTGTCAGCACAGAGCCTGANGTAGGGCTNGAGCTCATGAATGGCAAGG

>A3:119649650-119649950|varpos=119649800

TGGTAACTTTGTGTGATGATGGAAATATGCTGTATCTTGATTGGATACATGATTACATAGGTATGTATGGTCATCTGTCAAAACTCATCGGGTTTTACACTTAAGATCTATGCATTCACAGTATGTAGACTACACCTCAATANNANNNN[AAACAAAAACAAA]AAAACAGTTGCCAGGGGCCAGGGAGTGGGCNAATTNGGGAGTTGTGTTTAACGGGTATGGAGTTTCAGTTTTGCAAAACGAAAAATTATAGAGATCAGTTGGACAACAATATGAATATACTTAACACGACTGAACTTAATAAGGTAAAT

>A3:123003456-123003756|varpos=123003606

GTGGTTCAGTCGGTTAAGCAACCAACTCTTGATCTGGGCTCAGGTCACTACCTCACGGTTCCGTAGTTCGAACCCCACNTGAGGCTCCACGCTGACTGCACAGAGCCTGCTTGGGATTCCCTCTCTCCCTCTCTGCCTCCCCCTGTTCC[TTCTCTCTCTCTCTCTCTCTCTCTCTCTCTC]AAAAATAAATAAANNNNNNNNNNANNAAGGGGGAAAACCTATTCATATTCATAAAGAGAAAAAATGTATTTTGTAGGGCAGGGTCCGTGTCACTTGTGCCTTTACAGCCTGCNGAGCAAGGTTCAGCC

>A3:124239756-124240056|varpos=124239906

TCTCTCTNNNNTTAAAAAACTTANAAGAACTAGTGCATCTCCTGTGCTCACCATGCCAGGCACTGCTCTAAATCTNTACATTCATCAATTCACTCAAGTCGTCCAGGATTTCCAATGGAGAGACACTGCTTTTCTTTTCTTTAAAAAAA[AATTTTTTTTT]TTNNNNTAACATTTATTCACTTTTGAGACAGAAAGAGACAGCACAAGTCGGGGAGGGGCAGAGAGAGAAGGAGACACAGAATCTGAAACAGGCTCCAGGCTCTGAGCCATCAGCACAGAGCCCGACATGGGCCTTGAACCCACAAACTAT

>A3:124465144-124465444|varpos=124465294

ATCCTATTTTCTTACCTTACATAGATTTCAAAATTTAAATTTGCAAACTAGATACATTTGAGGATAACTGTTAACATTGGGAAGTCTTTTATTTTACAAAAGAGTTTTTCTTTAACTTTCTTTTTCTTTAATTAAAAAAAANNTTTTTT[TGAGAGAGAGAGAGAGAGA]GAGAGAGAGAGAGACAGAACATGAGTGGGNGANGGGCAGAGAGAGAGGGAGACACAGAATCTGAAGCAGGCTCCAGGCTCTGTGCTGTCAGNACAGAGCCCAACACTAGGCTCGAACTNGTGAAACANNAGATCATGACCTGAGCCAAAG

>A3:124544759-124545059|varpos=124544909

AAATCCTGTCTCTTAGGGAAGAATTTGCAGTAAATTCACCAANTGACTAAAGTCACCTAAGGTAATGGATAGGTCCTAAAACCAACCTGGAACATGTCCTCCCCNGTATCAAGGAAACTTGGAAGCCAAATTCATAATTAGAAGTTNNN[TTTTGTTTGTTTGTTTGTTTGTTTGTTTGTTT]NGTTTNTTTGTTTGTTTTTGTGAACACAGAATTAGAATAGCTGCAAGTTTCTGCACGATTTAAATATTTAAGGATATCAAATCAGGTAGACCATCTCCATAGTAACTGCANTGGGGCCTGGTGTGCCTGAATTCCTGGAGAGGACCCC

>A3:124562379-124562679|varpos=124562529

TTAAAAGTTNNNTTTAATCAATGTATTATCTCCTTTCAAAAGTCCAAATCATATATAAGNTAGATTCTGAGATACAGAGAAAAAAANNNNNTGGATGTAAGGAGGCACTTGAACTTTATTTTGTATAGAATGGCTTTGAAGTACTTAATTTTTTTT[TTAAAAAAA]AATGTAAATTCTAGTTAGTTAACATNCAGTGCAATATTGNCTTCAGGAATAGAATTCAGTAATTCATGACTTACATCCAACATTCAGTGCTCATCATAACAAGTGCCTTCTTTAATACCCATCACCCATCTAGTCCATCCCCTACCCACC

>A3:124666717-124667017|varpos=124666867

CATTTACACTTAACACTTTCATCTCCATTCCCATTTCCCACAAAGTGAACTCAAACTGCCTACCACCTACATATCCTACAGGCTCCTGTGAGGTTGAATGTGTTAATGGTTATAAGNGCTTTGACCTTCTCTCTTGGAATGTACTGCAG[GAGTAAAA]NTAAAAAAATATTATTATTATCATTTCACAGACATTTTGCAGTTCCCACTGGTTAGTTTCATTCTCTCCTTCAATCTTGAATGCATTAAAATGGCTAAATAAAAAGTATTTTGAGTAAATAAAGATACCCATGATTTTTAAAAGTTCTT

>A3:127621700-127622000|varpos=127621850

TGCCTCATCAGGTAAGCGTCCGACTCTTGNNNNNGGCTCAAGTCACGATCTTGNGGTTTGTGAGGTTGAGCTTTGCATCAGGNTTCATGCTGAGAGCGCATAACATGCTTGAGATTCTCTCTCTCCTTTCTCTTTCTTTCTCTCNCTCT[GCTCTCTCTCTCTCTCTCTCTCTCTCTCTC]AAAATAAATAAACATTTTTAAAAANNNNTGCTGCTGAAATTTTCAACACTTGTATCAGAGCTCTCTCGTAGATTGTTAACACTTCGGAACATAAATGCCCTCCCTCATGTATTTGGCTGTCCCTTACAAAGTCTAG

>A3:129070569-129070869|varpos=129070719

GACTCCTTTGAAGTCACTACACAAGTTTCCTAGACAGGGATACTNNNCTTCTTTCTGGTGTTGGGTATTTTTCCTTCCTGTGCNTAGCAGGGGCCTCCACTGTCCTTTTATTTAACTTTAATTTAATTTTATTTTAATGTTTATTTATT[TGAGAGAGAGAGAGAGAGAGAGAGAGAGAGA]ACACANGTGCANGCAGGTGCATGCGAGCAGGAGAGGGGNNNNNNNNNNNNNGGAAAGAGAGAATTCCAAGGAGGCTCCACGCTGNCAGCACACAGCCCAATGTGGGGCTCAATCTCACGAAC

>A3:129490524-129490824|varpos=129490674

AAGGAACNCCAAGGAACTCCAAGGAAGCTAAACATTTAAGCAAGTGCTTTCATCAATAGAATAAGCCTGATTTTGAAATACAGTTAATTGTACATTGAAAAGTTTTCTGAATTTGGAATGTGCTGAATTCTTCAAATAAATTAAATCTC[TAACAACAACAACAACAACAACAACAACAACAACAACAACAA]AATGTTCATTGGCTGAGCTATTCCCATTTTATAAAAGGTCNAAAGAATAATCCTTTTCTTAAATANTATGGTTTTTAAATTTACAGGCTATTTTTGGATAGCCACAGGCACTTCTAGAAAGTCTCAT

>A3:131222419-131222719|varpos=131222569

ATTTATATTAAGTTTATAAATTATCAACAATTTGTAATCTTCAACAATGAAGCTCNTCATGATGAGTGAACTATAGTTGTTTCCTGTGGTCATACCCCAGGTGNTTAAATGGGGTAAGGTTTCTAATGTTTTATTATTTATTTTTGAGA[TAGAGAGAGAGAGAGAGAG]GAGAGAGGGAGGGAGCAAGAGCNAGCACAGTAAGAAGANNNNGGGGCAGAGAGAGAAGGAGACACAGAATCTGAAACAGGCTCCAGGCTCTGANCTGTCAGCACAGAGCCTGACATGGGGCTCAAACCCANGAACCGTGAGATCATGACCTGAG

>A3:132099158-132099458|varpos=132099308

AAAAAGACCCCTTTCCCAGNAACCTGTCTGACCTCAGACATATTAATTAAAATAAANNNAAGAACAAGAAGTTTTTATTTTAAAAGCGTTTTCAGTAGTGAAGGAAGTGTATTGCCAACAGTAAGCAATTAGATCNAGTTATGGCTGTG[GTTTTTTTTTTTT]TTTNATTTTNTTTTCATTTTTGTANATATATAGAATCTTATTTTCAAAGGGAAGTTAAAGGAGTCACAGTATCTACTATTTTTAAGCACCTACTATGTGACAGGTATAGTTCTCAGCTTTTTAATNCNTTGTCACTANAGTCTTTCA

>A3:133382518-133382818|varpos=133382668

TCTCTGAATATAGGGATTTGAAAACCCGATGTGTCTCAGCATTTCTGACAGTTAAAANNTAGTATAAGCGAGTTTGAAAAGCAGTGTTCACTGGGTCCCCACGTGAATTTAAAGCAGCAGTAAAATGGAGGGGCCCAGAGCGTCTCNATCCAG[AGGTGGGGGGG]NNGGGGACCCAGGAGAGCAGTCAGGCTGCTGGGTCACCAGACCTCTGCAGCCAAATGTCCCTCTAGAATTTTGTTCTACGATCCCAGAGTTGTCTGTCNCGCTCTAAAAAGACTAGCTNTATTCTTACACCGTGGTTAGTTCTAACACT

>A3:136487763-136488063|varpos=136487913

AGACACAGAATCCGAAGCAGGCTCCAGGCTCTGTGCTATGAGCACAGAACNTGATGCAGGGCTTGAACCCANGAACTGTGAGATCATGACCTGAGCTGAAGTTGGACNCTTAACCGACTTGGCCACCANGCACCCCTAATGTTTATTTT[AAGAGAGAGAGAGAGAG]GGAGAGAGAGAGAGCATGAGTGGGGAGAGGGGCAGAGACAGAGAGAGGGAGAGAGAGAATCTCAAGCAGACTCCACGCTGTCAGCACAGAGCTGGCCGTGTGGCTTGATCTCATGAACCATGAGATCATGACCTGAGCTGAAACCC

>A3:137154911-137155211|varpos=137155061

TGGTACCAAGCATAGCACCCTAGCAGATGATGGGTGATGAAATGATGAATAATAANANNTGAGTAACACTGTTGNTGTGTCAAGCACTTGGAANAGTGGAATGAACACAGCAAACATTATCTCTGCTCTGACTGAGCTTAGATNTNNNN[TTTTAAAAAAA]AAAAAATTTTTAATGTNTATTTATTTTAAAAGNNNNNNNNNNNNNNNAACNGAGCGTGAGTGGGGGAGGGNCAGAGAGACACAGAANCNGAAGCAGGCTCCAGGCTCTGAGCTATCNGCACAGAGCCCCACGTGGGCTTGAACCCACGAA

>A3:138225108-138225408|varpos=138225258

GGTTTGAACCCNGCATCAGGCTCTGCATCGACAGCATGGAGCCTGCTTGGGANNNNNNNNNNNNNCCTCTCTCTCTGCCCCTCTCCCACTCACACTGTCTCTCTCAAAATAAATATGTAAACATTAAAAAGAAAGAAAGGAAAAAAAAN[AAAAACCC]CAGTAGTNCACATATGCATGGAATCCCNTGGTATTTGTCTTTCTCTGTCAGACTTATTTCACTTGGCATTATACTCTCTGGGTCCATCCATATTGTTTAAAGAAAAAATACCAAATGATGTCATTCATAGTCCGTGGAATTTAAGAAACC

>A3:140779458-140779758|varpos=140779608

GGGTCTAAATATGTCTTCTTTATGGCTATTCATCCCATTGCTTAGTGCTGTTCGGGGAATGCTTCATTCATCAGCATNAAGGGGTGGCAAGGTGTGTACTAAATTAATTTTGCAGATAACTGAAGTTTACCAAGTTGAGAAGCAAAACT[TACACACACACACACACACACACACACACACACA]TTTTGAATTCTNAATTGAAATATCTCTAAAATGGGCAGCACACTTTCTCNTATACAGAACANGAGAAATGTAATTTAACCATTCANNNNNTCCACCTTATAAAACAGAGATACTTTTANNGGCCCTGANACTGTG

>B1:2411169-2411469|varpos=2411319

TTTAGGTTAGTTTGGCGTAGCANCCAGAAGGGAGAAAGCCTTGAGAACAGATTGCTGAACGAGGAGTTCTAACCCCACACTCNNCTCTGTTCATATCATTGGGAGCAGCNGTTGGTTTCAGAGAGGTTTTTGAATGTTTATTTTAAGCA[GGTGTGTGTGTGTGTGTGTGTGTGTGTGTGTGTGT]NNNNNNNNAATCTTTCTTACTAAATATTTGTGAAGATGTCATCACTCTATCTCTTTGTGTGTGGATGTCATTCTTTTNAAAATAGTATNGTTACTTGGAATCAACTAGAAGGTAAAAGCTCA

>B1:2579133-2579433|varpos=2579283

TTCACTAACTATTGTGAGAAACTTGTGATTCACTGCCCTATTTGCCNTGCCATTTTCCATGTCCTCCTTCTACACTAAATTTTGTAAGTTTTGAATCAACAGAGATGTTTATTTTAGAAAAGGAATAAGGCACCCTGGCCTTCAGCTGC[AAAAACAAAACAAAACAAAACAAAACAAAACAAAACAAAACAAAACAAAA]AGATAATTTNTAGGAAGAAGGAGCAGACTGTAAAACCNTGTTCCAANGGTAGAGATGAGATAACATCTGAAATGTCCCTGAAGGGACAGGGAGACCTCAGAATTCACTGCA

>B1:2961890-2962190|varpos=2962040

CCAGCCCTGGAAGGAAGGATTTTACAGATGATAAAATAGGCTCGTGGAGCCACTCCCTTACTTTTAACTACATGGCTGATAAAAGACCGATTTCATAGTGGATCTTAACCCCTTTCCATGGCANGGACTTTGCTTTCTTTTCTTTCTTT[CTTTTTTTTTTTTT]ACTTGTGATTTCTTGCAATTCACCTAACTTGAAGGAATGCCAAATAACTTCTCGGGTTCTTCTCATCTCCAATAGCCTGTATTTATCACGAATACTATTTCGCTGNTCATTGTATCCAGGCCTTAGCAGAGACACAGCACTAC

>B1:3477676-3477976|varpos=3477826

AAGGAGAAAGACATCCCAGTTCTATTTGAAGCCATATTAATTCTACCTATTATTCATGTTTTATTATAGTAGTAATACTCATATAGCACACACTATGAACTAGACACTGTTTTAAGAGGTAAACACACACACACACACACACACANNNN[CACACAGACAGA]NNNNNNNNNNNNNNTACAGTATGTATGAAATAAATACTATTATTATCCTGTTTTTATAGAGCAGGAAACTGTGNCAAAGAGAAGTTAAGTAATTTCTTCATGGCCACACAGCCGGTAAATACTGGTGGTAGGATTTGCACGTGGNNGAT

>B1:4350075-4350375|varpos=4350225

ATTCAACATATTTTGGATAATGAGAGCTATGTTTCTCAGGGATGGNAAAAGGAGGNACAAATAAGGAAAAAGAGAAGAAAAAGATGGGTCTGTGGTGTTAGATGACTGCAAGAGGTATCAGNATGAACTCACNTTTATAGGCAGGAAGA[TGATGGATGGATGGATGGATGGATGG]NTNGATGGATANATGAAAAAAAGAAACAAATGGATGGGAAGAAATAGATTGATAGGTAATAGATAGATGATAGATAAATAGATGAAANAAAGAAAGAAAGAAAGAAAGAAAGANNNNNNNNNNNNNNNNNNNNNCGAACAT

>B1:4653261-4653561|varpos=4653411

TACAGAGAANGAGANANANGACTCCATGGGACTCACAGTTCTTTTGTTATGATTTTCTGTCATGCTACTATCTTAGCTTTTGGCTCCTTTCTATTTACCTTAAAATATATCATTGAACATTATTCCCATTATTAAAAAGGAACTTGAAA[GTTTTTTTTTTTTT]NNNNNAATTTCATGGATTATCTGCCTATGTTATGTTCTTGGAACNATTTCTCTACCTGCTTACTCATGTATGATATAAGGGATCTCTTTCAGCATTGATCCTCAAAGACAGANNNTAATACAATTTTGTCCATAACCATTTAATACACTCCCAA

>B1:4654277-4654577|varpos=4654427

AGTTCCTCAATCTATAGAATAGTGATAATAATAGCACGTACTTAACATGCTTACAATGAANATTACATAAGAACTAATGAAAAGACTCAGTGCCGAGCCTGGNAAAAATGAGAGAAATAAANGTCAGGGCCACAGACTGAACGTGCACN[TCCCCCCCCCC]AAAATTCATAGGTTGAAACCTAACCCCCGGTGTGATGGTGTTAGGAGGTNGAGCCTCTGTGAGGCGAGTAGGTCATGAGGGTTGAGCCCCCATGAATGGGACCTTATCAAAGAGACCTCAGAGAGATCCCACNTCCCTTCTGAA

>B1:6023695-6023995|varpos=6023845

AAAAGAAATCAACTTATTTCACAAATTTTACCTTACGTNTTTGTCCAAGAAAAATTACTATGAATCGAGGCACAACAACNGTACTTGTGAAAATATCGGACCTGAGATATTTATNATTTTACAATAAGAACATATTAGAATTAAGAATC[CTTTTTTTTTTTTTTTT]NNATAAATCAAGCATGTGTTTTAGATTTAGAAAAGGTTCGCGTTAAAATTAAATGTTACTTCAACAAAGAATATTTTCCATTTAGCAAAATGGAATATTTTGAAAAATATTCTTCTGAGTTTATGAGAACCTCAGCANTGGTAG

>B1:6030513-6030813|varpos=6030663

TANGAAATGATGGTTGAAATTTCTCCCAATTTAATAAAATCTCAAAATCCACTGACTCAAAAACACAAAACAAAACAAGCCTCAACGTTTATAAGCACAGAGAAAACCACACCGAGGNATTTCATAATCGAATTGTTGAAAAGAGTTAA[TAAAAAAAAAAAAA]NTCTTAAAAGCAACCAGAGAGAAAACACACATTAATTGTCAAGGNACAGAAATGAGACCTTCAGACTTCTCATCAGACACAATACAATCTAAAACACATGGAACACTGCTTTTAAAGTGCTGGGGGNAAAAACTGTCAACCA

>B1:8371440-8371740|varpos=8371590

ATCTAACTGGGAAGTTCCTTTTACTCTTGAGTATTGGGAAGGATCATATCAGGACTCNACTGGNNNNNNGNGAGAGTAGGTTACAAGCGAATGTCCAATTTTCCTAACAAGCTGTTTTTCTCTCTCCTCATTATTAGGTAGAGAGGTGG[GAAAAAAAAA]NTGTATTANATGTTGTTTCTGGGAATTAAGTGAATGCATTTAGCTGGAGTAACCCATGGATTTCATTTCTTGGGAAAGTGTGTTGGGCNATTATACCAAGAACATAAGAATNCTGGGNGGGTGAAAACAAAGGCCATCGNCTCTA

>B1:8939255-8939555|varpos=8939405

AACATATCATAAATTAGAAATTTTAAAGGATCCACTAAAAACTATTAGACCTAATAAGAAACTCAGTTAAATTGTAGGGTACAAAATCCATATACAAAAATCATTTGTGCCTGTACATTAACAATGAACTATTGAAAGAGAATTTTTTT[TTTAAA]AATCCCATTTACAATAGCATCAAAAATAATAAAACACCTAGGGTTAAATTTAACCAAGGAGTTTAAAGATCTATACAATGAAAGCTATAGTCCTTTGATTAAAGAAATTGAGGAAGACACAAATAAATAGANAAGATATCCCATGCTCATGAATC

>B1:9717800-9718100|varpos=9717950

CAGTTCGTGGTTTTGAGCCTGAGTCGGGCTCTGTGCTNACNGCTCAGAGCCTGGAGCCNGCTTCNNATTCTGTGTCTCCCTCTCTCTCTGCTCCTCCCCCACTCAGGTTCTGTTTCTCTGTCTCTCAAAACTAAATAAACATTAAAAAA[AAATTT]TTAAAAAAAATAATAAAATATTCAGCAANCTGTGCACCTTAACATTGTGTGCTTTGCTTATGACATTATGCCTCAATAAAAATAAGTAGGTAAATAAATATAAATGGCTGGTGCTCAGTGGGNCTTACCAAAACTTGTNNNNNNNTTTAA

>B1:10151117-10151417|varpos=10151267

TTTCATAGGAAGTGGTTTAGGTANGACCTCCAAGAANAAGAATGTAGGACAGCTTTAATCAAACTTGGCAATATCTACAGCTGACAAAGAACAAACCCTCTGTGGTCTTCTTAAAAGTATTNTCAGACTGTAAAATTGCCTTTTAAAAA[GTTTTTTT]NTTTTTTAATGGGTAAGTCATTTGGATATTTCTATGCACTTGCCCAAGTCATTACTTTATTACATTTTGTGTCACNCTATCTTCTTTTGTGCTAAAGAAAACCTTAAGAATTAGGTTTTATTGAAACCGAATCTCCTCGGATACAGG

>B1:10369809-10370109|varpos=10369959

ATGAGAAATCCCAGGGTTCAATGATAGGTTGATTTTCANTGTACTCATTTGGTGATGGAAACTGTTTCACCTTGCTGACTTTTCAGTGCTAAACAAGTACCGTTTGCCAGTTCATTAGAACTTGCTTCATTTCTTCTCAGTTTTGTTTT[GTTTTTTTTT]NTTGTTTCTGTTTTATCAACTTGGCGCTTCTCTTTGCTNGGGCTCTGCTTCATAGTACAATTCTCAACTNTTGATGGATGTGACACACCCACTCTGAAACACGAAAGAGTGAAAAATGCAGCANCATCACAGGGAACACAGATGCA

>B1:10903953-10904253|varpos=10904103

GTAAAACTCTTTGGCNATAAAGCCCTTCNNNNNNNAAAGATGTAATTTATGGATTGATGTAGGTAGAAAATGCCACATATATGTATGCATCTATTCTCCACTTTAGGGTCACTTCCTACTTCNGTACAACTAATGGNACNTCAACATAT[TTGTGTGTGTGTGTGTGTGTGTGTGTGTGTGT]AAATAAATTATTAAAAACAAATTTGATTCAACAAACAAANCTAATTTAATANNAAAGGACAGAATCTGTTTCTTTGAGAAATCTGCCATAAAGATNCATTTTACACTTNAAGTTAAA

>B1:11017273-11017573|varpos=11017423

GCATCTAATTTTGAATGGCATTTATTCTACTAGGATTNTAGAAGCTTTTCTTGGCACTTAATATAGCAGGAGCAAGCAGTTCATTAAGTAAATGTATTCCAGAAGGTTAAAGATGAAGGTGTGAAGATATTTTTCCTGCTTTTGTGTTT[TTTTTTTAAATTTTTA]NATTTTTGTATTCTTCATGTTTCTTTTTTATTTCTTCATATTTGGACGCTGGATTGTGAAATTAGTTCATTGTGCTGTAAGACATCTAGGATGTATGAAGAAACAGAGNNTTTCAAGACGTTACAGCTACANTGTCAACATCATTACAAG

>B1:13007744-13008044|varpos=13007894

GGTCTGGGGGAGGAGGCAGATGATTGACCAGTCCTTTAAATCTGTGTGTAACGTAACTTTTTATTCCTCATTTCTTCCTAATCTGATGATCTCGATTCTTTTTGATTAGCGTGAAAAATTCTGTCTGCTTGTTTGGAACAAAAAAAAAA[AAAT]TGTTTTTTCTTCTTCTNACTCTCCTGTGTGACTTTTAGGCATGTTATTTCCTCTCATCTGGGTGTCCGGNTACANATCTGTAATACAAAATACAAGGTTGGCTTCAGTATCTCCAAGGNTCTTTTGAAATCTATAGTTTTTGCACTATGG

>B1:15038706-15039006|varpos=15038856

TGATGAATCCCCCCATGTGAGACAAAGCCTGAATGCCAACTCTAAGTAAATTTTGGAACATGAAATACATTCATTAATTTTTAAAGTAGAGTTGCTCAAACACAAACTAAAATGTNTTTTATGTTAAAGATCNGTAGTCATCTTAAAAA[AAAATATATATATATATAT]TATATATATATTTACAAAAAACACTTTCATTATTNTTTCCATAATAAATCATCTAATTCCTACTGCATTTACTTTTTCACTGAGTTTTTTTATTCTTCTTATTTTTGAAAACTGTATCATAAGCTANGGCCTTCTGTGTTATCAATATA

>B1:16179262-16179562|varpos=16179412

GTTGATTTTCTCCTCCTCAGGCAAGTTTGTTGATTAAAATGATTATTTTAGTATTTTATGGTGGGATTGCAGATGTGGAACTGGACCCTACAAATTTAAGTCATTGCCAGTCCAGATCACTTCAGGTGCCTAGTAAGACTAGTTGNNNN[TTTTGTTTGTTTGTTTGTTTGTTTGTTTGTTT]NNNNNGTTTGTTTGTTTGTTTTTTAAGGTTTATTTATTCATTCTGAGAGAGAGACAGCATGAGTTGGGGAGGNGCAGAGAAAGAGGGAGAAAGAGAATCCCAAGCAAGCTCTGATACAGGGCTCAAACCCACAGACTGTGAGATATGA

>B1:16825545-16825845|varpos=16825695

TTTACTGTAGACTTTAATAACCTGAACAAAANTAAAAGTTGGGATAAAAAAGGAAAATAAACATAAACCCAAAGTGTAGTTCTTTTGTGTGATTATAAAATGATCTAAGTGGCAGGCACAATGTANGGCACAGTAAGCAGGATAGTGAT[TTGATGATGATGATGATGATGATGATGATGATGATGATG]GTGAGGGTGATGAAGATGATGCTATATTAAGCCAAGTCAATCTGTTTTGTATGGGGTACTTTGNNAGATGTTNACAAATATACGAGTATATAAAATAGACACTGTTCCAGCCCTGACAGAATTT

>B1:17643308-17643608|varpos=17643458

ACAGACACTTTCTGGAATTTTCAAAAAGNCTCACACTGGGTAATCATGTCCCAGACATTGAACTTGTCAGAAGAGAACAGGTCCAAAAGTATTTACCTCCAACNTTCCAGAATCACAGAGTAATCGACATTTAAATTTAATGATGGTTT[CTTTCTTTTTTTTTTT]NNNTTTTTTTAAGTTTATATATTTATTTTGAGAGAGAAANAGAGAGAGAGCTGCGGAGGGTCAGAAAGACAGGAGGAGAGAGAGAATCCCAAGCAGGATCCACACTGTCAGTGTGGAGTCCAATGTGGGACTCAAACTCACGAGCTGTGA

>B1:17794375-17794675|varpos=17794525

TAGCCAATGGTCATATTATTGCAGTTTATTTTTTGNCACTAAAAATATTCAATAAATATTCATATATGNGTAAACATTTCAAGATGGGATTATTAGGCAAAAATACGAACAAAACTATTTTCATTGTACTTGATCTTGNCAGTNNNNNN[ATATATATATAT]NTATATATATAGCATTGAATGAGAGTGTCCTTTTCCACACATCATCACCAACAGAAAGTATTTTAATTCTTGTTACATTTTCCTCACTGGGAATAAAGTAGTATTTCATTGTTACTTAGTTTGCATTTTCTGGCTTACAANTGAATTCAA

>B1:17823343-17823643|varpos=17823493

TGTATTTTATCCCTTGTTNNNNNNTTTAATCTCCCCTTTTCACCAGTTTTGATTATGGTGTGNTTAGGTGTGGTTTGCTTCAGACTTACTTCCTTCGATTTCGTTAATATCCTTGGATCTTTGAGTTTATAGTTTATGTCAAATTTGGG[GAAAAAAAAAAA]AAAAAAACTGGCATCATTTCTTGAAACATCTCTTTCTNNNGCCCTTACTTCCACTCTTTCNTGTCCTTCTGAGACTTCAATGTGGAACATCTTGATATTTTCCCACAGTTCCCTAATATTTTGTACATGATTTTTTAATCTTATTTTCTCTCTGTGC

>B1:18375688-18375988|varpos=18375838

GTAGCTGAGTCAGTTAAGCATCCAACTCTTGATTTTGGCTCAGGTCTTGATCTCANGGTCATGGGATCGAGCCCCATGTGAGGCTCCACACTGGGCATGGAGCCTGCTTAAANNTCTNNNNTTTTNTTTCTCTCTCCTTCCCTCCCTAA[AAATTAATTAATTAATTAATTAATTAATTAATTAATTAAT]NNNNATAAAACTTTNATTAGCAGNGTNAACTTAGGTTAGAAACATAAAATAGGAGGAAAATAATTTNATATAGCTGAAACTTGAATATACAATGACTTCTAAAATCTTAGANACCCATTGCAGCCACTGTAATTA

>B1:18892342-18892642|varpos=18892492

CAAAAAGTTTTGAATCTCTCCTGNNNNNGAATATGACTTTGNTATGGTNGTGCTCAATTTGTCTCACTCAGCTTTAGACATTGTCTACTCTAATACTTCAGTGATGGATAAAAGTTTAGCTTACCTTCAACCATTTTCCATGNACCTTT[CTTTTTTTTTTTTTT]NNNTTTTTTAAGTTTTGATATATAACTGACATACCATACAATTCACCCCCTAAAAGTATATGATTTAATGGCTTCAGCATATTTACAGAATTGTGCAACTATCTCTCCAGATAGTTTTAGAACATTTTCATCATCCCTAAGGTAAGGAAA

>B1:19701824-19702124|varpos=19701974

TTAGTCTTACTCATTGAATTTGAAATGTGTGGCCTCAAAGGAGCACCATAGGAAAGAGCTATCATTTTAGTTAATGAACAGTCAGTACTTACATTGCTGAAATATAAGTTACCAGTGGTACCTTTTGGTTAAAATTATNNNNNNNNNNN[TTTCCCCC]CCCCAAAATTCCTTTTTAGATGTTCTTTGAAGATCATATTGATGATGCCAAATATTGTGGTCATTTGTATGGCCTTGGTTCTGGTTCATCCTATGTACAGAATGGCACAGGGAATGCATATGAAGAGGAAGCCAACAAGCAGTCATGACA

>B1:19874755-19875055|varpos=19874905

TCAAAATGACTAAATTAACAACANAGGAAACAATAGATGTTGGTGAGGATGCAGAGAAAGGGGAACCCTCTTACAATGTTGGTGAGAATGCAAACTGGTTGCTTCCACTCTGGAAAACAGTATGNCAGTTCCTCAAAAAATNAAAAAAA[AAAACCAC]CGNTACTACCTTATGATCCAGCAATTGCACTACTAGGTTTTTGCCTGAAGGATACAAAAATACTGGTTCAAAAGGATACATGTCCCCTGATATTTACAACAGCATTATCAACAATAGCCAAATTANNNAAAGAGTTCAAATGTCCATTG

>B1:20277284-20277584|varpos=20277434

AAGAGCCATTCATGAAAGGCAAATGAGCATTCATTTGGGACCAAANAGTTGNAATTTGGGGAGCACAGATCAGGTAGAAANCCAAATAGTGTNCTGCTCNNATGGCCAAAGCTTAAGAAGAAAAGGAAGGGNCAATTACTTGAGTTGAA[GAAAAGAAAAAAAAAAA]NNAAAAGTTTTCTATGGGTGTTAACAAGCTCAGTTACCCTGGGTTTCACACTGAGTGATGACTGATNCTCTNAGCAGCACGTNNGTCCACAGCCATGGTCATGACCAGGACGTCTGTTCTCCTGCTGAACTTTCCANCAGTTGCTTGA

>B1:20613071-20613371|varpos=20613221

GTTCTGGAATTAAATGCTTAGGANNAAGAAATGCTTACTACCTACNGGCCTNTACTGGTTATACNGTCCCAACTCAGTGAAANCTCAGAAGTNGGGCTATCATCTNNTACCTAGATGAGAAAATAAANCACAAAGGTAAATATGGGTGA[GAAAAAAAAAAAAAAAAAA]NNNNNNCAGTATCTTAGATGATTAGTTTATTTAAACTTAAAAAGATTACCAAAGGCCCTCTCTGANGAAAACAATGGATCAGATATTANGATATATATAGAAATGAGCCCANTGCGGTTACAGACTAGAAAGTAAAGCATTCATCTA

>B1:21098814-21099114|varpos=21098964

GTATGTAAATTGAAATTCCAAGTCACAGNTAAAATTATGCATATTAAAAGATATAGGTTAAACATAAGGGCCAAGATGTAAATTAAACTGAAATAAAGGGAAAAANTTTTATAATACAAAATATAGCAAGAAATGGGGGCATAGAGAAN[TAAAAAAAAAAA]AAAAAAAATGAANATAAACAGAATACAAATAAATTGATAGATGTAAATCCAAACATCTCATTAATAACATTAAATTTTAGGGTCAAAATACTTGAATTAAAAGGCAAATGTTGACAGAATATNTACACAGGAAGGGACCACTATCTGCTA

>B1:21489009-21489309|varpos=21489159

ACGATTCGTGATTTCNGGCCCNGCATCGGGCTCTGTGCTGACAGNTGGGAGCCTGGAGCCTGTTTCAGATTCTGTGTCACCCTCTCTCTCTGCTCNTCCCNCACTCACACTTTNNNNNNNNNNNNNNGTCTAAAAAATAAATAAAAACT[TAAAAAAAAAA]AAAAAAGAAAAATTTTNTGATGTTTCATACATNTTTAGTTTTGTACTATACTTGAGAGAGAANTTTCTCTTTATGATGCTATTCCTTTCCCANCAATAGATTATTATTATGANCCAACACTTTTTAGCCTAGTGAGGGGAAATAGGAAGAAG

>B1:21864612-21864912|varpos=21864762

GCTCTGGTGGCCCTCTCTAAGAGGGCCATATTTCCCACCCCTCCATGTGAACCTTGTGGATGTGACATTGTGGCCAATGCATGCTGAGACTGANCAAAAGTTGNGTAGTCTTAGTTTTGCTCCTTCTCTCCAGAGAATGGCATATTCCA[GGTGTGTGTGTGTGTGTGTGTGTGTGTGTGTGTGT]CTTTCTTTAACATGACTCTCCCAGTGAAGGGACACACAGAACCAAGAAGCNGCTGATCTACAGCCAACAGGTGCTGTNACTAAGAAATACACCTTTGTTGTTTTTAGCCATTTGGATTTTATTAGCAAGACT

>B1:22276754-22277054|varpos=22276904

TCAAGCCCNNCGTCAGGCTCTGTGCTGACAGCTCAGAGCCTGGAGCCTGTTTCAGATTCTGTGTCTCCTTCTCTCTCTGACCGTCCCCCATTCATGCTCTGTCTCTCTCTGTCCCNAAAATAAATAAACTTAAAAAAAATGAAAAAAAA[AACCC]CAACCTAATACTCTTATATTGAACATGTTTTTAAGAAGTAGGAAGTTTATAAATACATGAACTTAAGATGTTTGTGTGATANCTATGTGTTTTCTCATAGCCTCACTAGTAAAATACAAAAATTTCTGTTCNATTCCAGACTNCCTTTTT

>B1:23736442-23736742|varpos=23736592

ACCTTAGGCAAGTTGCTTAAATTCTTAAATGGGGAAATAAATAATCATAAGATTATTGGNGGATTAAACTGTTTAGGAGACGAAAGCAGTCAGAGAGTGTATACCATAGATAGNNNNNNNTATATGTTAGCAATCATTGCTCGTACAAC[ATGTTGTTGTTGTTGTTGTTGTTGTTGTT]GTTGTTGTTGTTGTTGTTGTTGTTGTTGTTTTGATATTTCACTACANTGATGGATTAACTAAGGGAGAAGTGACAGATATAAATATTGAGTCTTCCAAGTAAATATCACATGGTTTTTCATGTCTGTGAGACGTCTTTTGTGTCTACTG

>B1:23981371-23981671|varpos=23981521

AGNACATCTGAAAATCCTTTACTCCATAAAAGNAGTGAGAGAACTGATAACAGTGGTCAGAATCAANTTTTTCAGACTTTGGAAATTANACAAAAGCTTTCAGAAATNTGAGTAACTTTTATTCAAGAAAAATATCAGAATCTTGGTAN[AAAAAAAAAACAAAA]NNNNNNGTGAGCTTTGTAGCATNTNAACATACCCTATTTCCNTCTCCTCTTTCTCAGCTTTGCAACAGCCNTGAGAACCAGCAGTCTTCAACCACAANGAAAACCAGCTATCTAGTGGTTTTGAGAGAGGAAAGAACAGGGTTGTGCTTG

>B1:25892365-25892665|varpos=25892515

CAGTTAAGCATCCACTTTTGGCTCAGGTCATGATCTCGCAGTTCAGGGGTTTGAGCCCCACGTCAGGCTCCACNCTGATGGTGCAGAGCCTGCTTGGGATTCTCTCTCTCCCTCTCTCTATGACCCATCTCAAAATAAATAAATACACT[TAAAAAAAAAAAAA]AAAAAAAAGGCTTTGAAAACAGTTCATAACTCATTTGGCAGTGANACTTGATGTAAACTGACATGAAACTACCCAATAGTCTTTATCCCATTTAGTGTGANTATCCAGGCATTTTTGCTGCAGNAATCTTAATATAGTTAATTATAGGAT

>B1:26360551-26360851|varpos=26360701

TGGTCTGTGAGTTTGAGCCCCACATTGGGCTNTGNGCTGACAGCTCAGAGCCTGGAACCTGCTTCGAAGTCTGTGTCTCCCTCTCTCTCTGCACTTCTGCTTGTTTTCTGTCTCTCTCTAATTATCAAAAATAANTAAACATTAAAAAA[AATTTTTTTT]TTTTTNAACCAACATACACATAGTGGAAATCCCAAGGTGCAAGGTGAGAGACAGAAAGAGGCATAAAGAATATTTAAAGTAATGACCAAAAATTTTGCCAAATTTGATGGAAAACATTAATCTATAAATTCAAGAACNTTACCAATCCNC

>B1:27350330-27350630|varpos=27350480

AATAGAAAACTGGGGCACCCAGGTGGCTTAGGCAGTTAAGCATCCGACTTTTGCTTTAGGCTCAGGTCATGATCTNGGAGTGTGGTGAGTTTGAGCCCCACACTAGGCCNTGTGTTGACAGCAAGGAGCCTGCTTGGGATTCTCTCTCTC[CCTCTCTCTCTCTCTCTCTCTCTCTCTCT]NNGTNCCTCTNCCACTTGTGCCATCTCTGTCTCTCTGAAAATAAATAAATCTAGGGGCACCTAGAAATTCTAGGCTAATCTCATTTTATTTATGAAGAAAACTGTACCTAAAGAAGTGCAGAGTAAAGCAATTGTGGAAG

>B1:27431504-27431804|varpos=27431654

AGTGACATTCATCCTATGAAAACAGTACATTGACTTTATNTTTAGAACTTCCCCAAACTGTNATGTGTGACATTCATACAAAATTTTCTCTACTCTCACTCTGAAAATAGAAGATATTTTCAGAAAAATCTTGTTTATTTTATTGATTT[TGAGAGAGAGAGAGAGAGAGAGAGAGAGAGAGAGA]NNNNNNGAGAGATTGTGCTTGAGTTGGGGAGGGGCAGAGAGAGAAGGAGAGACNATCCCANGCACACTCCATGCTGTCAGTGCAGAGCCCAATGCAGGGCTCAGTCCCATGACCATGAAATCATGACCTGAGCCAAAATCAAGAGTTG

>B1:27636938-27637238|varpos=27637088

GAATCTTTCCACCCATTCTAATTGCCCTCAGTTACAGTGAGGTTACAGCCGGAGGTACAGAGGAGGTAGGACTTTCCCAAGCTTGTTAGGGACCCAGCCAGACCTGGAAGGNCACTTCCTTAGGGTAGACTAATCCTCTTTTCAAAAAN[AAATTTTTTTTTTAATTTTTTTTTAA]TTTTTTTTTAACATTTTATTTATTTTTGAGACAGTGAGAGACAGAACATGAACGGGGGAGGGTCAGAGAGAGGGAGANNCAGAATCTGAAACAGGCTCCAGGCTCTGAGCTGTCAGCACAGAGCCCGATGNNGGGCTTGAACTCACGG

>B1:29542840-29543140|varpos=29542990

CCCCAAATTCCTATGTTGAAGTCTTAATCTCCANTGAGATGGAATTAGGGAGNAGGATTTGGGGGGAGTAATTAGATTTAGATGAGTCATGAAGGTGGGGTCCCAATGATGGGANTAATGTCCTTTTAAAAAGAGAGTGACCAGCTCTT[GCTCTCTCTCTCTCTCTCTCTCTCTCTCTCT]NNNATCCNGTATGAGGACACAGCAAGTATGAGGCTGTCTACAAAACCAAGNAGAGGGTTCTTACCAGACACCAGAACTCCCAGCACCTTGACCTTGGACTTCTCAGCCTCCAGACCTGTGAGAAA

>B1:31000948-31001248|varpos=31001098

CCCCTTTATTGAACTGTGAACACTTTCCAATATATTATAGGGAAGATCATAATTCATGTCATCCATTAATCAGGTACCAGAGCCCCATCAAAACTCTCTAGACGAGTCTCANGTTACTTTGTCCTTTGCTTTACATTTGNAAAAAAAAA[AAAAATT]TCCCCCAATATTATTTTCCTGTTTTGACTAATCCTAATGATTCTAATTATNTATTTTATTTTTTTTCTTGGCTAAGTCTTGGCAATGGAATTTGCTTTTGCAGAACTTCACAGCTACAAGACCTCTTCAGTTCCAAAGCACTCTATAGAT

>B1:32714327-32714627|varpos=32714477

CTGAGAGGGAGAGGACCAAAAGAATAGGGGGAGCACANGNNNNNNNAGGGTGGTNAAATTATTCTGTATGATATCGAATGGTGAAGACATAACACTGCTTTTGTCAAAACCAATAGAANNGAACACAGAACAGTGANGCACGCAAACTT[TAAAAAAAAAAAAAAA]NNGATCGTTTAGGAGTTTGGGAGAGANTCGAATTGAATGCACAGTATGACAAAGACACTCNNTATTACAAATGTCTGAAACAGCCTCATCAGAGGGGGTTNGGGAAAAGATGCTGATATTAACAGACTTTGGAAGTGAATGAA

>B1:33231488-33231788|varpos=33231638

CTGGGTGGCTTAGTCGGTTAAGCATCGACTCTTGGTTTCGGCACAGGCCATGATCTCATGGTTTGTGGGTTCGAGCCCCACATCAGGCTCTTTGCTGGAAGAACGGAGCCTGCNTGAGATTCTCCCTCTTTCTCTGCCCCTCCCCTGCT[TTCTCTCTCTCTCTCTCTCTCTCTCTCTC]TCTCTCTCTCTCTCTCTCAAAATAAATAAATAAACTTAAAAAAAGAATATCAAGTTTATTATTGTTATCAAAAGTCCTTGTAATGTTTGTATCGTTTGGCATATTAGAGAATCTAACACATTAGGTGGTGATTCCAATTTAAAAAGTGAA

>B1:33484620-33484920|varpos=33484770

TGCAAGGGCAGAGGGAGAGGGGAGGGGCTGCCTCCCCANCACGATGATGGCAGTGNNCCAGTCAGCAACGGGAACACTGGCTGCAAGGCCATGTGCTATTCACACCTGGGCCATGACACCTCTGCTGGGGTTTGAAAATGCCTTTTTCC[CTGTGTGTGTGTGTGTGTGTGTGTGTGTGTGTG]NNNNNNNNNNNNNNTGTGTGNNNNNNGTACATGCTCACTTGTACCAGCTTGCACACACATTTTCCTCTAGTTAAGCCTAAATGGGAAAAACTGTCCCTGTAATTTTGAAGAGGGGGAGGGAGCAAATAAAACCTTCAGTAGAAA

>B1:33822431-33822731|varpos=33822581

TATAGAAATAAATATACCAGTGGAATAAAACAGTCCAGAAATTAATCCAAGTACATACAATATTGGTATATAATAAAANNAGCATCCTAAGTCACAGGGGGTAATGATGGACTTCTCAAAAAATGCCTCTGGGACAACNGGTGGCATTT[CAAAAAAAAAAAAA]TTAGATCTATATTTCTCACATAATACATAATGAATGACAAATGGATAAGGGAGATAAATGTATCAAAAAACAAATATATCAAAATGAAGTCATACAAGCTCTGGAAGAAAAAGTGCTGAGTTCTTTATCCTTTAGTGTAGGGG

>B1:33851497-33851797|varpos=33851647

ATCGAGCCCTATGTCAGGCTCTANGCTGACACTGTGGAGCCTGCNTGGGATTCCCTCTCTCCCTCTCCCTCTCTGTCTCTGCCCCTCTCCTGCTTATGCACTTGCTCTCTCTTTCTCTCTCTCAAAATAAATAAACATTAAAAAAAAAA[AACAAAAAACCACC]CTGATTACATACATCAGTAGAAGATTCTGGTTAACAATGCTGGTTAATATAATAAGTTAACTAAAGCCTANAGCTCAATATCCACAAGTGGTTTCTGCTTTTTCCTTACATTCACCTAAAGTTGCCATCTATAATCTAAGATCAAGTATT

>B1:33969289-33969589|varpos=33969439

CTTTTAGTATATTCATAGATATGTACAATATTANATCAGAAACATTACCANCCTAACCACCGTTTCATCCTCTCTCCCCACCTCAGGCCTAAGCAACCACTGGNTATACCTTTTGTCTCTGGTATCTCTCTCTNCTGAACATTTAAAAAAN[ATTTTTTTTTT]TTTTTTTANGTTTATTTATTTATTTGGAGAGAGACAGCACAAGTGGGGGAGGGGCAGACAGAGANNGAGACANAGAATCCCAAGCAGGCTCCATACTGCCAGCACAGAAGCCGATGTGGGGCTTGATCTCANGAACCATGACATGATGAC

>B1:35350973-35351273|varpos=35351123

CAACTGTGCTAATCACTGATTTAACTGTAAGAATTTTAAATGTAGAAGACAGTGTAAGAAGCTTTATTCTGAGATCTGCATAAAAACATGCTAGTGTAGTAGCTAGTAGGCTCCTGTGTATTTTCATTTGGCATATAGAATATTTGAAT[AGTGTGTGTGTGTGTGTGTGTGTGTGTGTGTGTGTGTGTGTGT]TGCAGCTTTGATCTGAATAAANCATTTAAAAATTATAAGAAATAAGAATAAAATTAATGTGGAACTGTTGAATTAATGGTCTCCACTGCTTTAAAATTCTGGACAACA

>B1:35387972-35388272|varpos=35388122

GAACNCCTGTGCACTGTTGGTAGGAATGTAAATTGGTGCAGTCACTATGGAAAACAGCATAAAGAAANNTCTTCAAAAAATTAACAATAGTATTACCATATCACCCAGGAATTCCACTTCTGGGTATTTACCAGAAGAAAAATTAAAAAANN[AAACACACACACACACA]ACACACAAAAAACACACCACTAATTCAAAAAANNNNNGATATATAAACACCCTATGTTTTATTGCAGTATTATATACAGTGGCCAGGATATGGAAGCAGCCCTAGTGTCCACTGGTACATGAATGGATAAAGAAGATGTGGCCTACATACACAATA

>B1:36169848-36170148|varpos=36169998

AGAGCCAGAGCACAAATATAACAGCATTTTCTAGCCCCTACTTCATAGCCAGATACCTCCCGCATCTTTAAATCTTACCTAATTTTGGTGAAGNACAGGGCAGTAAGTAAAGTCAGTGCTAACATGTACAGTGCCTTTAAATGAATTAC[CAAAAAAAAAAAAAAA]NTGCCCACTGTGGGCAGCTGAACTAAANAAAGGCATCCACTTCTGAGCACANGAAGCATATGAAGATCCATGTNGCACATANCTTTCCTATCTACTCNCATAAGCCTGCTAAAACTTGCCATAGGTACTATGACCCTAGCACCT

>B1:40236835-40237135|varpos=40236985

ACATTAAAAAAAANNNNTTAACAAACAGATTCTCCCTCCCACCTCTCTACCTTCACCCGTCCTCAAATCCTTCTCCAGAGACTAACCCAGTTAATNGTTATTTTCTCCCAGTTTTTCAGAGACTGTCTAAGCCTTAATTAATATATATC[CTTTTTTTTTTT]ACATAAATGGGACCCTATTACATAATTTTTCTATCACATCACTCTCTTTCATAATAGGTTGTAATAATCATGTGTGCTAATACACTTGTTTCTTTTCAAGAGCTCCATAACATTACACAGTATGTATGGGCCATAATCTAATT

>B1:40368277-40368577|varpos=40368427

AACATCACAATATCAACTCATTCTTCATTTGGCTCCAGAGCATCCTGNAAAAAGAACACAGTTTGCCCCTAAAACTAGAGAATGGTGATAAGTAAACATGCTGAGAACTCACACTTATTAGTTCACATACATAGATATTTGGGCATATG[TACACACACACACACACACACACACACAC]NNNNNNNNNNTATAAGAGGATCAAGATTCTCTTCAGGTACTATGTGACACTCTCATTTTGGATATTCCACCGCTCTTTTNAAAATTTTTTTCTTATGTTTTTNTTTTTTTTATGTTTATTTATTTTNNNNNNNNNNNNNNNNNNNCAGA

>B1:40870169-40870469|varpos=40870319

CCACAGGCAATTAAAAAGCCAGTCACAGAGAATCCTCTAAGCAAGCTCTGGGCTGTTGAGAAACTGCTTATATCACTCCTAATTCACTGATCATTTCAGAAGGAACATCAGAAAGACAGGATGGAATGTAGGTCAACANAGTATTTTNN[TTCTCTCTCTCTCTCTCTCTCTCTCTCTCTCTC]AAAATTAAANNNNNNTTAAAAATTAAAAAACAAAAGCAAAAACCCTAAGAGCAACAACTAAGAAGTCTGACAATAGGGGCACCTGGGTGGTTCAGTTGGTTAAGCATCTGACTGTGGCTCAGGNCATGATCT

>B1:41495144-41495444|varpos=41495294

GTAATCAGAAGATACATCACAAGGACTTAAGGGGTGCCTGGGTAGCTCAGATGGCTAAATGTCNGACCTGGGCTCAGGTCATGATCTTGTGAATTGTGAGTTCGAGCCCCCATTGGGCTCTGTGCTGACAGTGCAGAGTCTACTTGGGA[TTCTCTCTCTCTCTCTCTCTCTCTCTCT]CTCTCTCTCTCTCTCTCTCTCTCTCTGCCCCTCATCCACTTGCATTCTCTCTCTCTCTCGCTCTGAAAATAAATAAACTTAAAAAAAAAGGACTTAAGTAACAATGCGCTCATTTCTTTTGTATCTGTGAGGTGCTTCC

>B1:41784379-41784679|varpos=41784529

TCATCTTGGAGTGTTTTCCATGCCAGCATATATATTNACTTTGATATAATTTTAAACTTAAAGAAAACCTGCAAGAATAATAGAAAGAATTCNTGACACAGATTCACCAATTATTTATACTTCACTCCTTTTACTGAATCTTTTTCTCT[GTATCTATCTATCTATCTATCTATCTATCTATCTA]CATATTATTTTTCCTGAACCATTTTCAAGTATGTTGGAGACATTGTACCCCTACCCCATAATNTCCTCAGTGTGTATTTGCTAAGAACAGGANTTTTCTTTTACATAACCACAGTTCAATTATTACAATTAG

>B1:44147494-44147794|varpos=44147644

GAAAAAATTCCGTGTACTTTTNAAAAATTATACTAGGGCATACATATCACTTCAGTAATAAGCATGCCAAAACGTATAGCATGAATCTATGAAAAATATCCACCAAAGCCAAATTGAAGGACATCTTTAAAACAATTACTCTGTAGCAT[TAAAAAAAAAAAAA]CTNCAGAAAAGAAAAAAANNNNNNTTAAAACAAAGAAAATATCTAGATTAAAGATNAAAAATACATTATGACTAAATACAAACAAATATATGACTCTGGATTTGTTCCTGAAAAAGACAAAATAGACATAAANACTTCCCTAAGACAA

>B1:44697229-44697529|varpos=44697379

GCACCAAAATAAAAAGCTTCTGCATAGCTAAGGAAACCATCAGTGAAACAAAAGGTAAGCCTACTGAATGGGAAGATGTTTGCAAATGCTATATCCANTAAGGGTCTAATATCCAAAATATATAAAGAACTTCTACAACTCAACACCAA[AATCATCATCATCATCATCATCATCATCATCATCATC]TAGTTAAAAATGNGCAAAGTATCTAAGTAGACATTTTTCCAAAGAAGACATGCAGGGGCCAACAGACANATGAAAAGATAATGAACATCACTCATCATCAGGGAAATGCAAATC

>B1:45286994-45287294|varpos=45287144

TGAGCCCCATGTCAGGCTCTTTGCTGACAGCTCAGAGCCTGGAGCCTNCTTTGGATTCTGTGTCTCCCTCTCTCTCTNNNNNNNNNNNNGCCCCTCCACCACTCTCANNNNNNNNCTCTCTTTCAAAAATAAACATTAAAAAATACTCAAAAACANNNN[CAAACAAAAAAAAA]NNAAAAACAACGCAATTAAAAAAAGAGCCTAAGACCTTAAGAGACACCTTACCAAAGAAGATATACAGATGACAAATAATCATTTGAAAAGATGCTCCACATTATATGTCATCAGGGAAATGCAAATTAAAAAGCAATGAGNTTAAACA

>B1:46692008-46692308|varpos=46692158

TTTCCAGGTCACTAATTTTACCTTCTTCTTTACTCAAACATCTGTTAAACCCATCTTTTACATACAATTTTACTTCNTATACTTCTTAAATAATTTTTGACGGATCATCATTNTCTGGTAACTTTCTCCATCTTGTCACCCAATTTTTC[TTGTGTGTGTGTGTGTGTGTGT]TTGNNNNNNTAAACATTTTTATAGAAATTCAAATATTCTTTGTTTTCCAATAATATTCATTGACATAAAAATATCATAATATAATAATAGTAAATGGTAGAAGTTAATATAAAAGATTATAGATACAGTTTAATACCAATTTT

>B1:48398759-48399059|varpos=48398909

GTCCCACCTGCTTCTTTTAGTTTTGTTGTTAGCACCTGCTGTTTAATTCTGGCTCAAATATCCATTACACATTTCCTGGTCTCAATCTCACATAGTGACCTTTAACCCTAAGCCTTTATAAAAAGGTCCAATAGCAGGATGGGTTTCAT[TAAAAAAAAAAAAAAAA]TTGAAGAAGATGTTTAAAGATTATTTTTGNTTACAAATCTTACTTGTATGTAAATTATGATCTGCNTCTATGGTANCCCCTTTCCATATATTTTTTTTCTGCTTTAANTATAGGAATCATTGATATTTTGATATCAGNTACA

>B1:48558597-48558897|varpos=48558747

GGGTTCAAGCCCTATGTCAGGCTCTGTGCTGACAGCTCAGAGCCTGGAGCCTGCTTTGGATTCTGTGTGTNCCCCTCTCTCTGCCCCTCCCCAGCTCACATGTTCATGCTCTCATTCTCTCTCTCAAAAATAGATAAACATAAATTTTT[TTTTAAAAA]AAAGTATATGCTATGTAGGAAAGTAATACAAATTGATTTTTATTTTCAAAATATTGTTCCNCCCTCAAAGTGGTGAATAGAGGGAGAGAATCTGGGCTAATTATTAGAAAACNATTCAATAATTTAGGCCGNAACAGGTAGTGATGGAAA

>B1:48635417-48635717|varpos=48635567

AGAAAGGTCAGGNACTGTCATGGCGNTCATGTCTCTGGTTCTAGCCTATGTGGACCTGGGTACAGAGNTCAGCACTTCACTATCCTTCACAGCTCCACAAAGCCCTATAAAACAAACCAACANTTTAACTAAGAACNTCTCTGGGAGGA[GAAAAAAAAAAAAA]AAAAAAAACAGCAAACAACTTAAACCCTCTTTAAANATTTCTATTCTTTTCCAAAGACNCCTTTAGCCACCAGTCCTCAGAGTTCACTCAAGCATTATGTCCCTCTCTTCTCCCCACGCTCTTCTTCCCCTCTCTCTTCTGACCACCAGT

>B1:48867989-48868289|varpos=48868139

CTAGTTTTCTTTAAGATGGAATCTTTTTAAAAAANNNNNGTCTCTCCTCCCTGGAGCTTCATACTAGTATCTGCNTTAATATCTCTAAGCAAAAGCTCAATGAGTGTGACTATCTGTGAAAGGAAACATCGTAACTGCTCCTGAGGGAGTGTGTG[CGTGCGTGTGTGTGTGTGTGTGTGTG]TGTGTGNNNNNNNNNANGCACACATGCAATAATGGGAGATGCCTGAAAAAAGAGGGGTCATTATTGACTCCCCAAATTCTACCACATATGATNCATGAGGCAGAAATATTTCTAGGAATTTCTACTGCAGGCAGCTCCATGAGGAGA

>B1:49288733-49289033|varpos=49288883

ATAAACTTCACCCCTCATTTTCTGTCTCTTTGTTGGCTTTTGGATTAGCATTGGTGCAGAAGTTTTGGCCCTCTGCAAAGTAGTTGCCATACAAAGAAAAGTTTNGAGACTNTATTTTCGCTTTGTAAGGTTATAGATGTGTTTCCTTT[GTGTTTTT]NTTTTTTTAACTAGNATTTNGATATGAATCATTTCAAAGGACTCCTTTAAAAGGAGGTAGGCATCTAACACAATGCAAATTCTTAGAAAGTAATCATTTCAATTCTAGGGTACATCAATCACTTTTGAGCTAGGAAACTCTTCATTCCTG

>B1:50744536-50744836|varpos=50744686

CACACACACACACACNNNNNNNTATCTTTTTACTGGTAGCTTCTCCCTAATTCCAGCTCCCCNCCTCCTCANCATAGCCCCCAAGAGGCAGTNGTCATCATGAATGTTGTGTTTATTAGTCTCTGCCTTTTTTTTAAATGTTTATTTTT[AAGAGAGAGAGAGAGAGAGAGAGAGAGAGAG]CACAAGTGGGGTGGGTGGCAGAGAGAGAGGGAGACACAGAATCCAAAGNAGTCACCAGCCTCTGAGCTGTTAGCACAGAGCCCCNATGTGGAGCTTAAACCCACAAACCATGAGATCATCACCTGAGCTGAAGTCAGA

>B1:50854660-50854960|varpos=50854810

TGTGTGCATGTACTCNTGCACGATGGCTGCTCTCTCCTACCACCCAAGGACACTGCAGCAAGCCAGGACATCACCTAATGGCACTTACAGGATGCAAGNNNNAAGGCAACGTGATTCAGTACACAACTAATGGGATTTCTTGGGANNNN[TTTGTTGTTGTTGTTGTTGTTGTTGTTGTTGTTGTTGTT]TTAAGGAGAGTGAAATTTGGAATAAGGCTCCCATTTGCTAAATCCTGGGTGCTTGCTTCCTCTCTAATGAATAGTTTATGGAGCATGGTGAGGGACATCAACCCTCTGCCAAGAGTTTCAGGTCCTC

>B1:51416968-51417268|varpos=51417118

CTGAGCCTNGGTTCATTCTTTTTTGTGAAATTGTAATATTCATACCTATCTAATAGGCTTGTAATGATGTTCAAACAAAATAGGTATAAAAATATTAGAATGCTATTGCTATTTTTTTATTAAATTTTTTTAAAATATTTTTTTATTCT[TGAGAGAGAGAGAGAGAGAGAGAGAGA]NNNNNNNNNNCAGAGCATGAGTGGAAGAGGGGGCAGAGAGAGAGGGAGACACAGAATCTGAAGCAGGCTCCTGGCTCTGAGCTGTCAGCACAGAGCCTGATGCGAGGCTTGAACTCACAAACCGTGAGGTCATGACCAGAGTTG

>B1:51429942-51430242|varpos=51430092

ATCGTGACACAGATAATAGACACATACGATAAATATATAACATGCTGATTAATATTGGTCATATATATTAATCATATATATTAAAGATTAATATTTGCTTAGAAGAAGAAGCAAAAGTGAAACTATTTTGCTTTTTTGTTAAAAAAANN[AACAAACCTGA]AGTTTAGGAAAGGGAAGTTTGGCNTAAAAAAGAAGCCTGATTCAGTTAACATCATAGATATTTAATCACTGCTTCATGAATGAATGTGTGGAAACTTTTTTTTNNNNNNCTGCAAATTTAGTACACACAAAATTGACTTGGTAATGGAAAAAAGTAAT

>B1:51906587-51906887|varpos=51906737

CTCTCTCTCTCTCTGCCCCTCCCCTGCTNGTGCTGTCTCTCTCTCAAAAATAAATAAAGCATTTAAAAATAAAATAAACAAACACATTTGGTTATAAACAATAAAGAAACAAAATAAGTGCTTGGCTATTACCNGAGATCACAGTTCTT[TTTCCTTCCTTCCTTCCTTCCTTCCTTCCTTCCTTCCTTCCTT]NTCTTTCNTTNNTTTNTTTGTTTTGTTCTCTCTNGCTCTCTTTCTCTCTATCAAGAAATGATTTCTTGTGTTAAGGAATCTTAAAGACAAAGAGAAATGTGGGTCTACA

>B1:52814115-52814415|varpos=52814265

AAGACTGGATGGACATATCTAAAAATTTACCAGTAATGTCTCAAAGTTNTGGATAATAGATGATCTTTTCTTCTCTAAATTTTCTACAAAGACTGTCTCTTTTGAGAATTTCAGTATCAATAGAAGATAGATGATAGATGGGCAGACAA[AGATGGATGGATGGATGGATGGATGGATGGATGGATGGATG]TAAAGATNATAAATGGGTATGTGGGNTGGGTGGATAAGTGGATGGATGGATGGTTGGATGGATATATTAAAAAGATGANAGGTGGATAANTAGGTAGGTAGATAGGTAGGTAGGTAGGTANATAGATCTC

>B1:54953001-54953301|varpos=54953151

NAGTAACTTTACATGGTGGCCCAATACTCCATTGCATGGATGTGCTGCTATAGTAATATACTTACTATTAGGACTGACACAATAATGAACTTTAATATGATGAACATTATATTCATGAATATGTATGTGTGTGTGGTATGTANGTGTAT[GTATATGTATATATATATAT]NNGTNNGTGTGTGTGTGTGTGTATAGCTTTACATATACTTTGCACTTTTCCTTAGTATAAATTCTTAGAACTGGATTCACTGTACTAATAGCTGTACGTATTGATATGTATTGCCAAATGTATGTCAAGAAAAATTCACNGCAGTTACT

>B1:55534441-55534741|varpos=55534591

CACCTAGCCTGGAATATTCTCCCCACCCTCCATTAATTTCAGGGACAATATATAATACTATTCAATATATATTTTTTATGTTCGATAAAATTTGAGGTGAATGCATGGTTTTGGGATGGGGGTGTAAGTGGGGAAGGATACATGTTTTN[ATATTTTT]TTTTTTTTCCCCAAAGGTATTACAGGGCAATTCCAACTACAAAATTTTGTACTTCTTTTTTTAAAGCTCCATGTAATAGGAATGAAAAAGCCTATATACTGAACAGAANGAAGATATCGAAAAATATTGTGTGTACAACTTTACCGCAG

>B1:55913163-55913463|varpos=55913313

GGCGCCCAGGTCAATGGCCCCTCTGGCCTTGTCTAAGGTGATCATCTCTACAAGCATTACACAGATGAATGGAAAGAGATCAGCAGCCTGTTTAACCTTGAAAACAGTCCAAGTCAGGACCTGGGATAGGTTACATTTTTCATTTTTTT[TTTAAA]AATGTTTATTTATTTTTGAGAGAGAGNNNNNNNGCAGAGAGAGAGTGAGCGGTGGAGGGGCAAAGAGGGAGAGGGAGACACAGAATCAAANNNCGGCTCCAGGCTCCNAGCTGTCAGCACAGAGCCCANTGCAGGGTCTGAACTCACAAANNGCGAGAT

>B1:55947768-55948068|varpos=55947918

AAATTAAAAATTGGCCACACACNCCTGCCTGCTTAGCTGTAACAGATGTGATTAATGTCACAATTTAGTACCCACGCAATAATGAGGTGCCCAAAGAGGACTCGGTAAAGGTAATTAATGCTAAATTCNCAGCAGTTTGATTTTTAACN[TAAAAAAAAAAAA]TTGTTCTAAATAGTAAATATTTTCCTATTTAATAATTAAAATNCCTTACATAGGCATGTTTTTTCCACTTCAATAGTATAAATCCAAGATTCTCCAGGGCTAACTGAATGACAANCTAGTAACATCAATGAGATACACCATAC

>B1:56875619-56875919|varpos=56875769

AAGGTCCAACCAATTTCTTCTTATTTTTCCATGACCTAATTTCTCTCCCGTATGCACCCTAATATGTTTTACTTATTTAGCACTTATTTAATCATANACATTTTGGGGGTCATCTAATTCATTGCATTATTATTTAACATTTCACACAN[GTTTTTTTTTTTTTTT]AATATATATGACCAAGTCTGCAGGCTCTTAAGAGCAGAAATTGGTTTACAATTGATTGTATTCCCCAACATTTAAGGCTAGACCTGTCAAGAGTAGCTGCTGAATAGATGTTAATTGAATAAATAAATGAATGTATAAAGAA

>B1:57813865-57814165|varpos=57814015

CCTCAAGTTCTACTTTGCAAGCCACTTCTCATGATTACTTATGCAGGTGGAAATAGAGGGAGGCAAGTTAATTAATAACAAAAATGTATCTCATAGTAGCACATAATATTTACNGAATGTATTACAGTGAATTCTATAGCTCAGAATTC[CTTTTTTTTTTTT]GTGATGGATTTACACATCCTGTGGGAGTTGGAACACAANGAACAAGTGTGACCATGTAGCTCTNTCTACTATTCTTCTATTGTGTCTGAACCAANCAGTCTCATGTATTGTCAACATTCTTGAANNGTGTGAGGCTAACTTACTAG

>B1:58228125-58228425|varpos=58228275

CTGGTTTTTCTTCCCCAAGCTTTACTGAGGTAACATTGACAAATAAAAATTATATATTTATGATATACAATGTGATGTTCTGATTTATGTATACATTGTAAAGTCATTACCACAATCAGGCTAATTAATATAGCCATCAGTTCACATAG[GTTTTTTGTTTTGTTTTGTTTT]NTTTTGTTTTGTTTTGTTTTGTTTTGTTTTGGTGATAGCATTTAAGATCTGCTCTTTTAGCAAATTTCAAGTATACAATATANNNNNNNNNACTGCAGTAAACATTCTATACATTAGATCTCCAGAACTTACTCATCCTACATAACTGA

>B1:58412314-58412614|varpos=58412464

CACCCAAGCTTCTTTTCCTCTCATACATCTTCTAGGCAAGTACACAGAAGGTGCTGTATCAATTAGAAAGCATCTCAATACTTTTAGAGATAGTAACATAGTCATATGAAATTGTACAGTGAATACCATAACTCATAGATTTCTATNAT[TTAGATAGATAGATAGATAGATAGATAGATAGATAGATAGATAGAT]GATAGATGTTTACCATAAGATAAATATAGTCCGCTATAAGCACTTCATAGAAAATTACTCTCCTGCTAACAGAAACCATTTAGTTTGTTTCCAAAGAAGCCTACC

>B1:58485192-58485492|varpos=58485342

TTTGTTATTTTTCATTCCTTCTTGAATCTACAAGTTCTCTTCTGGAAATATTTTTCTTCTGAATATAATATTATGATATTTTCTTTAATGTAAATTTGCTGGTAACAATTTCTCTCCATTTTTGCTTTTCTGACCATGTCTTTTTTTTT[TTTTTTC]CATCTTAATGNTTGTTGGTTGTTTTAGCTAGCTATACATTCTATGTTAAAACTTAGTTTTTTGAGTACATTAGCAATAATATTTCATTGTCTTCAGACATAACATTGTTTTAGTTGAGAAAAACATTGTCAGTCTTATTGACCCTTTGAA

>B1:59375239-59375539|varpos=59375389

GTTTGGTTCGTTTTTTTNNNNNNCATGCGTCTGGTACATCTTCTAACTGTACCAAACTTAAAATACTCTCAGGGAAAATANTTTTTTCTTGGATAGTGAAGAATTCAACACACAAACTCACACACACATGCNNNNNNNCNCGCGNACACACACACA[CTCATACACACTCA]CACNNTCACACACAAATACTCTTCTCAGATTAGTAATAGAAGCAGAAAGTGAAAGACAAGTAAATTNAGAGTAGACAAACACTTATATATATTAATGTGTATCTCCAGGCCTGTTTTAGGTCACTATTATTATCTCTCTATATTGAGCT

>B1:60198339-60198639|varpos=60198489

GCCTCAGAAAGGTGCCTGAAGGCAAGGATCAGAGTTCCAAAGTTATTGGAGAGAGGTTGNCTTATCAGATCTGCAGATAAGAGGAAAGATGAGAGGGNAAACAACCCAACTGACTAGGATGGGCAGATTGAACAGAGGAGAGGGANNNN[CAGAGAGAGAGAGAGAGAGA]NGAGAGAGAGAGAGAGAGAGATGGAAATACAGTTAAATTTGNATTTGATATAAACAATGAATAATTGTTTTAGTACAAGTATGTCCCATGTAATATANTTTATCTGGCAACTCTACAGCTGACGCAGTTGTCCTTGGAAGGACCTACTG

>B1:60702130-60702430|varpos=60702280

AATTTGGATTACNTCCTTAGAGACTGGGATNTTCTTTTGGAGAAGGCTGGGTGATGCATGTCTGCTAAAAAGTGTCCAGCCTCCCACTGTCTTCTAGTGCTAAGATTCTGTGAGTTAGAAACAAAAAGATTCNACTAATCATTTTTTTT[TTAAAAAAA]AAAAAGCAAAACAAAGTTTATTTTTTTATTTTGAGAGAGAGAAAAAGTGCACAGGCATGAGTTAGGTAGGGGCTGAGAGAAAGNNNNNNNNNNNATCCCAAGCAGGCTCCATGCTGTCAATGCAGAGNTCTACACAGGGCTAGATCTCAC

>B1:61669904-61670204|varpos=61670054

CTGCCCCTCCCCTGCTTGTGCTCTGTCTCTCCCTNTCTCTCANNNNNNNNNNNNNNNNNGTGAAATGGAAATTAGGACAGGAGGCCTGCCAATTTTGAGAAACTACATAAAGCAATTAATGAGGAACATCGTGGGGCACNTTGGGAGAA[CAAAAAAAAAAAAAAAAA]CAAGAAGGAGAAAATGGGACATGAGATAGAAGGAGTAAGCAATTAGAATCAGTAGNCACTAGTTACATTAAGNTAAACTGTTACCACATTAAAAATTCATATCAAAAAATAAATATTAGGTTTATTTTTAAACACACATTNAT

>B1:61975737-61976037|varpos=61975887

ATAGACCTAAAACTTATTTACAACTCAATTTATGGATTTAGGTCAGTTATGAGACCTAGTTATCTTCATGCAATTCCTACACTACTTTGTGGAATTTGCTGAAGTACTTTCTCTCCCTAAGGGACCTGACTTAAATAGCATTATTACAA[CTGTTTTTT]TGTTTTTTTTCCAACTTTTATCTCTTCAAAAACCCTCTTTATCTTAGACCCACCGNGTGTGAAGCTCTTATTTACTAAATGTTTCCTCTGTGTTAAGGTAAATGAAAGTAATGAATTTNGAGAAAGTATNTTCTCTTCAAGAAATAGGG

>B1:62126924-62127224|varpos=62127074

AACATGGAACTAAGTTCCTAGCTGTAGGAGACAAACAATAAGCCATAAGCATAGTGTTGAATCAATTATATATTATGTTAGAAGAAAATAAACACTGTGGCAAAAGTCGAGCTGAGTAAGGATTTGGACATCTATTTAAATCATTTTTTT[TTTTTTCTCT]CTGCTAATCCACTTTTACTGAATTGGCNACCTCAACATCAAACTCTGCTTTTACGGTCCCATTTTGGTGTTCAGCCCAGACTGGCAGGAATTTTTCAATTCCATTTCCTAATTCCTATGGGAGGGAATCTGATTGTTACAATCAAGTGA

>B1:62328816-62329116|varpos=62328966

TCTTATATGTTTCATATGATTCAGTTTCATATGATTGGAAGAATGGGGCTTGCTNNTGTTAACTTCATANNNNNNNNACTTTACATCATTTATCACCTAGTCCTGTGCCTTCCCACTACAGTATAAAATTATTACCTACTATTTTTTTT[TTAGAGAGAGAGAGAGAGA]AGAGAGAGAGAGAGAAAGANAAAGCACCAGTAGGGGAAGGGGCAGAGGGAGAGAGACATAGTAGGAGGGAGAGAGAGAATCTNNGACTTCGCCCTCAGCACAGAACCTAATGCAGGGCTTGATTCCCNNACCNTGGAATCATGACCTGAG

>B1:62452653-62452953|varpos=62452803

TGCACTCTTCAGGCTTTGATCTGTAAAATAACAGTAAACGTGATAAATCATTAGCCATCATTGTAGTCCTTGAAGGCAGCACTAATGGGGGACTTGGGAAATGTAGAGTGCTCTTTGTCTGCTGNGTATTTTATTGCCCTNAAATGGTC[CAGAAGAAGAAGAAGAAGAAGAAGAAGAAGAAGAAGAAGAA]NNNNNNAAGCTGTCAGAAGGGCAAGAGAGACTGTGGCAAAGTATCTGTCAAAGGAAATTAGCATGAGATTGTAGATTAAATNGATTTTATTATTCTAAAAAGTTAAGTNNNNNNAA

>B1:62717980-62718280|varpos=62718130

TCACAGATAATAGTACCAGCAGGAAAGATGTTTTCATTTTTGCTGATATGTATGGGAATCTGAGATAAAGAAAGTGATGACTCTGTTGTTGTAGTNNTTTGGTTTATTTCTTTCCTTTCTCCTTGATTAAAATGAAAGTTCTTTTTTNA[AAATTTTTTTTT]AACATTTATTCATCTTTTGAGAGACAGAGCNTGAGTTGGAGAGGGGCAGAGAGAGAGGGAGACANAGAATCCGAAGCAGNCTCCAGGCTCTGAGCTGTCAGCACAGAGCCCAAAACGAAACTCGAACTCNTGAACCGCTAGAT

>B1:63078191-63078491|varpos=63078341

ATGTAAAATATATCACTTCATTGATCACCAGGATTTTGATTTAACAAATTAGCCTGGTAGCATTTTCTTCTTTCTTTAGAGTCAATGCTGACCTAGTGGTATTCAAACTTGGCTGCAGATTAGAATTACCTGAANGGTTTATTTTTTTT[TTTTTAA]ATTTTTTAANGTTTATTTATCTTTGAGAGAAAGAGAACATGAGCAGGGGGAGGGCTAGCGAGAGGGNAACTGAGGACCCAAAGCAGGCTCTGCACTGNCAGCACAGAGACCAGNGTGGGGCTCNAACTCATGANCTGTGAGATCATGACC

>B1:63496108-63496408|varpos=63496258

TTTGNCTCAGGTCATGATTTCACAATTTGTGGGTTCGAGCCCCGCATNNGNCTCTGTGCTGACAGCTTGGAGCCTGGAGTCTGCTTTGGATTCTNTGTCTCCCTTTCCTCCTTGTGCTCTGTCTCTCAAAAATAAATAAACATTAAAAA[AAAAAGAGAGAGAGAGAG]NNAGAGAGAGTGAATAGGAAAATGTGTTAAACTGTGGATAAAATGAGAGAAAATGAATGTTAGGTAGCAACTTGAAGACTTCCATTGAGAAAGCTTTAGTATTTAGATGNTTATAACANGTTTTTATTACNCAAACTTTTCTCTTTTGTG

>B1:63664155-63664455|varpos=63664305

GAACNAGCAAGTCAGAAATCTGTATGGCAGGCTGGAAATTCANGCAAGAGTTGATGTTGCAGTCTTCAGTCTGAATTCCNTAAGGCAAAGGGCTGGAAACTCAGGCAAGATTTCTATGTTGTAGTTTTGTTTTTTGGGTTGTTTTTTGT[TGTTGTTGTTGTTG]TTGTTNTTTTGTTTTCTTGTTTGTTAATTTAAATCCAAGTTAGTTAACATATAGTGTAATAATGATTTCAGGAGTAGAATTCAGTGATCTATCACTTACATACAATACCCAGTGCTCATCCCAAGTGCCTTCTCTAATGCTCATCATCCA

>B1:65786965-65787265|varpos=65787115

GGAGAAAACAGAAATATACACGTTGAAAGCTTTGTTATAAACNGAAATGAATAAAAAAAGAAAAAGGTCTACCTTTCTCCACATCTATTGCCAAATCTATGGTTGTAGCTTTCTCCAAGATTATCTGTACTAATTTGATGGNTTTTTTT[TTTCC]CATATCAGACCATAATTTAGATTTTTAGAAATGTTCACAGAGGGAATTGAGCTTTGTTTCTTCCAACAGAATTAGAAAAATTATCGGCAGTTGAGTTTTTAAAGATTTTAGTATGGAGATTACANGCCTGAAGAGAGAGATCTGTTAAAC

>B1:66132041-66132341|varpos=66132191

TTTCTATGTGCTCCAAAGAAGACCTGAATATCTTGCCCACTAAATGATGTGAGAAATTAAGAGACTGAGTGTGAGGAGACTAGTTTATTGCNNACNGACATTTAATCACTGAAGGATTTANTCAGAANGTCTAAGGCATAAATCAGTAA[AAAACAAACAAACAAACAAACAAACAAACAAA]NCAAACAAACAAACAAACAAAAACTCTCNCCATGTGTCTATATTATTTTCAGATGGCTTAGAGAGAAGTATCTTGTCTGATCTCCCTGGAATGGAGAATGAGGGAGTGAAGTCAGCATTTGGGGACTTTGTACTATGTGGAGCTTGGG

>B1:66553122-66553422|varpos=66553272

ATTATTTATTTTCATAAATTATTTTTCTGGGTGAATATTAAGAAAGAAAATGTGNNCATTAATTCTGGTTTTATGATTCTGTATATTTCTTTATGTCAGCATCTATAGTCAGGTCTTGATCTGACCCATCACTTTTTTATTACAGGAAAAAAA[AAAACCC]CCTACTTGATACTTTATTTTAAATGTCATTTGGTGTCTCCTTTAAGGCAAAAAAATCTCTTTGCCNAATTCACCTAATATAGAAAAAAGCCAGTTTGCCCCCAGAACAGTAGATGTTCAGAAGTCTCACAGGCAATTTCCTGGAATAGTC

>B1:66635030-66635330|varpos=66635180

TTTTCCAATTGTATTGAGATATAATTGGCATACATCACTGCATCACTCTACAAGTTTAAGGCATGCAGCATGATGCCTTGATTTNNNTNTGTAGTGAAATGATTACTACCGAGCTAACATCCACATTCTCATAGGTACAATTAAAAAAA[AAAAAAAGA]NNNNCAAAATTCCTTANGGTGAGAACCCTTAGGATTCACTGTCTTAACTTTACTATATATCACAGAATGTTGGCTATATTTATTATGTTATATGTTATATCCCTGGTACTTATTTACAACTCGAAGTTTGTACTTTTAGACTACCTTCC

>B1:66728360-66728660|varpos=66728510

CCATGGNATCCATCTAACNCTTGCAATACTTTATTTTCTCCATTTCCCTTCAAGTAACNNCTGAAAAGGGCTGAGATNCTCCAGTAGCCTTTTCTGNCTGTTGCCAACATATTGTNCACAGCCACTTGAAANTATCATCACTTTTTTTT[TTTTTTA]AAGGATTCACCATTCTATTATTTTTTAGTTTTCTTTTTTAACATTTATTCATTTTTTTGAGAGAGTGTGAGTGGGGGAGGGGCAGAGAGAGAGGGAGACACAGAATCTGAAGCAGGCTCCAGGCTCCGAGCTGTNAGCACAGAGCCTGAT

>B1:66900888-66901188|varpos=66901038

GGAATCAAGGTAATTCTGGCTTCATAGAAAGAGTTTTGAAGTTTTCCTTCCATTTCTATTTTTTGGAACAGCTTCAAGAGAATAGGTGTTAACTCTTCCCTAAATGTTTGGTAGAATTCCCCTGGAAAGCCATCTGGCTCTGGACTCTT[GTTTTTTTTTT]NGGAGATTNTTTTGTTACTAATTTGATTTCTTTACTGGTTATGGGTCTGTTCAAATTTTTTATTTCTTCCTGTTNCAGTTTTGGAAGTGTATATGTTTCTAAGAATCTGTCCATTTCTTCCAGATTGCCTATTTTATNGGTATAT

>B1:67266542-67266842|varpos=67266692

GAATAAATNCCNAAGAACTAGCTTTGCNCCCTTGACCATGTCAATAATATCAAACTATTCNGAGTGTAAAAATGCTATAAAAATTGGCATATTCCCAAGAAAAAGCAAAAGATAAGTGATTAAATTTTAGGAAAGACTTTTAAAAGTGA[ATAACTTTTTT]TTTTTTTAAACTGTAAAGCACTTAAAACTCATTCANCTATGTGGTTAAGTTGTATGNTTTTTGTGAAAACTTCCCTAAAGGTGAATTATAGTATATTGATAGTATAGTTAGAAAATTGGAGGTCTAAACAAAATGNGAAACAAACAATTT

>B1:67659457-67659757|varpos=67659607

AGATAGATAGATAGATAGATNCTAGATGGATGTTAGATAGATGATATATAGATAAAAAATGGAACAAAAATCTGTATGTTGATACATCCCTTACATGTTCAATGATGAAATGTTTGTTTTAGGTACCCCCTAGGAACATTANGGTTATT[CTTTTTTTTTTTTT]ATTATGGTTTAAANNAAAAGGAAAATCAAACATATGCTTGTCATTGTGAGATGAACTGCAAAGTCATCTTATTTNCTCTATGACTCTAATATTAGCATATANTGTATTGTTAGGCATTATCTATTTCTGACAGGACCTGAAGC

>B1:67949031-67949331|varpos=67949181

AAATTAATTCTGTACTCTATAACTCAATAGGTGATTANAAAATATACAGGGCATTAGATGACATCAAAATTATACTTCAGAAAACANNNNNNNNNAGATATATCAAAAGTAAGNNNNNNNATAGGGAGGATGAACAAGAATGGAATAAG[CAAAAAAAAAAAA]NNNNCCTATTAAGAAGAAATGAAATTATGCTTCATTTAAAGAGCTTAGTAGTTATCACATGCAGAAGAAAAATCTGGATAAACTAAAAATNAGTGACTGTTTNTGGACTCAGAGAATTGAAATTTCAGGGGAAATTGCTATCTGGA

>B1:68109789-68110089|varpos=68109939

AAAAACTGAATCTGCTATAAGTAGTGTAAATTTAAGAGGAATCATTTCAGAGTTTTTGATAATTTACAAAANCATTTGGNAATTGCTAAAAGAAGAAGAAGAAGGAGAAGAAGAAGAAGAAGAAGAAGAAGAAGAAGAAGAAGAAGAAG[AAGCAGCAGCAGCAGCAGCAGCAGCAGCAGCAGCA]NNAATNCCCAGGCACATTCCTCAAATAACACCTAATATCTCAAATGCAAGAAGGGGTTCTTCTCATGATGCTATCNTGAAGNTTAAGAGCAGAGGGATTAAAAGTAGGTAAACAAAAT

>B1:69118808-69119108|varpos=69118958

GTGGNTCTAAAACACTATTTCTCCTACGAGTCTTTTATTTTATTTATTTTTGAATATACACACTCAAANCATTTTAATGACTAAACTGNAAGATCAATTTATGTTTATAACCAGTTAAAAATTGACCTATTAAATCAAAAGACCACANN[GTTTTGGTTTTT]TTTTTTATGTATATGCAGACTACCAACCCTCCTATCACAATGCCACTATTTCATAATTACCATTCAAATGTTCTTGGCAGGGAAACAGATGTGAAAAAAATTTACTTCAGTAAAGAACTATACAAAATAAACAAANNAAAAAAGCATT

>B1:70567213-70567513|varpos=70567363

AGCTCTCCAGAGTGAAAATAATTATGGTTTTCTATTTCTTTAAAATATTGACTGAAAAAGATTTCCAGTTTCAGAAACATAAANACTAACNTCATTTGGTTCTAAACAAAAATATACCTTAATTATCAAGCTAAAACCATAGCNAAAAA[AAAAAAAATAAT]TGACATTATCACCTGAGGTTTTCCTCAGCCTATTTGTTNACCATGTAATCAGACTCTCTTAAATTAATTTCAGCAGAAAGTCTGCCANTTCATGGGCTTGCCCCANAGCACATCAGGGGATCGTTAATACATTGGGCAGTTAGAAGAA

>B1:71315628-71315928|varpos=71315778

ACTGATTCAAGAGCTTTGTAAGAGCGATAGTCATTGATGCGCCATTTGGCCTAAGCAAANGGTCAGATTTGCAGAGCCAGATGAGCATTAATGTATCGCTGGAAGTAACCAGCAGGTGCAACAGTTTGCAACNAATCCNTTAGTTAACA[ATTTTTTTTTTTTTT]CCTTGGGATACNNNNNNAGNGGGNAAGAGAGGGAGAGAGAAGTGGAGGGAGAGAGAAAGAAGAAGTNAGGTGTCCCGGTTCATCCAATTGCTGTGTCCAGCATGGCGTTGGGAACTTGGTGGCACCGTGTGCCCACACTGT

>B1:71382332-71382632|varpos=71382482

TCCAAATTCATTCTATGGGACCAGAACTACCCTGATACCAAAACCAGATAATGACACCACTAAAAAGAGAACTACAGGCCAATATTCCTGCTGAACATACAAACAAAAATTCTCAATAAAATATTAGCAACCTGAATTAAACAATGCAT[TAAAAAAAAAAAAAAAA]NNCATTCACCACAACTAAGTGGGATTTATTCCTGAATTGCAAGAGTGGCTCAATATTCATAAATCAATCAATATAATACATCACATAAATAAGAGAAAGGGNTAAGAACNATATGATCATTTCAATAGATGAGGAAAACACGCT

>B1:71508319-71508619|varpos=71508469

GCATTTAATTTGAAAATATAATTGGAATAAATCTGCTATTCTAAAGCAAGGTAATATTCTAACAAGAAAGCATTGAGTAAAAGTTATCAAAAATATTTNTAACCATTTATCACAGGCTTGGTATTGTATTTTTAATATTTATTTATTTT[TGAGAGAGAGAGAGAGAGAGAGAGAGAG]TGCAAGAAGGGGAGAGGCAGAGGGAGGGAGACACAGAATTGAAGCAGGCTCCAGGCTCTGAGCTGTCAGCACAGANNNNAATGCNAGGTTTGAAATCACANACTGTGAGATCTTGAC

>B1:71531716-71532016|varpos=71531866

TTATTATGTTGAGGTACATTCTCTCTNTATCNACTTTCTTANNAGATTTTTATTATAAGTGGATGNTAAATNTATCAAANGCTTTTTCTGCATCTATTGAGATAATNATAGGAGTTTTATCATTCATNTTGTTAATGTGGTATATNACA[CTGAT]ATTGATTTACAGATGTTGAACCATTCTTGTATCCCTGGAATAAATTCCACTTGNTCATTGTGTATGATNTTTTTAATGTATTTTTGAAACAGGTCTGCTAANATTTNGTTGAGGATTTCTGCAGGTATATTGATCAGGGATATTGGCCTG

>B1:71563975-71564275|varpos=71564125

ATGCTCCTTTTCTAGTTGTCCCAAGCAGTGCCATCTACACTGATAAAATTAGAACTTCAAATTAGGATGATTACATTTATTTGACAATTTTGGTGATGTTACTATTAATGTCAGGAAATACCANTTGTAGTATTAATTATGATANTTTA[CAAAAAA]AACANNNGTAAGGTAGTAGGATGCAAATAAACACATCGAAATGAGAAATAAGAATTTTNAGCTATAAAATGGGATCGTAAAACAAGCTGTAGGGGCATATAACNTGGAAAGATCTTAAGGAATATTAATATGACTGTAATTTCTCCTTCA

>B1:71831488-71831788|varpos=71831638

GACATATTAGGAAAAGGTATTNTAACATTTCTATTGGAAGCAGTCACTTTCCTTGATGGGATTAAAGCTTCCCATGGTCAGAGGGTNGACCTTTCACAATACAGGACTNGGTGCATTTGTTGGCCTTTCAACACTCCTATTAGTAAGTG[GTTTTTTTGTTTTTTTTTTT]TCCCCTCATTTCATAATACACTGGTATTTAGAAGCTGGTTTCCATGGATATTGCCTGTAGACAGTTACGATATTTGGTGNTAACAGTTCTGTATAGACTNTANNNNNNNTAAAATTTTCTGGAAAATGTTGTAGTGGATAATG

>B1:73530052-73530352|varpos=73530202

ATAGGGAATTTCTCTTTCATTCCTTGATGCATTGTATAAAATTATTTCCAGATATNTCATCATTCCTGTTGCCTGTCTCATGTTATTTGCTGCAAAGTTAAGTATGATTGCAAAAAAAATGTATGCACAAAGAGAATTGATACTTNNNN[AAAATTTTTTTTT]TTTTTTTAATGTTTATTGATTTTTGAAGGAGAGACAGAGCTTGAGCAGGGAGGGGCAGAGAGAGAGGGAGACACAGAAATGGAAGCAGGGTCCAGGCTCTGAGCTCTCAGCACACAGCCCGATGNGGGGCTCAAACTCACANACCATGAG

>B1:74646868-74647168|varpos=74647018

ATCATGGAAAAANTCTCTCAAGTGTGCACAGCNAAAAGTATGCAAAGATGTTCACGGTAGCTTTCTCTGTGACAACAAAAATTTGGAGAGAAGACAGTCTAATTGTCTATCAGAAGGAGAATGAATTCCATTTATCTGATGGAATACTG[TAAAAAAAAAAAAA]TTACAATTACTGAGATAGAGCTGATGTGCCAAGATGGATAAATCTCCAAAACAGGGTACCACCCTTAATTCCAATCGAAACATGGTACCACCACCAAAAAAGGTACCACCTTTAATCATGAAGTGTGGNCTGCCTTCCATTT

>B1:74671249-74671549|varpos=74671399

GACAAAATAATGATTGGCNAAATCACAGCATCTTNGATTTATCTGGTTAAGTTTCAGTCATTTCCTGGTGGGCTGGAAAAGCCATGGTTACTCAAATTCCCTTTCTGAAANNNNNNNGGAGGGAGAGAGAGATCCCCAAAGTAGTTANN[GACACACACACACACACACACACACACACACACACACACACACAC]CTCTTCCTCTGTTACTAATAATATACTGAACTCCATCAAAATCAGGAAGTGTCACTGGCACNTCATCTATANGGAACACTTAATCAGTGTCGTTAACAGTCTTAGGACACATTA

>B1:75755751-75756051|varpos=75755901

TCTCGCGGTTTTTGAGTTCNGGCTCGCGTTGGGCTCTGTGCTGACAGCTCGGAGCCTGCAGCCTGNTTNGAATTCTGTGTCTCCCTCTCTCTCTGCCCCTCCCCTGCTCATACTCTGCNTCTCTGTGTCTCGCAAAAGTAAACAAATGT[TAAAAAAAAAAAAAAA]NNNNNNAAAAAGAAAATAAAGATTATTGATTTTTGTTGTTGTTGTTAAGGAGCAAGAAGAAAACNTAGTGATTCAACTTCTAATCTTAAAGANGAGAGGGGAAAAAGAAGTTTGTAGATAAGTTTGAACAAATTTCTCANCCC

>B1:75805658-75805958|varpos=75805808

GTGAGTTCNAGCCCCNTGTAGGGCTCTGTGCTGACAGATCAGAGCCTGGAGCCTGTTTCAGATTCTGTGTCTCCCTCTCTCTCTGCCCCTCCCCNACTCATGNNNNNNNNCTGTCAGAAATAAATAAATAAACATTAAAAANNTTTTTT[TTAAAAA]AAATGTTTAAACTTCCAGGTATTGGATTGGAATTAGACATTTTCAGTGTGAACTCATGAATTGCACTGTATCTATATTTATATGGCATTGATATTAATATATAAAAACAGATGTAAATGTGTNTATGNATGTGTGTGTATNTATACATGT

>B1:75822034-75822334|varpos=75822184

GTCTTTAACATGATGAAGTGGGGCTAAAAATAGCTGGCAGAGAAGCCCGCTTCCCCTGCTTGCAGTTGATGTGTGTCTTTGTGTCCCATACAATGGAGGGGGATATTCCTGTGTTGGCACAGCAGTGCTGAAAATGCCTTTTTTTTTTT[TTTTAAA]AAGCCTTTTTATTAAAAAATAAACAAACAAAAAACGACTTGGCATTTAAAAAAATTCAGCTGTCTTTGAAGCATGAGACACNGTAAGANNNNNNNNGTGGTGAAACCTGCAGCTAATGGAACTAGCATTTAGTGTTGACTGACAAAGATA

>B1:77990956-77991256|varpos=77991106

TCTGTGAGTTCNAGCCCCATGTCAGGCTCTGTGCTGACAGCTNNNNNNNNNNNNNNNNNNNNNTTGGATTCTGTGTCTCCATCTCTCTCTGCCCCTCCCTACTCATGCTCTGTCTCTCTCTGTCTCAAAAATAAATNAAAAAATNNNNNNNN[AATTTTTT]TTTTTAATAAATAGAAANTTTTAAAAAGTGAGAAGGAAAGTCAGCAATGTAGAAAGAGAATCTGGAGGTAAGGTATCAAAGAAGCTTACAGAAGAGAGAGAAACATAAACGCCATAACCCNGAGAGGTGANNNNNNATAAGCATCCTTTG

>B1:78638788-78639088|varpos=78638938

AAATATTCAGGTCAACATCCTTANAACTGGAAGGGAGTGNCATGTGCATATTTTTTTGAGTGATGCATGTATACGAAAACATCCTGTAAAGTCGTCCCCTAAATTCCNCAGTCATTCTAGNTTCCAGATTTATTTTTTTNNNNNNNNNN[TTTTTAAA]AATTTTAGAAGGGNGTAGAGAGGAACGGAGAGAGAGACAAAGAGAAACAGAGAGAGAGAGAGAATCTTAAGCAGGCTCCATGNTCAGGATGGAGCTGACTTGGGGCTTGATNCCAGGGTCATGGGATTATGACTTGAGCTGAAATCAAGA

>B1:78877902-78878202|varpos=78878052

GTAAACATGCAAATAAGTTATATTTTTATTTAAAATATTTCAAAAGCATAGAGAAATACAGAAACTAATATAATGAGCANTCGCCTATTTACTATCCAGATAGAACTTTAAAATTTTTGTTTATATACTTCTCTTTANNNNNNNNNNNN[CTCTCTTTTTTTTTTT]TTTTTTTTTCCTTAACAAGGTCTTGANACAAAGCATGCGGAAGAAGAGTTCCTCGAGAAACTGTGTATATATGGTTCTGAGTAGCCCTGGCAGATGGATAGCTAGAGCCTCAGGGTGGTCCTGGCATTCCCCNTCCCCAACCAGGGGAG

>B1:79723821-79724121|varpos=79723971

CCAAGGAGACACTTTCACAAACGGACATTTCTCTTACACATGTAANNNNNNNNNNNNNNNNNNNAACTTCTAATTGGTTTTCAGAGATTCTCTTATGTCCACAGTTTCTTAAAAANAACCAGCTTAAAATAATCAATATGTCAAAAAAA[AAAATAAT]TAATATGTCAAAGAGGCATATTTNGGGNTGGTAAAATTTGCCCCCNNNNATAAAGAGGTTCCTGAAGACCTCATGTAGCAAAACCACCTATCTACCNTGGACAAGGTTCATTTTTCTTGACCTTATGTGATATAGAATTAAACTTCTTTCCTATC

>B1:79957302-79957602|varpos=79957452

TTCTCCCCAGAATTANTGAGGTAAAACTGGCAAATAAAATTATAATACATTTAAAAGTGTGTGATGATTTGACACACATACACCTTGTGAAAGAATTCCCGCCATTGAGTTAATGAATAAATGCNTTACCTCATGTATTTGTCTTAAAA[AAATTTTTTTTTT]TTTTTTTTCCTTTTGTGAGAACATTTAAGTTCTACTCTCTGAGTAAATTTCACTTATACAATACAGTATTATCAGTTATGGTCANCATATTATATATTAGATTTTCAGACCTTTTTCATCTTCGTACCCCTTTATCAGTTACTTCCTATT

>B1:80378984-80379284|varpos=80379134

TCAGGCTCCTCTGTGAACCTCCACAACATATCTCTTAATTACAGCCACTGGTTTTTAAACTAGGGTAGCAAAGGAAATCAGGTTGCGTGATAGCATCCTACAGTTTCTTACGTGAAATGATCAGGAAATGGGGCATTCAGCCAAGAGGN[GAAAAAAAAAAAA]AAAAAAACACAGATTCTCAATTTTCAAACAATTAGAAGACAATAAGTGTGTGGCATTAAAGCTATACTGAATATAATCTTTGATGATTCAAGAGGATTTTGTGGATCCTCAGGGTAATCANTCTAAGTATTAATCATCGCTGTAAAAAGA

>B1:81907088-81907388|varpos=81907238

CTATGTTTCTCCACCATGCAAAAAGTGGTCTGGCTCCATTGTCAGTACCATGTTTTTCCACTTCTGCCTTTTACTGGGGGAGGAGNGTAAATTTNATTNTGGTAGAAATATTCAGAAACCTATTGTTATTCTATACNNNTTTTTTTNTT[ATTTTTTTTTTTT]AGCATTTATTTATTTTTGAGAGACAGAGATAGAGCACAAGTCNGGGAGGGGCAGAGAGAAGGAGACACAGAATCCNAAGCAGGCTCCAGGCTCCGAGCTNTCACCAGAGAACCCNACNTGGGGCTCGAACTCACAAACTGTGA

>B1:82166824-82167124|varpos=82166974

GAAAATGGTGACCTCCCAGTTCTCGAACAAGGATTGTATCTGTGCAAAGTTCTGCAGTAACAGAAATAGTTAATTCTGTAAACTTAACGGGACACACAGATCTCTGCAAGCTCCCAAAAGCTCCAAATCATACAGGATGCNTTTTTTTN[TAAAAAAAAAA]AAAGCAGGCTGAAAGTGTATCTAAAAACAATGTAACTTTGGGGGCNCATGGGTGGNTCAGTCCATTAAGCATCCGNCTTTGGGCTCAGGTCATGATCTCACAGTTCATGAGTGTGAGCCCCACATTGGGCTCTGTGCTGACAGCTCAGGG

>B1:82730321-82730621|varpos=82730471

GATAACATATATNNNNGCAAATTATATTTGAAGTTTTATTTTTTAAAAAGATACTTTTATATAGGTACCTTACAACTTAGTTANNNTTCTATTACTATCTTTTATTTATTGCTCCATCTCCAATGCCTGGAGCACATATGTGCCTTAGCACATATTAGGCTC[TTGAATGAATGAATGAATGAATGAATGAATGAATGAAT]NNNNNAAATGAATACTTAAATCTCAAATCATGAAATACATTCCCTCAGAAGTAATTGCTAAATTTTATTTTCAGGAAAGATTTTATAATTTATCGGGAACCAAATGTTTCTCCTTCACAAGTAACTGAAAATAA

>B1:83436716-83437016|varpos=83436866

NNNNNNNNNNNNANGAATGAATGAACAAATTGCCATCAACTCAAGAGCATGGTTTTTAGAGATTATCTCACCCTCCCTTTTGCNTCATGTGCAGACAACAAAGATTTCCCAAGACAAAGAGCAATAAAGAATCTCAGNCATTATGCTGC[ATCATTCATTCATTCATTCATTCATTCATT]NATTCATTCATTCATTCATTCATTNGAGGGCTTTTGTTGAGCCTTACNTATAGTAGCTGTTAAAAAGGACACNNAAGGGGGGCACCTGGNTGGCTCAGTAGGTTAAGCATCCAANTCTTGTTCTCAGTTCAGGTCTTGATCTCAGGATA

>B1:83498920-83499220|varpos=83499070

TGAGACAGTGTGGTCCAATAAAGTTAAATTGACTTTGGGTTGAACTTGTTTGTCTGATCCCTTGCCAGAGGTTATAGTCACACACTTGAACTGATTTTCACTCTGTTTCTTGGTATCTCTCTNNNNCTCTCTCTNCCTCTCTCATTTTT[TTTTTCTGTCT]CTCACTTTTATGTCCTTTTGTACTCATTATCCACAACCTCCCTTCCCCTTCCTAATGTACAACTGCAGAATTACTTCTTAANGCTTCTGTAGACCAATTATTTAAAAAATGAGTTCATNCATATCTATCTTCTATCATCTGTCCTATCT

>B1:84858384-84858684|varpos=84858534

AAAAGTGCCAGCTTGGTGTCTTAGATTTTGTGTAACTGCATGTTATAGTTGGCTGACCATATCCTAAAGAGGAATTGATATCTATGCATATATTAAATTAATTTTGAGGGTTTCANTTAGGCAAGGCCTAGATATAAATATGAAGGNAT[TTAATAATAATAATAATAATAATAATAATAATAATAAT]TAAATCAACACATATTCATTAAAGGCCTGTTACATGGCAAACACTCATTGTGGGGTAATTGGTGATTTTACAGNACAAAACAGGCATGGTCCATTGCTGCCCTCAAGGAGCTTGTGGAGGTGACA

>B1:85009888-85010188|varpos=85010038

AGAATAAGAAATACCTAATAGAAATACCCAAATACCTAATAGATAAATTAAGTAATTAAGGANTCTTCTTTCTAACACTGTGTANCACTGATGAATTTGTTACCCTATGGGGTAGTTTTACTATCTTGCATATAAAGTAAGTTTTTTNT[TAAAAA]AATTAACTGAAATTACTGAATGAGGGTTTAATTGAACCTGATCTCAGTGGTACTGGGCATATATTACATTATTNCAAGCAAATGAAATGTCCAAATTCNCTTGTAGATGCATGCTGAATATTACTTCTTTCTTATGTTCAAATATGTAAG

>B1:85062832-85063132|varpos=85062982

TATGGATTCTTGGTATCATTTAATGAGAGGTCCTGTAGAGCACTTAGTATTGTCAAGCTGGCGATAGAAATATGTGTTTTATTTTGCTGTTTGAATATTACGATATCTGCACAGGAGCAAATATNGCAAAACTCATTTAGAGGTTTTTT[TTTCCCC]CCCTATTGTGCCAAGATTATTTAAAATGGAATGAGCAGAGGAAAGTGTGCNGTAATGGTAAAAACACAGAATTGGAATTACTATGGACCTTAGTTCAAAAACCTCTGCCACTCAATACATGCTTATGATTATAGAAAATTACTTAATCTT

>B1:85732647-85732947|varpos=85732797

CATGATCTTGTAGTTTGTGAGTTCGAGNCCCGTGTTGGGCTCTATGCTGACGGCTCAGGGCCTGGAGCCTGCCTCGGATTNTGTGTNTCCCTCTCTCTGTCCCTTCCCNGCTCATGCTCTGTCTCTGTCTGTCTNNNNNNNCTCAAAAATGAATAAATGT[TAAAAAAAAAAAAA]TTATNGAAGTGCNCATCAATAGAGGAATGCATAAAGAAGAGGTGGTNTATATCTATAATGGAATATTATTTAGCCATAAAAAGGAATGAAAGCTTGCCATATCCAACAACATGGATGAAGCTAGAGAGTATAAAGCTAAATG

>B1:85960409-85960709|varpos=85960559

ACAAAGACCTTACTAAAACATATAACACATGCACACAAAATTTCANTCACATTTTNGTGTACCAGATTTTACTGAAATTCTCACGGGATCACATTATATACTNTATAGTTCAATACTGCATTTTAGATATTTTTGTCCTTGAAAAAAAA[ATTTTTTTTTTT]TTTTTTTTAACTTTGTTTTGTCNAGTCACTTCATGGTGGAGCCCTGTGTTCAAACTGGCATCTCTGGTCCCCGATTCTTCCATCTGTNNNNNTGAAATAGNTATTATCTCAGAAGTCAAAGCAGTCCTTCCTAGCCTTTCTATATTCTCA

>B1:86231588-86231888|varpos=86231738

TGTCATCTATATCTTTCCATGAAGATCCAAGGTTATGTGCCTCATTATTCCCCAATGCAGTTCCATCCCTCTGAAGAAACNTTTTGTTAATGATACTAGACAACAGGGATCTTCTAGTTTCCTTACCCCCCATGGCACTTACAGGNNNN[CTTTCTTTTTTTTTTTTT]NNNTTTTTTTTAATGATACCTCATGATTTTCATGTCTCTCTCTTTTTTTAATTTTATTTTAGAGAANGAGTGCACAAGTGGGGGAGAGGGGCAGAGGGANAGCNAAAGAATCTCAAGCAGGCTCCATGCTTAGNNNNNNNNNNNNNNCAG

>B1:86792625-86792925|varpos=86792775

CTGTTACCAGAGGACGTAGGCATGGTAATTGAGAAATACAAAACAACAATACATAAGGTGGATTAACATAGTTTACCAANNAAAAAATAAACACTAGTGCTTTTTTGTGGAGAAAAAAGGTTTTCTTTTTCTTTTTCTTTTNNTTTTTT[TTTAAAAAAAAAA]GGTTTTTAATGACACAAATCTGTGTAACATCAACCTGCAAAATGTCCACGACTGGAACTTTATTTTAACCAACGGATGCCACACTGTTTCTATTTTAAGGTTGGGTGACTCCTGCCCCTTGCCAATCAGCCTTGTCTTCTTGTCTTT

>B1:87561737-87562037|varpos=87561887

TAAAAGCTTTTTCCTAGGTCTATACTTTCAAAGCAAACTTTCATTTCTCCTTCTATATACAAGATCTAGATAAAACAACTCTTACAGATGACACACAAGAATGTCTAAATAATCTCCTACATGATGAAATCAAATGGAATCTTCTTCTT[CTTCTTCTTCTTCTTTTTTTTTTTTT]NNTTTTTTTTTAAGTTTATTTTGAGAGAGCAGGGAGGGACAGAGAGNGGGGGAGNGAGAGAATACCAAGCAGGCTTCCACCAACAGCCTGCAGTCCTGCAGGGAGTCCTGCAGGGNCCCACAAACCGAACNGTGANATTATGACTGGAGC

>B1:88023332-88023632|varpos=88023482

TCTTTTGTTTTGAGTTACATTCTCCATAACACCCTANCTACATATTTGATTGTACCCNTCTTAAACATTGATAAAAAGACAGAATTACTTTTTCTTTTTTTGAGTTGTGTTTTCTCCAGTGTNGTCACTTACAACAAAGAANNNNNNNNN[TTTTTAAA]AAGTTTATTTATTTACTTTGAGAGGGAGGGAGTGCTCAAGTGGGGAATGGGTAGAGAAACAGAGGGAGAAAGAGAATCCCAAGCAGGCTCNGCACTACCAGTGCAGAGCCCGATGCGGGGCTGGAACTCAGGAACNGCNAGTTCGTGACC

>B1:89092618-89092918|varpos=89092768

TGTCAGGACAAANACGGACGCGGGGCTCAAACTCCTGAACTGTGAGATCATGACCTGAGCCAAAGTCGGACNCTTCACCAACTGAGCCACCCAGGCACCCCCTGTTTTCTAATTTTAAATGGCATCTTNAATAATCTTCATATGAGGCA[ATTATTATTTT]NTTTTTTTGTAGGAAAGAAGAAAAACTATCCTTTACATTANNNTACTTTCATCAATCTGAAATAAAGCTAGGCAGTAAGTCAGTCTGGAAGTCTNNGAAAANAGAGAGAAAGACAGAGTAAGGGAAAGAGAGAGATAGCAATTTGCAGAGGCAATTG

>B1:89144605-89144905|varpos=89144755

CACTATAAGAAAGCATTATTGATCNTTACACATAGCAGACCTTTAAGACAAAACCAGTACAAAGGAGCATAGAAGATGCTTTCACAGCTTGTTGGTAGGCATGTGGAAAGCACATGCAATTTTTCTAAATGTCAACATGGTAAAATAGA[TAAAAAAAAAAAA]AAAAAATCAATCTACTGTTANAATTTACAGTTAGAAAATTACCAGATTTACATTATTCTTTAGTGTGTTTATAGTTTGGAAAAAATGGAAACAACATAAGAATAATTCACAATATGATTGATTAATTAANNNNNATATATTTTNAAAATATCTAGGCAA

>B1:89286553-89286853|varpos=89286703

TGTTTGCAAAATATAAACCAAAANNNGTCAATGAAATTTGATAANAGAAAANAATTTTTGAGACATTTAATCTCAATATTATCTCCNTTGCCTTTTTTCTTNGGAAGTNACTAGTTGNTATGTTTCACCCAAATAAGGAAGTAAACCAAAAAC[GAAAAAAAAAAA]GAAACCTATGTGACNTAGGAAGAAGATTCCATGTAAAATGAGGAGAAAGGTATCTCGAGGATGAGAGAGGAAGATCNNNNNNNNNAAGCCTCANAGCCTTCAGCCAAGACTGGAGACTNNNNCCCGGAGAAAGNGTTCCCTCA

>B1:89318018-89318318|varpos=89318168

AACTGATANAGGAATACATTTGCTCAAGANATTGCAAAAGAGAGCAACACTAAAGAACAAAGACAAAAACTTTGTGATTTTAAAGAAGAACCTTATTTNAAAATTCCTTGCATTTGCAAGTGGAGGTAACTGAAAAACTGTATGAAAAA[AAAATATT]TTGTTTATGGAATCTGGGGCTTTTGGGATTATGTTTTTATTTTAGTACTGAATGGTTCNTATTTTCGAGTCAGCATTTCCCATTAGACTANAGTTTCCTAAGGCAGAATAATGCTNCATTCATTTCTGTATCCTGAAAGGTTAGCAGACT

>B1:89435715-89436015|varpos=89435865

CTCTTCGGCATTACGTANANCGAGTGTCTGTGTACTTCATAGTAAGAAGAGATGTGGAGGACATACAGCTCTTTTGTTAAACCCCNATGACCTTTTTAAGAATCACTTTAATCATTTTNNNGATGGGAAAAGTAAAAGATTTGAAAACAAAAAA[AAAAGG]GCAACAGAGGGGCATCTGGGTGGTTCAGTCGGTTAGGNGTCCTCGTAACCCCTCTCCCTCAAAAATAGANGAAAAANGNACAAAAAGATGTGTCAAAGAGTCTCAAGAGTAGTGTGGTATGTAAAGAAGAATTTCTTACCAATTTCTCAT

>B1:89465764-89466064|varpos=89465914

ATATTGTATTGTCAAATGCTTTTTCTGCATCTATTGAAATGATCATATGGTTTTTACTTATTGTCTGACTGATGTGATGTATCATGCTTGTGAATATTGAACCACTCTTGCAACCCAGGAAAAATTCCACTAGGTCATGGTGANTNNNNNNNN[TTTTAAA]AATGTATTTTTGGATTTGGTTTGCTATTTATTGTTGAGGATTTNTGCATCTATGTTCATCAGAGTTCTCTTTTTTTGTGGCATCTATTCGGATTTGATGTCAGAGTAATGCTGGCTTTATAGAATGAATTTGAAAGTTTTCCTACAATAT

>B1:90088527-90088827|varpos=90088677

TTATTCTTTAATAGTGCAACNTTGACAAAACACTGTTACTNTTGTCCCCTGACTCTGTATATTCTAAGTTCCATGAACACTTCACCTTGTTTATGGTTATAAAGCTAGAGCCGANCACAGTTTTCTAAAACTGACTTTATTGGAAAAAA[AAAATAT]TGAGACATTAAATANACCNTTAATACTTTAGCTGAATTATCTTTTGCTTAAACGATAGCTAATTAATACACCATGATTTTATTGTGATATAATTATGTGTAATTNATATGTAATGAATTGTGATCTAATTTAATGTGATGTAATTATGA

>B1:90371082-90371382|varpos=90371232

AATGCAACTNNNNNGGAAGTGCTTATGTCTACCAAAACAACATATAAAATGTTCACNGATGATAATTTTAATAGACAACANCTACAAACAACCCAAGATCCATCAAAATTAGAATGGATAANCTTTTTTCCTTTCCTTTTCTTTCTTTT[GTCCTCTTCCTTTTCCTTTTCCTTTTCCTTTTCCTTTTCCT]NNNNNNNTTCCTCTNCCTCTNCCTCTCCCTCTCCCTCTCNTTNTCCTTCCTTTCCCTTCCATTCTCTTCCCCTCCCTTCCNTTCCCTNNCTTTCCTCTCTTAATAAAGGAATACAC

>B1:90701653-90701953|varpos=90701803

GGCTAGATGGGTGATGGATACTAAGGACAGCATTTGTGATGAGCACTGGGNGTTGTATNTAAGTGATAAATCACTGAATTCTACTCCTGAAACCAATATTGCACTGTATGTTAAATGACGGAAATNTNNNNNNNNNNNAAATTTCATTA[GGAAAAAAAAAAA]AAAAAACNGTTGTCCTATTTCTACATGTTTCATCAAAATCTAAACTTTTTAGTACATGATGAATTCAACTTTATTATGACACAGGAAGTTTATATCACAAGAGCCTTAACTTTTCAAAGTAATTTAATTAGAAATTTAAAAACAAGTAAA

>B1:91050335-91050635|varpos=91050485

ACGACTGTAGAGGTANACACTGGACANAAAAAAAGTNTATTCTCAAATTTACTCTTAAGTGATTTGGAAAATAATATCACAATGTANCAAGGTATTTTCTTATTCATAGATCATTCCCTGTCTTTCTCTGTCTAGTTCACACACACAAA[TACACACACACACACACACACA]GTGTAAAAACAGTAAAAAGACAAAATTATGCACCNGAGTGGCCATTTCTAGGGACAGTATCTAGGATCAATTTTAGTTTCTATTGTATTTAAATTTTTCTTCAATAAATATATATGTGCATATTTTATAAAATTGAT

>B1:91405309-91405609|varpos=91405459

AGACCTGCAGGATGACTTAATGAGTATGCAATGCAATTCTCTTAAGTCAAATTAAATTTAAGCTGTCTATGTTCCATACTGCAATAGTACATTGTATATGATATAAATTGAGAGTGCTCAAAAAAGGACGGTTGTATGTCTTCCATANN[TAAAAAAAAAAAAAAA]TGCAATATAATTGGCNAGCTATGGATTTTTACTCCTNCCACCGAGAAATTTCTGAGTTATAANATTGCAGATCTATGGCATTGTGTCCCTGAATATGAACAGCAACAGGACCTAAAAAGATCANAGCTCAAGTTTGCCTTTGA

>B1:91495036-91495336|varpos=91495186

ATTGATAAGTAAAGGTATTTTAGTAGAAATATATGTCATCATTTTATCTAAGCATGCCTTTGAACTTATTCTTAAACAATGTTCAACCATAGTATAGTATTCTAAGTTACATATATCATCTATCAGAATGTTGAAGTGATTATTTCTGN[GTTTTTTTTTTTT]TTTTTTTTNCAGTTTTATTTTGCTCTTGGTAAGTTTGCAGTCACTTACTGAGATTTGTCTATTGGAAACCTATTTCTCTGGATATTTAGGAAGTCCTTCACTGTAATTGATAACAGTTTCACTATGATGTGTCTNCATCTGTTACTGTTG

>B1:91504006-91504306|varpos=91504156

TATCTATAAAGTGAACATATTCTATCATAGATAACCTANGTATGGCCCTCTTCTGAGTTCAAACCNAAGGGGCTTAAATGATAGAGAATTCANTTTCCCTTTGANGNCATATGCATACATGCAACTAATAGAGAATTGAAGAAGNNNNNNN[AAAAAATT]TAATATTCAATTTCTATCTTATTCAAGTTTGTCATATTAGTTTATATTATATTTANACTCAAAGTCATTTAAACAATATGAGTNACATCAGTGCTGTTATTTCTGTTCTACTCCTACTCATTTCCCTGACTCACTTCATAGATGTCACTC

>B1:91810273-91810573|varpos=91810423

GAATGTAAGTAAGTTTTCATGTAAGATTTCCATAATTTTAACTCTTCTTTCAAAAAATGTTAACATTTTTTNNNNNCTATTATGATAAAATATGTATAATATCTAAAATTTATNTCTATTAAATAANNNGTCAATTGAAGAGACANGGTGAGATA[ATTTTTTTTTTT]NAAATNCTCCATTAAAACAAGNGAAGGCAAAGAAAGAGATCAGAAATCTACAGNTAAAATCATACTTAATGGTGAGAAAATAGATGCTTTCCCTATAAGTATGGGAACAAGGCAGAAATGTCTTTCTCACCTCTACTCTTTAGT

>B1:91850681-91850981|varpos=91850831

GAATAAACTTTGTGTTTTCTCTTGTTAATATGTCCTTTGAATTTTGACAGTTAATTCAAAGNCCCCAATCACTGAACCCAAGTGGGTANATGAAAAAGTTTTTAATTCCTTCCAACATCACACTGATGGAAATTAAGTAGTAAACATAN[GTTTTTTTTTTTT]TTTTTTTTCATCCTTTCTTACAAGTAAGATGCATTGATTCTCACTTTACACAGAATAGCATATAGTGTAGAAACACTACAATAATTTTCAATATTCCTTGGCAATTAGGCAAATAGATTGACGAATTAATTGATAATTAGCTTTGGAAAC

>B1:92358679-92358979|varpos=92358829

AATCAAGTCTCCATTTGCTCTACTGTGGTGTAATTGTGGTAAGTTGTTTCCTAATAACTTGTAAGTTCACCTACGTTTTTGCAAATATCAGTTGGGNNNNNNNNNNCTTAGTAGGCAATAATAATAACCAGATCTGATTAAGAANNNNN[GGGGGGTGGGG]NNNNAGGAAGCCTCTTTAAAGCTANCTGAAAATCTATAAGTTTACAGNTTCAATTGTTATAATTAGAAAGAGAACAAAAACATAAAAATCAGGATCACAAATATTCAAATATTCAGATACCAGTGCTAGCATGNNNNNNNTTGTAATGTT

>B1:92557276-92557576|varpos=92557426

GATTCAACTATGTATGGTCTATGAGAGACTCTTCTTAGATATAGACACAGCTAAAGTAAAGAGATAGAAAATTATATTTCATATAATAGTAATCAAAACAGAGCTGAGTTGGCTATGCTAATATCAGAAAAGCAAAATAGACTTTAAAT[TAAAAAAAAAAA]NCTAACAAGAGACAAAGGACATTATATATTAATAAAAAATTCAATGCACTAAGAAAATGTAATTATAAACATTTATGCATCTAACTATGGATCCTCAAAACTTAAGGTACAAAAGTTGACAGAACAAGGGCACCTGGATGACT

>B1:92742335-92742635|varpos=92742485

AAAGAGTTATAATAGGAGGGGCTCCNTGGGTGNCTCAGTCGATTAAGNGTNCAACTTCGGCTCAGGCCATTATCTCAGGGTTGGTGGGTTAGAGCNCCACATGGGGNTCTGTGNTGAAAGCTCAGAGCCTTGAGCCTGTTTCAGATTCTNN[GTCTCTCTCTCTCTCTCTCTCTCT]CTCNCTCTCTCTCTGCACCTCCCCGCTCNCTCTCTGTCTCTCTCTAAATAAACATTGGGAAAAAANTTTTTTAAAGAGTTATAATANGGGAGAGAACATAAACAATCATGGAAAAGGTAGTGACGGACAAAAGAAAAATATATTCATAA

>B1:92873763-92874063|varpos=92873913

GTTGTTCATAAAGCCCTCATCTTAACTTTTTATGGTCTGTANAATTTTTAACTGTTCATATTTTCATCTGTGAGATNGAAATCACTTTCTTTTTTTCTTCTAGTTCAGTATANTCATGATTTATCAATTTTACTTGTTTTTGGGNNNNA[AAACCCAAC]NNNTCTTTCTAGTTCTCTTTGCTGTACAGTATTTTATTAACTATGCANTTTTCTATTTTATTTTCTTTCTTCTAATTTCTCCNTGTAAAANTTNCCAAACTTTTTCCACTTTCTTGAGGAAAATANTTAATTTTTAATAGGCTTTCAGA

>B1:93262183-93262483|varpos=93262333

CTTGACCTAAGCCAGCCTTCTTTNGTGAACAGTGATTGATTTGTTGGGCATATTGGGTCAGAGTTCTATTTGGGGGAATANAATGAATTAATTATAAGAGGACTTCTACTTTTCTTTGATCAGGAGATATAAAAACTATGCAGCCCNAGTGA[ATGTGTGTGTGTGTGTGTGTGTGTGTGTGTG]AGTGTGGTGGTGGTGAATCTATCTAGAAAGCCTGCTGAGAGAACCAAGNCTACATATTATACCACAGAGCTAACAGATGATAGATCATGAGAGCAGTAGATGGATAATGAGCTGGATCCT

>B1:93515804-93516104|varpos=93515954

CAACATCATATCATTTACNTTCTCTGAGAATCTATTATTTCAAATATTATTTTTAATTCCAGGATCACCAATTATTTTCTTTTCATTGACTTGTGTTTCTATTTGAATAGTCACAAGTTGACTTGTTTTGGATGTGGGAAAGTTTTATT[TAAAAAAAAAAAA]NGCCTCTTTTAATTTCCCATATTTCTCTACTTCCCTTTCACCTCATATACTCAAAATATTTACTCATATTTCCTTGTTGTCATTCATTTTATTTTTATTTCATATAACCTAAATGGCCATCACTTGCATCCACATATCCCAA

>B1:93624075-93624375|varpos=93624225

CCTCATTTCTTTTTTTCTTTTTTAATTGGAGAATGGCATTAGAAACCAAAANCTGAGGAGCAGATGTGTTTATTGCTACTGGGGNNNCATCATTGTTTCAAAAACTTCTTGGCTGACAGAGAAAAAAATATGTACTTACCATTATNNNTATAT[ATATATTTTTTTTTTT]CCTGTCTTTCTGTTGCTTTANGGGCCTTTTAAAAATTGTTGAATAATNTTTCATTGCATGTGTTTATTTATTCACCTACTAAAAGACTTCTTGGTTGCTTTCCATTGTTGTTAATTATGAATAAAAATGCTATAAATAATTAT

>B1:93659627-93659927|varpos=93659777

CANCAAAAATTTGAAAAGACAATTCATCCAAAGAGATACAAAGATGGCATAAGCACATGAAAAGATTCTTAACATAATTACNCACTTGGGAAATTCANATTTAAATCACAAAGGGATACCACTACAAACATAATAGAATGACTAAACTT[TAAACAAACAAACAAACAAACAAACAAACAAACA]CAAACAAACAAACAAACAGAAACNCTGAAAGTCACAAGTCCTGGGAAGGAAANCATTTAGAGATCATCTGGAACTCTCATACACTGTTGATGANTTTTTAAACCGTACTGACACTGAAGAAAATCAGGNAGTTTTTGTAAAATTAAA

>B1:94694921-94695221|varpos=94695071

TTGACTTCAGCTCAGGTCATGATCTCACTGTTCGTGGGTTCGAGATCCATGTCAGGTTCTGTGCTGACAGCTCNGAGCCTGGAGCCTGCTTCAGATTCTGTGTCTCCCTCTCTNTCTGCCCCTTCCCTGCTTGCACTCTGTCNNNNNNN[TTCTCTCTCTCTCTCTCTC]TCTCTCTCTCTCTCTCAAAAATAAATAAACATAAAAAANNNNNNNGTTGTTAAAAGAATGAGGGAGTGTCTGCTTTGTGTGAATGGAGGTCTTGGTTCTTGGATTCCCCATGAGATGATTTTCATGAGGGTCCACGCTTCAATAAAGACA

>B1:94807186-94807486|varpos=94807336

CCCAGGNGCCCAAAAAATGTTCTATATCTTGACAGTGGCTGTAATTACATGGGTGTAGACTTTTGTCAAAACTCATGAAACTCTAAATAAAATAAGTACNATTTATTGTTTATAAGTTATACTTTAATAAAATTGATTAAATGGAATTT[ATTTTTTTT]ATTTTTTGGAGTTTACTAGCTAGTACTTAACCAGAANTTCTANTTAATTTGGTGGCAATNTTTTNAATGTTTATTTATTTTTGAGAGNGAAAGAGTGTGTNCACACGNGNNNNNAGAAGGCCAAAGAGAGGGAAACAGGATCCAAAGNGG

>B1:95234014-95234314|varpos=95234164

TCTTCTTGGTTAAATTTATTACTTTATTCTTTTTTATTCTATTGTCAATAAAATTATATTTTTTCTGATAGTTCATTATTAGTATATATAAATACAACTGATTTTTGTATTAATTTTGAATCCTGCAACTGTACTGAATTTATTTAGTA[GTTCTTTTTTT]NNTTTTTTTTAAGATTTTTAAGTTTTTATTTTTTTAAGTAATCTCTACACCCAACATGGGGCTTGAACTCAANCAATCCTGAGATCAAGAGTCACATGCTCTTCTGAATGAGCCAGCCAGGTACCCCTGAATTTATTTATTAGTTCTAACAG

>B1:95370605-95370905|varpos=95370755

GCGAATTTAGATGCTCTACAAAAGACACTGTCAAGAGAATGAGAATACAAGCCATAGACTGAGAAAAAATATTTTCAAAAGACACATCTGGTAAATGGATGCTATCCAAAATCTACAAAAACTCTGAAAACACAATGATAAGGAAAAAA[AAAAAAAACAA]CTCAACTACAGCTTGGCAAAATATCTGAACAGACAACTTATCAGAGAAGATATACAGTTGGGAACTGAACACGTGAAAACATATTCAANATCAGTTATCATTAAGAAATTCAAATGTAAAGGGCAGTGNTATACCACTATTAAAATGA

>B1:97692109-97692409|varpos=97692259

ACTGGGCATTGTCTCATAAAATGTCAGACTGAGAGCCACATTTTGGGAAATGATTAATAAGAGTTGAGGAANTTTGCAAGTAGCTGCCATGAAGCCAGAAATCTGTCCCTTAAAATTTTTTTTNNNNNNNCTGAAGTCCTGAGTAATATTAAATGTA[GTTTTTTTTTTT]TTTTTTTTCCATGCTTTGTAGGCTGATTCATGTAAGAAAGGTTATTTTAGNNNNTAATNNNNNTTTTTATTTACCATTTCCATGTGTTTATATATGAAATGTAGGCAATTATAACTCAACTAATTTAGGCTTGGAAAACACCATATGTGT

>B1:97785807-97786107|varpos=97785957

TTTACCCAAAGAATACAAAGCACTAATTCAAAAGGATACATGCACCCCTATGTTTATAGCAGGGTTATTTATAGTAGTCAAGATATAGAAGCAGCCCNAGTGTCCATTGATTGGCGAATGGATAAGAAGATGTTTTATACACACACACA[CACACACACACACACACATACACACACA]TATGTACATATATATGTATATATATTATACACACGCATNTATGCATGCACCATGGAATATTATTCAGCCATAAAAGAGAATGAAATCTCGCCATTTACAATAACATGGGTGGACTAGAGAGTATAATGCTAAGTGAAATAA

>B1:99152846-99153146|varpos=99152996

TGGCTCCATATTAACGTANNNNNNNNAGTTTCAGGTTCTATTCGGAACAACACAATCTGGACTCACTGGCTTCTCTACCCTCTCCCTTGCTGGGCTCCTTNNNNNNNNNNCAAAACAAAAACAAAAAGACAAAAATAAAAACAAAGAAA[CAAAACAAAAAAAAAAA]AGTATTTGCTTCAGGTAAAGTGATTTAATGCTCTTTCACACTTTCCTCACCTTGGTTTTTCTGGCTGTTCATTCTNCCTGGAATGCCCNNNNNNNNNNNGACTTTTTGTAAGACTCAGATCAAGAGTTGTCACCATCATGCCTTTG

>B1:99865821-99866121|varpos=99865971

CTCAATCCCTTAACAAAATAAGTAAGATTAATAGCTTCCAAGATACNGTTTACATTAGGAATACAAATCTATAGNCCCACTTGCTTCCTAACAAATAGGGAAAAATAATAANNNNNCATTTGATATTATTTGTGTGCGTATACATATGTGTGTGTGT[GTGTATATATATATATATA]ATATATATATATAAACATTAATNTTGTATTTTCCAACAAACATNTGATGTTTATGAATCCAACTATTGATTTAAGCAGAGGCGAAAGCCAGAGGTATCCAATTTTCTCTATAGTCAATAAAGTAAACACAAATTGCTCTGCTTTGACAA

>B1:100380178-100380478|varpos=100380328

TAATGTAATGNNCCCAGCATATTATGAGTGCTCTCTAGATAGGTATTTTACTATTATTATCCCTTACACTTAAATANTGGATTCACCTAGAATATTTCCCTGAGAAGTCAGAAACACCTTTTNATTTTTTAATTTGTTTACTTTTTATT[TGAGAGAGAGAGAGAGAGA]CAGNNNNATAGGGAGAGAGAGAAAGAGAGAGAACACGAGTGAGGGAGAGGGGCAAAGGACAGAGAGAATCTTGAGCAGGTTCCACGTTCAGTGCAAAGCCTGATGCAGGGCTNNATCCCACAACCCTGGGATCAGGACCTGA

>B1:100810771-100811071|varpos=100810921

TTTAATCTTCTACTCTGNAGTTTTCCCTGTGGCTAACTGCCCTCCTGGCTGCAAGATTAAAATCATACTTTTCATCCACACATGCAGACATGGCAATGTCCTGGGGAAGGAAAGGGATAATTAATTCCTTATTCACCTCTCTCTCTCCC[ACTCTCTCTCTCTCTCTCTCTCTCTCTCTCT]NNNNGTCNTTTGACTACAATAATCTTTAATAATCAAAATACCAACAGCAGATTTCTCTTCAACGTGATCACTCCATGAGCAGACATAATTGTACTATTATACAATGAAAATAAATTAANTTCTGCTAATGGAGT

>B1:101875009-101875309|varpos=101875159

AANCCATTGTCATGAAAAGAGTAAATGCCACTAGAACCAGAAGTCTTANGATACAAGGAATATAGCTATTCAAACAATGTTCTGGCGAAAAGTTTAAATTTTGAACAGACCTCAATTATCTGCGTCCCTCATCAATGTGGTAAATACNT[AGTGTGTGTGTGTGTGTGTGTGTGTGTGTGTGT]NNNNNNAATAGTATACACTATTATAAATGTACTGATAAATAATAGAATGGTAGAGTANNNTAGTATTGTGACAATGGTGTTAAAATATTATAAGAATTGCATGCAGGGAAAGGTTATTTATAGCANTGCTTTAG

>B1:102221489-102221789|varpos=102221639

GATCACCATAACAAATNTAGTAATAATTGAAAAGTTTGAAGTATTGTGAAAATTACCAAAATGTGACACAGAGGAAGTGAGCAAATGCTTTTGGAAAAATGGCACCAATAGATTTGCTTGACTTAGGGTTGCCACAAACCTTTAATTTA[TAAAAAAAAAAAAAAAAA]TGCAATATCTGCAAAGTGAGATAAAGCAAAAACACAGTAGAATGAGCCATGTTTTTATACAATACAGTGTTGCTAACTATGGTCACCATGGTCTCTACATTACATCCTCAGGACTTATAACTGAAAGATTNTACCTTTTGAT

>B1:102391295-102391595|varpos=102391445

AGAACCTGCTTGACATCTTGATAATTGCCTTTATTATATAACTCTGAACCATGGACAAATTCAAGCACAAATATAAATGTGAATAAACATTGGTTTTTATCCTAAATTCCAACATTAAAATTTTNAAATGANAAATAAATGTCTAATTG[ATTTCTTTTTTTTTTTT]TTAATGTTTTTATTTATTTTTGAGAGAGACAGAGCACCAGCAGGGGAGGGGCAGAGAGAGAGGGTGATACAGAATTTGAAGCAGGCTCCAGNCTCTGAGCTGTTAGTCCAGAGCCTGACACGGGGCTTGAATTCATGAACTGTGAGA

>B1:103506549-103506849|varpos=103506699

CTCAGAGATGAGATGGTAACAAGACTATNGAGTTACTATAGAGAAAACAGCACTTAAGTGAGCTTTGTTGGTGGTCCATTTAGACAGATGGCCCACAGTTTCCAGAGCATTTGTGCTTTCTTTAAATAAATACCCCCAACACTGAGGGG[GAAAAAAAAAAAAAA]AAANNNNNNCTGAAAAGAAAGGTGNTTAACTTTTTCGTTGTGCCAGTGATTTATTATTTAAANTTATAACCTACTGGCTTTATTCAAAGGNCTGGTTAAAATTGCACAATGCCAGATAATCTATGTCAATCTCTCTATAACATAATTGTAT

>B1:105858312-105858612|varpos=105858462

TCTGCTCCACTGTGGATAGAGAATCTCACCTGAGGTTTGCTCACTTTACTCATGACAATGCAATGAGTATTCAGATGGCTTGATAATAGNTTTTGAACANTGATTTCTAATACCTATTAAATATCTAATTTCTAATAAGAGGGACCTGG[CATTATTATTATTA]NTTATTATTATTATTACCAGTATTATTGACAATTTATAAAAGAGCACAGAGTACGCTCTGCAGGGCCAAGGTGAGCATGTGCTCCCTTTTTTGTCCCAGGACNTACACTGCTCATTGGCCTCATATCTGGCTGTGAACTCCTGACACACT

>B1:107330011-107330311|varpos=107330161

ATATATTTAAATTTCTTGTCTCTTTNGGAATCTTGGTTTTTTACCTTGTTTAATTTTTNCACTAAATGTCAACTTTATTTTAAATTTTGATTTCTACAAGAATGTGCTCCTCTACCAAGCTTATGTACTTTCTTAGGATTTCTTAAATT[CTTATTTATTTATTTATTTATTTATT]NNNNCATTCATTCATTCATTNATTCATTTATTGCATAACACTCTTCTAGATTCATACCATATATTACAGGCTTAAATTTTNAAAATANCATATAGCTTGTTTTGCTCTATTGTTTTTTCTAGACTGTATT

>B1:107610365-107610665|varpos=107610515

AACAGTTTAGAAAAATGACCACATAATTTAGTGAAACTATGCTATTAAGTTACCATTAATTTTTGAGTTGCTGCCTTTCAGTCNTTCTTTNGTTTTGTGTTTTTTAACATGTGGTAAATATAAAGCATCAGTTTTTCTGTATATATANN[AAAAAAAATA]TATATATATATTTTCTGTAAATATAAAGCATTCAGTTTCGAGGGAGAAGACAGTTCCTCCCTTAACTTATTTTAAAGCAGTTTCCAGATTCAATTGGAATTTTCTAATAGCTTATTTATTAATATATTATAAATATTTACCNAATATTC

>B1:107898377-107898677|varpos=107898527

TTCTTTTTGAGTTGTGTAGCCCTTTANATTAGCAANTCAAATAGAAGGTGTCTATTGTACAACACAGTAGTAATTGAAGATAACTGGGGATTTATTACATTCTAATACAGATCATTTACAAATGCAATTTCTTTATTAAAAAGCTTCCA[CTTTTAT]NTTTTTTGTTTGTGTACAATTTGCAAAACTATTCTGAGCNCGGAATTATGTGCACCAGTGTGCAAAGAGTGCAGATTTAGGATATGGTAATGGGGGCACCTGGATGGCTTAGTCAGTTAAGCATCCAACTCTTTAATTTCAGCTCAGGT

>B1:109209484-109209784|varpos=109209634

TCAGAGAGTCTCAGCCAATTGTTCTGGAGCAGCACCTGGTATCTGATACTTTTCTAAAATTTTCCAAGTGGCACTAATGTACAACCAGAATTTAAATCATAGGCTTACTCTACCTAACCTCATATGTTAAATGTATAAAACATTTTGAA[ATGTTTTTT]TTTTTTTTAAAGCTTTGGTTAAGTCCATGATTTGGGATTAAGTCACATGAAATAGTTCCATTTAAATAAAAATTCTTAACCNAGGTGAGTAGTCATGCTGGTTTAATATTTCATGCTTACCAAAATTCAACTTTATGACTGGAGTTTCA

>B1:109906578-109906878|varpos=109906728

CATTTCCTTTTACCCGCTTTCTCTGGGCNAGACAACCAATNTTGATATTTCTCTGAGCCAGACAACCAAACTTGATATATAAGTGGAAGAAAAATAGGTAGCTAGAGGCTTCACATTNCTAGGTGTANATCCTCTACAGAGAAAAAAAA[AAAAAT]NTACCACTTAAATCCAGATCTTTTATTTATTCCTTTCTTCCAAGATAAGAAACATGAGAACTTTGTAGGAAAATTGTTCTCTTCAACCAGCCACGTTGTACTTATTTTTCTACAAAAAGAAAGTTGGACTAAATCAAAGCAAGACAGCAA

>B1:111429186-111429486|varpos=111429336

CTCCTTGGCTTGGGGCGCCTGGGTGGCTCAGGAGGTTAAANGTCTGACTCTTGGTTTTGGCTCAGGTCATGATCTCACAGCTGTGAGAGTGTGGAACTTGCTTAAGATTCTCTCTCTCCCTCTCTCTGGGTNCCATCCCTTCTTGCATG[TTCTCTCTCTCTCTCTCTCTCTCTC]AACAATCAATCAATCCACTCCTTGGTGATCTCATTCAGTTCNTAGTTTTAAATATNATGTATTGGCTGACAATCATCAACTTTGTATCTCCAGTCAAAACTTCTGCCTTGAAATTTTCCGATGCATATTTTCAACTGT

>B1:111875484-111875784|varpos=111875634

AAATGAAAGACACAAATGAAAGAAATGTTTGTTTGCAGTCACNGTGTCTTTACAGAAGATAGGATTCATTAGAAGATAAATGTAAATTTCTTATTTCCATATTTGGAATAATTGATTATCANTTNNNNNGATCTGAATGGATAGAANNN[GTTTGTTTTTTTTTT]TTTTTTCTTTCTGAAAAATCATAGATTGCAAGATTTTTNCCAACTTACCAAAGNTAACNTAATCATANATCTCATAGTTAAGNAATTTCTGTTTCACATACCAGGTTCATCTTAGGACCAACTGCAGTATATTGGTTGATTTGTCAC

>B1:115302171-115302471|varpos=115302321

TGTTGTAGTTCCTGAGTTNTAGCCCTGTGTGGGGCTCTGTGCTGACAGCTCAGAGCCTGGAGCCTGCTTTGGATTCTGTGTCTCCCTCTCTCTCCCACCTTCCCCTGCTCACACTGTCTCTCTCAANAATAAATAAACATTTAAAAAAA[AAAAAGAGAGAGAGA]AGAGAGAGGAATAGAAGAAAAAGTGTTAAAGAGTGACATTCTTCTTAAAAATTAAATGTTNTCTCACTTCAGTTAGCTTGATTATAATCGTTAANGTTTCCTGATGTTTGATTTTTGTAGGCTGATGAATTGCTCNGATCCCTNTTGTTC

>B1:115405699-115405999|varpos=115405849

CAGTGAACATAATAGTATAATTGTGATTGAGTTGCATGAAGTTTTTACATTTTATTCATTGTTAACATATGGGAAAACAAATTATTCATCTTAATTTCTGTGTTTTGAAAAATAGTGACTTGTCCTCACAGAAAGAGGTGNNNNNNNNN[TTTAAAAA]AACTTTTTGTTCTTACAATGTATCAAAGTTTACTAAGTCCTTGGTTAGTCACATTACAAAAATTATAGTGCTGAATGCTCTAAGGGAAAGAAATGGTTGACCAGTATGTTCTTGTCAATTTTTTCTAAACTAGTGNATACTTCAAATAAC

>B1:115815312-115815612|varpos=115815462

TATTGAACAGTGCAACTGTGTTTCTCCGTCAAGCTCTCTCATGTCCCCATGCCTTTGTTTACATGATTCCCTCCCCTTAGAATATTTTTTCTTTTCTTTTCCTCATGCTCATTTCTTTTTAATTTTTGAAAAAGTCTATTTATTTATTT[TGAGCGCGAGAGAGAG]GANAGGGAGAGAGAGAGAGAGNGCACACACATGGTGGGAAGGGGCNGAGAGAGAGAATCCCAAGCAGGCTCAATGCCNTCANTGCAGAGCCAGATGTGGGGCTTGATCTCATGAACAGTGAGATCATGACCAGAGCCAAAATCAAGGGTT

>B1:116737048-116737348|varpos=116737198

CCNACTTTGGCTTGGGTCATGATCTCATTGTTTATGAGTTCAAGCTCTACATTGAGCCCACTGCTGTCAGCGCAGAGCCCGCTTCNGATCCTCTGTCCCCCTCTCTGCCCTTCTGCNCTTGCGCTCTCAAATATAAATAAAACATTAAAAAA[AAAAAGAAAG]NNCTGTAGCAACTTCTTTCTGGATATGTTTTCTGAGGCAAGGGAAACAAAAGCAAAAATAAACTGTTGGGACTTTATCAAGATAAAAAGCTTCTGCACACCNAAGGAAACAATCAACAAAGCCAAAGACAGCCTACAGAATGGGAGAA

>B1:117378946-117379246|varpos=117379096

TGTCTACCAAANGCTTAACTGGAGTTTTGCGGGGTATAATAGAGAATGAATAGAGGCAATATTTGAGGTAACAGTTNCTGAGGACTTTTCAGAATTTTTGAAAGACATTAATCCTCAGAATCAGAAATTACAATGAATTTCAAACACAA[TAAAAAAAAAAAAA]GAAAACTACACCTAGATACAGATANCATTTGAAATTTCAACATAATGAGAGTGAATGGAAGATCTTAAGAACAGGCAGTGAAAAAGGACACATTACCTAAAAACGGGTAGCAGTTAGACTCATGGCNGCCTCCACAACAGCC

>B1:117705970-117706270|varpos=117706120

NTGTTTCAGTTAACTTGTAGGGTGTTACAAAGTTATACTTAAGGGAAAAATNAATATTCCACTTAAATATTAATAATGGAGATGTTAATAAGCCCCTTACCCACCAACAGCTATCAATCTANTATCTTCAGTTATGTGCCAGTCCCCCC[CCCAAAAAAAAA]AAAAAACTGCCAACATTCATTTATAATATAAATAATTTTGGTCTACTCAAAAGAAAAAAAGTAAATTTTTAGTGGGTAAAGCATTAGGAAAATGAGAAACATCAACTACATAGCTGTTTTAAAAGAATAGCATATAACCACATATTCATC

>B1:118821097-118821397|varpos=118821247

CTGTTGGCTGAGCACCNGACTCTTGATTTTGGATCAGGTCACCATCTCACAGCTCAGCTTGTNGGTTCAAGCCCCATATCCGGCTCTGCGCNNNNNGTGTAAAACCTGCTTAGGATTCTCTCTCTCCTCCTTCTGNCCCTCCACTACTT[GCTCTCTCTCTCTCTCTCTCTCTCTCTCTCTCTC]AAATAACTAAATATATATTGTAACATAATATAAACTTATGTAATCACTTGCAAGCTTTAATTTTGTTCTCCAATATATTTGGCAATGATGACTATCTTTAGAAAAACTGATGGAAAA

>B1:118928161-118928461|varpos=118928311

ATTTATGGGTCTGCTCTTGCTTTTTTGTTGTTTTATTTTNNNNGTTTTTTAGATTCCATGTTTAAGTGAAATCATANGGTAGTTGTCTTTCTCTGACTCATTTCACTTGTCATAATACACTCTAGGGCCATCCATGTTGTCACAAATAGCAAGATATC[ATTTTTTTTTTTTT]ATGGCTNAGTAATAGTCCAGTGTGTGTATATANCACATCTCCTTTATCCGTTCTTCTATCTATGGACACTTAGGTTGCTTCCATATCTTGACTATTGTAAATAATGCTACAGTAAACAGAGGGGTACAAATATCTTTTCCTCA

>B1:119396742-119397042|varpos=119396892

TCATGAATGCATAAAGAACTAAAAGAAAATGTATCAAAATGTTAATAGGGATTGTTTCTGGTTTTCTGTGTTATATTTNAAATTTTTAAATTTTNAAAATAATTAAATAGTATTTCTGTAATCCANAATCAATAAGAGATCCTTCCCCC[CCTTTTTTTTT]TTTTTTTAACACAAACATCTTAATTTGTTTCTGCATTACAGAAAATATTAAGCATAAGTTTTGTTAAAATTCTAGGGCAAGGACTATTTTTGTTACATTAGAATGAATTGTTAAATGAATTCTAATTTTCCTTAGGAGCTTCTGAAGAGA

>B1:119850294-119850594|varpos=119850444

TCTTGATTTTGGCTCACGTCTTGATCTAGCGGTTTGTGAGATGGAGCCCCATGTNGGACTCTGCACTGACAGCATAGAGCCTGCTTGGGATTCTCTCTCTCTCTGCCCCTCTCCTTCTCTCTCTCAAAATAAATTGATAAACTNNNNNN[AAATTTTTTTTT]TTTTTTAAGTTGAATTATTTGTTACCANTTAAAAAAGGGACATTCTGGGATGCCTGGGTGACTNGGTCAGTTAAGTATCTGACTCTTGATTTTGGCTCAAGTCATGATCTCATGGTTTGTGAGCTCNAGTACCACATNTGGCTCTGCACT

>B1:120081593-120081893|varpos=120081743

TTCTGAAGATAGCATTTTCATCCTAGATTTTTTATAGAATTTCTATTTATTAATCTTCCTTCTGTTTTGCTTCAACCTTTCTATATGATTTGAAAACTTGTTTGCCAGATTTTCTGTTGTTGTAAAAATGTTTAGTAACATTGAATAAT[GTTTTTT]GTNNTTAATTTATTGATTCATTTATTTTACCAATNTTTATTGAAGACCTCAAATCTGTGAAGTACTTGAGACATGATGGGAAATAGAAACCCTCAAAAGTTATCATCAAATNTATGACATAAGCAAATAAACANGCAATTAAAATATAG

>B1:120560115-120560415|varpos=120560265

TAGATGGACAAGAACAGGATCCTAGCAAGACAGACTAGAAAGGCACACCCTTCTACAATGCCTAAAAGTTGGAGAGATTTCTGCCCTTGGAAAGAGTTAGAAAAAAGAGGTTGTATGTTTGAATTACCTAAGTTTAAGTCANGAGAATT[GTTTTTTTTTTTTTT]TTTTTTAANATCCATGAATGAATAATTAGATTAAATTAAATNATAGAAGCATGAAAAAANNNNNTATATAAGAATATCACCATTCANTTCAAATTCCANNCCCTTGCCANTGGACTTGAGTTTCTCTTGTATCAATAGTGAGCAAATATATTG

>B1:120988791-120989091|varpos=120988941

ATCTCTTAGAATTTTAGCTATACTTGGATTCATTGGCAAATATGAAATCCTGAATTGATGGATTNTCTGTTTCATTTCTATGATTTATTTAACATAGTGAGAATTGACTAATTCTTGATTTTTAAATGAGTCTCCTTTCATNAAACTTA[ATTTTTTTTTTTTT]GGTGATTTTAATCTTTGCTTAAAAAAAGATTTTAGCATTTCTCACTATTCCATTTAGTTTTAATTAAATTTTCTACTCCTNTCTTTAGGATGATGAATTTCAAGTAATTTGCTGTTCCATCTCAAATACATTGATATGAATTG

>B1:121697150-121697450|varpos=121697300

TCAAGCATGTAGGTAGAACATTCTTACACTTAATTAAGGCTGACTTGTGTGACTCATAGAATACAGTGAAAGAGATATTGTNTGATTTCTAAGGCCTAGGCAGTAAAAGACATTATGGCTTCTACCTCAGTCTCTCNGANTGCTTTCTN[TGGGGGGGGG]NNGGGAGCCAGCCACCGCGTTGTAAGAACAATCCGGCAACCTTGTGGACANACCCAGTGGAGTAAAANTGACAGCCAGCACNAACTTACCAGCAATGTGAGTGAATCACCTCATAAATGGATCTTCCAGANCCATCACACACTTGGAGA

>B1:122110886-122111186|varpos=122111036

CTNAAGTTGGATATATCAACAGGGACAAATCTCAAAAATAAATNTTAAGGAGAATAAAAGGAAGCCTCAAGNTATTTCNGTGAAGATAATATTCAACTACAATAGTATTGCTAAAGCTAAAACATCAAATTGCTTTACTCTTGGTTGGA[ATGGTT]TTTTTTTAGCCAGTATAGTGTTTATATCAACAGTGATCAGGAGCCAGAGATTATTTTTAAATTGCAGTTTGTTTGAGAGAGATCTGTTAAAACACAAAATTCATTAAGGAGGAAACTAGTTTTAANTGGGTCCTTATGTGATGACAAGA

>B1:123555879-123556179|varpos=123556029

CGGTTCATGGGTTCAAGCCCCATGTCGGGCTCTGTGCTGACAGCTCAGAGCCTGGAGCCTGCTTCAGATTCTGTGTCTCCCTCCCTCTCTGTCCCTCCCCTGCTCATACTCTGTCTTTGTCTCTNTCAAAGATAAATAAANGTTAAAAA[AAAAACC]CAAACAAACAGTAATTGTTACAGAATAAGTCATGAGAGACTTTCAGAGACCATAAATGATTTTATATTTGTTGAGAGACATCAAGAACAAGGGTAAAGAACAATTGGCAAAATACTGATAATCCTATTCAAAATATGTCTAGCACTACAA

>B1:124677952-124678252|varpos=124678102

AGACCTAATGGACACAGAGTTACTGGTAACTCATGATTTATGTAGCCTTCTTTATNACAAAGAGCAGNTTCAGTCAGGTCCCTATCTAAGAAATTTGAAGCAATAATACAGTTACAGTGAAAGGTATATGGTTGAGTTAAAGAAAAAAA[AAAAAGGG]GGAGGGATAAGGTAGAGTAAGAACTGCAGTGGCAAATATGAGTCATTGTGCATGAGAGGTTATGAGGGAGAAAAACCTCAGNGGAAAACAGTTAAGAGAGAGATGAATGAGATACACTCATGGAAAGTGGAGCACTAAGAGAAATCCTGT

>B1:128030505-128030805|varpos=128030655

AGTGAACACTAGTGTGTCTACACCTATCATGACAAGGAATNTGAGGACACNCTGTAGGACCTTGGGCAGTGGAGTAATATGACTATCTACACAGAATGCTATGTACTTACATATCATACTACTATCCAATGCTTAGCACTGGCTGAGAG[TTATATATATATATATATATAT]TTTACACATATTTTCATAATTATCTTACAAATTGGNCATTATNACTGAAGNTCTCAGTAGTTANTGNCATCTCCTGGNCCTAAACTGGTATGTGATATATCTGCTATCATATTGCAAAAAGACCTGTCCACTTCCAGTGCT

>B1:129989670-129989970|varpos=129989820

TTAGTATAANCCCCTCAAAGTCAGGGGCTGTTTAGTTATCTTGTTAAGAGTANNATCTTGTTTCTAATATGGAANCTGGGCCATATTTTTTGAGTTGGTTTAGAACGATGGAAAGTATTTGTATTCCAAAGTCAGNNNNNNAAACNAAA[CAAAACAAAAAAAAA]AAAAAAANCAGAAGTTGAATTCTGACTCTGCCNTTGCACATTGTACTGGGCTGAATAGTCACCCCCAANATATATTTCCAAGTGGTAATCCAATTCATTCTGTGACTTNATCTGGAAATAGAACCTTTACNNNTATAATTAAGTTAATGA

>B1:130507454-130507754|varpos=130507604

AGTAATGTACCCATATTGGTTGTTGGTTGNGACAAATGTACTCTGCTCAGGTAGGAAGATAACAATAGGNGAAACTTAATGAGGGGTAAATGGTGTTCTCCGTACTACCTCTGCAACCTTTCTGTAAATACAAAACTTTTAAAATAAAA[AAAAAAATAT]TTTTAATTATTCTTCATTAGCAATATAAGCAGGATTTTTAGGCTTATAATACANGACTGTCATTGTACTCACAGCTTCTAAGCTTTCATTAGAGCTTTTATCACTTTTATAAAAATGAATTTTGACAGACTANTTTTTGGGGATGAGTA

>B1:131583344-131583644|varpos=131583494

TCACTTTATTGCANGANTTTTTCATTACCACCAATGTTTCTAAATGTAGCATTGTATCATTTCCTATGTTTTAAAAGAATAAGTTAAAAAAATTAAAAAGAAAACCTGGATTACCAAAATGCTTAAGGGATTGTGCTTTNNNNNNNNTG[TGTTTGTTTGTTTGTT]TTTTTGTTTTTTGTTTTTTCTACTCTGATTTGCCCAAGAAAATCAGATCCCNCTCTCTTGTGGGCTAGTAGCACTGTGTCTTACTGTCTGGAAGTATTTATTACAATAGTAATTAATTTTGGCCAAACATTTGAAACATCTGCAACCCT

>B1:132345751-132346051|varpos=132345901

TGAGTTNGGGCTCNGCATGGTGCTCTGTGCTGACTGACNGCTCAGAGCCTGGAGCCTGCNTCAGATTCTGTGTCTCCCTCGCTCTCTGACCCTCCTCTGTTCATGCTCTGTCTCTCTCCNTCTCAAAGATAGATAAANACATTAAAAAA[AAAATTTTTTTTTT]TTTTTNNAATNGTCTTGAAGCTGCTGGGTTGGAGAGGGTGCTGTGTAATACTACCTTTAGAGAATAGAAAAATTGTAATTCGGGAGGTATTTATGTTTAAAATTAGCTATTGACTAAACTTTATAGAAAATGTTTAGGAGTATAATTAGT

>B1:132998399-132998699|varpos=132998549

TTTGCTTAAGTTCCCACTTGGAAATGCAAANTCTCAGAAAATATAAAAAGAAGTCATTCCCTGTTAAAGCTCATCAAGAGCTGACTAGCATTAATAAAAACTGTGTGTGGGAATAGTAAATAAATCAGTATTCAATTNNNNNNNNNNNA[AATTTTTTTTTT]TTTTTTTAATGTTTATTCATTTTCTGAGACACAGAGACAGACANAGCATGAGTGGGAAAGGGGCAGAGAGAGNGAGACACAGAATCCAAAGCAGACTNCAGGCTCTNAGCTGTCAGCACAGAGCCGGATGTGGGGCTCAAACTCATGAAC

>B1:133313903-133314203|varpos=133314053

GGAAAGGAATTAATAAAGNNNNNNNTAAAAATTAAAAAATTAAGAACAANGCAAAAAAATAAAATAAAGGATGGTAGGTCCTAGGTGTGTTTGGTCTGGTTGTTGAAAGAAGCTTGATAGATTAGAGGAAAAGGGAAAGATAAGAAANN[GGAAAAAAAAAAA]AAAAAAAAAGGAAAACATTTGAAATTTTGAAAAAATGGATACAATAAAATAGAATAAAATGAAATGATGGAAGTAAAATAGAATTTTTAAAAATTACAAAAAAGTAAAAATAGAGTAGAAAAATTTAAAGGAAAATCTTNTTAATAAAAA

>B1:133605459-133605759|varpos=133605609

ACTCAAGCTCCCCTTGGTATTCAGACAATATTCCATAATTAGCCAATGAAAATCTCCAATGGGAATTGAAGCTATTTCATCTAGGAATTATTTTAAGGNAGAATGAAATCCTAAATCCTAGTTGCTCAAGTATTCTACCTAAAAAAAAC[AACAAAAAAACAAAAAAC]AAAAAACCTAGCCTGGACTGACCCAATATCNCAACAGTCTAAAGTTTCCTGAAATTAAAATGCTGAGCCTNAAAATATTGGGATAGTCTTAATCTGCACTGGCCATCACATCCTTCCCCAAAATAGATAGAAATTCCTGGTACACTTTAG

>B1:134332986-134333286|varpos=134333136

ATTGAANCTATTTCTCTTCAGACAGAATAAATTCTTGATTAAGACTTTTTCACATAACTAAAGGTCAAATATACTNACAAGGATGCTAAGTGAACTAGTATCTAGTGAACTAATTTATAAGCTAGAGTAACATTAATTCTAGNNNNNNN[TTTTCCCC]CCCTTAGAACTATTTGGTAGTTTATAAAAAGCAANCAGAAAAGGAAAAGGCGCCAGCCACCACTATGTACACTTTATTAATCAAGGCAATGACAAGTAATCCTTTAAATGATTTGGCCAGCTAAGACACTATTTTTAGAGGCCTACACNGAA

>B1:135087759-135088059|varpos=135087909

GGAAGTGAAATAAAGGAGTGAATCTATTGGGANGCTGATAATGACAAATACAACATTTGGTAAAGTAAAAAGGCTGCTTTGTCTCATTCCCAAACCCATCTTATTTCTGAATNNNNNNNNGGATGAACAGCCAGTCAAAATTNNNNNNN[TTTAAAAA]AAAANGAAAGAAAAGAAACTATTGAAGCAAGCTTTCAATTATCTCTCATGTCATAAATAATACAAATGAAAACAGTTTTTGGCACTTTCTTTGAATTATAAATTTGTCGCATTAAGGTATTTTGCTTTTCAAACACCACAGGTAGTGTTT

>B1:136380226-136380526|varpos=136380376

AAATTTAAAATTANAAAAATTATACAATAAAATAGAATAAAATGAAGTAAAAGAAATAGAATAAAAATTTAAAACAATAAAAATAAAGTAAAACAAATAAAAATTAAAATTAAATTTTATCTCTGTATCCAAGAATGAGGAAGGAAANNNN[GAAAAAAAAAAAA]GAAGAAAGAAAACAGTAAGCAAAAACAGAAGCAAAGAAAACAAAAATAAATCAGCAAAGAGAATTAAACCTGAATGAAGTTACACCCACATTCCCCTAGAACTGGAATTATGAAGCACTCTATAGTCCACACACTAAGCAGGTG

>B1:136799264-136799564|varpos=136799414

CCTAATAGTGCTATTGCTGGATTGTAGAATAGTTCAATGTTTAACTTTTTGAGGAACNTCCATGCTGTTTTCCACAGTGACTGCACCAGTTTGCATTCCCACCAACAGTGGAGGAGGGTTGCTCTTTCTCCACATCCTTGCCAACCCCT[GTTTTTT]NTTTTTTGTATTATTTNTTTTAGCCAATCTGATATGTGTGAGGTGATAGCTCATTGTAGTTTTGATTTGTATTTCTCTGATGATGAATGATGTTGAGCATCTTTTCATGTGTCTTTTCATGTGGCCATCCATATGTCTGTGGGAAAAT

>B1:138978298-138978598|varpos=138978448

AGAAGGGAGCCGAGGAGATTTTCACTCAACAACTAGCTTTGCAAGGAGACACAACTGTTAGGAATTACAATATATATTAGTTGAATATAATCATTTTTGTTTCATCAGAAGAGANTCTGAATAGGTTTTAAGCAAGTTGCACTNCAAAA[AAATTTTTTTT]TTTTTTATAACGTTTATTTATTTTTGAGACAGAGAGAGACAGAGCATGAACAGGGGAGGGTCAGAGAGAGANGGAGACACAGAATCCGAAACAGGCTCAGAAACTGTGCTGATGGCTCAGAGCCTGACACGNGGCTCAAACTCACGGACC

>B1:140105071-140105371|varpos=140105221

ATAATGTATATAATCGTTGAATCANTAAATTGTACCCCTGAAACTAATTTAATATTGGTTGTCCACTATCACTTNAATTAAAATATTTTTTAGAAAATTTAAAGAANGAAAAAGCATCAGACAAGAAANAAATGAAGCNNNNNNNNNNN[TTTAAA]AAGTACATCGCAATGCTGGAGGATTTGTAGTCTGTGATTTCCTCTACCAGGGGGATGAAGGACTCCAGAGAGTGTGAGTGTGGATGTGTGTGCGTTTTCATCGTTTGCATTACCATAGCTCTTAATTACAGTAATCATTTTCACAAATGG

>B1:140153201-140153501|varpos=140153351

TTTCCAGACTTTACAAAAAGCAGCATCTACTTACCTTTTTCTAGACCTAAGTTCATCTCTGAATCCCCAATAGGTAACAAAGCCAGCTGNTGAAGAGGAGATTCTGGACGACTGGAACAGAAACATAGGGGCATTTAAGTTTNNNNNNN[GGTGGGGGGGTTGTTTGTTT]TTGTTTTGGAAAGTGAGGCCCCAAGCAACCTGTGTTGTTTGTTGGTGGGCTGGTTTTAAAGATACTTTTGCTCGAAAGATCCACTAGCATCTCAGGTATGGACAATTTAGTTANCCTCTCAAGCATTGTGATTTAAGGATCTCCTGAGGCC

>B1:142010038-142010338|varpos=142010188

CTTCAATATTTAAACTGAGATGGTCATNCGTGCATTCAACCCACAAACTTTAGCCATGTGCCTACTCAGAGTTCTGCATGGCTGCACTAAAAGCTCTGAATACCAAATTCAGAACAGTGGCTGTGCATTGNTTATATTGCCAGAGATAN[TTTTTTGTTTTGTTTTGTTTTGTTTT]NNGTTTTGTTTTGTTTTGTTTTAAGGAANACCTGATTTCTTGTATAAACACTAAACGCATATCNAATGAAATAGGGATGGGGTCNNNNNCCCAGCCAAACCTGAAGTAGAAGTCCATTAGCCACAGGCTGCCTCTTCTGATTCCCTAT

>B1:142558648-142558948|varpos=142558798

CATTCTGACAGGTGTGAGGTGATATCTNATTGTGGTTTTGATTTGCATTTCCCTGATGATGAGTGATGNTGAGTATCTTTTCATTTGTCAACTGTNATATCTTCTTTCAAAAAATTTCTATTTAGATCCTCTGCCCATTTTTAAATGTAANN[ATGTGTATGT]NNGTGTGTGTNNNNGTGTGTGTGTTAAGTTATAGAAGTTCTTTATATATTTTGAATACTAAACTTTTATCAGATNTGTCTGATAAAGGTTTGCAAATATCNTCTCCCATTCAGTAGGTTTGCTTTTNNNAGTTTTGTTGATGGCTTCCTTTGC

>B1:145215875-145216175|varpos=145216025

AGAACCCTTAGTAAACAGTTATACAGGAAACTTGAAAGACANGTAAGTATGTAGGATCTGGTCATTGGATNATGGTGATTACTATTAAGTTGGGTTAGCTTTACTGAAGGGAAGNNNNNNNNATTANTATTTTTTAGAGAAGANNNNGA[AAGAGAGAGAGAGAGAGAGAGAGAGAGAGAGAGA]NNNNGAGAGAGAAAGGGAGAGCATGAGTGGGGAAGAGCAGAGAGAGGAAGAGAGAATTTCAACTCAAGCNGGCTCCACGCTCAGTGTGGAGCCNGANGCNGGGCTTGATATCATGATGCTGAGATTACAACCTGAGCCAAAACCAAG

>B1:145474799-145475099|varpos=145474949

GCAAAATAGGTGGTGGGGATTAAGGAGGGCACTTGTGATGAGCACTGAGTGTTATNTGGNAGTATTGAATCACTATATTGTACACCTGAAACTGATCTTACACTGTATGTCCACTAACTGAAATTTAANTAGAAACTTAAAAAAAAAAA[AAACCC]CCTAAATAGTGNAGGCNNAACAAAAAGGAGGGTAAGAGAGAAGGTAAAATTATTGTGGGAAATACCCTGGTTNCTCAGGATAACCTTTGACTCAATATTCAGACTNAGTCCAAAGTAGAGCTCTCTTGAACCACATCCAGAGTCCTCAGG

>B1:145483642-145483942|varpos=145483792

TTTTAAAAGGAGGCATGGGGTGGTCAGCAACACAGTGATTGTAGAAAGTCATTCATTATGATGAATGGCTCCATCGTAAATAAGATTCACCTTTATTCAAGGCAACTGGTGCTAAATCANCAATAAAAATTGCTAACTNGATTTTTTTT[TTTAAAA]AAACACTAGCATTGATTTTGTATTTGCACCTACCATTAATATTAATTTTACTTGTCTTGGCAACTCGAGCACTAATTCAAACTGTGAATTATGAGTGGCAATGTATTATAGCAATGGAAAATACATTAAGCTTGTGGTTCTCAACCTGGA

>B1:146954745-146955045|varpos=146954895

TCTTCTTGAGTTTAAGATTTCTTCTTACATTCTAGATATTGTATTTTCTTCTGTCTGTGGCTAGCCTTTTTCATTAACTGAGTNTTTTATTGTATTGTATTCTAGTTTTTTAATATATTTAAAACNNNNNNAAATGTTTTTATTTATTT[TGAGAGAGAGAGAGAGAGAGAGAGAGAG]TGAGCATGAGTATGAGCAGAGGAGGGGCTGAGAGAGAGGGAGAGAGAGAATCCCAAGCAGACGCGGGGCTTGAACTCACAAACTGTGAGATAATAATCTGAGCTGAAATCAAGAATTGGATCCTTAACTGACTGA

>B1:147191374-147191674|varpos=147191524

GCTCAATCTTAAGAACCCTGGGATCATGACCTGAGCCAAAATCAAGAGTCAGAGGCTTAACCNACTGNGCCACCCAGGTGNCCNNNNNNNNNNNNNNGGAGACCTTTATTCTTATAGCCTAAGCCATTACTCAGTCCTCTGAGTTGATT[TTTGTTGTTGTTGTTGTTGTTGTTGTT]AATTCTCATGGCCTAGAGTATTTGTTAGCCTTAGATTTTCCATGATTTTTCTGCTAGACATTAAGCAAAAAGAAGGCAATCCTTAATGATTTACCTCCATTTGAAAAGAAAATTTCACCTATTTCTTCTCTTAACC

>B1:147581215-147581515|varpos=147581365

AACTCTGAGACGGCCCAAACATAGTATTTGGTAGACAAAGAATACANNNCAGNNNNNNNNAATACGGAGAACAATTTAAAGAATAATATGCCTGAAGAATTAAAAGCAAATATGGCCATAAGTAAACAGGGAAATAGNNNNNCAAATTA[TAAAAAAAAAAAA]TAGAAATCCTAAAACTGAAAAAAANGCTAAATTCTATAAATTNAATGAATTCTNTGAAGGTAGAGATAGAAGAGTAATGAATTTGANGCTAATAAATAGGAATNACCCAAACTGAAGAACAGCAAGAACAAAGGTTAAAGAAG

>B1:149439901-149440201|varpos=149440051

CTGTTAGANGAAATGAGAATTTTGTCATNTGACTGTAGTAATTTTCTGAAAGCAATTTGGAATGATGAGCCAAAGATGTTTGACAGTGTTCTTTGATAAAGCAATTGTGTGTGGGTGTTGTGTATATCTATATAGATCTACATATATAT[TTATATATATATATATATATATATATATAT]GAAAGGTGANGTGAGAGAAATGTGAGAGGAACATTTTATCTCTAATGTCATTCACATCAGTACATTCACCAACAAAATACTAACAACCAAATTCAAAGGCACATTAAGATCTTTCACCATGCCAAGTGGAAATCATCTC

>B1:150200888-150201188|varpos=150201038

CACTTTTTTTTTTAGTTTTTTATTTACTTACTTANNNNNNNAGTAATATCTACACCTAACATAGGGCTTGAATTCTGGATCCTGAGATCAAGAGTCCAATGGTCTTCTGACTGAGTCAGCCAGTTGCCCCTAAATTGCTATTACTTCTAAGANTTTTTTT[TTTTAAA]AATACGTCAAGTTGTTTGTATGNAAGATAGTATAATCTTTTTGTATGGAAGGTANTTTAAAAGTTTTTAGNNNNCTTCTGTATGAAGAAATAGATTATGTAGATAGATTTTATGTAAATAGAATTTGGTACAATAAGCTATTATGTATTT

>B1:150274827-150275127|varpos=150274977

AGATAGTCCACAATACTGTAATAGCGTTCTGTTGTAACAGATGGTAGCAACACTTGTGAGCACAGNGTAACTTANGGAGAAGTTGAATCACCATGTTGTACACNTGAAAGTAATGTAACATTGTGTGTCAACTATATTCAAATAAAAAA[AATTTTTTTT]TTTTTTAAACAAAAAGAAGNTTCCTAGCCCGCATTCCTAGTGGACAGAGCTGCTAGACAGAAGCCAAAAGAATTATGTGTCANTAACTTTTCTGGTCTTAATAGCTCTGCAGTGTTTTGTGGCTTCAGTTGCTTCTTTCTNTACCAGAGT

>B1:151207116-151207416|varpos=151207266

TTTTGGCTCAGGTCATGATCCCAGNGTCTTGGGATTCAGNTCCATGTTGGGCTCTGNACTGAGTGGGAAGCCTGCTTGAGATTCTCTCTCTCTCTCCTCCTGCTCCTCTCCCCTGCNCGCATGNTCTCTAATATGTTTTGTTAAAAAAA[AAACC]CACAGAAAAACAAAATCAGNATAAGTAAACCATAAATTTATTCTTAGAACTTTTATAAACCTTATTGTGAGAATTAAAATTTNNTNATANCTTTCAATCAAAATTGTTGTTTTGATTGCACTCTGTTTAGATAAAATCTATCNCCACTTG

>B1:151813528-151813828|varpos=151813678

CTAACTAATAGATNGGGGAGTCAAAATATGAACAGAAGAAGGATCAGGGCATGTGTACTCAGATGTGGNTAAATACAGGGATTTATTTACAGCAATCAGAAAAAAAANNNNNNNTAGAANCAAAATAAAANTTTAAAACATATATANTCCCTNNNNN[AAAACAAAACAAAACAAAACAAA]NCCTTGATATGTAAGCTCCNACAGATCCTCAANTATCTCAATANAAATCTTATATTGTTTATTAGCTTTATAATTTTTACCTCATAAGTCTGAATTATGTCATAAATGTCTTAGCTATTCATTTNTCTCAAGGATNTGTTGTTTCTAGAG

>B1:152085961-152086261|varpos=152086111

TTGATCCTTAAACTCCACATATAAGTGAAATCTTATGCTATTTGCTTTTCTCGGACTGACCTATTTTACTTGAGATTATANTGTTTAGATTCATCCATGTTGTCGCCAATGGCAAGATTTAATTCTTTTTAAGGTTGATTAATATTCTN[TTGTGTGTGTGTGTGTGTGTGTGTGTGTGTGT]ATGGCATATACCATATATACTATATATATTTTTCTTTATCCATTTATCTATCAATGGACACATGGGNNCTGTTTCCATNATTTGGCTATTGTAAATAATGCTACATTAAGTATAGAGGTGCATATA

>B1:152543491-152543791|varpos=152543641

GATGAACTTGGAGTTTTGAGCAGTAGTATACTTGTGATNNNNNNGTATCNAGTTTTGAGCAGTAGNATACTTGTNATGTTTTTTGTTTGAAATCTTTCCTTTTGAAAAATTATTTTGAATTAGTTTTTATCAAGGTAAAGCATGTATTA[ATTTTTTTTTT]CTTTTAAGCTAGGATATTACAAAAAGCTCCATAAGAGTGAGTCATTCTCTAACCCATTTCTTTGTACCTTCTGTTCCTCACAGGCAACTCTTTTCAATCTTTCCAGANGTTTGTTTTGTTAGTTATCTTATGTTCCAAAAAATTAC

>B1:154216706-154217006|varpos=154216856

GGAAGATGTGGAGAAAAGGGAANGCTCATGCACTGTTGGTGAAAATGTAAATTGGTNCAGTCNCTATAGAAAATAGTATTGAGGTTACTCAAGAAATTAAAAATAGAAATACTATATGATCCAGNAACTTCACTTCTGGGTATTTACCT[GAAAAAAAAAAAAAAAA]CTAATTTGAAAAGATATATACACCATTATGTTTATTGCAGCATTATTTCTGATAGCCATGATATGGAAGAAGCCCCAGGGTCTTATCGATAGATGAATGGATAAAAGATATATGGTGTTTATATCTGTATCTATATCTATCT

>B1:154217540-154217840|varpos=154217690

TTATGTTATGCTTGAAACAGAGTAATCGAATCCCTCCTCCANGATTGCATTCCATTCATATTGAAAAGGTAATGAGAAACTGTCTGTGAGATCAATTTCTAGATTTTCTATGAATAAATGCATCCTTATGATTTTATTCTACAAAAAAA[AAAATGAAT]TTAATTTTCAATANAAACACACTTTGGAAANTAAATATAATATGTTTGCCTAAAAATATTTNTATATAGCNTTGGTNGTTANNAATATTAGTTGATTGTCCTTTATCTTGGATTAGCCACTNNTAATTGACTGTGGGCTATAATTGTGAA

>B1:154524637-154524937|varpos=154524787

TGTGATAATTGCATTATGTGCATCAATTTCAAAGACTCAAGGTAAATAAACAAANAGTATGCTCTAATATGGGTACTGAGTAATTGANATAATATTGAGTTGCTGTATGCTACATCTTTGGGAACTTATAGATATTGTTATNTGGGTGT[CTTTTTTTTTTTTTT]AAAGTTTTTGCATTTTAACCCAAAGAGAAGTTGCTTATTCTGTATCTAGGCTCTAAGAGAAGTTTCAAGTCAGCACTTTTCCTTTCTGCTCAATTTAAAAATCCAAAATTCCTTCTGAAAAATCCACCATGTTTATTTGCCTAT

>B1:154570673-154570973|varpos=154570823

GGAAGAAAAGAGGAAGCTTGAAGTATTAACAAAGTTATCTTCACCAGAAAACTCCTGAGAGCTCANTATTGCTGATGTTTCCCACAAGTATATGTTCCCCATCCTAATNTAGACATCAAAGCTTTGACTCNAAATATCCAGGAACATCT[GTTTTTTTTTTTTT]TTTTTTTCAGATATCTATATGCAATAAAGGATCTTTAGTAAAGGACTATATAAAGACAATAAAAATATTCACTAAAAAGGAAGGAGTTTAAACACAGTTACTGAAATGTAAAGTTCACTGTATAAATAGATAAAATTAATAACAATGATTA

>B1:154857477-154857777|varpos=154857627

TGGAACCCAAAGAATTTTTGTAGTTAACTCAAAANAATGATCATAGAAAGGAAAGGAGGAAAAACAAAATAAAAAGTTTTAAATATGGAGTAGTAATTACCAAGATGCTATATCTAAACACAATCATANCAATTACTGCATTAAAAAAA[AAAAAACAACAA]GACCCAACTACATTCTGTTATCAATAAATGCATTCAAAATAGGAAATAAAGAGGTGTGGAAAGAAAAAATATGNNNNNNNNTATAGCATCCAGTTTGTTTACCTAAGTTAATACCAAAGTAAACTTCAAGACAAAGGAATATTCTCAG

>B1:156375658-156375958|varpos=156375808

TTTGTGACTTCTTCAGGAATCACAGCTTTGGTGCTAAGAACTCAAATTTATTTATAAGTTATTATTTTTGAAAATNNNNNGAGTACTCAAAGTATTAAGAATATGAAAAATCTATAATACCTACCAATCCACAATTCATTTTCTTTTTT[TTTTTCTGTTTCT]NNNTNTTTCTTTTTTTCTTTTTTTTAAGTGGTAGAACGCTCATAATCTCCACCCTGCCAGCCAAGTCTACATTTCAGTATGGGCCTACATTGTGTATTNGTTTTTGAGATGTTTCAAAGCAAAGATACTTTGGATTATTCCAGTATCA

>B1:157029155-157029455|varpos=157029305

TTTGACTTTTCATTTTCACATGNCCAATTGCTGCTGGTCTCAAAATACATGAGTATTGAGTANTGAGTATTTGTCTCTATCAAACTNNAACAGTCCTTTAGGATTCACCGTCCCNNTTTTAACTTCTNTTTGCCAGATTTACTNAATTT[CTTTTTTTTTTTTT]AAGTTTATTTATTTATTTTGAGAGAAACAGAGGACAAGCAGGGAGAGACAGAGTAAGAANNNNNNNNNNNNNNNCAATCCCAAGCAGGCTCTGTGTGGTTAGTGCCACAGAGCCCTNNGTGGGGCTCAAACTCATGAAAACCAA

>B1:158436475-158436775|varpos=158436625

ATGTATTGAGTTGCTTGCCCTTTGATTGTCAATTTGTAAGAGCTGAGAGCTCTTTGTGTAGACTAAGAATCTACACTTTTACCATTTGAATATTTTTTNNNNNGTATTGAAGAATGTGTGGATATATAATTTCTCCAAACAGGCATGCTTATACN[GTTTTTTTTTTTT]TTTTTNATGGTTTGTAGGGTTCTACTTTTGGTAAGTAATATTTTCCATGCCTCCAAACTGTACAGGTATTGATGTAGATTTTTCTGCAATTATACATTTGGTTTTTTACCTACATGTCTTTATCATCTAAAAAATCTTTCTTCTGTACTG

>B1:158523953-158524253|varpos=158524103

AAAGAATAAAACTAANTGGTTAATGATAAGAAAAATCAAAATTGCATGAAAAGACATTGNTTGNATTATATTCAGAATGTCTTCTTTTAAGTATAGAAAGAATCAAGCATNCAACTTTTGTTTTTAGAAAGAGAATTAGCTATGNNNNN[TTAAAAAAAAA]AAAAAAATGTCTTTCTATCCATCTAAATGAAAGTGCTGTCAGCAACACTGAATTAAAATTCAGAATAAACACGAACATATNTCAACTCAAAAAAAAAANTCTATATACCATTTTTTACCTCCTGGTAGTAGCAATAAGCTTGAATAAAAT

>B1:158810797-158811097|varpos=158810947

CTGACATGCACTTTCAATTTGGAAANTTGTGGTAAAAGATGTAAATATCATGAGGGAAGGAGTACATGAGTGACAGAATGGAAGGTTATGTGCTCTTCAGACAATAATTGCTCNTAGTATAATTATCCCTCATTCACTTTGTAATGCAG[AACACACACACACTCACACACACACACACAC]NNNNNNNNACANACACACACACACANACAATGTGCTTTATGAGCTTGTTCTTAGAATTTCTATTTAAAAGAGCACTGCNATCAAAACCATTTGACCAACCTNTTTTTAAAGAATACCATTTGTNGATTTTCATGNTGCTTACAGGG

>B1:162431413-162431713|varpos=162431563

TAAATAACNATGGCATTGAAAACTAAGGAGGTGGATTATAGCNTAGCACTTTCGAATAGGAGTCTATTGATTGACAAAGTACTTCATGGTCTTAATATATTCCCACTGAATTCCTTTTCCTCCGTCTAGGTCAAAGAATAAATAANNNN[GTTTTTTTT]NTTTNTTTTTCATTTANCATAAGACAAGTTATATAAACTATGAANCAAAAGATAGGGAAAAATATAGANANAGTTCTTTTTAAATCATGAAGTAGACACTTCTGTTTTCTTTGGCTGATCATTTTCTTTTATATAAAATAAAATGATAAA

>B1:163860438-163860738|varpos=163860588

GCGCAGGCAAAGGCTTCAGGGTGAGGAGTAGACAACAGACTAGGGGAGGAGACCTTGGCNAGAGATGAGGGTGGANAGAGAGGTNGGGCCCCCTNNNNNNNNNNNNNNNTTCTCNNTCTCTNTCTCTCTCTCTCTCNNACACACACACA[CACACACACATACACACACA]CACACACACTAGTTATTTGCACATGTCTACATTTTACAAGAGCCATTATTTTCCCTTTAAACTTAAAAATTAAGAACAATTCTTCTTGGAAAAAAGATAACTTGCATTCCTCTTACTCGTCTTGAACCTGGTTGAGTTGATTACGAG

>B1:163941567-163941867|varpos=163941717

GTGCAATGCTTTGTTTAAAGAGCAGCNACCTGAATACAGCTGTGAAAAATCTAGAGTTACATACANCTTCATTGACTATTTTTAGATTCACTAAGCAATATTTGCTTTCTCTGTCTNGAATGNATNTGNAATATACATATATATGTATA[GGGGTGTGTGTATGTGTGTG]TGTGTGTGTGTGTGTGTGTGTNTTCCAGAAACANAGTGAATATACTTAATGCTCTACCCAGAAGGGCCCTGTTGTTACAAATATCTTCAATCTGAAAAAAAGGAAAATGGTAATATCTGTTTCCAGACTCTCTGGACCCTGCAAATACC

>B1:164858128-164858428|varpos=164858278

ATTCATATCTGTTTTCTTATTCTACTGTCCTGGATCTGACTTTCCTCATCATTAGTCACTTTAAAGGGCCAAGNTCCGGGTCTGAAGTCCTANGATCTGATTTATTCCCTTAATTTGCCAAGTTAATCTNTTTTCATTTAATGATATTC[ATTTTTTTTTTTTT]CTCTAGGAAGCAGAAACCTATTTAAGACAGAAGAGGGGGAGAATACCATTCCTAAAATTTGGGGGGGTANCTTTTTATTTTGCTGTAGTTTCAACTTACAGAAAAGTTGGAAGAATAGTACAAGGGATTCTCAAGTAACCTTTATT

>B1:167163072-167163372|varpos=167163222

TTTCAGATGCTTCAGTTATTCTGAGCTACAAAAATTCCAGTATCAAATTCTTTCCTGTTACTATTTTCTGTTNCCCTGATTGGTACCAAGAATATGGGTNTCTTTATCATTTAAAAATTGTTTTCTAATTCCTACTTTTTATTTCTTCA[GTTTTTTTTTTTTT]ATTTCAATTATGCCANCTTCTAAACGGTCTATGTTTACTTTGTTTNGCTCTTCCATGCTTATGNTTTTTCCTTCAAATACTGNGGATAGTTTGCNGGTTGCTTACTGATCCAATGGATACCTCTGTNAACATGATTTTTTGA

>B1:168249453-168249753|varpos=168249603

ATCATTTTAAAGATTTTCTTAGGACNCCTATAAGATGAAATACACTTTTTGAGTTTCAGTCTCTGAACCCATGAATAAATANTTCAGCTTTCTCTCAATGTTTTTACTATAGAAACATGNCTGTAAAATGAGTCCTTTGTACTAAAAAA[AAACCCA]ATCTAAACAAAAGNAAATTTAGTATCTTTTTAAAATGTAGATTTAATGTACTTCATTATGCTGCAACTACTTTTAACCACAACCAAATTAATTCCATTTGTAAGATACTATAATTGCAATAAATTGTGCTTTAAATAGCAGTTCAAAATT

>B1:168390064-168390364|varpos=168390214

CATCTTGTACATACCCNAAGATACTGAAGAGGTTTCTCTTTTGGCCNTTGACTTTTACAGNAACCGCTCTCATTTCATTTCTGGAGTCACTACCTAGAATTAACCTGGTAGCGACTTGTTATTCCATCAAACAGACACAGGAAAAGAAA[CAGAGAGAGAGAGAGAGAGAGAGAGAGAGAGAG]GAGAGAGAGANNNNNNNNAGAGAGAGAAGAGAAAGGAAAAGAGAAGGGNNNNNNNNNNNNNNGAGGGAGGNGAGGCNGGGCGGGGCGAGGNNAGAGACTGATTCATTNCAAGACAAATTTTGTATAANAAGTTGAAATTAAAAACAT

>B1:168526161-168526461|varpos=168526311

GGAAAATAATACTAAAAGCATAATTAAATGATACATGTACCTGCTTATGGCNTAGAAGAACAGGATTTATATTCTGATTCTAGAATTTGTAACTTGTGTGAACTTCGGTAGTTTATTTAAACTCTATGTGCCTTAGTTTCCTTATACAT[ATTTTTTTTT]TTTTTATNTTTGTTTATTTTTGAGAGAGNNAGACAGAGTGCGACTGGGGGNNNNAGGGGCAGAGAGAGAGGAAGACAGAATCTGAAGCAGGCTCCAGGCTCGNACTCACGGACCATGAGATTATGACNTGAGCTAAAGTCAGCCGCTTAACTGA

>B1:168884565-168884865|varpos=168884715

AAATAAAGCTCTTTGGAATGAATGCATTGNCATTATTAAATATGTGTACATATTTAATTCTAGCTTTCTTTATGTGAAAGGAAAATTGAATGAATATTACAGCCCAAGGCAACTGGAAGATGGATTTGTCATTAAAGTGAGGAAAAAAA[AAAAAATT]TAGAGCATAGGAATAAAACTTTGATTTTGTAAGGGGTATGGAGGACTTTAATTGCAGGTTGTGATGAAAATAATAGCTCTCAGCTCACATTAGTTCTAAGCGCAGTGTTATAGGACACATAATNAGCACATATGTACATACACAATTTTG

>B1:169276207-169276507|varpos=169276357

AGGTCATGATCTCACNGTCCATGAGTTCTAGCCCNCGTCAGNCTCTGTGCTGACANCTCAGAGCCTGGAGCCCGCTTCAGATTCTGTGTCTTCTTTGTCTGCCCCTCTGTCACTTGCACTCTGTCTCTTGCATTGTCTCAAAATTAATT[TAAAAAAAAAAA]AAAAAAAACATTAAAAGAATGAAATAAAATTATATACAGTATTTTTGATGCTAAGGCAAGACAAAACANGGTTGGAGCAAAGGGGCAGGAAGTTGGGAGGAAAAGACTNAGGGAAAGTATTCTGTCCTCTTACTCCTAGGAAGGGTAATA

>B1:169514469-169514769|varpos=169514619

TGGTTCACGGGTCCAGGCCTCGCGTCCGGCTCTGTGCTGACAGCTCGGAGCCTGGAGCCTNTTTCAGATTCTGTGTCTNCCCCTCTCTCTCAGCCTCTCCTCCACTCATGCTCTNNNNNNNNNNNNNNNNNAAAAACAAATAAAAACAT[TAAAAAAAAAAAA]NNGATAAANAAGATAAGGGGAAAATGTCTACTGTCCTTAATGCGACATCTTTATAGCTNCCTAGGTGCCTGAAACATCAAAAAGATAACTATTCATTAATTCATTCATTTAACAAATATCCATTAAATGCTTCCTATGNGCCAGG

>B1:171335882-171336182|varpos=171336032

ATCTGAAGCAGGCTCTGCAGACAGCAGACAGCCCGATGTGGGACTCAAATTCATGAACCATGAGATCATGATCTGAGCTGAAGGCAGATGCTTAACCNAGTCACCCTGGTGCCCCAAAACTTCCCCATTTGTAAATCTGACAGAAAAAA[AAAAATAAT]TTACAGAGCTTATTTTTTCCATGTGAGCTAAGATGAACTAGATGTGGCAAANTGCTTGGAGAAGTGGGGGTGGAGAGCAGGCAAGGGAGGAACCCTCAGGGGNAATAGAAAGATAGAAAAAGGGATTGGGGGGCACTGGCTGGCTCAG

>B1:172237883-172238183|varpos=172238033

ATGATCTCATGGTTCNTGAGTTCNAGCCCCACATTGGGCTCTGTGGTGACAGTGCAGAGCCTGCTTGGGATCCTCTCTCTCCCTCTCTGCCCCTCCCCTGCTTGGCTTCTTTCTCTCTCAAAATAAATAAATAAACTTAAAAAAAAAAA[AAAGAAAATTAACTCAA]AAAACAGATCACAGACCTAAANGTAAAATACAAAAGTGTAAAACTACTAGAAAATAACATGGGAAAATCTAGATTAAACTTGGTATGGCTATGACTTTTTACATGTTAACATCAAAGCTACAATCCATAAGAGAAATAANTGATAAGTAGGA

>B1:172505678-172505978|varpos=172505828

TGTTCTATTGCAGATTTCAGTGATTATATGAAAGATGACCTAAGCTGTGATAAAATCTTGGTTGTATCAGCATTTTAATGAGAAATAGAACAGCTTAGAAGACAACAGACACGTACATTGGCAACTCCCTTCCAAGCAAGTTTTCTGGA[GATACACACACACACACACACACACACACAC]CACACACACACACACACACACACACACGTGTTGTCATGCCCCAAGGTGACCTTNGTATTCCCCAGTGTCTCTTTCACCTTTGCTGCTATTTNCATAGGCTATCAAGGTATCCAGGGTTATCTTTGCTCAGGACTGAAGACAGCCTCTCC

>B1:173019485-173019785|varpos=173019635

GGTTGCTCAGTTGGTTAAGTCCAACTCTTGGTTTCAGTTCAAGTCATGATCTCAAGGTAGAGCTCCNTGTCAGGCTCCACGCTGACAGTATGAAGCTTGCTTGGGATTTCTNTTTCCCTCTCTCTCTCTCTGCCCCTCTCCCACACTTG[TTCTGTCTCTCTCTCTCTCTCTCTCTCTCTCTCTCTCTCTCTCT]TTCGCTCTCTCAAAATAAATAAATAAACTTAAANCAAACAAACAGTCAGCTTCACATCCTAATTCACAGAGAACACAGAAGCCACCAGATGGAAACTCTCTTAACTT

>B1:174780797-174781097|varpos=174780947

TCCAGGCTCTGAGCTGTCAGCACAGAGCCTGACACAGGGCTCAAAGTCACAACCTCTANATCATGACCTGAGCCAAGTCAGATGCTTGACCGACAGAGNCACCCAGGTGCCCCAGGATCTCATGCTTTTTTATGACTGAATAATATTCT[AGTGTGTGTGTGTGTGTGTGTGTGTGT]NTGTGTNTGTGTGTGTGTGTGTGTGTGTGTGTGTGNNNGTNTAAATGGATTATACACATTATATAAATGGAATCCATCTATTCATGGATTCTTGGGTTGCTTTTCTATCTTGACTATTNTAAATAATGCTGCAATAAACATCGGGGTGCA

>B1:174934785-174935085|varpos=174934935

TACTATCTANCTTTCTACTTAGTTNNTATTAAAATAAATAAGTGTAAATATTTTACTAACCAAGTATGTGCATCATTGNTAATNTCTCCCCATATTGGCTACCCTTCAATACCCTAACACTAAAAGTTTTATGTTTAACTGTGAGTTAATATGCA[GAAAAAAAAAA]AAAAAAAAAAATCTTCCNTGGAAAGAGGCCTATGCAAGCGAAAGCTGCCAAAGCTTAAGGTTCATTTAACTCCATGTTAAATCTCCCTTCATCTCTGTGGGTTCATATTTTATATACNNGGGTTCATTTTGTACTTTAAAAAATCTTTGT

>B1:176061118-176061418|varpos=176061268

TCATTNGTACATTNCNTCCACTTNTAAACACCTCATCCTTTGNAGACTTTGATGCCAAATATGCCATAATCCAACGTTCTTGACCCATGAATGTCATATCTGATAAATGGCTTCTCAGATTTTTTTNNNNNNCACCTTTTCATTCCCAAACTCTAC[ATTTTTTTTTTTTT]CCTNATGTGTCTTGGNTATTTACTTTCCTTGCTGCTTTCATAGTCCAANNTACCATCAACTTTNCCTAGATNGTCTTAAAANTTGGTATTCCAATTTTATTCTCCTCCNAACCATTNTCCAAATTGCAGACANAAGATTT

>B1:176655214-176655514|varpos=176655364

TGATTTCAGCTCAGGTCATGATCTCGNGGTTTGTGAGTTCAAGCCCCTCATCNGGTCTGTGCTGGAAGNGTTGAGCCTACTTGGGATTCTCTGNCTCTCTGCCCCTCCCCTNCTTGTNCTCTCTCCTGTTTTCAGAATAAATNNNNNNN[AAATTTTT]TTTAAATAAAAAGCAATAAACAAAAACATTATTTAAAAAAAGTCACCTTCCTTTTGTGTTGTAAAGTCTCATTAAAATTCAATTTATAAATCACACATAAGGACTCTTTNGTAAAGAAAGAACTTATTTCTCCCCAACAGTCTGGCTAAGCTT

>B1:176935036-176935336|varpos=176935186

TTCTNCAGGAGCACACTAAGANAAGTTCAGGTGAGCAAACAGTGAGATGGAGACTGCTTACAAAATCAAGAGAAGAGGCTTCAGAATGAAACCCAGNTTGCCAGCCCCTTGATCTTGGACATTCCANCCTCCAGGACNGTAAGAAAAAA[AAAATTGTTT]TTTGTTTAAATCCCGCTGTCTATGGTANTCTGTATGATAGCTTGAGCTGACTAAAANAGTNTGTCATGTTTTATACTAGGAATTCCAAATGATATGNTCTTTGCTTGAAGTTCAAAACTCAAGTTCTTTTGGTAATTGAGGATCTGCTTT

>B1:177005454-177005754|varpos=177005604

ATGNNNNNNNAGTTCATGACTTCAGGCCACACGATGGGCTCTGTGCTGATAGCTCAGAGCNTGGANTCTGCTTCGGATTCTGTGTCTCCCTCTCTCTCTGCCCTTCCCTGCTCACACTCTGNCTCTCTCTCTCAAGAATAAATAAACAT[TAAAAAAAAAAAAA]NNNCTGAAGAGGAAACACAGATATATTAGCATGTGCCACATTGCCATACCACTATGAAATGTAGCTGCTNTTATTTGAACTTAATTTCCATTGTGGTCCTGACATGCAACCACACTAGTAAACCCATGTTTAACAAGATATTCACA

>B1:177282515-177282815|varpos=177282665

GAAATAAACAAAGTAAAGAACAGATAAACAATAGAGAAAATGAACAAAACCAAAGCTTGGATCCAAAAACCAAAACNTGGGTCTTCAAAANTAATAACACAGTTATTATTTACCTTCATTATTAACTTTATTTAGATGNACTAAAAAAA[AAAAACCAGA]GAGATGACTTAAATTACTATAATCAGAAATAAAAGCAGAGACATAACTACTGANTTTATAGAAATAAAAGGATTATAAAAGGNTTATAATAAACAATTGTATACCAAGAAATTNGATAACTTAGATGAAATGGACAAACTCCTAGAAACAC

>B1:177320436-177320736|varpos=177320586

TGGTTTCAGCTCAGATNACGGTCTCATGGTTCATGGAATCAAGTCCTGCATCNGGCTCTGTGCTGNCAATGCAGANCCNNNTTGGGAATCTCTCTCTCCTTCTCTNCCTGTCCCTNCCTCACTCTCTCCCTCAAGATAAAAATAAACAT[TAAAAAAAAAAAAAAAAAAA]AAAGAAATCTTATGAGAAACATATCAGAAGGTACAGCTTGAAATCCTAGTGTTTAATATATATGGNTCTAATCCTGTAAGTATTAAGGACACACCCATGCTATGGGCATTTTTAAAGCAACAGTTTTACTATAACTGTTTACTATA

>B1:177653463-177653763|varpos=177653613

TTTATAAGCAGACTATGAACNGACTGCTAACTTTCTCAAAATCTTACTGCAGGATCAGTTCTACTAATTTAAACCTTAAGGTAGGANATGATCATATAGANAAATGGGNTTCTCAGAGTTTGCATATAGAGAATTTAANNNNNNNNNNN[AGAAGGAAGGAAGGAAGGAAGGAAGGAAGGAAGGAAGGAAGGAA]AAGGAAGGNACATAAGGAAAGGAANNNNNNNNNNGAAGGAACAGTAAGAAGGGAGGGAAAGAGAGGGAAGGAAGAAAGGAAAGGAAGGAAAGGAAGAAGGTAAGGAAGGAAAGAAAAAGAGAGGTAAGAAGAGGAGA

>B1:177718508-177718808|varpos=177718658

GGTGNGATGTAGGGCCAAGTAGCTTTAAAAACTGTAAAACAATAGATATAATCCTTAAAANTTCACAATAATNCAAATTTGGCTTTTCAAAGGTTTGCATATGTCATCTGTAGACTGTCAGCAGTAACCAGTGAGTTTGTGTTTGTATG[TTGTGTGTGTGTGTGTGTGTGTGTGTGT]NNNNNNNNGTGTGTGTGTGTGTGTGTGTGTGCCATAGCCAGTGTAACCCAAACCTTTGCTTCTTTTCCTTTTTTGTTTTGTTTTTGTAATGTTATTCTTCATGTCTGCAAAAGCTTGAGATTTGGGAAATGGTGGTGAATTGATT

>B1:179607553-179607853|varpos=179607703

CTCTCNGTCTCTCTCTGCCCCTTCCCCGCTTGCACTCTCCTTCTNTCTNTCNNNNNNNNNNNNNNNNCATTAAAAATGTTTTTATATTCTAGCCATGGCAGATATTTAAACTTCCATTAGAGAAANATAATTACAGTTGCTTTATTCCT[TAAAAAAAAAAAA]NNNNNTAACATTTTTAAAAGATTACATTTATGGAGTTTCNGGGAATGATTGCCACATATTTCTACGTGAAAACACAGCAATAAGGGTGAAAAAAGTAAAAGTCAATAGTGTAAAAGAGAAAAGGAATTGAAAANATGAGATCACATT

>B1:179647489-179647789|varpos=179647639

TCTTCTTACTCTTTCCACAATATAGTGCCTGGAGTGATGTATCTTCTTTCTTTCAATGTACACTCTCTCCTGAAATTTCTTTACCAGACTAAATAAGAAGAGCTCATGATCATGAGCCTGGACTGTCTATAAGAGGAAAGAAAAANAAA[AAGGGG]GGAGATAAACCGTATCTCCATTAATTTCCCACTTCTGTCCTTCAGTCTTTCCTGAGACCTNTTGCCTCCCAAGAATACCAGTATAGCTCTTCGGCATGTCCAATTTATGTTGTATGTTTATCTACACAGGTATTTGACAAATACTTTTAT

>B1:180503766-180504066|varpos=180503916

AGAAGTCCTTCCTAGCACCTTAGAAAATTTATGTNTAATTCTATATATGCTTTGATTTGAAACATTCTTTAAGATTTTATTTTTTACAAAGTGTTTTATTTCTATTTATCACCCAATAAATTTATTTAGCAGCATCGAAATTTANNNNN[ATATTTTTTTT]TTTTTTGTTTTTTCTTAAATNGTATCTATCTTGAAATTCACTCNTAAAAAATGTACTTTTCTGCATTCTTAACAGTAATTGTATTAGCATTATTTAGGTTTCATAACTTTTCATTTTTTCTTTTGAAAATAAATCATGTTGTCTTGACA

>B1:181013167-181013467|varpos=181013317

ATATACTGAAATATAATTAACTTGTAGTATTTGAANACCTCTCCATGGATACTCANCAGCTAAATGAAGACATGCCCATGGCTGAGGTATAGCGTCTGGATCTTGGCGGGTGTGAGGCTGCCTTTTATTTATTTGTATTTCAATTTTTT[TTTTTTAA]ATTTTGGAATAACTTTAGATTTACAGGAGAGTNGAAATACAAGGAATTTCTGCAAAGCCCTTACCCANTTTCTNCCACTGTTAATATCACTCTAGTATTTTTGTCAAAACTAGGAGACTGACATTGGTATAGAAAGAAGAANTGGACTAA

>B1:181212220-181212520|varpos=181212370

TAGAATCTGCTCTGACTTTGTAACTGTGCATTTGGGATGGGTCAAAAATACTATTTGTTAAAATAGAACCTTCATTTATTTTGTAAAATTCTGAAATGTTTCTCACTTTGAGATTTTTCNTGTTTNTAGCTGTCTCTATTGACCTTGTT[TAAAAAAAAAAAAAA]AAAAAAATTCTACCAAGTAAATTCGAAGATCCATGNGTTAAGTGATCCATGATTGGGGNGGCATCCTGTCTAGCNAATAGAGAGGNGCTCCTAAGAGTTGAACAAAATGGAAGGCTTTCATAGGAAGGAAGGTAAGGTAAGGGANTTATT

>B1:181907745-181908045|varpos=181907895

ATTTTCCTGGAAGTACAGATGTTAGACTAAACACCTCACTCAGGGTGCAGGCAGAAAGGGGGTGGGGCAGGCTGATCTACATGGTTTTGCACTTTCTTTACTTTTCATGTGACTTCTATACTCCTATCCCTATTTATTCTTAGCTGGGA[CTTTTTTTTT]TTTTTTATGAATTTAGACATAGCACTTGTGACTTGTATAGATGTAAACTGCCAGGTCCAATNTTTGTAGAAAGAGTATCANGTTGNCTAAAACACAACTCAGGTTTTTGTCTGTTCCTTGTTCATTTTTGTGGGTATAATGCATACCCC

>B1:182306275-182306575|varpos=182306425

TNCTTTAATTTGTCAAGGTGCAATCAAACTTAACTCAGAGTTTTGTTTGTAGTAGAATATGATATAATGTTTAAGAAAATGCATANGACCATGCATACACCCAATAAGTCTATTTTCAACAGATCTGAGAATCTNTNTAATATTTTAAA[AAAAAAAACAA]TGTGGTTTTAGAGTTACTGTATTACACAAAAATCAGCATACTATTATTTATATTATTTATAAAAAGAATGTATGAATAGNTCAGAAGTGNAGATTCTCNTTGGACAAATAGTATNAGAGCCAATCTCNTGCATTTTGGAGGTAAGGTG

>B1:182327985-182328285|varpos=182328135

CAGGTTTTAATCAGTAGGTGAAGGAAGGCTTCATAAAGGAGGTCAAGCCTGGTTTGAATCTTTAAATGAAAGTAANAGTTCANCAGGGGTTCCAGGCAAAAGNATGAAAACTGTAAAATTTGTGGACATGAAAGNGTTTGGCTCATTAG[GAAAAAAAAAAAAA]TTACATTTAAAGCNTTCCTGGAAAACATATTTTAGCCTTGACATATGACTTGAGATTAGAGTTGTCCAAAACAGGACNCCTATTAGGCAAAGCNCTTTGTACCACAGGAAGGGATTNCGACTTTCTTCCNTGTGCTGTGAGG

>B1:182415644-182415944|varpos=182415794

TTTTAAAAATCACCTGTAGACATCCATTAGCCAATATTTTGAGAGAAACAAGCCNGAGGAGACAGTTCTATCAATACTAAAGGGACTGCTTTTAAAAGTGACCTTGCTTATTGAGTACTCAGCACTGTAAAAGAGTAGCATTTTATACA[TACACACACACACACACAC]NNNNNNTCACTCACATACACATCCGCTGGAAAATGCTGCGCGGGGAAGAAAGCTCTAGAGAACGCTAACANGACGGTTAGCAGAAATGAAACTTGTTAAAACGTGACCTAGACCAACCTGCCATCNTATCAGTGGAGCAAAATC

>B1:182495987-182496287|varpos=182496137

GAGATGATGGATTTCAGAAGGCAATGAACATAGCCTATGCCACTTGATTAAGAGTAACTGCAANAAAAATATGTTATTCCTCAGGTGAGATTTTTGACTAGAGACAACAAAACAGTGCTGGCATAGAATATTTACGTACNNNNNNNNNN[ATATATTTTTTTTTT]CAGTAGTGGAGACGATGAGTTTCAAATNCGCAACATTANGAAAAGTGTAAAACAGAANGCAAAGGTGAAGAACTTAAGANTTATTCAACTTGTTGTCATATTAAATTGCAAAAGTGTGACAGATATAGAAATCATCTTTTATGT

>B1:182603315-182603615|varpos=182603465

ATTTCAACAGTATTAAGCCTTCCAATCTATAAATNTGGATGTTTGTCACTTATTTGTGTCTTCCTTAATNCTTTTCATCAACAGTTTGTAATTTTCAAGACATAGTTTTGTACTTTTCATGTCCATAGTTGTTTGTTACTCAGTATTTT[ATTTTTT]CTTTTTGGTGCTATCATAAATGGAATNATTTTCNTGATTTCCTTTCAAATTGTTTGTTGTNGGNGCACAGAATACAACTGATAATTGAATGTTGATTTTGTATTCTGNATCTTTCATTTTCATTTATTAGTGCTAACAGTTTTTCTAT

>B1:182986024-182986324|varpos=182986174

TTCTATGCTTCTTTAAAAAAANNNNNGAGAACTCTCATTTTATTTAGAGGACNGTCTATCTTAATAACATAGCGCTGTAGGCTACCTTNTGTGTCTTCTGAAGTTCAAGCCAATGAGAAGGAAGAGAAAGTCAACAACATCNTTCACACACACACA[TACACACACACACACACACACACAC]TCAAACTCAGNTGACCCATACTTTAGCCATTCATACTGTATATTTCCCTCATCTTCTTATTTAGAACTNNGNCACAAAGCCTTTTTATATAGGAGCCATCTTGAAACTCTGAGGACAAAATCCATTTGTGAAGATG

>B1:183034159-183034459|varpos=183034309

TTACAGACTCTAAAAGTAGTCTAGAGTTGGAATTCAAATACCTAGACTCATTTGTNTTTTGAAATATGTAAAGAAATATCACCTATGACTAGATCCTTCCAGGTTTTTTTTNNNNNNAATAGATGTNAAGGATGATATACCTTGCCNGCATTAATAAACA[GACAGAAAAAAAAA]AAAAANTTTTTCATTCAATTATTTGAACTACCTCTGTATTATACCCAAAAGTTACATGGTGACCTATCATCAAAAATTATTTNTAATGAAGTCTCCACTGACAAATCAATTTGATCTTCCATCAATATTGTTCAAATCAGAAATTGAAA

>B1:184811875-184812175|varpos=184812025

TACCTATCCTTCTGTGACTGGCTTATTTCACTTAACATAATGTCCCCAAGGTTCATCCATGTTGTACCATGTGTCAGNAATCTCCTTCCTTNTTAAGGCTAAATAATATTTCATTACATGTACCANNNNNNNNNNNNNGTNNNNNNNNNNGTGTGT[GTGTGTGTGTGTGTATGT]ATGTATATTTACANACATATATATACACATATTCACCAAATTATTTTGTTCATTCATCTGTCAACAAACCCAGGGGTTGTTTCTAACCTTTTGGTTATCATGAATAATGCTGCTATGAACATGGGTNTACAAANATCCCNGAATCTT

>B1:186081454-186081754|varpos=186081604

ACTGGCTGCTGAGTGATTCATATGGTGCTGGGGGAGATGTGTTCAAATGAAATGTGTTGCCACTATGTAAAATAGCTTATTACTTCCAGGATATTCCAACCTATTGATGCAATCCTGCAGAACAATGCAAATTCTGGTGTGTGNGTGTG[TGTGTGTGTGTGTGCGCGTG]TNNNTGTGTGTTTGTGTGGTGTTGGGGTGAGGAGAAGACAGGAGCACANGCACAAGTGCGCATTTCTAGGGCATAGAGCATGCCTTCACATTTCCCTCCTCTGACCTTTATGCCAGAAACATAAGAATAACCCATTGTCTGCTGGCCCT

>B1:186339403-186339703|varpos=186339553

TAGATACAAAAGTATAAAAGAGAAAGAGAGNNNNTTGTTTGAGAGTGAGTATTCAAAATATTAACCTCTCCAGCAGAGGCTCAGACCCCTCAGAGCAGATGCANACAACAAACCATCAGTGANGTGGGATCTGTCCTGATTAAAATGCTTTAAAG[GAAAAAAAAAAAAAAAA]NNNNGCCAATAACCTCAAAGTCTAANTCAGTAGTCTAAGTCTGAATTGTTATTTTTTNNNNNATTTCATTTCAAAAAAGAAGAAATTATTATTTTTTAAATTTTAGTTCAGCTTCTTGCAAATCTTGAATCAAACTTTANACATTAAAACCA

>B1:187262145-187262445|varpos=187262295

TAGTGCTTCGTGTTATTTTATGTCTAAGATTGTGGAAGAAACCTAAGCTCAGAAGAGAGAAGCTGTCCTGCTTTTCTGGATAATAACTAAATTCCTATGTAGAATTGTATTGCACTCAAAGTTTGTTCAGAAGTAGGGTCCTTTTTTNN[CTGTTTTTTTTT]TTTTTAACTTCATATCCTCTGATTAAAAAATAATTTAGGCTTTTCAGGGTGCCTGGGTGGCTCAGTTGGTTAAACAANTGACTTCAGCTCAGGTCAGGATCTCACAGTTCANNNNTTTGAGCCCCATGTCAGACTCCATGCTGACAGCT

>B1:188322470-188322770|varpos=188322620

GGCTCAAGCTCACAACCCTAAGCTCAAGAGAGTCGCACNCTCTATGGACTAAACCAGCCAGACNTTCCTATACAGGACATTTTCCTACAACCACCTTAAAGACTAAGTAGATATGGAGACAATTTTCAATTTTATTTCNNTTTTTTTTT[TTCCCC]CCCAACATTGCATTTGACTGTCCTGATCTTTCTTGACATCTCAATGAGTGCGAAATAATGCGATTCTGGTAAAATCCACGGTGAGACTAGCCAAGGTAATTACATTGATGTTGTCACAGTATAATTTGCTGTACATTTGTATACATTTCC

>B1:193624729-193625029|varpos=193624879

GACTCACATTACAGGATGAGTCACCATGAAAGATAAAGGCTGAAGCGNCAAGAGGAGGTGACCTGAGCCTCTGATTGTGCTCCCAACAAGCGTCAGGTAGGAGGGCCCTTGTGTGGGAAAGCATTTCTCTCTTTCTTTCTTNNNNNNNN[AATTTTTTTTTTTT]TTTTTTTTTACAAGCTCTTGTTACACGGTTCAGTTATTTCAAGTTTTCTGATGGTTTGGGGACCAAAATGGGCTATTCTAAAAGACACAGTGAGAGCAGTTACTAACTAGGACGTACGCAGGCAGTGAAATTCTGATACGCCTCTGGGTC

>B1:194543676-194543976|varpos=194543826

TAAANCAATCAATGGGGTGAAAAATCAGAATAGTGGTGACATTTCGGGAATAGAATAAAGTTAATGATTGGAACAAGGCAGAAGGCAGTTTCTGGACATGTTAGTACTATTCTATTTATTGACTTGTATGACGTCAAATGGATATGTTC[ATTTTTTTTTTTT]GGGTACTTTATGGAGCTCTGATTTGTGGATGTTTCTCAATGCATGTTATTTCTCAATAAGAGTTATTTCAAAAGGNTTCAAAACTTAAATATTCAAATTAACAAAAGGAAGAAAACCACCCATTACACATTCCTATTTTATTAT

>B1:194563415-194563715|varpos=194563565

TAAATNANGAAAATATTTCAAAACTTTGATAAGGATNGAATCTGGACAATGCGAGGGGTGGAATTTATTACACTGATNTTTTATTTTTGTCTACCCTTAAACTTTTCACGTTGATCTTTTGAATAGTACACCATGATAAAATCATGAAA[AAAAAAAAATAAAA]AAANAAAACTAGGGCACGTGCCCAACTCNCCTTCCAGTTAAAACAAATATATATTAAACAAGCACTCTAAGTAGGAGCTATGAATANNNNNNNNTAGCAAAATGCTAGAAGCCATTAACTTCTCAGATTCTCCCTGAGGTAACTTCTAG

>B1:195034760-195035060|varpos=195034910

CAAGTACCCAACCTAGTGTTTTGCTTTTACTGTTCTCTTTGCCTGGAATGCTCTTCCCTCCAATATTCAAGTAATGAATTCCTTCATTCCACCAAGAATTTGCTCAAATGGCATCTTCTCAATGAAATCTCACATGACCAGTTTTTTTT[TTCCC]CCTGACATTTTCATAAAGATCTAATTGGCATGGAATAAACTGCATGTATTTAAGTGTACAATTAGATGTCGTGTCATACAAATAGAACTGGTAAATCACCACCAGAAACAGAATAGAGAGCATATCTACCACCTCCAATGTTTGCCCACG

>B1:196252326-196252626|varpos=196252476

TTGTTNTCTCTCAGTTCTGGANGCTACAGGTTTGAGACCAAGGTGCCAGCATGGCTGATTTCTGTGAGAGCTCNCTCTTCCTGGCCCTAGAGACAGCCACCTTCTATCTGGGTCCTCACATAGCAAGGNNNNNGANAGAGAGAGAGAGA[GAGCAAGCAAG]AGAGAGAGCACAGATCTCTCCCTCTTATCTCTTCTTATTAGAGCCCTGATACCTTCACAAAGGCTCTACCCNCGTGATCTCATCTAACTCTGATTATCTCCCAAAGGACTCATCTCCAAATCTACACTGTAGGTTAGGCTTCAACCTACT

>B1:197210817-197211117|varpos=197210967

GCCTCTTCTTCTTCTGTACATCTCGCTTCTTTCTGTGTGNTTTTATAAAGACACTTGTCATTGGATTTAGAGNCATCTGAAGGNTCCANTATGATCTCCTCNTCTGAAGATCCTTGACTTAACTACATTTGCAAAGNNNNTNNNNNNNN[TTTAAAAAAAA]AAAAGCTACATTCTGTGATTCTGGGTGGACATGAATTTGGGGGGATGCTAGTCAAGACGTAGAGAAGCAGAATGCTAGGCATTTACTCAGGACANTGCCTCTACGATTCCCAGATCCAAGGGTACACTGACACCTATCAAGGTTTAAACC

>B1:198748660-198748960|varpos=198748810

TTGGTTGCANGTNCAACTCTTGATTTTGGCTCAGGTTATGAGCTCATNGTTAGTGGGATCGAGCCCCACATCGGNNTCTGCATGGACAGCATGGAGNCTGTTTGGGATTCTCTCCGACTCTCTCTCTCTNNNNNNNNNNNNNNGCCCCTGCCCCANTCACA[TGCGCTCGC]CCTCTCTCTCTCTCTCTCTCTCCCTGAAAAACATAAATAAACTTAAAAAAATAGCCAAGGGTATTTGCTTGGTTGCTGTAGTGTCCCCGTTTCTGCATGCACACCCAAACCCAGGTTCCACGAGACAGACTGCATCTCATGCAAACTCAT

>B1:200371131-200371431|varpos=200371281

GGCTTTAGCCTTCCCTNGGCTGTNTGACCTGAGATGNGCCATTATTACCTGACCACCTTTAGTGCTATTATGCCCCAAGNTGCCGTATTTTCTTTGGGTCTATAATAAGTTTAGCTGTAAAGTCTTTATTTTTNNNNNTTTATTTATTT[TGAGAGAGAGAGAGAGAGAGAGAGAGAGAG]NNNNCAAGCACATGCAGGGGAGGGACAGAGACNGAGAGGGAGAGAATCCCAAGCAGGCTCCACACTGTCAGCGTGGACCCTGATGTGGGGCTTGAACTCATGANGCAGAGATCGTGACCTGAGCTGAAATC

>B1:200841767-200842067|varpos=200841917

ACCCTGCCACATGAGGATTCTTGACTTCCTCCTTTTTGTGGGCCACTCCTTTGTTAGTGTTTCTTTTGGCATTTCCATCNACATTTCTAGATAACGTGCTATATAGCATATATATCGTAGCAATATACAGCTAGGTGGCCTGGTTTTTT[TTTTTTTGTTT]TTGTTTGTTTGTTTGTTTTTAAATGAGTGATAAATTTAGTTCTNACTGGCTTCCATCTTCTTACTGTAGTTACATTAGTATTCAGTGTGGCATTTCATGCCCAAGTAATAGTCACAACGATGTTCNGTGCTATGATTCTATTTCTATGT

>B1:203831816-203832116|varpos=203831966

AAGCACCCAACTTTGGCTCAGGTCATGATCTCACGGTTTGTGAGTTTGAGCCCCATGTCAGGCTCTGTGCTGACAGCTCACAGCCTGGAGCCTGCTTTGGATTCTGTGTCTCCCTCTCTTTCTGCCCCTCCTCTGCTCACACTTTNNNN[GTCTCTCTCTCTCTCTCTCTCTCTCTC]AANAATNAACATAATAAAATAAAATAAAAAAGCAAATAAAACACCTACACAAACAGATAACAGATAACCTAAGTGAGTGTGATCTGACTTTATTTACTCCAATACTAGGGCACACAGGANATGCTATGGTTGGCAT

>B1:204216423-204216723|varpos=204216573

ACTGGNNNNNANACGGANGTGAGGAATCGCTAAATTCTACTCCCGAAACCATTATTACACTNTATGTTAACTAANGTGAATTCAAATAAATACATTTAAACATAAATAAACNAATAAACAAACATTAAAAAGTTCATTTAAGGCCAACT[GAAAAAAAAAAAAAA]CAGACTTCAGAACATGGAAGAAGATGTTATGTAATGACACATGCAACAATTNAGCAAGAAGATGTAGTAATCCTAAAANTGTATACACCTTACTGCGGAGCATCCAACTATGTGAGACAAAACNGTAATGCAAAGAAAAATAG

>B1:204516176-204516476|varpos=204516326

ACATTAAAAATAAAAAGCTTCTGCACANCAAATAATCAACAAAGTGAAAAGGGAGAACCCACAGAANGGGAGAAAATATCACAAACTATATATTTTTGATAAGGGGCTCGTCTCCAAAATATATAAGGAGTTTCTACAACTCAATNGTT[TAAAAAAAAAAA]AAAAAAACTCTAATAACCCNATTTTATTTCTTTTTTAAAGTTTTTTANTGTTTATTTTTGAGAGACAGAGAGAGAAAGAGANNNNNNNNNNNNNNNNNNNNGTGTGAGCAAAGGAGGGGCAGAGAGAAAGGGAGACAGAATCTGAAACAG

>B2:902116-902416|varpos=902266

TGATATTTATTACTGGGCTGCAGNGGTGGNTGAGTATTCACAANTTAATCAACGTGATACATCACATCAGAACAAGAACCACCTGATCCTNTCAATATGTGCAGAAAAAGCATTTGACAAAACAGCATCCCTTCTTGATNNNNNNNNNN[AAAAAACC]CTCAAGAAAGTAGGTATAGAAGAAACACACCTCAACATCATTAAGNCANATACAAAAGACTCACCACTAATATCATTCTCAGTGGAGGGAAACTGAAAGCTTTCAGGAACACAAGGTTGTCCTTGGAACACAAGGCTGCCNATTCTCACC

>B2:3791182-3791482|varpos=3791332

TAATAAAATGTGTATGCTTTTCTCCTGCTAATTTATCTTTGGCNGTTTCATTATCAGACCTNGCAAGGAGCCCTAAAAGGGTTGGGGAAAAATTNTNNTCCCATACATGTTAGGNCTTAAGTTTGCCATTTTAGTTTTTGTTTTCTGGN[GTTTTTTTTTTTTTTT]TTTTTTTGTCTGTTTGTTTGTTTGTTTNTTTTCCCCCTCACTGTTACCCACTTTTTGTTTTCTAGGTCTGGCTTTCCTGTGGGCTACTTGAGTATTTTCTTTAGACAATTTGATTCTGATTCATTTATTGTCTTTGGATAGTTTTTTAGT

>B2:6329696-6329996|varpos=6329846

CTTTCCATTCTCAAAAACGCTCTTCTGTTAGCCNTAAGAACCATCCCTATTTCGATCTATCGTTCTAACATTTCTTATTTATAAACCTATAATGTGCTCGTTGTTTTGGTTTTATTTTTTAAAGTTTATTTATGTATTTTGAGAGAGAT[GGAGAGAGAGAGAGAGAGAGAGAGAGAGAG]TGCAAGTGGGGGAGGGGCAGAGAGAGAGGGAGANAGAGAATCCCAAGNNGGCTCTGAGCTATCAGCACAGAGCCCAATGCAANGTTTGAACTCNCGAACCCTTGAGACCATGACCTGTGCTGAAACCAAGAGT

>B2:6847081-6847381|varpos=6847231

CATAAAACAGTATCTGTTGCAACAANTCAAGTTCTGGGCTGCTTTGCATGGCATCCTTTGTTGCCCAAGGATGGCAGCATCTCTCAGGCCTTTGAAGACATGAAATGATGACAGATAAGGAACCTGCGCCAAGAAGCGAGAAAAAAAAA[ACAAACC]CTCACATTTTGATGTGCAACTACTTGATNCNCAGCCATCTAGAAATACAGAGAGGAGGCTGTCANATGCACTCAATGAACTGCTTTGGTAGCGCTGGATGATTAATTTCCAATTTTGCCCTGCTCTGTCACATAGTGGAGGGCACATTAC

>B2:8093266-8093566|varpos=8093416

CCTCATTTGACCCGTAGTGTTTGTTCTTAATTAGTTGAACGCTGAAGNGGACAAGGGATGTGTGGTGGANACTCCAAATTTCCAGCCACATCATGCGTTTACTGAGGACCTGCCAACGGCCAGTCAGCNTAGCCACACGTTTGGATTCA[TGGCGTTTGG]GCTTTTTCCTCANTGCAAATGGCTTTCNGGGCCCATTTCCCATCAACTGAAGAGACCTGCCCATCTCTCAAGCATAGGCTTAAAATTGACCTTCTCCATGGAGCCTCCAGAAATAATTTCGAGTTACTACATACCACAGNTCTACTTAGA

>B2:8400900-8401200|varpos=8401050

AAGTAAAAATGAAAACAATAAATCAGCCCATGCCATGCCNGCTATAAAAATAAGCAAACAAAAACAAANANNNNNNNTACCCCCACAAAACCCCTANTTTGATGATTACAGAAATAAAAGANTAAGCCAAATAAAGGGCCAANNNNNNN[AAAACAAAAAAACCAAAA]AAANNNCTGNCACTAATACAAAATACAAAAAGATTATTAAAAAATTAAGTAGCATCTGGGTGGCTCAGCCAGTTAAGCAGNTGCCTCTTGATCTCCGCTCAGGTCATGATCTCACGGTTCACNAGATCGAGCCCTGTGTGGGGCTCTG

>B2:9025610-9025910|varpos=9025760

TCAGATAAACCTNTCAGCATTTAGGGTCTGTAATTAGTTGCTGAAGGCAGTTAAATCAGTCAAGAACAGAGTAATCCCTAAAGAGGGCCTATTGTTATGTGATGCTATGAAGGAAGACTTTTTTTAGTTTGCTGTAGTCCCACTTNNNN[TTTTTTTTTTTGTTTGTTT]NNNGTTTGTTTGTTTTGTTTTTNNNNGCTTTTGTTGACTTTGCTTTTGGTNTCAAATCCAAAACAATCATCACCAAAACCTATGTCAGGGAGCTTATTGCCTATGCTTTTTTCTAAGAGTTTTGATTTCACCTCTTGTGGTTAAATCTTTATTCT

>B2:9473663-9473963|varpos=9473813

CAATAAACCACTTAGGTTNNNATAAAGATTAGTCAGTTCCATATAAATCATATGAATAAAGCTTAATTTTTTCAAAATTTCNTATAGACAACAATGATTAAGTTTTTCTGAGNATTGACATCAAGCACCGTCCTAAGTGTTTTGTATAC[CGTGTGTGTGTGTGTGTGTGTGTGTGTGTGT]AGACAGCCATCTTCTAGCACACATATGACTTCTCATAAATTCCCATNACAAATGAACAATGAAGGTGCTGCTGTTACCAGTTCATTGTAAACGAGTTAATGGAGGCANGATAGATAAAATAATTT

>B2:13162895-13163195|varpos=13163045

GGTCCNTGAGTTCGAGCCCCGCGTCNGGCTNTGTGCTGACNGCTCAGAGCCTGGAGCCTGTTTCAGATTCTGTGTCTCNNNNNNNNNGACNNTCCCCNGTTCATGCTCTGTCTCTCCCTGTCTCAAAAATANATAAAATGTTAAAAAAA[AAATTT]TTAAAAAAAAAAGGCATACAGNNNNNGAAAAAGAAAAATCCCTTTCCCACACTTGGTGTTCTATGGCCCATTTCCCCTCCCCAGGTGAATAACGTGTTTAGGAGCTACAGCACCTTGTAAGCATTGGTAGTATTTAGTGCTATTCCACTT

>B2:14100589-14100889|varpos=14100739

ATTCTTCTTGTGGGGAAGCTGGGTTTAGGCTTTATTGACACTTTTGGNNTGTNNNNNNGTGTGTGTGTACNTGTATATACATATANGTACATACATATTTTAAAAATATCTTAATATATATTTCTATCATCTGTCTCCTATCTATCTTC[AATCTATCTATCTATCCG]NTCTATCTATCTATCTATNTATCATCTTATTCTCTANNNNNNNNNNNNNNNNNNNNTATCTATCTATCTATGTTTAATATCAAAATTGTTACCTTCCCAGAGTGTGGNTTATCATGAGCACAGCTGAGTTTGCTCTATAAGCCTGTGCTG

>B2:15549883-15550183|varpos=15550033

CTAGGATGAGCACTGTGTGTCCTACGTGANNGACGGATCACTCANTTCCCCTCCTGAAACCATGATGATGCTGNATGTTAACTAGCTTGGATTTAAATAAAATTAAAAAATAAATAAATAAACANGGACCAGTGAAATAAGAAAAAAAN[AAACAAACCAACAA]NCAATAAAAGGCCACTCTCACTAANTCTGCTTCTCTCTGNAGCGCTGCNACGGTCTCTGAAGGGGAATAAAAAACGCAGCCACCATATAATGAGATTATGCACTTGGCCAGCNGACTATCTCCTGTGAAANNTGACAGGAAGGGTGACTGTA

>B2:16095016-16095316|varpos=16095166

CTCTCTGCTTCAGATTGGATAATTTCNATTTCTTAGTCTTCAAGGTCANTAATTNTTCCATAATATCCAATCTGCTGGTTAGCTCATCTGTTAAATTTTTTATTGCAGATATTTAATTTTTTCCNTTNTAGAATTTCCATTTGATTCTT[CTTTTTTTTTTT]TTTTNNNNNTAGTTTCCATTTCTCTTCTGTATGCCNTACCGTTTTCTTCCTTACAGCACTCTAGCAGACTTTAGCTTAGCTGNGATAGCTACTTTAGATTACCTGCCTGCTAATTTCAAAATCTGGATCATCTTGAGGTCAGTCTCNA

>B2:17169472-17169772|varpos=17169622

GGTTCANGAGTTCGAGNCTCNTGTCGGGCTCTGTGCTTACAGCTCAGAGCCTGGAACCTGCTTCANATTCTGTGTCTCCCTCTCTCTCTNNNNNNGCTCCTCCCCCATTCATGTTCTGTCACTTCCTCTCTCTCAAAAATAAATAAAAACATCAAAAAT[TAAAAAAAAAAAAA]TAAAAGCAATNAGANTAAAAGGTAGATTACCTTTTATTAGACTAAAGGCAGAATTCTCATTAGCATTACAAGAGACCAGAAAGCAACAGAATAATGCCTTCCAGTAATGGGAGAAAGTGGTTGTCAACCGGGAAAGTTCTAT

>B2:20031199-20031499|varpos=20031349

TGTTNGTGTTGTTTACTTTGGAGAATGCAATTATCATGGCNGTGGNTATGATTTTTGGTAGTTCAAAAGCTACCTTATGATTTCCCTACACACAATATGAAATTAAGTTTGGTGTTTTGCCCACAAGAGTATTCATGTCACTCCTGANA[GTTTTTTTTTTT]TTTTTTTTCTCCTTTCATTCCCACAGAATGCCAAGAACTTCCAAAATGCATTACCATGCAAATGTCTTGGGTGACTTTACTACACATTTATATTGCNAGGTAATCAATAAATTATTATTTCAATATTTATTGGAATCTCAGAATAAGAAA

>B2:22023290-22023590|varpos=22023440

CTCTGTGCATTGCCCAGGTATAAAACCAGTTCTTTGTTCAATTGGGTATATATGATATTAGAAAGTAAGATTGACATTGGTGTAAATTTTCACCTCCTACTTGCATCATTCATTTGTTCATTCATTCAACCTTTATTTTCTGGGGGACN[TAGAGAGAGAGAGAGAGAGAGAGAGAGAGAGAGAGAGAGAGA]TGGATAATCTGTTTTTCTGAACTTCTGATTCTTCTGTGCATTGANCCAAGAGAATTCCTTTTCAGAGCAAGTAGAATGACACATGTGCCAAGAAATGACTAGGTAACAGTTTCTGAAGTGACCCA

>B2:22201371-22201671|varpos=22201521

GAGTGGTAGGTCAGAGAGAGAATCCAGAGGGCAGGATTACAAAGGGACATAAGGACATTTTTGTGGTGATGGGTGTGTTTACTATTTTGATTGTGAAATTGTACACTTTAAATGTGTGCAGTTTATGGGATATTAGTANCCACATAAAT[ATGTGTGTGTGTGTGTGTGTGTGTGTGTG]NNNNNNCATATTTGTTTACAGTTGCATGAAACCAGGCAAATAGTAGGCAAAAGGGAGAAGAAAGANNNNNNNNNNNNGGAAGATAGAACAAAGTATTGGAGATAAGACAAGAACAAGGATTCTCCAAAGAATCAAGCAAAGTTATA

>B2:22508219-22508519|varpos=22508369

CACTGTGAATACACTTTAATTCTTACTGAGAGACATTTTCTTTTCTCAGTTAACAGAGACGGAAGACATTAATNAAATTACTTTCAACATGGTGGCTTTGCTNNNNNNNNNNNCTTTTNNNNNNNNNNNNNNCACAAGCAGGGGAGGNG[CAGAGAGAGAGAGAGAGAGAGAGAGAGAGAGAGAGAGAGA]CTCTTAAATAGGCTCCACACCCAGTGAGGAGCCCAATGCAGGGCTTGATCTCATAATAGCGAGATCATGACCTGAGCCAAAATCAAGAGTCGCATATTTAACTGACTGAGG

>B2:23128316-23128616|varpos=23128466

GTAAGGTTANTGATGGCTAGGNGCAGTAGCTAAATNGGATGGATTCAGATCCTTAGCAAGAAGTGCCTACCACATCCCAATATATGGCAATTAATGAATTTAGTTAGGTCATGATGATTTTATTTTTTTAACTTTTTAATGTTTATTTT[TGAGAGAGAGAGAGAGAGAGAGAGAGAGAGAGA]ATGTGGGTGGGGGAGNAGCAGAGAGAGAGGGGGACACAAATCTGAAGCAGGCTCCAGGCTCCGAGCTGTCAGCACAGAGCCTGATGCAGCACTCNAACCCACAAATCCGAGATCANGACCTGAGCTGAAGTNAG

>B2:24608657-24608957|varpos=24608807

TTACCAGTTATAAAAAGAGAACTTCTCCCTTGGAATAGTAATTAGCAGTTGAAAGACATTTTTATAGAGTAAGACTTTAAAATAGGCTAAAAGTTTATAGTNTATAAAGAATAGAGAATAAGGCACATTCTTGAGAATGTAAGTGTGGA[GTTTTTTTTTTTTTTTT]NNTTTTTTCTTTTAAAAGGGGTCCATTATTATTCAAGTTCAGGGTATGCTGACTCAATGGGAAAAGTGNGCCCTCAGCAGANAGGCCAGAGACGGGATGTGAAATCTGTGGTGAANCATCTGTCTTATGGGGCTTCCTCTCCCCCATCNG

>B2:25105982-25106282|varpos=25106132

GCCAGGACTTAAGGCTTTAATGCTCTGATGTATTTCTTCCTGTCTTCCACTTCAGGAAAACTGATTTCTCATCATCCAAAAGGGAACATGTGATTAATGGCAAGTGTAAAGTGCCCACGGTTCTTTGATAATTGCTTCTTTTTTTTTTT[TTTTAAA]AACATCTTTATAGCCACCTTCAGACTGTAGTTGATTTCAGGCAGTTGGGAAGGCATTTCTGGTGAGTCAAGGCTAAAAAGGTCATTATATGCAAGTGTCATACCTGTGTCCTTACATCAAATCACTGTGTTTCCCCTGCCCTGGCAGGAA

>B2:28537896-28538196|varpos=28538046

TTCTGTAACNAATATGTAAAATTATTCATAGAATGGTTTACAATTTTTGTACTGTCTGGTGTGTATTTTAAACTTAAATCACATCTCATTTGCAGTTAGCCACATTTTATTTTTTAAAATCTTTTAAAAATATATTTATCTATTTATTT[TGAGAGAGAGAGAGAGAGAGAGAGAGAGAGA]AAGAGAGAGTGCATGCATGCAANCTGGAGGAGGGACAGAGAAAGAGGGAGACAGAGGATCCAAGCAGGCTCTGTGCTGAGAGCAAAGCAAAAGAAAACTTAGGAACTAAACAATGTAGATTTTCTTACGACTGC

>B2:33932262-33932562|varpos=33932412

AGACTCNGAAGAAGAGGTCCGTAGTGCCCTGAGGTGCAGTATACCCTATTCCACAGGGCCTGGAACTTCTGGGAGTGTCTCCAATATATGATGTGTGTGCTCTGTTATGTCCTAGCCACTTTATCCTTCAGGCCAGTCATCTGCAGAGG[CTTTTTT]CTTNNNACCTGTTGTGAGCAGTGTTTGNTCCCTGGTCTGAATGTGGTGTGTTTTTAACTAGGTGTGCTCTGGTCTGCTTATGAAATTAGACTTGTCACTATTGCCACCAAAATGGAGGCTCAACAAAACTCCCAAGTCAGGAGATGCAT

>B2:37374632-37374932|varpos=37374782

TTTTAATTGGGTTGTCTTTTTGTTGTTGAGTTTTTAGTTCTTTNTATATTCTGGATATTAANCCTTTNTTAGATATGTGATTTGCAAGTATTTTCTCCCATTCTGTGGGTAGTGTGTTCACTTTCTTGATAAGGTCCTTTTTNNNNNNN[AATTTTTTTTT]TTTTTTTAATGTTTANTTTTGAGAAACAGAGAGAGACAGAGCATGAGNGGGGGAGNCAGAGNGGGGAGAGAGGGAGACACAGAATCCNAAGTAGGCTCCAGGCTCTGAGCTGTCAGCACAGAGCCCAATGCAGGGCTCGAACCCACAAGC

>B2:38549344-38549644|varpos=38549494

TTTCCCTGATTCCTTCAGCACAGAGTCTTACATTTGGGGGTGGGCAGAGAAGAAATAGTAGAATAAATGAGTAAAAGAAAAGGTAGAGTGAATGAGACTGTGGAGCCATTAACCCTACCTCCCCATCACATGCACACGCGCNNNCGCGC[GCACACACACACACACACACACACACACACACACACACAC]ACACANNNNNNNNCACACACACACACACTAAGGCTGGATCACTCCATCTGGGATTTTACCTCCCCTCANTCACCCTGANTTCCTCCAAGTCTAGAGAACCCATCTGTGTAATCACGGCCCACCCCACCCCCANNATGACTAAATGTAGC

>B2:41309718-41310018|varpos=41309868

TTTTGTATCTTAAATTCCACATATNNGAGTTAAATCATTTGATANTTTTCTTTCTCTGACTGACTTATTTCACTTAGCATAATANACTTTAGTTCCATCCTCATTGTTGCAAGTGGCAAAATTTCATTCTTTTTGATTGAAGAGTAATATTCC[AGTGTGTGTGTGTGTGTGTGTGTGTGTGTGTGTGTGT]ANCACATCTTCTTTATCCATTCATCAGTTGATGGACATTTGGGCTCTTTCCATACTTTGGCCATTGTCAATAGTGCTGATACAAACATTGGGGTGTATGTGCCCCTTTGAATCAGCAT

>B2:41778382-41778682|varpos=41778532

GTTCTATAAGTTTTTGCTTCATATGCCTGGTTTATGGCTTTAGGACCCATTTTGACTTATTTCATTATGTCAAATAACNGCTTTAATCCTGTTCATGCTTCTATCATTAAAANTGGGATTGTCTGCTCTTCACATGGCCCCAGGACNTT[ATTTTTTTTTTTTTTT]GAATCAACCTTTTATAAGTTTTCCCAGACATTTATCTTCAGTTTTTTGATTGTCTTAGGTGTATTTTATAAACAGCATTTAGTTTTTAATCCCATCTGAGAGTGTGTCTTTCAGGAATCCACTCAATTCACATTTATGTGGT

>B2:43114678-43114978|varpos=43114828

AAGNGGGACAGAGGATCTGAAGTGGGCTCCGTGCTGACAGCAGAGAGCCCNATGTGGGGCTTGAACTCATGAACCGTGANATCATGACCCGAGTTGAAGTCAGATGCTTAACCNACTGAGTCACCCAGGGCCCCCAAATTTATATATAT[ATATTTTT]NTTTAAATAGGAANTGNATCTAAGGGGTTCAAAATTCAAGAAGTGTAACAGTATACAAAAGAGTGAAAACTACCCCAAGTAAATAAATGCTTGAAAATAAANTGAAAAGTTAGAGCAAAATTTTTAAAAAGGGTATACATGAAAAGTCCT

>B2:43335348-43335648|varpos=43335498

TGGGTGATGGGTATTGAAGANGGCACTCGTTGTGATGAGCATTGGGTGTTATATGNAAGTAACGAATCACTAAATTCTCCTGAAACAAAACAAACCAAAAAGGGCATCACATTAAAATAATTAATTTTNNNAAAAAAAAAAANAAGAAG[AAGAAGAAGGAGGAG]GAGAAGCAGTGGTGAACAAACAGGTAGACTGGTAATCTAAACTGTAACAAACATTGACCATTAAAAGNTACTAATTTGGAGGGGGNGCCTGGGTGGCTCAGTCCGTTAAGCGTCCTCCTCTTGATTTCAGCTCAACCATGATCAAGCC

>B2:44128421-44128721|varpos=44128571

CATCTCCAGTCAANCTTCCAGATGACTGCAGCCACATGAATGAGCCCAGGCAAGACCAACAAAAGAACTACTGAACTGAGCTCATATTACCATGGGTTGTTNNNNNNNNNNNNNNNNNNNNNNNNGTTGTTTTAACATTTATTTATTAT[TGAGAGAGAGAGAGAGAGAGAGAG]NNNNNNNNAGAAGAGCACAANTGGGGGAGGGGCAGAGATGGGGAAGACAGAGGATCCAAAGCAGGCTCCACACTGTGAGCACAGAACCCGATGTGTGGCTNGAACTCATAANCCATGAGCTCATGGTCTGAGCCAAAATCA

>B2:47527471-47527771|varpos=47527621

GTCCNTGAGTTCGAGCCCNCGTCGGGCTCTGGGCTGATGGCTCAGAGCCTGGAGCCTGCTTCTGATTCTGTGTCTCCCTCTCTCTCTGCCNCTCCCCCGTTCATGCTCTGTCTCTCTCTGTCTCAAAAATAAATAAACGTTAAAAAAAA[AAAATTT]TTAAATATCCAAACTAAACCTTGAATTCCCCNGTCAAAATGAGGGAGTTCTTAAAATATATNCNACTAAAATTAGAGCTTTTAGCCACAGGAAATATAACTGCAGAGACAGGAGAAAGTTGGCACCTATGCCACACAGTGGAGAAGCTCA

>B2:47622267-47622567|varpos=47622417

ATTCTGTGTCTCCCTCTGTCTCTGACCCTCCCCTGTTCNTGCTCTCTCTCTCTGTCTCAAAAANNNNTAAATAAAGGNAAAAANNNNNNNNNNAAAANNNTGAAATTTCCCTACAGGTAAAATTTTCTGTCTCTCCTTNTCTCTCTCTCTCTCC[CTCTCTCCCTCTTTCTTT]ACTTTTTTGTCTCTGGTTATCTAGATCACTAAAAACTCCTTTATTTTGGATTCTGGACTGTGGATCCTACAGGTCACAAAAAGAAGACTGATTGATTTGGCTGACTTCAAACATGTTAATGGCAAGGCCNGGATCCTACAGNNTT

>B2:47942564-47942864|varpos=47942714

CTTTTTTTCTAGAACTGTTATTTGGATGTTGAATTGTCTGGATTGATCATCTGTCTTTTATTTTTTATATTTTCCTTATGTCTTTTTGCTTCACCTTTTCTGGAAAACTTCCTTGGATTTTGTCTTCACTTCTCCAAAAGAAAAAAAAA[AAAAATT]TAATGGGGCATCTGGGTGGCTCAGTCCATTGAGCATCCAACTCTTGGTTTCAGCTCAGGTCATGATCTCAGGGTTCCTGAGTTTGAGCCCNGCATGCATTGGGCTCTGTNCTGACAGTGTGAAGCCTGCTTGGGATTCTATCTCCCTTGT

>B2:48350043-48350343|varpos=48350193

ACCAATATTGAAGCAGACACTTGATTTTACTACATATATTCTGTTATAAATTTTCTCAAACTTAAAAAAATTGAGACCTTTGCTGGAGAAAAGAGTTTCAGAGNCTTCCAAGTGCCAAAGGAATATACAGTACATATATGTTTTATTTT[ATCTATTCTATTCTATTCTATTC]ATTCTATTCTATTCTATTCTATTCATTTTTAAGTAGGCTCCATGCCCAANGTGGGGCTTGAACTTATGACCCTGGGATCAAGAGTCAGATGCTCTATTAACTGAGCCAGCAGGGGGCCCCCANTACATGTCCTTTAAATGTAGTTAT

>B2:48676335-48676635|varpos=48676485

AATAATGAGGNNNNGTAATATTTTAGGAAAAGATCTATGAAGACTAAACTCAGATGTTATTATAGTAGNACAGNGGCCNNCNAATCATATTTTAAGGATATGGGAAGAACCTCAAAGGGAGGTAACACATGCTTTGAGTCTTNAAAAAA[AAATTTT]TTTTNAAACATNTATTCATCTTTGAGAGACAGAGAGAGAGAGAGAGAGAGAGAGTGGGGGANGAGCAGAGAGAGAGGGAGACACAGAATCTGAAGCAGGCTCCGGGCTCTGAGCTATCAGCACAGAGCCTGATNCAAGACACAAACTCAC

>B2:48930637-48930937|varpos=48930787

TGATAGATACACAAATTATTGTAAAAAAATAACTACAAAAATTATGCCAACAAACTGGACAACCTAGAAGAAATTAATAAATTCCTACAAACATGATTTTTAAGACTGAATAAGTGAGAAATAGAAAATATGAACAGACNAATTACTAG[TAAAAAAAAAAAAA]NTTGAATTGATAGTTAAAAANNNNNNNTCCCAACAAAGANAAGTTAATACATATTCTCCTCAACCCTTCTAGAAGAGGAAGAAATGATTCCAAATANATTCTAGGAGGACAGAAAAACCACACAAAGACACTACAAAAAAAC

>B2:50466777-50467077|varpos=50466927

AGTTATATTACAANTTATCCTTTGCAAATTATNTATAAATGGGATTGCTTTTGGTAGTTTTTATGGGACCTATGGACAATAGGTGCAAATCAGTTCCACAATCCAGAGCTAAGGNAAAAACACTGGACTTTTAGAGAAGGATTCAAAAC[CTTTTTTTTTTTTTT]CCCCCAGTCCTTAGCACCTCACACACCTTGGGTGTGTTAAAGTTGAAGAGAACATATTTTGAGACAATTATCAGTGTAATGAAAGNNNNNNNNAAATGGAGTGNGGATAGGATGTGCCTCCTCCTTAGCTCAAGTCTAGTGA

>B2:50652050-50652350|varpos=50652200

GTTAATGGCCTCTCTTCATTGTACAATTTTTCTNGGAGAATATGTCTGGATACTATCCTCTAACACACCTTATTACTTGGCACAACTTCCATTGGCTCTGGTCTTTACTTTCAGCCATCCCACTCCAAACACTTGGGAATTCTTTCTTT[ATTTTTTTTTTTTT]AGTTTATCTATTTATTTAGATCGNGANCAAGTGGGAGAATGGCATACACAGAGAGAGAAAGAATCCTAAGCAGTCTCTGCACTGGTGCTCAGTCNCACAAACCATGAAATCTTAACCTGAGCCAAAGTCAAGAGTCANACACT

>B2:52305123-52305423|varpos=52305273

GATCAAGAGTCTCACGCTCTACCAAGATTCTTAACTAAATTGGTTANATGCAATTCTATCTGAAAGTAAGGGAATTACTAATAGTTCCTAACAATTTGTGAGGCACATTTTCTGGGATCATTCAATGTCCNTAATAGACCTACTTGCTA[GAAAAAAAAAAAAAAA]AAAAAAAATACACAAAAATGTACTCAAATTGTTTTATATGACTCAGGATGTTGATAGAATCCTTGAGTACCATCCTGGATCTTAANAAACCAACAGAGTTGGATTCCTCAGTTCCTCTCCATTTTTAGACACTGTAATTAAAAGTCTTT

>B2:53620870-53621170|varpos=53621020

CCCAAGNCAGCGACTTCTGGGTAAGAATGGTAATTACATGTTTAGTTTCATAAGACATTGCTGAGCTGTTTTCCAGAGTGCCCCATTTTACAGCAAGGTATAAGTGATCCAGTTTTTTGGCATTCTTACCAGCATTTAGTATTATCGCT[ATTTTTTTTTTTTT]NAATTTTAGCTTTTCTACTAGGTGTGTAGTAATAACTCATTGTGGCTTTAATTTGCATGTCCCCAGTGACTAATGATGTTAAACATATTTTCATGTGCTTTTTGGCCCTCTGCCTCTCTCCTCATCAGTGAAACATCTCTTCA

>B2:54513408-54513708|varpos=54513558

NNNNNNNNAAAAACCCCTAATTAGTTAAGATTATACAAAGGCTAAAAGAATCCTTTGNCTATTATATCGAATGGCTTCCAAGAATATTTTAACTTTTTGAAAAAAAGTNTAATTATTTAAATTACTTAAAAATGCCTCCCACATGGATT[CTTTTTTTTTTTTT]NNNNAAGTTTTTTTATAAAACATTTTAATGTTTATTTATTTTTGAGAGAGAGACAGAGTCACAGAGCAAGCAAGGGAGGGACAAAAAGAGAGGGATACACAGAATCCNAAACAGGCTTCCAGGCTCTGTGCTGTCAGCANAGAGCC

>B2:54936740-54937040|varpos=54936890

ACCTNATGCACCCTAGATTGTTATTGTCCCACCCCATTTAGCCAAGATATTTGCTTTTTATTTTAAGGCTCCTGTGACTAATCACAAATAGNTTAAAGTCTTTGTTTACATATTGTTTGTTNAATGTTCAGTTATTAAGTAATGAGTCA[ATTTTTTTTTTTTTTT]AAGTTCAATTATTTTGAGAGAGAGGGACTGAGCACAAGCAGGGGAGGGGGAGGGAGAGAGGGNGAGACACTCTCAAGCAGGCTCTGCTCTGTCAGNGCAGAACCTGACATAACACTCTGTCTCACACACAGNGAGATCATG

>B2:55642052-55642352|varpos=55642202

GGCACCTAGGTTGTCAGGCACAGTGCCTATGAAATCTGATACNTCTAATGATACTGAGCAAGTAGGAAGAGAANCCATGAAGAGAAGGTGGCTGCTTTTATACTAGGCTATCTCATTAACTCAGTGATTTTGCTTAGAAAGGAAAAAAA[AAAAACCC]CTNGTTTTATATCATTTAGAAAAACAATAATCTTATTGACCTTCCAATCTGTTACTGTCATTGGATATAGCCTGAGAAAAGAAACAAAGCTTTNACAGAATTATCTTTATTAACCAGTNNNNNNNCAAATAGGAGTGAGGAGAAAGGGGA

>B2:56230241-56230541|varpos=56230391

CTTCTCTAATAGATTATTNTCATCAACATACAAACATGNTGTAAACACTCCTATCTTCAAAAAGTATATATTTAGTATNGATCCTACTAGNTACTANCCCACTTNTTGGGCCTATTTTTCTTTCTTTTTTGTTTAATTTATTAAAAAAA[AATTTTTTT]TTTTAANGTTTATTTATTTTTGAAGGAGAGAAAGACAGAGTGTGAGCAGGGGAGGGGCAGAGAGAGAGGGAGACACAGAGTCCAAAGCAGGCTCCAGGCTCTGAGCTGTCAGCACAGAAGCCCAACATGGGGCTCAAACCCACAGGCTGT

>B2:58111157-58111457|varpos=58111307

GGAGACTGAGAAGTCCCAAGATCTGCAGTCAGCAAGCAGGAGACCAAGAGAGATAATGGTATAATTCCAGNGTGAATCTGAAGGCCTGAGGAAAAGGAGAGACAACAGTGTAAGTTCCAGTCTGAATTANAGTCNAAAGNCTGGAAAAA[AAAAAAAACAAAA]NNNNCCCACTGTCCCATCTGGAAGATAAGTCAGGCAGAGAGAAAATGAATTCTCNCTTACTCTGCCTTTTTATTCTATTCAAGCCTTCAATAGATTGCATAAGGCCCACCCACATTGGAGAGGGGAATCTGCTTTACTTAGTCTACCAAT

>B2:58321740-58322040|varpos=58321890

GGATATATGAGAATTTTGTTTGAGATGCTATTTAAGTTTAGAGGGGNAATGAACTAGTCAATAAATGATGTTGAGATGGCAAAGAAATAGGGGCATGTGTATATTCACATAATATAGTTAGGATAGATTAAAGAGCTAAATGAAATTCA[CACACAAAAAA]AAAAGCCTCTAAAAAGATTCTAAAAAGATTACAAATACTAGCAGTTTTTCTTTTATTTTTTTCTCTTNNNNNNNNNNNNNNNNNNNNTGAGAGAGAAAGTGCACTTGTGCATGCATGAGCAGAGGAGGGGCAGAAGGAGAGACAGAATC

>B2:59256382-59256682|varpos=59256532

TCTGTTTTTTGTCTCTATGGATCTGACTACTCTAGGTACCTTATATAAGTGAAATTACATGGTATTTATCCTTTTGTGATTGGCTTATTTCATAGCTTTAAGTNACATTCATGTTGTGTCATGTGTCAGNATTTCCTTCTTTTNNNNNN[AAATTTTTTTTTTT]TTTTTTTTTCTTAACGTTTATTTATTATTGAGAGANNNNNNNNNNNNNAGNGCATGAGCATGGGAGGGGCATAGAGAGGAGGAGACACAGAATCTGAAGCAGGCTCCAGGCTCTGAGCTGTCAGCACAGAGCCCAACGCAGGGCTCAAAC

>B2:59748556-59748856|varpos=59748706

TCACCAGTTCNATATTTTTTATAAAGGGATAATGATATTATGATGATTCACTTGTAGGTCTNCCCCATTGTGTGTGGGGTTCATACTAAGAAGAAACAGAACTTCTGCAATAATAAAATACCTTCCTTGTTCTCTCTCTCTCTCTCTCT[CTCTCTCTCTCTCTTTCTCT]CTTCCTGAAACTAAGCTATGAGGACAGTACATGCGATGATTTTCTATTGCCAGATCAGGAGATATTTTAAGTCTTCTTGTCATTTATAATCATAGNCTTTGCCAATGAAGCCAAAGAAATGTAGCCAGCCTGCTGTTCATATTTGCA

>B2:59855018-59855318|varpos=59855168

TTTGGAATTNNAATACAAATTAAATTTAAGCTGATCCAGGTGGGAGTTGATAAACCCCATAAAAGCAATTATAGTCATAAGGAGATGTTGTAAAATTGATATTAGAGCTAACTATTCAACCCACAACCCCTTCCCACCCTCCACCTCCT[TCACACACACTACACACACAC]TNNNNNACACACACACACACACACACAAGCACGCACACGGTGCTTTTCTTGTATGCATGTGCCAGGAGTGTATTCCGGGTCACCATCAGCTGCATAAAGGTATTGGTTTTAAATTTCCCTCCATTAAGTATAAGTCAAGAAAAACCA

>B2:59946583-59946883|varpos=59946733

AGGTTGTGAGTTCAAGCCCCACATCAGGCTCTGTGCTGACAGCTCTGAGCTGGAGCTTGCTTCNGATTCTATGTTTCNNNNNNNNNNNNNGCCCCTCCNCCACTTGTGCTCTGTCTGTNNNNNNNNCTCTCTCTCTCAAAAANAAACAT[TAAAAAAAAAAAAAAAA]GAATTGGGTTTTCTTGTCAACACTCATTTGATATAGACCTAAAGGAGGTAAAATCCTTGAAATCACTGTCAGCTCCTCTCCTGTGTCTTCAGATACACATTTGTAGAAATAGCATGGGGTCTGGGATACCAGAATTATT

>B2:61680719-61681019|varpos=61680869

CAAAAGAAAAAGTCCCACTTCTATACAAAATATAAATTTCTGCTTGCCATAATACAAACATTTNNACATTTTTAAATGTAGAATAAANTGTGACTTATGTCATCATACCTACAAAGATATTAAAGTCTTCTAGGTCTTCCATCTTGATTGTT[ATTTTTTTTTTTTT]NCTGTTTGTGGTCAACATGGTACAGAAAGAAAAAAANNNNTAAAGCTTCCCACAATGTTGATACTGTGTAATCTGTCCCATTANATATGAAAATTTGTACATTATCACTAGGCCAGTTGGAAATAATTAGTGTTAATAATAGGGAAAAG

>B2:62700022-62700322|varpos=62700172

CAGNAGTCAATTNCTTTTTTATATGCCAGCAAGGAACATCTGGGATTTGACTTTCCAAATTATAACACCAAGAAAAATGAAATAATGGGTACAAATCTACCAAATATGTATANGATCTATATGCAGAAAGNTACAAAACTCCGGTGGGG[GGAAAAAAAA]TCAAGATCTCAACAAATGGAGAAATACTACATTTGTGGATTGGAAGATTCAATATTGTCAAGATCTCAGTTTCTCCAAACTTAATCTATGGATTCAATGCAACTCCAATCAAAATTTCAGCAAACTATNTTGTAAATATCAGTNA

>B2:63784636-63784936|varpos=63784786

AGTTGATTNNNNNNNNNNNNNNGACTCAGGTCATGATCTCATAGTTCCNTGAGTTTGTGCATTGCATTGGGCTCTGNGCTGACAGTTCAGAGCCTGGAGCCTTCTTCGAATCTTGTGTCTCTCTCCCTCTTTGCCTCTCNCCCCTTGTGCT[TTCTCTCTCTCTCTCTCTCTCTCT]TTCTNNNNNAAATAAACATTAATTTTTTTTNNNNNNNAATTAAAAAAGAAATTCAAGGCAAAAGCATGTGGTAGGATATANAGGNGTAAGCATAATGTGTGAAGATCTTTGTATTACATGGTAACATGTAATATGCACTTTAAAT

>B2:64389219-64389519|varpos=64389369

TTTATGGAGCCATCATTGGAGCTACACCAGCANGCCTGAAAACCATGCACTTTTTGCAAAATACATTTTTATGTCATATTTAAAAAAGCAGAGTTTATGTAAGCACAAGACTGTTATAAGAAAGACATACCTATAGATTCTAAATGATT[CAAAAAAAAAAA]TAAAGAAAGCAAGATGAAGGTAAAAGATCTAAACTNGAGAACTGAATGCCAACAAAGGATGTTTTGATAATTNTAGAAAGANGGTTGGCTTAAAAAAAAGTCAAAATAACAGAAGAAGCAACTTCTGCCCACCAAGAGGCAAC

>B2:64445580-64445880|varpos=64445730

TTCTGCACNGAAAAAANCAACAAAAACAAAAANCATCAACAAAANGAATAGACAACATACTCAATGGGAGANGATACTTGCAAATGGGATATCTGATNAAGAGTTGGTATGCAAAATATATAACAAATTTATANAACTCAACACCCCCC[CCAAAAAA]AAACCCAAATAATTCAATTTAAAAATGAGCAGAAGACATGAACAGACATTTCTCGAAAGAAACATACAGATGGCCAGCACACACATGAAAAGATGTTCAACATCAACCAANACCTGGGAAATGAAAATCAAAATACAATGAGATATCACC

>B2:64934791-64935091|varpos=64934941

TAAATGTCACCCGATTCCAGACTGAATCCCCTGNATTCCCTNTGAGTCCCCTGGAAAATCAGTCTACCATTATTATACTACTCTTCTCTTTCTCCCAGCCTGCCCTCCTGTTGATCTGAGTCTCTAAAGGTCTCAGGGATCTACTGAGG[ACATATGGGGT]TCTTCTACTGGTATTGACTTCACTTTGGCTACCAGAGATAAGTAGCAGTGCCTCTACTACTTCTTTTGNCCTCTGGGCATGGANATCCTTCCAGTGCTTGATTTGGACACACNGACGTACCATGCAGCCNTTTCCCTGACTTCTTGCCCT

>B2:65708738-65709038|varpos=65708888

CCACATCTGGCTTAATGTGACTCATACTTATCTCCCAGACTATTCATATAGCATCCCCAGGCTTAACTTTGNNNNNNNNNNNCAGCATTTGTACTTCTTTCAGGCTCTTTTTCTTAGAAGTAGAAATGCTTAAGGGTTCTGTTAAAANN[TAAAATAAAAAAAAAAAAAA]NAAAAAAAGATGAGTTTTCTCACTTGGAACAAGAACATATGCAAAGTGCCATAGAACATATATCTCTGGTATNATATTCTCANAGTAAAACTTTCTTAATNTTTTAAGGTTTACTATTTAAACTAGAAAGCTGATTTATGTTAATG

>B2:67212909-67213209|varpos=67213059

TTTTTCCAGTTGTAGAAAATTTTGTAATTTAAATTAAATTTACTTTAATATACATTAGTTTTGTAATTTGAGGCTGNNGTATACTGTCTTCCAGTGGTAGAAAATTTTGTAATATAGTTAAATGCATTTGCATCCTAAATTTGAAAAAA[AAAACA]NCTTCTATTTCTGAACCTATCAATATACTTCCAGAATATTAAATATATATATGAATATATATAAAATCAATACTGTAAATTAAAAAATAGTTCTTATGAATGAATTATTATTAATGTGTTAGAAGGCAGGACCANTATTTCAGAAACATG

>B2:67423721-67424021|varpos=67423871

ACATAGGAAGTTATTCTGTCTCTATTCCCATTNNNNNNTTGGGGACAGTTACACAGAATTTGTTGATTTTATTGTTTGTTTTCTTTTGAAAATACTATTCTGTTTTNAGGCCATAAGCTCTTATTGATTCTGACCAAACCAACTCCTNN[ATGTGTGTGTGTGTGTGTGTGTGTGTGTGTGTGT]AAGCCAAAAATGAAATTAAANNNNNTTTTAATTACCAGTAGCAGTTCTTTTAAGTTACAACTCTTTGTGAACTAATTTTTAAGTCTAATTACATGAGATGCTAATAGCCTTCAATCT

>B2:67469750-67470050|varpos=67469900

CCTAAAAGACATCAATGATATGTTCTTATATCCCAGTAGTTGTCTTATTTTAGGAAATGATGGCATTACTTATAATTAAAGTTAATAATTCAAAAATACTCAAATTTGGGGCAGAAAGTCAGAAGGAAAGGTAGGAACAGGGAGAGGAA[AAGAGAGAGAGAGAGAGAGAGAGAGAGAGAGA]CCTCATGTAGATAGATGTTTTTGGAAATTGTGGATCACAGAGCAAAGGTGAGATATGTAAAAATGGAAGATTTAATCTGGAATACACATGCTGCTGAGGTCACCTGAACATAAGATATACCTGAAGGGTGGNG

>B2:70470616-70470916|varpos=70470766

AGCTCAAGCCCCAAGTNGGGCTCNGTACTGACAGTGCAGAGCCTNCTTGGGANNNNNNNNNNNTCTCTGCCCATCTCCTGTTCAATCTCTCTCTCTCTCTCTCTCTCTCTCTCTCTCTCTCTCTCAAAATGAATAAACTTTAAAAAAAA[AAAATGCAACCTGAA]AGACTGAACTTGGCAATGTTCTATNTCTTCCAATCTGCTTTGGAGTCTTAGAGGAATGACTATGTGTGCACAGGTGTGATTGGTGACCACCAGGCCTGTTTCTGAGTTTACTGCAAGATCAGGAAGATTCATTTTCCAGGTACAGCACT

>B2:71192457-71192757|varpos=71192607

GTTTCAGCTCAGCTCATGATCTCACGGTTCATGAACTCAAGCCCCACACTGGGCTATGNACTGACAGCACTGAGCCTGATTGGGANNNNNNNNNNNNNNNNNNNNNNNNNNNNNNNNNNNAGCTCCTCCCCCATTCCCACATGCATATG[TTCTCTCTCTCTCTCTCTCTCTCTCTCTCTC]AAATATAAATAAAATAAAACTTAAAANTAAATAATACATCTTTTATTGTTTGCNTTTTCTACCTTCCTGTACCACCCGTCCATCCAATGTCGGTATTTTAGCCCCTCCCAAATAAACTAG

>B2:73010962-73011262|varpos=73011112

TCCAGGCCTATCTCAAGAAAAAGAAAAATCCCAAATACAAGAAATAACAGCACACCTAAAGAAACTAGAAGCAAAACAGCAAAGAANCCCCAAGAGCAGCAGAAGAAGAGAAATAATAAAGATTAGAGCAGAAAGAAACAACATAGAAT[TAAAAAAAAAAAAA]NNCAGTAGAATAGATCAATGAAACTAAGAGCTGGTTNTTTGAAAAAATAAACAAAATTGATAAACCCCTAGCCAGACTTCTCAAAGAGAAGAGANNNNNNGAACCCAAATAGATAAAATCATGAATTAAATTGGACACACCACAACCAACCCCTC

>B2:75908414-75908714|varpos=75908564

CCTAAAGAAGGAAGAAAGGTCTCAAATACACAAGCTAACCTTACAACTAAAAAAGCTGTAAAAAGAACAGCANATAAAACCCAAAAATAGCAGAAGATGGGAAATAATAAATATTAGTGCCAAAATCAATAATATCAACANAAAAAAAA[AAAAAAATAGTAG]TAGAACAGATCAATGAAACCAGGAGGTGGTTCATTGAAAGAATGAACAAAATTGATAAATCCCTAGCCAGATTGATCAAAAAGAACAAGGAAAGGACCCAAATAAATAAAATCACGAATGAAAAGGAGAGATCATGACCAGAAGTGCGGA

>B2:76283439-76283739|varpos=76283589

AAAGAGTTCTAGGATTGTTTTGGTCCACCTTAAAAACCTCTCCATCCCCTGATCCTTTAAATTGTCAATTTCTAGATTTTCTTCACTATTAGGTCTTAAAGTCAAGGGATTAAAGATGAATTATTATAGGCAGAATATTATTTTAGGGG[GAAAAAAAAAAAAA]AAAAAAAACCTTTCACTTTCCTGGGCTTTTTGGTGGTAAAGTNNNAGGTTACTATTTTCATGGAATTGTTTACANGAGTTATAGTGCATAGTAGTGCTTCTCTTTCATTTTACTAGCAAAAGAACTCTTTTTCCCAATGAAATTTCATATAAT

>B2:77379706-77380006|varpos=77379856

ACCCTTTCTAATATGACAAGTTAGAACTCTTATTGCTTCGGTCACTCGTGTCTGTTTGCCAACGACCCTGAAAAGAATGGCATCCAAACTAAATGCAATATTCTTGACATGATGTTATATAAAGAACTATTGCCTCCATNTGAAAAAAA[AAACC]CTTTATTCTTAGTAATAACAACACTGATTTATAACCCTTCTTAAAAATACATAATGATGCCCTAATTAATATATTTTCATAAATACTGCTGGAGATAAAACTTGGCAATTTTCTTAATTCTCTGTATGCTGTGTATAAATTTAAATCAAA

>B2:78394860-78395160|varpos=78395010

GTGTTGGACTCTTGATTTTGGCTCAGGTCANGATCTCATAGTCATGGGATTAAACTCCACATTTGGCTCCATGCTTAACGTGGAGCCTGCTTGGGATTTTCTTTCTTTCTCTTTCTCTCTCTCTCCCCCCCTNCCCCTCGCCACTTGCN[TTCTCTCTCTCTCTCTCTCTCTCTCTCTCTCT]AAAGAAAAATATGNTTTTAAAAAAGTTTTAAAAAAAGAAAGAAAATGAGATGAAGAGAAATTACACAATGTACTCAAGACTACACAGCTTTCTTTGAACAGCATATCCTTATTTTATGC

>B2:78468081-78468381|varpos=78468231

CAAGCTAAAATAAACGGAAATAAAGTGCATGTTATTCATGAGCTACACNTGCACGTGCAAATCAGAAGGTCTAGNGTTTATAGGTGATTTTAAAGGTGATCTTTCATACATGGTGGTTTATAGTGTTACAGCTTGACTTTTGATTTTTT[TTTTTAAGTTAAGT]AAGTGAAAAGGGTTGCCTTTCCCTCAGATAGAAGAGGTGTTTTTGTATAGCCGATATAAATACTTCTAAAATTTTATAGGGTGGCTAATACCCTACAGGGATAAACCAGGCTGTCAACTTTCCTGAGATTTTTGGCTGGAACCCCAAA

>B2:78492076-78492376|varpos=78492226

CTCTACAGGGNAGAGAGAGAGGTAAAAATATGTAGTTTAATTCTTCCTTCCCTTCTTCCATGAATGGTGCTTGGACATGTGATGTGTCCTTAACATTTCATCTTACATCAGAACTATTTCTTCTTTCCCTCTGCTTTTGAAAAAAAAAA[AAAACCC]CCTAACTTTAAAATAGTCCCTGTTTTNTATTCATTTTTCAGATCCTTTTCCTTNGTCAAAAGTCATATAGAATTGAGATCTATGGGTGAATGGCTCCAAGAATTTTCAAAGTAACAAAAATAAAAGGAAAAGANNCTAATTAAGAGATTG

>B2:79948978-79949278|varpos=79949128

TGCTGCAATTATTGTTTTGAATTACCCCAGCAAGAAATATGCTAGTAATAAAAGCCAACCTCTATTAATATTTCTAGNGACTTCTTTGTACAAGTTTACCTAGTGATTTTATTTTATTACCAGAAAAGAATAATTTAATTTAATTTTTT[TTTTTAAA]AATTGTGAACTATATCACTCATATAGAATAGTATATTGTCTTTTTGATGTGTCAGCTTGGNTAATCTCTTTCCAGTTAGGGTGGGCTATAAGAGATATTCTCNCAGTTGGATGGACAGATGGATGACAGACAGAAGGGGCACACTGGCCA

>B2:80053340-80053640|varpos=80053490

ATTTAATTATAGTGTTATAATTATGCTTCAATTGTCACACATAATTTAGAAAATTCAAGAGGGNAAGAAAATCCTTTTCTACTTGTGTATAANTTTTTACTACTGCCTTCTTTCTTCATTCCTTATATTCCAAGTTTCCTTTTTTNNNN[CTTTTTTTTTTTTTTTTT]TTTTTATCGTTTCCTTTCTTCCTTTGCCATTACTTTAGGGTAGATCTNCTTGTAACAAATTCTCATACTTGCCCTTTATCTGAGAATGTCTTGATTTCCCTTTCATCCNGGTAGGATATTTTTCTGGACACAGCAGTTGGGNTTGAT

>B2:80463076-80463376|varpos=80463226

TGGCAAGGATGTCNACTTTCACCTCTGTTATTCAATACAGTACTGAAAGTTCTAGCCAGNGCAATATGGCAAGAAAAAGAAATTAAAGGCACACAAATTGAAAGGGAAGAAATACATCCTTATATGTAGATGACATAATCATNTNTGTT[TTTTAAAA]AAAAAAAGGAACTTACAGAAAAGTTCCTGGAACTAAGCAATGAGTTCAACAAGATTGCATGACNTAAGATCAATACACAAAATGAATTGNATTTCTTCATACTAAAAACAATAATGTAGAAACAAATTTAAAACACAATACCATTTGCAA

>B2:83004809-83005109|varpos=83004959

TGATCCCAGGGTTGCGGGATTGAACCCTGCGTCAGGCTCTGGGCTGAGCATGGAACCTGCTTGAGATTCTCTCCTTCCCTCTCAATCTCTCTCTCTGCCCCTCCCTTGCTTNNNNNNNNNNNNNNNNNNNNNNNNNNNNAAATTAAAAA[AAAATTTTTTT]TTTTTAAATTGTGGTAAAAAAACACGTACCATAAAATTTACCATAGTGATCATTTTCAAGTGTATAGTTCGGCNGTGGTAAGTATATTCATATCGTTGTGAAACAGACCTCCAGGANTTTTTAATCTTGATATCTGAAACTCCATAACCA

>B2:83814515-83814815|varpos=83814665

AAATGAAACTTCTAGCTAAGATCTCCAAAATAAACCTTGATGGATCATGAAGAAAGGATGAATCATGAGGAAGGGGACCTATATATTTCTTACCAGCTCTGAGAAAGGTAGTATCCCAATAAATATGAAGGTCCGGGGTAAAAAAAAAA[AAAAAAAC]CAAAAGCNATAAATATNTATTACTCAAACAATTCTCAGTTAAATGCTTTATGGAGTCTTTTCAGGTTAAATGGAATTTTGGGGGCCCATGCTCTTATTCAAAGAGTGGCCAGGTGGCTCACCCGCTGTCTCACAGTGTGCTGGTGACAGG

>B2:83909730-83910030|varpos=83909880

CAGTGCCCCAGCCATACCTCAACCTTTCAAGACTGTTGCTATCTCCAGGCAGTGAAGAAACAGTTTTTTCCTGGGCATTTTATGATCAGTATATGTAGTATATTTCTCTGGGAAAATTGAGATCTCTCTCTCNCTCTCNCNCTCNCTCT[CACTCTCACTCTCACTTACTT]TTCTCTTTGTGAGCATTCCTCTGGGATTATAACTACACAACCTGGTNGTCAGAGGGCTCTGTCACTGGGNAGTAGCTACAGAGCCAGATTGCAGCATCAGTTCTATTTGAGATTTGCCACATGATGATGAGGGGCAGTCCGTCTGCA

>B2:84445410-84445710|varpos=84445560

TCTGGTGGCTTATACTATTTATTTATNTATNTATTTATTTANTTANTTATTTATTTATTATTTTTNNNNCTTTATGNCCAACTTGGGTCTCAAACTCATGACCCCAATATCAACAGTTGTATGCTCTACTGCCTGAAGCAGCTAGGCACTCCAT[ATTTTTTTTTT]TTTTTTAAGTATTAAATAGGAATTTAGGAAGTGATTCTGAGATCAAGANCAAAGCCCTTCTGTGAGAAGTGACAATATCTATCTTTTATTTTTATTTCTCCTCTGAGGATCAAAAAAGGAAAAGCTGGGAAATGAATAAGATATTTCTGG

>B2:84510313-84510613|varpos=84510463

TAGTTGTNTGCTAAGCTAGTTTTGTATTTCTCTNNNTTTAACATAAAGGCGTGTAAATTNTGNTGCATTTATTTTGATGATCAGAAGCTCATTGATTCTCAGGTTGATTTCATGGGCAGTTCACTATTTGCTTAGTGCCATTTAGAAACTTCCN[ATTTTTTTTTTT]TTTTTTTCTACTATTTTGATAAACTATTACATTTAGCTTGCAATAGTAAAATGCACTTATTGCTAAACATCTTGATTACTAAAATTTCTGAAAGAAAATAACTTATTTCTAAGTCCACATTTTTCTAGTCACTTTCCTGCTTTGTTTTTC

>B2:85162811-85163111|varpos=85162961

AGAGACAATGTTTGGGCANGTTTCAATCATTCCCAAATGTGTATTAATTTGATTATTTATNTTTTTATATATAGAATTTCTATTTTANATACAGAATTTATATATAAAATATATAAAGAGAAGATTTCTGTTTTTATTTTATCTTTTTT[TTCCC]CTCTATTTACAATTCTTTTTTGAGATAAATGTGTATNNNNNNNNNAAGTGCTGTTGNGGAGGGAGAATTCTTTCTCTACCCTCAAGTTCTTCCAGCTGGAAGATATATGACAGATTAACAAGANNNNNNAACCAAGTTTTATTTGCACAC

>B2:85415477-85415777|varpos=85415627

TAATGGGAAGTCCTATTGACTGAATTGTTTTCTCAAAACCACTTANNAGGTATAAGTAAAATATGAACATTCCCGTGGGAGAAATGGTGCCAGCAGCTGCTATGAATGTTTGTAGAAAGGTTCTTGTGGGTAAAGAGTAAAAGAAAAAA[AAAAAAAAAGAAG]GNTATTGTAAATATAAGATAGGNAGAGATATTACAAAGCTTACCTAAAGAATGGCTTTANAGCATTTTTATATAAAAGTGAAGAAAACTTCATCATGTGCAAATTGTTCATTTTTATTTAAAAGCAGAAACATGTTTTAACGTTATGG

>B2:85463560-85463860|varpos=85463710

CCCCAAAATAACAATCCATAAAATAAAAATAAAATAATTGGCTGGACTTNAACAAAACTAAACACTTCTCTTCAAAAGACACTTAGAATGAAAAGATATGCTACAGAATTGGAGAAAATATTTGCAAAGTATAGATCTGATAAAAACAA[AAATTT]TTGTACCTAGACTATGTAACTGACTCTCAAAACTCAATGNTTAAGAAAACAATCCAATAATAAACATGCAAATATTGGAACAGTAAGTTTACCAAAACAGATAAANGGATAGATAAATAAGAACATAAAAAGATGCTCANTATAATTAAT

>B2:86583681-86583981|varpos=86583831

ACAATATTATTATTACTGACCAAAATATGTANGGATTCTAAATGAACAAGAAATGTTTCAAGAACCTCATATTTAACTACACAAACAGACAANATATCTTTAAATTAGAGTCACAAAGGTATCCCTATAACAGAAGAACCTCCCNCCCC[CCCCCAAAAAAA]AAAAACACAACCCAGAGATGTCACAGTATGCTGGGGACATGCGACATCACACTGGGTACTAGGAGGGCTCAGTTTGTTCCTGGTCCTTGGCTCCTAGCTGACATTCCAATGAAGGAGTCAGGATACAGATATAAAATCACAAGAAATTNT

>B2:87431312-87431612|varpos=87431462

CAAAGTGATTTTATTCCTCAGAGGATATTTGGCAATGTCTGGAGACACTTTTGTTTGCCACACTGGGGAGGTGCTACTGGCATCCAGTGGGTAGAAGCCATGGATGCTGCTAAACATCCTACAATGCATAGGACNNNCCNTNNNNNNNN[AAAGG]GAATTATGTGNGTTAAAATGTCAATAGTGCCAACCTTAAGAAACCCTGATGATGAAAAGAAAGTGGAATTACCTATTGTTGTCGTGNTCAAATTAACACAGGAGTGAAAGTAGGAAAAGCCTGGATGTCCAGGACTTATAAGTTAAATTG

>B2:88023218-88023518|varpos=88023368

TAATAGCAAAAGCCAGGANNNNNNNNGTCTTAGTTTTCTCTGATTGCATTATGAAAGGAGGAAGACCAGTCAGTGGCATCACCTTTNNNNNNNNNNNNNNNNNNNATTGTTTTTCCTTTTTAAAATTCTTAATGTTTATTATTTATTTT[TGAGAGAGAGAGAGAGAGAGAGAGAGAGAGA]NNNNNNNNNNNNNAGAAGGAGAAAGAGAGAGAGAGAGAGACATAGTGTGAGCAGGGGAGGGGCAGAGAGAGAGGGAGGCAGAATCTGAAGCAGTCTCCAGGCTCTGAACTGCCAGCATAGAGCCTGATTGGGGGCCCAAACCTGTGAACT

>B2:88207969-88208269|varpos=88208119

TTGGTGTTGGTTGTTCTTTCTCNTTCATTTCTGNNNNNNNNNNNNGAGGCCTCTTTTTTTTCTTGATGAGTCTAGCTAAAAGTTTATAAGTTTTGTTTATCTTTTCAAGGGACCAGTTTGTTTCTTTGATNNNNNNNNNCCTGTTTTTT[TTTTTC]NNNNNNATTTATTTCTGCTCTAAACTTTATTATTTCTTCTACTAGCTTTGGGCTTCATTTGTTCTTTTTCTAGCTCTTTTAGGTGTGGTTTTGGTTATTCGANNTTTTGTTTNTTGAGATAGACCTGTATTGCCATAACCTTCTCTCTTA

>B2:88644445-88644745|varpos=88644595

ACAAACTGAAAATAAAGNAANNNNANAAAACAAAAACAGAGTCAAAAAGAGTAAAAGGAAAACAAAAATAAAAATACCAGAATAGAGAATCCAAGATNTCTGGGATAATATCCAAAGTTCTACTATACATATAATTCTAGAAAAAGANN[CAGAGAGAGAGAGAGAGAGAGAGAGAGAGAGAGA]ACAAGCAAAAGAAATATTTGAAGACATAGAAGAAACTCTCTTTATAAACACAGGCAATGAACTAGATTTTTCNTGACATTTTTTTAAGAATAATGAAAGTCATGGAGCACCTGGGTGGCTC

>B2:91449364-91449664|varpos=91449514

CTCGATTTCAGCTCAAGTCATGATCTCANGNTTCANGGATTGGAGCCCCACATCNGACTCCATGCTGGCAGCACAGAGCCTNCTTGGAATTCTCTCTCTCCCTCTCTCTGCCCCTCCTCANCTCTCAAAAATAAATAAATAAACATTTT[TTAAAAA]AAAATCCATGAATTCATTAGAAGCTTCTCTGTAACATTAGTTTAAGGAGTTACAAAACCAAAGAAGGGATGACNGTTTGGGCTTCANGATGGAAACCTTAGANACTTAAGAGCATTGCATATCACAGAGAAANGTGGCAAAGTGGTGTGTT

>B2:92998026-92998326|varpos=92998176

TCATGAACTATCATGCATAATTTCTAACCACANTGCTGTGANACNTGAAGNCAANCANAAGAAAANATTTGGANAGACAANAAATACATGGAGGTTAAAGAACATCCTACTTAAAAATGAAAGAGTTAACCAGGCAATTAAAGAAGAAN[TAAAAAAAAAAA]AAAAAAAAATACATGGAAACAAATGGTAATGAAAATGCTATGATCCAAAATCTAAAAGCAGTTGTAAAANGGAATAAATAGCAGTGCAGGACTACTTGAAGAACCATGAAAAATCTCAAATATACAACTTAACCTTACACCTAAAGGAGC

>B2:94543682-94543982|varpos=94543832

GTGAGTTGGTCAGNNTCTGGATGCTGCGCATATATTGATAAAATAACTAGAGGAATTTCCAGCAACTGNTTCATTGTGTCACTGATGNAGTTGTCTCACAAATTCTTAGTCATTAGATCATGTTGATGGTCTCAATTGACTTTAATGTC[TACACACACACACACACACACACACACACACACACACAC]NNNNNNNNNTATAAANTTTGGTATTTTNCATTATATACTATTGTAAAAATGTAATTAAGTTACNACATAAAGGACTCAAACTACTTGATTTAGAGATATATTACAAAGCTTATATNNTCAA

>B2:95575505-95575805|varpos=95575655

TTGAAGCTGGGCCTTATCTTATTACCTGAGAAAGATTTTTACTCCTTTGAATTTCTGAAACNAATTTCTGATACAAAGCTTTTAGAAAAGTGTAAGGTACAAATGGGATATAATTCCATTTGGAAAGGGACTTCAAACATTACTCATTT[GTTGTTTTTTTTTT]NTTTTTTCTTTTNNNNNNCAATTTTAAGTAAATTGCAGACCAAGTGAAGAATCTATATTTTATTTTCTAATGAGCAAACTAAAAATACATGTAAANAACACTTAAAGAGTTCTCTTCACATNACAAAATATTTTTCTATAATTTACCT

>B2:97165455-97165755|varpos=97165605

ATAATGCCACANNACCAACCTGGTATNNNNNNAGAAGAGACAGAATAAATACCAAAAAGTGCCCTGCAGATTCACTTTTGACAGTGATAGATAAAAATGTACCTAGTGCNTTGCTAAAAAACAAAACAAAAATNNNNNNNNNNNNNNNA[AAAAACCC]CCACCAATTCTGATTATGGTCTAAAAGGAATGTTTCTGAAAAATACATACTTCATTAGATTAAAATAAACAGATTACAAAGGGAAACAAAATGTGAAAACACGGACTTGTTTTTAGAGGGGCAGNGGTGCCTGCAACATGTTTGAAAATA

>B2:97225853-97226153|varpos=97226003

TTTTTTTTAATCTTAAAACTAAGATTTTTATCTTAGGTTTTAATGCAAATTAATAATGGGCTTTGCTGTAGTAGCTTGAGATTTGTTGAACATTTTGGATCTGTGGATTTGTCTATTTTATCAAATTCGTAACGCTTTTGGCCTTTTTT[TTTTCCCC]CCTCCTTTANATTTTTGTGTCCCTTCACTTTACTGTAGGACTTCAGNTACACAATGTTAGATCACTTAGCACTNNNNNATAGGTCAGTGGGGCTGTGTTCATTTTTGTTTCAGTTTTTTTCTTTTTGGTTTATTTTCAATTGTCCTGTTT

>B2:97618933-97619233|varpos=97619083

TAGGGTTTGGTACAATCTGTGATTTCAGGCATCCACTGGGCGTTTTNGAATGTGTCACCTGCAGACAAGGGGAAACTACTGTGTAGCTAGAATGCAAAGTTTTTGCAATTTTGTGGGCAAAACTCAAAATATAATAATAATTGAGTACA[GTTTTTTTTTTTTTT]NNNNNATAAATACAGGTCTATTAATAAGAATAACGAAATTCTTTGGTGGGGGGAGGGCCTAACACTTCCTTAATCAAGGTTTAAAACTAGTGATTCCTATTACCAAAATGGGTTGCAAATGTTCATCTGTTAATGCTGATCTGGAATA

>B2:98439046-98439346|varpos=98439196

TGGCCATTATATGTTAAAATATTTTTTCTGCTCTCCCCCTTTNCTGGGACTCCATTTATATGTGTGTTAATTTGTTTGACNTCATCCAAGAGCACTAATGGCTCCATCCTAGTTTCTNNNNNNNNTTTTTAGTCTTTGCGCTTCTTTTT[GTTTTGTTTTTTTTT]TNTTTTATTTTTTAAAAAGTGTTTATTTTGAGAGAGAGTGTGTGTTGGAAGGGCAGAGGTGGGNGACAGAGACAGAGGATCTGAAGCTGGCTCTGCACTGACAGCAGAGAGCCTGACACAGGGCTTGAACTCANGAACCCTGAGATCA

>B2:99504600-99504900|varpos=99504750

GGATACAATGTAGTTTTCTATTCAACAAAGCTCCACAGTAAGAGACAGAATGCAGAAGTACCTTTAAAAAAACAGCAACNATTTTCAAAGAGTTCANNNNNNNNNCACCTAAACCATAATTTCTNTCTNNNNNNNNNNNNNNNTTTTTT[TTTTAA]AGGTAAGCTCTACACCCAATGTATGGGNTTGAACCCATAACCCTGAGATCAAGAGTTGTATGTTCTACCAAATGAGCCAGCCAGGTGCCCCCCTATTTTTGTTTTTTTTAATTGAAGTATAGTTGACACACAATGTTACATTAGTTTCAG

>B2:102679995-102680295|varpos=102680145

GACTCCCACCTTGACACCCAATAAAAGCAGAACCCCAGACCCCTGCATGCACCGTGCTCTCTTCCTCTTTCTACCCATGGCCTTGCTGTGTGGCCCCAAATGTGCTGTGTAATTTCCAGGTCCTCTGAGTCATAAACNNNNNNNNNNNN[ATATATTTTTTTT]TTTTTTAAGTTTCCTGATAGTTATTGCTGAANGGCACCTAGTAATGATCAGAACCATAAGGACCGCTCTGGCCACAACACTGGTTATTGACAGGCTGGAACCAATACACAACTGGCATAGTTGGCAGGATTGCATGATTGCCCTTGCAA

>B2:106604590-106604890|varpos=106604740

CAGCAACACACAAGACAGGAAGATCAAGATGAAATTGTCAAAAGCACATAAGAGGAGTGAGCATCCAATGACAGAAAGCCATGTGTTGAGTACAAGAGCTCCCAAATTCTTCACCACAGTGTATAAAGGAAATGACNCTTTTGGTCTGN[GTTTTTTTTTTTTT]TTTTTTTCTTTTTCCCATACCTCTGTTCATCCCTATTTCTCTTCCCAATGTTTCATTNTCCCATATTTTCTCTTGGTTTCCTGCTTGNGCTCTCTGGATGCTCCTTACCAAGANTCAGAGAAGTCNACACACCCTCCTCTCAACAGCA

>B2:109087071-109087371|varpos=109087221

GGCTGGACAACTGTCTTCTGTGCTGTTGTGTCAAGCTTGCTGCCCTTGATCACAAATNTCTGCCAATACTCTCCTACTGGATACTGTTTATGCTATTTGTTGACCACAAGCTGATTACACATAATCACTAAGTCTGGGTTGTTGTTGTT[GTTGTTGTTGTTTTTT]TTAAGCCCTGTTCACTGATGAGAAGCTNNNNNNNNNNNNNNNNNNNNNTTTTTAACTTAAAAGTTTCATGTTGGTTACTAAANTNTGAGCTCCAGTAATTACCTCCAGGTAAATCANCTATCACCAAGAAATTTTCCCTATTCAATTT

>B2:110193009-110193309|varpos=110193159

AGTAGCCAAAACACTGAANATNGGAAGAGATCTCTAAGACCNTAGACACTGAACCAATAGGATGTTAAAGGTGGGTTTACTGAGCTGACTGATGTAAGAATGAGTTAGGAAGAAAAAAACAAAACNGTATATGGAAAGGGAGGTTTNGT[TAAAAAAAAAAAAAAAAAAAA]AAANNNNNCTGTTTTAGTTTCATATTGTTGATTAANCATATTATAGTACTATAATTCAAGCATTCCTCTGCTTCTGTGTGCTTTAAAACTCATCANTAGGTTGTGTGGGATAAGAAAATCATGTAGAGACCAGTCACAGCATCCTGGAG

>B2:110386941-110387241|varpos=110387091

ACAATTTGGAGTGGGNGTAGCACTATCCGTATANACTACTTATATGGCANTCCCCTGGAGTANGCACTAGGCAGATTTACATTTGCCATGTTNGTGATAANTGNNNNNNNNNNGAGAAATAACACATCCATCATGGAATTGAAAAAAAA[AATTTTTTTTT]NNNNNACNTTTATTTAATCCTATCTTCCCTTGTCTAAGTAGAAAATAAGTCTAAAGCTGATGTGTACCTTTCCTATTTGTATTTTATACTGTTTTAAAAATATTACTTTTAAAAGATAATAAGTAGAAGGACAGAAGTCTATGAATAGTA

>B2:110857261-110857561|varpos=110857411

GAGAGGGAGAGAGAGAGACTCCCAAGCAGGTTCCACGGTTCCACACTGTCAGCAAAGAGCCCACCATGGGTCTCCATCTCAGGAACCATGGGAGCATGACCTCAGCCACTTAACCAACTGAGCCACCCAGGTGCCCAGGCAGATGANNN[TTTAAAAAAAAAA]AAAAAAAAATTGATATNAGTTAATGTGGAAACTGATTTTCTTAAAGCTGTGGTCTGTATCCACATCCACTGGAAAGTTTTAGAGTAATCTGGAAACATTATATAGTTTGAAGACCTCTGCTCTGAGCCTTGAGGATCAGATGAACAAAGG

>B2:111221658-111221958|varpos=111221808

CTTTATTNNGTCTTCATTTAAAAATTAAACTTATTTTTNNNNATTCTCTCTTAAAATGAATTGAACACAATAGTCCAGGCATTAATTATACCATTGGTACAAGCATTTCACAGGATCAGAATGTTCCATTTCTAGTAATGCAGCCTAAGAATTA[GCTTTTT]TTCTTTTTTTTAATAAACATGGTATCATATTGACCCTGTGGTCTTCCAAAGTCCTAAATATTTTCAAGATGTACTTCTCTCAAGCCAGTCTCCCTTTATAACATTCTACTAACATAANNNGTTCTGTACATATGGTTTAATGGAGAAAATGGTACATT

>B2:111711768-111712068|varpos=111711918

GTTTGTGGGTTCGTGCCCCTTTTTGGGCTCTGTGCTGACAGCTCAGAGCCTGGAGCCTGCTTCACATTCTGTGTCTCCCTCTCTCTCTGCCCCTGCCCCGCTCACACTCTGTCTCTCAAAAATAAATAAACATTAAAAAGATTTTTTTN[TTAAAAAAA]AAATTACACTTCTCTCCCTATGTTTCCTATAACTTTACACACCAACTTGTTCAAAACATCCACATAGGATGTCTTTCTTGACTNNNNNNACTTATCCCCAAACTCCATCATGTTAAGCTTGAGGGAACATCTAACTTGACCATTCAGGTT

>B2:111786356-111786656|varpos=111786506

ACATAATTTAGGGAAATAAAGGTGAGCCTTAGAAGCTCTGTTTAATGNTTATTGGTCACAAACTTCCTTTTTTATTAAAATTAGCTTGTAATAAAAAANNNNGAGAAGCCACTCTATAGCACAGATTAATTCCCCTCAATTAATCTTNNNTTTTT[TTAAA]ATTTAATGCAAATTATTGGAGGAACCAAATAAAAGGAAGAACTTTCTGGAATTNCCAAGGGNAAATGAATTTGAAGGTCTCAGTAAAGGAGGTCACGGTGAGGAGCAATGGCTGGTAGCAGTGAGGGAGAAATTGTGATATGAGAAAATA

>B2:112408606-112408906|varpos=112408756

GACATCAAAGGAGGCCTTAATGGGAAAGAAAATCAGGGAAGGACTCCAGTATAAGAATAGAATAGAATAGAATAAATAGACATGGGGAACTCTTTAATCAAGGTTTATAATTTCAAATANCATAAACTGACCATGATTTTANNNAAAAA[AAAAAGA]GAGTTTATTGAAAAGTGAAAGGGTTACTCACAATTCTCTGAGTTAAAGAACAGGGGGAAATGAAGGCAAGGAACCATGGCCAAGCCATTCCAGAAAATCAGCCCAGGGAAGTCACAGCTGTNTNNNAAATGCTGAATGCCATGGGTTTC

>B2:113002951-113003251|varpos=113003101

GTGAAGAAATAGTACTTCACCTTCTACTAACAAGCTAGAATTAATTACCTNAAACAAATGATTAAAAGCAANGGCTCTGTGATGTCTTCTTTGGAGAGGTCAATATTTTGAGGAGGTGATAACATTTAATATCCTAAGATCTTTTTTTT[TTTAAA]AATATGCTTATTTCAGTTTATAACAAGTTAANNNNNNNNNGATGAGCTACTCAGTATTTGAATATTGGGTCTGTCAAACTACTATATTATTAAACTGGGAAGAAATGAGGTGAGCAGATAGATGGTCCTTACTCAAAATTCAAATTTTAT

>B2:113205509-113205809|varpos=113205659

ATATATTGCTTTGTTGCAAATAACTGAATATGCAATAGGCAGATCAATGTGTTTTCACATTTCATTATACAATATTACCTTTTTCCTTTAGGGAAACTGGAAGCTTCACTTCTNTCCCCTTAATAGGCTCTGGAAAAANAAAACAANNA[AAAAAACCAAAA]AACATTGAGCCTTTATTTGAAGCCAAGATAAAAGGATTTGAGAACACATATATAATGGAGTCAGTGAGCTATAAGTAAGGTGAACTTGGGTGGAACTTATATAATGTTTTCTTACAAAAATGATTTTAATCATACAGAGACAAGGAAA

>B2:113450548-113450848|varpos=113450698

ATCATGTTTATTTCTCCNTTACCANCTCCTATATCCACTACCATCAAAGGCNATCCGTTTGTTTGTCAAGTTGTTTTTTAATACCTTTTTCCTTTATTCAAGTTCATATGTCAGTTCTGAATGTAAGCAAAAATATCTCCTCTGGGTGG[TGGTGGGGGGGG]GNNNGGGAGCAAAGTCAATGATAAAGTAGCACAAATGGANAAAATCCTTTTTGGATATCCTGGCTTAATTGTATAACTGGCTGTTCTATTGAGGCCATTCATTTTTATTTGTGAAGGGTCACAGAAGATTTAAGAGAAAATCTTGTTATA

>B2:114265902-114266202|varpos=114266052

TGGCATGCAGTGGTATTTAATAACTTTCATACAGTTAGTTTATATAGAAGGAATACAAGAACACATTAATAAATTAGGAGCTGCACTAGAATTTTGACTAGAGACCTCAAATTCTGTCTCATAAAAATAGATTTGTAGGACCCTCAAAT[TAAAAAAAAAAAAA]NNNNGTTAATTAGTACCTGCTACATAGTATGAATTACCTGCATTATTTGNTGTATTTTTGACAAAAATTAATGTTCTCCAGATTGGCAATATTCTACATATTTTTGCCAAATTCATGTTTAACTAGCTTGAATAGCTCAAAATCAC

>B2:114266905-114267205|varpos=114267055

AAAAAGCAAGATGTAGAGCAACACTANGTAGAATTGTCCCTTCTGTGTGAATAAAACTTTTGAGAGGGATATGNNNCCTTGTTTANATTTCCTGGGGAAAGGAATTAGGAGCTGGAAGGGAGAAGATGCCACTCACTTCTTTTTTTTTT[TTTCC]CTTTCTTTACTATATGAGTTTTACTTTTTTNAATTTTTTAATGTTTATTTANTTTTGAGAGAGAGTGAGAAAGAGACAGAGCGCAAGCAGGGGAGGGGCAGAGAGAGAGAGAGNNNGAGNNACAGAATCTGAAGCAGGCTCCAGGCTCTG

>B2:114995503-114995803|varpos=114995653

TATTCTGCCATGTTCCAGTGAANNNNNNCATCCCTTCCAACCATTCTAATCACNTAGCCTTCCAATCATTCTTCGAACCATGTCAACCACACCATATTTTTAGCACNCCTCTTTTTGATNAATACATTGCATNGCCTNATTTAAAACCT[TAAAAAAAAAAAAAAA]NNNNNGACAACATATACTTATTACTTCTTGGTGTCCTCTCAACCAGCCATTTTNTTATTAGAGATTAGGAGGCCCAACNCCATAATTCAAATAGAATTGTATGCAAACAGCAATCATAGTTTTGGGGACAAAGTGACAAATGCTTGG

>B2:115507185-115507485|varpos=115507335

AACTTGCATAAGTTCACTTAACTGAATTCTCTGTTTCCATCTTTTAATAAGGTAGTGTCAGACATTTTCAAGGGCCTTAGTGAAATCTTGATATACTGTACCTTGTGTCATGCCCATTGACATGCCAAATCTAGCAATACTGTNNNNNN[AAAAAAAAGAGAGA]GAGAATTATGTTAGTTTTCACTCATTTTCGTATACCCATGGTGGTTCCCNGCAGTCAACAATTTATCTTCAATTAATCACAGAAGATTTGTCTAATATCTGTCAGTTTATTTTTCAGGGAGTCGTTATCAAGCTGCTCAGTGTACTATT

>B2:115974403-115974703|varpos=115974553

CTGCTTTTTAGTTTCTTTACATGTTCCCTAATTTTGCATTAGAAATTTATGTACAGAGGTAGATGGGTAGAAAGAATAGAAGCTTGGGTTTTGGAGGATATTACAAGGATACTGTTTTCATTACCTGCTTGTAGTNAAATGCAAAAAAA[AAATTTTTTTTTT]TTTTTTNAAATACATCTTTATGGGGGCTCCTATCTGGCTCAGTCAGTAGAGCNTGTGACTCTTAATCTCAGGGTCATNAGTTCAAGTGTCTCATTGGGTGCAGACTCTACTTAAAAANNNNNNNCNCAAAAACNACATCTTTATGTTTGG

>B2:116532051-116532351|varpos=116532201

AAAATCTATTTTGTCTGACATAAGTATAACTATGCCAGCTTTCTTTTTGTTTTAATTTGCACGAAGTATCTTTTTCCATCCCTGCACTTTCAGTCTATGTGTGTCCTTACTTCTGAAATGAGTCTCCAGTAAGCAGCATATAGATNAGT[CTTGTTGTTGTTGTTGTTGTTGTTGTTGTTGTTG]NNNCTGTTTTAAATCTATTCAACCATTCTACATCTTTTGATGTGTTATTTGTTCCATTTACATTTAAAGTAATTATTGATAGATATGTATTTATTGCCATTTTGTTGTTTTCTGGNTGTTTTCTGG

>B2:117431100-117431400|varpos=117431250

CATTNAGTGAGAAAATCATAANCTAAGGAAATTCTTTGAGCAAGAAGAGAAGTTCTGATTAAGGTTCAGTCANAGGAAAGGACAGTGGAGCCCCTGAAATAAAATTACCCCAAGAGACAGAAGCAGTGTGTTCGGAATGGAGGAAAAGN[GGGAAAAAAA]AAAAAATAAANTTTTTGATCCATGTTCTANNNNNNNTGGGAGATCTGACTCAGAACAGACTAGCACTCTGGGGAAATACATGCATCTAAAATNACNTAACCCCNAAAGCCCTGGCTGGGTTTGCATGGAGCTCTCTTGACACAGTGTCTC

>B2:117550472-117550772|varpos=117550622

TTGGGACAATTTCATTAGATTTGTAATCCATTATAGAAATATTACATCTTTATTNAACTGTAGTTTTTCAAAAACAAAAGAATATCTTTGTTTCCCAAACAGGTTGAAAAATCTATTAGCTATTAGTTTTTTAGTAACNATCTCTATAT[TAAAAAAAAAAAAAAA]GAATTTACAATGATAAATCCTTCAACAAAGCACCNGGAAATACTGGTGCCTAAAGGAGGTTAGTTCCAAGCCANGAACCACTGATTTAAGCTTTATTAAAACTGTACTTGTTCCCAGCTTGTTTTCACTTAAGATATGCTTTC

>B2:118097002-118097302|varpos=118097152

GGAAATATAACAGCNATATATGCATATGTGCCCANTAACAGACCTTTAAAATTCTTGNAAAAGCACCTGACAGAACCAATNAGAGCAATAGACATTTGCTCATGATATTTCACTCATTAAGATAGAANAANTAGACAAAAANNNNNNNN[AAATT]TCAGTAAGGATATAGAAGATCAGAATAACACCANATACCTTGAGCTAACTGACNCCTAGNAAGTGCANAATATACATCCTTAAGTGCACGTGNAACATTTACTAATATAGTTGATGTGTTANATCCTAAAATAAGTCTGAATAAACCNTCAA

>B2:118421518-118421818|varpos=118421668

TGTGAGATCATTACCTGAGTCAAAGTTGGACACTTAACTGATTGAGGCACCCAGGAGTCCCATATTTGTTTTAATTTGAAAGATTTACTACCTAGGTAGACAACAAAGTCAGAATTCCTGGACACCTCCATTTAGATGACTCTTNNNNN[AAAATTTTTTTTTT]TTTTTTTTCAATGTTTGTTTATTTTTGAGAGAGAGACNNNNNNNNNNNNGGGGAGGGACAGAGAGAGAAGTTGACAGAATCAGAAGCAGGCTGCAGGCTCTCCACTGTCAGCACAGAGCCCAATGCACGGCTCAAACCCAATAATTGNGA

>B2:118453376-118453676|varpos=118453526

ATAGCTAGTAAGTGACAATCNAGATTGGAATCTNTGCTCCTTCCNAATGATCATCCTTNTCCTTGAAGTACTTTTCAACTGAAACTGCTTCCTGGTTTGTTTCTTAAGTTTTCTGTGAAAACCCATTACCATTTGTAAGTAAAAGCAAT[TAAAAAAAAAAAAAA]AAAAAAAAGGTCCTNTAACNTTTTCTTCTCACAAAGAAAGTTTGCNTAGAGTGGANTTCATTAGTGGATTTAATCTTTAGGGAGGAAAAACATACTTTGTATAAAATGCTAGGATGTCCTTTATTATTTCATGTCACCTGGATTCTAACC

>B2:118457071-118457371|varpos=118457221

ATATTATGCAGTAGAGTAAAGCTCGTTTGAATTTTTAAAAGATCCAGTCTGCTTCTTCAAAGAAATTTCAGAGTTGTGGCTGTTAAATTGNGGCTATGCCACTAGACCCCAGGGTNTATTCAATTTATTCNTTTTGACCTTTAGGGAAG[TTTTGAAAAAAAAAAAAAAGTTTGA]GAAGCACTGAGATAATGCAGNCCTCTGTAGTTGTTCTATTTGTAGACTGGTGGTAAGTTTTTNATACTTATTATGAGCCCAAGANTGATGATGCTGGTTATTTTTATANACCTTATATGTACTAGTCTGTGTTNATCAAATAGGA

>B2:119577880-119578180|varpos=119578030

TAAACACTCTAAGTTGTGTCTGCTCATTTATTGATGGCAGTGATGGCATAAATTGATATTTTTACAAAATTATCATGAATGTCATTNTCACTGTTAGTTGAACACTGTTTCTGATCAGAGGAAAGGAAAGGGTTACTTTGAGTTAAGCA[CTTTTTTTTTTTTTT]ATTTTCAAGTNNTTATTTTTGAGAGAGAGAGAGAGAGAGAGAGAGAGACAGAGTGTGAGCAGGGGAGGGTCAGGGAGACAGGGAGATACAGAATCCGAAGCAGGCTCCAGGCTCTGAGCTGTCAGCACAGAGCCNNACAC

>B2:120288945-120289245|varpos=120289095

ATGAGAAGACTTTAGTTGATANTTCCAGAATANGAGCTCCATAAAATTTTTCCTTTCATTCTCCAAAGAGTGATTTCTTAAATTTTTAAGATTTTTAAATACTTAAAAATACATTTCAANTATTTTTAGAACTTCTACGNNNNNNNCAA[AAAAATTTTT]TTTTNAAGACATCATCTTCTGCTTAAAAGCAACAAAGAGAGATTCAAGCAAGGTAAAGCTACNTGTAATAACACAAGAGGCGATTCTCCATCAACACAGGATTCTCACTGACATTAACNCAGAAAAACTATANCTCAGAATTAAATTGAG

>B2:122195460-122195760|varpos=122195610

TCATTTTCCTTCTCCATGCTAACTACAACCCANTATTTTGCATATATATACANTGATACATTGAAAAATGAAGNNNNNNGNCCCCAATGAACTATTTACTAATGTTGATGTGTTATTTATTNTATTANTTTTTTAATGTTTATTTATCN[TAGAGAGAGAGAGAGAGAGAGAGAGAGAGAG]CATGAGCAGGGGAGGGGCAGAGAGAGAGAGGGAGTCACAGAATCTAAAGCAGGCTCCAGGCTCTGANCTGTCAACACNGAGTCTGACNCGGNGCTNGAACCCATGGNCCACAAGATCATG

>B2:123672404-123672704|varpos=123672554

GATTCNTAAGTCCNCAATCAGGAGAAAACATAAGGGGTATTTTCCGCAGTAAAATGTTTTATACTTTTAAAGTTTATTAAACAATTATAAGGGGCAAAAGCCTATCACNCCCAATAATTCAAGCCATAAAATAGAATATTCGTGANNNN[TATTAAA]AAAAAAAGNATCCAATGAACGAATGAAATGTTAGTGTTTAATAATTTGNCTTGCACTCTTCAATATAGTTGAANTGNAATTATGTAGGAAGAAAGANNNNTTCAATCAGTAAGTATTTTTCAAGTACCAGCCCTCCACTTGGTTTCAGGCAAGAATT

>B2:123814608-123814908|varpos=123814758

AATGGATGCAAAAGTGAATAATCCTCCCAGGCCCCNNGACTCTCCCTTCCCTTCATTCTGCATTCTTTCTTCTACCTCCATGTAGCCCCCAAACAAGTAGGGAACTGAATCACTTAGCTATGCAGTCACATGTTTTTAAAAACNTAAAA[GTTTTTTTTTTT]NNATTATNNNNNNTATTATTCTGATGCANAGGAGGACTCTGAGAGAGAAAGAACGGGTTTGGAGGAGGTTAACCTGCTATGGCTGTCNTTTCAAACAGAACTTNCTAACTGCAAGCCTNGTCCTTAGCAGGACCANNTAGATTTAAAAG

>B2:124681826-124682126|varpos=124681976

TCCACTNCGTGTACCATGCAGCTTCAGAAGGCAGGCTCTTTACCACTTTTGATTTGTAATTTTTATTTTGTTAACCAGATTGTTTCTATNGTGAGAATGAAGATTATTATCTATCTTTAGTAGGCTTTCTTTTTTAGAGCAGTTTTNNN[TTTTAA]NNNNTTAATTTTTTGGGTTTAGTTGACACACAATGTTACATTAGTCTCAGGTTTACAACATAGTGATTCAACATCTCTACATGTTATGCTATGCTCACCCCGAGTGTAGCTACCATCTGTCACCATACAATGTCACTGCAATACCATTGA

>B2:125200607-125200907|varpos=125200757

TACAAATATTAAGAGCATTAAGAAATCATCAGGGGCGCCTGGCTGGCTGGNNCAGTTGGTAGAGTATGTGACTCTTGATCTCAGGGTCATGAATCCGAGCCCCACGTTGGGGGTAGAGATTACTTAAAAACATGTCAAATGTTTAAANN[AAGAGAGAGAGA]AAAAGAGAGAGAGAGNAATCATCAATAAATTACGTGCTAAAATTTTCTTTGGACGGCAGCACAAGTGTGTGTGCAGCTTGCGATGGCCCAGATCTGCACTTTAAGGAGACTCTAGGAAATGTCAGGGCTTTCATTTTGGGTTTTCCCAA

>B2:125454872-125455172|varpos=125455022

TCAAGATAATTCTGCACAGCAAAGGAAACAACCAACAAAACTNAAAGGCAACTGACAGAATGGGAGAATATATTTGCAAATGACATATCAGATAAAGAGTTAGTATCCAAAATCTATAAAGAACTTATCAAACTCAACACCGCCCCCCN[CCAAAAAA]AAAATAATAATCCAGTGAAGAAATGGGCATAAGANNNNNNNNNNNNGACNNTTTTCCAAAGAAGACATTCAGATGGCTAACAGACACATGAAAAGATGCTCAACATCATTCATCATCANGGAAATACAAATCAAAACCACAATGAGANAT

>B2:127416052-127416352|varpos=127416202

CACAAGCAAACCCAGGCTTATAACTGCCCTTCTAAAACACCTTTAAAGACAAATGATCCTATTTAACNAGTAAGATACATAAGGCGACAAAAAAGGCCAACACTTATACCCTGTTTCCTTAAANNTGCCATATTAAATATTTCTNNNNN[AAAATTTTTTTT]TTTTTTTAAATGTTTATTTATTTTNNNNNNNNNNNCAGAGTGCAAGCAGGAGGAGGGCAGAGAGTGNGAGAGAGGGAGACACAGAATCCGAAGCAGGCTCCAGGCTCTGAGCTGTCAGCACAGGGCCTGATGTGGGGCTTGAACTCATGG

>B2:127791098-127791398|varpos=127791248

CCATACGACTATCTTATCCCAGTGCTNCAACATTGTATCCCCAGTGCAAAACCAGTACATGATGCAGAGGGGGTGATCATGAAGCTANAATTTCTAATTCTTTTCCCCCTAGCAGTTAAGCAATTGAGATTTAATTTAAAACCTCNNNN[TTTAAAAAAAAAAA]AAAAAAAAAATTGAAATTGTTTTTTAGTTTTCTAATTGGCTACTCTGTGGACCCTCTAAAAGTTTTNNNCTCCGTATTTGCTAATCTGTGCTGAATAATTCAGCAGGATCTTTATAAAATTACCATTTGCNTTNGAAAACGTTTCACCACCTTC

>B2:128064897-128065197|varpos=128065047

GTTTAGGACAGTGGTACTCAAACTTATTGAATGTCAGAATTNCCCAAATCTGGATNATATTCTAATTGCTAGAACCCCAAAGAAAATCAAATTTCTCATTCAAGAATACTGAGGAAAGAATCCAGAAAGCTGCCATTTCTTAAAAAAAA[AAAAAATTGAT]TGTTTATGTATCTTTGAGACAGANAGACAGAGCATGGGCAGGGAAGGGGCAGAGANAGAGGGAGACACAGGGTCCAAAGCAGGCTCCAGGCTCTAAGTGGTCAGNACAGANCCAGACACGGGGCTCGAACCCACAAACCGTGAGATCAT

>B2:128834123-128834423|varpos=128834273

TTTGCTGTGTTTCTTCATAGTATGTAATGAATATTTTTNNNNCATATCAGACATAGANATTTACATTATGCTTTTAAAATAATTACATAGTATCACTTTTTAAGGTTATATCATAATTTATTTAACCAGTTCCTGAATAATAAACTTTNNNNNN[TTTTTTTCCCCC]CCCCTTGCTATTATCACAGTGTTGGAAGAAAGATTCTGGACATATAAATTCAGGCATTTATGTGAATNTATCTAAAGATAATGTATANAAATGGATTTAATTGGAAGTATTAGGGATATTAATCATTTGTCTCTTCAATATGTTTTAAGT

>B2:128906277-128906577|varpos=128906427

CAGCATACTTAGCTTCAATTTCTCCATACCCTACTGAATGTGTAGTTTGTTATTTTTGGAAGAGACAGAGCTGTTTTATGATTTTCTCTCTCTTCACCTTCACAAAAGTTCTTTAAGCCACATCTCTGGAACTTGGANTAGAAAAAAAA[AAAACAACAC]CTGCATTTCTGTCTTAATGACACCCCTGCTCCAGGAACTGAGTGCTCAGTGGGAGAGAGACAGTAAGTAGCATGGAGTCTTATCAGCTTGCCTTTACAGCTATGGAACCATTNCTTTATGGACCTACAAAAGAGTGACTGGGGCCCCAA

>B2:128967555-128967855|varpos=128967705

CCTATCTCNAGAGGCAAGAAAGGTCNCAAATACAGAACCTATCCTCACACCTAAAGGAACTAGAAACACAGCAGCAAAGAAAGCCCAAAGGCAGAAGAAAAANNNNNNTAATAAAGATTATAGCAGAAATAAACAATATAGAATAAAAA[AAAAAAACAAAC]CAAAACAAAACAGTAGAACAGATAAATGAATCTAAGAGCTGGGTTTTTTTGAAAGAANAAACAAAATTGATAAATCACCTAGCCANACTTCTCAAAAAGAGAGAGGACCCAAATAGATAAAATCACAAATGATAGAGGAGAGATCACAAC

>B2:128995687-128995987|varpos=128995837

GCTCGTGAGTTTGAGCCCCGNGTCGGGCTCTGCNCAGACAGCTCAGAGCCTGGAGCCTGCTTCAGATTCTGTGTCNNNNNNTCTNTNTACCCCTGCTCCGNTCATGCTCTGTCTCTNTCTGTCTCAAAATTAAATAAACATTAAAAAAT[TAAAAAAAAAAAAAA]GAAATTACTGGAATGTANCAGCTGGATGAGGTCAGGGAGAAANATANTGAAATACAGGCCTGCCAGAACCACTGCCNATACCAGAGCGACTGTGGGCAAACCCAATGCCCCTNTNTTAGTCTGCTAGGGCTATTGTAAC

>B2:129600775-129601075|varpos=129600925

GTTCATGGGTTCAAGCCNTGCATCGGGCTCTGTCCTGAGNGCTCAGAGCCTGGAGCCTGCTTCAGATTCTGTGTTTCCCTCTCTCTCTGCTCCTCTCCCACTCATGCTCTGTCTCTCTCTGACTCTCAAAAATAAATAAATGTTAAAAN[AAAATTTTTTT]TTTTTAAAAGAAAAGTAAAGATGGTCAAGAATTGGGCATTCCTAACTTTAGATTCAATAAGATGAAAAACAAGCTAAGAAGGTTAGGAAGGAATAATGAACCAAGAAAGANTGATGTCCTGACACTACATGAACTGTANGAAGAGGTATT

>B2:130757186-130757486|varpos=130757336

ATNTGTATAGCAAAGGAAACTATGAATAAACTAAAAGACAACCTATGGAATGGGAGAAGGTATTTGCAAATGATATACCCGATAAANNNNNNNNATCCAAAATATACAAAGAACTAATACAACTCAAAACAAAACAAAACAANNNNAAA[AAAAAAAACCC]CCACAAATAATCCAATTAAATAATAGGCAGAAGACATGAACAGATATTTCTCCAAGGAAGACATCCAGATGGCCAACAGACACCAGGAAAGATGCTCAACATCACTAANCATCAGGGAAATACAAATCAAAACCACAATGAGACATCACC

>B2:131947880-131948180|varpos=131948030

ATCNCCATCCTCAGTAAGTAACCATTGTTATGTGTATCTAGTATATCCTTCCAGAGACTTTTTTTAGTGGAGGTATATAGGCAAACACAAATATATATGATATTTTACTCCTTTTAAAATGTAGATGGTAGCAAAGTGCTTTTTTTTTT[TTTTTA]ATNTATCATGGAGTACATGNAAGAGCTTCCTCATTCTCTCTTTTTGGTCTTGTTTTACAGCTGTATAATATTTCATTGTATGGATATATCGTTCTCTAACCACCCTCTTCTTGTCAAATGCCTTCTATACCCAGAAATTACATTTAAATT

>B2:133014476-133014776|varpos=133014626

TATNTTTTGTATTAAGTATTTTCAGGTTTCCATATAAATAAGAGATCATATATTTGCAATTTAGAAAGAAATCAACATCTAATTAGAACTTACATATGTTTATGTTTTTCCTCCATTTACTGATTTGTATGTAAAATTATTTTTCATNN[CTTTTTTCTATTTTTTTT]TCCTTTTCTGATTTTTCTNCTTTGGCATNTTCTCCTTATGTTTGTTTCATGGACTGAGACTTTGTGGTCTAATATAAATCACAAAACCATCAGAGAAGCAAGATTTAGTAAGGACTGTGTGTAATTCAACAAGCTGTACATCTG

>B2:135204091-135204391|varpos=135204241

CAGCCACAGAAACCATTTCCACAGCTGCGGTCCCTGAGATGTCAACTGACNTAGTTTTTGCCAAGANNNNNATCTTTCAATGAAGGAGCTATCTCTAGTGTGAAAATATNCTTTTGAAAATACAGTAGGATAGTTCGTATGTCTATTTN[TCCCCCCC]CCCCCTTTTTTCCTGTGGAGGTCTTAGAAATGAACAGTTAAACTATGTTCAACCCTCTGGTGTTTTTGCCCTCCCAGGCNGCTTCTTTAAGCAAAGGCTNTGAAAGGATTATCATTTTTGCCTGTTTAATATCTAATTACTCTCTGTGAG

>B2:135290178-135290478|varpos=135290328

CATCAGTGCCATTCCAAGCTATATTGGAGTTTTACACAAAAATGACAATAAGTGATCTAGGTTGCCTTTATTTGAAAATTTGACATTTAGCTCGCTATGGATTTTTTTCATTAGCTAAATCCATACTTTATTCACATTTCTTNGTTTTT[TTTTTTATTTTT]TTTTAACGTTTATTCATTTTTGAGAGAGAGAGACAGAGACAGAGTGCAAGCAGGGGAGGGGCAGAGAGAGAGGGTGACACAGAATCGGAAGCAGGCTTCAGGCCCCAAGCTGTGGGCACAGAGCCCGATGTGGGACTCGAACTCAC

>B2:137817225-137817525|varpos=137817375

CAGTTCACATAAAGGTTATGAACCCCCCACGGTTGTTACTCTGAAACAATAATAGATTCTTTTTTTAAATTATTATTTTGGAAAACAGCNCTGCNGGANATCAATAGAGTATCCAAGCCCTTCCTACAAGTTCAAGTACATTTTTTTTT[TTTTAAA]AAGAGTGTTTTCCAAAGTATAGAGTGTGTTGAATGCCTTTCGTTATGAATGGATTAGATGTGTGTTATCTCCTGGAGAAAAATGTNTCTGGAAACATACTGCATTCTTAGAGGGATAATTAGTTCATTGTGCTTAGCACAGAGAGTCCTC

>B2:138561699-138561999|varpos=138561849

TCAGGTCATGGTCTCATGGTTGGTGAGTTTGAGCCCTGTGTCAGGCTCCATGCTGACAGTGTGGAGCCTGCTTGGATTCNCTCTCTCTTCCTCTTTCTTTGTCCTTCTCTCACTTGTACTTTCTCTCTCTCTAAATAAATAAATAAACT[TAACAACAACAAA]AAAAANAAATTCAAGTAANNNAAAAAGGTGGTTGAGTTAAAAAAAACAATTACCATAGGTGATACGTGGATATGGCAAAGATTGTGGGGGTGGAATGGGAATAAATGAGCTATAGACAAAGAGATTGGAGGAGGAGATAACACTAGTG

>B2:139174196-139174496|varpos=139174346

ACAGAATAAAGGATTGTTCANAGACCAAAATGAGCTCAGGGAAAATTAAACACATGAGAGCAGAAATACAAAACTCCCATAGAAANGTATAGAGTTGAAATCTAGGNAAGTCTTCCAAAATATAGAGCAAAATATCTCTTAACANCAACAN[CAAAAAAAAAAAA]AANNTTAAAAGANTGGACCAGAATATCCAACATCTGCATAAAAAGAGTTTCAGAAAGGGAGACAGATCAAATGGAGAAATAAAAAATATTAAATCAATCATTAGACAACTNTCCAGANGGAAAGAGGAAAGCTGTTAAATCAAAACNG

>B2:139329066-139329366|varpos=139329216

CCTTTGNTGGAGCACTTTTATTTTTTAAATGANAAAGTAATNTTTCCCCCATCTATACTTATCTGAATAGNTTGGTTANGTAAAAGCAAACAAAACTCTGTTTTCTTACCTTATATGCTAACCATTTNNNAAAAAGAATGATATAATTT[ATTTTTTTTTTTTTT]TTTTAGAAAACCCAACCTTTTGCTAATTATCTGTGGCAAATCTCTCCATCTCTCATCCAACTGCTCCAAATGTAGTTTCATCATTTTCACATCATCTTTGGAGGCCACTGAAAATAGTGATTCCTTCAATTGCAAAAGGTTGCTAT

>B2:139369642-139369942|varpos=139369792

GTTTGAAAGTCTTNACTCTTTGAAATGGTCCGTTNTAAAGAAAATTATTTTCTCACAAATTCCAAAGGGATAAGAGAAGCTCTAAGGGGTGTGAGATGAAAGGAAAACAATATGTGTCCATCTTTGTTTCTTACAAAAGCCCCTATTAT[CTTATTATTATTATTATT]TTGTTATTATTATTATTATTATTATTATTATTATTACTAAAAATTTGGGAAAAACTAAGTCTCCTAGAAATTAAGTTATTTATAAAGTCACATGGGTAGTAAGTAGCTGAANCATGATTCAACACATTCCCATGTAACATCCATTTAGTA

>B2:139865977-139866277|varpos=139866127

CACACACTTAAACCTCTCAGAGGTAAAATAATCAGCTGTTAAAAATCTGTCCACTAGATGGCTGTGTTGCCTTTATAAATATAATTTTTAATCAAATCTGAATAAAAGAAAATGAAAAAAGTTTCTGGGAGTAATTACCCCTTATTTGG[GAAAAAAAAAAAAAA]GGAGTTGATGGAGTTTTGATAATTATATATTTCTAAGGAATTGGANAAGAGTCATTCAAGTTTTCTTCTNNNNGGGAGTGAAATAATCAAACTTGAAGGGGATAAATTTAATCTCGGGAGGTGTGCATAATGGCACCAAGTTTTCCTTTT

>B2:140353096-140353396|varpos=140353246

TGGCATTAATGTCATTCTGTCACCATTACTAACATGTAGTAGGAATATATGCTCTGTCAACCTACAAATAGGGGTTGAATATGCATACCTGTTTTAACANGGAGAATGTGGTATGTATTATANTCCAAGGACTTACCTTCCAAGATGAA[CTTTTTTTTTTTTTTT]ACTTTAAGCAGTTTGTATGTTGCCTTCTACCCTCATCACCATGAGACAATAGAGATAAAAGTCTAAAATAGAGACAAAGTGTNTCTTGAATAACTGAAATTTCTAATGAAGATTAGAATAGAAAATGTCTAAGTGCAATGAAAA

>B2:140508975-140509275|varpos=140509125

AGGGAATATCTTTTATAAAGTTNATACTTTAAAAATTCAGCTGCTGTATTTTCATCCATGNATATTTCTCCAAAAACAGATATCTACATTATTTTTTTTNNNNNNNNNCTTCCTGTGGTCCTNAAATTAGAACTTCATGCTTCATTATCATTTCTTT[CTTTTTTTTTTTTTT]AATTCTTCACGTTGGGGTCATTTTAATTACATTATNATAATCAGCTTCACCAAAATCTATTTGAATACAGAGCTGATNCTACGAATATGTGGCTGGAATACCCAAAAAAGCAGGATGGATCTTGGAAGAAGGTGGCCATGTT

>B2:143183266-143183566|varpos=143183416

ACCTGGGTGGCTCAGTTCGGCATCTGACTCTTGATTTTGCCTCAAGTCATGATCTCACAGTTTATGGGATTGAGCCCCACGTCGGGCNCCTTGCTGAGCATGGAACCTACTTAGGATTCTCCATCTGTGTCTGCCCCTTNCCTGCTTGT[GCACACACACACACACACACACAC]ACACACACACACNCACTCANAATACATAAATGTTTANNNNNNNNNNNNNCTCCANGCTTGGATGCTTATATNAAAGACTTCCAAGTGTTAAGGCCTCCCTNNNNNNNGAGTGTGCCACANGTGCTNCCTTGTGCAGCTGNCATTAACAG

>B2:144646806-144647106|varpos=144646956

AGTTTTATCCATTGTGTAAGATGCTTAGAGGAAAATAATATACTCATTTTCTAATGAATCAAACAAAGCCCTCATCACAGGTATAGTTTTTCTGCACAAGTATAGATAGAGATATTATTGTTTAACAAAAGAGTCACCTGAAAAATAAA[GTTGTTTTTTTTTTTTT]TTTTTTTTTAAATAGGTTTCTGACATCATCAAAGTAAATGAACTCNNNNTTATGGTCAGCCAAGTNNNNNAATTAAAATCTTACTATGAACTATATAGCACAGACTGGACTGAGTTCTGTGATGATGGCAAAGGTGACACTTCCCGCCC

>B2:144892986-144893286|varpos=144893136

AGGCTCAGGTCATGATCTCNCAGTTCGTGTGTTCAAGCCCCACGTTGGGCTCTTTGCTGTCAGCACAGAGCCNGCTTTGGATCCTCTGACCCCTNGCTCTCTGCCCCTCCCNCATACTCTCTCTCAAAATAAATAAATAAAGTTAAAAN[AAAAAAAACAACAACAAAA]GAACAATNCAAGTTTAGANGTTTTGGCTTTGAGAAACATGAGAAGGCCAANTTAATAAAAGTTAAATTCTTANGTTATCGCCGTGGCANAAAGTGNAAGGGTCTGTGTGGAACACTCGGTGTTTCCGAAATAATGCCGCGGGCGAA

>B2:147012736-147013036|varpos=147012886

TCTCTTGCCTGGGTGGTAGACTATGGCATCTATATCCAGTGCAACCCTCAACCCCCTGANATTAAAAAAAAANTNNNNNTTATCATTTTATTCACCAAGAGAAAATAGAAAACACTGTCAATTAGNCACGANATACATACTTTTTAACT[TGAGAGAGAGAGAGAGAGAGAGAGAGAGA]ATGAGAGCAGGGAAGAGGGGGCAGAGGNNNNNNNNNNNNNNATCCCAAGCAGGTTCCATGCTCAGCTCGGAGCCTGACACAGGGCTCNATCNCACGACCCTGGGATNGTGANCTGGGCCNAAACCAAGAGTTGGAT

>B2:147563305-147563605|varpos=147563455

ATTGCTTGGCAAACACCAAGGAATATCTAATGGAATTAGTGNGCNATTAGGAATCAAAACTAAAGAAATTAANNTTTCCATAAAATAATCAGCNTCCTGGATAAAATGAGTTTTGATTTACTTACCAAATCTTAAGTACAAAGCTAAGAGGGCGTTTTT[TTTCCCCCC]CCCCCAAACAAGTAAAATTAAGACAAATAAGATGATGTGGGGCACCTGGGTGGGTCAGTNNNTTAAGCGTCTGACTTCAGTTCAGGTCATGATCTCATGGCTCATGGGTTCAAGCCNCGCATTGGGCTCTGTGCTGACAGCTCAGAGCCC

>B2:147817529-147817829|varpos=147817679

TTACTAAGGGACATGCAGTTACTTGGTCCAGCATGGATCAGGCCAGTTCCAGAGTCTAGTGCCCTCGCCACTATTCAAAAATGGCCAATGGAGCTTGTCCAAAGATGGAAGAAAAATAAAATGGAGGTAAAGGCAAAGGAAAAAAAAAA[AAATT]TCCGGGGAGATCAATGATCTCCAAACACTTAAAAGGTTTGGTGAACAGAGGTTTCTTCCNATGAAAAGGGNCAGAACAAGCCCTAAGGTGGAAGGAACAAAGACAAGGGCTGAACCAGCCCACACCCACACCTAACAAGAGAGTCTGAGC

>B2:148096431-148096731|varpos=148096581

GCGCCCATCAAAAATAATCCCCAAAGAGGCTCACCAAATCTTCCATACACAATCTAGAGCAAGTCTCTGTCATAACAAATCCCCNAATAACAACNGTATCTAATCATACTGTGCATTAACTCTAATTTCAGAGATTACCGCNNNNNNNN[AAAAAAACCC]CCAAAAACAAAACCCCCAAACCCCAAACCCACAANTTTGGCTTAACAACCCTGTAAGGGAGAAACTTTGCTAACCTTATACATCACAGTGTTTAAAAANNNAAGAACCATTTCCAAAGCTTTANTCACCGAAATGCACCTAGGTCATTTT

>B2:149034472-149034772|varpos=149034622

TCTTTCCAATAGATGGAGAAAAAGGCCATATATATCCTCATACACAAACACACTCATATACATTAATANNNTTTGTCTCCNTCGTATATGCAGACAAGTATCAAATTACNGNGCAAAATCAGCATGAATTTATTAGCTAGAATAAAAAN[AAAAAAACAAAAACTAAAA]ACTTTGGGCAGNTCCTGATGGTTTAGAACTTTGATCCTANATTCCCTCCTTCCCAAGATTTATTGTTTCCTANCTGTGTGAATTAGATAGCAGATAAACCCTTCTGAATGTTAGTAAATGTATTTGTAAGATGAAATTAATAATACT

>B2:149049204-149049504|varpos=149049354

ATTGTTCTTTTTCCACCTGAGCTAACTGANATGGGAATGTTTGTGANTTCCAGCGATTCAGCCACTATAGGAATACTGATAGAAGACTGTTTGAGANTGTTTGAGAATGTTTACCCAGCTCTTCCAGAGCCTTANATAAACAAATATTTGGTAAAAAAN[AAAAACC]CATGGTATTTTGTTGTCTNATTTTAACCTTTTTACCTTAANGAGAAATCAATTTCACATCATCTGTTCGGACCTGTGTGTATTTCCCTGCTCTCAGAATCGTACACAAGAAAGCAGATAGCCTNGAGTGTGTTCCCACTGTGTGGAACAA

>B2:149110278-149110578|varpos=149110428

ACAAAATGTGGAGAAGCCCCGGATGGTGGGNAGTGTTGCAATTAAATGAAGATTGCTCCCTGCCCNCNNCACCATGTAGATGGAAAGNGGGGCCCCTGAATGGTTAAAACATCAGGTNGTGATGCAACAATTTTATAAACTCTCAAGTT[CTTTTTTTTTTTTTT]AACATTTATTTATTTTTTGGGGAGACAGAGAGAGANAGAGCATGAGCATGGGAGGGGCAGAGAGAGAGGGTAGACACAGAATCCNANGCAGNNNNNNGACTCTGAGNTATCATCACAGTGCCCGGTGCNGGGCTNGAACTC

>B2:149223481-149223781|varpos=149223631

AAAGAGATAACAGTATTCTTGCTGATGATATGGGTTCATATCTACAAAATCCAAGATACTCCAGCATGGAAAACTGGAACAATANTGAAACTTTTAAAATGGNTCGCAGTACATTATGGGAACTTGACAAAGGNATAAATTCAGNAAGA[AAGAGAGAGAGAGAGAGAGAGAGAGAGAGAG]NNNNGTATGGCCACAGAGGTACANNNNNNNNNNNNNGTCTAAAATAAAATCATCTTGGGGGTGCCTGGNTGGCTCAGTTGGTTAAGCGTCTGGCTATTGATTTTGGCTCAGGTCATGATCTCATGGTTCGTGGGAT

>B2:149277111-149277411|varpos=149277261

GTTTCTGGGCNTCTCTTAGCTTGGTCAAGCTGACACACAAAATTAACTAGNGTAATGGTGTAGGGAGACNTTTCCCTAGTCTCCAAATGCNTTCTTACACCCTCTTCCCAGNAAAAAANNNNNNNNNTTTCCTGACCAAAAAGGCTTAN[TTGTGTGTGTGTGTGTGTGTGTGTGTGTGTGT]NTGTGTGTGTGTGTGTGTGTGTGTGTGTGTGTGTGTGTGTGTTAAGGAATAAAGAATGTACAGTTGACCCTTGGACATCACAGATCCACAGANCCACTTAGATGTGGNATCTTTTACAGTACGATACTGNNNNTGTATTTTCTCTTCCTTA

>B2:150055308-150055608|varpos=150055458

TATGTTNACTNCTAAGAGTACTGTATATGTAAATATGTAAATAAATATATGATCGTACATATATGTAGTTGGTTAGGCAATTAGTTAGTTACCTTGCANTAGTCANNGATTTCTTAGAAAACACACAAAAANAAATAGCTAGTTTAAAA[AAATTTTTTTTT]TTTTTTAATTAAAAAAAAGAAATAGCTAGAAAACAAAATTTATAAATCGGACTCCAACAACANTATAAAAGAGTTCAAAACACTTTTGCTCGATAAAGGACNCTGGTAAGATAANTAATAGGAAGGCCAGACACTGGAAGAATATATTCT

>B2:151100926-151101226|varpos=151101076

TGATGCTGGCATTTCAAGAAAACCNTAGAGCAAGGTTCACCCCTCTCCCACCCTCCCCTGTTTGCTTCTAGGGGTTANCCGAACCCTTAAGATCTTGCAACTACAAAAATGTCTTTCAAGGTTCGATTATAAGAGCTNCGGAAAGAGTA[CTGTTTGTTTGTTTGTTTGTTTGTTTGTTTGTTTGTTT]GTTTGTTTGTTTGTTTNATCTTTNAGGAACTCTTTGCAAACATAATCCTAAACCACTCCCTCCCCATTCAGTGCAGACTGTGTGCGGGGACTGCGGGTGGGAGGGAGCCCCTGGCCAAACCAAGGAAAATGGAAACATTGCCAAG

>B2:151969413-151969713|varpos=151969563

GGCTCAGTTGCTCAAGCGTCCGACTTCGNCTCAGGTCATGNTCTCACTGCTGGAGTTNGAGCCCCACATCNGGCTCTGTGCTGACAGCCTGGAGCCTGGAGTCTGCTGCGGATTCTGTCTCCGTCTCTCNGCCCCTGNCCTGCTTGCNC[GCTCTCTCTCTCTCTCTCTCT]GTCTCTCTCTCAAATATAAAATAANACATNNNNNNNNTNNNNNNNNNNCCCTCCCACAAAGAAANCACAAAGCCNGGATATTTTGTAGTTGAGTTCTACAAAACATCCCCAGAATACCTAANTTCGATATTACATGAAGA

>B3:3486726-3487026|varpos=3486876

GTCTCTCAGGCAGATGGTCTCAGGTAGAAAACAGCAACTCTGAGAGCACNACTCCTNCCAGACAGATTTGGCAAGGACAGCTTTCTTTCCTTGCGNATATTTCAAACCACAGATTTCTCTGCAAGAGTNGAAGGAGGTTATTTACTTCN[GAAAAAAAAAAAA]CGATCAACCCCTGAGTCTCCCAGAACNTTCCAGCACACAGCTCTAGGATATGAAGTTCTGCCGTCAGAATGCTCTATTGTGCTCTTTCAAANGTGTGATTCAAGAAAAATGTTGCACTGTTCCCTTTTTCCAGCCTGAGATT

>B3:3693821-3694121|varpos=3693971

CTCTGGGGTGACCCCAGGNNCCTNATAGAAGCCTCNCAGGCCANAGGGAACATGNGAAGCAGCCNGGCGGGCAAGTACCGTCACCAGAGTCCATCCCNCTTCTACCTGGTTGTATATATTAGGGCTTAGTATTTTGTTTTGTGTTCTGC[TGAGAGAGAGAGAGAGAGAGAGA]AAGNCTTTCTTCTTTGAAAATCATGACAGTAGGTGATCTCTCAAGNCCTTCCAGCACTTTCTCTGACTCCTGCCNTATGGACATTTGTGNGGAGACGTGTCCCANTGTCACNCNNNGTGCCACCCAGCCCCGCTTTTCAA

>B3:3935159-3935459|varpos=3935309

CAGTGAATGGTTTGTGATAACTCAATTTGTCTAACATTACCAAAGGAAGCACTTTCACAAGTAATCTNCCCAACTGATTNNNNNNNNNNNNNGGTACAAGTTGCCTCAAGCTGGGAGTATATTTCCCACATTTCCTANNAAAAAAAAAA[AAAATT]TCCCTTTCACTATAAACAGTGTTAATTAAAAGAANTCAAAGGCCAGGGGTACAGATTTCCATACACAGCCAAATCTTNNNNGGGTTAAATGAATCAAANGGAATAGGTCTGGGAAAATTTTAAAGGTGTTAAAATAACTGTATTTGTTTT

>B3:4257696-4257996|varpos=4257846

NTGGTAGNATTCACCAGTGAAACCGTTTGGTCATGGACTTNCTTTGTGGGAAGTTTTTGATCATTTGTGTCAATTTTTGCAGTTTGTGTTTTTGTAGGAATTTGTTCATTTCACCTAAGTTTTCTAATTTGTAGGCATACAATCTNNNN[CTTTTTTTTTTT]TTTTTTTTAAAGNTTNTTTTGAGAGAGAACACAAGTTGGGGAGAGGGGCAGACAGAGAGGGAGAGTGAATATGCCAAGCAGGCTCTGTGCTGTCAGGACAGAGTCCANCGTAGGGCTCGATCCCTTCAACCATGACACGATGCCCTGAGC

>B3:5564798-5565098|varpos=5564948

GAGCTACTTTTTTGATATCAACTTTGCTCTAGTAAAGAGTTCCCCCNAAACCTCAAATTACGCAATTTAACATTTTCAACCTAAGCACAGGACTTCATATGGATCTCCTTTAATTGTTTAAAAAAANTTTTTTAAGGTTTATTTATTTT[TGAGAGAGAGAGA]CAGAGAGAGAGAGAGAGANAAGAGTGTGAACAGGGCAGGGNCAGAGANAAAGGGGGAGACACAGAATCTGAAGNGGGCTCCAGGCTCCGAGCTGTCAGCACAGAGCCCNACGTGGGGCTCAAACTCATGAACCGGGAGATCATGACCT

>B3:5863303-5863603|varpos=5863453

GATCAACATGATTTTTTTAGGCTTTTTAAAAGTTAGAAATGGCTTACAGATCCTTTTTCATTATCTTCATGGAAACTGAGATGCCTAGGGACCCTAGCTTGAGAACCATTAATTTANNNNNATTTCATCATTTTAGATTTTTTTTTTTT[TTAAA]AAGTAATCCTGNACCCAATGTGGGGCTCNAATTCACAGCCCTGAGATTAAGAGTCTTATGCTCTACTGACTGAGCCAGCCAGGCGCCCCTAATTCCATCATTTTATAGGTGAAATTTTAGGCAGGCTTCCTTTAAGTGACTCGAATAAGT

>B3:8121960-8122260|varpos=8122110

TATTTCTCAGGTTATTAATTTTGAGATCATTCTTTCTTCTTTACCAATATCCCACTAATNTCTGACGACTCTCCCTCATGACCAGGTGACTTGGTCTGTGTCATTTCGTCCATTAGTTCTTTAAAGCCACAATCTCCAATTCTTTTTTT[TTTC]NTTTTTCTTTTTTTGTACAATTCAATGGCTTTTAGTATATTCACAGAGTCATGTAACATCACCACAATTAATTTTATAACGTTTTAATCACCCCCATAAAAACTCCATAACCATTAGAAGACACTTACNTCCAACCTCCATAGCCCTGGG

>B3:8166740-8167040|varpos=8166890

TATACAAAAGCAACNGAATCTGCNTTCTTCTTAAGTNCATATGGNACATTCTGCCAAACACAACAAGTCTCAATAAATTCAAGGAAGATTATATCAAGCATTTTCTGACAACNATGNTNTGAAACTAGAANTCAATTACAAGAAGGCTG[GAAAAAAAAAAAA]GTGCAAGTTCAACAACATACTANTGAAAAACCAATGGTTCCACAAAGAAGGTAAAGTAGAAATAAAAGAAGNACATATAGACAATTGAAAATACAAATAACACACACCAAAATCTATGGGATGAAGCAAAGGTTGTTCTAAGA

>B3:8407185-8407485|varpos=8407335

CATTTTANTGTATGCAACTATGTCTTAATTAANNNNATTGAATTTTAAAAACTCACAAGTGTGGTTAACATAAGAAAGAGTAAAGGGCATCCAGGAGGCTTCAAAGGCATTGGTGATATTTTTATTATCAGATGATATGNTGTGTATTCAGCACAAG[GTTTTTTCTTT]TGTTTTAATATTTTAAGCCTTCTATATGTACACCATATCTGTACTTTGTATGGATGAAATACTTCATAATAAAAAAATAAATTAGCTGTGATCTGGGAGATGATAGTGGCCATGTGTATAACTGACAAATATTAATATACTGACTATAT

>B3:8625881-8626181|varpos=8626031

CCAATGAATGTTCCTGCCAATGTCGTTTTTCAAAGCAAAACAACACTGCANACAACAAACACAATCGGACAACAATCCAAAGGCATAATAGTGGGGGCTGGTTAAATAAATGATGTNATAGCCTTAAAACANGAATACTACACAGCTGATAAAAA[GAAAAAAAAA]TCATTCCCTGTGTGTTGTTATAGAGAAGTCACCAGGATATATTGTTCAACAAAAGAGTGGCTACAAACCATGTACATGGTATGATTCCTTTTTGTATTAGTAATCACTCACACNNNNNNANACACACACACACNCACACTCACATA

>B3:10188951-10189251|varpos=10189101

AATTGGGCTGACNCTTGGTGTTACACATTCAATGCAATGACATGTATCTATTATAATAGTATCATACACAGGTAGTTTCACTACCCTAAAAAATCCTCTGTGCTCTGNGCATTCAGCCTTTCCCTGACTNTGACCCCTGGAAACCACTG[ATTTTTTTT]CTTTTTCTGTTTTTGTAAGGTTTTGCCTTTTCCAGAATGTTATAATAGTTGGGTCGTACAGTATTTATGTAGCCTTTTCACACTGCCTTCTTTCACTTAGTAATTTGCATTTACATTTCTTGCAAGTCTTTTCATGGATTGATGGCT

>B3:11074573-11074873|varpos=11074723

CNCCTCTTAGAAAGATTTGGCCTTCACAAAGGCCTCTGGGCTTGGAATCCGGCTGCTTGGGGNNNTTACATGGAGTAAAACCCAGTATTTCATTGCATGACTTCTCTTGTCCATTCCACTNGAAAGCCCTGATGCTATTATATAAGCCTTTAA[AATATATATATATATATATATA]GGAAAACCAGAAAAGACCGATCTCTTTTTCCTCTTGGCTCTTCTTCCCTCCTCCCCTTTTAGGCACAGAGTACCAAGACATTGAGACTGAGAAAACCTGCCCTGAGTCACATTCACCTTCAGAAGACTC

>B3:11218225-11218525|varpos=11218375

ACAAAAGAAAGGCCTCCACTACCAACCTGTTGAATGGTTATAATGGAAGTAAACAAAACCTTGTCNAAATAGNAAAGATTTTTGCTACTTCAAAANNNTTTTTGCTACTTCTGACATTTTTGCAGTGTCAGAAGCGTGTCTCATTAAACAGCA[TAAAAAAAAAAA]NTAGTAATATGGTAGCTCAAAAAGAAAATGACAATTTTCTANCAACTNCACCCAAAGACCTGGAATATTGTGATATAACCAATAAANAATGTGAAATAACCATTATGAAGAAATTCGACAAGCCAGAAAACTCAGAAGGTAATG

>B3:11800615-11800915|varpos=11800765

TTTCCTTTTGTTGCCTGATTGTTCTAGGTAGGACTTAAAGTACTGTGTTGAGGAGGAGCAGTGAGAGTGGGCACNCTTGACTTGTGCCTGATCTTTTAGGAAAAGCTTTCTGCCTTTTCAGTGCCATTGAATATGATGTTAGCCTTNTTTTT[TTTTTAAAAA]AAAACACCTTTTTTCCCTAAATATTATTTTAAGTCTGCTAAATATGACTTTCTGGTTTCNNNNAAAAATAATTTAAAAAACACNTAGTCTTTCTTTCAAATCTGTAGCTCATTTTGTGTAGGATTGGGGTACTAGTATCTCTTGCCTTGCATTTGATTCTT

>B3:13645629-13645929|varpos=13645779

GGAGTTTCATGGTTTCAAGTGTTACATTTAAGTCTTTCATCCATTTTGAGTTTAATTTTTCTGAGTGATGCAACATATAGGCCCAATTTTATTCTTTTACATATGAATATCCAATCTTCCCAGCACCACTTATTAAAGAGACNNNNNNN[TTCCCCC]CCCCATTGAGTATTCTTGGCTTCCTGGTCAAATATGAGTTGAGTGTATATGTTTGGATTTATTAGTGGGCCTTTGANGCTGTTTTATTGGTCTATTTGTCTGTTTGTATANGTGTATTATACTGTTTTGATGACNACANTNTTATAGTAA

>B3:14315022-14315322|varpos=14315172

CAGAAGGCAACTAAGGAAGGANGCTATCNCATTTCTCATTTTCTTGGGTGTGCNAGCCAGTCCTGCCCCAGACAACTAGGCAAATGGCCTTTTGCCAAGCAGGATTTGATCACCNGGAGGCAACTCCTTGTCCCCACCTGTGACCACNN[ACCACCCCCCCC]CCCCCCAAGGTAATTGTAACAAGCACTTGGATTCCTTACAAGTAAAGGTGTGAGGGTGGGGTTACCCATGTCAACTGGTAAGAGGANNGCAGGGATCATGCTAATCCTTTTACAAGGGATGCAAATCCACAAAGCATCTCAATGCCAGCC

>B3:15114711-15115011|varpos=15114861

TTGCAAGCAGGGCAATCGAAATGACCTGATTCAAAGGATCTAGGCCTATTCTGTATTTGCAGAGATGTCATTTTCCTTTACTCAGTGGACTTGTGAAGGGCAAAAGACCACCTTTTAAAAGAAGTTTATTTCTTTATTTTGAGAGAGAG[AGAGAGAGAGTG]TGAGAGAACATGCACGTGGGGGAGGGGCAAAGAGAGAGGAAGAGAGAGNNCATCCAAAACAGGCTGCACACTGTCAGCACAGAGCNTGATGTGGGGNTCGATCTCTTGAACCCTGACATCATGACCTGAGCTGAAATCAAGAGTTGGAC

>B3:15341621-15341921|varpos=15341771

AAATTTTCCAGAAGATAAGTTGACCATTCATATTGGCTGCACTNAGCATCNGTATGGTTTTTTATTGCNTTTATTTGTGAGGTATATGTATCAAATTTTAAATCACTCATGTAAACCATAACTCCTATCAATTTATTTCCTATTTTTTN[TTTTAAAAAA]AAATATATGTGANTTAAGGGNTTGGTAAAGAATAAAACTTTGTCCAAAAATTTATGAAACTTCAGAGAATTGATTATCTCAGGCAGTTTTAAGTANGGTGATGAGAATTTGAAAGATACAAATGTTGGATTTTAATTTGGTTCTCTTTTG

>B3:15467559-15467859|varpos=15467709

TGAATCATATTAGTGGTATTTGACATCCATAGGCAGATGGCTCTGTTGGATATGAAATACTATTCNTGTTCATTCAAGACTCTGTTGTTAATATCATTGTGATGATAGACACAAGCTTGATAAAGATGTGGAACAAATGCATTTTNTTN[TTAAAAAAAA]AAAAAGCATTCTGAAAATGAGAATGGCCTCAAATCTCAAAGTGTTGCCAGATGTTGTTGTGGACTGTGTGGTGTCTCTTCTCCAAATCCCTCTTCTCATGGCAGAGGGAGTCAAACAGAAGAAGGGCATCCTTCTTCTGAAAGACTCTCT

>B3:16284775-16285075|varpos=16284925

GAAATTAAGACTCTTCAATAAATTGCTTTGTAGAAANATTTTCAATTTAGTAAGCTAAGGTATTTTTTAAGTTNCACACAAATTTAGGATATCATTAAATTTTGGAATTATTTAAAAACAAACCAGACATTTCATTCTTTTCTTTACNT[GCACACACACACACACACACA]ACACACACACANACACACACGTGCACTTCCTACCACGAATTATTAGAAANGCCAATAAANGAGACATTGTAGTATTAGTAGCATAAAATAAAATTNTTTGCTAATTATTCTTGTCTATTTAATTAGAAAGCATTATTTCCTGTTTCTCAAAA

>B3:16714593-16714893|varpos=16714743

ATGTGTCCAGGTAGGTTCATCAGTTGTAACAAATGTTATCACTGGTGGAAGATGTTGATAAGGTGGGAAGCTGGGTGAGGAGGGGGTCAGGGGTATACGGGAAATATTTGTAANCTCCTCTCAATTTTTCTGTGAACCTTCAACTATTC[TAAAAAAAAAAAAAA]AAAAAAAATCCNTAATTTTAAAATCCCATTATGAAAAAAGGATAAAATAGAACTCTTCTGATGAGAAGGAGTTAAATCCCCTACCACCACNAGGGTGCCATCAGAGGACACACGGCACACAGTTTGAAAACCACCACCCTAAAATAAATT

>B3:16797216-16797516|varpos=16797366

CNCCCCAGGTTTCCATCGTCTGTCTTTTCCACATGCTAATTCTCTCCTGTTAGCCTTGGCCGCCCTCCATCCTTGGGTGGGGGAGGGGGGCTTTGCTTCTAGGCTGTCCCACTACATCTCCTCCAAACNNACTCCCCATATGCACACAG[CTGTGTGTGTGTGTGTGTGTGTGTGTGTGTGTGTGTG]NNNNNNNNNNNNNNNNNNNNTTGCAGGAAGGTTTTCAGAAGGAACCTTCTGTTCAAACAATTTCTCCCAGCTGTCTCTGCCACTAGCACCTTCTCTTCTTAAAAACAGTTCTACCGTAGGGATGAG

>B3:17546211-17546511|varpos=17546361

CTCGTCTAGGCCAGAATTGAAAATACTCNCAAATCGTATTATCCTTAGTTCTCAGGANGGCAGTTGACAATAACATCCACAGAAATTCAGAACTATTTTAAGGGGATAATTTGGGGTTGAAGATTTGGGTAGAGTGATTTTGTNNNNNN[CTTTTTTTTT]TTTTTTTAAAGTTAAAAGTAAATNAAACATGATGGTTGTGGCATAGCGGACCATGGTGATGTGACAGGTGTCCACAGTCAGCAAATATCTATTGGCTGCTCGCTCCTAGGTCCTCAGCACTGNGGTGGGGTTTCAGTATTCAAAGAGCTG

>B3:17964157-17964457|varpos=17964307

GTTTTATTCTTTTTGATGCAATTGTAAATGGAATTGTTTCCTTAATTTCNCTTTCTGATAATTCATTTTTAGTGTAGAAATGCCACAATTTTTTATATGTGATTTTGTATCTTGCTACTTTACTGGATGCATTTATTAGTTTCAAAAAN[AATTTTTTTTTT]TTTTTTTGGTGGAGTCTTTTGGGTTTTCTATATANNNNNNNNNNNCTGCAAATAATGACAGTCTTACTTTTTCCCTTTCAAGTTGGGTGACTTTTATTTCTTGTCTAACTGTTCTGGGAAGGACTTTCAGTACTATGTTGTATAAAAGTG

>B3:20815929-20816229|varpos=20816079

GCCTACCAGGGCCCTGCTGGTGGAGACGTCAGAGTTTGCACCTGGAAGCTCTNTGGCCTGCAGTCCTCTATCTTGCTACCTCCCCTCCCACATTCAGTTACCAGCTNACTAGACAAGATCCCNTAAGACTCTTCTCCTGAAGCNNNNNN[CCACACACACACACAC]ACACACACACNTCCTTCCCCTGTGCTGGGCTCCTCTGAACCCAGGCTCAGCCTTCTCTCCTCNCTGCTAGGGCTATNACANGTGCNTGTTTANGGGAGTGTCTACTCTGTGGCCCCCTGATGNCTTCCTTTGGCCTATCCTCCCCTCAG

>B3:21064464-21064764|varpos=21064614

TCATTCTTTCTACTATGTGAGATACCGTTGATGTCTGATGGAAAAATGGATAAACAAAATGCGATGTATACTTACAAANGAATATCACTCAGCCTTAAAAAGGAATGAAATCCTAAGCATATAAGGAACTCACACAACTCAGCAAAAAA[AAAAATAATAATAA]ATAATAAACTGATTTAAAAATGGGCAGAGGACCTGAACAGACATTTTTCCAATGAAGACCTACAGATGACCAACAGACACAAGAAAAGGTGCTCAACATCATTTATCATCAGGGAAATGCAAATCAAAACTACAGTGTGATATTACCCT

>B3:21348864-21349164|varpos=21349014

AAGTCGACCCGATCTAAGACTAAAGCATCCAGCATGGCTTGCTCCAGAGCATTCACCTGTGCAAACCAACACCAGGGACCCTGTGAGTGGCNTCTCAGAGGGGTGCCAGGCNAGAGGCAGACNGGAAGCATCTGGGAAAGTGCATTCTG[ACCCCCCCCCC]NNNCCCAGACCCTCGAGCAAGTTGAACCTGGTTCTCTTTGCTCTTTGTCTTGAGTAAACANGCCCCACCTCCCATCGTTTTTCTCAATTTGGCCCCTACCTGTCCTGTCAAAGTATCAAAGCTTGATAAAAGGAGAAAAGTAGATGCTTC

>B3:21816433-21816733|varpos=21816583

GCAATTTCATAATTGCAACTAAAAGACAAAATACTGAGAAATAAGTTTAAAAGAGATATGTAAGAGATCCATGTTCTTAGTTTGGAAGACTCAANNNNNNNNNTTCTTCCTAAGCTAATTTATAAATTTAACACGATCCCAATNNNNNN[AAAAACAAAACAAACA]TAAGCAGGGCTTGGAATCAGACAGGCTCATTTTAACCTTCATCTGGAAAACTAAGCAAGAATAACCAGGGACTCTCTGAAACAAAGGAACAATGAGGAGACAGGGGCACTCACAGACATAAACCTGCTCTACTGTCTCGATAATACTACA

>B3:23422039-23422339|varpos=23422189

GNGNNNTACTGTCAGTTTTTCTCCATTGAGGACGATATTAGCTGTGGGTCTTTNNTATATGGCCTTTNTGATGTTGAGGTATGTTCCATCTATTCCTACTTTCTTGAGGGTTATTTTTTATCAAGAATGGATGCTGTTTTTTGTCAAGT[ATTTTTTTTTTTT]NNNNGGCATCTGTTGAGCAGATCATGTGGTTCCTATCCTTTNATTCATTAATGTGGTGTATCACGTTGAATGATTTGCAAATATTGAACCAGCCCTGAAAGCCCAGGAANTAAANCCCACTTGATNGTGGTGAATAATACTTTTAATAT

>B3:24859675-24859975|varpos=24859825

AGAGACAAAAATCTCATGGCAGGAATTTCCCAGGAAGAGGAGAACAATCCACATTTGAATTTTCATTCAACAACAAGGAGAAACTAGAAGGACACCTAAGGAAACAAGTATATGATACTGAGTTCCACAGTGAAATGAGTTTANTTTTA[CTTTTTTTTTTTTTT]AANNNNNNNGATTAGTTTCTTGGACTAGTAACTACCAGAGTGTATGAAATACACAACTGAGCTTTTTGTAAAGTATTTCCAAGTATTCTGTTGACGAAGTTGAGATAAAGGGCAATTGGAAGCTGGGCTAAATGACATGTATT

>B3:26824040-26824340|varpos=26824190

CTTGAATGGCCTNGGCCACTTTGCCTGTTTTTTATACTTTACATAAATGGAATCAAGCAGAGGGTGCTCTCTGAGGATTTACTCATGTTGACATAGTCTTTAGAGGAGAACATACACATGAATGCCATTTGTAATATTGATAAACCTCA[CTTATTTATTATTATTATTATT]NNNNTATTATTATTATTAAATAAGCTCAGTGTCATCTTTATTTGAATTACTTTCCTTTCACTTGTGCTCTAGATAGNTGTGACNTGATTTTATCTTTCTTNATTCCNTGAACTATTAAAAAATACTGACGTGCATTGAAACAACANNNNTTTTA

>B3:27061540-27061840|varpos=27061690

ACAGGTNTTAACTAAAGGCTCATAGCAAAATTGCAGGCCTCTATAATTATACATTCTTTGGAAAAAGTCTCCCTTCNTCTCTAGGCTGCACAGGACAAAAAGGATTCTCTGCTATATTTTTTACATAGTCTGTGACCTATTCTTACCAG[ATTTTTTTTTTTT]ACAAAATCAAAGTAACAGAGGCTGGGACACATTCTATTATAATATTTAATGTACCTTTATTAGCAAAGCAGTCTGTTCTCCAACATATTCTATTAAGTGGAGATGTGCCAGTGCCTAGAAGAAATGGATATATNATGAAAAT

>B3:30779511-30779811|varpos=30779661

CACTTTCCAGCCTNTTTATACTGCTTCCCCTTCTACCATGAGATCTGTATTTAAATTTAGTGTTTTCTACAAATAGAGTACAATTTAGATTCTTTTCCACAGAGCTTCCATCTGGTCTATCCTGCTCTAACCAAACCAAAAGTNNTTTT[TTTTTCTTTCTTT]NCTTTTGACTTAACATCTCTTTTATGTGAAGTGGCATGGGGTATGGAGNGAATGAATGCTGAGTTTGGTAATAAACACAGCTGTGTGGCTTCAGTTTCCTCCACTCTANAATATAGATAATACATACCTTTTGGATGATTACTGAGTA

>B3:31342781-31343081|varpos=31342931

CTAACTAAAGAGCTAGGAGATCTGAGTTCTTGCCTTTACTCCAGGCATTCACTAGCTGTGTGACCCTGGGAAAATTGTNCAATTTTCCTAGTTTTCAAGGTGGAGAATTTTTTAGAAAAGTGAACTTTTCAGTGAAGTATAACACACNN[ACGCGCGCGCGCGCGC]NCNCNCNCNCACNCACACACACACACACACACACACANACACACCTCTAAAGTGAATAGCTCAATTTTTACAAAGTGAATNTACCTGTGTTGCCAGTACCCAAATAAAGAATGAGAACATGACTAGGGGTGTTGGATCTTTAAATACAT

>B3:32163515-32163815|varpos=32163665

AAAAAAGTAAAGAGTTAAGTATCTACAGACTAGGAAGTAAATTTACAGTACAGATTCAACTACTTATTCACTAGTATAATAGAAAGACAGGAAATACNTGTTGAANAAAGTGCCATAAATAGAAAATAAATTGAAACTGGATTTTTTTT[TTAAAAAA]AANGTCTATTCTAAGATCACCCTTTTATCTCTCTCTTTTAAATGTAAAATATACNCTCAACCATTCCATATACTCTCAACCACTAAGTCTAAACACTAGTAAAGGGAGATTTTCAAGGTACCCAATTACAAAGTCAAAACTCTGCATCCA

>B3:34467218-34467518|varpos=34467368

GGCTCTGCTCTGTCAGCAGGGAGCCTGACACGGGACTCAATCTCACGAATCGCAAGATCATGACCTGAGCCGAAATCAAGAGTCAGATGCTCAACTGACTGAGCCACCCAGGTGCCCCTAAGTTTTCAACTTTTAGCAGCTNNNNNNNN[AAATTTTTTTTTTT]TTTTTTTTCATTTAAAAATCNAGTAGAATCTCAAGTAGAGTTGTTTAGGGGGAGAGGGAGATTCCTAATTGTTCCCCAGAATCCTTAGGGTTGGAGGAGCAGAATTTTAAAATTGATGAAATGGGTGACCTCTTTGTGGACCTACTACNG

>B3:34614637-34614937|varpos=34614787

CTAACTTCAGCTCNGGTCATGATCTCGTGGTTCATGAGTTTAAGCCCCACCTCAGGCTCTGTGATGACAGCTCAGAGCCTGGAGTCTGCTTCAGATTCTGNGTCTCCCTCTCTATCTGNCCCTCCCCTGCTCGTGCTCANNNNNNNNNN[GTCTGTCTGTCTGTCTCT]NNCTCTCCCTCTCTCTCTCNAAAATAAATAAAAAATGTTAAAAAAAAATTATCCACTCTGGGTGTCAAATGCTCTAGGCACATCACTTAACATAATAAATAAATAANNNNNNNNNNNNNNNNNTCTAGTATATCAGAAAGTGCTAGGAGGTAGGGAAATAGT

>B3:35334260-35334560|varpos=35334410

TTTTTTAAATCTCAACTCTTTTACGGTTACTTACTATAGAACAGGGGTCAGCAAACTATGGCCCACAGGCCAAATCTGAGCCAAACNGGCTATTTTTGTACNGCTCTCAAGCTAAGAGTATTTTCCTATTTTAAAGGGTTATAAAAAAA[AAAGAGAGAGAGAGAGAGAGA]AGAGAGNNNNAGAGAAGATGAAGAATATGAAAGAGACTGTGGCCGACAAAGTCCAAAATATTTACTATCTGGCCCCTGCAGAAAAAGTCTGTCAACCCCTGTCTAGAATCTTACTAGGCAAGCTAAGGGGAATAGGAAGATTTCCAGGCA

>B3:38690017-38690317|varpos=38690167

CCTTGTAGTACCATTGCTGTGTTCTTCNTNAAGGGTCCCCAGATGCCCCTCCATTCTGGATCTGTGCATTGATTCAGATCTTGGAATCAGCAGTAGGGTTATGCCTCCCTAGAGTGAGGGCTCCATTTACTAAACCCAGCCTGGGGTTC[ATTCTTGTTGTTGTTGTTGTTGTTGTTGTTGTTGTTG]NTTGTTGTTGTTGTTGTNGTNGTNGCTATTGTTCAAATTAAGTAGAACCTTAATGGGTTGCCCCTATTATGGTTGATCAGAGGGGAATAGTTAAGGAACTAAGGATACCATGGAATACTAGTGAAAAATAACGAGGTATATCTAAATA

>B3:40460093-40460393|varpos=40460243

GATTTTGGCTCAGGTCATGATCTCACAGTTTTCTGAGATCGAGTCCCATGTTGGGCTCTGNGCAGTGTGGAGCCTGCTTGGGATTCTCTCTCTCCTTCTCTCTCTGCCCCTCCCATCCCTCAAAATAAATAAAGAAACATTAAAAAAAA[AAAAAACC]CTGTTAATAGTTTAAGCCANTATGGCTTAAACCAACAACAAGTTTATTATTTNATCATTCTGGAGGTCAGAAGTCCAAAACAGGTCCGTAGGACTGTGTCCTTCTGGACGTCAGAAGGAAAGAGGATTCCCTGTCTTTTCCAGCTCCCNC

>B3:40964522-40964822|varpos=40964672

GTCNGTGNGTTCGAGCCCCNNNTCGGGCTCTGTGCTGANNGCTCAGAGCCNGGAGCCTGTTTCAGATTCTGTGTCTCCCTNTCTCNGACCCTCCCCNGTTCATGCTCTNNNNNNNNNNGTCTCAAAAATAAATAAACGTTTAAANNNNN[AAAAAAAAG]GTCCTTTCTAANTTGACAATGTAAATTCAGGGGAGTGNGGTGACTTAGTTTGAATGTNAATGGTTAGNGGCACAGTTAGGTACCCTAAGAGCTATTACCCAACTCTGTGTCAGCCATCTAGGAATTCANAGCCCCAAGGAGGATNAGAAC

>B3:42519343-42519643|varpos=42519493

ANGGAACACAGTGTCCTGCATATGCAAAAGCACTCAATTCACACACTGACTAATCTTCCCTGAAGATTAAGTATGATACCAGTCAGCAAACCTAGAAAATAAGAGTCAAGGATTGGGACCCTCTTCCTCTTGTGAAAGATTTTTTCTTT[CTTTCTTTCTTTCTTTTTTTTTTT]TTTTTTTAAGTCCAGTTTGAATGTACTCCTTCAACAGACCTACATCCAGAGAGTTACATATGCACCCACAAACCAAGCTGCTGGACAGGTCTGCAACAGAACTCTGGTTCTGAAACAGTTTACTGAATGACCACTGCTATACCTGTA

>B3:44266630-44266930|varpos=44266780

ATTAAAATTGTAAAAAAANCTATTCACCAAGGAACAACNTTGAGAGTGAAAGGTAAGCTCCAAGATAAAAGATANATGGAAATGAAAAGGACTTCCTACAACAAANACAAAGATAAACAAACATNAAAAATGCAACTNTGAGTTATTTT[TGAGAGAGAGAGAGAGAGAGAGAGAGAGAGAGA]NNNNAAGGGAGGGAGGNAGAGGGAGAGAAAATGAGCCAGGNAGGGGAGAGAGAGAGGGAGACACAGAATCNGAAGCAGGCTCCAGGCTCTGAGCTGTCAGCACAGAGTCTGANGNGGGGCTGGAACTCAGGAAC

>B3:45897902-45898202|varpos=45898052

TTAATTATGGGAAATGGAATGATATAGTGAAGTTAGACCTGTATCTCTGAAGTTGTTGACTGTTGCTTTCTCAGGAGCCTTTTTAGGTATTGTTTTTTCCTAATTGTCCTCCCCATGAAATTTTAGTGTCACAGAAATAGTGTGTATTT[GTTTTGTA]GTATTTTTTTAATTTTTTAATGTTTTTATTTATTTTTGAGACAGAAACAGANCATGAGTAGGGGAAGAGCAGAGAGAGAGGGAGACACNGAATCTGAAGCAGGCTCCAGGCTCCNAGCTGTCAGCACAGAGCCCAACGTGGGGCTCGAAC

>B3:50012192-50012492|varpos=50012342

TTGTGAGTTTGAGCCCTGCATCAGGCTCTATGCTGACAGTTCAGAGCCTGGANCCTGCTTCAGAGTCTGTGTATCCCTCTCTCTCTGCCCCTCCCCTGCTCACACTCTGTCTCTCTGTTTCTCTCAAAAATAAATAAACATTAAAAAAA[AAACCC]CCAATATCATTTCCTCCAATTTCTGTATCCCAAGCCAACCTATTTAGCCATTCTCTTAACTGGCATTTCTACTTCTTGTAAGGAAAACCTGACCATTTAGATGTCTGTTAACTACTTGNAGACAGTTCCAGGCTTTAGATTTCATTCTTT

>B3:50981910-50982210|varpos=50982060

AAAGCAATCACCACAATAACTCCAGTTCATGTGTTATCATATGATTAACCCCCTTCACCCATTTTGCCTACCTTCCANTCCCTTCCCTTCTGATAACCACCAAACAGTTCTCTGTATCTATGTGTTTTGTTTTGTTTGAGACNCNCACA[CACGTGTGTGTGTGTGTGT]GTGTGTGTGTGTGTTCATTTCATGTAAATGAAATCATACAATATTTGTCTTTCTCTGACTTATTTTACTTAGCNTAATACCCTCAAGGTCAAGGTCCACACGTGGTGTTGCAAATGGAAGAATTTCCTTCTTTTTATGGCTGAATAGTAT

>B3:51546803-51547103|varpos=51546953

AGATAGTTTTGACAAGGTATATAAATTTTTTTTTTNNNNNNNNNNNAATTCCTAAAATTGTTTCCTCAACAACGTTTTTTGCCTTTCTTGGTGTTTTTGGGTTATCCATTATCTGTGATGAAGTGCAAGACAATTTAGCTTTTTGGTTNNNNTTTTTTT[TTTTTAAA]AATATGGCAAAGTNAAGTACAACATAGTCGACANTTTTACCTCTCNNGAGGTATATAGCTCAGTGGCATTAGATGCAATCACATTGTTGTATAACCATAACCACCATTCATTTCCATATAGTCTATCCTTAGCAAGAGAAATCACTTGAC

>B3:51779800-51780100|varpos=51779950

GTCTAATTCCAAGTCTTGCTGTTAATAAGTTTTGCTATATTTAAATANCTTTTGGGAAGAATAAATGTNNAATTGCTATATTGTCCACCTGAAACTAATATTACCCTGTATGTTAACNAGCTGGAATTTAAATAAAAAATTGAAAAAAA[AAACCCCACC]CCTCTTTGTGAAGAATAAATGATATACTGTATGTGAAAAAGGTGTTTTGTCAATGTTACACATTAGTTAGCTATTGTTGGAATTTCTAAAGTGATTAATTAGCTCTGTGGGTACTTGAAAAGTAGCTCANTTTTTGTTATATTGAAAGA

>B3:52094691-52094991|varpos=52094841

GGGACTGTGTAAAGTCTTCTTGGATTCACAACCAGCCCTTAAAAATTTAATTAAAACATTTCAGATGNTATGACTAAAANATTTTGAATCTGGCAACCACCAGNTTGTTCTCTATATTTATAGATCTGAATCTACTTTTTTGTTTATTC[TTTTGTTTTTTTTTAAATTT]NNNTTTTTTNNTTTAATTTTTATTTATTTTTGAGATAATGCGAGAAGCAGAGTGCAAGCGGCAGAGGGGCAGACAGAGGAAGACACANAATCCGAAGCAGGCTTCAGGCTCTGAGCTGTCAGAGCAGAGCCCGACTCAGGGCTCAAACTC

>B3:52147221-52147521|varpos=52147371

AAACCTCTTTAGCCAAATACAGATTACAGATTACNAAGGGAAGGATGCTCATATCCTAAACAATGATTTTGTTAAAAATCTGTCCTCTAATANACTATAATCTTGGGTGAAATGCTATAATATGTCACTTTCTGAACAATTAGNNNNNN[CACAAAAAAAA]AAAAAATGAATTGTCTTTTCTACATAACTATATGAGAAAATTAAAATGAGCCCAATAAATAAAGCATTCAGGCTTCCTGCCAAAGGCAATGACTAGTGTTTTGAGACAATCAATATTCCAATCAACCAAATGTTAATACAATNGGAACA

>B3:52254830-52255130|varpos=52254980

GGCTCCACTCATGATCTCATGGTTCGTGAGATCAAACCCCACATGGGGNNNCTCTATGCTGAGTGTGGAGCCTGCTTGGTATTCTCTCCACCTCTGTCTCTGCCCCTCCCTGATAGTGCTTGCATGCTCTCTCTCAAAANNNTAAACATTAAAATAA[AATAAAACC]CAAAAAGAAAATGAAATGTATTTACTAAAAACACATGATAGTATGTTCAGAATATAAAGAACTCTTACAGGTCTCTAAAAATTTGAGCCTAATATCCCCCCAAACTGGTCAAGAATGTGACTGGATGATTCACAAAGTAGAATGCATGAA

>B3:52638473-52638773|varpos=52638623

TGTCATTCAAAATAGAAGGGGAGATTAAAAGTTTCCCAGACAAAAAATAAAGGAGTTCATGACCACTAAACCAGCCCTGNNAGAAATNTTANNNNNNNCTCTCTGAGGGGAGAAAAGCAATAAATGAATGAATGAATGAGCAAATTATA[AATACATACATACATACATACATACATACATACATACATACATACATA]AATACCAAAANCAACAAAGACTAGAAAGGACCAGAGAACACCATCAGACACTCCCAACTCTACAAGAAACATAATGGCAATAAATTCATATGTTTCAGTACTC

>B3:53722453-53722753|varpos=53722603

ATTGCTTAAATTCNTTTGTAAGAGAACCAAAGGGAGCAGATGGTCTCAAGCAGGGCATCAATCATAGGGTAAAAATATGGAAAAGAATGATAGAAGCCAAACTTGGTTCTAAATTGTTACTAAATTTCTGTTCCTTTCAGGTTTTTTTT[TTTTTAACTAA]AAAGTTCTCTTGTATGTCATTCTTAAGGTTAGTATAAAAGGGACATCGTGAAAAGCTAAGAATCACAATATAGACCTCTACAGGCAAGTTTAGCTTCCCAGAGGGAAAACTAACTGAAACATTCAGTTTTGTGTTTTTTCCTGGTAGTT

>B3:54556551-54556851|varpos=54556701

AGCACCATTAACCCACACATGGGCTGTATCCTGTAGGTGATCCTTTTGGCCAAGGTAACAGGCAAGTCTGCCACGCAAGAACTGAGAGTTGCAGGAAAAGGGGACTTCAGAGAATTCATTTGAAAATCAAAAGCTGAGATGCTCAAAGC[ATTTTTTCTTTTTTT]AAGAGAGAGTGCAAGCAGGGGAGNNNNNCAGAGGGAGAGAATCTTAAGAAGGCTCNATGCTCAGTGCAGAGCCNAACACAGGGCTCGATCCCGATACCCTGGGATCATNNNCTGAGTCAAAATCAAGAGTTGGACCCTCAACTG

>B3:57018321-57018621|varpos=57018471

AATATTCCTTATTTTGTATTTACAAACGAAATGCTTATTATGCAGTCTTAAAATAATACCTGAAGTAAAGTTTTAAAAAAATGTATTCAGCTTTTGATAAATNCTTGGAATTCTGCCTATTTGTTAAGTTNTTCTACATGATTNNNNNN[AAAATTTTTTT]TTTTTTAACGTTTACTTATTATTGAGAGAGAGAGAAAGAGCATGAGCATGAGAGNGGCAGAGAGAGGGAGACACAGAATCCAAANNNNNNNNNAGGCTCCGAGCTGTCAACAGAGCCCGATGCGAGGCTTGAACTCGCAAACTGTGAGAT

>B3:57119004-57119304|varpos=57119154

TTTCCTGTGCTCCTTATATTGGATAAATTTCTATTGATTAGTGTTCAAGTTTCTTGATTCTTTTCTCTATCTTCTCCATTCTATTAAACTCATCAAGTATATTTTTAATTTCAGACATCAAATTTAGAATTTTTTTCATTCTTTTTTTT[TGGGGGGGGG]GGTTTCTATTTCTCTGCCTTTTCATTATGGTCATATTTTCCTTTACTTCATTGATCAGAATTATAATACCTGCTTTAAAAATTCTTGTCTACTCATTCCAACATCTGGAACATCTCAGGGTTTGTCTCTATTGATTGTCTTTTTACCTGA

>B3:58158318-58158618|varpos=58158468

AGCTCATGATGTCATGGTTTCATGGGTCTGAGCCCTGTATCCAGTTCTGGGCTGGCAGCATGGAGCCTGCTTGAGCTTCTGTNTCCCTCTCTTTCTACCNNTCCCCTACTCANATTGTCTCTGTCTCTCTCAAAATAAATAAATAAACT[TAAAAAAAAAAA]AAAAAAAAGGNAGTATCATGAAACTCACCCCTAAAAAATAAAATACTTAGATAAAATAACAAAATATGTANATNATCTGTGTGATGNAAATTAAAAATACTGATGAAAGAAATTAAAGTAAACANNNNNNNNTCAGGAGATACACCATAT

>B3:58237469-58237769|varpos=58237619

AGCATTGTGAAAGTATCCAGAATCAATTTTAGTAGGTTAGCATCCTATCATAGAGTCATAGGACTGAAATAATCCCCCAACAGTAATATAATTCCAGAACTGGAAAAAAACACTAGAGATCATTTAGTCCAAGACCTTTAGTTTAAAAA[AAAAAAAAACAAAAAAAA]AAAAAAAANAGATTTGATTTAATAAGTTGTTGCAAGTTTAGACTAAAATGTAGGCCCTGTAATTCTTATTCCAGGATTCTTCTATACCATGAAGTTTCATCGTTAGTCATGGAATCATTAACCCATTTGAGACCCCTTTTATTACACAT

>B3:58880660-58880960|varpos=58880810

TTATAAAGTCATTAAGTAATGTATGAGGNGTAAAAGGGAGGTAGGAGTTACCTTGAGGAATGACTGAGGAAAAGGTCATGAACGCAGTGACTACTTTCTAAAGGAAAAGAATAAGAGATGAGAGACAGAGGGAAACGGTAAAGGATGTG[ATTTTTTTTTTTTTTTT]NNNNNGAAGCAGCAACTTGAGGGNAAGAGTAGTTCAAGAGTAATCTAGTGAATATTAATATGAGTCTTTCATTGTTTTCATTCAGTTTCTATCTCATTACCTCCATCAACAAATATCTTTGAGCAAAGATCTGTCATATACTCTTA

>B3:58957226-58957526|varpos=58957376

ATTAATNAAAACTGACATTTCAAATTTCATCTGTTATGTATCTGAATAGATACAGATTATATAAANAAAAGAGCACTGTAGAAATTTCCTGTATCTATTCTGCCATGTAGGACGACCTAAAAATGTTTTATTTATATTAGGTTAAAAAA[AAAAGG]GTTTTATTTAGGTTTATTTATTTTAGTTGGTTTCTGAGATATGATNGTGCCAAATTTTTTCTTATGTTCTCTCAAGAATATTAATAGCAAGAAGATGGTAATTGTCTATAGATGNAAATTCTAGAGAAACATCAGTAGATACTNTAATCA

>B3:59410597-59410897|varpos=59410747

CTTTTTATTTAAATTCCAGTTTGTTACCATATAGTATATTAGTTACGAGTGTACAATTTAGTGATTCAACACTTCCATACAACACTCCTTAGTCCCCATCACCTATTTCACCCATCCCCCCCAACCACCTCCCCTCTGGTAACTATCNN[GTTTTTTTTTTTT]TTTTTTCTATAGTTGAGTCTGTTTCTTGGTTTGCCTCTCTTTTTCACCTATGATNGCTTGAGTAAACACTATTTTTTGAAGGCATATTCTGTGTTTAGAATTTTAGATGTATTAATCTCAAAACAACACTTTGAATTACAGNGTACNA

>B3:59460497-59460797|varpos=59460647

CCAGGTTAGAAAAGTTTTTAGNCATTATTTCCTNAAATAAGTTTTCTGTCTCTTNCTTCTTCTGTGGGTCCCCTGTAATGTGAATGTTACTCCACTTGATGTTGTCTGGTGGGTCTGTTAAACTATCTTCANNNNNAAAATTCTTTAAA[AATTTTTTTT]TTTTTTGNTGCTCTGTTTGGATGAATTCTATTNCCTTATCTTTGAGATCACTGATTATTATTTTTTTCTGCATCCAGTCTACTGTGGAACTCATCTAGTATATTTTTCAGTTCAGTTATTGGGTTCTTAAGCTCTATGACTTGTGTTTGG

>B3:59579502-59579802|varpos=59579652

AAATATTACTGTACTGCCCTTACTCTCCAAGCACACAGCCTGCTGGTGCAATCAATTTCTGGGAGCATGATTGACCTATTTTTCTCTGCCATTCAGACTTTCTCTAAGTAGACATATCTCTAACCCTTGGCATATAAATTCTCTANNNN[AAAAAACAAAAAA]AAAACAATCCAGATACTTCATAAATGGCTAGGTTGAATGTAAACTGGAGATAGCTCTCCCTTAAGAATCGTTAAATAGGGNAGGTGGGAAAAACATGGACATTACCACTATGATACCAGATACTTAGTGAGCACTAATGTCTATTTG

>B3:59876385-59876685|varpos=59876535

TTAACAAAATTGATAAACCCCTAGCCAGTNTGATCACAAAGAAAAAGGAAAGGACCCAAGTAAATACAATCACAAATGAAAGAGGAGANATCACAACNAACACCACAGAAANACAAACAATAAGAAGAAGAAATACNNNNNNNNNNNNN[AATAATAATAATAATAATGAT]GAGCAATTATATGCCAACAAATTGGGCAATCTGGAAGAAATGGACAAATTCCTAGAAACATATAAACTACCAAAACTGAAACAGAAAGATATAGAAAATTTGAACAGATCCATAACCNGTAAAGAAATTNAAGCAGTAATCCAAAATC

>B3:60015172-60015472|varpos=60015322

GTCATGGTTCACAAGTTCAAGCCCCAAGTCAGGCTCTGTGCTAACAGCTCAGAGCTTGAGGCTGCTTCAGATTCTGTGTCTCCCTCNCTCTCTGCCCCTCCCCCATACATNCTCTGTCTCTCTCCCAAAAATAAATAAACATTAAAAAN[ATTTTTTTTT]TTTTTTAATTACTAAGTTCTCTTGACTGGCTTGGAAAGAAAACTCCATGCAGTCTGTTTAAGTTATGGAATTAAACTTTGAGGCCATAATGNACAGACAATAATTTCTCTACTTAGATGAGCATCTATCTNNNNNNNNNTCAAGGACCAA

>B3:60867490-60867790|varpos=60867640

AACCCCAGTGGCTCACCTGGTTTTATTTCTGCTTCCTCCTTTTTTTCATCATCATCATCATCCCAGTTATCCTNAAGAAAAGGACTTCTATTAATAATTCTTGCCTGTTTACTTTTCCATGTGCAACTTATCTAACTCCACAGAAAGAN[GTGTGTATTTT]ATTTTTTTTGTACTGCATTAAAATTGTAAATTAATGTTAGGAAAATGCACATCTTCATAATGTTGAGCCATCCTAGCCAAGGAAAAGGGCTGTTTTTCCATTTGTTCAAGTCTATTTCTGTATCTTTCAAGGAATGTTTTAAAGTTTCC

>B3:61281614-61281914|varpos=61281764

CAGAACAAGTTACTTGCAATCCAAGGTTTTACTGTATTTGTCTTTCTCTGATTGACTTATTTTGCTNAGAATTNTACTCTCTAGCTCTAGCCCTATTGTTGTAAANGGCAAGATTTCATTTTTATGGATGAATAATACTNCATTGTGTN[TACACACACACACACACACACAC]CACACACACACNGCATCTTCTTTATCCATTCATCAATCANTGGATACTTGGGCTCCTTCCATAATTTGACTATTGTAAATGATATATAGAGATGCATGTATCCCTTTGAATTAGTGTTTTTATATTCCTTGANTAAATACCCAATAGTA

>B3:63964345-63964645|varpos=63964495

TCATGCTGCACANTGAGTGTGGAGATTACTTAAATAAAATATTTAAAAACAACAACAGTAAAGTTTTGATAGGAAACATGCAACTTGAATCCAGATTCCATGTTCAAGATCAATTTTAAAGGTGAANTTTGGGGTGATTCATTTTTTTT[TTTCC]CTTATGTAATCTTTATGCCCGCTGTGGGGTTCAAACTCATGACCCTGAGATCAAGGGTTGTATGCTGTACCAACTGAGCCAGCCAGGCACCCTTNGGATTATTCTTAAGTGNACAGACCTGAAAGAAAAGATGAGGGAAAATGGTAAAAT

>B3:65748651-65748951|varpos=65748801

CATTTACCCAGACTAGGGTGGTGANTCCTTTCCCTGCCTGCTGGNCTCTTGGCCAAGGAACCTGAAGTAACTGGGTGATTGTCATACCTTAAGGTTTTATAAAACTCTTTCTGTGTTTATTCCCTTATAAAGATGTTCCTTCTTTGGNA[AAAACAAACAAACAAACAAACAAACAAACAAACA]NCATTTCCAATGTTGATCCAACCACAATGTTGATCTATTTTATTCTTAATATGTTTCAGANTGACACATAAAAATAATTGGTTTGAANCTGTTTTTTCCTTTTAAAAAGAAATTCATTTATCCTNNNNNNNNNCACTA

>B3:66647765-66648065|varpos=66647915

CTCAGAATCTGGAGCCTGCTTTAGAATCTGTGTCTCCCTCTCTCTCTGCCCCTCCTCCACTCATGCTCTGTCTCAGTCTCTCTCTTTCAAAAATAAGCAANNACATTAAAAAATTAAAAAAAATTCTGATTTCAAAAATAAACAAACATTAAAAA[AAATTTTTTTT]TTTTTAATTCAACTAAATTTCTCAGAGGAGAAGATATTCGTGGCCAAGAGTGGATGGATAGTCACCAAACCTGTTTCTTATTTTTTCTGGGCTCACANTGAGACTGCAGTTCTCAACTTTCCTTGCAGTGAAGCACTGTCCTATGTCTGG

>B3:67257795-67258095|varpos=67257945

TGGTTAAGCATCAGACTCTATTTTGTTTCAGGTCCCGATNTCACGGTTCAGGAGACAGAGCCCTGAGTAGGGTTCTGTGCTGATAGTGGGTTTTCTCTCTTGGGGTTCTCTCTCNCNCTCTCTCTGCCCCTCCCCTGCTCTCTCACACA[TTCTCTCTCTCTCTCTCTCTCTCTCTCTCTC]AAAATAAATANATAAAATTTAAAAATAATTGTAAAAAATAATTCACTGAAAAAGGATTTTGTGAAACATCATAGAGAATGATCCCTGTTGACATATAGTCAGAAAAATAGGCACCATTCCCAACCTTTTATTTC

>B3:67271876-67272176|varpos=67272026

TTAGTGATCAAAAGAGAATCTAGATTTCTGGAATATTAGCCTTAGGCTCTTATTTAAACATTTATTCATTATTTATGTTGTACCAGTTAATTTGCCAAGTGCNTTACTAACATTATCTCAATTAATCTTCACAAAAACAGTATGGTNNNN[ATTATTATTTTTTTTT]TTTTTTNAACTTTATTTATTTTTTTNNNNNNGAGAGAGAGCCTGAGCTTGAGTNGGGGAGGGGCAGAGAGAGAGGGGACAGAGGATCTGAAGGGAGCTCTGTGCTGTCAGCAGCGAGCCCAACATGGGGCTGGAACCCAAAAACCATAAGATCAT

>B3:68100681-68100981|varpos=68100831

AGGCTATGAAANGTTGTCTATGTGNNTGCAGAAAAATAAAATGGATTTGAATGGATAGCAGTGGTCTTTGCACCAAGCAATAGCCCTTTTCCTGAAGTCTACATACCTGCATTTATAAATTTATATAGAGGTGAAGCAGTCTTTTNNNN[GGAAAAAAAAAA]AAAAAAAAGGGATCTTGGATCTTTTATTCTCAATCCCCACCTAAGCTTTAAGATTGTTAAGAACACATTTAATGAACTGTGAAGGCTCAGATCAAGACTGTATTTCAGGAAGAAAAATGCACTCTCTTAGAGAAAACCTTTNTGAGGAGT

>B3:69177398-69177698|varpos=69177548

AGCTATNTTTTGGGTATAACCAAAATGATTTGTATCTTGATTGTGGTGGCAGTTTCACNGGTATATACACATTTGTTAAAACTCATTGAATTGTGCACTTTAAATGGATACAGTTTATTNTATGTAAATTGCACCTCAATAGACATAAT[TAAAAAAAAAAAA]NNNTTCCTACNGGGAAGGGCTCTCCATTGCCTTCAGGATGCAGGNCACATTGCCCAACAGTTTGATCCCAGGCTACATTTTGCAGCCTTTTTTCCTTCCACTTCTTGATATGTGCTTTCAACAGCAGCTACTCAATACAGCTGAC

>B3:71350357-71350657|varpos=71350507

GTAGGTTTCAAGCCCAGTGTGAAGCCCAATGCCAGGCTTGAACNNNNAAACATAAGATCAAGACCTGAGCTGAGATCAAGAGTCCAAGNCTTAACCAACTGAGCCACCCAGGTGCCNCAGCATACACATTATTTTAAAATTNAAAAAAN[ATTTTTT]TTTATGGTTTTTAAAGTAATTANATACTGCAGAAAAGAGAAGCCAAATGGTCAACTTAGGCTAGTTTTCCAAGGCTTAAGAGAGTNTTTTCATTAAAACATCATTACTTAGGGCAAACTCTAAAGAATACCAGATTCGCAAAGACAAATA

>B3:72560618-72560918|varpos=72560768

ACCAGCAGAAGGAAGGAAATAATAAAGATTAGGNCAGAAATAAATGATACAGAAACTAAAAATAGAATTGATCAATGAAACCAAGAGCTGGTTCTTTGAAAAAAATAATAAAATTGATAAACTTCTAGTGAGACTTATCAAGAAAAAAA[AAAGG]GATAAAGGACTCAAACACAATCACAAATGACAGAGGAGAAATAACCAACACCACAGAAGTACAAACAGCTNTAAGAGAATATTATAAAAAACTATCTGCCAACAAATTAGACAACACAGAAGTAAGGGAAAAATTCCTGTAAATATATAA

>B3:72777706-72778006|varpos=72777856

GTGCCTATACTGCATGATTGTGGGACAGAGAAAAATATGTNTTATNTTTTGAGTGTNGAAATCTGAGTTNGAGAANCTACATTTCTAGTTGAATATGAAACCATTACTTTCAAAAGTTGTCAACTGGAAAGGCAAAAGAAAAATAAAAG[AAATAAATAAAAGAAAGGT]TAATTATATTGCCTTCATTTACTCTGTTCTACTATCATCACAGTATTTGTGACCTTATTCTAAACGACAGAATTCTCCATAAAGTTTATTGAANAAAAGTAAAACCCAGAATGGAAACTAATATCAATTTCCTTTCTGTTGCTTTTAGGG

>B3:73401448-73401748|varpos=73401598

CTCAGGTCATGATGTTGCAGATCTTGAGCTCGAGCCCCATGTCGGGCTCCATGCTGACAGTGTGGAGCCTGCTTGGGTTTCTCTCTCTCTCACTCTCTCTTCCCCTCTTCCGCTTGCTCTGTCTCTCTCTCAAAAAATAAATTAAAAAA[AAAATTT]TTAAATATAATNTTTTCTAAAAGTAAAAAAGAGTAACTATCATGAAAATTTTAAAAATAAATCCATGTTATTGAATAACTTCATAAATAAGCCCATTTTATTGGACTCATTAATTAATAAGGAAATCAGTAACATGTTAAAAATGGTTCA

>B3:73511696-73511996|varpos=73511846

TGTGAGTTAGAGCTTTGCATTGGGCTCTGTGCTGACAGCTCGGAGCCTGGANCCTGCATCAGATTCTGTGTTTCNCGCTCTCTGCCCCTCCCCTGTTCATGCTCTGTCTCTCTCCATCTCAGAAATAAACCTTAAAAAAAAATTTTTTT[TTAAAAA]AAAGAAAAGAAANAGATANAGGGGCATCTGGGTGGTTCCTCTGTCTCCCTCTCTCCCTGCCCCTTCCCCACATATGCTGTNNNNCTCTCTCTCTCTCTCTCTCNCTCAAAAATAAGGATTGGATAGTTCTCAAANGAGCTCCAAGGTGTA

>B3:78998305-78998605|varpos=78998455

AAGTATCACTAAAGTCTCTCTGCTNTGTCTGGATCAGAATTCCANCATCTCCCAGCAGTGCTTGATCAAACAGAGTTTATTGAATAGCGTTAAGTGACAGAGAATAGCAAATGGAATGTCTGGGTGACTCANAAAAGAAAGGAGAATGA[TTTGTTGTTGTTGTTGTTGTTGTTGTT]NTTGTTGTTGTTGTGGGGCTAGGGATTTATATTGGAAAAGGGTCTAGGATATGTGTTTCTTCAGGAATCCATGGTAGGGTCCAATCAAAGGCGAATATCATGTGAACAATAGGTGTTATTCCATATATTACTGAAAGTGGGG

>B3:79335313-79335613|varpos=79335463

TGTGTGTGTGTGTGTGTGTGTGTGTGTGTGTTTAGAACAGAAGGGTAGCCNACTGACATAGGATCATCCCTTTGATTAGANTAACCAGTACCATTTACAAAAAGTCAGCCTTAGCATATTTTAGGGGAAAGAGAGCAANGGATTATAAA[GTAGATAGATAGATAGATAGATAGATAGATA]AACNAGATTNCATGGCCTCCAAGAGCATTTCACAACAAATACCTTCACTGATTACATGTCAATATGCTCTACCCCTGGATAGGACAGGTTTCCTAAANGGAAAATTTCCAGACACTTGGAGTTATTTTTGATCAAAATAA

>B3:79805251-79805551|varpos=79805401

NNNNNNNCTTAAAATATATGTATTTCCTCTCAGGAATACTGATTTAAGGAAATATTCTCTTACAGATTAGAAACAACTATATTTTCACTATTGTTTTATCTTCTTCTCTACCTCAAGTCTCTGGTTTCATTCATCAGTAATCTGGTTAG[GGTTTTGTTTTGTTTTGTTTTGTTTTGTTTTGTTTTGTTTTGTTTTGTTTTGTTTT]TAATCAAGAATTTACCTAGATGAACTAGCTTGAGTACTCTCTGGATCCTTGCATATCTGTGAATATCTTTTATTCTGACTAGTGAATGATATATGGACTGTGTGTAGTTT

>B3:79850735-79851035|varpos=79850885

AGCAGTGGCTCCCAGGTCCTTAATATACACATTCGTGGGTTGTAAAACNGACAACAGGCTTGTAAAATGTTTTCTAAAGAAAATACCCTAAAAANAAAAGACTTCAGGGCCCAGTATCAGGAAGAAGCCNATAAAAAGTTTAGTTAAGG[TAAAAAAAA]GAAAAATGTTTGGTCAACNTGGTAAGATTAAATTTNAAAAAATCTCTATGCATTATTATTTAANNNNNNTCACTGTGCTAAGATCCTANCCCTGTTTTNAAGTATCCAGNTTTGTAAACTGTTGCTTTTAATGANAAAACTGAACATAAA

>B3:80987577-80987877|varpos=80987727

TTGGCAGAAATCTACACTTCAGAGATAATAACATATCATTCCANGAAATAAAGGAAAAGGCATAATCAAAAAAAGAAGTTAAATATGGAAACCTGTTTTGACAACAAAGAAATACAGATGAAGCCAGACAGGAAAGGTGTTATGTATGC[ATGGTTTT]TTTTTTCATGGTTCACATAAATTATGATATTGTAAGAACCNATCAAGTTTTCTGTTTTCTTTTTTNNNNNCAAATACTTTAAGTCAGATAGATTATTTATATTGTTCTCCATCTTACACCAAAGAAAAATTTCAAAGACTAGTGCTTTATATGAA

>B3:81125878-81126178|varpos=81126028
[truncated: 534,282 more chars]
